# Physics at the $e^+ e^-$ linear collider

**DOI:** 10.1140/epjc/s10052-015-3511-9

**Published:** 2015-08-14

**Authors:** G. Moortgat-Pick, H. Baer, M. Battaglia, G. Belanger, K. Fujii, J. Kalinowski, S. Heinemeyer, Y. Kiyo, K. Olive, F. Simon, P. Uwer, D. Wackeroth, P. M. Zerwas, A. Arbey, M. Asano, J. Bagger, P. Bechtle, A. Bharucha, J. Brau, F. Brümmer, S. Y. Choi, A. Denner, K. Desch, S. Dittmaier, U. Ellwanger, C. Englert, A. Freitas, I. Ginzburg, S. Godfrey, N. Greiner, C. Grojean, M. Grünewald, J. Heisig, A. Höcker, S. Kanemura, K. Kawagoe, R. Kogler, M. Krawczyk, A. S. Kronfeld, J. Kroseberg, S. Liebler, J. List, F. Mahmoudi, Y. Mambrini, S. Matsumoto, J. Mnich, K. Mönig, M. M. Mühlleitner, R. Pöschl, W. Porod, S. Porto, K. Rolbiecki, M. Schmitt, P. Serpico, M. Stanitzki, O. Stål, T. Stefaniak, D. Stöckinger, G. Weiglein, G. W. Wilson, L. Zeune, F. Moortgat, S. Xella, J. Bagger, J. Brau, J. Ellis, K. Kawagoe, S. Komamiya, A. S. Kronfeld, J. Mnich, M. Peskin, D. Schlatter, A. Wagner, H. Yamamoto

**Affiliations:** II. Institute of Theoretical Physics, University of Hamburg, 22761 Hamburg, Germany; Deutsches Elektronen Synchrotron (DESY), Hamburg und Zeuthen, 22603 Hamburg, Germany; Department of Physics and Astronomy, University of Oklahoma, Norman, OK 73019 USA; Santa Cruz Institute for Particle Physics, University of California Santa Cruz, Santa Cruz, CA USA; Laboratoire de Physique Theorique (LAPTh), Université Savoie Mont Blanc, CNRS, B.P.110, 74941 Annecy-le-Vieux, France; High Energy Accelerator Research Organisation (KEK), Tsukuba, Japan; Faculty of Physics, University of Warsaw, 02093 Warsaw, Poland; Instituto de Física de Cantabria (CSIC-UC), 39005 Santander, Spain; Department of Physics, Juntendo University, Inzai, Chiba 270-1695 Japan; William I. Fine Theoretical Physics Institute, School of Physics and Astronomy, University of Minnesota, Minneapolis, MN 55455 USA; Max-Planck-Institut für Physik, 80805 Munich, Germany; Humboldt-Universität zu Berlin, Institut für Physik, 12489 Berlin, Germany; Department of Physics, SUNY at Buffalo, Buffalo, NY 14260-1500 USA; Physikalisches Institut and Bethe Center for Theoretical Physics, Universität Bonn, 53115 Bonn, Germany; Physikalisches Institut, University of Bonn, Bonn, Germany; Physik Department T31, Technische Universität München, 85748 Garching, Germany; LUPM, UMR 5299, Université de Montpellier II et CNRS, 34095 Montpellier, France; Department of Physics, Chonbuk National University, Jeonju, 561-756 Republic of Korea; Universität Würzburg, Institut für Theoretische Physik und Astrophysik, 97074 Würzburg, Germany; Physikalisches Institut, Albert–Ludwigs–Universität Freiburg, 79104 Freiburg, Germany; Laboratoire de Physique, UMR 8627, CNRS, Universite de Paris-Sud, 91405 Orsay, France; SUPA, School of Physics and Astronomy, University of Glasgow, Glasgow, G12 8QQ UK; PITT PACC, Department of Physics and Astronomy, University of Pittsburgh, Pittsburgh, PA 15260 USA; Sobolev Institute of Mathematics and Novosibirsk State University, Novosibirsk, 630090 Russia; Ottawa-Carleton Institute for Physics, Department of Physics, Carleton University, Ottawa, K1S 5B6 Canada; ICREA at IFAE, Universitat Autonoma de Barcelona, 08193 Bellaterra, Spain; University College Dublin, Dublin, Ireland; Institute for Theoretical Particle Physics and Cosmology, RWTH Aachen University, 52056 Aachen, Germany; CERN, Geneva, Switzerland; Department of Physics, University of Toyama, 3190 Gofuku, Toyama, 930-8555 Japan; University of Hamburg, Hamburg, Germany; Kavli IPMU (WPI), The University of Tokyo, Kashiwa, Chiba 277-8583 Japan; Institute for Theoretical Physics, Karlsruhe Institute of Technology, 76128 Karlsruhe, Germany; Instituto de Fisica Teorica, IFT-UAM/CSIC, Universidad Autonoma de Madrid, Cantoblanco, 28049 Madrid, Spain; Department of Physics and Astronomy, Northwestern University, Evanston, IL 60091 USA; The Oskar Klein Centre, Department of Physics, Stockholm University, 106 91 Stockholm, Sweden; Institut für Kern- und Teilchenphysik, TU Dresden, 01069 Dresden, Germany; Université de Lyon, Université Lyon 1, 69622 Villeurbonne Cedex, France; Centre de Recherche Astrophysique de Lyon, CNRS, UMR 5574, 69561 Saint-Genis Laval Cedex, France; Ecole Normale Supérieure de Lyon, Lyon, France; Department of Physics and Astronomy, University of Kansas, Lawrence, KS 66045 USA; ITFA, University of Amsterdam, Science Park 904, 1018 XE Amsterdam, The Netherlands; Laboratoire de L’accelerateur Lineaire (LAL), CNRS/IN2P3, Orsay, France; Niels Bohr Institute, University of Copenhagen, Kobenhavn, Denmark; Department of Physics and Astronomy, Johns Hopkins University, Baltimore, MD 21218 USA; Department of Physics, University of Oregon, Eugene, OR 97403 USA; Theoretical Particle Physics and Cosmology Group, Department of Physics, King’s College London, Strand, London WC2R 2LS UK; Department of Physics, Kyushu University, 6-10-1 Hakozaki, Higashi-ku, Fukuoka, 812-8581 Japan; Department of Physics, Graduate School of Science, and International Center for Elementary Particle Physics, The University of Tokyo, Tokyo, 113-0033 Japan; Theoretical Physics Department, Fermi National Accelerator Laboratory, Batavia, IL USA; SLAC, Stanford University, Menlo Park, CA, 94025 USA; Department of Physics, Tohoku University, Sendai, Miyagi Japan; CNRS, Aix Marseille U., U. de Toulon, CPT, 13288 Marseille, France; Institute for Advanced Study, Technische Universität München, 85748 Garching, Germany; TRIUMF, Vancouver, BC V6T 2A3 Canada

## Abstract

A comprehensive review of physics at an $e^+e^-$ linear collider in the energy range of $\sqrt{s}=92$ GeV–3 TeV is presented in view of recent and expected LHC results, experiments from low-energy as well as astroparticle physics. The report focusses in particular on Higgs-boson, top-quark and electroweak precision physics, but also discusses several models of beyond the standard model physics such as supersymmetry, little Higgs models and extra gauge bosons. The connection to cosmology has been analysed as well.

## Executive summary

### Introduction

With the discovery of a Higgs boson with a mass of about $m_H= 125$ GeV based on data runs at the large hadron collider in its first stage at $\sqrt{s}=7$ and 8 TeV, the striking concept of explaining ‘mass’ as consequence of a spontaneously broken symmetry received a decisive push forward. The significance of this discovery was acknowledged by the award of the Nobel prize for physics to Higgs and Englert in 2013 [1–4]. The underlying idea of the Brout–Englert–Higgs (BEH) mechanism is the existence of a self-interacting Higgs field with a specific potential. The peculiar property of this Higgs field is that it is non-zero in the vacuum. In other words the Higgs field provides the vacuum with a structure. The relevance of such a field not only for our understanding of matter but also for the history of the universe is obvious.

The discovery of a Higgs boson as the materialisation of the Higgs field was the first important step in accomplishing our present level of understanding of the fundamental interactions of nature and the structure of matter that is adequately described by the standard model (SM). In the SM the constituents of matter are fermions, leptons and quarks, classified in three families with identical quantum properties. The electroweak and strong interactions are transmitted via the gauge bosons described by gauge field theories with the fundamental symmetry group $SU(3)_C\times SU(2)_L\times U(1)_Y$.

However, the next immediate steps are to answer the following questions:

- Is there just one Higgs?
- Does the Higgs field associated to the discovered particle really cause the corresponding couplings with all particles? Does it provide the right structure of the vacuum?
- Is it a SM Higgs (width, couplings, spin)? Is it a pure ${\textit{CP}}$-even Higgs boson as predicted in the SM, or is it a Higgs boson from an extended Higgs sector, possibly with some admixture of a ${\textit{CP}}$-odd component? To which model beyond the standard model (BSM) does it point?

In order to definitively establish the mechanism of electroweak symmetry breaking (EWSB), all Higgs-boson properties (mass, width, couplings, quantum numbers) have to be precisely measured and compared with the mass of the corresponding particles.

The LHC has excellent prospects for the future runs^1^ 2 and 3 where proton–proton beams collide with an energy of $\sqrt{s}=13$ TeV starting in spring 2015, continued by runs with a foreseen high luminosity upgrade in the following decade [6]. High-energy $e^+e^-$-colliders have already been essential instruments in the past to search for the fundamental constituents of matter and establish their interactions. The most advanced design for a future lepton collider is the International Linear Collider (ILC) that is laid out for the energy range of $\sqrt{s}=90$ GeV–1 TeV [7, 8]. In case a drive beam accelerator technology can be applied, an energy frontier of about 3 TeV might be accessible with the Compact Linear Collider (CLIC) [9, 10].

At an $e^+e^-$ linear collider (LC) one expects rather clean experimental conditions compared to the conditions at the LHC where one has many overlapping events due to the QCD background from concurring events. A direct consequence is that one does not need any trigger at an LC but can use all data for physics analyses. Due to the collision of point-like particles the physics processes take place at the precisely and well-defined initial energy $\sqrt{s}$, both stable and measurable up to the per-mille level. The energy at the LC is tunable which offers to perform precise energy scans and to optimise the kinematic conditions for the different physics processes, respectively. In addition, the beams can be polarised: the electron beam up to about 90 %, the positron beam up to about 60 %. With such a high degree of polarisation, the initial state is precisely fixed and well known. Due to all these circumstances the final states are generally fully reconstructable so that numerous observables as masses, total cross sections but also differential energy and angular distributions are available for data analyses.

The quintessence of LC physics at the precision frontier is high luminosity and beam polarisation, tunable energy, precisely defined initial state and clear separation of events via excellent detectors. The experimental conditions that are necessary to fulfil the physics requirements have been defined in the LC scope documents [11].

Such clean experimental conditions for high-precision measurements at a LC are the ‘sine qua non’ for resolving the current puzzles and open questions. They allow one to analyse the physics data in a particularly model-independent approach. The compelling physics case for a LC has been described in numerous publications as, for instance [7, 8, 12–16], a short and compact overview is given in [17].

Although the SM has been tremendously successful and its predictions experimentally been tested with accuracies at the quantum level, i.e. significantly below the 1-per-cent level, the SM cannot be regarded as the final theory describing all aspects of nature. Astro-physical measurements [18, 19] are consistent with a universe that contains only 4 % of the total energy composed of ordinary mass but hypothesise the existence of dark matter (DM) accounting for 22 % of the total energy that is responsible for gravitational effects although no visible mass can be seen. Models accounting for DM can easily be embedded within BSM theories as, for instance, supergravity [20]. The strong belief in BSM physics is further supported by the absence of gauge coupling unification in the SM as well as its failure to explain the observed existing imbalance between baryonic and antibaryonic matter in our universe. Such facets together with the experimental data strongly support the interpretation that the SM picture is not complete but constitutes only a low-energy limit of an all-encompassing ‘theory of everything’, embedding gravity and quantum theory to describe all physical aspects of the universe. Therefore experimental hints for BSM physics are expected to manifest themselves at future colliders and model-independent strategies are crucial to determine the underlying structure of the model.

A priori there are only two approaches to reveal signals of new physics and to manifest the model of BSM at future experiments. Since the properties of the matter and gauge particles in the SM may be affected by the new energy scales, a ‘bottom-up’ approach consists in performing high precision studies of the top, Higgs and electroweak gauge bosons. Deviations from those measurements to SM predictions reveal hints to BSM physics. Under the assumption that future experiments can be performed at energies high enough to cross new thresholds, a ‘top-down’ approach becomes also feasible where the new particles or interactions can be produced and studied directly.

Obviously, the complementary search strategies at lepton and hadron colliders are predestinated for such successful dual approaches. A successful high-energy LC was already realised in the 1990s with the construction and running of the SLAC Linear Collider (SLC) that delivered up to $5\times 10^{10}$ particles per pulse. Applying in addition highly polarised electrons enabled the SLC to provide the best single measurement of the electroweak mixing angle with $\delta \sin ^2\theta _W \sim 0.00027$.

However, such a high precision manifests a still-existing inconsistency, namely the well-known discrepancy between the left–right polarisation asymmetry at the *Z*-pole measured at SLC and the forward–backward asymmetry measured at LEP [21]. Both values lead to measured values of the electroweak mixing angle $\sin ^2\theta _\mathrm{eff}$ that differ by more than 3$\sigma$ and point to different predictions for the Higgs mass, see Sect. 4 for more details. Clarifying the central value as well as improving the precision is essential for testing the consistence of the SM as well as BSM models.

Another example for the relevance of highest precision measurements and their interplay with most accurate theoretical predictions at the quantum level is impressively demonstrated in the interpretation of the muon anomalous moment $g_{\mu }-2$ [22]. The foreseen run of the $g_{\mu }-2$ experiment at Fermilab, starting in 2017 [23, 24], will further improve the current experimental precision by about a factor of 4 and will set substantial bounds to many new physics models via their high sensitivity to virtual effects of new particles.

The LC concept has been proposed already in 1965 [25] for providing electron beams with high enough quality for collision experiments. In [26] this concept has been proposed for collision experiments at high energies in order to avoid the energy loss via synchrotron radiation: this energy loss per turn scales with $E^4/r$, where *E* denotes the beam energy and *r* the bending radius. The challenging problems at the LC compared to circular colliders, however, are the luminosity and the energy transfer to the beams. The luminosity is given by

1 $$\begin{aligned} {\mathscr {L}}\sim \frac{\eta P N_e}{\sigma _{xy} E_{\mathrm{c.m.}}}, \end{aligned}$$

where *P* denotes the required power with efficiency $\eta$, $N_e$ the charge per bunch, $E_{\mathrm{c.m.}}$ the centre-of-mass energy and $\sigma _{xy}$ the transverse geometry of the beam size. From Eq. (1), it is obvious that flat beams and a high bunch charge allow high luminosity with lower required beam power $P_b=\epsilon P$. The current designs for a high-luminosity $e^+e^-$ collider, ILC or CLIC, is perfectly aligned with such arguments. One expects an efficiency factor of about $\eta \sim 20\,\%$ for the discussed designs.

The detectors are designed to improve the momentum resolution from tracking by a factor 10 and the jet-energy resolution by a factor 3 (in comparison with the CMS detector) and excellent $\tau ^{\pm }$-, *b*-, $\bar{b}$- and *c*, $\bar{c}$-tagging capabilities [8], are expected.

As mentioned before, another novelty is the availability of the polarisation of both beams, which can precisely project out the interaction vertices and can analyse its chirality directly.

The experimental conditions to achieve such an unprecedented precision frontier at high energy are high luminosity (even about three orders of magnitude more particles per pulse, $5\times 10^{13}$ than at the SLC), polarised electron/positron beams, tunable energy, luminosity and beam-energy stability below $0.1\,\%$ level [11]. Assuming a finite total overall running time it is a critical issue to divide up the available time between the different energies, polarisations and running options in order to maximise the physical results. Several running scenarios are thoroughly studied [27].

In the remainder of this chapter we summarise the physics highlights of this report. The corresponding details can be found in the following chapters. Starting with the three safe pillars of LC physics – Higgs-, top- and electroweak high precision physics – Sect. 2 provides a comprehensive overview about the physics of EWSB. Recent developments in LHC analyses as well as on the theory side are included, alternatives to the Higgs models are discussed. Section 3 covers QCD and in particular top-quark physics. The LC will also set a new frontier in experimental precision physics and has a striking potential for discoveries in indirect searches. In Sect. 4 the impact of electroweak precision observables (EWPO) and their interpretation within BSM physics are discussed. Supersymmetry (SUSY) is a well-defined example for physics beyond the SM with high predictive power. Therefore in Sect. 5 the potential of a LC for unravelling and determining the underlying structure in different SUSY models is discussed. Since many aspects of new physics have strong impact on astroparticle physics and cosmology, Sect. 6 provides an overview in this regard.

The above-mentioned safe physics topics can be realised at best at different energy stages at the linear collider. The possible staged energy approach for a LC is therefore ideally suited to address all the different physics topics. For some specific physics questions very high luminosity is required and in this context also a high-luminosity option at the LC is discussed, see [27] for technical details. The expected physics results of the high-luminosity LC was studied in different working group reports [28, 29], cf. Sect. 2.3.

Such an optimisation of the different running options of a LC depends on the still awaited physics demands. The possible physics outcome of different running scenarios at the LC are currently under study [27], but fixing the final running strategy is not yet advisable.

One should note, however, that such a large machine flexibility is one of the striking features of a LC.

### Physics highlights

Many of the examples shown in this review are based on results of [8–10, 30, 31] and references therein.

#### Higgs physics

The need for precision studies of the new boson, compatible with a SM-like Higgs, illuminates already the clear path for taking data at different energy stages at the LC.

For a Higgs boson with a mass of 125 GeV, the first envisaged energy stage is at about $\sqrt{s}=250$ GeV: the dominant Higgs-strahlung process peaks at $\sqrt{s}=240$ GeV. This energy stage allows the model-independent measurement of the cross section $\sigma (HZ)$ with an accuracy of about 2.6 %, cf. Sect. 2.3. This quantity is the crucial ingredient for all further Higgs analyses, in particular for deriving the total width via measuring the ratio of the partial width and the corresponding branching ratio. Already at this stage many couplings can be determined with high accuracy in a model-independent way: a striking example is the precision of 1.3 % that can be expected for the coupling $g_{HZZ}$, see Sect. 2.3 for more details.

The precise determination of the mass is of interest in its own right. However, it has also high impact for probing the Higgs physics, since $m_H$ is a crucial input parameter. For instance, the branching ratios $H\rightarrow ZZ^*$, $WW^*$ are very sensitive to $m_H$: a change in $m_H$ by 200 MeV shifts $\mathrm{BR}(H\rightarrow ZZ^*$ by 2.5 %. Performing accurate threshold scans enables the most precise mass measurements of $\delta m_H=40$ MeV. Furthermore and – of more fundamental relevance – such threshold scans in combination with measuring different angular distributions allow a model-independent and unique determination of the spin.

Another crucial quantity in the Higgs sector is the total width ${\varGamma }_H$ of the Higgs boson. The prediction in the SM is ${\varGamma }_H=4.07$ MeV for $m_H=125$ GeV [32]. The direct measurement of such a small width is neither possible at the LHC nor at the LC since it is much smaller than any detector resolution. Nevertheless, at the LC a model-independent determination of ${\varGamma }_H$ can be achieved using the absolute measurement of Higgs branching ratios together with measurements of the corresponding partial widths. An essential input quantity in this context is again the precisely measured total cross section of the Higgs-strahlung process. At $\sqrt{s}=500$ GeV, one can derive the total width ${\varGamma }_H$ with a precision of 5 % based on a combination of the $H\rightarrow ZZ^*$ and $WW^*$ channels. Besides this model-independent determination, which is unique to the LC, constraints on the total width can also be obtained at the LC from a combination of on- and off-shell Higgs contributions [33] in a similar way as at the LHC [34]. The latter method, however, relies on certain theoretical assumptions, and also in terms of the achievable accuracy it is not competitive with the model-independent measurement based on the production cross section $\sigma (ZH)$ [33].

At higher energy such off-shell decays of the Higgs boson to pairs of *W* and *Z* bosons offer access to the kinematic dependence of higher-dimensional operators involving the Higgs boson. This dependence allows for example the test of unitarity in BSM models [35, 36].

In order to really establish the mechanism of EWSB it is not only important to measure all couplings but also to measure the Higgs potential:

$$\begin{aligned} V=\frac{1}{2} m_H^2 \Phi ^2_H + \lambda v \Phi _H^3+\frac{1}{4} \kappa \Phi _H^4, \end{aligned}$$

where $v=246$ GeV. It is essential to measure the tri-linear coupling rather accurate in order to test whether the observed Higgs boson originates from a field that is in concordance with the observed particle masses and the predicted EWSB mechanism.^2^ Since the cross section for double Higgs-strahlung is small but has a maximum of about 0.2 fb at $\sqrt{s}=500$ GeV for $m_H=125$ GeV, this energy stage is required to enable a first measurement of this coupling. The uncertainty scales with ${\varDelta } \lambda {/}\lambda =1.8 {\varDelta } \sigma {/}\sigma$. New involved analyses methods in full simulations aim at a precision of 20 % at $\sqrt{s}= 500$ GeV. Better accuracy one could get applying the full LC programme and going also to higher energy, $\sqrt{s}=1$ TeV.

Another very crucial quantity is accessible at $\sqrt{s}=500$ GeV: the $t\bar{t}H$-coupling. Measuring the top-Yukawa coupling is a challenging endeavour since it is overwhelmed from $t\bar{t}$-background. At the LHC one expects an accuracy of 25 % on basis of 300 fb$^{-1}$ and under optimal assumptions and neglecting the error from theory uncertainties. At the LC already at the energy stage of $\sqrt{s}=500$ GeV, it is expected to achieve an accuracy of ${\varDelta } g_{ttH}/g_{ttH}\sim 10$ %, see Sect. 2. This energy stage is close to the threshold of *ttH* production, therefore the cross section for this process should be small. But thanks to QCD-induced threshold effects the cross section gets enhanced and such an accuracy should be achievable with 1 ab$^{-1}$ at the LC. It is of great importance to measure this Yukawa coupling with high precision in order to test the Higgs mechanism and verify the measured top mass $m_t=y_{ttH} v/\sqrt{2}$. The precise determination of the top Yukawa coupling opens a sensitive window to new physics and admixtures of non-SM contributions. For instance, in the general two-Higgs-doublet model the deviations with respect to the SM value of this coupling can typically be as large as $\sim 20\,\%$.

**Fig. 1:**
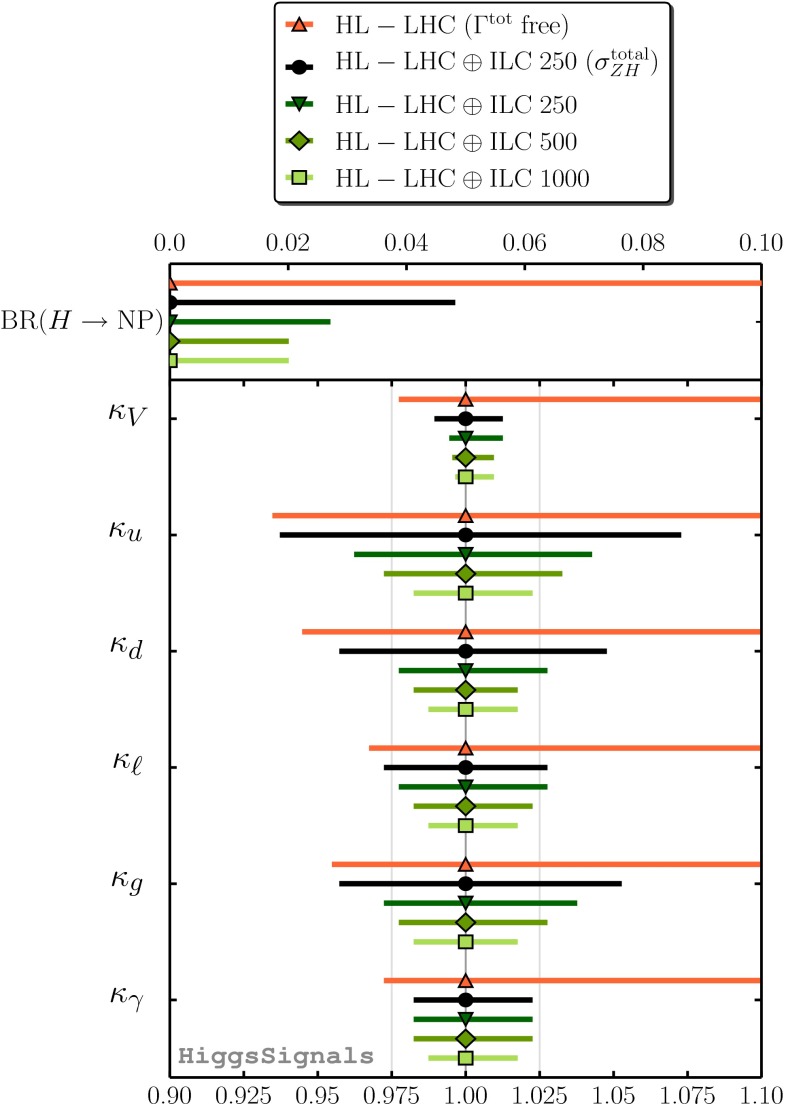
The achievable precision in the different Higgs couplings at the LHC on bases of $3 ab^{-1}$ and 50 % improvement in the theoretical uncertainties in comparison with the different energy stages at the ILC. In the final LC stage all couplings can be obtained in the 1–2 % range, some even better [39]

Since for a fixed $m_H$ all Higgs couplings are specified in the SM, it is not possible to perform a fit within this model. In order to test the compatibility of the SM Higgs predictions with the experimental data, the LHC Higgs Cross Section Group proposed ‘coupling scale factors’ [37, 38]. These scale factors $\kappa _i$ ($\kappa _i=1$ corresponds to the SM) dress the predicted Higgs cross section and partial widths. Applying such a $\kappa$-framework, the following assumptions have been made: there is only one 125 GeV state responsible for the signal with a coupling structure identical to the SM Higgs, i.e. a pure ${\textit{CP}}$-even state, and the zero width approximation can be applied. Usually, in addition the theory assumption $\kappa _{W,Z}<1$ (corresponds to an assumption on the total width) has to be made. Using, however, LC data and exploiting the precise measurement of $\sigma (HZ)$, this theory assumption can be dropped and all couplings can be obtained with an unprecedented precision of at least 1–2 %, see Fig. 1 [39] and Sect. 2 for further details.

Another important property of the Higgs boson that has to be determined is the ${\textit{CP}}$ quantum number. In the SM the Higgs should be a pure ${\textit{CP}}$-even state. In BSM models, however, the observed boson state a priori can be any admixture of ${\textit{CP}}$-even and ${\textit{CP}}$-odd states, it is of high interest to determine limits on this admixture. The *HVV* couplings project out only the ${\textit{CP}}$-even components, therefore the degree of ${\textit{CP}}$ admixture cannot be tackled via analysing these couplings. The measurements of ${\textit{CP}}$-odd observables are mandatory to reveal the Higgs ${\textit{CP}}$-properties: for instance, the decays of the Higgs boson into $\tau$ leptons provides the possibility to construct unique ${\textit{CP}}$-odd observables via the polarisation vector of the $\tau$s, see further details in Sect. 2.

#### Top-quark physics

**Fig. 2:**
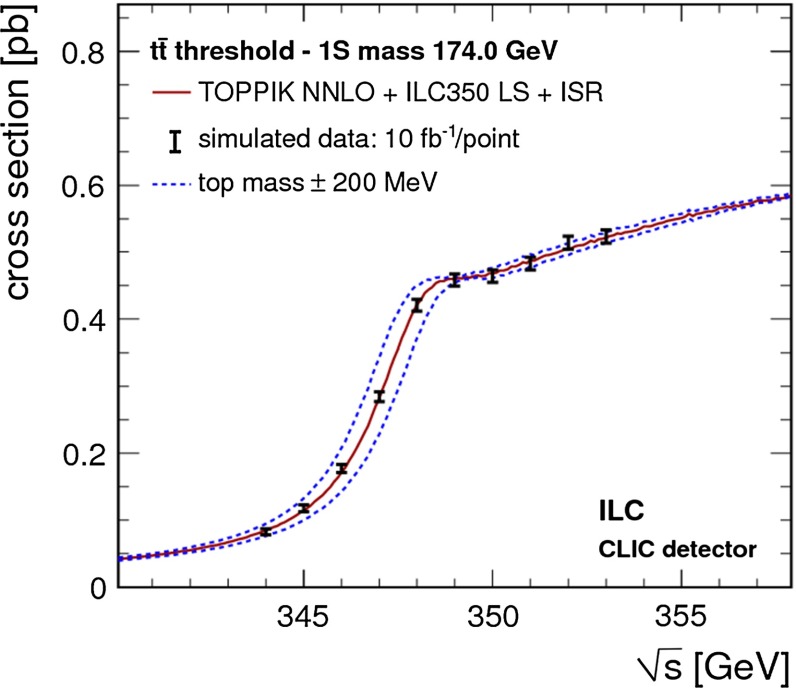
Simulated measurement of the background-subtracted $t\bar{t}$ cross section with 10 fb$^{-1}$ per data point, assuming a top-quark mass of 174 GeV in the 1S scheme with the ILC luminosity spectrum for the CLIC-ILD detector [40]

Top-quark physics is another rich field of phenomenology. It opens at $\sqrt{s}=350$ GeV. The mass of the top quark itself has high impact on the physics analysis. In BSM physics $m_t$ is often the crucial parameter in loop corrections to the Higgs mass. In each model where the Higgs-boson mass is not a free parameter but predicted in terms of the other model parameters, the top-quark mass enters the respective loop diagrams to the fourth power, see Sect. 4 for details. Therefore the interpretation of consistency tests of the EWPO $m_W$, $m_Z$, $\sin ^2\theta _\mathrm{eff}$ and $m_H$ require the most precise knowledge on the top-quark mass. The top quark is not an asymptotic state and $m_t$ depends on the renormalisation scheme. Therefore a clear definition of the used top quark mass is needed. Measuring the mass via a threshold scan allows to relate the measured mass uniquely to the well-defined $m_t^{\overline{\mathrm{MS}}}$ mass, see Fig. 2. Therefore, this procedure is advantageous compared to measurements via continuum observables. It is expected to achieve an unprecedented accuracy of ${\varDelta } m_{t}^{\overline{\mathrm{MS}}}=100$ MeV via threshold scans. This uncertainty contains already theoretical as well as experimental uncertainties. Only such a high accuracy enables sensitivity to loop corrections for EWPO. Furthermore the accurate determination is also decisive for tests of the vacuum stability within the SM.

**Fig. 3:**
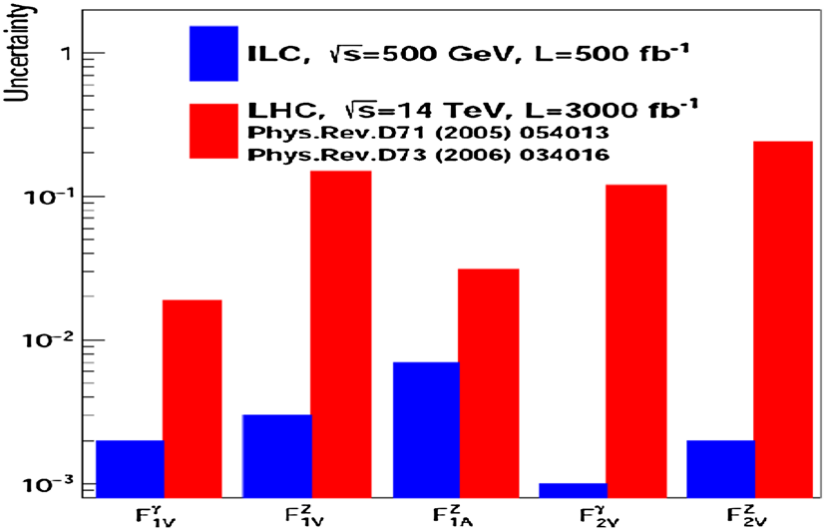
Statistical precision on ${\textit{CP}}$-conserving form factors expected at the LHC [42] and at the ILC [41]. The LHC results assume an integrated luminosity of ${\mathscr {L}}=300$ fb$^{-1}$. The results for the ILC are based on an integrated luminosity of ${\mathscr {L}}=500$ fb$^{-1}$ at $\sqrt{s}=500$ GeV and a beam polarisation of $P_{e^-}=\pm 80\,\%$, $P_{e^+}=\mp 30\,\%$ [41]

A sensitive window to BSM physics is opened by the analysis of the top quark couplings. Therefore a precise determination of all SM top-quark couplings together with the search for anomalous couplings is crucial and can be performed very accurately at $\sqrt{s}=500$ GeV. Using the form-factor decomposition of the electroweak top quark couplings, it has been shown that one can improve the accuracy for the determination of the couplings [41] by about one order of magitude at the LC compared to studies at the LHC, see Fig. 3 and Sect. 3.

#### Beyond standard model physics – “top-down”

*Supersymmetry* The SUSY concept is one of the most popular extensions of the SM since it can close several open questions of the SM: achieving gauge unification, providing DM candidates, stabilising the Higgs mass, embedding new sources for ${\textit{CP}}$-violation and also potentially neutrino mixing. However, the symmetry has to be broken and the mechanism for symmetry breaking is completely unknown. Therefore the most general parametrisation allows around 100 new parameters. In order to enable phenomenological interpretations, for instance, at the LHC, strong restrictive assumptions on the SUSY mass spectrum are set. However, as long as it is not possible to describe the SUSY breaking mechanism within a full theory, data interpretations based on these assumptions should be regarded as a pragmatic approach. Therefore the rather high limits obtained at the LHC for some coloured particles exclude neither the concept of SUSY as such, nor do they exclude light electroweak particles, nor relatively light scalar quarks of the third generation.

Already the energy stage at $\sqrt{s}=350$ GeV provides a representative open window for the direct production of light SUSY particles, for instance, light higgsino-like scenarios, leading to signatures with only soft photons. The resolution of such signatures will be extremely challenging at the LHC but is feasible at the LC via the ISR method, as discussed in Sect. 5.

**Fig. 4:**
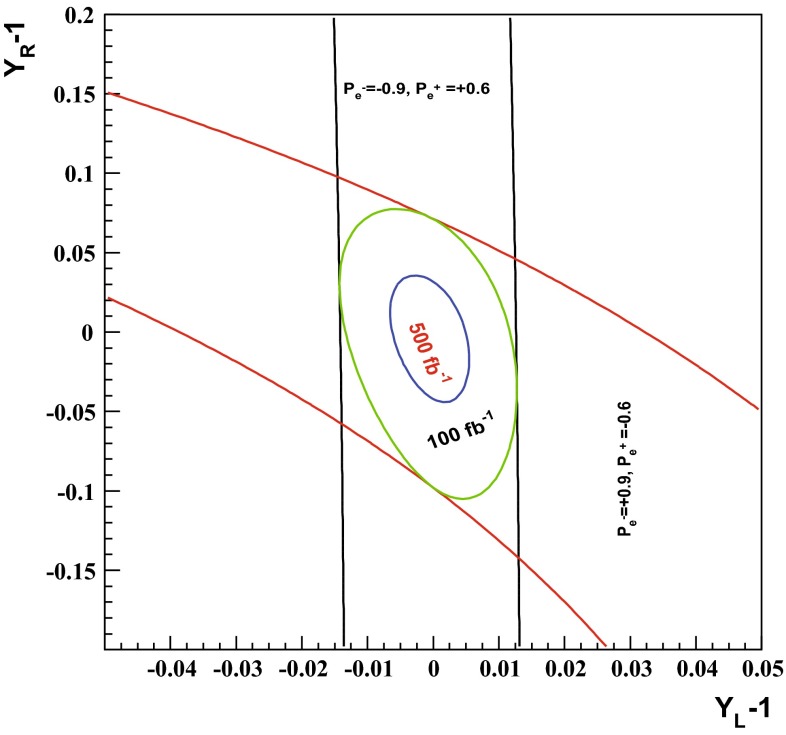
Equivalence of the SUSY electroweak Yukawa couplings $g_{\tilde{W}}$, $g_{\tilde{B}}$ with the *SU*(2), *U*(1) gauge couplings *g*, $g^{\prime }$. Shown are the contours of the polarised cross sections $\sigma _L(e^+e^-\rightarrow \tilde{\chi }^0_1\tilde{\chi }^0_2)$ and $\sigma _R(e^+e^-\rightarrow \tilde{\chi }^0_1\tilde{\chi }^0_2)$ in the plane of the SUSY electroweak Yukawa couplings normalised to the gauge couplings, $Y_L=g_{\tilde{W}}/g$, $Y_R=g_{\tilde{B}}/g^{\prime }$ [43, 44] for a scenario with the electroweak spectrum similar to the reference point SPS1a

Another striking feature of the LC physics potential is the capability to test predicted properties of new physics candidates. For instance, in SUSY models one essential paradigm is that the coupling structure of the SUSY particle is identical to its SM partner particle. That means, for instance, that the *SU*(3), *SU*(2) and *U*(1) gauge couplings $g_S$, *g* and $g^{\prime }$ have to be identical to the corresponding SUSY Yukawa couplings $g_{\tilde{g}}$, $g_{\tilde{W}}$ and $g_{\tilde{B}}$. These tests are of fundamental importance to establish the theory. Testing, in particular, the SUSY electroweak Yukawa coupling is a unique feature of LC physics. Under the assumption that the *SU*(2) and *U*(1) parameters have been determined in the gaugino/higgsino sector (see Sect. 5.7), the identity of the Yukawa and the gauge couplings via measuring polarised cross sections can be successfully performed: depending on the electron (and positron) beam polarisation and on the luminosity, a per-cent-level precision can be achieved; see Fig. 4.

**Fig. 5:**
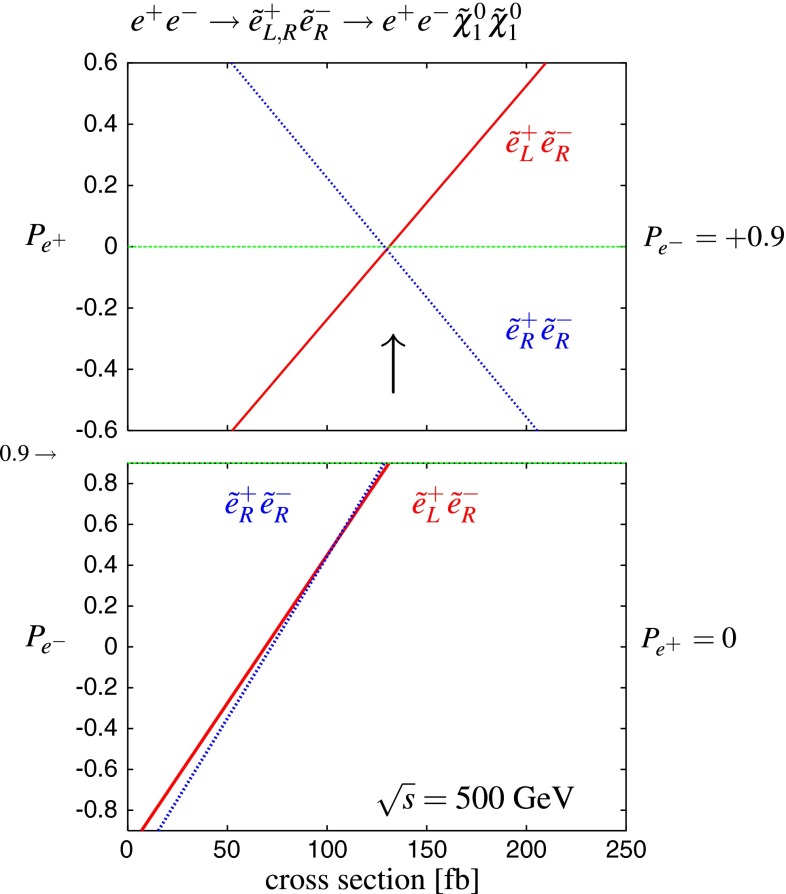
Polarised cross sections versus $P_{e^-}$ (*bottom panel*) and $P_{e^+}$ (*top panel*) for $e^+e^-\rightarrow \tilde{e}\tilde{e}$-production with direct decays in $\tilde{\chi }^0_1 e$ in a scenario where the non-coloured spectrum is similar to a SPS1a-modified scenario but with $m_{\tilde{e}_L}=200$ GeV, $m_{\tilde{e}_R}=195$ GeV. The associated chiral quantum numbers of the scalar SUSY partners $\tilde{e}_{L,R}$ can be tested via polarised $e^{\pm }$-beams

Another important and unique feature of the LC potential is to test experimentally the quantum numbers of new physics candidates. For instance, a particularly challenging measurement is the determination of the chiral quantum numbers of the SUSY partners of the fermions. These partners are predicted to be scalar particles and to carry the chiral quantum numbers of their standard model partners. In $e^+e^-$ collisions, the associated production reactions $e^+e^-\rightarrow \tilde{e}_L^+\tilde{e}_R^-$, $\tilde{e}_R^+\tilde{e}_L^-$ occur only via *t*-channel exchange, where the $e^\pm$ are directly coupled to their SUSY partners $\tilde{e}^{\pm }$. Separating the associated pairs, the chiral quantum numbers can be tested via the polarisation of $e^\pm$ since chirality corresponds to helicity in the high-energy limit. As can be seen in Fig. 5, the polarisation of both beams is absolutely essential to separate the pair $\tilde{e}_L\tilde{e}_R$ [45] and to test the associated quantum numbers.

*Dark matter physics* Weakly interacting massive particles (WIMPs) are the favourite candidates as components of the cold DM. Neutral particles that interact only weakly provide roughly the correct relic density in a natural way. Since there are no candidates for DM in the SM, the strong observational evidence for DM clearly points to physics beyond the SM. Due to precise results from cosmological observations, for instance [46, 47], bounds on the respective cross section and the mass of the DM candidates can be set in the different models. Therefore, in many models only rather light candidates are predicted, i.e. with a mass around the scale of EWSB or even lighter. That means, for instance for SUSY models with *R*-parity conservation, that the lightest SUSY particle, should be within the kinematical reach of the ILC. The lowest threshold for such processes is pair production of the WIMP particle. Since such a final state, however, escapes detection, the process is only visible if accompanied by radiative photons at the LC that recoil against the WIMPs, for instance, the process $e^+e^-\rightarrow \gamma \chi \chi$ [48], where $\chi$ denotes the WIMP particle in general with a spin $S_{\chi }=0,\frac{1}{2},1$. Such a process can be realised in SUSY models, in universal extra dimensions, little Higgs theories etc. The dominant SM background is radiative neutrino production, which can, efficiently be suppressed via the use of beam polarisation.

**Fig. 6:**
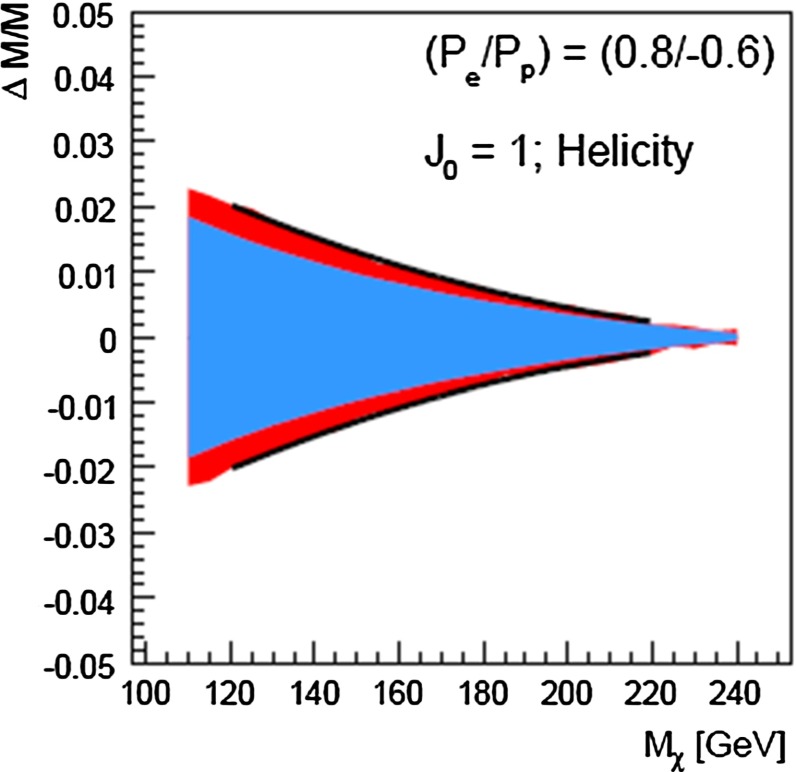
WIMP mass as a function of the mass for p-wave ($J_0=1$) annihilation and under the assumption that WIMP couplings are helicity- and parity-conserving in the process $e^+e^-\rightarrow \gamma \chi \chi$ [48]. With an integrated luminosity of ${\mathscr {L}}=500$ fb$^{-1}$ and polarised beams with $P_{e^-}=+80\,\%$, $P_{e^+}=-60\,\%$ with ${\varDelta } P/P=0.25\,\%$ the reconstructed WIMP mass can be determined with a relative accuracy of the order of 1 % [49]. The *blue* *area* shows the systematic uncertainty and the *red* *bands* the additional statistical contribution. The dominant sources of systematic uncertainties are ${\varDelta } P/P$ and the shape of the beam-energy spectrum

The present DM density depends strongly on the cross section for WIMP annihilation into SM particles (assuming that there exist only one single WIMP particle $\chi$ and ignoring coannihilation processes between the WIMP and other exotic particles) in the limit when the colliding $\chi$s are non-relativistic [48], depending on s- or p-wave contributions and on the WIMP mass. Due to the excellent resolution at the LC the WIMP mass can be determined with relative accuracy of the order of 1 %, see Fig. 6.

**Fig. 7:**
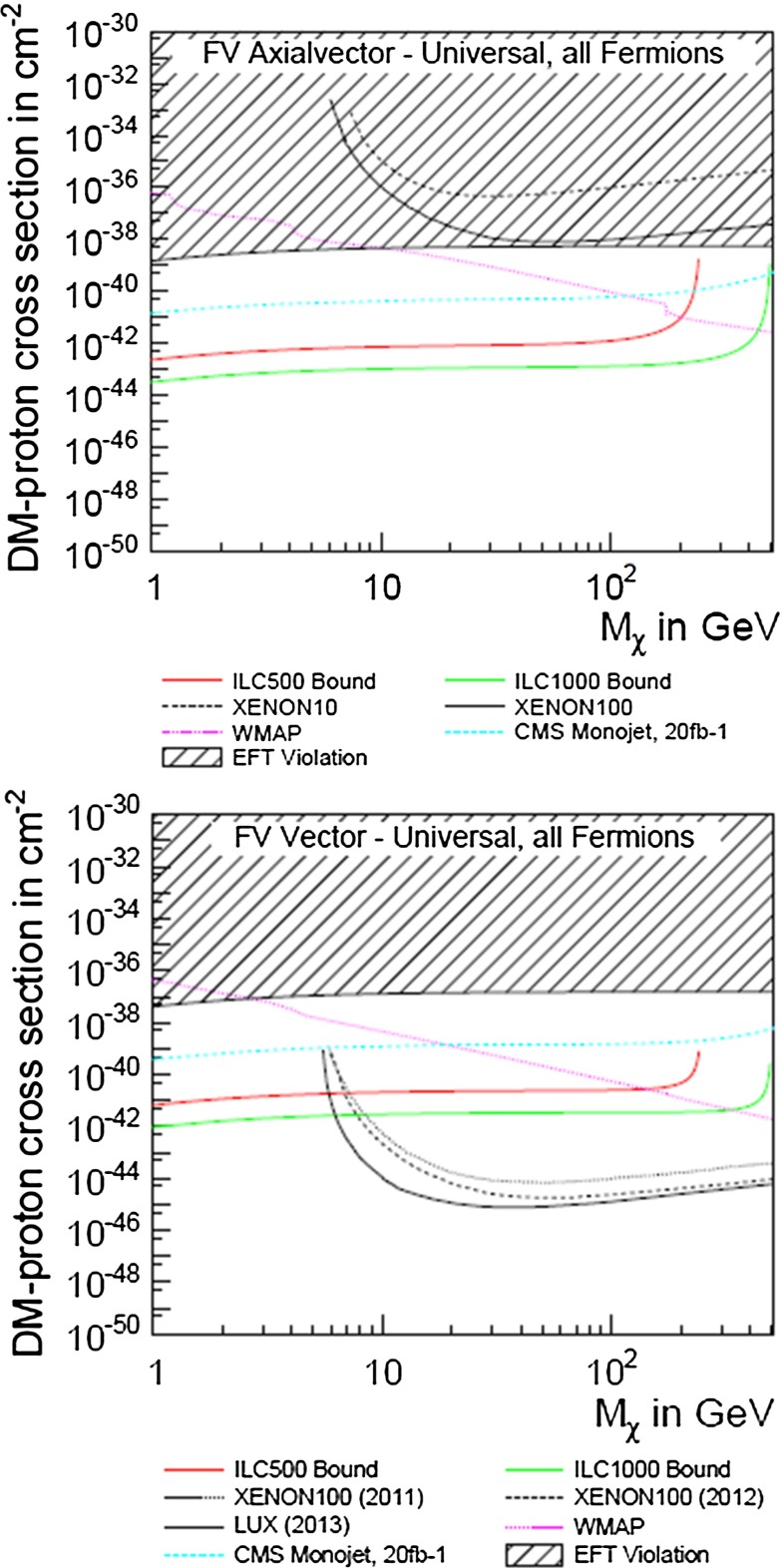
Combined limits for fermionic dark matter models. The process $e^+e^-\rightarrow \chi \chi \gamma$ is assumed to be detected only by the hard photon. The analysis has been modelled correspondingly to [49] and is based on ${\mathscr {L}}=500$ fb$^{-1}$ at $\sqrt{s}=500$ GeV and $\sqrt{s}=1$ TeV and different polarisations [50, 51]

Following another approach and parametrising DM interactions in the form of effective operators, a non-relativistic approximation is not required and the derived bounds can be compared with experimental bounds from direct detection. Assuming that the DM particles only interact with SM fields via heavy mediators that are kinematically not accessible at the ILC, it was shown in [50, 51] that the ILC could nevertheless probe effective WIMP couplings $G^\mathrm{ILC}_{\max }=g_ig_j/M^2 = 10^{-7}$ GeV$^{-2}$ (vector or scalar mediator case), or $G^\mathrm{ILC}_{\max }=g_ig_j/M = 10^{-4}$ GeV$^{-1}$ (fermionic mediator case). The direct detection searches give much stronger bounds on spin-independent (‘vector’) than on spin-dependent (‘axial-vector’) interactions under the simplifying assumption that all SM particles couple with the same strength to the DM candidate (‘universal coupling’). If the WIMP particle is rather light ($<$10 GeV) the ILC offers a unique opportunity to search for DM candidates beyond any other experiment, even for spin-independent interactions, cf. Fig. 7 (upper panel). In view of spin-dependent interactions the ILC searches are also superior for heavy WIMP particles, see Fig. 7 (lower panel).

**Fig. 8:**
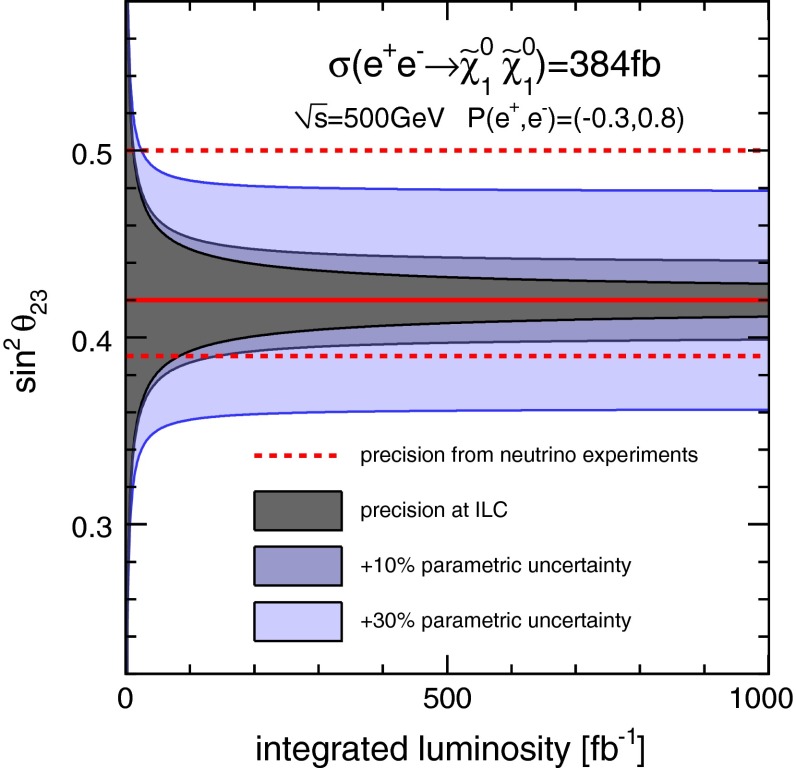
Achievable precision on $\sin ^2\theta _{23}$ from bi-linear *R*-parity-violating decays of the $\tilde{\chi }^0_1$ as a function of the produced number of neutralino pairs compared to the current precision from neutrino oscillation measurements [52]

*Neutrino mixing angle* Another interesting question is how to explain the observed neutrino mixing and mass patterns in a more complete theory. SUSY with broken *R*-parity allows one to embed and to predict such an hierarchical pattern. The mixing between neutralinos and neutrinos puts strong relations between the LSP branching ratios and neutrino mixing angles. For instance, the solar neutrino mixing angle $\sin ^2\theta _{23}$ is accessible via measuring the ratio of the branching fractions for $\tilde{\chi }^0_1 \rightarrow W^\pm \mu ^\mp$ and $W^\pm \tau ^\mp$. Performing an experimental analysis at $\sqrt{s}=500$ GeV allows one to determine the neutrino mixing angle $\sin ^2 \theta _{23}$ up to a per-cent-level precision, as illustrated in Fig. 8 [52].

This direct relation between neutrino physics and high-energy physics is striking. It allows one to directly test whether the measured neutrino mixing angles can be embedded within a theoretical model of high predictive power, namely a bi-linear *R*-parity violation model in SUSY, based on precise measurements of neutralino branching ratios [53, 54] at a future $e^+e^-$ linear collider.

#### Beyond standard model physics – “bottom-up”

**Fig. 9:**
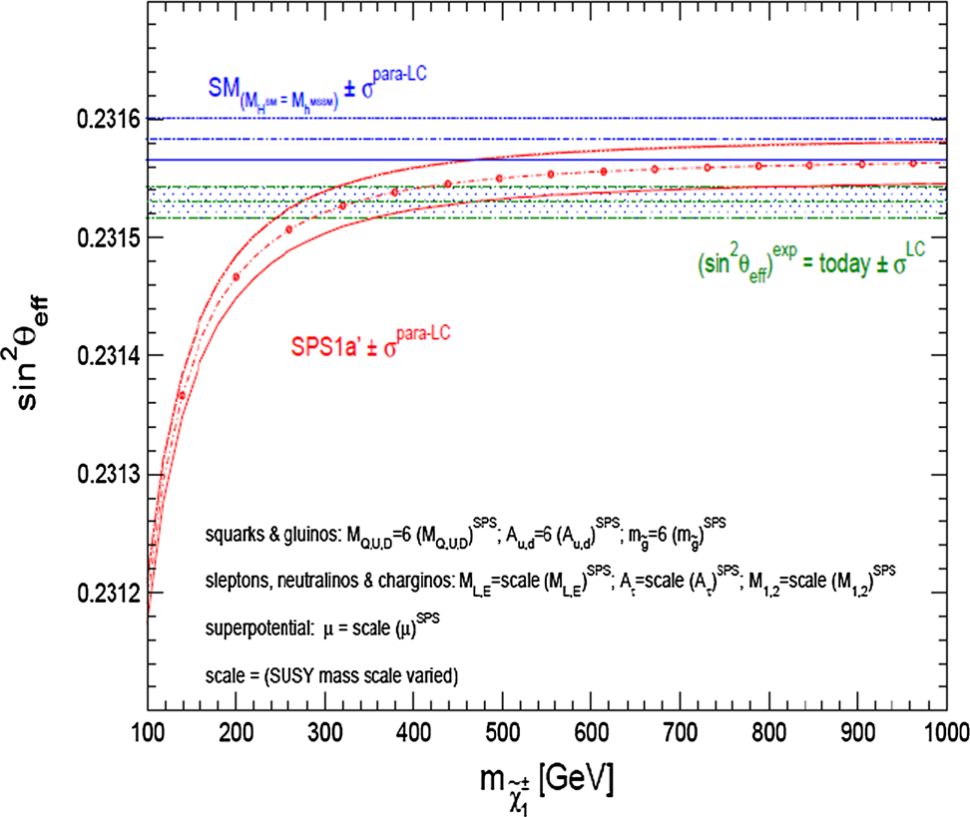
Theoretical prediction for $\sin ^2\theta _\mathrm{eff}$ in the SM and the MSSM (including prospective parametric theoretical uncertainties) compared to the experimental precision at the LC with GigaZ option. A SUSY inspired scenario SPS 1a’ has been used, where the coloured SUSY particles masses are fixed to 6 times their SPS 1a’ values. The other mass parameters are varied with a common scale factor

*Electroweak precision observables* Another compelling physics case for the LC can be made for the measurement of EWPO at $\sqrt{s}\approx 92$ GeV (*Z*-pole) and $\sqrt{s}\approx 160$ GeV (*WW* threshold), where a new level of precision can be reached. Detecting with highest precision any deviations from the SM predictions provides traces of new physics which could lead to groundbreaking discoveries. Therefore, particularly in case no further discovery is made from the LHC data, it will be beneficial to perform such high-precision measurements at these low energies. Many new physics models, including those of extra large dimensions, of extra gauge bosons, of new leptons, of SUSY, etc., can lead to measurable contributions to the electroweak mixing angle even if the scale of the respective new physics particles are in the multi-TeV range, i.e. out of range of the high-luminosity LHC. Therefore the potential of the LC to measure this quantity with an unprecedented precision, i.e. of about one order of magnitude better than at LEP/SLC offers to enter a new precision frontier. With such a high precision – mandatory are high luminosity and both beams to be polarised – one gets sensitivity to even virtual effects from BSM where the particles are beyond the kinematical reach of the $\sqrt{s}=500$ GeV LC and the LHC. In Fig. 9 the prediction for $\sin ^2\theta _\mathrm{eff}$ as a function of the lighter chargino mass $m_{\tilde{\chi }^{+}_1}$ is shown. The MSSM prediction is compared with the prediction in the SM assuming the experimental resolution expected at GigaZ. In this scenario no coloured SUSY particles would be observed at the LHC but the LC could resolve indirect effects of SUSY up to $m_{\tilde{\chi }^{+}_1}\le 500$ GeV via the measurement of $\sin ^2\theta _\mathrm{eff}$ with unprecedented precision at the low energy option GigaZ, see Sect. 4 for details. The possibility to run with high luminosity and both beam polarised on these low energies is essential in these regards.

**Fig. 10:**
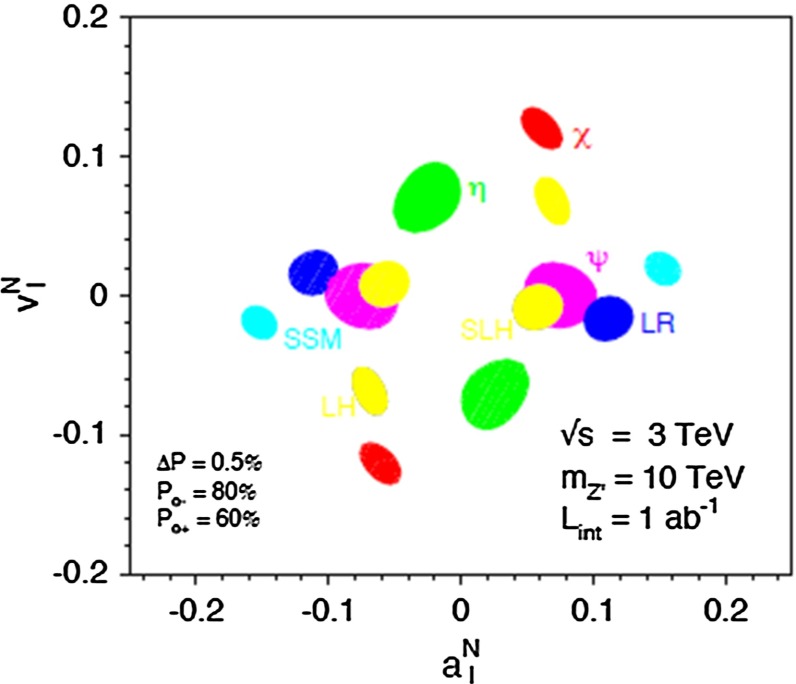
New gauge bosons in the $\mu ^+\mu ^-$ channel. The plot shows the expected resolution at CLIC with $\sqrt{s}=3$ TeV and ${\mathscr {L}}=1$ ab$^{-1}$ on the ‘normalised’ vector $v_f^n=v'_f\sqrt{s/(m_Z'^2-s)}$ and axial-vector $a_f^n=a'_f\sqrt{s/(m_Z'^2-s)}$ couplings to a 10 TeV $Z'$ in terms of the SM couplings $v'_f$, $a'_f$. The mass of $Z'$ is assumed to be unknown, nevertheless the couplings can be determined up to a two-fold ambiguity. The colours denote different $Z'$ models [9, 10]

*Extra gauge bosons* One should stress that not only SUSY theories can be tested via indirect searches, but also other models, for instance, models with large extra dimensions or models with extra $Z'$, see Fig. 10, where the mass of the $Z'$ boson is far beyond the direct kinematical reach of the LHC and the LC and therefore is assumed to be unknown. Because of the clean LC environment, one even can determine the vector and axial-vector coupling of such a $Z'$ model.

#### Synopsis

The full Higgs and top-quark physics programme as well as the promising programme on DM and BSM physics should be accomplished with the higher energy LC set-up at 1 TeV. Model-independent parameter determination is essential for the crucial identification of the underlying model. Accessing a large part of the particle spectrum of a new physics model would nail down the structure of the underlying physics. But measuring already only the light part of the spectrum with high precision and model-independently can provide substantial information. Table 1 gives an overview of the different physics topics and the required energy stages. The possibility of a tunable energy in combination with polarised beams, is particularly beneficial to successfully accomplish the comprehensive physics programme at high-energy physics collider and to fully exploit the complete physics potential of the future Linear Collider.

**Table 1:** Physics topics where the $e^+e^-$-linear collider provides substantial results at the different energy stages that are complementary to the LHC. The examples are described in the following chapters as well as in [7–10, 12–17, 27, 28, 30, 31, 55, 56]

$\sqrt{s}/$GeV	92,160	240	350	500	1000	3000	Threshold scans required
Higgs							
$m_H$	–	$\times$	$\times$	$\times$	$\times$	$\times$	$\times$
${\varGamma }_{\mathrm{tot}}$	–	–	$\times$	$\times$			
$g_{c,b}$	–	$\times$	$\times$	$\times$		$\times$	
$g_{ttH}$	–	–	–	$\times$	$\times$		
$g_{HHH}$	–	–	–	$\times$	$\times$	$\times$	
$m_{H,A}^{\mathrm{SUSY}}$	–	–	–	$\times$	$\times$	$\times$	$\times$
Top							
$m_{t}^{\mathrm{th}}$	–	–	$\times$				$\times$
$m_{t}^{\mathrm{cont}}$	–	–	–	$\times$	($\times$)	($\times$)	
$A_{\mathrm{FB}}^t$	–	–	$\times$	$\times$			
$g_{Z,\gamma }$	–	–	–	$\times$			
$g_{FCNC}$	–	–	–	$\times$	$\times$	(?)	
Electroweak precision observables							
$\sin ^2\theta _\mathrm{eff}$(*Z*-pole)	$\times$					($\times$)	
$m_W^{\mathrm{th}}$	$\times$						$\times$
$m_W^{\mathrm{cont}}$		$\times$	$\times$	$\times$	($\times$)	($\times$)	
${\varGamma }_Z$	$\times$						$\times$
$A_{\mathrm{LR}}$	$\times$						
$A_{\mathrm{FB}}$	$\times$						
SUSY							
Indirect search	$\times$	$\times$	$\times$				
Direct search	–	–	$\times$	$\times$	$\times$	$\times$	$\times$
Light higgsinos	–	–	$\times$	$\times$			$\times$
Parameter determination	–	–	$\times$	$\times$	$\times$		$\times$
Quantum numbers	–	–	$\times$	$\times$	$\times$		$\times$
Extrapolations	–	–	–	$\times$	$\times$	$\times$	$\times$
$\nu$ mixing							
$\theta ^2_{23}$	–	–	$\times$	$\times$			
Dark matter							
Effective-field-theory	–	–	–	$\times$	$\times$	$\times$	
Non-relativistic	–	–	$\times$	$\times$	$\times$	$\times$	
Extra gauge bosons							
Indirect search $m_{z'}$	$\times$	–	–	$\times$	$\times$	$\times$	
$v'_f$, $a'_f$	–	–	–	$\times$	$\times$	($\times$)	
$m_{W'}$	$\times$	–	–	$\times$	$\times$	$\times$	
Direct search	–	–	–	–	–	$\times$	$\times$

## Higgs and electroweak symmetry breaking^3^

After a brief description of the physical basis of the Higgs mechanism, we summarise the crucial results for Higgs properties in the standard model as expected from measurements at LHC and ILC/CLIC, based on the respective reports. Extensions of the SM Higgs sector are sketched thereafter, discussed thoroughly in the detailed reports which follow: portal models requiring analyses of invisible Higgs decays, supersymmetry scenarios as generic representatives of weakly coupled Higgs sectors, and finally strong interaction elements as suggested by Little Higgs models and composite models motivated by extended space dimensions.

### Résumé^5^

The Brout–Englert–Higgs mechanism [1–4, 57] is a central element of particle physics. Masses are introduced consistently in gauge theories for vector bosons, leptons and quarks, and the Higgs boson itself, by transformation of the interaction energy between the initially massless fields and the vacuum expectation value of the Higgs-field. The non-zero value of the Higgs field in the vacuum, at the minimum of the potential breaking the electroweak symmetry, is generated by self-interactions of the Higgs field. The framework of the SM [58–60] demands the physical Higgs boson as a new scalar degree of freedom, supplementing the spectrum of vectorial gauge bosons and spinorial matter particles.

This concept of mass generation has also been applied, *mutatis mutandis*, to extended theories into which the SM may be embedded. The new theory may remain weakly interacting up to the grand-unification scale, or even the Planck scale, as familiar in particular from supersymmetric theories, or novel strong interactions may become effective already close to the TeV regime. In such theories the Higgs sector is enlarged compared with the SM. A spectrum of several Higgs particles is generally predicted, the lightest particle often with properties close to the SM Higgs boson, and others with masses typically in the TeV regime.

A breakthrough on the path to establishing the Higgs mechanism experimentally has been achieved by observing at LHC [61, 62] a new particle with a mass of about 125 GeV and couplings to electroweak gauge bosons and matter particles compatible, *cum grano salis*, with expectations for the Higgs boson in the (SM) [63–66].

#### Zeroing in on the Higgs particle of the SM

Within the SM the Higgs mechanism is realised by introducing a scalar weak-isospin doublet. Three Goldstone degrees of freedom are absorbed for generating the longitudinal components of the massive electroweak $W^\pm ,Z$ bosons, and one degree of freedom is realised as a scalar physical particle unitarising the theory properly. After the candidate particle has been found, three steps are necessary to establish the relation with the Higgs mechanism:

- The mass, the lifetime (width) and the spin/${\textit{CP}}$ quantum numbers must be measured as general characteristics of the particle;
- The couplings of the Higgs particle to electroweak gauge bosons and to leptons/quarks must be proven to rise (linearly) with their masses;
- The self-coupling of the Higgs particle, responsible for the potential which generates the non-zero vacuum value of the Higgs field, must be established.

When the mass of the Higgs particle is fixed, all its properties are pre-determined. The spin/${\textit{CP}}$ assignement $J^{{\textit{CP}}} = 0^{+\!+}$ is required for an isotropic and *C*, *P*-even vacuum. Gauge interactions of the vacuum Higgs-field with the electroweak bosons and Yukawa interactions with the leptons/quarks generate the masses which in turn determine the couplings of the Higgs particle to all SM particles. Finally, the self-interaction potential, which leads to the non-zero vacuum value *v* of the Higgs field, being responsible for breaking the electroweak symmetries, is determined by the Higgs mass, and, as a result, the tri-linear and quadri-linear Higgs self-interactions are fixed.

Since the Higgs mechanism provides the closure of the SM, the experimental investigation of the mechanism, connected with precision measurements^6^ of the properties of the Higgs particle, is mandatory for the understanding of the microscopic laws of nature as formulated at the electroweak scale. However, even though the SM is internally consistent, the large number of parameters, *notabene* mass and mixing parameters induced in the Higgs sector, suggests the embedding of the SM into a more comprehensive theory (potentially passing on the way through even more complex structures). Thus observing specific patterns in the Higgs sector could hold essential clues to this underlying theory.

**Fig. 11:**
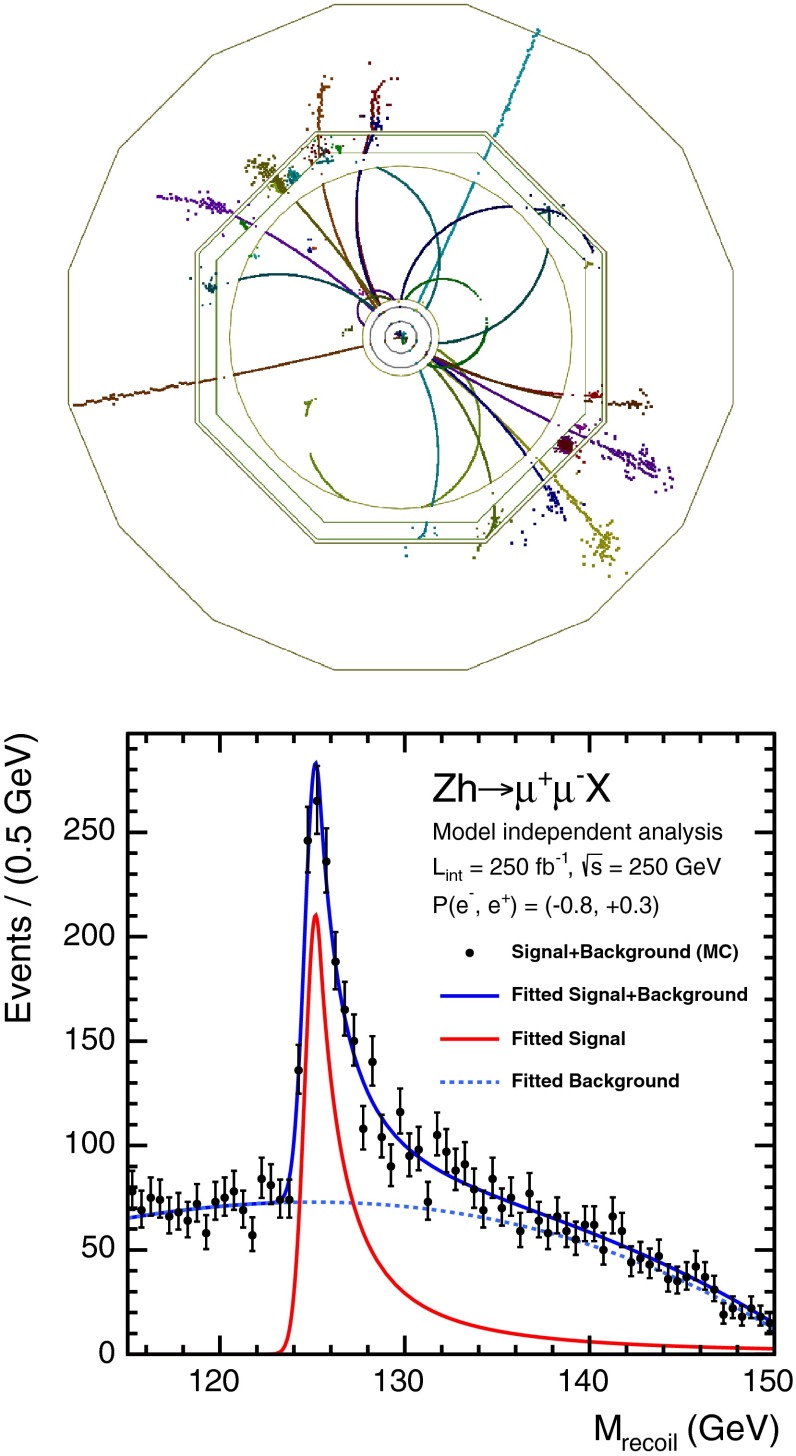
U*pper plot* Event in Higgs-strahlung $e^+ e^- \rightarrow ZH \rightarrow (\mu ^+ \mu ^-)(\mathrm{jet}\; \mathrm{jet})$ for a Higgs mass of 125 GeV at a collider energy of 500 GeV; *lower plot* Distribution of the recoiling Higgs decay jets

The SM Higgs boson can be produced through several channels in *pp* collisions at LHC, with gluon fusion providing by far the maximum rate for intermediate masses. In $e^+ e^-$ collisions the central channels [67–71] are

2 $$\begin{aligned}&{\text {Higgs-strahlung}} :e^+ e^- \rightarrow Z + H \end{aligned}$$

3 $$\begin{aligned}&W {\text {-boson fusion}} :e^+ e^- \rightarrow {\bar{\nu }}_e \nu _e + H \,, \end{aligned}$$

with cross sections for a Higgs mass $M_H = 125$ GeV as shown in Table 2 for the LC target energies of 250, 500 GeV, 1 and 3 TeV. By observing the *Z*-boson in Higgs-strahlung, cf. Fig. 11, the properties of the Higgs boson in the recoil state can be studied experimentally in a model-independent way.

**Table 2:** Cross sections in units of fb for Higgs-strahlung and *W*-boson fusion of Higgs bosons in the SM for a set of typical ILC/CLIC energies with beam polarisations: $P(e^-,e^+)=(-0.8,+0.3)$ for ILC at 250 and 500 GeV, $(-0.8,+0.2)$ for ILC at 1 TeV, and $(-0.8,0)$ for CLIC at 3 TeV

	250 GeV	500 GeV	1 TeV	3 TeV
$\sigma [e^+e^- \rightarrow ZH]$	318	95.5	22.3	2.37
$\sigma [e^+e^- \rightarrow \bar{\nu }_e \nu _e H]$	36.6	163	425	862

*(a) Higgs particle: mass and *$J^{{\textit{CP}}}$

Already for quite some time, precision analyses of the electroweak parameters, like the $\rho$-parameter, suggested an SM Higgs mass of less than 161 GeV in the intermediate range [21], above the lower LEP2 limit of 114.4 GeV [72] (for a review see [73]). The mass of the new particle observed close to 125 GeV at LHC, agrees nicely with this expectation.

The final accuracy for direct measurements of an SM Higgs mass of 125 GeV is predicted at LHC/HL-LHC and LC in the bands

4 $$\begin{aligned}&{\text {LHC/HL-LHC}}:M_H = 125\pm 0.1 / 0.05\;\mathrm{GeV} \end{aligned}$$

5 $$\begin{aligned}&\mathrm{LC}:M_H = 125\pm 0.03\;\mathrm{GeV}. \end{aligned}$$

Extrapolating the Higgs self-coupling associated with this mass value to the Planck scale, a value remarkably close to zero emerges [74–76].

**Fig. 12:**
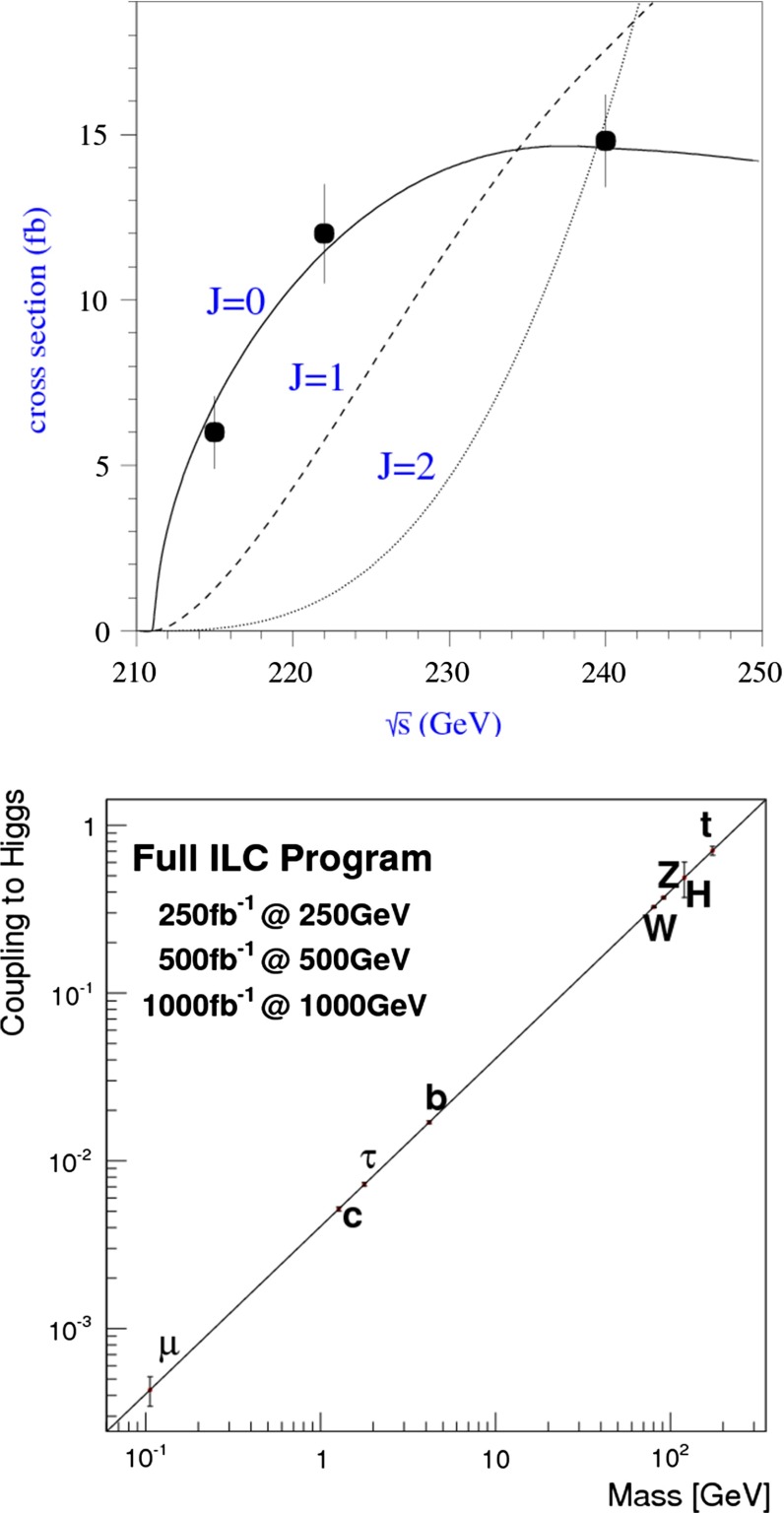
*Upper plot* Threshold rise of the Cross section for Higgs-strahlung $e^+ e^- \rightarrow ZH$ corresponding to Higgs spin $= 0, 1, 2$, complemented by the analysis of angular correlations; *lower plot* Measurements of Higgs couplings as a function of particle masses

Various methods can be applied for confirming the $J^{{\textit{CP}}} = 0^{+\!+}$ quantum numbers of the Higgs boson. While $C = +$ follows trivially from the $H \rightarrow \gamma \gamma$ decay mode, correlations among the particles in decay final states and between initial and final states, as well as threshold effects in Higgs-strahlung [77], cf. Fig. 12 (upper plot), can be exploited for measuring these quantum numbers.

*(b) Higgs couplings to SM particles*

Since the interaction between SM particles *x* and the vacuum Higgs-field generates the fundamental SM masses, the coupling between SM particles and the physical Higgs particle, defined dimensionless, is determined by their masses:

6 $$\begin{aligned} g_{Hxx} = [\sqrt{2} G_F]^\frac{1}{2}\, M_x, \end{aligned}$$

the coefficient fixed in the SM by the vacuum field $v = [\sqrt{2} G_F]^\frac{-1}{2}$. This fundamental relation is a cornerstone of the Higgs mechanism. It can be studied experimentally by measuring production cross sections and decay branching ratios.

At hadron colliders the twin observable $\sigma \times \mathrm{BR}$ is measured for narrow states, and ratios of Higgs couplings are accessible directly. Since in a model-independent analysis $\mathrm{BR}$ potentially includes invisible decays in the total width, absolute values of the couplings can only be obtained with rather large errors. This problem can be solved in $e^+ e^-$ colliders where the invisible Higgs decay branching ratio can be measured directly in Higgs-strahlung. Expectations for measurements at LHC (HL-LHC) and linear colliders are collected in Table 3. The rise of the Higgs couplings with the masses is demonstrated for LC measurements impressively in Fig. 12 (lower plot).

**Table 3:** Expected accuracy with which fundamental and derived Higgs couplings can be measured; the deviations are defind as $\kappa :=g/g_{\mathrm{SM}}=1\pm {\varDelta }$ compared to the SM at the LHC/HL-LHC, LC and in combined analyses of the HL-LHC and LC [29]. The fit assumes generation universality: $\kappa _u\equiv \kappa _c\equiv \kappa _t$, $\kappa _d\equiv \kappa _s\equiv \kappa _b$, and $\kappa _\mu \equiv \kappa _\tau$. The 95 % CL upper limit of potential couplings to invisible channels is also given

Coupling	LHC (%)	HL-LHC (%)	LC (%)	HL-LHC $+$ LC (%)
*HWW*	4–6	2–5	0.3	0.1
*HZZ*	4–6	2–4	0.5	0.3
*Htt*	14–15	7–10	1.3	1.3
*Hbb*	10–13	4–7	0.6	0.6
$H\tau \tau$	6–8	2–5	1.3	1.2
$H\gamma \gamma$	5–7	2–5	3.8	3.0
*Hgg*	6–8	3–5	1.2	1.1
$H \mathrm{invis}$	–	–	0.9	0.9

A special role is played by the loop-induced $\gamma \gamma$ width which can most accurately be measured by Higgs fusion-formation in a photon collider.

From the cross section measured in *WW*-fusion the partial width ${\varGamma }[WW^*]$ can be derived and, at the same time, from the Higgs-strahlung process the decay branching ratio $\mathrm{BR}[WW^*]$ can be determined so that the total width follows immediately from

7 $$\begin{aligned} {\varGamma }_{\mathrm{tot}}[H] = {\varGamma }[WW^*] / \mathrm{BR}[WW^*]. \end{aligned}$$

Based on the expected values at LC, the total width of the SM Higgs particle at 125 GeV is derived as ${\varGamma }_{\mathrm{tot}}[H] = 4.1\,\mathrm{MeV}\,[1\pm 5\,\%]$. Measurements based on off-shell production of Higgs bosons provide only a very rough upper bound on the total width.

Potential deviations of the couplings from the SM values can be attributed to the impact of physics beyond the SM. Parameterizing these effects, as naturally expected in dimensional operator expansions, by $g_H = g_H^{\mathrm{SM}} [1 + v^2 / {{\Lambda }}^2_*]$, the BSM scale is estimated to ${\Lambda }_*>$ 550 GeV for an accuracy of 20 % in the measurement of the coupling, and 2.5 TeV for 1 %, see also [78]. The shift in the coupling can be induced either by mixing effects or by loop corrections to the Higgs vertex. Such mixing effects are well known in the supersymmetric Higgs sector where in the decoupling limit the mixing parameters in the Yukawa vertices approach unity as $\sim v^2/m^2_A$. Other mixing effects are induced in Higgs-portal models and strong interaction Higgs models with either universal or non-universal shifts of the couplings at an amount $\xi = (v/f)^2$, which is determined by the Goldstone scale *f* of global symmetry breaking in the strong-interaction sector; with $f \sim 1$ TeV, vertices may be modified up to the level of 10 %. Less promising is the second class comprising loop corrections of Higgs vertices. Loops, generated for example by the exchange of new $Z'$-bosons, are suppressed by the numerical coefficient $4\pi ^2$ (reduced in addition by potentially weak couplings). Thus the accessible mass range, $M < {\Lambda }_*/ 2\pi \sim$ 250 GeV, can in general be covered easily by direct LHC searches.

*(c) Higgs self-couplings*

The self-interaction of the Higgs field,

8 $$\begin{aligned} V = \lambda [|\phi |^2 - v^2 /2]^2, \end{aligned}$$

is responsible for EWSB by shifting the vacuum state of minimal energy from zero to $v/\sqrt{2} \simeq$ 174 GeV. The quartic form of the potential, required to render the theory renormalisable, generates tri-linear and quadri-linear self-couplings when $\phi \rightarrow [v+H]/\sqrt{2}$ is shifted to the physical Higgs field *H*. The strength of the couplings are determined uniquely by the Higgs mass, with $M_H^2 = 2 \lambda v^2$:

9 $$\begin{aligned} \lambda _3 = M_H^2 / 2 v, \quad \lambda _4 = M_H^2 / 8 v^2 \quad {\text {and}} \quad \lambda _{n > 4} = 0. \end{aligned}$$

The tri-linear Higgs coupling can be measured in Higgs pair-production [79]. Concerning the LHC, the cross section is small and thus the high luminosity of HL-LHC is needed to achieve some sensitivity to the coupling. Prospects are brighter in Higgs pair-production in Higgs-strahlung and *W*-boson fusion of $e^+ e^-$ collisions, i.e. $e^+ e^- \rightarrow Z + H^*\rightarrow Z + HH$, etc. In total, a precision of

10 $$\begin{aligned} {\text {LC}}:\lambda _3 = 10-13\,\% \end{aligned}$$

may be expected. On the other hand, the cross section for triple Higgs production is so small, $\mathscr {O}$(ab), that the measurement of $\lambda _4$ values near the SM prediction will not be feasible at either type of colliders.

*(d) Invisible Higgs decays*

The observation of cold DM suggests the existence of a hidden sector with a priori unknown, potentially high complexity. The Higgs field of the SM can be coupled to a corresponding Higgs field in the hidden sector, $\tilde{\mathscr {V}} = \eta |\phi _{\mathrm{SM}}|^2 |\phi _{hid}|^2$, in a form compatible with all standard symmetries. Thus a portal could be opened from the SM to the hidden sector [80, 81]. Analogous mixing with radions is predicted in theories incorporating extra-space dimensions. The mixing of the Higgs fields in the two sectors induces potentially small universal changes in the observed Higgs couplings to the SM particles and, moreover, Higgs decays to invisible hidden states (while this channel is opened in the canonical SM only indirectly by neutrino decays of *Z* pairs). Both signatures are a central target for experimentation at LC, potentially allowing the first sighting of a new world of matter in the Higgs sector.

In summary, essential elements of the Higgs mechanism in the SM can be determined at $e^+ e^-$ linear colliders in the 250 to 500 GeV and 1 to 3 TeV modes at high precision. Improvements on the fundamental parameters by nearly an order of magnitude can be achieved in such a faciliy. Thus a fine-grained picture of the Higgs sector as third component of the SM can be drawn at a linear collider, completing the theory of matter and forces at the electroweak scale. First glimpses of a sector beyond the SM are possible by observing deviations from the SM picture at scales far beyond those accessible at colliders directly.

#### Supersymmetry scenarios

The hypothetical extension of the SM to a supersymmetric theory [82, 83] is intimately connected with the Higgs sector. If the SM is embedded in a grand unified scenario, excessive fine tuning in radiative corrections would be needed to keep the Higgs mass near the electroweak scale, i.e. 14 orders of magnitude below the grand-unification scale. A stable bridge can be constructed, however, in a natural way if matter and force fields are assigned to fermion–boson symmetric multiplets with masses not spread more than order TeV. In addition, by switching the mass (squared) of a scalar field from positive to negative value when evolved from high to low scales, supersymmetry offers an attractive physical explication of the Higgs mechanism. It should be noted that supersymmetrisation of the SM is not the only solution of the hierarchy problem, however, it joins in nicely with arguments of highly precise unification of couplings, the approach to gravity in local supersymmetry, and the realisation of cold DM. Even though not yet backed at present by the direct experimental observation of supersymmetric particles, supersymmetry remains an attractive extension of the SM, offering solutions to a variety of fundamental physical problems.

To describe the Higgs interaction with matter fields by a superpotential, and to keep the theory anomaly-free, at least two independent Higgs iso-doublets must be introduced, coupling separately to up- and down-type matter fields. They are extended eventually by additional scalar superfields, etc.

*(a) Minimal supersymmetric model MSSM*

Extending the SM fields to super-fields and adding a second Higgs doublet defines the minimal supersymmetric standard model (MSSM). After gauge symmetry breaking, three Goldstone components out of the eight scalar fields are aborbed to provide masses to the electroweak gauge bosons while five degrees of freedom are realised as new physical fields, corresponding to two neutral ${\textit{CP}}$-even scalar particles $h^0,H^0$; one neutral ${\textit{CP}}$-odd scalar particle $A^0$; and a pair of charged $H^\pm$ scalar particles [84–87].

Since the quadri-linear Higgs couplings are pre-determined by the (small) gauge couplings, the mass of the lightest Higgs particle is small. The bound, $M_{h^0} < M_Z | \cos 2\beta |$ at lowest order, with $\tan \beta$ accounting for Goldstone–Higgs mixing, is significantly increased, however, to $\sim$130 GeV by radiative corrections, adding a contribution of order $3 M^4_t/2 \pi ^2 v^2\, \log M^2_{\tilde{t}}/M^2_t + mix$ for large top and stop masses. To reach a value of 125 GeV, large stop masses and/or large tri-linear couplings are required in the mixings.

Predictions for production and decay amplitudes deviate, in general, from the SM not only because of modified tree couplings but also due to additional loop contributions, as $\tilde{\tau }$ loops in the $\gamma \gamma$ decay mode of the lightest Higgs boson.

To accommodate a 125-GeV Higgs boson in minimal supergravity the quartet of heavy Higgs particles $H^0,A^0,H^\pm$ is shifted to the decoupling regime with order TeV masses. The properties of the lightest Higgs boson $h^0$ are very close in this regime to the properties of the SM Higgs boson.

**Fig. 13:**
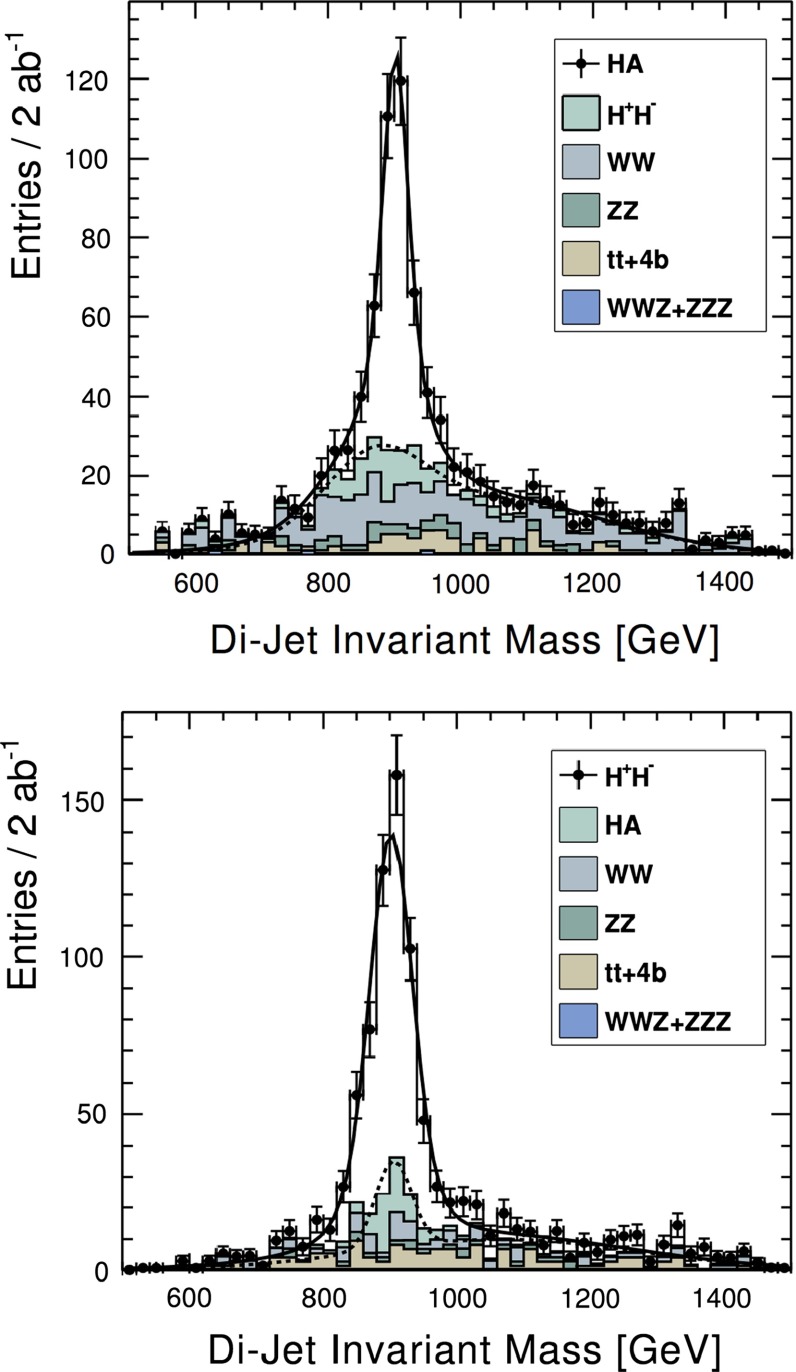
*Upper plot* reconstructed 2-jet invariant mass for associated production: $e^+ e^- \rightarrow AH \rightarrow b\bar{b}b\bar{b}$ for a Higgs mass of 900 GeV at a collider energy of 3 TeV; *lower plot* similar plot for $e^+ e^- \rightarrow H^+H^- \rightarrow t\bar{b}\bar{t}b$

The heavy Higgs-boson quartet is difficult to search for at LHC. In fact, these particles cannot be detected in a blind wedge which opens at 200 GeV for intermediate values of the mixing parameter $\tan \beta$ and which covers the parameter space for masses beyond 500 GeV. At the LC, Higgs-strahlung $e^+ e^- \rightarrow Z\,h^0$ is supplemented by Higgs pair-production:

11 $$\begin{aligned} e^+\,e^- \rightarrow A^0\,H^0\quad {\text {and}}\quad H^+\,H^- \end{aligned}$$

providing a rich source of heavy Higgs particles in $e^+ e^-$ collisions for masses $M < \sqrt{s}/2$, cf. Fig. 13. Heavy Higgs masses come with *ZAH* couplings of the order of gauge couplings so that the cross sections are large enough for copious production of heavy neutral ${\textit{CP}}$ even/odd and charged Higgs-boson pairs.

Additional channels open in single Higgs production $\gamma \gamma \rightarrow A^0,H^0$, completely exhausting the multi-TeV energy potential ${\sqrt{s}}_{\gamma \gamma }$ of a photon collider.

*(b) Extended supersymmetry scenarios*

The minimal supersymmetry model is quite restrictive by connecting the quadri-linear couplings with the gauge couplings, leading naturally to a small Higgs mass, and grouping the heavy Higgs masses close to each other. The simplest extension of the system introduces an additional iso-scalar Higgs field [88, 89], the next-to-minimal model (NMSSM). This extension augments the Higgs spectrum by two additional physical states, ${\textit{CP}}$-even and ${\textit{CP}}$-odd, which mix with the corresponding MSSM-type states.

The bound on the mass of the lightest MSSM Higgs particle is alleviated by contributions from the tri-linear Higgs couplings in the superpotential (reducing the amount of ‘little fine tuning’ in this theory). Loop contributions to accommodate a 125-GeV Higgs boson are reduced so that the bound on stop masses is lowered to about 100 GeV as a result.

The additional parameters in the NMSSM render the predictions for production cross sections and decay branching ratios more flexible, so that an increased rate of $pp \rightarrow \mathrm{Higgs} \rightarrow \gamma \gamma$, for instance, can be accomodated more easily than within the MSSM.

Motivations for many other extensions of the Higgs sector have been presented in the literature. Supersymmetry provides an attractive general framework in this context. The new structures could be so rich that the clear experimental environment of $e^+ e^-$ collisions is needed to map out this Higgs sector and to unravel its underlying physical basis.

#### Composite Higgs bosons

Not long after pointlike Higgs theories had been introduced to generate the breaking of the electroweak symmetries, alternatives have been developed based on novel strong interactions [90, 91]. The breaking of global symmetries in such theories gives rise to massless Goldstone bosons which can be absorbed by gauge bosons to generate their masses. This concept had been expanded later to incorporate also light Higgs bosons with mass in the intermediate range. Generic examples for such theories are Little Higgs Models and theories formulated in higher dimensions, which should be addressed briefly as generic examples.

*(a) Little Higgs models*

If new strong interactions are introduced at a scale of a few 10 TeV, the breaking of global symmetries generates a Goldstone scale *f* typically reduced by one order of magnitude, i.e. at a few TeV. The spontaneous breaking of large global groups leads to an extended scalar sector with Higgs masses generated radiatively at the Goldstone scale. The lightest Higgs mass is delayed, by contrast, acquiring mass at the electroweak scale only through collective symmetry breaking at higher oder.

Such a scenario [92] can be realised, for instance, in minimal form as a non-linear sigma model with a global *SU*(5) symmetry broken down to *SO*(5). After separating the Goldstone modes which provide masses to gauge bosons, ten Higgs bosons emerge in this scenario which split into an isotriplet $\Phi$, including a pair of doubly charged $\Phi ^{\pm \pm }$ states with TeV-scale masses, and the light standard doublet *h*. The properties of *h* are affected at the few per-cent level by the extended spectrum of the fermion and gauge sectors. The new TeV triplet Higgs bosons with doubly charged scalars can be searched for very effectively in pair production at LC in the TeV energy range.

*(b) Relating to higher dimensions*

An alternative approach emerges out of gauge theories formulated in five-dimensional anti-de-Sitter space. The AdS/CFT correspondence relates this theory to a four-dimensional strongly coupled theory, the fifth components of the gauge fields interpreted as Goldstone modes in the strongly coupled four-dimensional sector. In this picture the light Higgs boson appears as a composite state with properties deviating to order $(v/f)^2$ from the standard values [93], either universally or non-universally with alternating signs for vector bosons and fermions.

### The SM Higgs at the LHC: status and prospects^7^

In July 2012 the ATLAS and CMS experiments at the LHC announced the discovery of a new particle with a mass of about 125 GeV that provided a compelling candidate for the Higgs boson in the framework of the standard model of particle physics (SM). Both experiments found consistent evidence from a combination of searches for three decay modes, $H\rightarrow \gamma \gamma$, $H\rightarrow ZZ\rightarrow 4l$ and $H\rightarrow WW\rightarrow 2 l2\nu$ ($l=e,\mu$), with event rates and properties in agreement with SM predictions for Higgs-boson production and decay. These findings, which were based on proton–proton collision data recorded at centre-of-mass energies of 7 and 8 TeV and corresponding to an integrated luminosity of about 10 fb$^{-1}$ per experiment, received a lot of attention both within and outside the particle physics community and were eventually published in [62, 94–96].

Since then, the LHC experiments have concluded their first phase of data taking (“Run1”) and significantly larger datasets corresponding to about 25 fb$^{-1}$ per experiment have been used to perform further improved analyses enhancing the signals in previously observed decay channels, establishing evidence of other decays and specific production modes as well as providing more precise measurements of the mass and studies of other properties of the new particle. Corresponding results, some of them still preliminary, form the basis of the first part of this section, which summarises the status of the ATLAS and CMS analyses of the Higgs boson candidate within the SM.

The second part gives an outlook on Higgs-boson studies during the second phase (“Run2”) of the LHC operation scheduled to start later this year and the long-term potential for an upgraded high-luminosity LHC.

While the following discussion is restricted to analyses within the framework of the SM, the consistency of the observed Higgs-boson candidate with SM expectations (as evaluated in [38, 97, 98] and references therein) does not exclude that extensions of the SM with a richer Higgs sector are realised in nature and might show up experimentally at the LHC. Thus, both the ATLAS and the CMS Collaborations have been pursuing a rich programme of analyses that search for deviations from the SM predictions and for additional Higgs bosons in the context of models beyond the SM. A review of this work is, however, beyond the scope of this section.

**Fig. 14:**
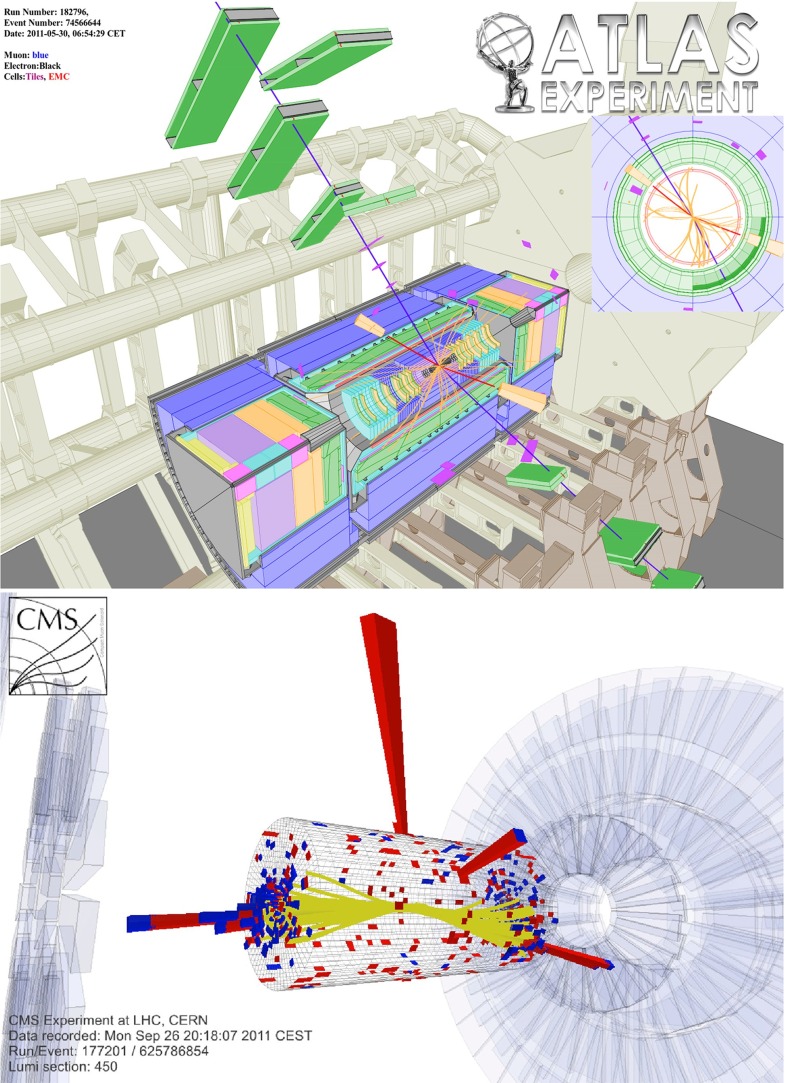
Displays of example Higgs-boson candidate events. *Top* $H\rightarrow ZZ\rightarrow 2\mu 2e$ candidate in the ATLAS detector; *bottom* VBF $H\rightarrow \gamma \gamma$ candidate in the CMS detector

#### Current status

The initial SM Higgs-boson searches at the LHC were designed for a fairly large Higgs mass window between 100 and 600 GeV, most of which was excluded by the ATLAS and CMS results based on the data sets recorded in 2011 [99, 100]. In the following we focus on the analyses including the full 2012 data and restrict the discussion to decay channels relevant to the discovery and subsequent study of the 125 GeV Higgs boson.

*Relevant decay channels* For all decay channels described below, the analysis strategies have evolved over time in similar ways. Early searches were based on inclusive analyses of the Higgs-boson decay products. With larger datasets, these were replaced by analyses in separate categories corresponding to different event characteristics and background composition. Such categorisation significantly increases the signal sensitivity and can also be used to separate different production processes, which is relevant for the current and future studies of the Higgs-boson couplings discussed below. Also, with larger data sets and higher complexity of the analyses, it became increasingly important to model the background contributions from data control regions instead of relying purely on simulated events. Another common element is the application of multivariate techniques in more recent analyses. Still, the branching ratios, detailed signatures and relevant background processes for different decays differ substantially; two example Higgs-boson production and decay candidate event displays are shown in Fig. 14. Therefore, the experimental approaches and resulting information on the 125-GeV Higgs boson vary as well:

- $H\rightarrow \gamma \gamma$: the branching fraction is very small but the two high-energy photons provide a clear experimental signature and a good mass resolution. Relevant background processes are diphoton continuum production as well as photon-jet and dijet events. The most recent ATLAS [101] and CMS [104] analyses yield signals with significances of $5.2\sigma$ and $5.7\sigma$, respectively, where $4.6\sigma$ and $5.2\sigma$ are expected.
- $H\rightarrow ZZ\rightarrow 4\ell$: also this decay combines a small branching fraction with a clear experimental signature and a good mass resolution. The selection of events with two pairs of isolated, same-flavour, opposite-charge electrons or muons results in the largest signal-to-background ratio of all currently considered Higgs-boson decay channels. The remaining background originates mainly from continuum *ZZ*, *Z*+jets and $t\bar{t}$ production processes. ATLAS [105] and CMS [102] report observed (expected) signal significances of $8.1\sigma$ ($6.2\sigma$) and $6.8\sigma$ ($6.7\sigma$).
- $H\rightarrow WW\rightarrow 2\ell 2\nu$: the main advantage of this decay is its large rate, and the two oppositely charged leptons from the *W* decays provide a good experimental handle. However, due to the two undetectable final-state neutrinos it is not possible to reconstruct a narrow mass peak. The dominant background processes are *WW*, *Wt*, and $t\bar{t}$ production. The observed (expected) ATLAS [103] and CMS [106] signals have significances of $6.1\sigma$ ($5.8\sigma$) and $4.3\sigma$ ($5.8\sigma$).

**Fig. 15:**
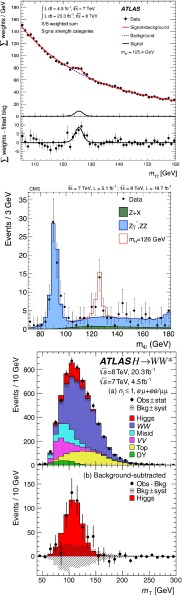
Reconstructed distributions of the Higgs boson candidate decay products for the complete 2011/2012 data, expected backgrounds, and simulated signal from *top* the ATLAS $H\rightarrow \gamma \gamma$ [101], *centre* the CMS $H\rightarrow ZZ\rightarrow 4\ell$ [102], and *bottom* the ATLAS $H\rightarrow WW\rightarrow 2\ell 2\nu$ [103] analyses

Figure 15 shows reconstructed Higgs candidate mass distributions from ATLAS and CMS searches for $H\rightarrow \gamma \gamma$ and $H\rightarrow ZZ\rightarrow 4\ell$, respectively, as well as the ATLAS $H\rightarrow WW\rightarrow 2\ell 2\nu$ transverse mass distribution. Other bosonic decay modes are searched for as well but these analyses are not yet sensitive to a SM Higgs boson observation.

- $H\rightarrow bb$: for a Higgs-boson mass of 125 GeV this is the dominant Higgs-boson decay mode. The experimental signature of *b* quark jets alone is difficult to exploit at the LHC, though, so that current analyses focus on the Higgs production associated with a vector boson *Z* or *W*. Here, diboson, vector boson+jets and top production processes constitute the relevant backgrounds.
- $H\rightarrow \tau \tau$: all combinations of hadronic and leptonic $\tau$-lepton decays are used to search for a broad excess in the $\tau \tau$ invariant mass spectrum. The dominant and irreducible background is coming from $Z\rightarrow \tau \tau$ decays; further background contributions arise from processes with a vector boson and jets, top and diboson production.

**Fig. 16:**
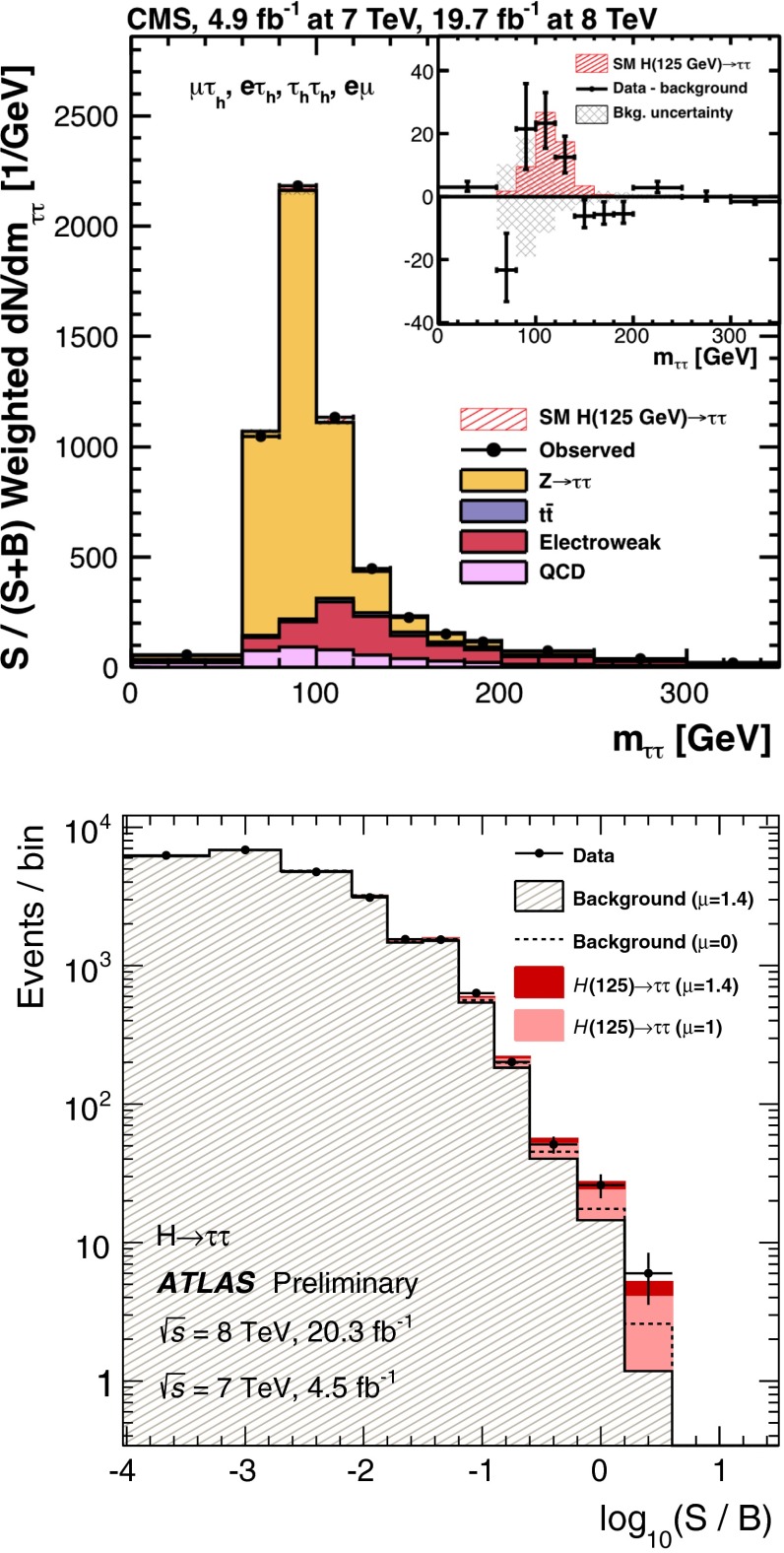
Evidence for the decay $H\rightarrow \tau \tau$. *Top* CMS observed and predicted $m_{\tau \tau }$ distributions [109]. The distributions obtained in each category of each channel are weighted by the ratio between the expected signal and signal-plus-background yields in the category. The *inset* shows the corresponding difference between the observed data and expected background distributions, together with the signal distribution for a SM Higgs boson at $m_H=125$ GeV; *bottom* ATLAS event yields as a function of $\log (S/B)$, where *S* (signal yield) and *B* (background yield) are taken from the corresponding bin in the distribution of the relevant BDT output discriminant [110]

While searches for $H\rightarrow bb$ decays [107, 108] have not yet resulted in significant signals, first evidence for direct Higgs-boson decays to fermions has been reported by both ATLAS and CMS following analyses of $\tau \tau$ final states. The CMS results [109] are predominantly based on fits to the reconstructed $\tau \tau$ invariant mass distributions, whereas the ATLAS analysis [110] uses the output of boosted decision trees (BDTs) throughout for the statistical analysis of the selected data. ATLAS (CMS) find signals with a significance of 4.5$\sigma$ (3.5$\sigma$), where 3.4$\sigma$ (3.7$\sigma$) are expected, cf. Fig. 16. In [111] CMS present the combination of their $H\rightarrow \tau \tau$ and $H\rightarrow bb$ analyses yielding an observed (expected) signal significance of $3.8\sigma$ ($4.4\sigma$). Searches for other fermionic decays are performed as well but are not yet sensitive to the observation of the SM Higgs boson.

In the following, we summarise the status of SM Higgs boson analyses of the full 2011/2012 datasets with ATLAS and CMS. The discussion is based on preliminary combinations of ATLAS and published CMS results collected in [112, 113], respectively; an ATLAS publication of Higgs-boson mass measurements [114]; ATLAS [115] and CMS [116] constraints on the Higgs boson width; studies of the Higgs boson spin and parity by CMS [117] and ATLAS [65, 118, 119]; and other results on specific aspects or channels referenced later in this section.

*Signal strength* For a given Higgs-boson mass, the parameter $\mu$ is defined as the observed Higgs-boson production strength normalised to the SM expectation. Thus, $\mu =1$ reflects the SM expectation and $\mu =0$ corresponds to the background-only hypothesis.

**Fig. 17:**
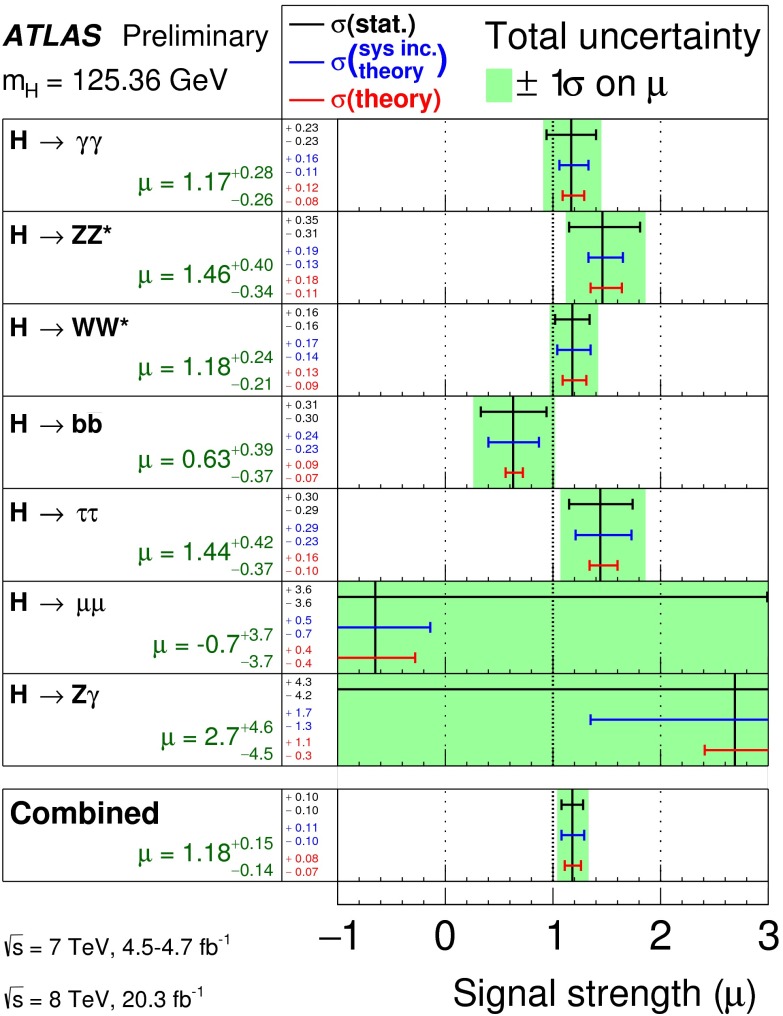
Higgs boson signal strength as measured by ATLAS for different decay channels [112]

**Fig. 18:**
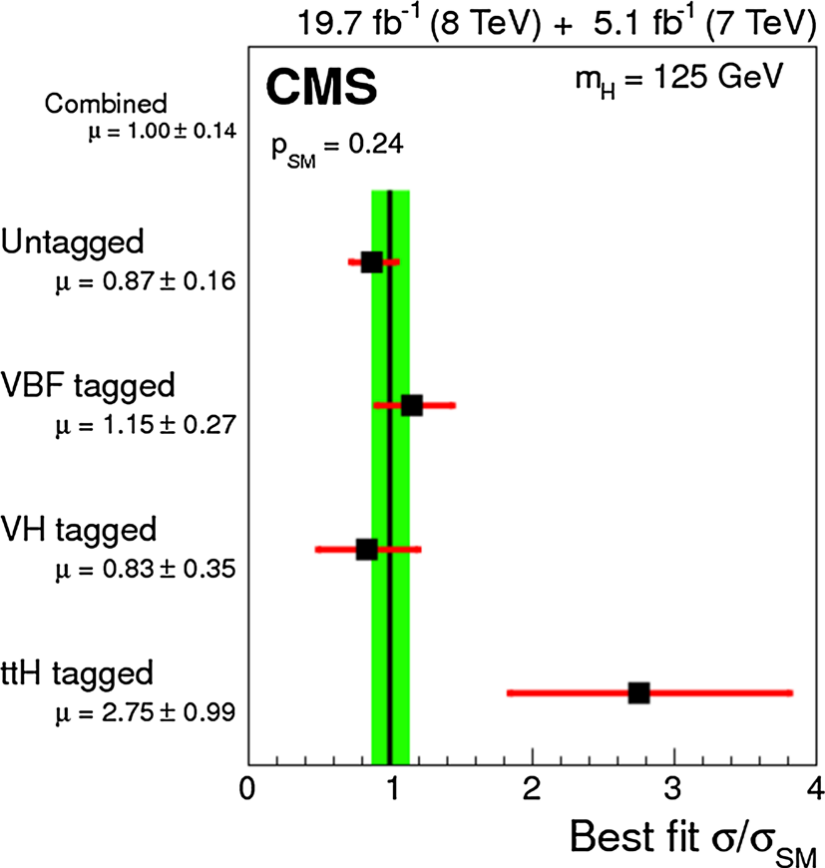
Higgs-boson production strength, normalised to the SM expectation, based on CMS analyses [113], for a combination of analysis categories related to different production modes.

Fixing the Higgs-boson mass to the measured value and considering the decays $H\rightarrow \gamma \gamma$, $H\rightarrow ZZ\rightarrow 4\ell$, $H\rightarrow WW \rightarrow 2\ell 2\nu$, $H\rightarrow bb$, and $H\rightarrow \tau \tau$, ATLAS report [112] a preliminary overall production strength of

$$\begin{aligned} \mu =1.18^{+0.15}_{-0.14}; \end{aligned}$$

the separate combination of the bosonic and fermionic decay modes yields $\mu =1.35^{+0.21}_{-0.20}$ and $\mu =1.09^{+0.36}_{-0.32}$, respectively. The corresponding CMS result [113] is

$$\begin{aligned} \mu =1.00\pm 0.13. \end{aligned}$$

Good consistency is found, for both experiments, across different decay modes and analyses categories related to different production modes, see Figs. 17 and 18.

**Fig. 19:**
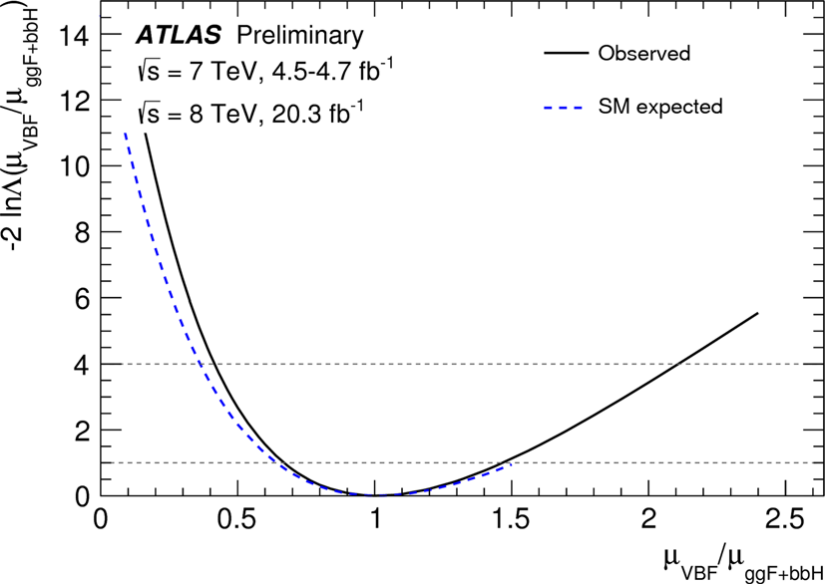
Likelihood for the ratio $\mu _{\text{ VBF }}/\mu _{ggF+ttH}$ obtained by ATLAS for the combination of the $H\rightarrow \gamma \gamma$, $ZZ\rightarrow 4\ell$ and $WW\rightarrow 2\nu 2\ell$ channels and $m_H = 125.5$ GeV [112]

ATLAS and CMS have also studied the relative contributions from production mechanisms mediated by vector bosons (VBF and VH processes) and gluons (*ggF* and *ttH* processes), respectively. For example, Fig. 19 shows ATLAS results constituting a 4.3$\sigma$ evidence that part of the Higgs-boson production proceeds via VBF processes [112].

*Couplings to other particles* The Higgs-boson couplings to other particles enter the observed signal strengths via both the Higgs production and decay. Leaving other SM characteristics unchanged, in particular assuming the observed Higgs-boson candidate to be a single, narrow, ${\textit{CP}}$-even scalar state, its couplings are tested by introducing free parameters $\kappa _X$ for each particle *X*, such that the SM predictions for production cross sections and decay widths are modified by a multiplicative factor $\kappa ^2_X$. This includes effective coupling modifiers $\kappa _{g}$, $\kappa _\gamma$ for the loop-mediated interaction with gluons and photons. An additional scale factor modifies the total Higgs boson width by $\kappa ^2_H$.

**Fig. 20:**
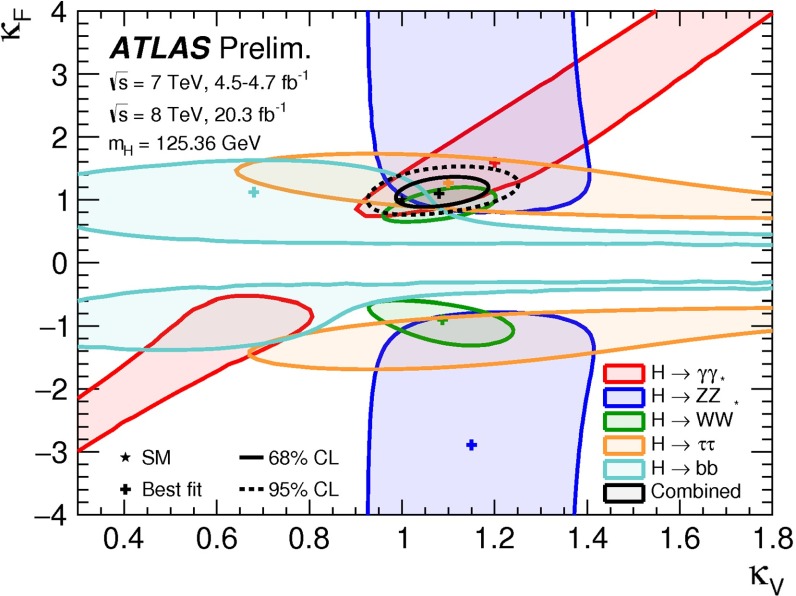
Preliminary ATLAS results of fits for a two-parameter benchmark model that probes different coupling strength scale factors common for fermions ($\kappa _F$) and vector bosons ($\kappa _V$), respectively, assuming only SM contributions to the total width. Shown are 68 and 95 % CL contours of the two-dimensional fit; overlaying the 68 % CL contours derived from the individual channels and their combination. The best-fit result ($\times$) and the SM expectation ($+$) are also indicated [112]

Several different set of assumptions, detailed in [37, 38], form the basis of such coupling analyses. For example, a fit to the ATLAS data [112] assuming common scale factors $\kappa _F$ and $\kappa _V$ for all fermions and bosons, respectively, yields the results depicted in Fig. 20.

**Fig. 21:**
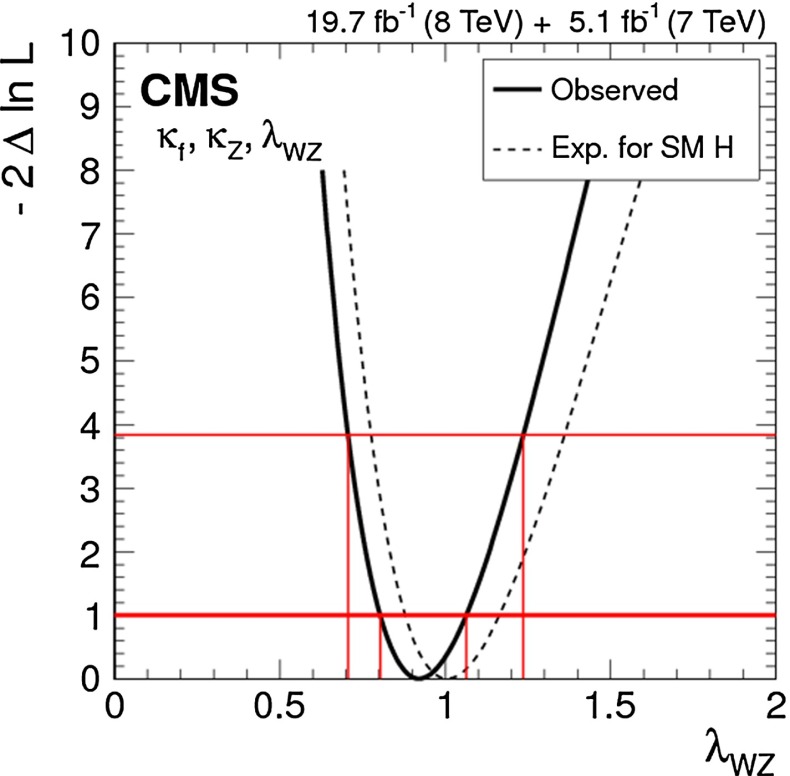
Test of custodial symmetry: CMS likelihood scan of the ratio $\lambda _{WZ}$, where SM coupling of the Higgs bosons to fermions are assumed [113]

Within the SM, $\lambda _{WZ}=\kappa _W/\kappa _Z=1$ is implied by custodial symmetry. Agreement with this prediction is found by both CMS, see Fig. 21, and ATLAS. Similar ratio analyses are performed for the couplings to leptons and quarks ($\lambda _{lq}$) as well as to down and up-type fermions ($\lambda _{du}$).

**Fig. 22:**
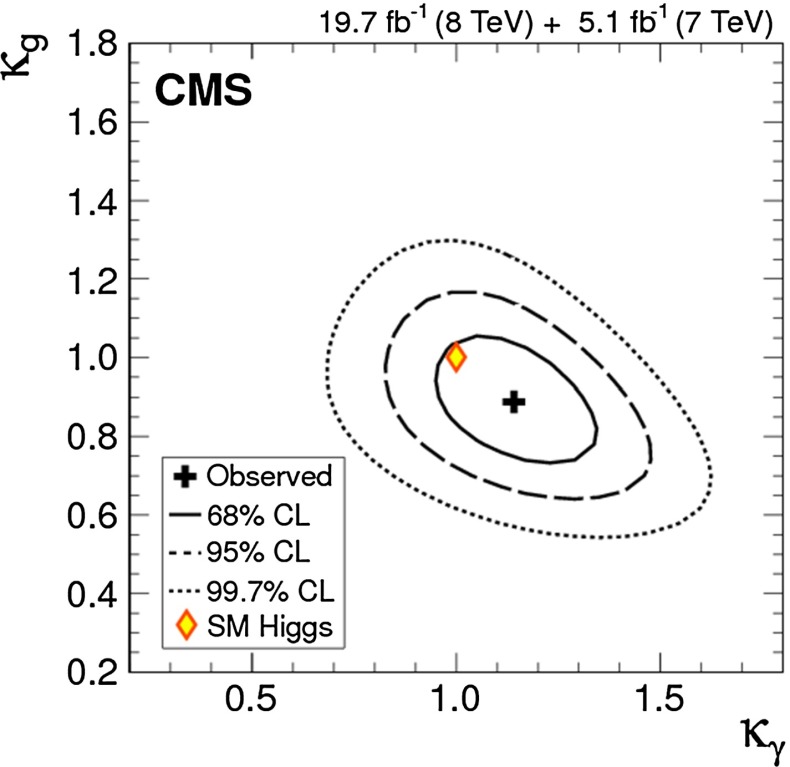
Constraining BSM contributions to particle loops: CMS 2d likelihood scan of gluon and photon coupling modifiers $\kappa _{g}$, $\kappa _{\gamma }$ [113]

Within a scenario where all modifiers $\kappa$ except for $\kappa _{g}$ and $\kappa _{\gamma }$ are fixed to 1, contributions from beyond-SM particles to the loops that mediate the *ggH* and $H\gamma \gamma$ interactions can be constrained; a corresponding CMS result [113] is shown in Fig. 22.

**Fig. 23:**
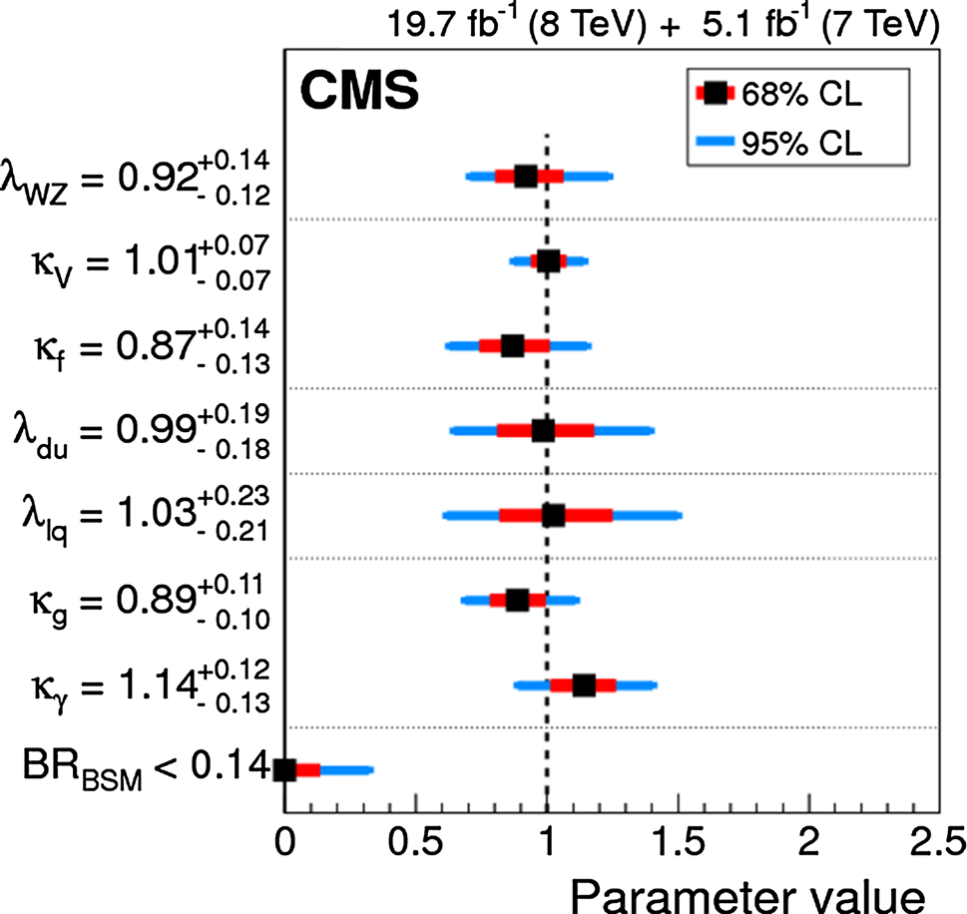
Summary plot of CMS likelihood scan results [113] for the different parameters of interest in benchmark models documented in [38]. The *inner bars* represent the 68 % CL confidence intervals, while the *outer bars* represent the 95 % CL confidence intervals

Summaries of CMS results [113] from such coupling studies are presented in Fig. 23. Within each of the specific sets of assumptions, consistency with the SM expectation is found. Corresponding studies by CMS [113] yield the same conclusions. It should be noted, however, that this does not yet constitute a complete, unconstrained analysis of the Higgs-boson couplings.

**Fig. 24:**
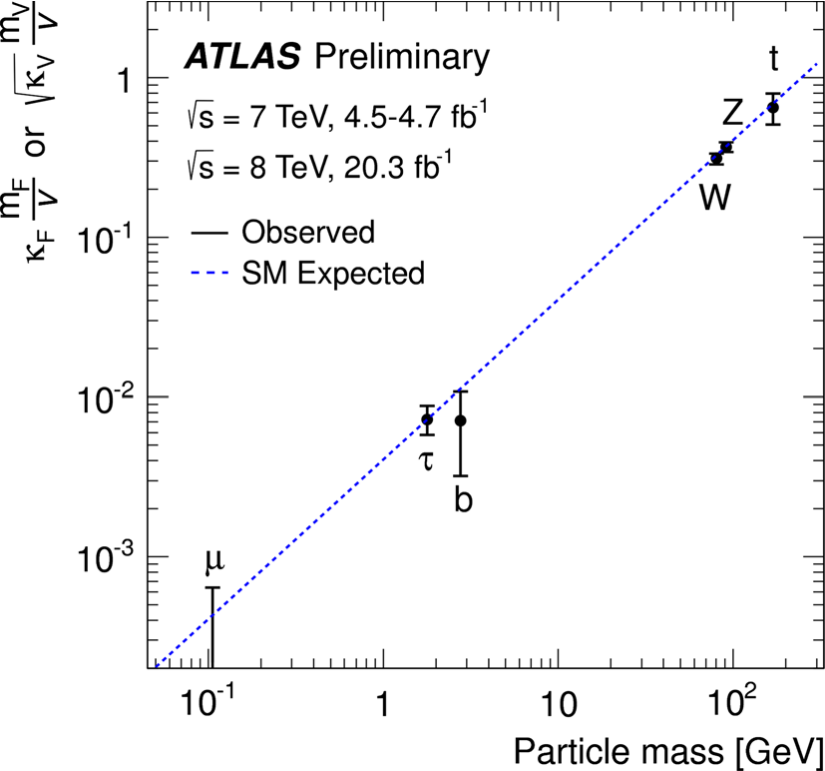
ATLAS summary of the fits for modifications of the SM Higgs-boson couplings expressed as a function of the particle mass. For the fermions, the values of the fitted Yukawa couplings for the $Hf\bar{f}$ vertex are shown, while for vector bosons the square-root of the coupling for the *HVV* vertex divided by twice the vacuum expectation value of the Higgs boson field [112]

For the fit assuming that loop-induced couplings follow the SM structure as in [38] without any BSM contributions to Higgs-boson decays or particle loops, ATLAS, see Fig. 24, and CMS also demonstrate that the results follow the predicted relationship between Higgs-boson couplings and the SM particle masses.

**Fig. 25:**
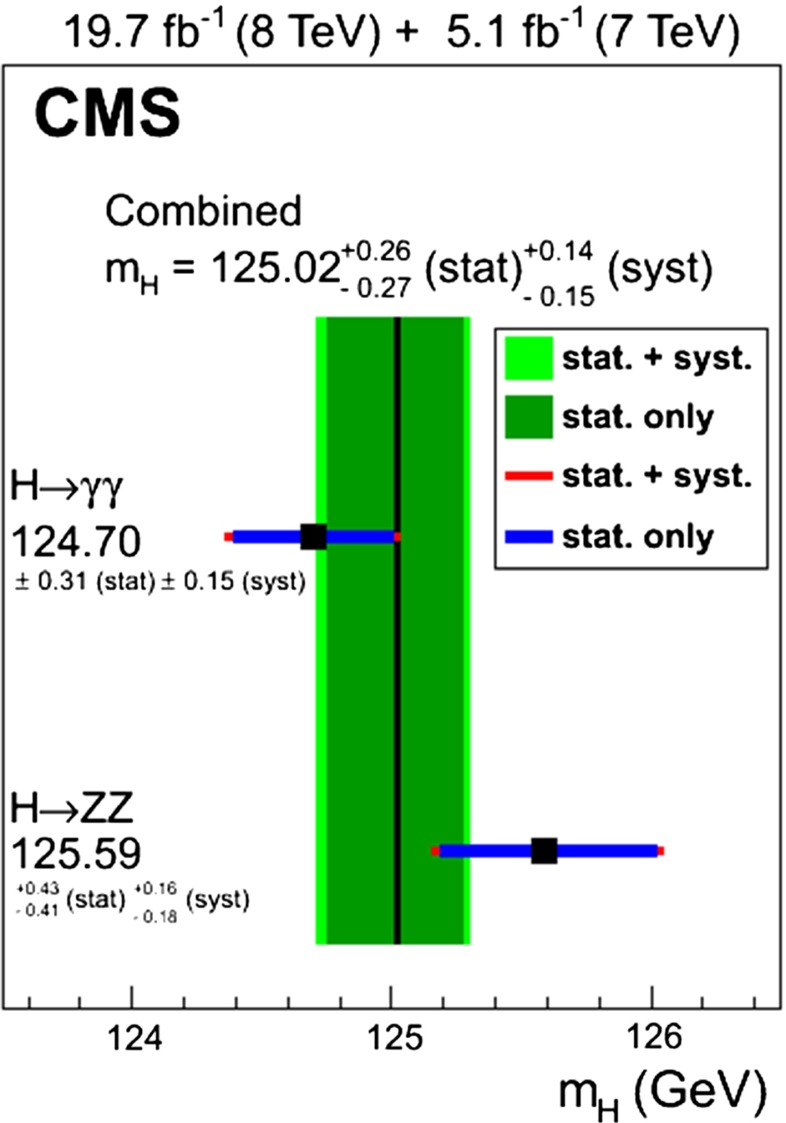
CMS mass measurements [113] in the $\gamma \gamma$ and $ZZ\rightarrow 4\ell$ final states and their combinations. The *vertical band* shows the combined uncertainty. The *horizontal bars* indicate the $\pm 1$ standard deviation uncertainties for the individual channels

*Mass* Current measurements of the Higgs-boson mass are based on the two high-resolution decay channels $H\rightarrow \gamma \gamma$ and $H\rightarrow ZZ\rightarrow 4\ell$. Based on fits to the invariant diphoton and four-lepton mass spectra, ATLAS measures [114] $m_H=125.98\pm 0.42{\mathrm {(stat)}}\pm 0.28{\mathrm {(sys)}}$ and $m_H=124.51\pm 0.52{\mathrm {(stat)}}\pm 0.06{\mathrm {(sys)}}$, respectively. A combination of the two results, which are consistent within 2.0 standard deviations, yields $m_H=125.36\pm 0.37{\mathrm {(stat)}}\pm 0.18{\mathrm {(sys)}}.$ An analysis [113] of the same decays by CMS finds consistency between the two channels at 1.6$\sigma$; see Fig. 25. The combined result $m_H=125.02^{+0.26}_{-0.27}{\mathrm {(stat)}}^{+0.14}_{-0.15}{\mathrm {(sys)}}$ agrees well with the corresponding ATLAS measurement.

A preliminary combination [120] of both experiments gives a measurement of the Higgs-boson mass of

$$\begin{aligned} m_H=125.09 \pm 0.21{\mathrm {(stat)}}\pm 0.11{\mathrm {(sys)}}, \end{aligned}$$

with a relative uncertainty of 0.2 %.

Other decay channels currently do not provide any significant contributions to the overall mass precision but they can still be used for consistency tests. For example, CMS obtains $m_H=128^{+7}_{-5}$ and $m_H=122\pm 7$ GeV from the analysis of *WW* [106] and $\tau \tau$ [109] final states, respectively.

*Width* Information on the decay width of the Higgs boson obtained from the above mass measurements is limited by the experimental resolution to about 2 GeV, whereas the SM prediction for ${\varGamma }_H$ is about 4 MeV.

**Fig. 26:**
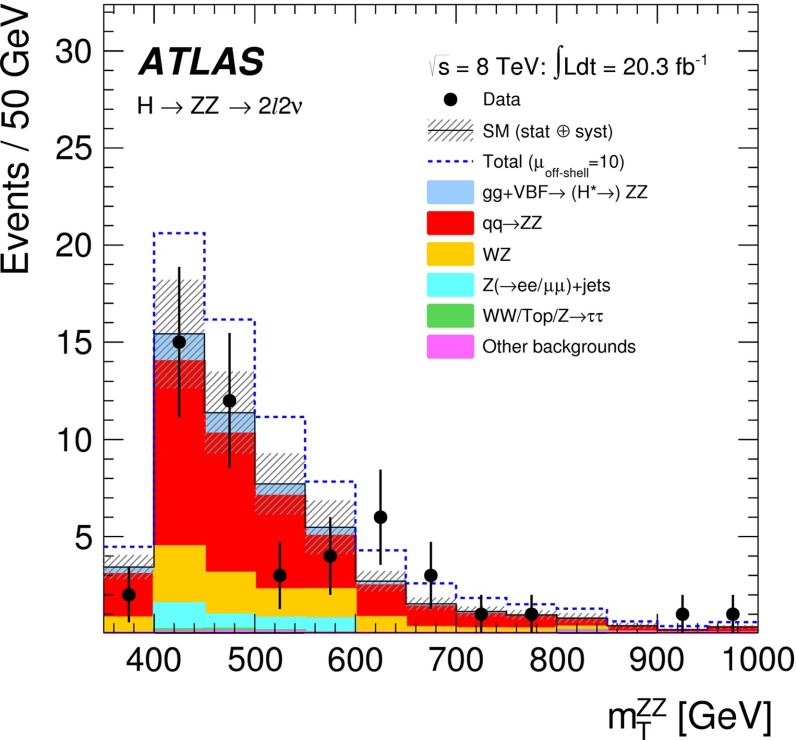
Observed transverse mass distributions for the ATLAS $ZZ\rightarrow 2\ell 2\nu$ analysis [115] in the signal region compared to the expected contributions from *ggF* and VBF Higgs production with the decay $H^*\rightarrow ZZ$ SM and with $\mu _{\text {off-shell}}=10$ (*dashed*) in the $2e2\nu$ channel. A relative $gg\rightarrow ZZ$ background *K*-factor of 1 is assumed

Analyses of *ZZ* and *WW* events in the mass range above the 2$$m_{Z,W}$$ threshold provide an alternative approach [34, 121], which was first pursued by CMS [116] based on the $$ZZ\rightarrow 4\ell $$ and $$ZZ\rightarrow 2\ell 2\nu $$ channels; a later ATLAS analysis [115] included also the $$WW\rightarrow e\nu \mu \nu $$ final state. The studied distributions vary between experiments and channels; for example, Fig. 26 shows the high-mass $$ZZ\rightarrow 2\ell 2\nu $$ transverse mass distribution observed by ATLAS with the expected background contributions and the predicted signal for different assumptions for the off-shell $$H\rightarrow ZZ$$ signal strength $$\mu _{\mathrm {off-shell}}$$. The resulting constraints on $$\mu _{\mathrm {off-shell}}$$, together with the on-shell $$H\rightarrow ZZ\rightarrow 4\ell $$$$\mu _{\mathrm {on-shell}}$$ measurement, can be interpreted as a limit on the Higgs boson width if the relevant off-shell and on-shell Higgs couplings are assumed to be equal.^8^

Combining *ZZ* and *WW* channels, ATLAS find an observed (expected) 95 % CL limit of

$$\begin{aligned} 5.1(6.7)<\mu _{\text {off-shell}}<8.6(11.0) \end{aligned}$$

when varying the unknown *K*-factor ratio between the $gg\rightarrow ZZ$ continuum background and the $gg\rightarrow H^*\rightarrow ZZ$ signal between 0.5 and 2.0. This translates into

$$\begin{aligned} 4.5(6.5)<{\varGamma }_H/{\varGamma }^\mathrm{SM}_H<7.5(11.2) \end{aligned}$$

if identical on-shell and off-shell couplings are assumed.

**Fig. 27:**
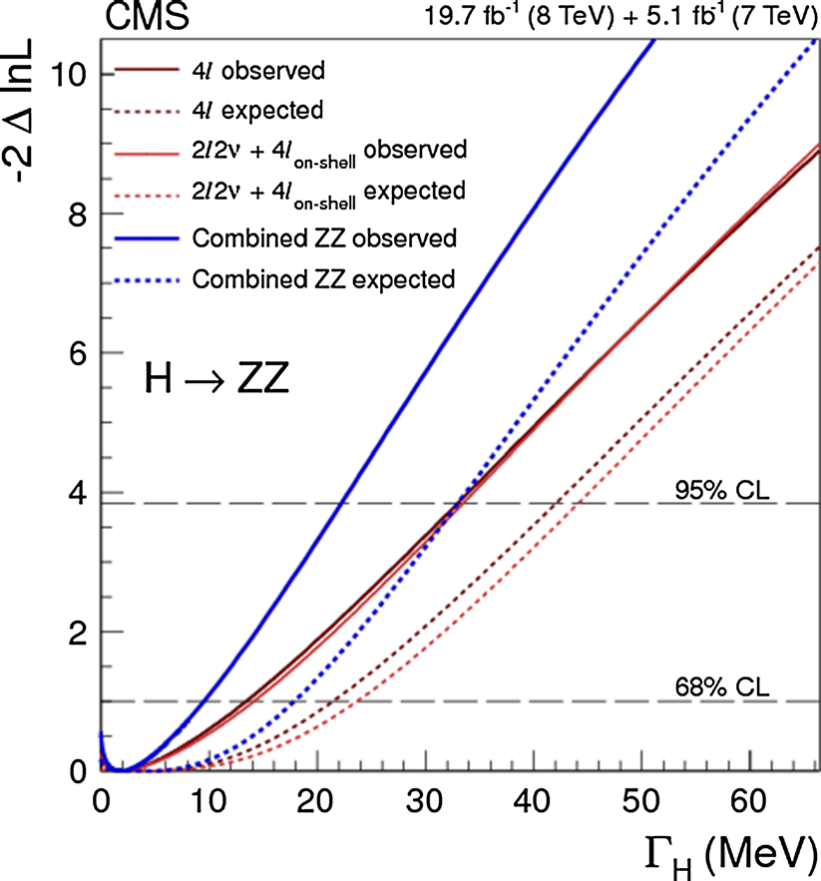
CMS likelihood scan versus ${\varGamma }_H$. Different colours refer to: combination of $4\ell$ low-mass and high-mass (*ochre*), combination of $4\ell$ low-mass and $2\ell 2\nu$ high-mass and combination of $4\ell$ low-mass and both channels at high-mass (*blue*). *Solid* and *dashed lines* represent observed and expected limits, respectively [116]

Figure 27 illustrates the results of a corresponding CMS analysis, yielding observed (expected) 95 % CL limit of ${\varGamma }_H/{\varGamma }^\mathrm{SM}_H<22(33)$ MeV or ${\varGamma }_H/{\varGamma }^\mathrm{SM}_H<5.4(8.0)$.

*Spin and parity* Within the SM, the Higgs boson is a spin-0, ${\textit{CP}}$-even particle. Since the decay kinematics depend on these quantum numbers, the $J^P=0^+$ nature of the SM Higgs boson can be used as constraint to increase the sensitivity of the SM analyses. After dropping such assumptions, however, these analyses can also be used to test against alternative spin–parity hypotheses. These studies are currently based on one or several of the bosonic decays modes discussed above: $H\rightarrow \gamma \gamma$, $H\rightarrow ZZ\rightarrow 4\ell$, and $H\rightarrow WW\rightarrow 2\ell 2\nu$.

**Fig. 28:**
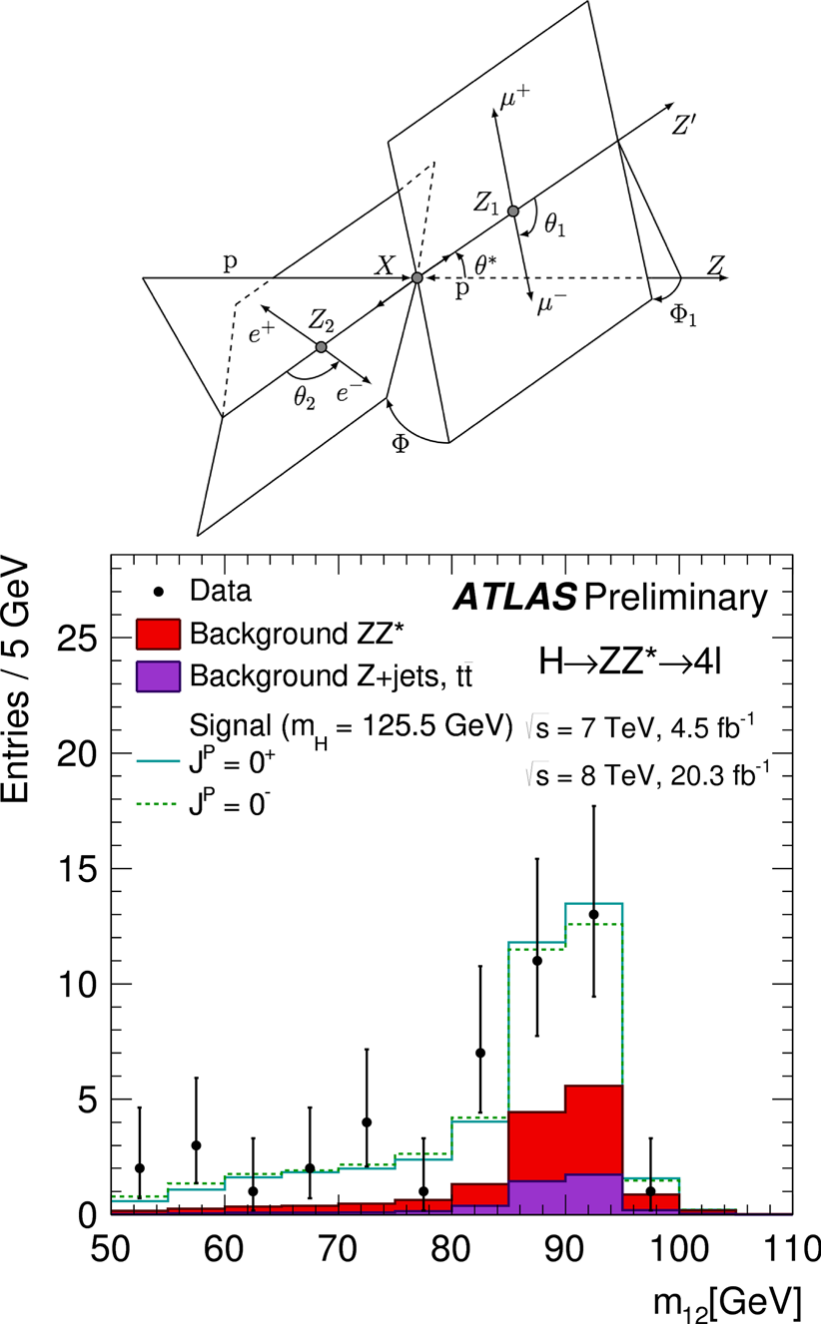
*Top* final-state observables sensitive to the spin and parity of the decaying resonance in $ZZ^*\rightarrow 4\ell$ final states. *Bottom* $\cos \theta _1$ distribution for ATLAS data (point with errors), the backgrounds (*filled histograms*) and several spin hypotheses (SM *solid line* and alternatives *dashed lines*) [119]

In the $H\rightarrow \gamma \gamma$ analysis, the $J^P=0^+$ and $J^P=2^+$ hypothesis can be distinguished via the Collins–Soper angle $\theta ^*$ of the photon system. Since there is a large non-resonant diphoton background, the spin information is extracted from a simultaneous fit to the $|\cos \theta ^*|$ and $m_{\gamma \gamma }$ distributions. The charged-lepton kinematics and the missing transverse energy in $H\rightarrow WW\rightarrow e\nu _e\mu \nu _\mu$ candidate decays are combined in multivariate analyses to compare the data to the SM and three alternative ($J^P=2^+,1^+,1^-$) hypotheses. The $H\rightarrow ZZ\rightarrow 4\ell$ analysis combines a high signal-to-background ratio with a complete final-state reconstruction. This makes it possible to perform a full angular analysis, cf. Fig. 28, albeit currently still with a rather limited number of events. Here, in addition to the spin–parity scenarios discussed above, also the $J^P=0^-$ hypothesis is tested.

**Fig. 29:**
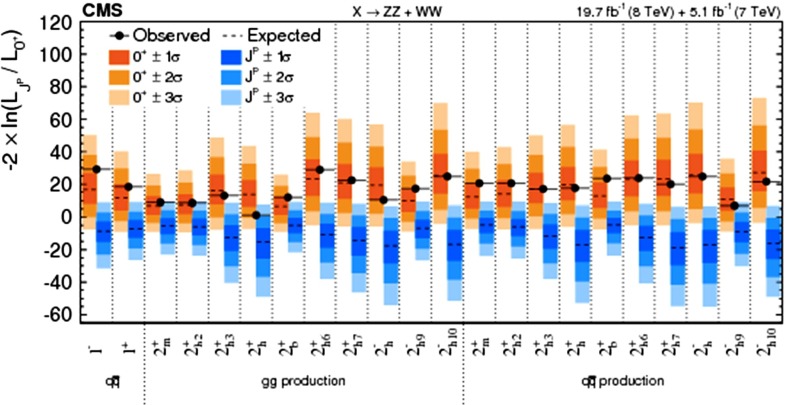
Distributions of the test statistic $q=-2\ln (\mathscr {L}_{J^P}/\mathscr {L}_{0^+})$ for the spin-1 and spin-2 JP models tested against the SM Higgs boson hypothesis in the combined $X\rightarrow ZZ$ and *WW* analyses [117]. The expected median and the 68.3, 95.4, and 99.7 % CL regions for the SM Higgs boson (*orange*, the *left for each model*) and for the alternative $J^P$ hypotheses (*blue*, *right*) are shown. The observed *q* values are indicated by the *black dots*

Including the spin-1 hypotheses in the analyses of the decays into vector bosons provides a test independent of the $H\rightarrow \gamma \gamma$ channel, where $J = 1$ is excluded by the Landau–Yang theorem, and implies the assumptions that the signals observed in the two-photon and *VV* final states are not originating from a single resonance. A representative sample of spin-2 alternatives to SM hypothesis is considered, also including different assumptions concerning the dominant production mechanisms.

For example, Fig. 29 shows the results obtained from CMS analyses of the $H\rightarrow ZZ\rightarrow 4\ell$ and $H\rightarrow WW\rightarrow 2\ell 2\nu$ channels [117]. Agreement with the SM ($J^P=0^+$) within $1.5\sigma$ and inconsistency with alternative hypotheses at a level of at least $3\sigma$ is found. Corresponding ATLAS studies [65, 118, 119] yield similar conclusions.

*Other analyses* In addition to the results discussed above, a number of other analyses have been performed, making use of the increase in the available data since the first Higgs boson discovery in different ways. These include, for example, measurements of differential distributions in $H\rightarrow \gamma \gamma$ [123] and $H\rightarrow ZZ$ [124] events and searches for rarer decays, such as $H\rightarrow \mu \mu$ [125, 126], $H\rightarrow ee$ [126], $H\rightarrow Z\gamma$ [127, 128], decays to heavy quarkonia states and a photon [129], and invisible modes [130, 131]. These searches are not expected to be sensitive to a SM Higgs boson signal based on the currently available data and thus are as of now mainly relevant for the preparation for the larger datasets expected from LHC Run2 and/or for using Higgs boson events as a probe for effects beyond the SM.

Additional production modes are searched for as well. Here, top-associated production is of particular interest because it would provide direct access to the top-Higgs Yukawa coupling. While the results from recent analyses [132–135] of these complex final states do not quite establish a significant signal yet, they demonstrate a lot of promise for LHC Run2, where, in addition to larger datasets, an improved signal-to-background ratio is expected due to the increased collision energy.

#### Future projections

Studies of longer-term Higgs physics prospects currently focus on the scenario of an LHC upgraded during a shutdown starting in 2022 to run at a levelled luminosity of $5\times 10^{34}$ cm$^{-2}$s$^{-1}$, resulting in a typical average of 140 pile-up events per bunch crossing. This so-called HL-LHC is expected to deliver a total integrated luminosity of 3000 fb$^{-1}$ to be compared to a total of 300 fb$^{-1}$ expected by the year 2022.

The following summary of SM Higgs boson analysis prospects for such large datasets is based on preliminary results by the ATLAS and CMS Collaborations documented in [136, 137], respectively. While the prospects for measurements of other Higgs boson properties are being studied as well, the discussion below focusses on projections concerning signal strength measurements and coupling analyses.

*Underlying assumptions* CMS extrapolates the results of current Run1 measurements to $\sqrt{s}=14$ TeV data samples corresponding to 300 fb$^{-1}$ and 3000 fb$^{-1}$ assuming that the upgraded detector and trigger systems will provide the same performance in the high-luminosity environment as the current experiments during 2012, i.e. the signal and background event yields are scaled according to the increased luminosities and cross sections. Results based on two different assumptions concerning the systematic uncertainties are obtained: a first scenario assumes no changes with respect to 2012, while in a second scenario theoretical uncertainties are reduced by a factor of 2 and other uncertainties scaled according to the square root of the integrated luminosities.

**Fig. 30:**
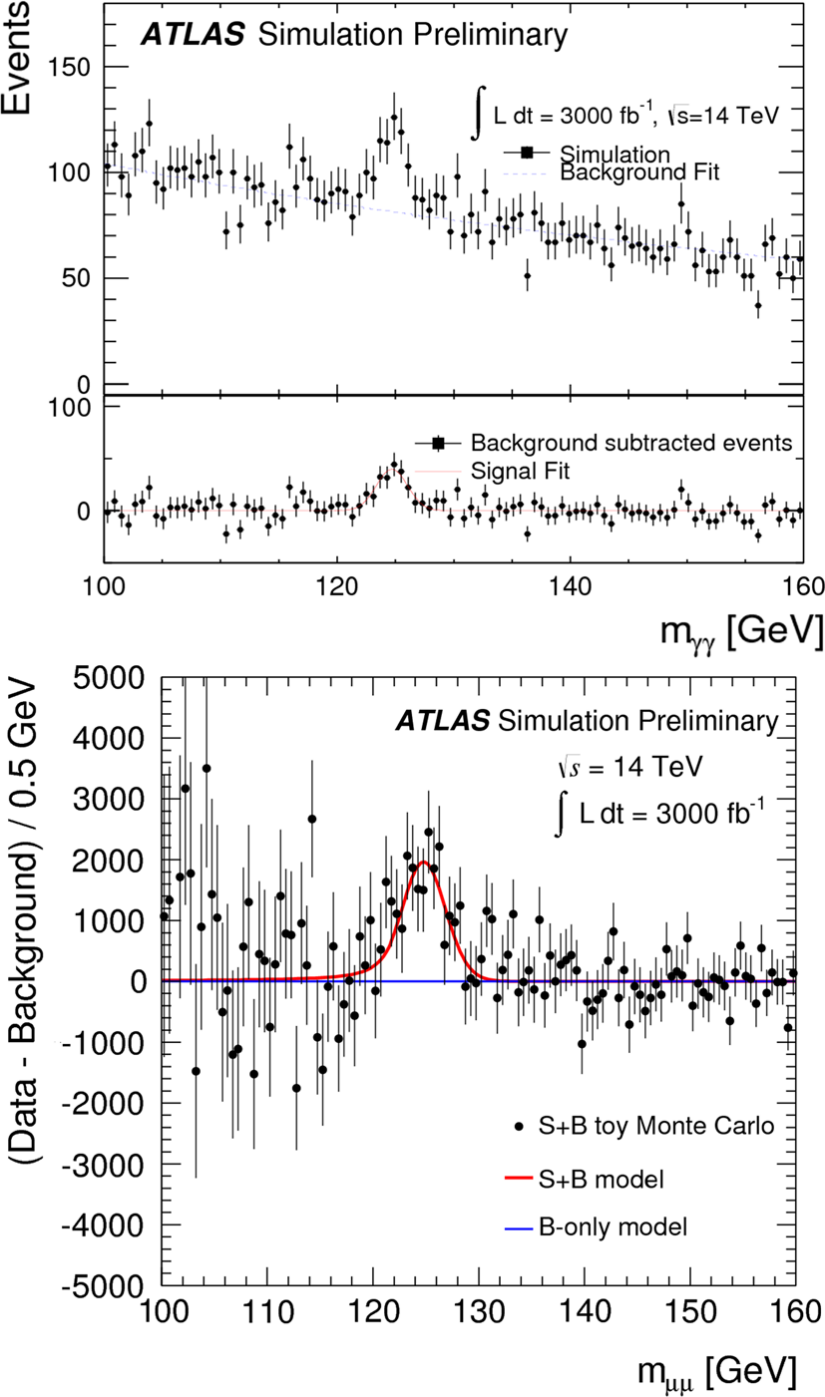
Projected **a** diphoton mass distribution for the SM Higgs boson signal and background processes after VBF selection and **b** background-subtracted dimuon mass distribution based on ATLAS simulations assuming an integrated luminosity of 3000 fb$^{-1}$ [138]

ATLAS investigates the physics prospects for 14 TeV datasets corresponding to the same integrated luminosities as CMS but here the expected detector performance is parameterised based on efficiency and resolution modifications at the detector object level. These are obtained from full simulations corresponding to current and/or upgraded ATLAS detector components assuming values for the number of pile-up events per bunch crossing ranging from 40 to 200. The theoretical uncertainties are assumed to be similar to those used in recent analysis of the Run1 data but some of the experimental systematic uncertainties are re-evaluated taking into account, e.g., the expected improved background estimates due to an increased number of events in data control regions.

*Signal strength* Both experiments study expectations for the experimentally most significant SM Higgs-boson decay modes $H\rightarrow \gamma \gamma$, $H\rightarrow ZZ\rightarrow 4\ell$, $H\rightarrow WW\rightarrow 2\ell 2\nu$, $H\rightarrow \tau \tau$, and $H\rightarrow bb$ but also include analyses of additional sub-modes as well as rare decays to $Z\gamma$, $\mu \mu$, and invisible final states. Figure 30 shows two examples for expected mass signals based on ATLAS simulations of SM Higgs-boson decays to two photons (after a VBF selection) and two muons, respectively.

**Table 4:** Relative uncertainty on the determination of the signal strength expected for the CMS experiment for integrated luminosities of 300 fb$^{-1}$ and 3000 fb$^{-1}$ [137] and the two uncertainty scenarios described in the text

$\mathscr {L}$	300 fb$^{-1}$		3000 fb$^{-1}$	
Scenario	2 (%)	1 (%)	2 (%)	1 (%)
$\gamma \gamma$	6	12	4	8
*WW*	6	11	4	7
*ZZ*	7	11	4	7
*bb*	11	14	5	7
$\tau \tau$	8	14	5	8
$Z\gamma$	62	62	20	24
$\mu \mu$	40	42	14	20

**Fig. 31:**
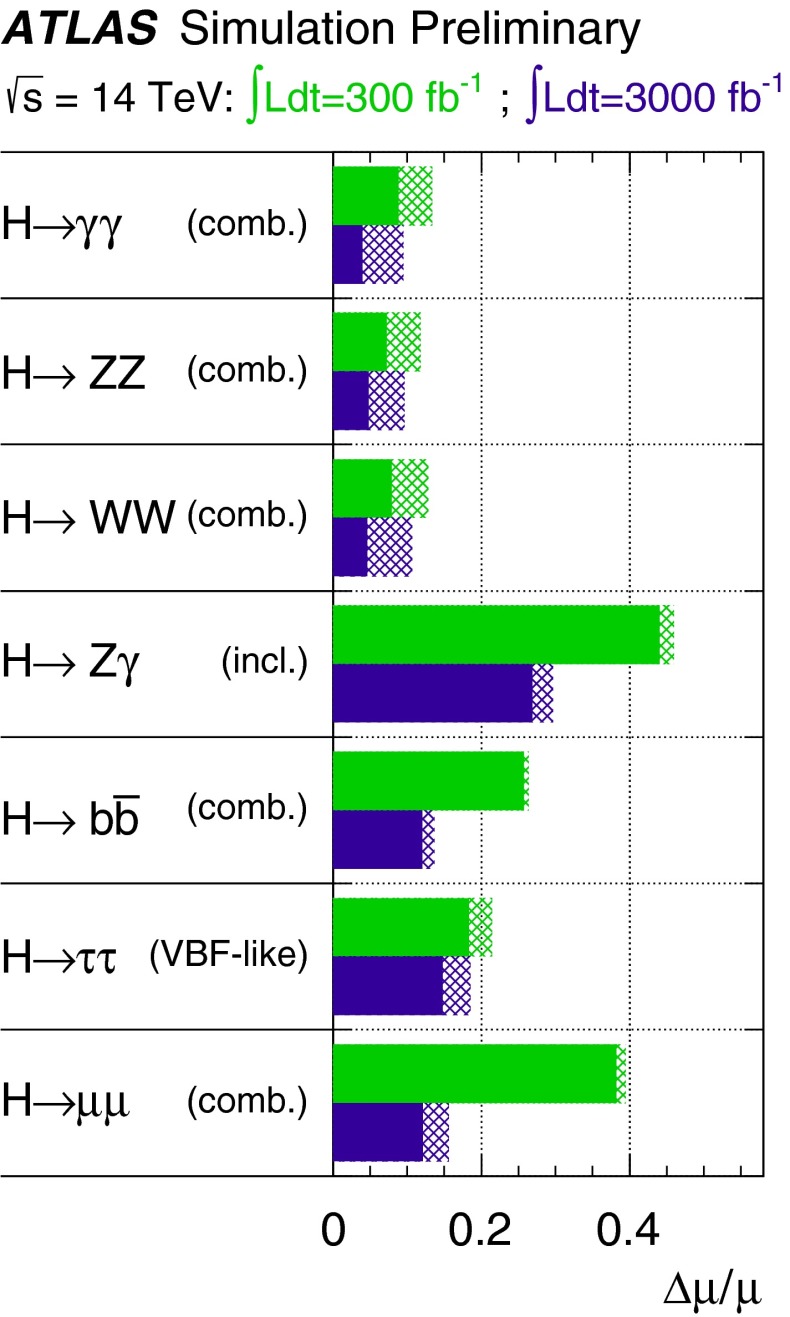
Relative uncertainty on the signal strength determination expected for the ATLAS experiment [136]. Assuming a SM Higgs boson with a mass of 125 GeV and 300 fb$^{-1}$ and 3000 fb$^{-1}$ of 14 TeV data. The uncertainty pertains to the number of events passing the experimental selection, not to the particular Higgs boson process targeted. The hashed areas indicate the increase of the estimated error due to current theory systematic uncertainties

The expected relative uncertainties on the signal strength for CMS and ATLAS are shown in Table 4 and Fig. 31, indicating that for the most sensitive channels, experimental uncertainty around 5 % should be reachable with 3000 fb$^{-1}$. Combining different final states and again assuming SM branching ratios, projections on the sensitivity to individual Higgs-boson production can be obtained; the corresponding ATLAS results are summarised in Table 5. For 3000 fb$^{-1}$, the expected experimental uncertainties on the signal strength range from about 4 % for the dominant *ggF* production to about 10 % for the rare $t\bar{t}H$ production mode. Figure 31 and Table 5 also indicate the contribution of current theoretical uncertainties, showing that reducing them further will be important to fully exploit the HL-LHC for Higgs boson precision studies.

**Table 5:** Relative uncertainty on the signal strength projected by ATLAS for different production modes using the combination of Higgs final states based on integrated luminosities of 300 fb$^{-1}$ and 3000 fb$^{-1}$ [136], assuming a SM Higgs boson with a mass of 125 GeV and branching ratios as in the SM

$\mathscr {L}$	300 fb$^{-1}$		3000 fb$^{-1}$	
Uncertainties	All (%)	No theory (%)	All (%)	No theory (%)
$gg\rightarrow H$	12	6	11	4
VBF	18	15	15	9
*WH*	41	41	18	18
*qqZH*	80	79	28	27
*ggZH*	371	362	147	138
*ttH*	32	30	16	10

**Fig. 32:**
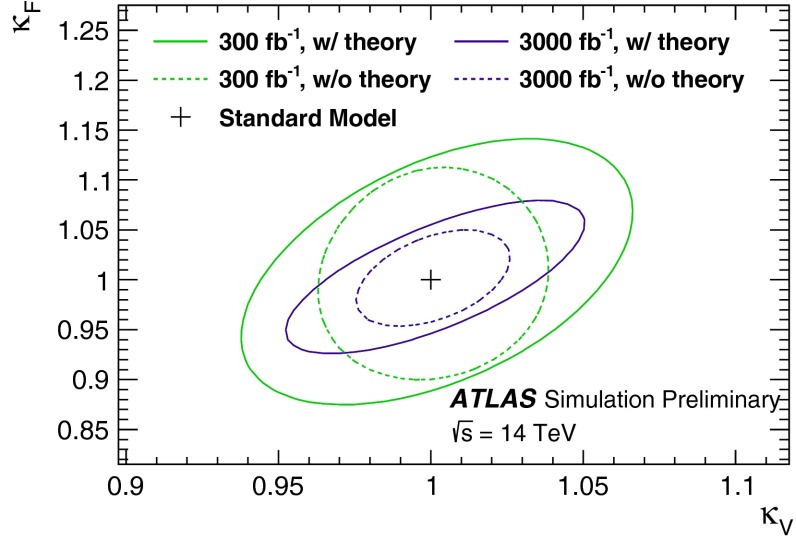
Expected ATLAS 68 and 95 % CL likelihood contours for $\kappa _V$ and $\kappa _F$ in a minimal coupling fit for an integrated luminosity of 300 fb$^{-1}$ and 3000 fb$^{-1}$ [136]

**Fig. 33:**
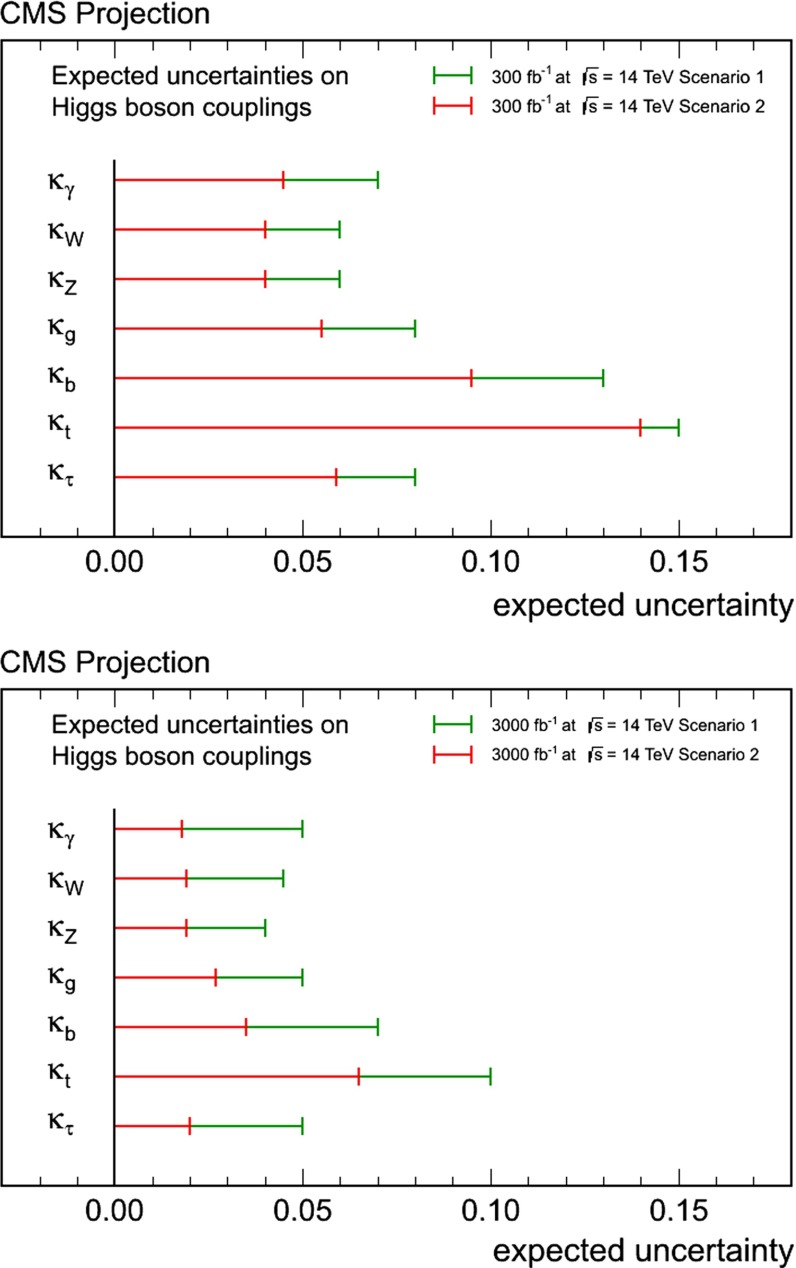
CMS projected relative uncertainty on the measurements of $\kappa _\gamma$, $\kappa _V$, $\kappa _g$, $\kappa _b$, $\kappa _t$, and $\kappa _\tau$ assuming $\sqrt{s} = 14$ TeV and an integrated luminosity 300 and 3000 fb$^{-1}$. The results are shown for two uncertainty scenarios described in the text [137]

*Couplings to other particles* The individual channels are combined to obtain projections on the experimental sensitivity concerning Higgs-boson couplings to other elementary bosons and fermions. Following the same formalism and set of assumptions used for the current Run1 results described above, coupling scale factors $\kappa _X$ are extracted. Figure 32, for example, shows the projected ATLAS results of the minimal coupling fit constrained to common scale factors $\kappa _F$ and $\kappa _V$ for all fermions and bosons, respectively, and assuming SM values for both; cf. Fig. 20 for the corresponding Run1 results. Figure 33 gives an overview of the precision on the extraction of individual coupling scale factors expected for the CMS experiment.

**Table 6:** Relative uncertainty on the determination of the coupling scale factor ratios expected for the CMS experiment for integrated luminosities of 300 fb$^{-1}$ and 3000 fb$^{-1}$ [137] and the two uncertainty scenarios described in the text

$\mathscr {L}$	300 fb$^{-1}$		3000 fb$^{-1}$	
Scenario	2 (%)	1 (%)	2 (%)	1 (%)
$\kappa _\gamma \cdot \kappa _Z/\kappa _H$	4	6	2	5
$\kappa _W/\kappa _Z$	4	7	2	3
$\lambda _{tg}=\kappa _t/\kappa _g$	13	14	6	8
$\lambda _{bZ}=\kappa _b/\kappa _Z$	8	11	3	5
$\lambda _{\tau Z}=\kappa _\tau /\kappa _Z$	6	9	2	4
$\lambda _{\mu Z}=\kappa _\mu /\kappa _Z$	22	23	7	8
$\lambda _{Zg}=\kappa _Z/\kappa _g$	6	9	3	5
$\lambda _{\gamma Z}=\kappa _\gamma /\kappa _Z$	5	8	2	5
$\lambda _{(Z\gamma )Z }=\kappa _{Z\gamma }/\kappa _Z$	40	42	12	12

**Fig. 34:**
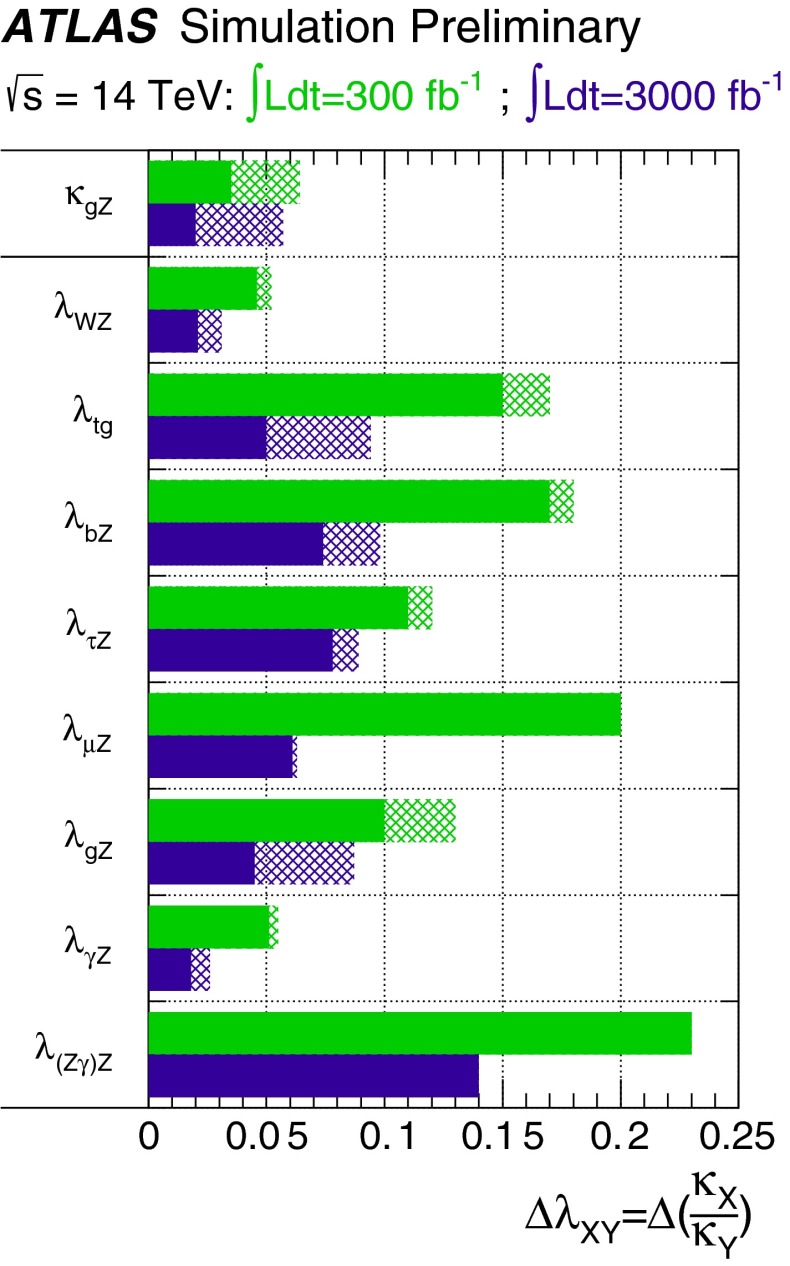
Relative uncertainty expected for the ATLAS experiment on the determination of coupling scale factor ratios $\lambda _{XY}=\kappa _X/\kappa _Y$ from a generic fit [136], assuming a SM Higgs boson with a mass of 125 GeV and 300 fb$^{-1}$ and 3000 fb$^{-1}$ of 14 TeV data. The *hashed* areas indicate the increase of the estimated error due to current theory uncertainties

The $\kappa _X$ extraction requires assumptions on the total width of the Higgs boson. Without total width information, only ratios of couplings can be studied. As for the current Run1 analyses, results are obtained for several different sets of assumptions. An overview of the expected CMS precision for the most generic of these scenarios, still with a single, narrow, ${\textit{CP}}$-even scalar Higgs boson but without further assumptions, e.g. on new-particle contributions through loops, is given in Table 6. Results from corresponding ATLAS analyses are shown in Fig. 34, where, for an integrated luminosity of 3000 fb$^{-1}$, the experimental uncertainties range from about 2 % for the coupling scale factors between the electroweak bosons to 5–8 % for the ratios involving gluons and fermions outside the first generation.

**Fig. 35:**
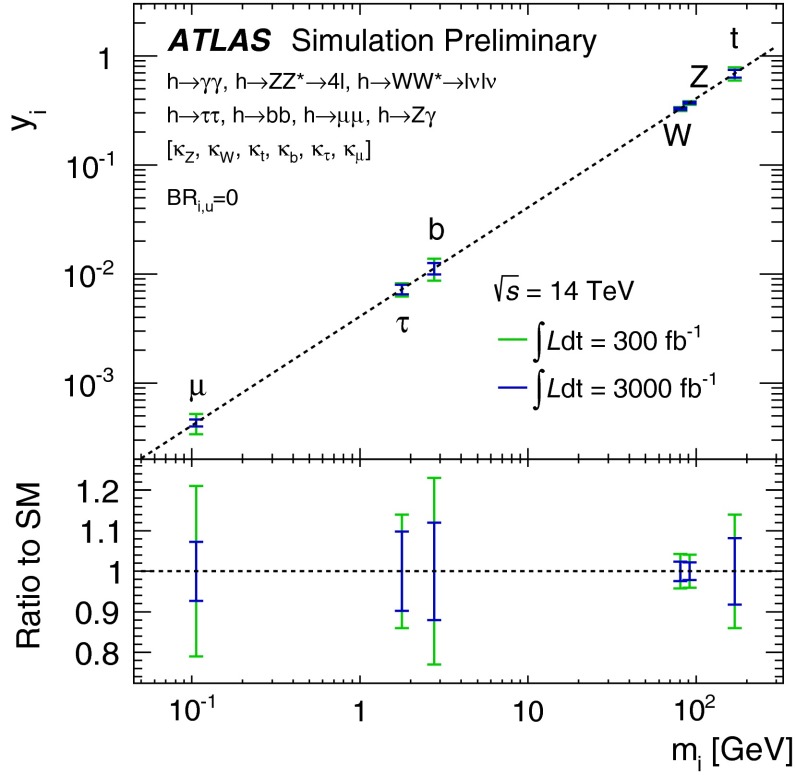
Fit results for the reduced coupling scale factors for weak bosons and fermions as a function of the particle mass, assuming 300/fb or 3000/fb of 14 TeV data and a SM Higgs boson with a mass of 125 GeV [136]

Figure 35 gives the ATLAS projection for the precision of the Higgs-boson couplings to other elementary SM particles as a function of the particle masses obtained from fits assuming no BSM contributions to Higgs-boson decays or particle loops; see Fig. 24 for corresponding CMS Run1 results.

**Fig. 36:**
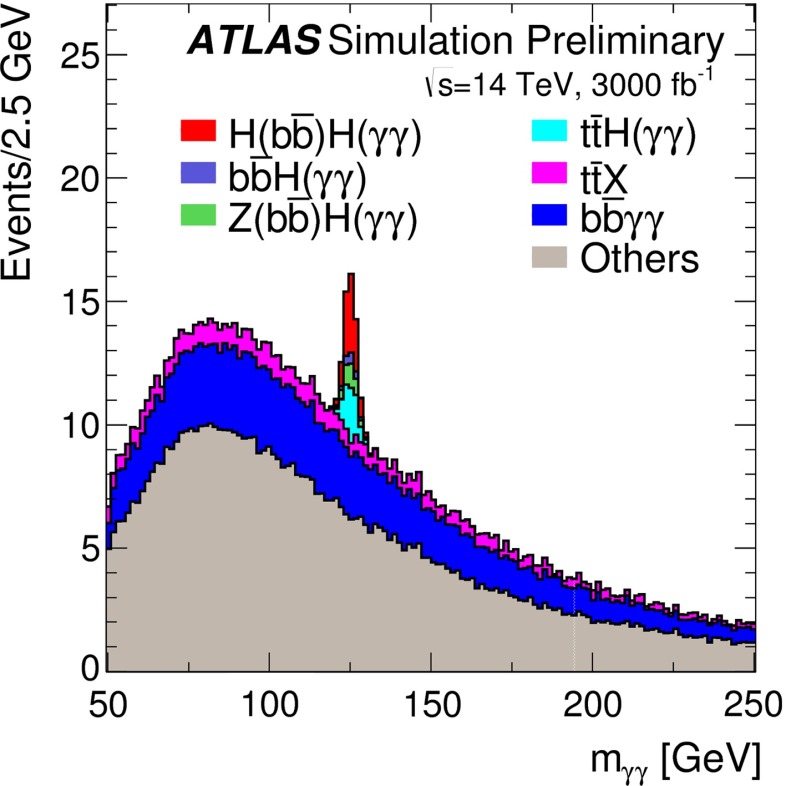
Projected diphoton mass distribution for signal and background processes based on ATLAS simulations for a search for Higgs boson pair production with subsequent decays $H\rightarrow b\bar{b}$ and $H\rightarrow \gamma \gamma$ assuming an integrated luminosity of 3000 fb$^{-1}$ [139]. The simulated distributions are scaled to match the expected event yields but do not necessarily reflect the corresponding statistical fluctuations

**Fig. 37:**
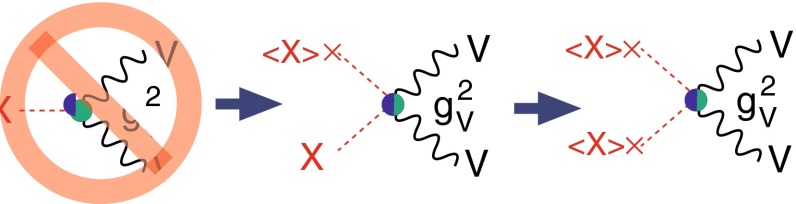
The origin of *XVV* coupling and its relation to the mass term of *V*

*Higgs self-coupling* One of the most important long-term goals of the SM Higgs physics programme is the measurement of the tri-linear self-coupling $\lambda _{HHH}$, which requires the study of Higgs boson pair production. At the LHC the dominant production mechanism is gluon–gluon fusion with a cross section of about 40 fb at $\sqrt{s}=14$ TeV. Several combinations of Higgs decays can be considered. For example, assuming 3000 fb$^{-1}$ of 14 TeV data [139] presents the ATLAS prospects for the search for Higgs pair production in the channel $H(\rightarrow \gamma \gamma )H(\rightarrow bb)$, which combines the large $H\rightarrow bb$ branching ratio with the good mass resolution of the two-photon final state. The projected diphoton mass distribution for simulated *ggF*-produced signal and background processes after signal selection requirements is shown in Fig. 36; the statistical analysis gives a signal yield of about eight events and signal significance of 1.3$\sigma$. Although additional observables, the application of more sophisticated analysis techniques and the inclusion of other production modes can be expected to improve on this result, a combination with other decay channels will likely be needed to find evidence for SM Higgs pair production (or to exclude that the Higgs self-coupling strength is close to its SM expectation) with an integrated luminosity of 3000 fb$^{-1}$.

### Higgs at ILC: prospects^9^

#### Introduction

The success of the SM is a success of the gauge principle. It is the success of the transverse components of *W* and *Z* identified as gauge fields of the electroweak (EW) gauge symmetry. Since explicit mass terms for *W* and *Z* are forbidden by the gauge symmetry, it must be spontaneously broken by *something condensed in the vacuum* which carries EW charges ($I_3$ and *Y* denoting the third component of the weak isospin and the hypercharge, respectively),

12 $$\begin{aligned} \left\langle {0} \,\right| I_3, Y \left| \, {0} \, \right\rangle \ne 0 \text{ while } \left\langle {0} \,\right| I_3 + Y \left| \, {0} \, \right\rangle = 0. \end{aligned}$$

We are hence living in a weak-charged vacuum. This *something* provides three longitudinal modes of *W* and *Z*:

13 $$\begin{aligned} \text{ Goldstone } \text{ modes: } \chi ^+, \chi ^-, \chi _3 \rightarrow W_L^+, W_L^-, Z_L. \end{aligned}$$

It should be emphasised that we do not know the nature of these longitudinal modes which stem from the *something*. The gauge symmetry also forbids explicit mass terms for matter fermions, since left- ($f_L$) and right-handed ($f_R$) matter fermions carry different EW charges; hence, as long as the EW charges are conserved, they cannot mix. Their Yukawa interactions with some weak-charged vacuum can compensate the EW-charge difference and hence allow the $f_L$–$f_R$ mixing. In the SM, the same *something* is responsible for the $f_L$–$f_R$ mixing, thereby generating masses and inducing flavour mixings among generations. To form gauge-invariant Yukawa interaction terms, we need a complex doublet scalar field, which has four real components. In the SM, three of them are identified with the three Goldstone modes and are used to supply the longitudinal modes of *W* and *Z*. The remaining one is the physical Higgs boson. There is no reason for this simplicity of the symmetry breaking sector of the SM. The symmetry breaking sector (hereafter called the Higgs sector) can well be much more complicated. The *something* could be composite instead of being elementary. We know it is there around us with a vacuum expectation value of 246 GeV. But this was about all we knew concerning the *something* until July 4, 2012.

**Fig. 38:**
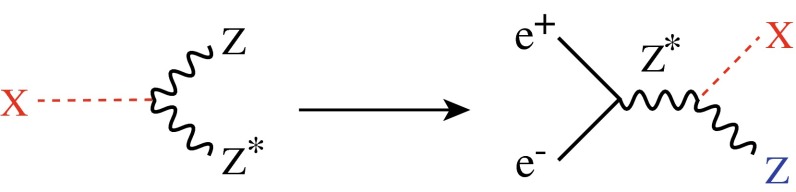
$X \rightarrow ZZ^*$ decay and $e^+e^- \rightarrow ZX$ process

Since the July 4th, the world has changed! The discovery of the 125 GeV boson (*X*(125)) at the LHC could be called a quantum jump [142, 143]. The observation of $X(125) \rightarrow \gamma \gamma$ decay implies *X* is a neutral boson having a spin not equal to 1 (Landau–Yang theorem). We know that the 125 GeV boson decays also to $ZZ^*$ and $WW^*$, indicating the existence of *XVV* couplings, where $V=W/Z$, gauge bosons. There is, however, no gauge coupling like *XVV*, see Fig. 37. There are only *XXVV* and *XXV*. The *XVV* coupling is hence most probably from *XXVV* with one *X* replaced by its vacuum expectation value $\langle X \rangle \ne 0$, namely $\langle X\rangle XVV$. Then there must be $\langle X\rangle \langle X\rangle VV$, a mass term for *V*, meaning that *X* is at least part of the origin of the masses of $V=W/Z$. This is a great step forward to uncover the nature of the *something* in the vacuum but we need to know whether $\langle X\rangle$ saturates the SM VEV of 245 GeV. The observation of the $X \rightarrow ZZ^*$ decay means that *X* can be produced via $e^+e^- \rightarrow Z^* \rightarrow ZX$, since by attaching an $e^+e^-$ pair to the $Z^*$ leg and rotate the whole diagram we can get the *X*-strahlung diagram as shown in Fig. 38. By the same token, $X \rightarrow WW^*$ means that *X* can be produced via the *WW*-fusion process: $e^+e^- \rightarrow \nu \bar{\nu }X$. So we now know that the major Higgs production processes in $e^+e^-$ collisions are indeed available at the ILC, which can be regarded as a *no lose theorem* for the ILC. The 125 GeV is the best place for the ILC, where variety of decay modes are accessible. We need to check the 125 GeV boson in detail to see if it has indeed all the required properties of the *something* in the vacuum.

**Fig. 39:**
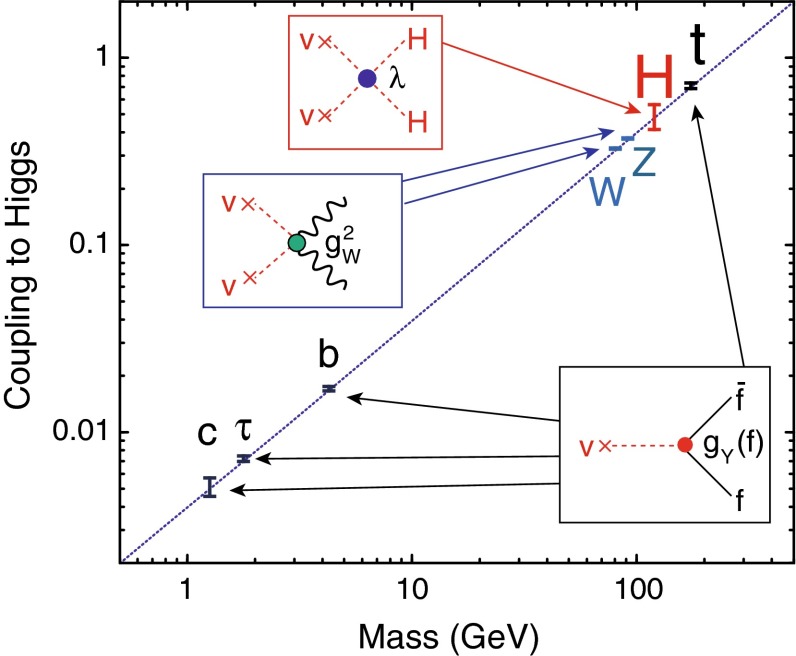
Mass–coupling relation [144]

The properties to measure are the mass, width, and $J^{PC}$, its gauge, Yukawa, and self-couplings. The key is to confirm *the mass–coupling relation*. If the 125 GeV boson is the one to give masses to all the SM particles, coupling should be proportional to mass as shown in Fig. 39. Any deviation from the straight line signals physics beyond the standard model (BSM). The Higgs serves therefore as a window to BSM physics.

Our mission is the bottom-up model-independent reconstruction of the EWSB sector through the coupling measurements. We need to determine the multiplet structure of the Higgs sector by answering questions like: Is there an additional singlet or doublet or triplet? What about the underlying dynamics? Is it weakly interacting or strongly interacting? In other words, is the Higgs boson elementary or composite? We should also try to investigate its possible relation to other questions of particle physics such as DM, electroweak baryogenesis, neutrino masses, and inflation.

There are many possibilities and different models predict different deviation patterns in the mass–coupling relation. An example is given in Table 7, where a model with an extra singlet and four types of two-Higgs-doublet models (2HDM) are compared. The four types of 2HDMs differ in the assignment of a $Z_2$ charge to the matter fermions, which protects them from inducing dangerous flavour-changing neutral currents [145, 146].

**Table 7:** The expected deviation pattern for various Higgs couplings, assuming small deviations for $\cos (\beta -\alpha ) < 0$. The arrows for Yukawa interactions are reversed for 2HDMs with $\cos (\beta -\alpha ) > 0$

Model	$\mu$	$\tau$	*b*	*c*	*t*	$g_V$
Singlet mixing	$\downarrow$	$\downarrow$	$\downarrow$	$\downarrow$	$\downarrow$	$\downarrow$
2HDM-I	$\downarrow$	$\downarrow$	$\downarrow$	$\downarrow$	$\downarrow$	$\downarrow$
2HDM-II (SUSY)	$\uparrow$	$\uparrow$	$\uparrow$	$\downarrow$	$\downarrow$	$\downarrow$
2HDM-X (Lepton-specific)	$\uparrow$	$\uparrow$	$\downarrow$	$\downarrow$	$\downarrow$	$\downarrow$
2HDM-Y (Flipped)	$\downarrow$	$\downarrow$	$\uparrow$	$\downarrow$	$\downarrow$	$\downarrow$

Notice that though both singlet mixing and 2HDM-I with $\cos (\beta -\alpha )<0$ give downward deviations, they are quantitatively different: the singlet mixing reduces the coupling constants universally, while 2HDM-I reduces them differently for matter fermions and gauge bosons. In these models, $g_V < 1$ is guaranteed because of the sum rule for the vacuum expectation values of the SM-like Higgs boson and the additional doublet or singlet. When a doubly charge Higgs boson is present, however, $g_V >1$ is possible. The size of any of these deviations is generally written in the following form due to the decoupling theorem:

14 $$\begin{aligned} \frac{{\varDelta } g}{g} = \mathscr {O} \left( \frac{v^2}{M^2} \right) \end{aligned}$$

where *v* is the SM VEV and *M* is the mass scale for the new physics. Since there is no hint of new physics beyond the SM seen at the LHC, *M* should be rather large implying small deviations. In order to detect possible deviations and to fingerprint the BSM physics from the deviation pattern, we hence need a % level precision, which in turn requires a 500 GeV linear collider such as the ILC and high precision detectors that match the potential of the collider.

**Fig. 40:**
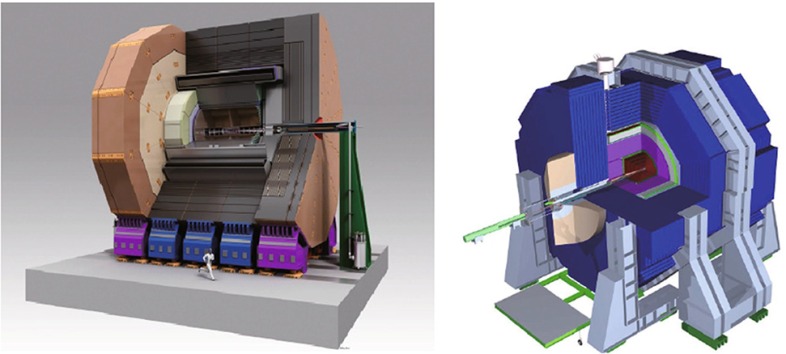
Two proposed detector concepts for the ILC: ILD (*left*) and SiD (*right*) [147]

The ILC, being an $e^+e^-$ collider, inherits all of its traditional merits: cleanliness, democracy, detail, and calculability. The two detector concepts proposed for the ILC: ILD and SiD (see Fig. 40) take advantage of these merits.

Moreover, they are designed with an ambitious goal of reconstructing all the events in terms of fundamental particles such as quarks, leptons, gauge bosons, and Higgs bosons, thereby viewing events as viewing Feynman diagrams. This requires a thin and high resolution vertex detector that enables identification of *b*- and *c*-quarks by detecting secondary and tertiary vertices, combination of a high resolution charged particle tracker and high granularity calorimeters optimised for particle flow analysis (PFA) to allow identification of *W*, *Z*, *t*, and *H* by measuring their jet invariant masses, and hermeticity down to $\mathscr {O} ( 10 \mathrm{mrad})$ or better for indirect detection of a neutrino as missing momentum. Notice that both ILD and SiD put all the calorimeters inside the detector solenoidal magnets to satisfy the requirement of hermeticity and high performance PFA. Furthermore, the power of beam polarisations should be emphasised. Consider the $e^+e^- \rightarrow W^+W^-$ process. At the energies explored by the ILC, $SU(2)_L \otimes U(1)_Y$ symmetry is approximately recovered and hence the process can be regarded as taking place through two diagrams: *s*-channel $W_3$ exchange and *t*-channel $\nu _e$ exchange. Since both $W_3$ and $\nu _e$ couple only to a left-handed electron (and right-handed positrons), right-handed electrons will not contribute to the process. This is also the case for one of the most important Higgs production process at the ILC: $e^+e^- \rightarrow \nu _e \bar{\nu }_e H$ (*WW*-fusion single Higgs production). If we have an 80 % left-handed electron beam and a 30 % right-handed positron beam the Higgs production cross section for this *WW*-fusion process will be enhanced by a factor of 2.34 as compared to the unpolarised case. Beam polarisation hence plays an essential role.

**Fig. 41:**
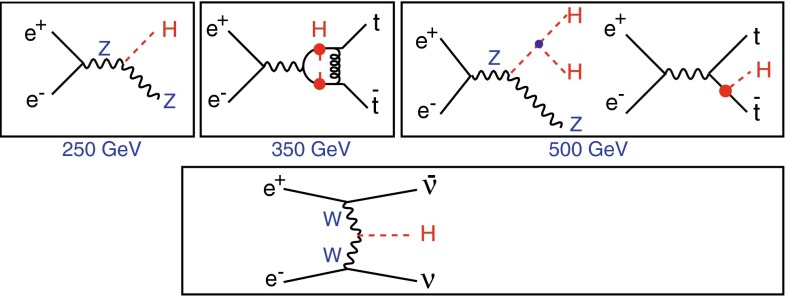
Why 250–500 GeV? The three thresholds

*Why 250–500 GeV?* The ILC is an $e^+e^-$ collider designed primarily to cover the energy range from $\sqrt{s}=250$ to 500 GeV. This is because of the following three very well-known thresholds (Fig. 41). The first threshold is at around $\sqrt{s}=250$ GeV, where the $e^+e^- \rightarrow Zh$ process will reach its cross section maximum. This process is a powerful tool to measure the Higgs mass, width, and $J^{PC}$. As we will see below, this process allows us to measure the *hZZ* coupling in a completely model-independent manner through the recoil mass measurement. This is a key to perform model-independent extraction of branching ratios for various decay modes such as $h \rightarrow b\bar{b}$, $c\bar{c}$, $\tau \bar{\tau }$, *gg*, $WW^*$, $ZZ^*$, $\gamma \gamma$, as well as invisible decays.

The second threshold is at around $\sqrt{s}=350$ GeV, which is the well-known $t\bar{t}$ threshold. The threshold scan here provides a theoretically very clean measurement of the top-quark mass, which can be translated into $m_t(\overline{{\mathrm{MS}}})$ to an accuracy of 100 MeV. The precise value of the top mass obtained this way can be combined with the precision Higgs mass measurement to test the stability of the SM vacuum [148, 149]. The $t\bar{t}$ threshold also enables us to indirectly access the top Yukawa coupling through the Higgs exchange diagram. It is also worth noting that with the $\gamma \gamma$ collider option at this energy the double Higgs production: $\gamma \gamma \rightarrow hh$ is possible, which can be used to study the Higgs self-coupling [150]. Notice also that at $\sqrt{s}=350\,$GeV and above, the *WW*-fusion Higgs production process, $e^+e^- \rightarrow \nu \bar{\nu }h$, becomes sizeable with which we can measure the *hWW* coupling and accurately determine the total width.

The third threshold is at around $\sqrt{s}=500$ GeV, where the double Higgs-strahlung process, $e^+e^- \rightarrow Zhh$ attains its cross section maximum, which can be used to access the Higgs self-coupling. At $\sqrt{s}=500$ GeV, another important process, $e^+e^- \rightarrow t\bar{t}h$, will also open, though the product cross section is much smaller than its maximum that is reached at around $\sqrt{s}=800$ GeV. Nevertheless, as we will see, QCD threshold correction enhances the cross section and allows us a reasonable measurement of the top Yukawa coupling concurrently with the self-coupling measurement.

By covering $\sqrt{s}=250$–500 GeV, we will hence be able complete the mass–coupling plot. This is why the first phase of the ILC project is designed to cover the energy up to $\sqrt{s}=500$ GeV.

#### ILC at 250 GeV

**Fig. 42:**
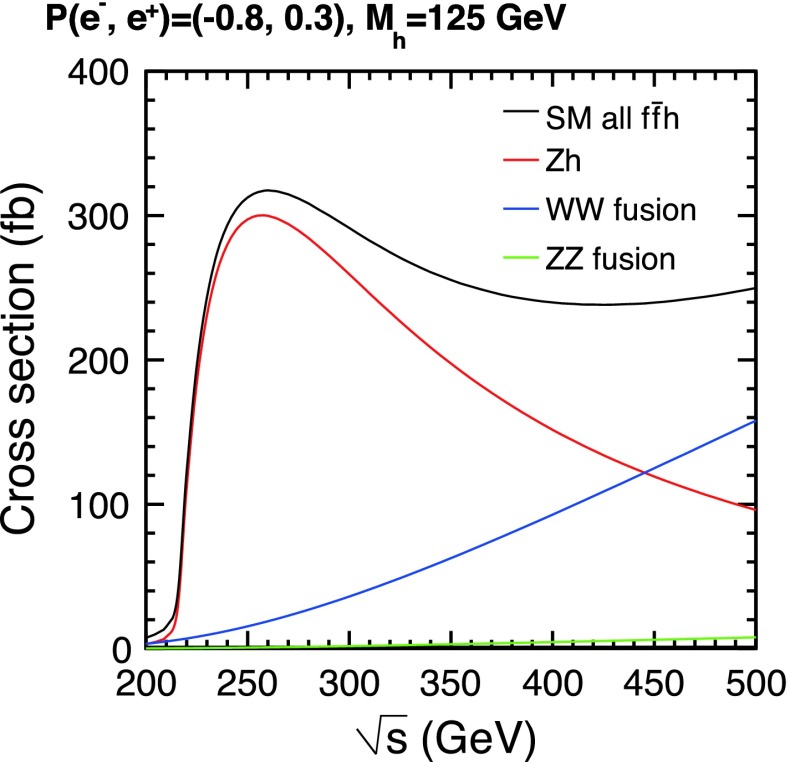
Cross sections for the three major Higgs production processes as a function of centre-of-mass energy

The first threshold is at around $\sqrt{s}=250\,$GeV, where the $e^+e^- \rightarrow Zh$ (Higgs-strahlung) process attains its cross section maximum (see Fig. 42).

**Fig. 43:**
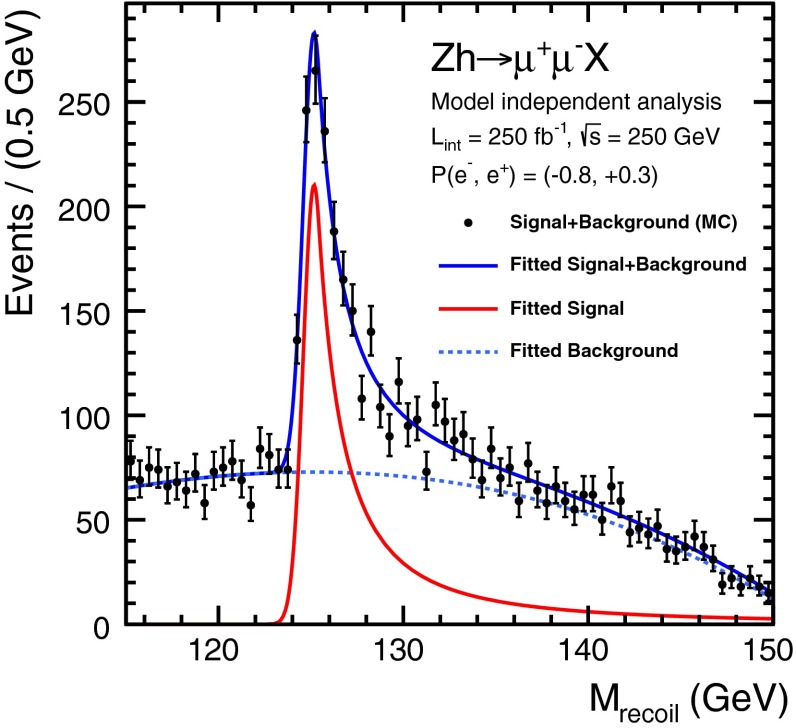
Recoil mass distribution for the process: $e^+e^- \rightarrow Zh$ followed by $Z \rightarrow \mu ^+\mu ^-$ decay for $m_h=125$ GeV with 250 fb$^{-1}$ at $\sqrt{s}=250$ GeV [151]

The most important measurement at this energy is that of the recoil mass for the process: $e^+e^- \rightarrow Zh$ followed by $Z \rightarrow \ell ^+\ell ^- ~(\ell =e,\mu )$ decay. By virtue of the $e^+e^-$ collider, we know the initial-state 4-momentum. We can hence calculate the invariant mass of the system recoiling against the lepton pair from the *Z* decay by just measuring the momenta of the lepton pair:

15 $$\begin{aligned} M_X^2 = \left( p_{CM} - (p_{\ell ^+} + p_{\ell ^-})\right) ^2. \end{aligned}$$

The recoil mass distribution is shown in Fig. 43 for a $m_h=125$ GeV Higgs boson with 250 fb$^{-1}$ at $\sqrt{s}=250$ GeV. A very clean Higgs peak is sticking out from small background. Notice that with this recoil mass technique even invisible decay is detectable since we do not need to look at the Higgs decay at all [152]. This way, we can determine the Higgs mass to ${\varDelta } m_h=30$ MeV and the production cross section to ${\varDelta } \sigma _{Zh} /\sigma _{Zh} = 2.6$ %, and limit the invisible branching ratio to 1 % at the 95 % confidence level [153, 154]. This is the flagship measurement of the ILC at 250 GeV that allows absolute measurement of the *hZZ* coupling thereby unlocking the door to completely model-independent determinations of various couplings of the Higgs boson as well as its total width as we will see below.

**Fig. 44:**
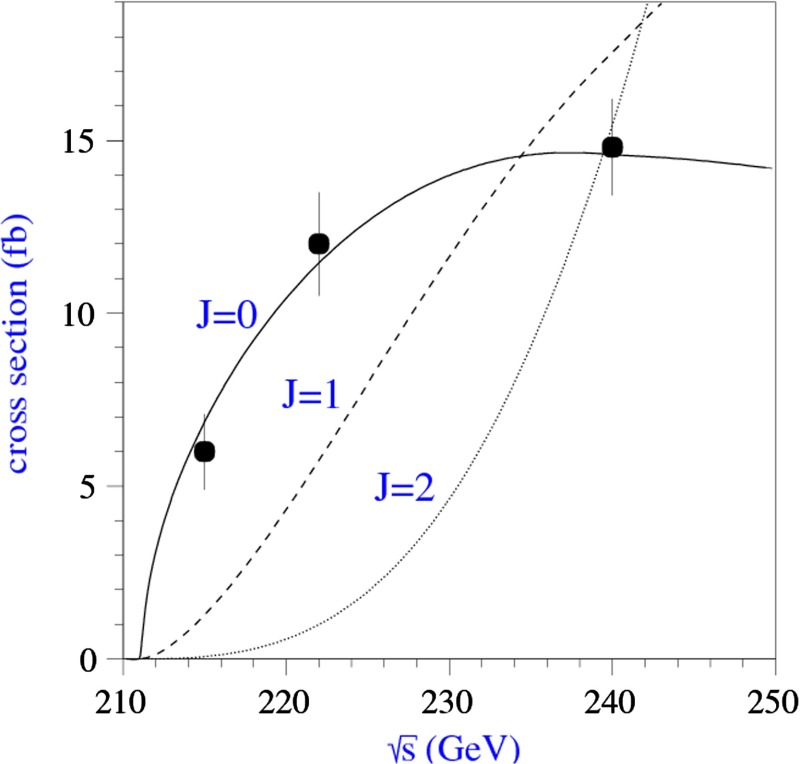
Threshold scan of the $e^+e^- \rightarrow Zh$ process for $m_h = 120$ GeV, compared with theoretical predictions for $J^{P}= 0^{+}$, $1^{-}$, and $2^{+}$ [156]

Before moving on to the coupling determinations, let us discuss here the determination of the spin and ${\textit{CP}}$ properties of the Higgs boson. The LHC observed the $h \rightarrow \gamma \gamma$ decay, which fact alone rules out the possibility of spin 1 and restricts the charge conjugation C to be positive. The more recent LHC analyses strongly prefer the $J^P=0^+$ assignment over $0^-$ or $2^\pm$ [155]. By the time of the ILC the discrete choice between different spin and ${\textit{CP}}$-even or -odd assignments will certainly be settled, assuming that the 125 GeV boson is a ${\textit{CP}}$ eigen state. Nevertheless, it is worth noting that the ILC also offers an additional, orthogonal, and clean test of these assignments. The threshold behaviour of the *Zh* cross section has a characteristic shape for each spin and each possible ${\textit{CP}}$ parity. For spin 0, the cross section rises as $\beta$ near the threshold for a ${\textit{CP}}$-even state and as $\beta ^3$ for a ${\textit{CP}}$-odd state. For spin 2, for the canonical form of the coupling to the energy-momentum tensor, the rise is also $\beta ^3$. If the spin is higher than 2, the cross section will grow as a higher power of $\beta$. With a three-20 fb$^{-1}$-point threshold scan of the $e^+e^- \rightarrow Zh$ production cross section we can separate these possibilities [156] as shown in Fig. 44. The discrimination of more general forms of the coupling is possible by the use of angular correlations in the boson decay; this is discussed in detail in [157].

**Fig. 45:**
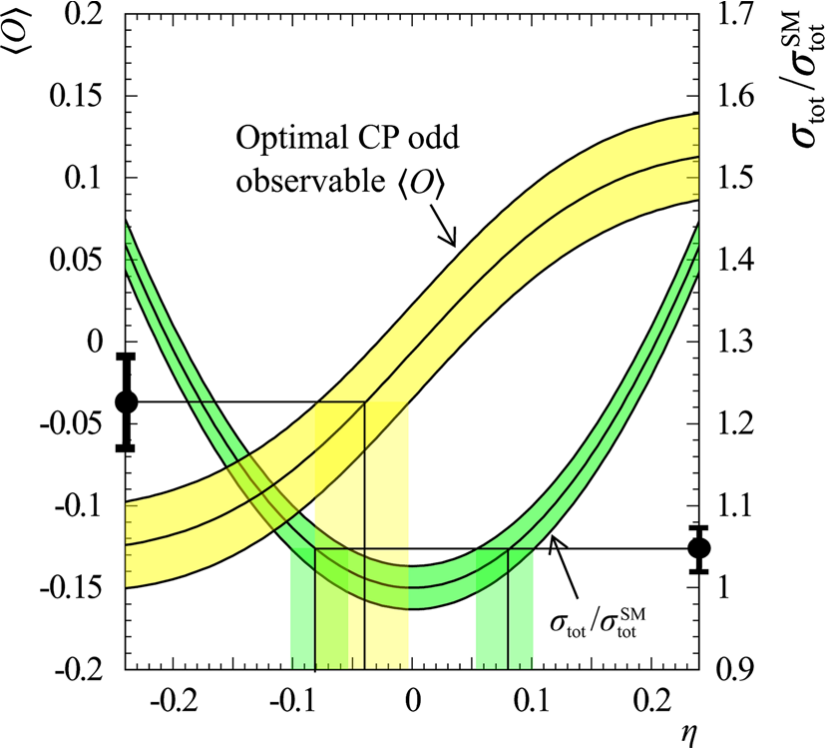
Determination of ${\textit{CP}}$ mixing with $1\sigma$ bands expected at $\sqrt{s}=350$ GeV and 500 fb$^{-1}$ [158]

The power of the ILC manifests itself when we ask more subtle questions. There is no guarantee that the *h* is a ${\textit{CP}}$ eigenstate. It can rather be a mixture of ${\textit{CP}}$-even and ${\textit{CP}}$-odd components. This happens if ${\textit{CP}}$ is violated in the Higgs sector. A small ${\textit{CP}}$-odd contribution to the *hZZ* coupling can affect the threshold behaviour. Figure 45 shows the determination of the small ${\textit{CP}}$-odd component $\eta$ at $\sqrt{s}=350$ GeV from the value of the total cross section and from an appropriately defined optimal observable [158]. The *hZZ* coupling is probably not the best tool to study possible ${\textit{CP}}$ admixture, since in many scenarios the ${\textit{CP}}$-odd *hZZ* coupling is only generated through loops. It is, hence, more effective to use a coupling for which the ${\textit{CP}}$-even and ${\textit{CP}}$-odd components are on the same footing as in the *h* coupling to $\tau ^+\tau ^-$, given by

16 $$\begin{aligned} {\varDelta } {\mathscr {L}} = - {m_\tau \over v} h\ \bar{\tau }(\cos \alpha + i \sin \alpha \gamma ^5) \tau \end{aligned}$$

for a Higgs boson with a ${\textit{CP}}$-odd component. The polarisations of the final-state $\tau$s can be determined from the kinematic distributions of their decay products; the ${\textit{CP}}$-even and -odd components interfere in these distributions [159, 160]. In [161], it is estimated that the angle $\alpha$ can be determined at the ILC to an accuracy of 6$^\circ$.

The $e^+e^- \rightarrow Zh$ process can also be used to measure various branching ratios for various Higgs decay modes. For this purpose $Z \rightarrow q\bar{q}$ and $\nu \bar{\nu }$ decays can be included in our analysis to enhance the statistical precision. We should stress here that as with similar Higgs-related measurements at the LHC what we can actually measure is *not* the branching ratio ($\mathrm{BR}$) itself but the cross section times branching ratio ($\sigma \times \mathrm{BR}$). The crucial difference is the recoil mass measurement at the ILC, which provides $\sigma$ enabling one to extract $\mathrm{BR}$ from $\sigma \times \mathrm{BR}$ model independently. Table 8 summarises the expected precisions for the $\sigma \times \mathrm{BR}$ measurements together with those for the extracted $\mathrm{BR}$s [162–169].

**Table 8:** Expected relative errors for the $\sigma \times \mathrm{BR}$ measurements at $\sqrt{s}=250\,$GeV with $250\,$fb$^{-1}$ for $m_h=125\,$GeV

Process	Decay mode	${\varDelta } (\sigma \mathrm{BR})/(\sigma \mathrm{BR})$ (%)	${\varDelta } \mathrm{BR}/\mathrm{BR}$ (%)
*Zh*	$h \rightarrow b\bar{b}$	1.2	2.9
	$h \rightarrow c\bar{c}$	8.3	8.7
	$h \rightarrow gg$	7.0	7.5
	$h \rightarrow WW^*$	6.4	6.9
	$h \rightarrow \tau \bar{\tau }$	4.2	4.9
	$h \rightarrow ZZ^*$	19	19
	$h \rightarrow \gamma \gamma$	34	34

Notice that the cross section error, ${\varDelta } \sigma _{Zh}/\sigma _{Zh}=2.5\,\%$, eventually limits the precision of the BR measurements. We hence need more data at $\sqrt{s}=250$ GeV so as to improve the situation. We will return to the possible luminosity upgrade scenario later.

In order to extract couplings from branching ratios, we need the total width, since the coupling of the Higgs boson to a particle *A*, $g_{hAA}$, squared is proportional to the partial width which is given by the total width times the branching ratio:

17 $$\begin{aligned} g_{hAA}^2 \propto {\varGamma }(h \rightarrow AA) = {\varGamma }_h \cdot \mathrm{BR}(h \rightarrow AA). \end{aligned}$$

Solving this for the total width, we can see that we need at least one partial width and the corresponding branching ratio to determine the total width:

18 $$\begin{aligned} {\varGamma }_h = {\varGamma }(h \rightarrow AA) / \mathrm{BR}(h \rightarrow AA). \end{aligned}$$

In principle, we can use $A=Z$ or $A=W$, for which we can measure both the $\mathrm{BR}$s and the couplings. In the first case, $A=Z$, we can determine ${\varGamma }(h \rightarrow ZZ^*)$ from the recoil mass measurement and $\mathrm{BR}(h \rightarrow ZZ^*)$ from the $\sigma _{Zh} \times \mathrm{BR}(h \rightarrow ZZ^*)$ measurement together with the $\sigma _{Zh}$ measurement from the recoil mass. This method, however, suffers from the low statistics due to the small branching ratio, $\mathrm{BR}(h \rightarrow ZZ^*)= {\mathscr {O}}(1\,\%)$, A better way is to use $A=W$, where $\mathrm{BR}(h \rightarrow WW^*)$ is subdominant and ${\varGamma }(h \rightarrow WW^*)$ can be determined by the *WW*-fusion process: $e^+e^- \rightarrow \nu \bar{\nu }h$. The measurement of the *WW*-fusion process is, however, not easy at $\sqrt{s}=250$ GeV, since the cross section is small. Nevertheless, we can determine the total width to ${\varDelta } {\varGamma }_h /{\varGamma }_h = 11\,\%$ with 250 fb$^{-1}$ [170, 171]. Since the *WW*-fusion process becomes fully active at $\sqrt{s}=500$ GeV, a much better measurement of the total width is possible there, as will be discussed in the next subsection.

#### ILC at 500 GeV

At $\sqrt{s}=500$ GeV, the *WW*-fusion process $e^+e^- \rightarrow \nu \bar{\nu }h$ already starts dominating the Higgs-strahlung process: $e^+e^- \rightarrow Zh$. We can use this *WW*-fusion process for the $\sigma \times \mathrm{BR}$ measurements as well as to determine the total width to ${\varDelta } {\varGamma }_h / {\varGamma }_h = 5\,\%$ [171]. Table 9 summarises the $\sigma \times \mathrm{BR}$ measurements for various modes. We can see that the $\sigma _{\nu \bar{\nu }h} \times \mathrm{BR}(h \rightarrow b\bar{b})$ can be very accurately measured to better than $1\%$ and the $\sigma _{\nu \bar{\nu }h} \times \mathrm{BR}(h \rightarrow WW^*)$ to a reasonable precision with $500\,$fb$^{-1}$ at $\sqrt{s}=500\,$GeV. The last column of the table shows the results of ${\varDelta } \mathrm{BR}/ \mathrm{BR}$ from the global analysis combining all the measurements including the total cross section measurement using the recoil mass at $\sqrt{s}=250\,$GeV (2.6%) and $500\,$GeV (3%). The numbers in the parentheses are with the $250\,$GeV data alone. We can see that the ${\varDelta } \mathrm{BR}(h \rightarrow b\bar{b})/\mathrm{BR}(h \rightarrow b\bar{b})$ is already limited by the recoil mass measurements.

**Table 9:** Expected relative errors for the $\sigma \times \mathrm{BR}$ measurements at $\sqrt{s}=250$ GeV with $250\,$fb$^{-1}$ and at $\sqrt{s}=500\,$GeV with 500 fb$^{-1}$ for $m_h=125$ GeV and $(e^{-}, e^{+})=(-0.8, +0.3)$ beam polarisation. The last column of the table shows the relative errors on the branching ratios. Then the numbers in the parentheses are for 250 fb$^{-1}$ at $\sqrt{s}=250$ GeV alone

Energy (GeV) Mode	${\varDelta } (\sigma \cdot \mathrm{BR}) / (\sigma \cdot \mathrm{BR})$			${\varDelta } \mathrm{BR}/\mathrm{BR}$
	250	500		$250+500$
	*Zh* (%)	*Zh* (%)	$\nu \bar{\nu }h$ (%)	Combined (%)
$h \rightarrow b\bar{b}$	1.2	1.8	0.66	2.2 (2.9)
$h \rightarrow c\bar{c}$	8.3	13	6.2	5.1 (8.7)
$h \rightarrow gg$	7.0	11	4.1	4.0 (7.5)
$h \rightarrow WW^*$	6.4	9.2	2.4	3.1 (6.9)
$h \rightarrow \tau ^+\tau ^-$	4.2	5.4	9.0	3.7 (4.9)
$h \rightarrow ZZ^*$	19	25	8.2	7.5 (19)
$h \rightarrow \gamma \gamma$	29–38	29–38	20–26	17 (34)

**Fig. 46:**
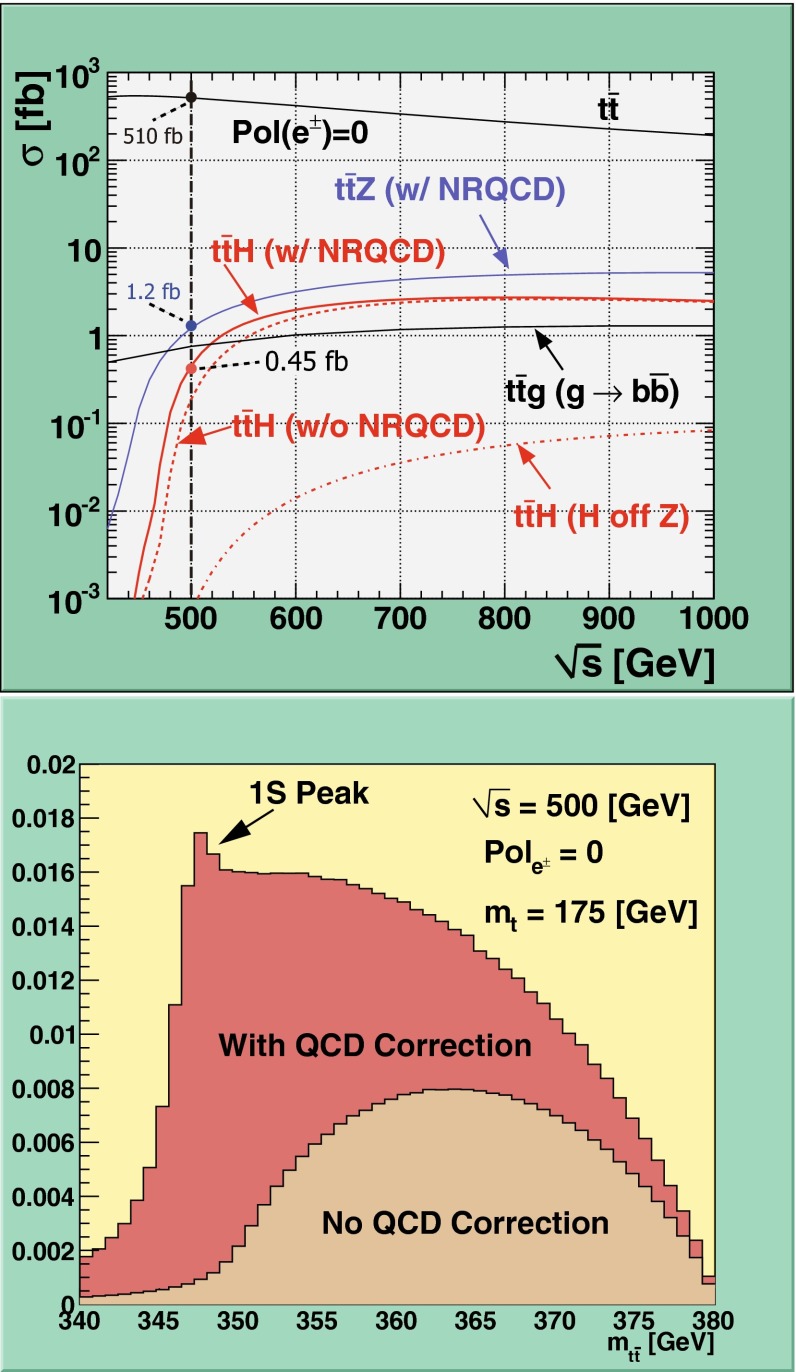
Cross sections for the signal $t\bar{t}h$ process with and without the non-relativistic QCD (NRQCD) correction together with those for the background processes: $t\bar{t}Z, t\bar{t}g (g \rightarrow b\bar{b})$ and $t\bar{t}$ (*upper plot*). The invariant mass distribution for the $t\bar{t}$ subsystem with and without the NRQCD correction (*lower plot*)

Perhaps more interesting than the branching ratio measurements is the measurement of the top Yukawa coupling using the $e^+e^- \rightarrow t\bar{t}h$ process [172–174], since it is the largest among matter fermions and not yet directly observed. Although its cross section maximum is reached at around $\sqrt{s}=800$ GeV as seen in Fig. 46, the process is accessible already at $\sqrt{s}=500$ GeV, thanks to the QCD bound-state effects (non-relativistic QCD correction) that enhance the cross section by a factor of 2 [173, 175–180]. Since the background diagram where a Higgs boson is radiated off the *s*-channel *Z* boson makes negligible contribution to the signal process, we can measure the top Yukawa coupling by simply counting the number of signal events. The expected statistical precision for the top Yukawa coupling is then ${\varDelta } g_Y(t) / g_Y(t) = 9.9\%$ for $m_h=125\,$GeV with 1ab$^{-1}$ at $\sqrt{s}=500\,$GeV [42, 181–185]. Notice that if we increase the centre-of-mass energy by $20\,$GeV, the cross section doubles. Moving up a little bit hence helps significantly.

**Fig. 47:**
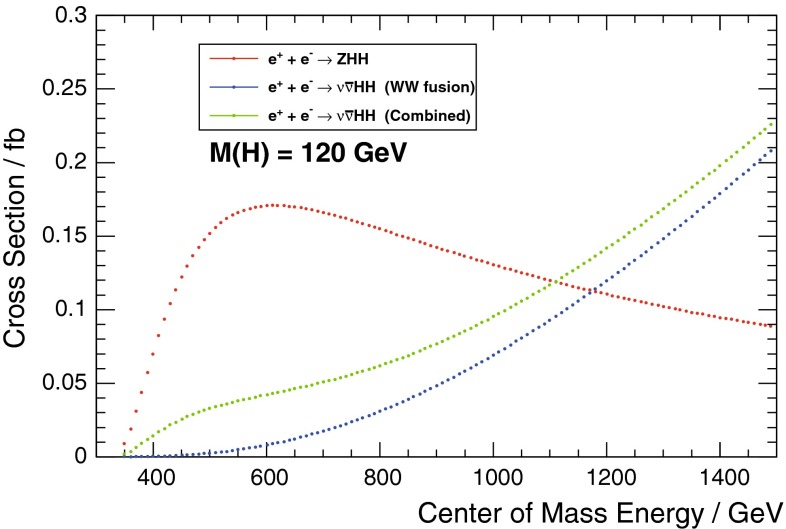
Cross sections for the double Higgs production processes, $e^+e^- \rightarrow Zhh$ and $e^+e^- \rightarrow \nu \bar{\nu }hh$, as a function of $\sqrt{s}$ for $m_h=120$ GeV

**Fig. 48:**
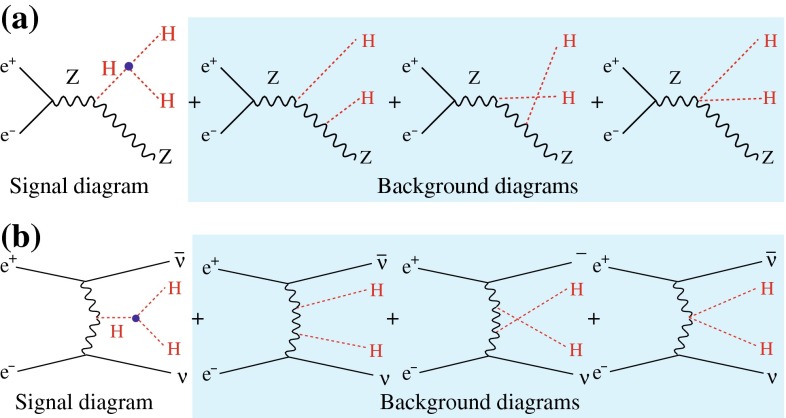
Diagrams contributing to **a** $e^+e^- \rightarrow Zhh$ and **b** $e^+e^- \rightarrow \nu \bar{\nu }hh$

**Fig. 49:**
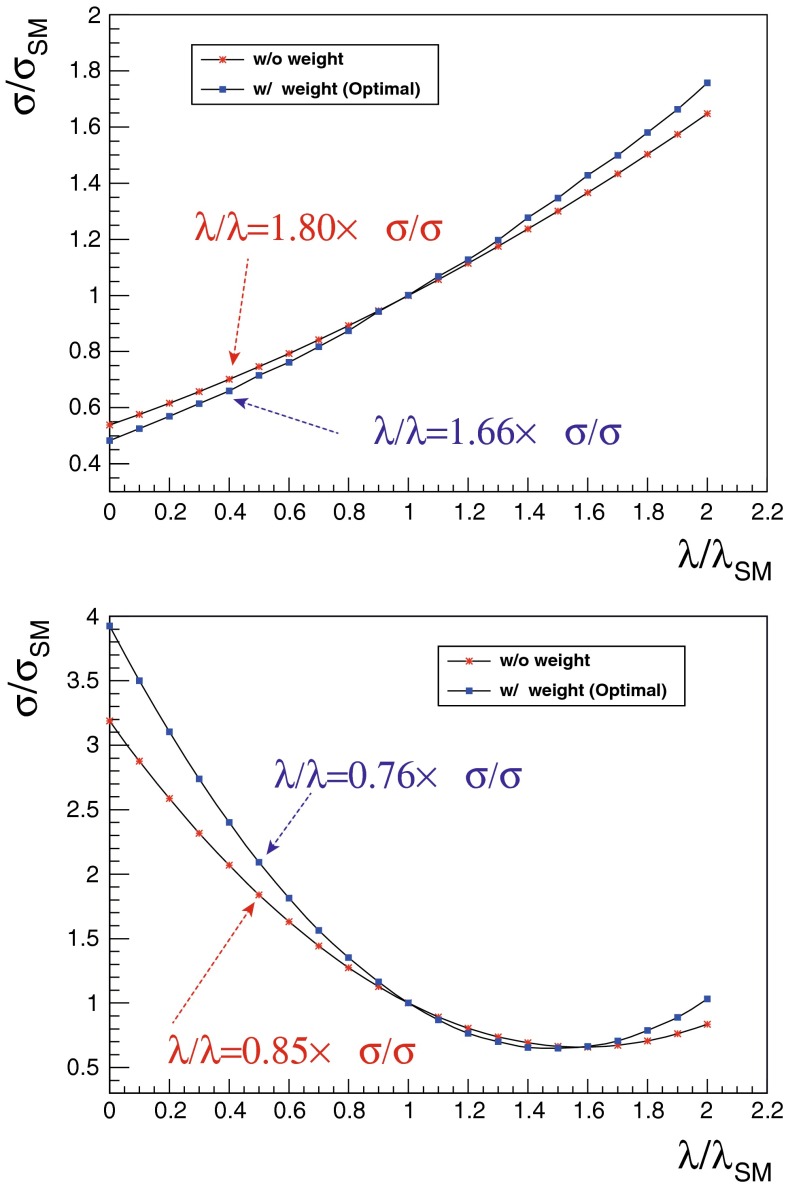
(*Upper plot*) cross section for $e^+e^- \rightarrow Zhh$ at $\sqrt{s}=500$ GeV normalised by that of the SM as a function of the self-coupling normalised by that of the SM. (*Lower plot*) a similar plot for $e^+e^- \rightarrow \nu \bar{\nu }hh$ at $\sqrt{s}=1$ TeV

Even more interesting is the measurement of the trilinear Higgs self-coupling, since it is to observe the force that makes the Higgs boson condense in the vacuum, which is an unavoidable step to uncover the secret of the EW symmetry breaking. In other words, we need to measure the shape of the Higgs potential. There are two ways to measure the tri-linear Higgs self-coupling. The first method is to use the double Higgs-strahlung process: $e^+e^- \rightarrow Zhh$ and the second is by the double Higgs production via *WW*-fusion: $e^+e^- \rightarrow \nu \bar{\nu }hh$. The first process attains its cross section maximum at around $\sqrt{s}=500$ GeV, while the second is negligible there but starts to dominate at energies above $\sqrt{s}\simeq 1.2$ TeV, as seen in Fig. 47. In any case the signal cross sections are very small (0.2 fb or less) and as seen in Fig. 48 irreducible background diagrams containing no self-coupling dilute the contribution from the self-coupling diagram, thereby degrading the sensitivity to the self-coupling, even if we can control the relatively huge SM backgrounds from $e^+e^- \rightarrow t\bar{t}$, *WWZ*, *ZZ*, $Z\gamma$, *ZZZ*, and *ZZh*. See Fig. 49 for the sensitivity factors for $e^+e^- \rightarrow Zhh$ at $\sqrt{s}=500$ GeV and $e^+e^- \rightarrow \nu \bar{\nu }hh$ at $\sqrt{s}=1$ TeV, which are 1.66 (1.80) and 0.76 (0.85), respectively, with (without) weighting to enhance the contribution from the signal diagram. Notice that if there were no background diagrams, the sensitivity factor would be 0.5. The self-coupling measurement is very difficult even in the clean environment of the ILC and requires a new flavour tagging algorithm that precedes jet-clustering, sophisticated neural-net-based data selection, and the event weighting technique [79, 186–191]. The current state of the art for the *Zhh* data selection is summarised in Table 10.

Combining all of these three modes, we can achieve *Zhh* excess significance of $5\sigma$ and measure the production cross section to ${\varDelta } \sigma / \sigma = 27\%$, which translates to a relative precision of $44 (48)\%$ for the self-coupling with (without) the event weighting for $m_h=120\,$GeV at $\sqrt{s}=500\,$GeV with $2\,$ab$^{-1}$ and $(e^{-}, e^{+})=(-0.8, +0.3)$ beam polarisation [186]. The expected precision is significantly worse than that of the cross section because of the background diagrams. Since the sensitivity factor for the $e^+e^- \rightarrow \nu \bar{\nu }hh$ process is much closer to the ideal 0.5 and since the cross section for this *WW*-fusion double Higgs production process increases with the centre-of-mass energy, $\sqrt{s} = 1 {\text {TeV}}$ is of particular interest, as will be discussed in the next subsection.

**Table 10:** The number of remaining events for the three event selection modes: $Zhh \rightarrow (\ell \bar{\ell })(b\bar{b})(b\bar{b})$, $(\nu \bar{\nu })(b\bar{b})(b\bar{b})$, and $(q\bar{q})(b\bar{b})(b\bar{b})$ and corresponding excess and measurement sensitivities for $m_h=120$ GeV at $\sqrt{s}=500$ GeV with $2\,$ab$^{-1}$ and $(e^{-}, e^{+})=(-0.8, +0.3)$ beam polarisation

Mode	Signal	BG	Significance	
			Excess	Meas.
$Zhh \rightarrow (\ell \bar{\ell })(b\bar{b})(b\bar{b})$	3.7	4.3	1.5$\sigma$	1.1$\sigma$
	4.5	6.0	1.5$\sigma$	1.2$\sigma$
$Zhh \rightarrow (\nu \bar{\nu })(b\bar{b})(b\bar{b})$	8.5	7.9	2.5$\sigma$	2.1$\sigma$
$Zhh \rightarrow (q\bar{q})(b\bar{b})(b\bar{b})$	13.6	30.7	2.2$\sigma$	2.0$\sigma$
	18.8	90.6	1.9$\sigma$	1.8$\sigma$

#### ILC at 1000 GeV

As we already pointed out the *WW*-fusion processes become more and more important at higher energies. In addition the machine luminosity usually scales with the centre-of-mass energy. Together with the better sensitivity factor we can hence improve the self-coupling measurement significantly at $\sqrt{s}=1\,$TeV, using the $e^+e^- \rightarrow \nu \bar{\nu }hh$ process. Table 11 summarises a full simulation result for the numbers of expected signal and background events before and after selection cuts with corresponding measurement significance values.

**Table 11:** The numbers of signal and background events before and after selection cuts and measurement significance for $m_h=120\,$GeV at $\sqrt{s}=1\,$TeV with 2 ab$^{-1}$ and $(e^{-}, e^{+})=(-0.8, +0.2)$ beam polarisation

Mode	No cut	After cuts
$\nu \bar{\nu }hh$ (*WW*-fusion)	272	35.7
$\nu \bar{\nu }hh$ (*Zhh*)	74.0	3.88
BG ($t\bar{t}/\nu \bar{\nu }Zh$)	$7.86 \times 10^{5}$	33.7
Meas. significance	0.30	4.29

With 2 ab$^{-1}$ and $(e^{-}, e^{+})=(-0.8, +0.2)$ beam polarisation at $\sqrt{s}=$ TeV, we would be able to determine the cross section for the $e^+e^- \rightarrow \nu \bar{\nu }hh$ process to ${\varDelta } \sigma / \sigma = 23~\%$, corresponding to the self-coupling precision of $\varDelta \lambda / \lambda = 18 (20)~\%$ with (without) the event weighting to enhance the contribution from the signal diagram for $m_h=120$ GeV [186]. According to preliminary results from a on-going full simulation study [192], adding $hh \rightarrow WW^*b\bar{b}$ would improve the self-coupling measurement precision by about 20 % relatively, which means $\varDelta \lambda / \lambda = 21\,\%$ for $m_h=125$ GeV with the baseline integrated luminosity of $1 ab^{-1}$ at 1 TeV.

At $\sqrt{s}=1\,$TeV, the $e^+e^- \rightarrow t\bar{t}h$ process is also near its cross section maximum, making concurrent measurements of the self-coupling and top Yukawa coupling possible. We will be able to observe the $e^+e^- \rightarrow t\bar{t}h$ events with $12\sigma$ significance in 8-jet mode and $8.7\sigma$ significance in lepton-plus-6-jet mode, corresponding to the relative error on the top Yukawa coupling of $\varDelta g_Y(t) / g_Y(t) = 3.1~\%$ with $1ab^{-1}$ and $(e^{-}, e^{+})=(-0.8, +0.2)$ beam polarisation at $\sqrt{s}=1\,$TeV for $m_h=125\,$GeV [193].

However, an obvious but most important advantage of higher energies in terms of Higgs physics is its higher mass reach to extra Higgs bosons expected in extended Higgs sectors and higher sensitivity to $W_LW_L$ scattering to decide whether the Higgs sector is strongly interacting or not. In any case thanks to the higher cross section for the *WW*-fusion $e^+e^- \rightarrow \nu \bar{\nu }h$ process at $\sqrt{s}=1\,$TeV, we can expect significantly better precisions for the $\sigma \times \mathrm{BR}$ measurements (see Table 12), which also allows us to access very rare decays such as $h \rightarrow \mu ^+\mu ^-$ [191, 194].

**Table 12:** Independent Higgs measurements using the Higgs-strahlung (*Zh*) and the *WW*-fusion ($\nu \bar{\nu }h$) processes for $m_h=125\,$GeV at three energies: $\sqrt{s}=250\,$GeV with $250\,$fb$^{-1}$, $500\,$GeV with $500\,$fb$^{-1}$ both with $(e^{-}, e^{+})=(-0.8, +0.3)$ beam polarisation, $\sqrt{s}=1\,$TeV with $1ab^{-1}$ and $(e^{-}, e^{+})=(-0.8, +0.2)$ beam polarisation

$\sqrt{s}$	250 GeV		500 GeV		1 TeV
Lumi.	250 fb$^{-1}$		500 fb$^{-1}$		1 ab$^{-1}$
Process	*Zh*	$\nu \bar{\nu }h$	*Zh*	$\nu \bar{\nu }h$	$\nu \bar{\nu }h$
	$$\varDelta \sigma / \sigma $$				
	2.6%	–	3.0%	–	–
Mode	$\varDelta (\sigma \cdot \mathrm{BR}) / (\sigma \cdot \mathrm{BR})$				
$h \rightarrow b\bar{b}$ (%)	1.2	10.5	1.8	0.66	0.5
$h \rightarrow c\bar{c}$ (%)	8.3		13	6.2	3.1
$h \rightarrow gg$ (%)	7.0		11	4.1	2.3
$h \rightarrow WW^*$ (%)	6.4		9.2	2.4	1.6
$h \rightarrow \tau ^+\tau ^-$ (%)	4.2		5.4	9.0	3.1
$h \rightarrow ZZ^*$ (%)	18		25	8.2	4.1
$h \rightarrow \gamma \gamma$ (%)	34		34	23	8.5
$h \rightarrow \mu ^+\mu ^-$ (%)	100	–	–	–	31

#### ILC 250 + 500 + 1000: global fit for couplings

The data at $\sqrt{s}=250$, 500, and $1000\,$GeV can be combined to perform a global fit to extract various Higgs couplings [195]. We have 33 $\sigma \times \mathrm{BR}$ measurements: 31 shown in Table 12 plus two $\sigma (t\bar{t}h) \times \mathrm{BR}(h\rightarrow b\bar{b})$ measurements at $\sqrt{s}=500$ and $1000\,$GeV. The key is the recoil mass measurement that unlocks the door to a fully model-independent analysis. Notice that such a fully model-independent analysis is impossible at the LHC. As shown in Table 12, we can measure the recoil mass cross section at $\sqrt{s}=250$ and $500\,$GeV. Altogether we have 35 independent measurements: 33 $\sigma \times \mathrm{BR}$ measurements ($Y_i : i=1\ldots 33$) and 2 $\sigma (Zh)$ measurements ($Y_{34,35}$). We can then define a $\chi ^2$ function:

19 $$\begin{aligned} \chi ^2= & {} \sum _{i=1}^{35} \left( \frac{Y_i - Y'_i}{\varDelta Y_i}\right) ^2 \end{aligned}$$

where

20 $$\begin{aligned} Y'_i := F_i \cdot \frac{g^2_{hA_i A_i} g^2_{hB_i B_i}}{{\varGamma }_0} \quad (i=1, \ldots , 33) \end{aligned}$$

with $A_i$ being *Z*, *W*, or *t*, and $B_i$ being *b*, *c*, $\tau$, $\mu$, *g*, $\gamma$, *Z*, and *W*, $\varGamma _0$ denoting the total width and

21 $$\begin{aligned} F_i= & {} S_i G_i \end{aligned}$$

with

22 $$\begin{aligned}&S_i = \left( \frac{\sigma _{Zh}}{g^2_{hZZ}}\right) , ~ \left( \frac{\sigma _{\nu \bar{\nu }h}}{g^2_{hWW}}\right) , ~\mathrm{or} ~ \left( \frac{\sigma _{t\bar{t}h}}{g^2_{htt}}\right) \nonumber \\&G_i = \left( \frac{\varGamma _i}{g^2_i} \right) . \end{aligned}$$

**Fig. 50:**
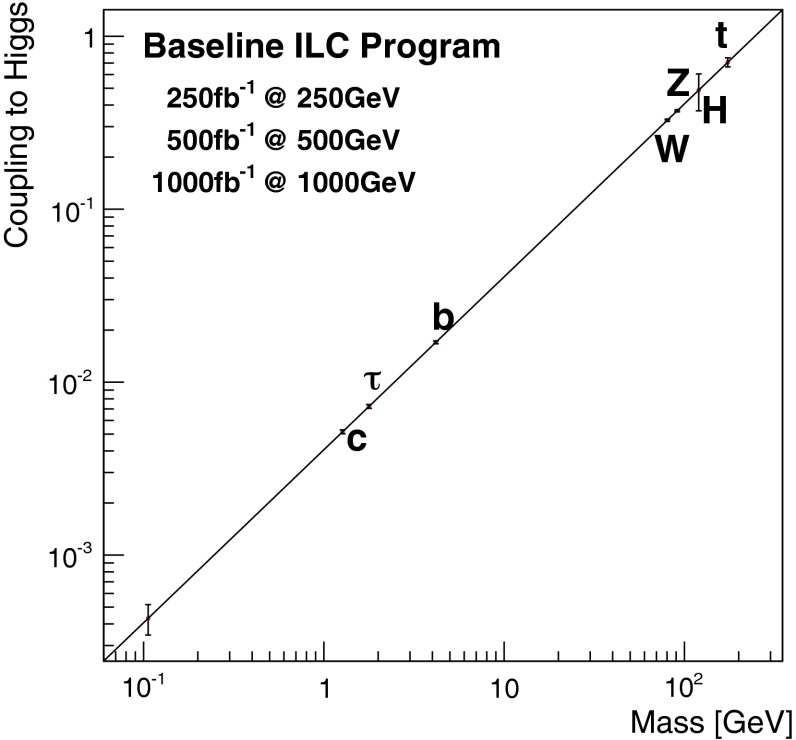
Expected mass–coupling relation for the SM case after the full ILC programme

Cross section calculations ($S_i$) do not involve QCD ISR unlike with the LHC. Partial width calculations ($G_i$), being normalised by the coupling squared, do not need quark mass as input. We are hence confident that the goal theory errors for $S_i$ and $G_i$ will be at the 0.1% level at the time of ILC running. The free parameters are 9 coupling constants: $g_{hbb}$, $g_{gcc}$, $g_{h\tau \tau }$, $g_{h\mu \mu }$, $g_{hgg}$, $g_{h\gamma \gamma }$, $g_{hZZ}$, $g_{hWW}$, and 1 total width: ${\varGamma }_0$. Table 13 summarises the expected coupling precisions for $m_h=125\,$GeV with the baseline integrated luminosities of 250 fb$^{-1}$ at $\sqrt{s}=250\,$GeV, 500 fb$^{-1}$ at 500 GeV both with $(e^{-}e^{+})=(-0.8, +0.3)$ beam polarisation, and 1 ab$^{-1}$ at 1 TeV with $(e^{-}e^{+})=(-0.8, +0.2)$ beam polarisation. The expected coupling precisions are plotted in the mass–coupling plot expected for the SM Higgs sector in Fig. 50. The error bars for most couplings are almost invisible in this logarithmic plot.

**Table 13:** Expected precisions for various couplings of the Higgs boson with $m_h=125\,$GeV from a model-independent fit to observables listed in Table 12 at three energies: $\sqrt{s}=250$ GeV with 250 fb$^{-1}$, 500 GeV with 500 fb$^{-1}$ both with $(e^{-}, e^{+})=(-0.8, +0.3)$ beam polarisation, $\sqrt{s}=1$ TeV with $2ab^{-1}$ and $(e^{-}, e^{+})=(-0.8, +0.2)$ beam polarisation, cf. [29] and Scen. ’Snow’ in [27]. $^\mathrm{a}$Values assume inclusion of $hh\rightarrow WW^*b\bar{b}$ decays

Coupling	$\sqrt{s}$ (GeV)		
	250	250 + 500	250 + 500 + 1000
*hZZ* (%)	1.3	1.0	1.0
*hWW* (%)	4.8	1.1	1.1
*hbb* (%)	5.3	1.6	1.3
*hcc* (%)	6.8	2.8	1.8
*hgg* (%)	6.4	2.3	1.6
$h\tau \tau$ (%)	5.7	2.3	1.6
$h\gamma \gamma$ (%)	18	8.4	4.0
$h\mu \mu$ (%)	91	91	16
${\varGamma }_0$ (%)	12	4.9	4.5
*htt* (%)	–	14	3.1
*hhh* (%)	–	83$^\mathrm{a}$	21$^\mathrm{a}$

#### Synergy: LHC + ILC

**Fig. 51:**
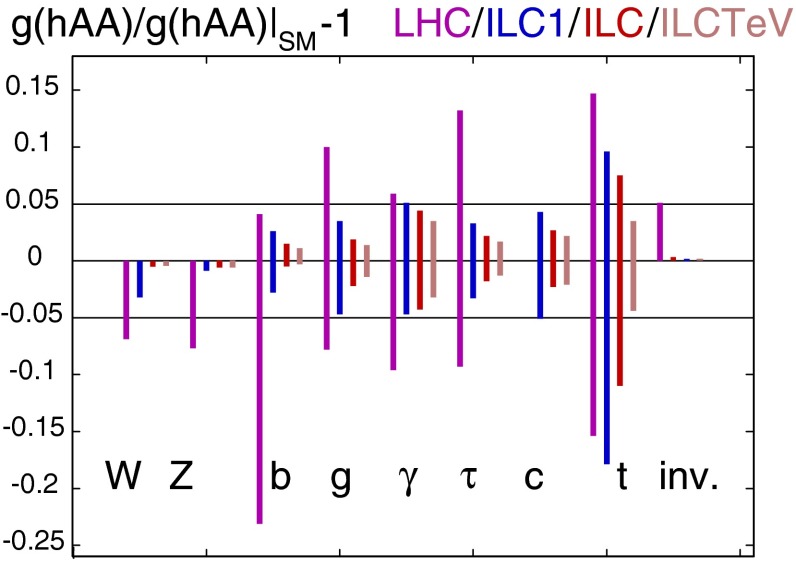
Comparison of the capabilities of the LHC and the ILC, when the ILC data in various stages: ILC1 with 250 fb$^{-1}$ at $\sqrt{s}=250$, ILC: 500 fb$^{-1}$ at 500 GeV, and ILCTeV: $1ab^{-1}$ at 1 TeV are cumulatively added to the LHC data with 300 fb$^{-1}$ at 14 TeV [197]

So far we have been discussing the precision Higgs physics expected at the ILC. It should be emphasised, however, that the LHC is expected to impose significant constraints on possible deviations of the Higgs-related couplings from their SM values by the time the ILC will start its operation, even though fully model-independent analysis is impossible with the LHC alone. Nevertheless, Refs. [196, 197] demonstrated that with a reasonably weak assumption such as the *hWW* and *hZZ* couplings will not exceed the SM values the LHC can make reasonable measurements of most Higgs-related coupling constants except for the *hcc* coupling. Figure 51 shows how the coupling measurements would be improved by adding, cumulatively, information from the ILC with $250\,$fb$^{-1}$ at $\sqrt{s}=250$, $500\,$fb$^{-1}$ at $500\,$GeV, and $1\,$ab$^{-1}$ at $1\,$TeV to the LHC data with $300\,$fb$^{-1}$ at $14\,$TeV.

The figure tells us that the addition of the $250\,$GeV data, the *hZZ* coupling in particular, from the ILC allows the absolute normalisation and significantly improves all the couplings. It is interesting to observe the synergy for the measurement of the $h\gamma \gamma$ coupling, whose precision significantly exceeds that of the ILC alone. This is because the LHC can precisely determine the ratio of the $h\gamma \gamma$ coupling to the *hZZ* coupling, while the ILC provides a precision measurement of the *hZZ* coupling from the recoil mass measurement. The addition of the $500\,$GeV data from the ILC further improves the precisions, this time largely due to the better determination of the Higgs total width. Finally as we have seen above, the addition of the $1\,$TeV data from the ILC improves the top Yukawa coupling drastically with even further improvements of all the other couplings except for the *hWW* and *hZZ* couplings which are largely limited by the cross section error from the recoil mass measurement at $\sqrt{s}=250\,$GeV. This way we will be able to determine these couplings to ${\mathscr {O}}(1~\%)$ or better. The *SFitter* group performed a similar but more model-independent analysis and obtained qualitatively the same conclusions [198]. This level of precision matches what we need to fingerprint different BSM scenarios, when nothing but the 125 GeV boson would be found at the LHC (see Table 14). These numbers can be understood from the following formulae for the three different models in the decoupling limit (see [147] for definitions and details):

$$\begin{aligned}&\text{ Mixing } \text{ with } \text{ singlet: } \\&\frac{g_{hVV}}{g_{h_\mathrm{SM} VV}} = \frac{g_{hff}}{g_{h_\mathrm{SM} ff}} = \cos \theta \simeq 1 - \frac{\delta ^2}{2} \\&\text{ Composite } \text{ Higgs: } \\&\frac{g_{hVV}}{g_{h_\mathrm{SM}VV}} \simeq 1 - 3\% \left( \frac{1~\mathrm{TeV}}{f} \right) ^2 \\&\frac{g_{hff}}{g_{h_\mathrm{SM}ff}} \simeq \left\{ \begin{array}{l@{\quad }l} \textstyle 1 - 3\% \left( \frac{1~\mathrm{TeV}}{f} \right) ^2 &{} \mathrm{(MCHM4)} \\ \textstyle 1 - 9\% \left( \frac{1~\mathrm{TeV}}{f} \right) ^2 &{} \mathrm{(MCHM5).} \end{array} \right. \\&\text{ Supersymmetry: } \\&\frac{g_{hbb}}{g_{h_\mathrm{SM} bb}} = \frac{g_{h \tau \tau }}{g_{h_\mathrm{SM} \tau \tau }} \simeq 1 + 1.7\% \left( \frac{1 \ \mathrm{TeV}}{m_A} \right) ^2. \end{aligned}$$

**Table 14:** Maximum possible deviations when nothing but the 125 GeV boson would be found at the LHC [199]

	$\varDelta hVV$ (%)	$\varDelta h\bar{t}t$	$\varDelta h\bar{b}b$
Mixed-in singlet	6	6 %	6 %
Composite Higgs	8	tens of %	tens of %
Minimal SUSY	$<$1	3 %	10 %$^\mathrm{a}$, 100 %$^\mathrm{b}$
LHC 14 TeV, $3ab^{-1}$	8	10 %	15 %

$^\mathrm{a}$ $\tan \beta > 20$, no SUSY found at LHC

$^\mathrm{b}$ All other cases, 100 % reached for $\tan \beta \sim 5$

**Table 15:** Expected Higgs precisions on normalised Higgs couplings ($\kappa _i := g_i / g_i (\mathrm{SM})$) for $m_h=125\,$GeV from model-dependent 7-parameter fits for the LHC and the ILC, where $\kappa _c = \kappa _t =: \kappa _u$, $\kappa _s = \kappa _b =: \kappa _d$, $\kappa _\mu = \kappa _\tau =: \kappa _\ell$, and ${\varGamma }_\mathrm{tot} = \sum {\varGamma }_i^\mathrm{SM} \, \kappa _i^2$ are assumed

Facility	LHC	HL-LHC	ILC500	ILC1000
$\sqrt{s}\,$(GeV)	1,400	14,000	250/500	250/500/1000
$\int {\mathscr {L}} \mathrm{d}t\,$(fb$^{-1}$)	300/exp (%)	3000/exp (%)	250 + 500 (%)	250 + 500 + 1000 (%)
$\kappa _\gamma$	5–7	2–5	8.3	3.8
$\kappa _g$	6–8	3–5	2.0	1.1
$\kappa _W$	4–6	2–5	0.39	0.21
$\kappa _Z$	4–6	2–4	0.49	0.50
$\kappa _\ell$	6–8	2–5	1.9	1.3
$\kappa _d$	10–13	4–7	0.93	0.51
$\kappa _u$	14–15	7–10	2.5	1.3

The different models predict different deviation patterns. The ILC together with the LHC will be able to fingerprint these models or set the lower limit on the energy scale for BSM physics.

#### Model-dependent global fit: example of fingerprinting

**Fig. 52:**
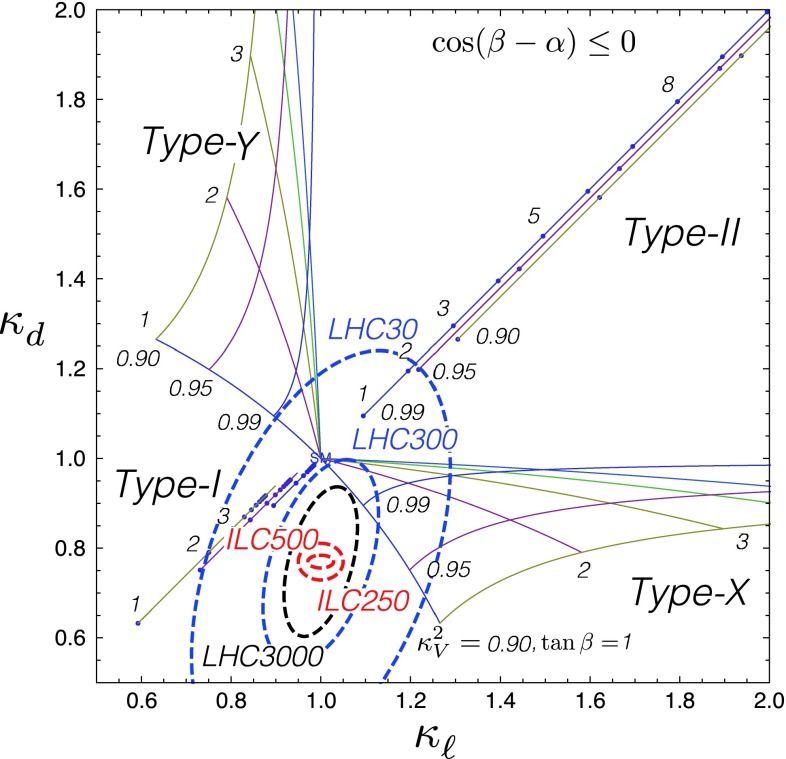
Comparison of the model-discrimination capabilities of the LHC and the ILC [200]

**Fig. 53:**
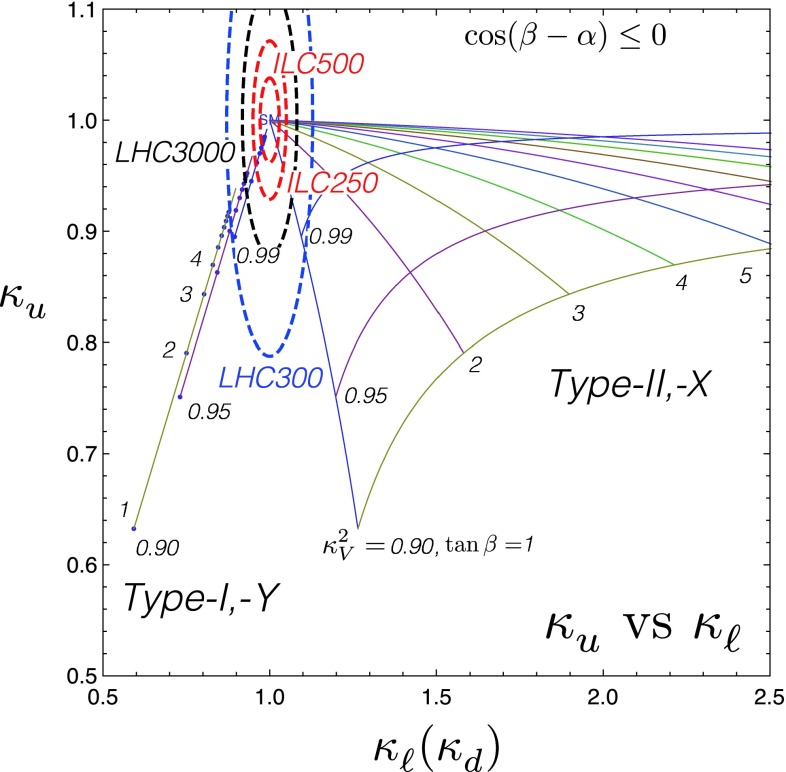
Comparison of the model-discrimination capabilities of the LHC and the ILC [200]

**Fig. 54:**
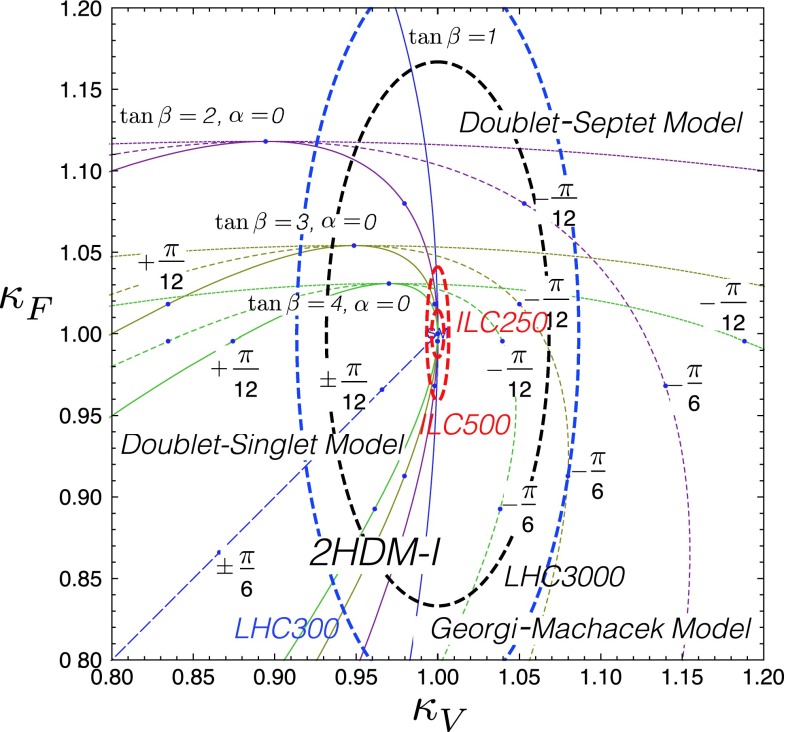
Comparison of the model-discrimination capabilities of the LHC and the ILC [200]

As mentioned above, the LHC needs some model assumption to extract Higgs couplings. If we use stronger model assumptions we may have higher discrimination power at the cost of loss of generality. As an example of such a model-dependent analysis, let us consider here a 7-parameter global fit with the following assumptions:

23 $$\begin{aligned}&\kappa _c = \kappa _t =: \kappa _u , \nonumber \\&\kappa _s = \kappa _b =: \kappa _d , \nonumber \\&\kappa _\mu = \kappa _\tau =: \kappa _\ell , \nonumber \\&\text{ and } \nonumber \\&{\varGamma }_\mathrm{tot} = \sum _{i \in \text {SM decays}} \, {\varGamma }_i^\mathrm{SM} \, \kappa _i^2 , \end{aligned}$$

where $\kappa _i :\,= g_i / g_i (\mathrm{SM})$ is a Higgs coupling normalised by its SM value. The first three of these constrain the relative deviations of the up-type and down-type quark Yukawa couplings as well as that of charged leptons to be common in each class, while the last constraint restricts unknown decay modes to be absent. The results of the global fits assuming projected precisions for the LHC and the ILC are summarised in Table 15 [195]. Figures 52 and 53 compare the model discrimination power of the LHC and the ILC in the $\kappa _\ell$–$\kappa _d$ and $\kappa _\ell (\kappa _d)$–$\kappa _u$ planes for the four types of two-Higgs-doublet model discussed in Sect. 2.3.1 [141, 200]. Figure 54 is a similar plot in the $\kappa _V$–$\kappa _F$ plane showing the discrimination power for four models: doublet-singlet model, 2HDM-I, Georgi–Machacek model, and doublet–septet model, all of which naturally realise $\rho = 1$ at the tree level [141, 200].

#### High luminosity ILC?

**Fig. 55:**
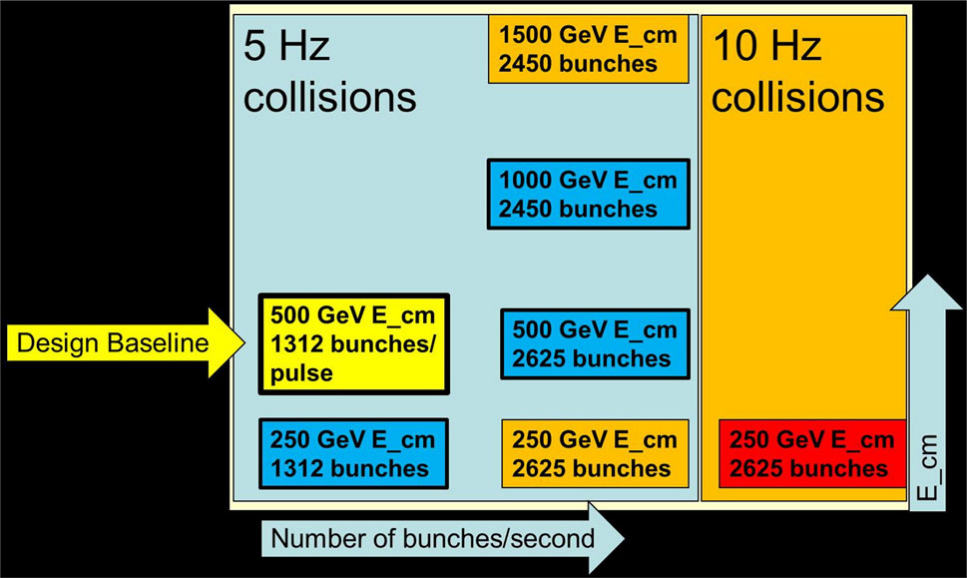
Possible machine upgrade scenarios for the ILC [141, 201]

We have seen the crucial role played by the recoil mass measurement for the model-independent coupling extraction. We have also pointed out that because of this the recoil mass measurement would eventually limit the coupling precisions achievable with the ILC. Given the situation, let us now consider the possibility of luminosity upgrade. As a matter of fact, the ILC technical design report (TDR) [201] describes some possible luminosity and energy upgrade scenarios, which are sketched in Fig. 55 as blue boxes.

In order to improve the recoil mass measurement significantly a new luminosity upgrade option (doubling of the number of bunches plus 10 Hz collisions instead of nominal 5 Hz) was proposed for the 250 GeV running in the Snowmass 2013 process [141] (see the red box in Fig. 55). It should be noted that the number of bunches was 2625 in the original ILC design given in the reference design report [202], which was reduced to 1312 in the TDR so as to reduce the construction cost. The 10 Hz operation is practical at 250 GeV, since the needed wall plug power is lower at the lower energy. The upgrade would hence allow a factor of 4 luminosity upgrade at $\sqrt{s}=250$ GeV. Let us now assume that after the baseline programme at $\sqrt{s}=250$, 500, and 1000 GeV we will run at the same three energies with the luminosity upgrade, thereby achieving $1150\,$fb$^{-1}$ at 250 GeV, $1600\,$fb$^{-1}$ at 500 GeV, and $2500\,$fb$^{-1}$ at 1000 GeV.

The expected precisions for the independent Higgs-related measurements are summarised in Table 16 for the full data after the luminosity upgraded running. Corresponding expected precisions for various Higgs couplings are tabulated in Table 17. The table shows that with the luminosity upgrade we can achieve sub-% level precisions for most of the Higgs couplings even with the completely model-independent analysis.

**Table 16:** Similar table to Table 12 but with the luminosity upgrade described in the text: 1150 fb$^{-1}$ at 250 GeV, 1600 fb$^{-1}$ at 500 GeV, and 2500 fb$^{-1}$ at 1 TeV

$\sqrt{s}$	250 GeV		500 GeV		1 TeV
Lumi.	1150 fb$^{-1}$		1600 fb$^{-1}$		2.5 ab$^{-1}$
Process	*Zh*	$\nu \bar{\nu }h$	*Zh*	$\nu \bar{\nu }h$	$\nu \bar{\nu }h$
	$$\varDelta \sigma / \sigma $$				
	1.2 %	–	1.7 %	–	–
Mode	$\varDelta (\sigma \cdot \mathrm{BR}) / (\sigma \cdot \mathrm{BR})$				
$h \rightarrow b\bar{b}$ (%)	0.56	4.9	1.0	0.37	0.3
$h \rightarrow c\bar{c}$ (%)	3.9		7.2	3.5	2.0
$h \rightarrow gg$ (%)	3.3		6.0	2.3	1.4
$h \rightarrow WW^*$ (%)	3.0		5.1	1.3	1.0
$h \rightarrow \tau ^+\tau ^-$ (%)	2.0		3.0	5.0	2.0
$h \rightarrow ZZ^*$ (%)	8.4		14	4.6	2.6
$h \rightarrow \gamma \gamma$ (%)	16		19	13	5.4
$h \rightarrow \mu ^+\mu ^-$ (%)	46.6	–	–	–	20

#### Conclusions

The primary goal for the next decades is to uncover the secret of the EWSB. This will open up a window to BSM and set the energy scale for the energy frontier machine that will follow the LHC and the ILC 500. Probably the LHC will hit systematic limits at *O*(5–10 %) for most of $\sigma \times \mathrm{BR}$ measurements, being insufficient to see the BSM effects if we are in the decoupling regime. The recoil mass measurements at the ILC unlocks the door to a fully model-independent analysis. To achieve the primary goal we hence need a 500 GeV linear collider for self-contained precision Higgs studies to complete the mass–coupling plot, where we start from $e^+e^- \rightarrow Zh$ at $\sqrt{s}=250\,$GeV, then $t\bar{t}$ at around $350\,$GeV, and then *Zhh* and $t\bar{t}h$ at $500\,$GeV. The ILC to cover up to $\sqrt{s}=500\,$GeV is an ideal machine to carry out this mission (regardless of BSM scenarios) and we can do this *completely model-independently* with staging starting from $\sqrt{s}\simeq 250\,$GeV. We may need more data at this energy depending on the size of the deviation, since the recoil mass measurement eventually limits the coupling precisions. Luminosity upgrade possibility should be always kept in our scope. If we are lucky, some extra Higgs boson or some other new particle might be within reach already at the ILC 500. Let us hope that the upgraded LHC will make another great discovery in the next run from 2015. If not, we will most probably need the energy scale information from the precision Higgs studies. Guided by the energy scale information, we will go hunt direct BSM signals, if necessary, with a new machine. Eventually we will need to measure $W_L W_L$ scattering to decide if the Higgs sector is strongly interacting or not.

**Table 17:** Similar table to Table 13 but with the luminosity upgrade described in the text: 1150 fb$^{-1}$ at 250 GeV, 1600 fb$^{-1}$ at 500 GeV, and 2500 fb$^{-1}$ at 1 TeV, cf. [29] and Scen. ’Snow’ in [27]. $^\mathrm{a}$ Values assume inclusion of $hh\rightarrow WW^*b\bar{b}$ decays

Coupling	$\sqrt{s}$ (GeV)		
	250	250 + 500	250 + 500 + 1000
*hZZ* (%)	0.6	0.5	0.5
*hWW* (%)	2.3	0.6	0.6
*hbb* (%)	2.5	0.8	0.7
*hcc* (%)	3.2	1.5	1.0
*hgg* (%)	3.0	1.2	0.93
$h\tau \tau$ (%)	2.7	1.2	0.9
$h\gamma \gamma$ (%)	8.2	4.5	2.4
$h\mu \mu$ (%)	42	42	10
${\varGamma }_0$ (%)	5.4	2.5	2.3
*htt* (%)	–	7.8	1.9
*hhh* (%)	–	46$^\mathrm{a}$	13$^\mathrm{a}$

### Higgs at CLIC: prospects^10^

#### Introduction

The CLIC accelerator [203] offers the possibility to study $e^+e^-$ collisions at centre-of-mass energies from 350 GeV up to 3 TeV. The novel CLIC acceleration schemes uses a two-beam acceleration scheme and normal conducting cavities, which operate at room temperature. A high-intensity drive beam generates the necessary RF power at 12 GHz, which is then used to accelerate the main beam. Compared to the ILC [204], the pulse length is significantly shorter (150 ns) with a bunch spacing of just 0.5 ns and a repetition rate of 50 Hz.

**Fig. 56:**
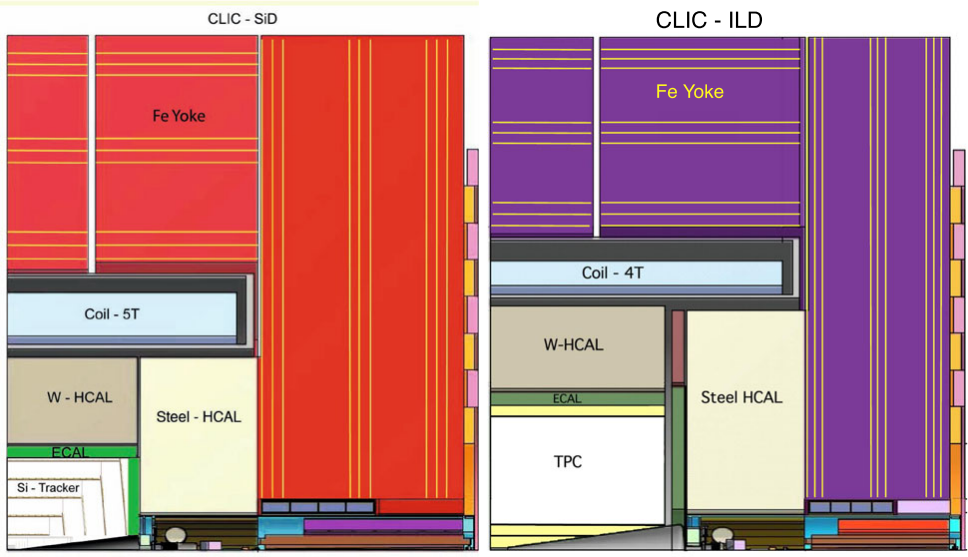
Longitudinal cross section of the top quadrant of CLIC_SiD (*left*) and CLIC_ILD (*right*) [9, 10]

The detectors used for the CLIC physics and detector studies [9, 10] are based on the SiD [205, 206] and ILD [206, 207] detectors proposed for the ILC. They have been adapted for the more challenging environment of running at $\sqrt{s}=3$ TeV. The most significant changes for both CLIC_SID and CLIC_ILD (see Fig. 56) is the use of tungsten in the hadronic calorimeter and an increase of the depth of hadronic calorimeter to 7.5 ${\varLambda }_{\mathrm {int}}$.

Running in the multi-TeV regime and with small intense bunches means that the CLIC detectors experience much higher backgrounds from beamstrahlung. This also leads to a long tail of the luminosity spectrum. To cope with these harsh backgrounds, the CLIC detectors plan to use highly granular detectors with time-stamping on the 10 ns level in for the tracking detectors and 1 ns level for the calorimeters in order to suppress these backgrounds [9, 10].

**Fig. 57:**
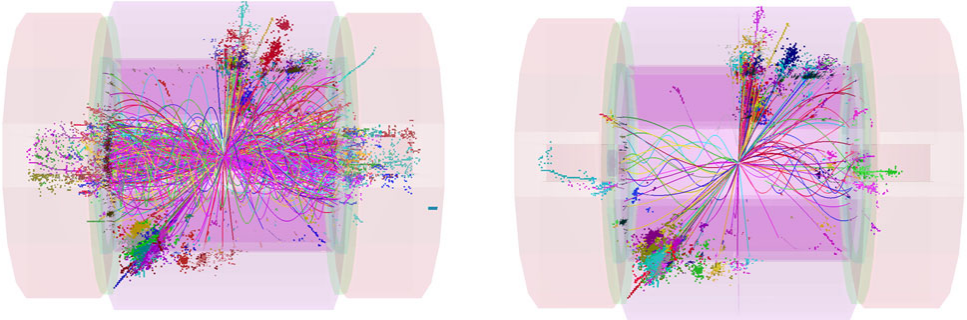
Reconstructed particles in a simulated $e^+e^-\rightarrow H^+H^- \rightarrow t\bar{b}\bar{t}b$ event at $\sqrt{s}$=3 TeV in the CLIC_ILD detector including the background from $\gamma \gamma \rightarrow \text{ hadrons }$ before (*left*) and after (*right*) applying tight timing cuts on the reconstructed cluster times [9, 10]

An entire bunch train at CLIC roughly deposits around 20 TeV in the detector, which is predominantly coming from $\gamma \gamma \rightarrow \text{ hadrons }$ events. By applying tight cuts on the reconstructed particles this number can be reduced to about 100 GeV. Using hadron-collider type jet-clustering algorithms, which treat the forward particles in a similar way to an underlying event this can be even further improved [9, 10]. The impact of this approach is illustrated with a reconstructed $e^+e^-\rightarrow H^+H^- \rightarrow t\bar{b}\bar{t}b$ event in the CLIC_ILD detector (see Fig. 57).

This section focusses on the production of heavy Higgs bosons ($H, A, H^\pm$), which are predicted in extended models like the 2HDM or supersymmetric models. The CLIC capabilities for studying light, SM-like Higgs bosons are summarised elsewhere [9, 10, 208].

#### Searches for heavy Higgs Bosons

**Fig. 58:**
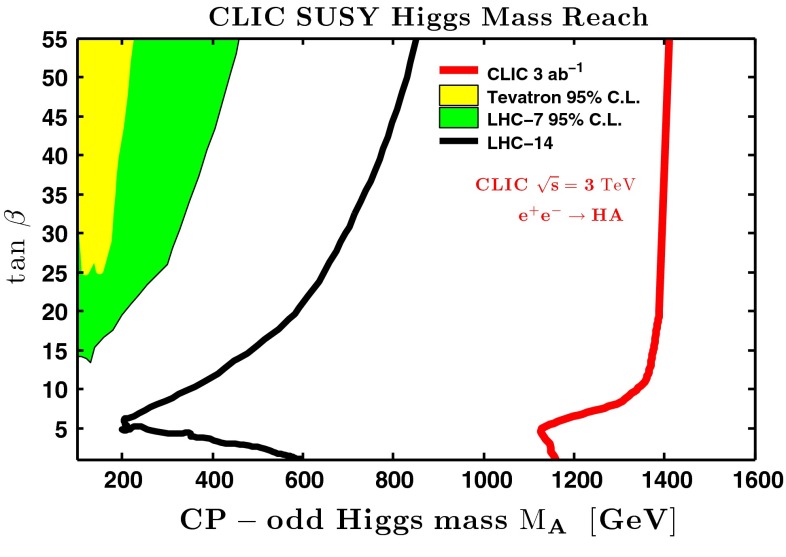
Search reach in the $m_{\mathrm {A}}-\tan \beta$ plane for LHC and CLIC. The *left-most coloured regions* are current limits from the Tevatron with $\sim$7.5 $\mathrm {fb}^{-1}$ of data at $\sqrt{s}=1.96$ TeV and from $\sim$1 $\mathrm {fb}^{-1}$ of LHC data at $\sqrt{s}=7$ TeV. The *black line* is projection of search reach at LHC with $\sqrt{s}=14$ TeV and 300 $\mathrm {fb}^{-1}$ of luminosity [211]. The *right-most red line* is search reach of CLIC in the HA mode with $\sqrt{s}=3$ TeV. This search capacity extends well beyond the LHC [9, 10]

In many supersymmetric scenarios, the Higgs sector consists of one light Higgs boson *h*, consistent with a SM Higgs boson, while the remaining four Higgs bosons are almost mass degenerate and have masses way beyond 500 GeV, see Sect. 2.5. These scenarios are consistent with current results from ATLAS and CMS on the Higgs boson [209, 210]. If this scenario for the Higgs sector has been realised, it will be extremely challenging to discover these additional final states at the LHC, especially in the low $\tan \beta$ regime, where e.g. the reach for the pseudoscalar *A* can be as low as 200 GeV (see Fig. 58).

The pair production processes $e^+e^-\rightarrow H^+H^-$ and $e^+e^-\rightarrow HA$ will give access to these heavy Higgs bosons almost up to the kinematic limit [212, 213]. Two separate scenarios have recently been studied [9, 10], with a mass of the pseudoscalar Higgs boson A of $m_A$=902 GeV (Model I) or $m_A$=742 GeV (Model II). In both scenarios, the dominant decay modes are $HA\rightarrow b\bar{b}b\bar{b}$ and $H^{+}H^{-}\rightarrow t\bar{b}\bar{t}b$. As already mentioned above, the analyses use the anti-$k_T$ algorithm that has been developed for the LHC in order to suppress the background originating from $\gamma \gamma \rightarrow \mathrm{hadrons}$.

**Fig. 59:**
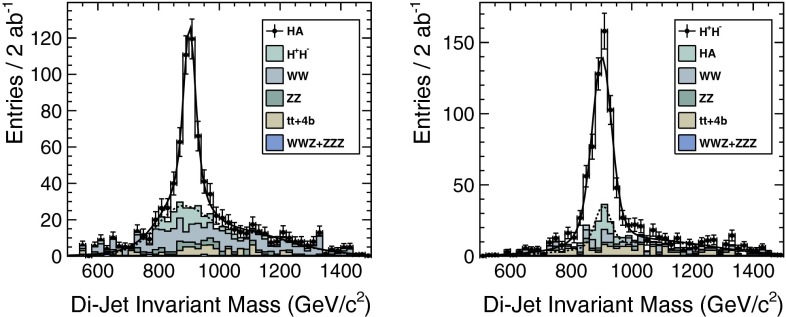
Di-jet invariant mass distributions for the $e^+e^-\rightarrow HA\rightarrow b\bar{b}b\bar{b}$ (*left*) and the $e^+e^-\rightarrow H^{+}H^{-}\rightarrow t\bar{b}\bar{t}b$ (*right*) signal together with the individual background contributions for model I [9, 10].

The resulting di-jet mass distributions including the background processes are shown in Figs. 59 (Model I) and 60 (Model II). The achievable accuracy on the Higgs-boson mass using a dataset of 2 $ab^{-1}$ at $\sqrt{s}=3$ TeV is about 0.3 % [9, 10] and the width can be determined with an accuracy of 17–31 % for the $b\bar{b}b\bar{b}$ final state and 23–27 % for the $t\bar{b}\bar{t}b$ final state, showing the excellent physics capabilities of CLIC for studying heavy Higgs bosons.

**Fig. 60:**
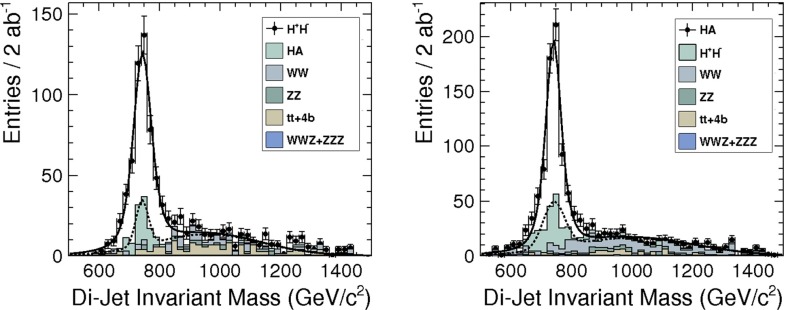
Di-jet invariant mass distributions for the $e^+e^-\rightarrow HA\rightarrow b\bar{b}b\bar{b}$ (*left*) and the $e^+e^-\rightarrow H^{+}H^{-}\rightarrow t\bar{b}\bar{t}b$ (*right*) signal together with the individual background contributions for model II [9, 10]

### Prospects for MSSM Higgs bosons^11^

We will briefly review the MSSM Higgs sector, the relevance of higher-order corrections and the implications of the recent discovery of a Higgs-like state at the LHC at $\sim 125\ \mathrm {GeV}\,$. Finally we look at the prospects in view of this discovery for MSSM Higgs physics at the LC. We will concentrate on the MSSM with real parameters.^12^ The NMSSM will be covered in Sect. 2.9.

#### The Higgs sector of the MSSM at tree level

Contrary to the SM, in the MSSM [216–218] two Higgs doublets are required (since the superpotential is a holomorphic function of the superfields). The Higgs potential

24 $$\begin{aligned} V= & {} m_{1}^2 |{\mathscr {H}}_{1}|^2 + m_{2}^2 |{\mathscr {H}}_{2}|^2 - m_{12}^2 (\epsilon _{ab} {\mathscr {H}}_{1}^a{\mathscr {H}}_{2}^b + \text{ h.c. }) \nonumber \\&+ \frac{1}{8}(g_1^2+g_2^2) \left[ |{\mathscr {H}}_{1}|^2 - |{\mathscr {H}}_{2}|^2 \right] ^2 + \frac{1}{2} g_2^2|{\mathscr {H}}_{1}^{\dag } {\mathscr {H}}_{2}|^2 , \end{aligned}$$

contains $m_1, m_2, m_{12}$ as soft SUSY-breaking parameters; $g_2$ and $g_1$ are the *SU*(2) and *U*(1) gauge couplings, respectively, and $\epsilon _{12} = -1$.

The doublet fields ${\mathscr {H}}_{1}$ and ${\mathscr {H}}_{2}$ are decomposed in the following way:

25 $$\begin{aligned} {\mathscr {H}}_{1}= & {} \left( \begin{array}{c}{\mathscr {H}}_{1}^0 \\ {\mathscr {H}}_{1}^- \end{array} \right) = \left( \begin{array}{c}\textstyle v_1 + \frac{1}{\sqrt{2}}(\phi _1^0 - i\chi _1^0) \\ -\phi _1^- \end{array} \right) , \nonumber \\ {\mathscr {H}}_{2}= & {} \left( \begin{array}{c}{\mathscr {H}}_{2}^+ \\ {\mathscr {H}}_{2}^0 \end{array} \right) = \left( \begin{array}{c}\phi _2^+ \\ \textstyle v_2 + \frac{1}{\sqrt{2}}(\phi _2^0 + i\chi _2^0) \end{array} \right) , \end{aligned}$$

where $\phi ^0_{1,2}$ denote the ${\mathscr {CP}}$-even fields, $\chi ^0_{1,2}$ the ${\mathscr {CP}}$-odd fields and $\phi ^\pm _{1,2}$ the charged field components. The potential (24) can be described with the help of two independent parameters (besides $g_2$ and $g_1$): $\tan \beta = v_2/v_1$ [with $v_1^2 + v_2^2 =: v^2 \approx (246\, \mathrm {GeV}\,)^2$] and $M_A^2 = -m_{12}^2(\tan \beta +\cot \beta )$, where $M_A$ is the mass of the ${\mathscr {CP}}$-odd Higg boson *A*.

The diagonalisation of the bilinear part of the Higgs potential, i.e. of the Higgs mass matrices, is performed via orthogonal transformations, introducing the mixing angle $\alpha$ for the ${\mathscr {CP}}$-even part (with $m_h$ denoting the tree-level value of the light ${\mathscr {CP}}$-even Higgs, see below),

26 $$\begin{aligned} \tan \,\alpha= & {} \left[ \frac{-(M_A^2 + M_Z^2) \sin \beta \cos \beta }{M_Z^2 \cos ^2\!\beta + M_A^2 \sin ^2\!\beta - m_h^2} \right] ,\nonumber \\&-\frac{\pi }{2} < \alpha < 0. \end{aligned}$$

One gets the following Higgs spectrum:

27 $$\begin{aligned}&\text{2 } \text{ neutral } \text{ bosons },\, {\mathscr {CP}} = +1 : h, H \nonumber \\&\text{1 } \text{ neutral } \text{ boson },\, {\mathscr {CP}} = -1 : A \nonumber \\&\text{2 } \text{ charged } \text{ bosons } : H^+, H^- \nonumber \\&\text{3 } \text{ unphysical } \text{ Goldstone } \text{ bosons } : G, G^+, G^- . \end{aligned}$$

At tree level the masses squares are given by

28 $$\begin{aligned} m^2_{H, h}= & {} \frac{1}{2} \bigg [ M_A^2 + M_Z^2 \nonumber \\&\pm \sqrt{(M_A^2 + M_Z^2)^2 - 4 M_Z^2 M_A^2 \cos ^2 2\beta } \bigg ] \end{aligned}$$

29 $$\begin{aligned} m_{H^\pm }^2= & {} M_A^2 + M_W^2. \end{aligned}$$

In the decoupling limit [219, 220], $M_A\gg M_Z$, the light ${\mathscr {CP}}$-even Higgs becomes SM-like, i.e. all its couplings approach their SM value.

#### The relevance of higher-order corrections

Higher-order corrections give large contributions to the Higgs sector predictions in the MSSM [221, 222]. Most prominently, they affect the prediction of the Higgs-boson masses in terms of the other model parameters. In the MSSM, in particular, the light ${\mathscr {CP}}$-even Higgs-boson mass receives higher-order contributions up to ${\mathscr {O}}(100~\%)$ [223–225]. The very leading one-loop correction reads

30 $$\begin{aligned} {\varDelta }M_h^2 = \frac{3\, g_2^2\, m_{t}\,^4}{8 \,\pi ^2\,M_W^2} \, \left[ \log \left( \frac{M_S^2}{m_{t}\,^2} \right) + \frac{X_t^2}{M_S^2} \left( 1 - \frac{X_t^2}{12\,M_S^2} \right) \right] , \end{aligned}$$

where $M_S = (m_{\tilde{t}_1}+ m_{\tilde{t}_2})/2$ denotes the average of the two scalar top masses, and $m_{t}\,X_t$ is the off-diagonal element in the scalar top mass matrix. Via this kind of higher-order corrections the light Higgs mass is connected to all other sectors of the model and can serve as a precision observable. The missing higher-order uncertainties have been estimated to be at the level of $\sim$2–3 GeV [226, 227].

Higher-order corrections also affect the various couplings of the Higgs bosons and thus the production cross sections and branching ratios. Focusing on the light ${\mathscr {CP}}$-even Higgs boson, the couplings to down-type fermions are modified with respect to the SM coupling by an additional factor $-\sin \alpha /\cos \beta$, and higher-order corrections can be absorbed into the ${\mathscr {CP}}$-even mixing angle, $\alpha \rightarrow \alpha _\mathrm{eff}$ [228]. For large higher-order corrections which drive $\alpha _\mathrm{eff}\rightarrow 0$ the decay widths ${\varGamma }(h \rightarrow b \bar{b})$ and ${\varGamma }(h \rightarrow \tau ^+\tau ^-)$ could be substantially smaller than in the SM [229], altering the available search modes for such a Higgs boson.

The relation between the bottom-quark mass and the Yukawa coupling $h_b$, which controls also the interaction between the Higgs fields and the sbottom quarks, is also affected by higher-order corrections, summarised in the quantity ${\varDelta }_b$ [230–234]. These, often called threshold corrections, are generated either by gluino–sbottom one-loop diagrams [resulting in ${\mathscr {O}}(\alpha _b\alpha _s)$ corrections], or by chargino–stop loops [giving ${\mathscr {O}}(\alpha _b\alpha _t)$ corrections]. Analytically one finds ${\varDelta }_b\propto \mu \tan \beta$. The effective Lagrangian is given by [233].

$$\begin{aligned}&{\mathscr {L}}= \frac{g_2}{2M_W} \frac{\overline{m}_b}{1 + {\varDelta }_b} \Bigg [ \tan \beta \; A \, i \, \bar{b} \gamma _5 b + \sqrt{2}\, V_{tb} \, \tan \beta \; H^+ \bar{t}_L b_R \\&\qquad + \left( \frac{\sin \alpha }{\cos \beta } - {\varDelta }_b\frac{\cos \alpha }{\sin \beta } \right) h \bar{b}_L b_R - \left( \frac{\cos \alpha }{\cos \beta } + {\varDelta }_b\frac{\sin \alpha }{\sin \beta } \right) H \bar{b}_L b_R \Bigg ]\\&\qquad +\,\mathrm{h.c.} \end{aligned}$$

Large positive (negative) values of ${\varDelta }_b$ lead to a strong suppression (enhancement) of the bottom Yukawa coupling. For large $M_A$ the decoupling of the light ${\mathscr {CP}}$-even Higgs boson to the SM bottom Yukawa coupling is ensured in Eq. (31). Effects on the searches for heavy MSSM Higgs bosons via ${\varDelta }_b$ have been analysed in Refs. [235, 236].

Deviations from the SM predictions can also be induced by the appearance of light virtual SUSY particles in loop-induced processes. Most promiently a light scalar top can have a strong impact on the prediction of $gg \rightarrow h$. The additional contributions can interfere negatively with the top loop contribution, leading to a strong suppression of the production cross section [229, 237, 238]. Similarly, it was shown that light scalar taus can lead to an enhancement of up to $\sim$50 % of the decay width of the light ${\mathscr {CP}}$-even Higgs to photons, ${\varGamma }(h \rightarrow \gamma \gamma )$ [239, 240].

#### Implicatios of the discovery at $\sim$125 GeV

The discovery of a new state with a mass around $M_H\simeq 125\ \mathrm {GeV}\,$, which has been announced by ATLAS [241] and CMS [242], marks a milestone of an effort that has been on-going for almost half a century and opens a new era of particle physics. Both ATLAS and CMS reported a clear excess around $\sim$125 GeV in the two photon channel as well as in the $ZZ^{(*)}$ channel, supported by data in the $WW^{(*)}$ channel. The combined sensitivity in each of the experiments reaches by now far beyond $5 \sigma$. Also the final Tevatron results [243] show a broad excess in the region around $M_H\sim 125 \, \mathrm {GeV}\,$ that reaches a significance of nearly $3\,\sigma$. Within theoretical and experimental uncertainties the newly observed boson behaves SM-like [244–247]. Several types of investigations have analysed the compatibility of the newly observed state around $\sim$125 GeV with the MSSM.

**Fig. 61:**
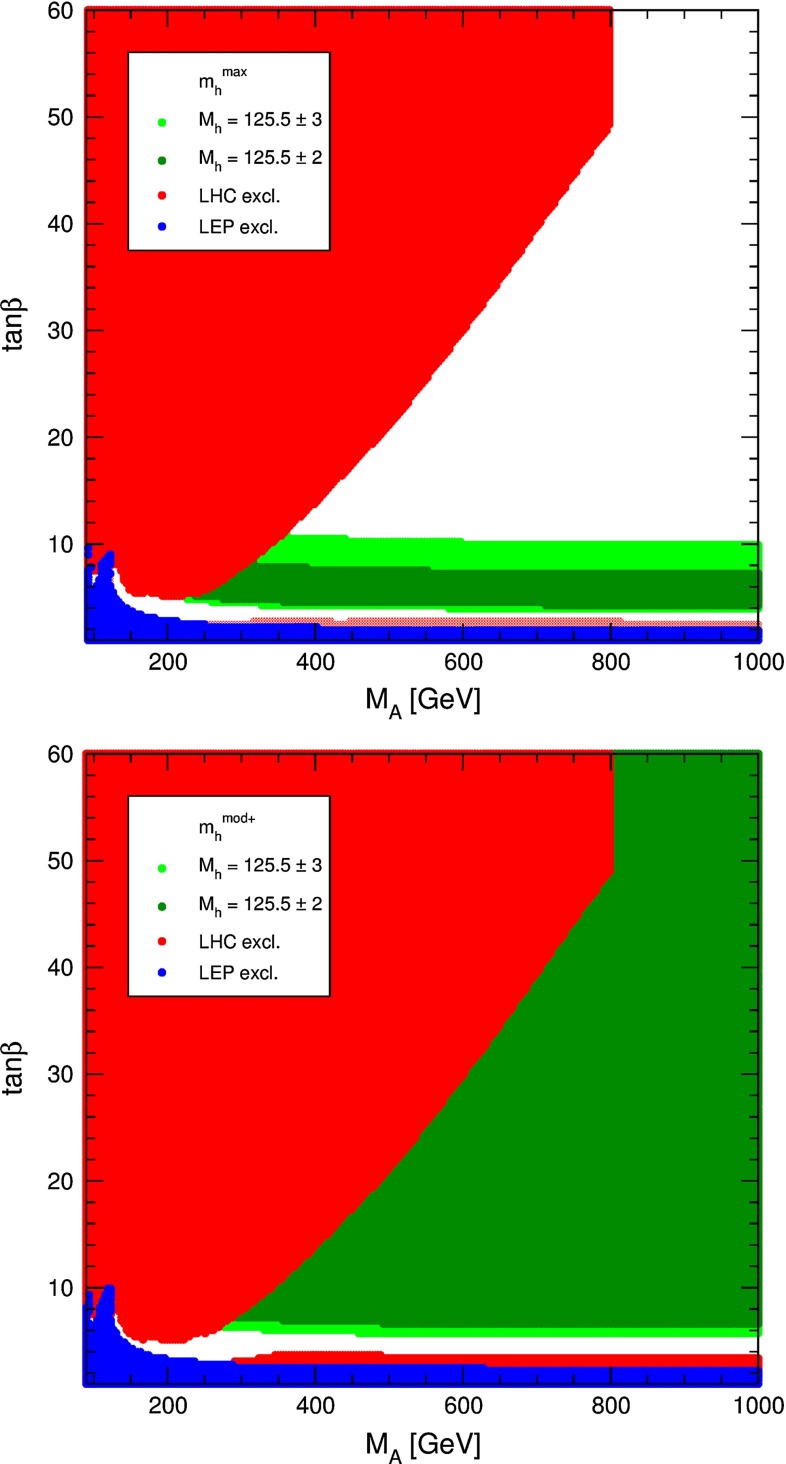
$M_A$–$\tan \beta$ plane in the $m_h^{\max }$ scenario (*upper*) and in the $m_h^\mathrm{mod+}$ scenario (*lower plot*) [238]. The *green-shaded area* yields $M_h\sim 125 \pm 3 \,\mathrm {GeV}\,$, the *red area* at high $\tan \beta$ is excluded by LHC heavy MSSM Higgs-boson searches, the *blue area* is excluded by LEP Higgs searches, and the *red strip* at *low* $\tan \beta$ is excluded by the LHC SM Higgs searches
  In the lower plot of Fig. 61 we show the $m_h^\mathrm{mod+}$ scenario that differs from the $m_h^{\max }$ scenario in the choice of $X_t$. While in the $m_h^{\max }$ scenario $X_t/M_\mathrm{SUSY} = +2$ had been chosen to maximise $M_h$, in the $m_h^\mathrm{mod+}$ scenario $X_t/M_\mathrm{SUSY} = +1.5$ is used to yield a “good” $M_h$ value over the nearly the entire $M_A$–$\tan \beta$ plane, which is visible as the extended green region.

- Looking into pre-defined benchmark scenarios it was shown that the light ${\mathscr {CP}}$-even Higgs boson can be interpreted as the new boson around $125\ \mathrm {GeV}\,$. On the other hand, also the heavy ${\mathscr {CP}}$-even Higgs boson can in principle be interpreted as the newly discovered state [248]. The latter option, however, is challenged by the latest ATLAS results on charged Higgs-boson searches [249].
  Here we briefly discuss the results in two of the new benchmark scenarios [238], devised for the search for heavy MSSM Higgs bosons. In the upper plot of Fig. 61 the $m_h^{\max }$ scenario is shown. The red area is excluded by LHC searches for the heavy MSSM Higgs bosons, the blue area is excluded by LEP Higgs searches, and the light shaded red area is excluded by LHC searches for a SM-like Higgs boson. The bounds have been obtained with HiggsBounds [250–252] (where an extensive list of original references can be found). The green area yields $M_h= 125 \pm 3$ GeV, i.e. the region allowed by the experimental data, taking into account the theoretical uncertainty in the $M_h$ calculation as discussed above. The left plot also allows one to extract new *lower* limits on $M_A$ and $\tan \beta$. From this analysis it can be concluded that if the light ${\mathscr {CP}}$-even Higgs is interpreted as the newly discovered state at $\sim$125 GeV, then $\tan \beta \gtrsim 4$, $M_A\gtrsim 200 \, \mathrm {GeV}\,$ and $M_{H^\pm }\gtrsim 220 \, \mathrm {GeV}\,$ [238].

**Fig. 62:**
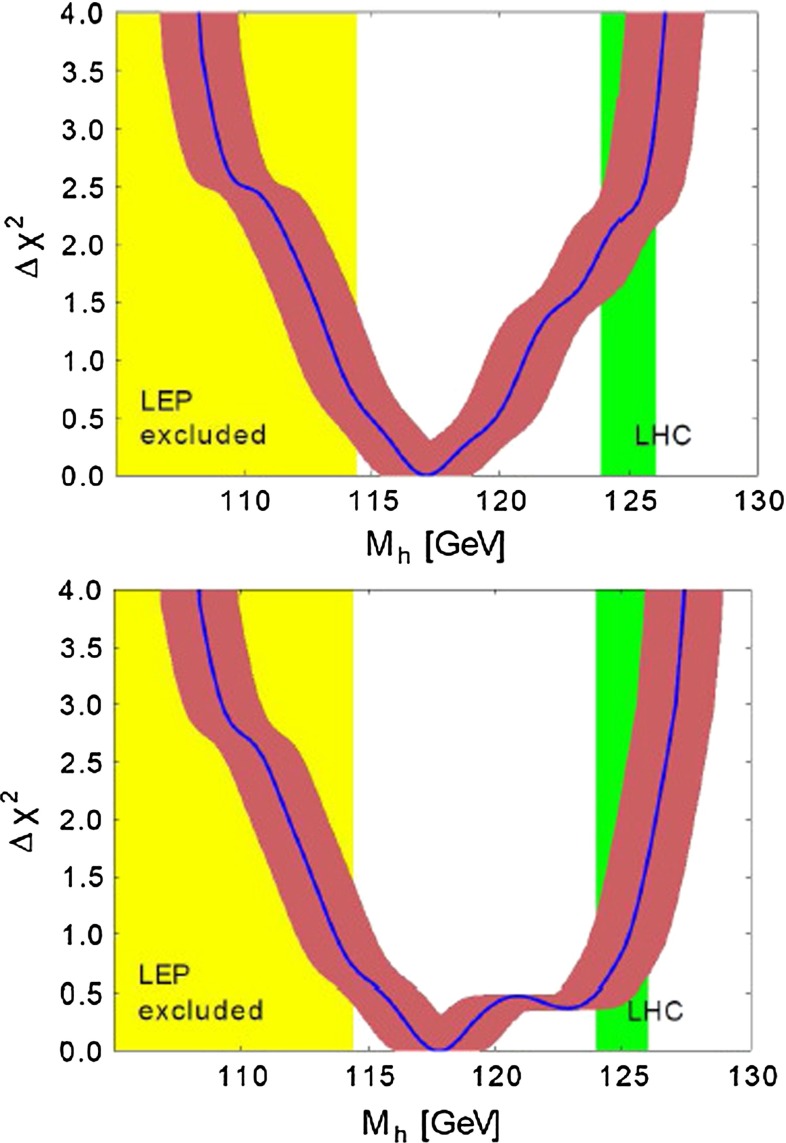
Fit for the light ${\mathscr {CP}}$-even Higgs mass in the CMSSM (*left*) and NUHM1 (*right*) [254]. Direct searches for the light Higgs boson are *not* included
- In GUT based scenarios such as the CMSSM and the NUHM1^13^  it was shown that a light ${\mathscr {CP}}$-even Higgs boson around or slightly below $125\ \mathrm {GeV}\,$ is a natural prediction of these models [254]. These predictions take into account the current SUSY search limits (but no direct light Higgs search limits), as well as the relevant EWPO, *B*-physics observables and the relic Dark Matter density. In Fig. 62 we show the predictions in the CMSSM (upper) and the NUHM1 (lower plot). The red bands indicate a theory uncertainty of $\sim$1.5 GeV on the evaluation of $M_h$. The green columns indicate the range of the newly discovered particle mass.
- Parameter scans in the MSSM with 19 free parameters (pMSSM–19 [253]) are naturally compatible with a light Higgs boson around $M_h\sim 125\ \mathrm {GeV}\,$, as has been analysed in Refs. [255, 256] (see also Ref. [257] for a more recent analysis in the pMSSM–15 and Ref. [258] for an analysis in the pMSSM–19). Taking into account the available constraints from SUSY searches, Higgs searches, low-energy observables, *B*-physics observables and the relic abundance of Dark Matter viable scenarios can be identified that can be analysed in the upcoming LHC runs. Also the effects on the various production cross sections and branching ratios were analysed, where it was confirmed that light particles can modify in particular the decay rate to photons [239, 240].

**Fig. 63:**
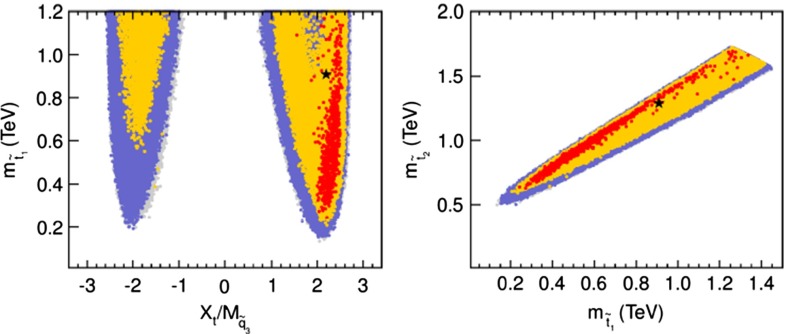
Stop mixing parameter $X_t/m_{\tilde{q}_3}$ vs. the light stop mass (*left*), and the light vs. heavy stop masses (*right*), see text
- Parameter scans in the MSSM with seven free parameters (pMSSM–7) in comparison to the pMSSM–19 have the advantage of a full sampling of the parameter space with ${\mathscr {O}}(10^7)$ points; but they have the disadvantage of potentially not including all relevant phenomenogy of the MSSM. The pMSSM–7 fits to the full set of Higgs data (and several low-energy observables) [259] allow one to show an enhancement of the $\mathrm{BR}(h \rightarrow \gamma \gamma )$, correlated to a suppression of the decays to $b \bar{b}$ and $\tau ^+\tau ^-$ via the mechanisms outlined in Sect. 2.5.2 (see also Ref. [260]). In particular, these scans (while not incorporating the latest data) demonstrate that light scalar top masses are compatible with $M_h\sim 125\ \mathrm {GeV}\,$ (see also Ref. [248]). In Fig. 63 we show $X_t/m_{\tilde{q}_3}$ vs. the light stop mass (left plot, where $X_t= A_t- \mu /\tan \beta$ denotes the off-diagonal entry in the scalar top mass matrix, $A_t$ is the tri-linear Higgs-stop coupling, and $m_{\tilde{q}_3}$ denotes the (common) diagonal soft SUSY-breaking parameter in the scalar top and bottom sector) and the light vs. the heavy stop mass (right plot) in the case that the light ${\mathscr {CP}}$-even Higgs boson corresponds to the new state at $\sim$125 GeV. The coloured points passed the Higgs exclusion bounds (obtained using HiggsBounds [250–252]). The red (yellow) points correspond to the best-fit points with a ${\varDelta }\chi ^2 < 2.3 (5.99)$, see Ref. [259] for details. In the left plot one can see that the case of zero stop mixing in the MSSM is excluded by the observation of a light Higgs at $M_h\sim 125\ \mathrm {GeV}\,$ (unless $m_{\tilde{q}_3}$ is extremely large, see, e.g., Ref. [261]), and that values of $|X_t/m_{\tilde{q}_3}|$ between $\sim$1 and $\sim$2.5 must be realised. For the most favoured region we find $X_t/m_{\tilde{q}_3}= 2$– 2.5. Concerning the value of the lightest scalar top mass, the overall smallest values are found at $m_{\tilde{t}_1}\sim 200 \, \mathrm {GeV}\,$, where also the regions favoured by the fit to the Higgs rates start, in the case of $X_t$ positive. Such a light $\tilde{t}_{1}$ is accompanied by a somewhat heavier $\tilde{t}_{2}$, as can be seen in the right plot of Fig. 63. Values of $m_{\tilde{t}_1}\sim 200 \mathrm {GeV}\,$ are realised for $m_{\tilde{t}_2}\sim 600 \mathrm {GeV}\,$, which would mean that both stop masses are rather light, offering interesting possibilities for the LHC. The highest favoured $m_{\tilde{t}_1}$ values we find are $\sim$1.4 TeV. These are the maximal values reached in the scan in Ref. [259], but from Fig. 63 it is obvious that the favoured region extends to larger values of both stop masses. Such a scenario would be extremely difficult to access at the LHC.

Searches for the other Higgs bosons of the MSSM have so far not been successful. This applies to the heavy Higgs bosons of the MSSM as well as to a potentially light ${\mathscr {CP}}$-even Higgs bosons in the MSSM in the case that the new state at $\sim$125 GeV is interpreted as the heavy ${\mathscr {CP}}$-even Higgs boson, see Sect. 2.2.

#### Prospects for the MSSM Higgs bosons at the LHC

The prime task now is to study the properties of the discovered new particle and in particular to test whether the new particle is compatible with the Higgs boson of the SM or whether there are significant deviations from the SM predictions, which would point towards physics beyond the SM. A large part of the current and future LHC physics programme is devoted to this kind of analyses.

The prospects for the SM Higgs boson in this respect are the following [262–264]:

- The Higgs-boson mass can be determined down to a level of ${\mathscr {O}}(200\, \mathrm {MeV}\,)$.
- For the coupling determination the following has to be kept in mind. Since it is not possible to measure the Higgs production cross sections independently from the Higgs decay (or, equivalently, the Higgs boson width^14^), a determination of couplings is only possible if certain (theory) assumptions on the Higgs width are made, see, e.g. Ref. [196, 266]. For instance, it can be assumed that no new particles contribute to the decay width. Under this kind of assumption, going to the HL-LHC, precisions on couplings at the $\sim$10 % level can be achieved. Without any assumptions only ratios of couplings can be determined (see also Ref. [78] for a recent review).
- Studies in the context of the HL-LHC indicate that there might be some sensitivity on the tri-linear Higgs self-coupling; however, this will require a careful estimate of background contributions. Further studies to clarify these issues are currently in progress, see Ref. [267] for a discussion.
- It can be expected that the spin 2 hypothesis can be rejected using LHC data.
- A pure ${\mathscr {CP}}$-even state can be discarded at the $2\,\sigma$ level already from current data (assuming that the coupling strength to gauge bosons is the same one as in the ${\mathscr {CP}}$-even case). However, the prospects for the LHC to determine a certain level of ${\mathscr {CP}}$-odd admixture to the Higgs state are less clear [268].

In the case that the light ${\mathscr {CP}}$-even MSSM Higgs boson is identified with the new state at $\sim$125 GeV, as can be seen in Fig. 61, the decoupling region, $M_A\gg M_Z$ is a viable option. In this case the SM Higgs analyses can be taken over directly to the MSSM case – and will yield (nearly) identical results. Only light SUSY particles in the loops mediating the gluon fusion process or the decay to two photons might result in somewhat different predictions. However, depending on the actual values of the SUSY mass scales, these differences might easily remain unobservable with the anticipated LHC precision. Furthermore, in the decoupling regime the heavy MSSM Higgs bosons can easily be too heavy to be discovered at the LHC, in particular for medium or lower values of $\tan \beta$.

Only in the lower allowed range for $M_A$ in this scenario larger deviations from the phenomenology of the light ${\mathscr {CP}}$-even MSSM Higgs with respect to the SM Higgs can be expected. Depending on the level of decoupling, the LHC might be able to detect this kind of deviations, e.g. in enhanced rates involving the decay to two photons or in suppressed rates in the decay to $\tau$ leptons or *b* quarks.

#### Prospects for the MSSM Higgs bosons at the LC

As outlined in the previous subsection, identifying the light ${\mathscr {CP}}$-even Higgs with the new state at $\sim$125 GeV can easily result in a scenario where the LHC can neither distinguish the *h* from the SM Higgs boson, nor be able to discover additional Higgs bosons. In this case the analyses at an LC offer good prospects to reveal the non-SM nature of the Higgs particle. The anticipated experimental precisions for couplings to SM particles, the self-coupling etc., as given in detail in Sect. 2.3. In particular, the following improvements over the anticipated LHC precision/potential can be expected:

- The mass of a SM-like Higgs boson at $\sim$125 GeV can be determined at the level of $50 \mathrm {MeV}\,$.
- Using the *Z* recoil method the production cross section of a SM-like Higgs can be determined independently of the decay products, see Sect. 2.3. This allows for a *model-independent* measurement of the Higgs couplings at the per-cent level; see Table 18. In particular, a determination of the tri-linear Higgs self-coupling at the level of 15 % can be expected.
- The spin can be determined unambiguously from a production cross section threshold scan.
- The ${\mathscr {CP}}$ decomposition can be determined, in particular, using the channel $e^+e^- \rightarrow t \bar{t} H$ [270, 271].
- The reach for the heavy Higgs bosons can be extended to higher masses in particular for lower and intermediate values of $\tan \beta$ up to $M_A\lesssim \sqrt{s}/2$ (and possibly beyond, depending on the SUSY parameters [272]).
  An indirect determination of $M_A$ can be performed via a precise measurement of the Higgs couplings, where a sensitivity up to $800 \mathrm {GeV}\,$ was found [273].
  In the $\gamma \gamma$ option of the LC the Higgs bosons can be produced in the *s*-channel, and a reach up to $M_A\lesssim 0.8 \sqrt{s}$ can be realised [274] (see also Refs. [275, 276]).

Another measurement at the LC can turn out to be crucial for Higgs physics in the MSSM: the determination of $m_{t}\,$ from a threshold scan. As can be seen in Eq. (30), the theory prediction of $M_h$ depends strongly on $m_{t}\,$. Only the LC determination of a well-defined top-quark mass can yield a theory prediction that matches the LHC precision in $M_h$. More details can be found in Sect. 4.4.

### General multi-Higgs structures^15^

#### Introduction

We here give a review of extended Higgs sectors and their collider phenomenology. In the SM, one isospin doublet scalar field $\Phi$ is simply introduced as the minimum form. Under the requirement of the renormalisability its potential can be uniquely written as

31 $$\begin{aligned} V(\Phi ) = + \mu ^2 |\Phi |^2 + \lambda |\Phi |^4. \end{aligned}$$

**Table 18:** Examples of the precision of SM-like Higgs observables at a $\sqrt{s}=500 \mathrm {GeV}\,$ LC assuming a Higgs-boson mass of $125 \, \mathrm {GeV}\,$. The results are based on the ILC set-up. For the direct measurements, an integrated luminosity of ${\mathscr {L}}^\mathrm{int} = 500~\mathrm {fb}^{-1}$ is assumed. For the indirect measurements at GigaZ, a running time of approximately one year is assumed, corresponding to ${\mathscr {L}}=$ ${\mathscr {O}}(10~\mathrm {fb}^{-1})$. Taken from Ref. [269]

Observable	Expected precision (%)
$M_H$ (GeV)	0.03
$$g_{HWW} $$	1.4
$$g_{HZZ} $$	1.4
$$g_{Hbb} $$	1.4
$$g_{Hcc} $$	2.0
$g_{H\tau \tau }$	2.5
$$g_{Htt} $$	10
$$g_{HHH} $$	40
$\mathrm{BR}\, (H \rightarrow \gamma \gamma )$	25
$\mathrm{BR}\, (H \rightarrow gg)$	5
$\mathrm{BR}\, (H \rightarrow \mathrm {invisible})$	0.5

By putting an assumption of $\mu ^2 < 0$ (and $\lambda > 0$), the shape of the potential becomes like a Mexican hat, and the electroweak symmetry is broken spontaneously at the vacuum $\langle \Phi \rangle = (0, v/\sqrt{2})^T$. Consequently, weak gauge bosons, quarks and charged leptons obtain their masses from the unique vacuum expectation value (VEV) *v* ($=(\sqrt{2}G_F)^{-1/2} \simeq 246$ GeV). However, there is no theoretical principle for the SM Higgs sector, and there are many possibilities for non-minimal Higgs sectors. While the current LHC data do not contradict the predictions of the SM, most of the extended Higgs sectors can also satisfy current data. These extended Higgs sectors are often introduced to provide physics sources to solve problems beyond the SM, such as baryogenesis, DM and tiny neutrino masses. Each scenario can predict a specific Higgs sector with additional scalars.

It is also known that the introduction of the elementary scalar field is problematic from the theoretical viewpoint, predicting the quadratic divergence in the radiative correction to the mass of the Higgs boson. Such a quadratic divergence causes the hierarchy problem. There are many scenarios proposed to solve the hierarchy problem such as supersymmetry, dynamical symmetry breaking, Extra dimensions and so on. Many models based on these new paradigms predict specific Higgs sectors in their low-energy effective theories.

Therefore, experimental determination of the structure of the Higgs sector is essentially important to deeply understand EWSB and also to find direction to new physics beyond the SM. The discovery of the 125-GeV Higgs boson at the LHC in 2012 is a big step to experimentally investigate the structure of the Higgs sector. From the detailed study of the Higgs sector, we can determine the model of new physics.

What kind of extended Higgs sectors can we consider? As the SM Higgs sector does not contradict the current data within the errors, there should be at least one isospin doublet field which looks like the SM Higgs boson. An extended Higgs sector can then contain additional isospin multiplets. There can be infinite kinds of extended Higgs sectors. These extended Higgs sectors are subject to constraints from the current data of many experiments including those of the electroweak $\rho$-parameter and for flavour changing neutral currents (FCNCs).

The electroweak $\rho$-parameter is calculated at the tree level for a Higgs sector with *N* multiplets by

32 $$\begin{aligned} \rho = \frac{m_W^2}{m_Z^2 \cos ^2\theta _W} = \frac{\sum _i \left\{ 4 T_i (T_i+1)- Y_i^2 \right\} |v_i|^2 c_i}{\sum _i 2 Y_i^2 |v_i|^2}, \end{aligned}$$

where $T_i$ and $Y_i$ ($i=1, \ldots , N$) are isospin and hypercharges of the *i*th multiplet field ($Q_i=T_i+Y_i/2$), and $c_i =1/2$ for real fields ($Y_i=0$) and 1 for complex fields. The data shows that $\rho =1.0004^{+0.0003}_{-0.0004}$ [277]. Higgs sectors with additional doublets $(T_i, Y_i) = (1/2, 1)$ (and singlets with $Y_i=0$) predict $\rho =1$ at the tree level, like the SM Higgs sector. Thus, multi-doublet structures would be a *natural* extension of the Higgs sector. The introduction of higher representation fields generally causes a tree-level deviation in the $\rho$- parameter from unity. For example, in the model with a triplet field ${\varDelta }$(1, 2) with the VEV $v_{\varDelta }$, $\rho \sim 1 - 2(v_{\varDelta }/v)^2$ is given, so that in such a model a tuning $(v_{\varDelta }/v)^2 \ll 1$ is required to satisfy the data. We note that there are exceptional Higgs sectors with larger isospin representations which predict $\rho =1$ at the tree level. In the model proposed by Georgi and Machacek [278], the Higgs sector is composed of an isospin doublet field with additional a complex (1, 2) and a real (1, 0) triplet fields, which satisfies $\rho =1$ at the tree level. Addition of the septet field (3, 2) to the SM Higgs sector also predicts $\rho =1$ at the tree level.

Extended Higgs sectors with a multi-doublet structure, in general, receive a severe constraint from the results of FCNC experiments. The data show that FCNC processes such as $K^0 \rightarrow \mu ^+\mu ^-$, $B^0-\bar{B}^0$ and so on are highly suppressed [277]. In the SM with a doublet Higgs field, the suppression of FCNC processes is perfectly explained by the so-called Glashow–Illiopoulos–Miani mechanism [279]. On the other hand, in general multi Higgs-doublet models where multiple Higgs doublets couple to a quark or a charged lepton, Higgs boson-mediated FCNC processes can easily occur at the tree level. In these models, in order to avoid such dangerous FCNC processes, it is required that these Higgs-doublet fields have different quantum numbers [280].

In Sect. 2.6.2, we discuss properties of the two Higgs-doublet model (2HDM), and its phenomenology at the LHC and the ILC. The physics of the model with the Higgs sector with a triplet is discussed in Sect. 2.6.3. The possibilities of more exotic extended Higgs sectors are briefly discussed in Sect. 2.6.4.

#### Two Higgs-doublet models

The 2HDM is one of the simplest extensions of the standard Higgs sector with one scalar doublet field. The model has many typical characteristics of general extended Higgs sectors, such as the existence of additional neutral Higgs states, charged scalar states, and the source of CP violation. In fact, the 2HDM often appears in the low-energy effective theory of various new physics models which try to solve problems in the SM such as the minimal supersymmetric SM (MSSM), to some models of neutrino masses, DM, and electrowak baryogenesis. Therefore, it is useful to study properties of 2HDMs with their collider phenomenology.

In the 2HDM, two isospin doublet scalar fields $\Phi _1$ and $\Phi _2$ are introduced with a hypercharge $Y=1$. The Higgs potential under the standard gauge symmetry is given by [86]

33 $$\begin{aligned} V= & {} m_1^2|\Phi _1|^2+m_2^2|\Phi _2|^2 - (m_3^2 \Phi _1^{\dagger }\Phi _2 + \mathrm{h.c.} ) \nonumber \\&+ \frac{\lambda _1}{2}|\Phi _1|^4 +\frac{\lambda _2}{2}|\Phi _2|^4 + \lambda _3|\Phi _1|^2|\Phi _2|^2+\lambda _4|\Phi _1^\dagger \Phi _2|^2 \nonumber \\&+ \Bigg [\frac{\lambda _5}{2}(\Phi _1^\dagger \Phi _2)^2 + \left\{ \lambda _6(\Phi _1^\dagger \Phi _1) + \lambda _7(\Phi _2^\dagger \Phi _2)\right\} \Phi ^\dagger _1\Phi _2\nonumber \\&+\, \mathrm{h.c.} \Bigg ], \end{aligned}$$

where $m_1^2$, $m_2^2$ and $\lambda _{1-4}$ are real, while $m_3^2$ and $\lambda _{5-7}$ are complex. We here discuss the case of ${\textit{CP}}$ conservation with taking these complex as real. The doublet fields can be parameterised as

34 $$\begin{aligned} \Phi _i=\left[ \begin{array}{l} w_i^+\\ \textstyle \frac{1}{\sqrt{2}}(v_i+h_i+iz_i) \end{array}\right] , \quad (i=1,2), \end{aligned}$$

where $v_1$ and $v_2$ are the VEVs of $\Phi _1$ and $\Phi _2$, which satisfy $v\equiv \sqrt{v_1^2+v_2^2}$. The ratio of the two VEVs is a parameter written as $\tan \beta =v_2/v_1$. The mass eigenstates for the scalar bosons are obtained by

35 $$\begin{aligned} \left( \begin{array}{c} w_1^\pm \\ w_2^\pm \end{array}\right)= & {} R(\beta ) \left( \begin{array}{c} G^\pm \\ H^\pm \end{array}\right) ,\quad \left( \begin{array}{c} z_1\\ z_2 \end{array}\right) =R(\beta )\left( \begin{array}{c} G^0\\ A \end{array}\right) , \nonumber \\ \left( \begin{array}{c} h_1\\ h_2 \end{array}\right)= & {} R(\alpha ) \left( \begin{array}{c} H\\ h \end{array}\right) , \quad \text {with}~R(\theta ) = \left( \begin{array}{c@{\quad }c} \cos \theta &{} -\sin \theta \\ \sin \theta &{} \cos \theta \end{array}\right) , \end{aligned}$$

where $G^\pm$ and $G^0$ are the Nambu–Goldstone bosons absorbed by the longitudinal component of $W^\pm$ and *Z*, respectively. As the physical degrees of freedom, consequently, we have two ${\textit{CP}}$-even Higgs bosons *h* and *H*, a ${\textit{CP}}$-odd Higgs boson *A* and a pair of singly charged Higgs boson $H^\pm$. We define *h* as the SM-like Higgs boson with the mass of about 125 GeV.

As already mentioned, in general 2HDMs, FCNCs can appear via tree-level Higgs-mediated diagrams, which are not phenomenologically acceptable. The simple way to avoid such dangerous FCNCs is to impose a discrete $Z_2$ symmetry, under which the two doublets are transformed as $\Phi _1\rightarrow +\Phi _1$ and $\Phi _2\rightarrow -\Phi _2$ [280–283]. Then each quark or lepton can couple with only one of the two doublets, so that the Higgs-mediated FCNC processes are forbidden at the tree level.

We hereafter concentrate on the case with the discrete symmetry. Under this symmetry, $\lambda _6$ and $\lambda _7$ in the Higgs potential in Eq. (33) are zero. On the other hand, the soft-breaking mass $m_3^2$ of the discrete symmetry can be allowed, because the discrete symmetry is introduced just to suppress FCNC interactions. As we consider the ${\textit{CP}}$-conserving scenario, $m_3^2$ and $\lambda _5$ are real. Eight parameters in the potential are rewritten as the following eight physical parameters; the masses of *h*, *H*, *A* and $H^\pm$, two mixing angles $\alpha$ and $\beta$ appearing in Eq. (35), the VEV *v* and the soft-breaking parameter $M^2$ defined by

36 $$\begin{aligned} M^2=\frac{m_3^2}{\sin \beta \cos \beta }. \end{aligned}$$

In terms of these parameters, the quartic coupling constants in the Higgs potential are expressed as [284]

37a $$\begin{aligned} \lambda _1= & {} \frac{1}{v^2\cos ^2\beta } (-M^2\sin ^2\beta +m_h^2\sin ^2\alpha +m_H^2\cos ^2\alpha ), \end{aligned}$$

37b $$\begin{aligned} \lambda _2= & {} \frac{1}{v^2\sin ^2\beta } (-M^2\cos ^2\beta +m_h^2\cos ^2\alpha +m_H^2\sin ^2\alpha ), \end{aligned}$$

37c $$\begin{aligned} \lambda _3= & {} \frac{1}{v^2}\left[ -M^2 -\frac{\sin 2\alpha }{\sin 2\beta }(m_h^2-m_H^2)+2m_{H^{\pm }}^2\right] ,\end{aligned}$$

37d $$\begin{aligned} \lambda _4= & {} \frac{1}{v^2}(M^2+m_A^2-2m_{H^{\pm }}^2),\end{aligned}$$

37e $$\begin{aligned} \lambda _5= & {} \frac{1}{v^2}(M^2-m_A^2). \end{aligned}$$

Under the softly broken discrete symmetry, the Yukawa interactions of the 2HDM can be written as

38 $$\begin{aligned} {\mathscr {L}}^\mathrm{2HDM}_\mathrm{Yukawa}= & {} -\bar{Q}_{L}Y_u\tilde{\Phi }_u u_R -\bar{Q}_{L}Y_d\Phi _d d_R \nonumber \\&-\, \bar{L}_{L}Y_{\ell }\Phi _{\ell }\ell _R +\mathrm{h.c.}, \end{aligned}$$

where *R* and *L* are the right-handed and left-handed chirality of fermions, respectively, and $\Phi _{f=u,d,\ell }$ are chosen from $\Phi _1$ or $\Phi _2$. There are four types of Yukawa interactions depending on the parity assignment of the discrete symmetry for fermions [285] shown in Table 19. Type-I is the case that all the quarks and charged leptons obtain the masses from $v_2$, while Type-II is that up-type quark masses are generated by $v_2$ but the masses of down-type quarks and charged leptons are generated by $v_1$. In Type-X, both up- and down- type quarks couple to $\Phi _2$, while charged leptons couple to $\Phi _1$. In Type-Y, up-type quarks and charged leptons couple to $\Phi _2$, while up-type quarks couple to $\Phi _1$. Because of these variations in types of Yukawa interaction, the 2HDM with the discrete symmetry can provide rich phenomenology. We note that Type-I is for example used in the neutrino-philic mode [286] approximately, Type-II is predicted in the context of the minimal supersymmetric SM (MSSM) [86, 217] and that Type-X is used for example in some of radiative seesaw models [287–289].

**Table 19:** Four possible $Z_2$ charge assignments of scalar and fermion fields to forbid tree-level Higgs-mediated FCNCs [146]

	$\Phi _1$	$\Phi _2$	$u_R$	$d_R$	$\ell _R$	$Q_L$	$L_L$
Type-I	$+$	$-$	$-$	$-$	$-$	$+$	$+$
Type-II	$$+ $$	$$-$$	$$-$$	$$+$$	$$+$$	$$+$$	+
Type-X	$+$	$-$	$-$	$-$	$+$	$+$	$+$
Type-Y	$+$	$-$	$-$	$+$	$-$	$+$	$+$

**Table 20:** The coefficients for different type of Yukawa interactions [146]. $c_\theta =\cos \theta ,~\mathrm{and }~s_\theta =\sin \theta$ for $\theta = \alpha ,~\beta$

	$\xi _h^u$	$\xi _h^d$	$\xi _h^\ell$	$\xi _H^u$	$\xi _H^d$	$\xi _H^\ell$	$\xi _A^u$	$\xi _A^d$	$\xi _A^\ell$
Type-I	$c_\alpha /s_\beta$	$c_\alpha /s_\beta$	$c_\alpha /s_\beta$	$s_\alpha /s_\beta$	$s_\alpha /s_\beta$	$s_\alpha /s_\beta$	$\cot \beta$	$-\cot \beta$	$-\cot \beta$
Type-II	$c_\alpha /s_\beta$	$-s_\alpha /c_\beta$	$-s_\alpha /c_\beta$	$s_\alpha /s_\beta$	$c_\alpha /c_\beta$	$c_\alpha /c_\beta$	$\cot \beta$	$\tan \beta$	$\tan \beta$
Type-X	$c_\alpha /s_\beta$	$c_\alpha /s_\beta$	$-s_\alpha /c_\beta$	$s_\alpha /s_\beta$	$s_\alpha /s_\beta$	$c_\alpha /c_\beta$	$\cot \beta$	$-\cot \beta$	$\tan \beta$
Type-Y	$c_\alpha /s_\beta$	$-s_\alpha /c_\beta$	$c_\alpha /s_\beta$	$s_\alpha /s_\beta$	$c_\alpha /c_\beta$	$s_\alpha /s_\beta$	$\cot \beta$	$\tan \beta$	$-\cot \beta$

**Fig. 64:**
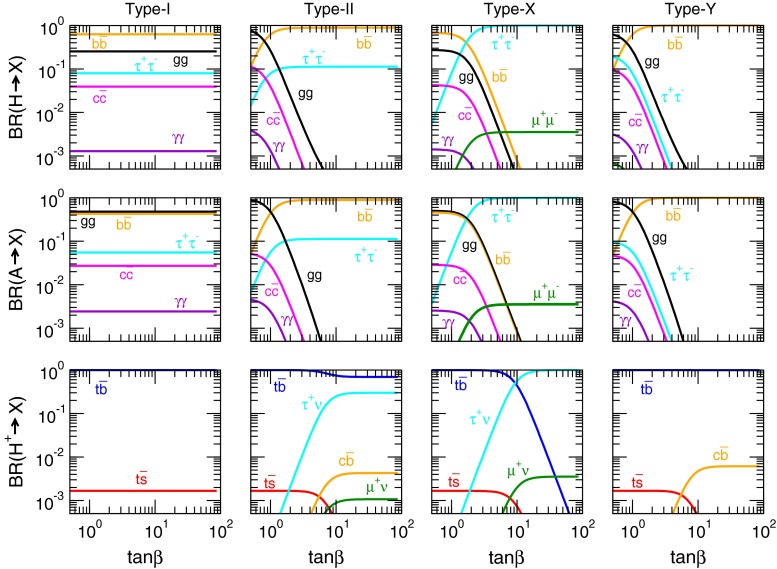
The decay branching ratios of *H*, *A* and $H^\pm$ in 2HDMs for Type I, Type II, Type X and Type Y as a function of $\tan \beta$ with $m_H=m_A=m_{H^\pm }=250$ GeV and $\sin (\beta -\alpha )=1$ [295]

Yukawa interaction in Eq. (38) is rewritten in terms of the mass eigenstates as

39 $$\begin{aligned} {\mathscr {L}}^\mathrm{2HDM}_\mathrm{Yukawa}= & {} -\sum _{f=u,d,\ell } \left[ \frac{m_f}{v}\xi _h^f\bar{f}fh+\frac{m_f}{v}\xi _H^f\bar{f}fH \right. \nonumber \\&\left. -\,i\frac{m_f}{v}\xi _A^f\gamma _5\bar{f}fA\right] \nonumber \\&-\left\{ \frac{\sqrt{2}V_{ud}}{v}\bar{u}\left[ m_u\xi _A^uP_L+m_d\xi _A^dP_R \right] dH^+ \right. \nonumber \\&\left. + \frac{\sqrt{2}m_\ell }{v}\xi _{A}^\ell \bar{v}_{L}\ell _RH^+ +\mathrm{h.c.} \right\} , \end{aligned}$$

where $P_{R,L}$ are the chiral projection operators. The coefficients $\xi _\phi ^f$ are summarised in Table 20.

There are two possibilities to explain the current LHC data, which show that the Higgs sector is approximately SM-like. When $M^2 \gg v^2$, the additional Higgs bosons *H*, *A* and $H^\pm$ are as heavy as $\sqrt{M^2}$, and only *h* stays at the electroweak scale, behaving as the SM-like Higgs boson. The effective Lagrangian is

40 $$\begin{aligned} {\mathscr {L}}_\mathrm{eff} = {\mathscr {L}}_\mathrm{SM} + \frac{1}{M^2} {\mathscr {O}}^{(6)}. \end{aligned}$$

Another case is for $\sqrt{M^2} \sim v$. In the limit where the *hWW* coupling takes the same value as the SM prediction $\sin (\beta -\alpha )=1$, all the Yukawa couplings and the self-coupling for *h* take the SM values, while *HWW* is zero. In this case, *h* behaves as the SM-like Higgs boson. Contrary, *H*, *A* and $H^\pm$ do not couple to gauge bosons, and they only couple to the SM particles via Yukawa interaction. When $\sin (\beta -\alpha )$ is slightly smaller than unity, the couplings *hVV* ($V=W$, *Z*) and *hff* ($f=t,b,c, \ldots$) deviate from the SM predictions depending on the type of Yukawa interaction. By detecting the pattern of the deviation in each coupling of *h* at future experiments, we can distinguish the type of Yukawa coupling in the 2HDMs even without directly discovering the additional Higgs bosons.

The decay widths and branching ratios of additional Higgs bosons can be calculated for given values of $\tan \beta$, $\sin (\beta -\alpha )$ and the masses for each type of Yukawa interaction. We refer to Ref. [146] where the total decay widths are discussed in details for $\sin (\beta -\alpha )\simeq 1$. Explicit formulae for all the partial decay widths can be found, e.g., in Ref. [146].

**Fig. 65:**
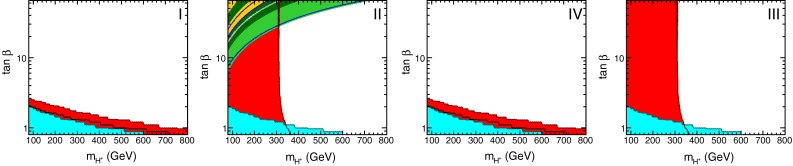
The constraint on the parameter space in the 2HDM for Type I, Type II, Type IV (Type X) and Type III (Type Y) from various flavour experiments [311]

In Fig. 64, decay branching ratios of additional Higgs bosons *H*, *A*, and $H^\pm$ are plotted in each type of Yukawa interaction as a function of $\tan \beta$ for the masses of 250 GeV. For simplicity, the SM-like limit $\sin (\beta -\alpha )=1$ is taken. In this limit, the decay modes of $H\rightarrow W^+W^-$, *ZZ*, *hh* as well as $A\rightarrow Zh$ are absent. In this limit, decay branching ratios of the SM-like Higgs boson are completely the same as those in the SM at the tree level, so that we cannot distinguish models by precision measurements of the couplings of the SM-like Higgs boson *h*.^16^

*Constraints on the Higgs potential from perturbative unitarity and vacuum stability*

The condition of tree-level unitarity requires the scattering amplitudes to be perturbative [296, 297]; i.e. $|a^0_i|<1/2$ [86], where $a^0_i$ are the eigenvalues of the s-wave amplitudes of the elastic scatterings of the longitudinal component of weak gauge bosons and the Higgs boson. In the 2HDM with the softly broken $Z_2$ symmetry, this condition gives constraints on the quartic couplings in the Higgs potential [298–300]. The eigenvalues for $14\times 14$ scattering matrix for neutral states are given as [298],

41a $$\begin{aligned}&a_1^{\pm } = \frac{1}{16\pi }\left[ \frac{3}{2}(\lambda _1+\lambda _2) \pm \sqrt{\frac{9}{4}(\lambda _1-\lambda _2)^2+(2\lambda _3+\lambda _4)^2} \right] ,\end{aligned}$$

41b $$\begin{aligned}&a_2^{\pm } = \frac{1}{16\pi }\left[ \frac{1}{2}(\lambda _1+\lambda _2) \pm \sqrt{\frac{1}{4}(\lambda _1-\lambda _2)^2+\lambda _4^2} \right] ,\end{aligned}$$

41c $$\begin{aligned}&a_3^{\pm } = \frac{1}{16\pi }\left[ \frac{1}{2}(\lambda _1+\lambda _2) \pm \sqrt{\frac{1}{4}(\lambda _1-\lambda _2)^2+\lambda _5^2} \right] ,\end{aligned}$$

41d $$\begin{aligned}&a_4 = \frac{1}{16\pi }(\lambda _3+2\lambda _4-3\lambda _5), a_5 = \frac{1}{16\pi }(\lambda _3-\lambda _5),\end{aligned}$$

41e $$\begin{aligned}&a_6 = \frac{1}{16\pi }(\lambda _3+2\lambda _4+3\lambda _5), a_7 = \frac{1}{16\pi }(\lambda _3+\lambda _5),\end{aligned}$$

41f $$\begin{aligned}&a_8 = \frac{1}{16\pi }(\lambda _3+\lambda _4), \end{aligned}$$

and for singly charged states, one additional eigenvalue is added [299]:

42a $$\begin{aligned} a_9 = \frac{1}{16\pi }(\lambda _3-\lambda _4). \end{aligned}$$

The condition of vacuum stability that the Higgs potential must be bounded from below gives [301–303]

43 $$\begin{aligned} \lambda _1>0,\quad \lambda _2>0,\quad \sqrt{\lambda _1\lambda _2}+\lambda _3+\mathrm{Min}(0,\lambda _4-|\lambda _5|)>0. \end{aligned}$$

The parameter space of the model is constrained by these conditions on the coupling constants in the Higgs potential.

*Constraints on the Higgs potential from electroweak precision observables*

Further constraints on the Higgs sector of the 2HDM are from the electroweak precision measurements. The *S*, *T* and *U* parameters [304] are sensitive to the loop effects of Higgs bosons [305, 306]. The *T* parameter corresponds to the electroweak $\rho$ parameter, which is severely constrained by experimental observations as has been discussed. The mass splitting between the additional Higgs bosons are strongly bounded [307, 308]. This implies that the Higgs potential has to respect the custodial *SU*(2) symmetry approximately.

*Flavour constraints on*$m_{H^\pm }$*and *$\tan \beta$

Flavour experiments provide strong constraints on the 2HDMs through the $H^\pm$ contribution to the flavour mixing observables at the tree level or at the loop level [146, 309, 310]. Because the amplitudes of these processes necessarily contain the Yukawa interaction, constraints on the 2HDM strongly depends on the type of Yukawa interaction. In Ref. [311], the limits on the general couplings from flavour physics are translated into those on the ($m_{H^\pm },\tan \beta$) plane for all four types of Yukawa interaction in the 2HDM, see Fig. 65, where Type III and Type IV correspond to Type Y and Type X, respectively. See also the more recent studies [312–314].

A strong exclusion limit is given from the result for the branching ratio of the $B\rightarrow X_s\gamma$ process [315]. For Type-II and Type-Y, a $\tan \beta$-independent lower limit of $m_{H^\pm }\gtrsim 380$ GeV is obtained [316] by comparing with the NNLO calculation [317]. For Type-I and Type-X, on the other hand, $\tan \beta \lesssim 1$ is excluded for $m_{H^\pm }\lesssim 800$ GeV, while no lower bound on $m_{H^\pm }$ is obtained.

By the results for the $B_{d}^0$–$\bar{B}^0_{d}$ mixing, lower $\tan \beta$ regions ($\tan \beta \le 1$) are excluded for $m_{H^\pm }\lesssim 500$ GeV for all types of Yukawa interaction [315].

Constraints in larger $\tan \beta$ regions are obtained only for Type-II, which come from the results for leptonic meson decay processes [315], $B\rightarrow \tau \nu$ [318] and $D_s\rightarrow \tau \nu$ [319]. Upper bounds on $\tan \beta$ are obtained at around 30 for $m_{H^\pm }\simeq 350$ GeV and around 60 for $m_{H^\pm }\simeq 700$ GeV [311]. On the other hand, the other types do not receive any strong constraint for large $\tan \beta$ values, because the relevant couplings behave $\xi _A^d\xi _A^\ell =\tan ^2\beta$ for Type-II while $\xi _A^d\xi _A^\ell =-1$ ($\cot ^2\beta$) for Type-X and Type-Y (Type-I).

*Constraint from the data at LEP/SLC, Tevatron and also from the current LHC data*

At the LEP direct search experiments, lower mass bounds on *H* and *A* have been obtained as $m_H>92.8$ GeV and $m_A>93.4$ GeV in the ${\textit{CP}}$-conservation scenario [320, 321]. Combined searches for $H^\pm$ give the lower mass bound $m_{H^\pm }>80$ GeV, by assuming ${\mathscr {B}}(H^+\rightarrow \tau ^+\nu )+{\mathscr {B}}(H^+\rightarrow c\bar{s})=1$ [322–324].

At the Fermilab Tevatron, CDF and D0 Collaborations have studied the processes of $p\bar{p}\rightarrow b\bar{b}H/A$, followed by $H/A\rightarrow b\bar{b}$ or $H/A\rightarrow \tau ^+\tau ^-$ [325–327]. By using the $\tau ^+\tau ^-$ ($b\bar{b}$) decay mode, which can be sensitive for the cases of Type-II (Type-II and Type-Y), upper bounds on $\tan \beta$ have been obtained to be from about 25 to 80 (40 to 90) for $m_A$ from 100 to 300 GeV, respectively. For the direct search of $H^\pm$, the decay modes of $H^\pm \rightarrow \tau \nu$ and $H^\pm \rightarrow cs$ have been investigated by using the production from the top quark decay $t\rightarrow bH^\pm$ [328–330]. Upper bounds on ${\mathscr {B}}(t\rightarrow bH^\pm )$ have been obtained, which can be translated into the bound on $\tan \beta$ in various scenarios. For Type-I with $H^\pm$ heavier than the top quark, upper bounds on $\tan \beta$ have been obtained to be from around 20 to 70 for $m_{H^\pm }$ from 180 to 190 GeV, respectively [328].

At the LHC, additional Higgs-boson searches have been performed by using currently accumulated events at the experiments with a centre-of-mass energy of 7 TeV with the integrated luminosity of 4.9 fb$^{-1}$ in 2011 and also 8 TeV with 19.7 fb$^{-1}$ in 2012. The CMS Collaboration has searched *H* and *A*, which decay into the $\tau ^+\tau ^-$ final state, and upper limits on $\tan \beta$ have been obtained in the MSSM (or in the Type-II 2HDM) from 4 to 60 for $m_A$ from 140 GeV to 900 GeV, respectively [331]. By the ATLAS Collaboration similar searches have also been done [332]. In the Type-II and Type-Y 2HDMs, CMS has also searched the bottom-quark associated production process of *H* or *A* which decays into the $b\bar{b}$ final state [333], and has obtained the upper bounds on $\tan \beta$: i.e., $\tan \beta \gtrsim 16$ (28) is excluded at $m_{A}=100$ GeV (350 GeV). ATLAS has reported the $H^\pm$ searches via the $\tau$+jets final state [249, 334]. In the Type-II 2HDM with $m_{H^{\pm }}\lesssim m_{t}$, wide parameter regions have been already excluded by the data for 100 GeV $\lesssim m_{H^\pm }\lesssim 140$ GeV with $\tan \beta \gtrsim 1$. Moreover, the parameter regions of $\tan \beta \gtrsim 50$ at $m_{H^\pm }=200$ GeV and $\tan \beta \gtrsim 65$ at $m_{H^\pm }=300$ GeV have been excluded for $m_{H^\pm }\gtrsim 180$ GeV, respectively. The searches for $H^\pm$ in the *cs* final state have been performed by ATLAS [335], and the upper limit on the branching ratio of $t\rightarrow bH^\pm$ decay is obtained assuming the 100 % branching ratio of $H^\pm \rightarrow cs$. For $\sin (\beta -\alpha )<1$, searches for $H\rightarrow W^+W^-$, *hh* and $A\rightarrow Zh$ give constraints on the 2HDMs with Type-I and Type-II Yukawa interactions [336, 337].

**Fig. 66:**
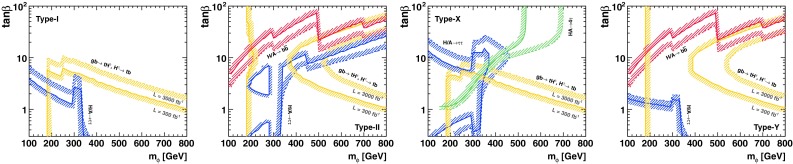
Expected exclusion regions ($2\sigma$ CL) in the plane of $\tan \beta$ and the mass scale $m_{\phi }$ of the additional Higgs bosons at the LHC. *Curves* are evaluated by using the signal and background analysis given in Ref. [338] for each process, where the signal events are rescaled to the prediction in each case [339, 340], except the $4\tau$ process for which we follow the analysis in Ref. [341]. *Thick solid lines* are the expected exclusion contours by $L=300$ fb$^{-1}$ data, and *thin dashed lines* are for $L=3000$ fb$^{-1}$ data. For Type-II, the regions indicated by *circles* may not be excluded by $H/A\rightarrow \tau ^+\tau ^-$ search by using the 300 fb$^{-1}$ data due to the large SM background

*Prospect of extra Higgs-boson searches at the LHC (13–14 TeV)*

At the LHC experiments with the collision energy of 13–14 TeV and the integrated luminosity of $L=300$ fb$^{-1}$ and also 3000 fb$^{-1}$, the expected discovery potential for additional Higgs bosons have been studied in the 2HDM in Refs. [295, 339, 340], by using the signal and background analysis for various channels given in Ref. [338]. Processes available for the searches for additional Higgs bosons are [295]

- $H/A (+ b\bar{b})$ inclusive and associated production followed by the $H/A\rightarrow \tau ^+\tau ^-$ decay [342].
- $H/A+b\bar{b}$ associated production followed by the $H/A\rightarrow b\bar{b}$ decay [342–345].
- $gb\rightarrow tH^\pm$ production followed by the $H^\pm \rightarrow tb$ decay [346, 347].
- $$q\bar{q}\rightarrow HA\rightarrow 4\tau $$ process [341, 348].

For the production cross sections, the tree-level cross sections have been convoluted with the CTEQ6L parton distribution functions [349]. The scales of the strong coupling constant and the parton distribution function are chosen to the values used in Ref. [350]. For details, see Ref. [295], where the latest recommendations from the LHC Higgs Cross Section Working Group for 2HDM cross section (and branching ratio) evaluations can be found in Ref. [351].

In Fig. 66, the contour plots of the expected exclusion regions [$2\sigma$ confidence level (CL)] in the $(m_\phi ,\tan \beta )$ plane are shown at the LHC $\sqrt{s}=14$ TeV with the integrated luminosity of 300 fb$^{-1}$ (thick solid lines) and 3000 fb$^{-1}$ (thin dashed lines), where $m_\phi$ represents common masses of additional Higgs bosons. From the left panel to the right panel, the results for Type-I, Type-II, Type-X and Type-Y are shown separately. Following the analysis in Ref. [338], the reference values of the expected numbers of signal and background events are changed at the several values of $m_\phi$ [295, 340], which makes sharp artificial edges of the curves in Fig. 66.

For Type-I, *H* / *A* production followed by the decay into $\tau ^+\tau ^-$ can be probed for $\tan \beta \lesssim 3$ and $m_{H,A}\le 350$ GeV, where the inclusive production cross section is enhanced by the relatively large top Yukawa coupling with the sizeable $\tau ^+\tau ^-$ branching ratio. The $tH^\pm$ production decaying into $H^\pm \rightarrow tb$ can be used to search $H^\pm$ in relatively smaller $\tan \beta$ regions. $H^\pm$ can be discovered for $m_{H^\pm } < 800$ GeV and $\tan \beta \lesssim 1$ (2) for the integrated luminosity of 300 fb$^{-1}$ (3000 fb$^{-1}$).

For Type-II, the inclusive and the bottom-quark-associated production processes of *H* / *A* with the decay into $\tau ^+\tau ^-$ or the $b \overline{b}$ can be used to search *H* and *A* for relatively large $\tan \beta$. They can also be used in relatively small $\tan \beta$ regions for $m_{H,A}\lesssim 350$ GeV. $H^\pm$ can be searched by the $tH^\pm$ production with $H^\pm \rightarrow tb$ decay for $m_{H^\pm }\gtrsim 180$ GeV for relatively small and large $\tan \beta$ values. The region of $m_{H^\pm }\gtrsim 350$ GeV (500 GeV) could be excluded with the 300 fb$^{-1}$ (3000 fb$^{-1}$) data.

For Type-X, *H* and *A* can be searched via the inclusive production and *HA* pair production by using the $\tau ^+\tau ^-$ decay mode, which is dominant. The inclusive production could exclude the region of $\tan \beta \lesssim 10$ with $m_{H,A}\lesssim 350$ GeV. Regions up to $m_{H,A}\simeq 500$ GeV (700 GeV) with $\tan \beta \gtrsim 10$ could be excluded by using the pair production with the 300 fb$^{-1}$ (3000 fb$^{-1}$) data. The search for $H^\pm$ is similar to that for Type-I.

Finally, for Type-Y, the inclusive production of *H* and *A* f ollowed by $H/A \rightarrow \tau ^+\tau ^-$ can be searched for the regions of $\tan \beta \lesssim 2$ and $m_{H,A}\le 350$ GeV. The bottom-quark associated production of *H* and *A* with $H/A\rightarrow b\bar{b}$ can be searched for the regions of $\tan \beta \gtrsim 30$ up to $m_{H,A}\simeq 800$ GeV. The search of $H^\pm$ is similar to that for Type-II.

For Type-II and Type-Y (Type-X), if all the curves are combined by assuming that all the masses of additional Higgs bosons are the same, the mass below 400 GeV (350 GeV) coud be excluded by the 300 fb$^{-1}$ data for all value of $\tan \beta$, and with 3000 fb$^{-1}$, the mass below 550 GeV (400 GeV) could be excluded. On the other hand, for Type-I, the regions with $\tan \beta \gtrsim 5$ (10) cannot be excluded by 300 fb$^{-1}$ (3000 fb$^{-1}$) data. In the general 2HDM, however, the mass spectrum of additional Higgs boson has more degrees of freedom, so that we can still find allowed parameter regions where $m_H$ is relatively light but $m_A (\simeq m_{H^\pm })$ are heavy. Thus, the overlaying of these exclusion curves for different additional Higgs bosons may only be applied to the case of $m_H=m_A=m_{H^\pm }$.

**Fig. 67:**
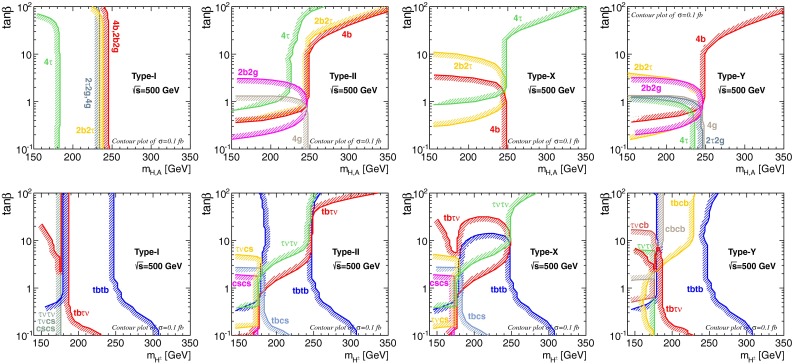
Contour plots of the four-particle production cross sections through the *H* / *A* production and $H^\pm$ production process at the ILC with $\sqrt{s}=500$  GeV in the $(m_{H^\pm },\tan \beta )$ plane. Contour of $\sigma =0.1$ fb is drawn for each signature [295]

If $H^\pm$ is discovered at the LHC, its mass could be determined immediately [338, 352]. Then the determination of the type of the Yukawa interaction becomes important. At the LHC, however, we would not completely distinguish the types of Yukawa interaction, because the Type-I and Type-X, or Type-II and Type-Y have a common structure for the $tbH^\pm$ interaction. In addition, as seen in Fig. 66, there can be no complementary process for the neutral Higgs-boson searches in some parameter regions; e.g., $m_{H,A}\gtrsim 350$ GeV with relatively small $\tan \beta$, depending on the type of the Yukawa interaction. At the ILC, on the other hand, as long as $m_{H,A}\lesssim 500$ GeV, the neutral Higgs bosons can be produced and investigated almost independent of $\tan \beta$. Therefore, it is quite important to search for the additional Higgs bosons with the mass of 350–500 GeV, and to determine the models and parameters at the ILC, even after the LHC.

Notice that the above results are obtained in the SM-like limit, $\sin (\beta -\alpha )=1$. A deviation from the SM-like limit causes appearance of additional decay modes such as $H\rightarrow W^+W^-$, *ZZ*, *hh* as well as $A\rightarrow Zh$ [86, 353–355]. Especially, for Type-I with a large value of $\tan \beta$, branching ratios of these decay modes can be dominant even with a small deviation from the SM-like limit [146, 354]. Therefore, searches for additional Higgs bosons in these decay modes can give significant constraints on the deviation of $\sin (\beta -\alpha )$ from the SM-like limit [336, 337], which is independent of coupling constants of *hVV*.

*Prospect for the searches for the additional Higgs bosons at the ILC*

At LCs the main production mechanisms of additional Higgs bosons in the 2HDM are $e^+ e^- \rightarrow H A$ and $e^+ e^- \rightarrow H^+ H^-$, where a pair of additional Higgs bosons is produced via gauge interactions as long as kinematically allowed. For energies below the threshold, the single production processes, $e^+e^- \rightarrow H(A) f \bar{f}$ and $e^+ e^- \rightarrow H^\pm f \bar{f}'$ are the leading contributions [356]. They are enhanced when the relevant Yukawa couplings $\phi f \bar{f}^{(')}$ are large. The cross sections of these processes have been studied extensively [206, 356–358], mainly for the MSSM or for the Type-II 2HDM.

Here, we discuss the result in the general 2HDMs but with softly broken discrete symmetry. The following processes are considered:

44a $$\begin{aligned}&e^+e^-\rightarrow \tau ^+\tau ^- H, \quad \tau ^+\tau ^- A,\end{aligned}$$

44b $$\begin{aligned}&e^+e^-\rightarrow b\bar{b} H, \quad b\bar{b} A,\end{aligned}$$

44c $$\begin{aligned}&e^+e^-\rightarrow t\bar{t} H, \quad t\bar{t} A, \end{aligned}$$

44d $$\begin{aligned}&e^+e^-\rightarrow \tau ^-\nu H^+, \quad \tau ^+ \bar{\nu } H^-, \end{aligned}$$

44e $$\begin{aligned}&e^+e^-\rightarrow \bar{t}b H^+, \quad \bar{b}t H^-. \end{aligned}$$

For energies above the threshold of the pair production, $\sqrt{s}>m_H+m_A$, the contribution from $e^+e^-\rightarrow HA$ can be significant in the processes in Eqs. (44a)–(44c). Similarly for $\sqrt{s}>2m_{H^\pm }$, the contribution from $e^+e^-\rightarrow H^+H^-$ can be significant in the processes in Eqs. (44d, 44e). Below the threshold, the processes including diagrams of $e^+e^-\rightarrow f \bar{f}^*$ and $e^+e^-\rightarrow f^*\bar{f}$ dominate.

**Fig. 68:**
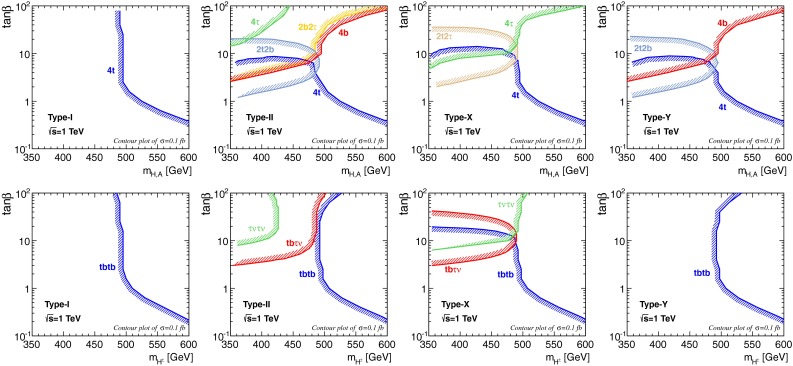
Contour plots of the four-particle production cross sections through the *H* / *A* production and $H^\pm$ production process at the ILC with $\sqrt{s}=1$  TeV in the $(m_{H^\pm },\tan \beta )$ plane. Contour of $\sigma =0.1$ fb is drawn for each signature [295]

**Table 21:** Expected signatures to be observed at the LHC and ILC for the benchmark scenarios with $m_\phi =220$ GeV [295]. Observable final states are listed as the signatures of additional Higgs bosons, *H*, *A* and $H^{\pm }$. LHC300, LHC3000, ILC500 represent the LHC run of 300, 3000 fb$^{-1}$ luminosity, ILC run of 500 GeV, respectively

$(m_\phi , \tan \beta )$	Type-I		Type-II		Type-X		Type-Y	
	*H*, *A*	$H^\pm$	*H*, *A*	$H^\pm$	*H*, *A*	$H^\pm$	*H*, *A*	$H^\pm$
(220 GeV, 20)								
LHC300	$-$	$-$	$\tau \tau$, *bb*	*tb*	$4\tau$	$-$	*bb*	*tb*
LHC3000	$-$	$-$	$\tau \tau$, *bb*	*tb*	$4\tau$	$-$	*bb*	*tb*
ILC500	$4b,2b2\tau ,4g$ $,2b2g,2\tau 2g$	*tbtb*	$4b,2b2\tau$,$4\tau$	$tbtb,tb\tau \nu ,$ $\tau \nu \tau \nu ^{}$	$4\tau$	$tb\tau \nu$,$\tau \nu \tau \nu$	4*b*	*tbtb*, *tbcb*
(220 GeV, 7)								
LHC300	–	–	$\tau \tau$	*tb*	$4\tau$	–	–	*tb*
LHC3000	–	*tb*	$\tau \tau$	*tb*	$\tau \tau ,4\tau$	–	–	*tb*
ILC500	$4b,2b2\tau ,4g, \,2b2g,2\tau 2g$	*tbtb*	$4b,2b2\tau ,4\tau$	$tbtb,tb\tau \nu$, $\tau \nu \tau \nu$	$2b2\tau ,4\tau$	$tbtb,tb\tau \nu$, $\tau \nu \tau \nu$	4*b*	*tbtb*, *tbcb*
(220 GeV, 2)								
LHC300	$-$	*tb*	$\tau \tau$	*tb*	$\tau \tau ,4\tau$	*tb*	$-$	*tb*
LHC3000	$\tau \tau$	*tb*	$\tau \tau$	*tb*	$\tau \tau ,4\tau$	*tb*	$-$	*tb*
ILC500	$4b,2b2\tau ,4g, 2b2g,2\tau 2g$	*tbtb*	$4b,2b2\tau$, $4\tau ,2b2g$	*tbtb*,$tb\tau \nu$	$4b,2b2\tau$, $4\tau$	*tbtb*,$tb\tau \nu$	$4b,2b2\tau ,\,2b2g$	*tbtb*

Both the pair and the single production processes of additional Higgs bosons mostly result in four-particle final states (including neutrinos). In Ref. [295], the cross sections of various four-particle final states are studied for given masses of additional Higgs bosons and $\tan \beta$ with setting $\sin (\beta -\alpha )=1$, and draw contour curves where the cross sections are 0.1 fb. This value is chosen commonly for all processes as it could be regarded as a typical order of magnitude of the cross section of the additional Higgs boson production [358]. In addition, this value can also be considered as a criterion for observation with the expected integrated luminosity at the ILC [56, 206]. Certainly, the detection efficiencies are different for different four-particle final states, and the decay of unstable particles such as tau leptons and top quarks have to be considered if they are involved. We here restrict ourselves to simply compare the various four-particle production processes in four types of Yukawa interaction in the 2HDMs with taking the criterion of 0.1 fb as a magnitude of the cross sections. Expected background processes and a brief strategy of observing the signatures are discussed in Ref. [295].

In Fig. 67, contour plots of the cross sections of four-particle production processes through *H* and/or *A* are shown in the $(m_{H/A},\tan \beta )$ plane (upper figures), and those through $H^\pm$ are shown in the$(m_{H^\pm },\tan \beta )$ plane (lower figures) for the collision energy to be $\sqrt{s}=500$ GeV. From left to right, the figures correspond to the results in Type 1, Type II, Type X and Type Y. We restrict ourselves to consider the degenerated mass case, $m_H=m_A$.

In Fig. 68, contour plots of the cross sections of four-particle production processes through *H* and/or *A* are shown in the $(m_{H/A},\tan \beta )$ plane (upper figures), and those through $H^\pm$ are shown in the$(m_{H^\pm },\tan \beta )$ plane (lower figures) for the collision energy to be $\sqrt{s}=1$ TeV. From left to right, the figures correspond to the results in Type 1, Type II, Type X and Type Y. We restrict ourselves to consider the degenerated mass case, $m_H=m_A$.

We here give a comment on the SM background processes and their cross sections [295]. In general, for the four-particle production processes, the SM background cross sections are larger for $\sqrt{s}=250$ GeV, but decrease with the collision energy. The typical orders of cross sections are of the order of 1–10 fb for the $Z/\gamma$-mediated processes, and of the order of 10–100 fb for the processes which are also mediated by $W^\pm$. For the four-quark production processes, gluon exchange diagrams also contribute. In order to reduce the background events, efficient kinematical cuts are required.

The cross section of the 4*t* production is very small in the SM. Therefore, a clean signature can be expected to be detected in this mode. Detailed studies on the signal and background processes for *tbtb* production can be found in Ref. [357], and the signal-to-background analysis for the $4\tau$ production can be found in Ref. [359] with the reconstruction method of the masses of additional Higgs bosons.

Finally, we discuss some concrete scenarios to show the complementarity of direct searches for the additional Higgs bosons in the 2HDMs at the LHC and the ILC. As benchmark scenarios, three cases $\tan \beta =2$, 7 and 20 are considered for $m_\phi =220$ GeV and $\sin (\beta -\alpha )=1$, where $m_\phi$ represents the common mass of *H*, *A* and $H^\pm$. In Table 21, the expected signatures of *H* / *A* and $H^\pm$ are summarised to be observed at the LHC with 300, 3000 fb$^{-1}$ and at the ILC with $\sqrt{s}=500$ GeV.

First, for the case of $(m_\phi , \tan \beta ) = (220~\mathrm{GeV}, 20)$. no signature is predicted for Type-I, while different signatures are predicted for Type-II, Type-X and Type-Y at the LHC with 300 and 3000 fb$^{-1}$. Therefore those three types could be discriminated at the LHC. On the other hand, at the ILC with $\sqrt{s}=500$ GeV, all the four types of the Yukawa interaction including Type-I predict signatures which are different from each other. Therefore, complete discrimination of the type of Yukawa interaction could be performed at the ILC.

Next, we turn to the second case with $(m_\phi , \tan \beta ) = (220~\mathrm{GeV}, 7)$. At the LHC with 300 fb$^{-1}$, Type-I cannot be observed, while Type-II, Type-X and Type-Y are expected to be observed with different signatures. At the LHC with 3000 fb$^{-1}$, the signature of Type-I can also be observed with the same final state as Type-Y. Type-I and Type-Y can be basically separated, because for Type-Y the signals can be observed already with 300 fb$^{-1}$, while for Type-I that can be observed only with 3000 fb$^{-1}$. Therefore, at the LHC with 3000 fb$^{-1}$, the complete discrimination can be achieved. At the ILC, the four types of Yukawa interaction can also be separated by a more variety of the signatures for both channels with the neutral and charged Higgs bosons.

Finally, for the case of $(m_\phi , \tan \beta ) = (220~\mathrm{GeV}, 2)$, signals for all the four types of Yukawa interaction can be observed at the LHC with 300 fb$^{-1}$. However, the signatures of Type-I and Type-Y are identical, so that the two types cannot be discriminated. With 3000 fb$^{-1}$, the difference between the Type-I and Type-Y emerges in the *H* / *A* signature. Therefore the two types can be discriminated at this stage. Again, at the ILC, the four types can also be separated with a more variety of the signatures for both channels with the neutral and charged Higgs bosons.

*Fingerprinting the type of the 2HDM by precision measurement of the Higgs couplings at the ILC*

Extra Higgs bosons in extended Higgs sectors can be discovered as long as their masses are not too large as compared to the electroweak scale. On the other hand, at the ILC [360], these extended Higgs sectors can also be tested by accurately measuring the coupling constants with the discovered Higgs bosons *h*. This is complementary with the direct searches at the LHC.

**Fig. 69:**
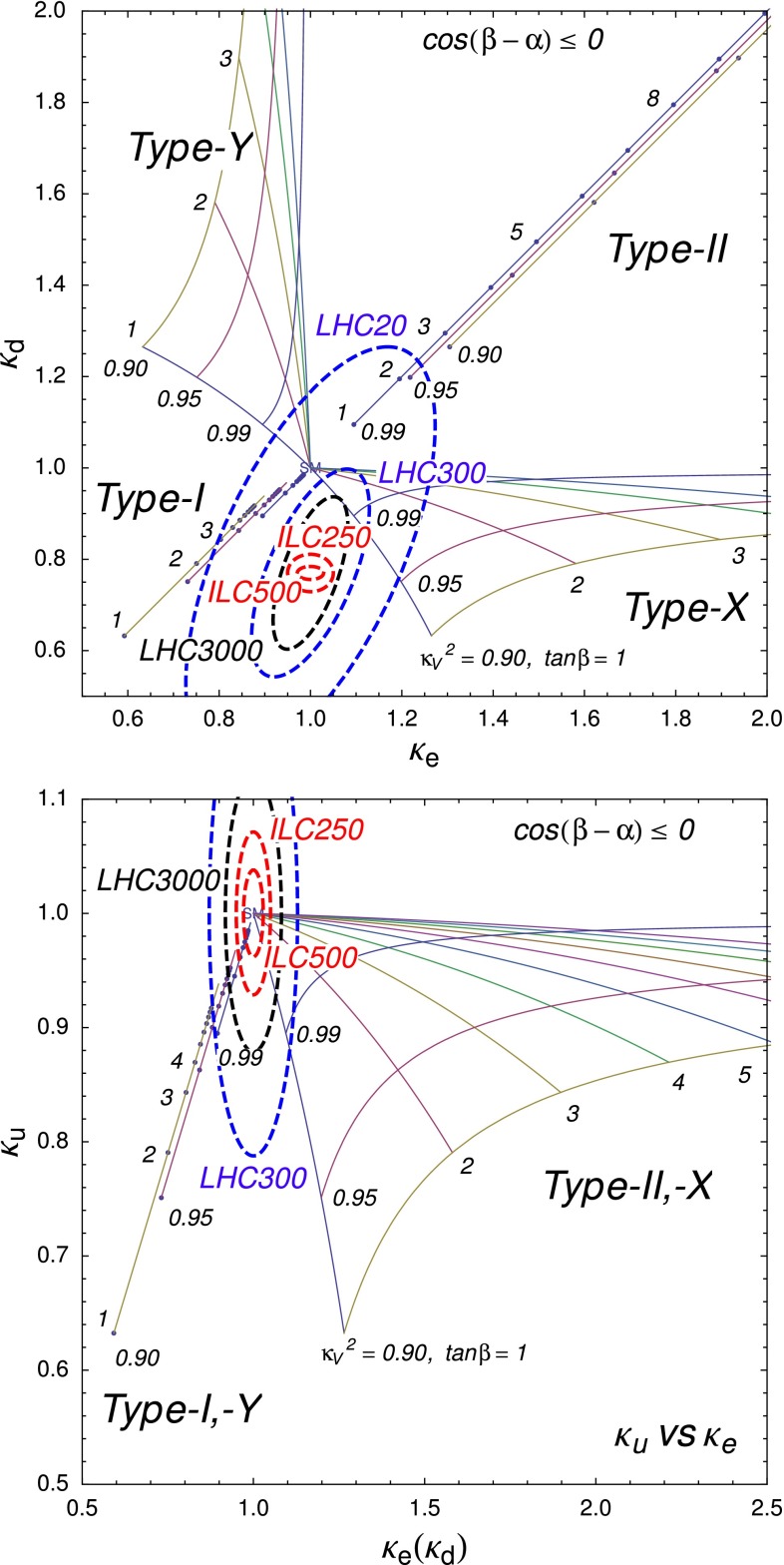
*Left* the scaling factors in 2HDM with four types of Yukawa interactions. *Right* the scaling factors in models with universal Yukawa couplings. The current LHC bounds and the expected LHC and ILC sensitivities are also shown at the 68.27 % CL. For details, see Refs. [339, 340]

In the extended Higgs sectors, the gauge couplings and Yukawa interactions of *h* are parameterised by

45 $$\begin{aligned} {\mathscr {L}}^\mathrm{int}= & {} +\kappa _W \frac{2m_W^2}{v} hW^{+\mu }W^-_\mu + \kappa _Z \frac{m_Z^2}{v} hZ^\mu Z_\mu \nonumber \\&-\,\sum _f\kappa _f\frac{m_f}{v} {\overline{f}}fh + \cdots , \end{aligned}$$

where $\kappa _V$ ($V=W$ and *Z*) and $\kappa _f$ ($f=t,b,c, \cdots$) are the scaling factors measuring the deviation from the SM predictions. In the SM, we have $\kappa _V=\kappa _f=1$. According to Refs. [268, 339, 360], the *hVV* couplings are expected to be measured with about 4 % accuracy at the LHC with 300 fb$^{-1}$ (although requiring some theory input). The accuracy for the $ht\bar{t}$, $hb\bar{b}$ and $h\tau \tau$ couplings are supposed to be about 16, 14 and 11 %, respectively. At the ILC250 (ILC500) where the collision energy and the integrated luminosity are 250 GeV (500 GeV) and 250 fb$^{-1}$ (500 fb$^{-1}$) combining with the results assuming 300 fb$^{-1}$ at the LHC, the *hWW* and *hZZ* couplings are expected to be measured by about 1.9 % (0.2 %) and about 0.4 % (0.3 %), respectively. The $hc\bar{c}$, $hb\bar{b}$ and $h\tau \tau$ couplings are supposed to be measured by about 5.1 % (2.6 %), 2.8 % (1.0 %) and 3.3 % (1.8 %) at the ILC250 (ILC500). For the $ht\bar{t}$ coupling, it will be measured with 12.0 and 9.6 % at the ILC250 and ILC500, respectively.

In the 2HDM, the scaling factors $\kappa _V$ are given by $\kappa _V=\sin (\beta -\alpha )$, while those for the Yukawa interactions are given depending on the type of Yukawa interaction [146]. For the SM-like limit $\kappa _V^{}=1$, all the scaling factors $\kappa _f$ become unity. In Fig. 69 (left), the scale factors $\kappa _f$ in the 2HDM with the softly broken symmetry are shown on the $\kappa _\ell$–$\kappa _d$ plane for various values of $\tan \beta$ and $\kappa _V^{}$ ($=\sin (\beta -\alpha )$). The points and the dashed curves denote changes of $\tan \beta$ by steps of one. $\kappa _V$ ($=\kappa _W=\kappa _Z$) is taken as $\kappa _V^2 = 0. 99, 0.95$ and 0.90. The current LHC constraints as well as the expected LHC and ILC sensitivities for $\kappa _d$ and $\kappa _\ell$ are also shown at the 68.27 % Confidence Level (CL). For the current LHC constraints (LHC30), we take the numbers from the universal fit in Eq. (18) of Ref. [361]. For the future LHC sensitivities (LHC300 and LHC3000), the expectation numbers are taken from the Scenario 1 in Table 1 of Ref. [362]. The central values and the correlations are assumed to be the same as in LHC30. The ILC sensitivities are taken from Table. 2.6 in Ref. [360]. The same central value without correlation is assumed for the ILC sensitivity curves. For more details see Ref. [339], and for some revisions see Ref. [340].

**Fig. 70:**
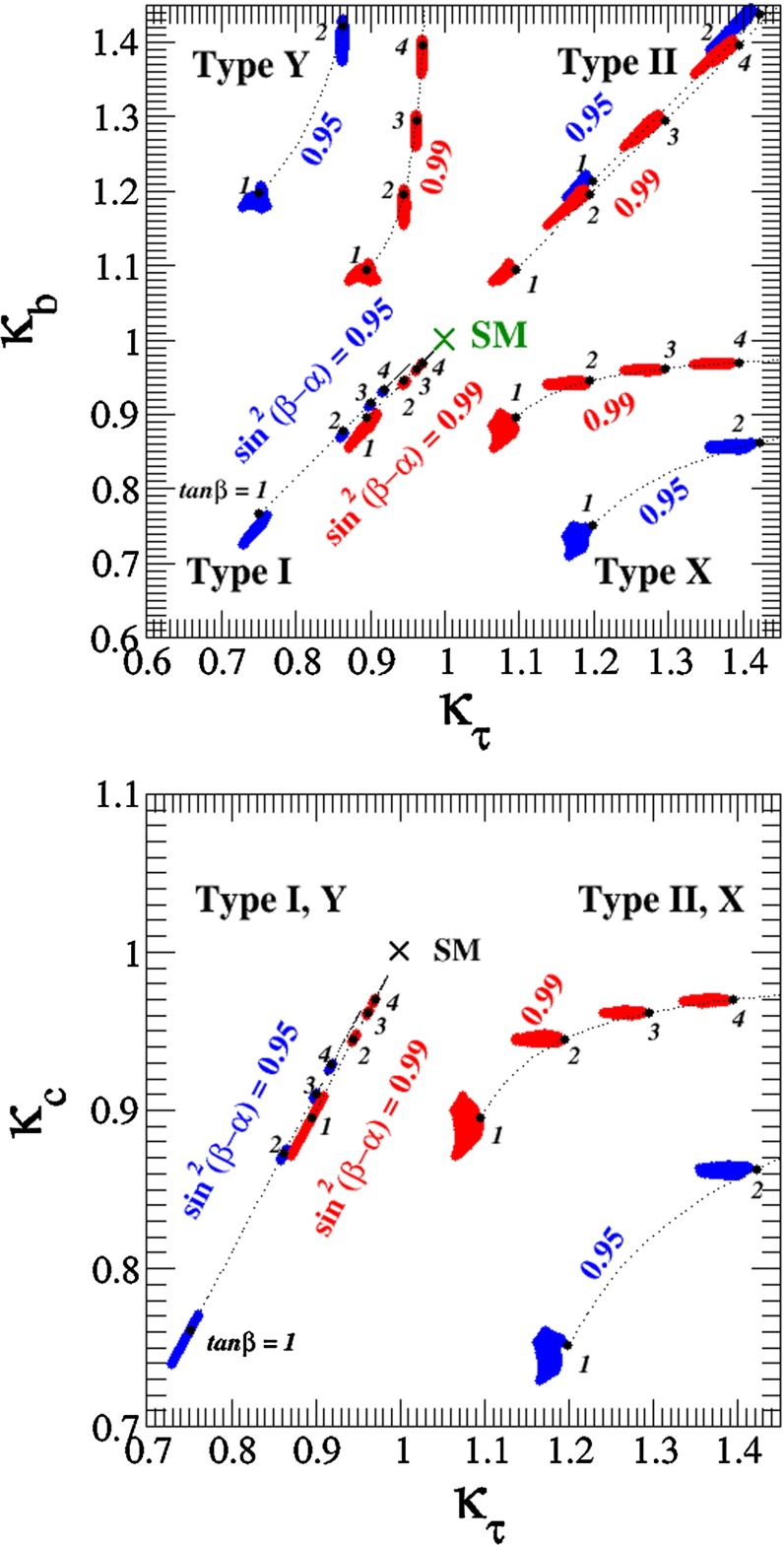
Predictions of various scale factors on the $\kappa _\tau$ vs. $\kappa _b$ (*upper panel*), and $\kappa _\tau$ vs $\kappa _c$ (*bottom panel*) in four types of Yukawa interactions in the cases with $\cos (\beta -\alpha )<0$ [293, 294]. *Each black dot* shows the tree-level result with $\tan \beta$=1, 2, 3 and 4. One-loop corrected results are indicated by *red* for $\sin ^2(\beta -\alpha )=0.99$ and *blue* for $\sin ^2(\beta -\alpha )=0.95$ regions where $m_\Phi$ and *M* are scanned over from 100 GeV to 1 TeV and 0 to $m_\Phi$, respectively. All the plots are allowed by the unitarity and vacuum stability bounds

The analysis including radiative corrections has been done recently [293, 294]. We show the one-loop results for the Yukawa couplings in the planes of fermion scale factors. In Fig. 70, predictions of various scale factors are shown on the $\kappa _\tau$ vs. $\kappa _b$ (upper panels), and $\kappa _\tau$ vs. $\kappa _c$ (bottom panels) planes. When we consider the case with $\sin (\beta -\alpha )\ne 1$, the sign dependence of $\cos (\beta -\alpha )$ to $\kappa _f$ is also important. We here show the both cases with $\cos (\beta -\alpha )<0$. The value of $\tan \beta$ is discretely taken as $\tan \beta$=1, 2, 3 and 4. The tree-level predictions are indicated by the black dots, while the one-loop corrected results are shown by the red for $\sin ^2(\beta -\alpha )=0.99$ and blue for $\sin ^2(\beta -\alpha )=0.95$ regions where the values of $m_\Phi$ and *M* are scanned over from 100 GeV to 1 TeV and 0 to $m_\Phi$, respectively. All the plots are allowed by the unitarity and vacuum stability bounds.

Even when we take into account the one-loop corrections to the Yukawa couplings, this behaviour; i.e., predictions are well separated among the four types of THDMs, does not so change as we see the red and blue coloured regions. Therefore, we conclude that all the 2HDMs can be distinguished from each other by measuring the charm, bottom and tau Yukawa couplings precisely when the gauge couplings *hVV* are deviated from the SM prediction with $\mathscr {O}(1)$ %.^17^

The Higgs-boson couplings $h\gamma \gamma$ and *hgg* are absent at the tree level but are produced at the one-loop level via the higher-dimensional operators

46 $$\begin{aligned} \frac{1}{M^2} |\Phi |^2 F^{\mu \nu } F_{\mu \nu }, \quad \frac{1}{M^2} |\Phi |^2 G^{(a)\mu \nu } G^{(a)}_{\mu \nu }, \end{aligned}$$

where $F^{\mu \nu }$ and $G^{(a)\mu \nu }$ are the field strength tensors of $U(1)_\mathrm{EM}$ and $SU(3)_C$, and *M* is a dimensionful parameter. In the 2HDM, the coupling can deviate from the SM due to the mixing effect of neutral scalar bosons and, for $h\gamma \gamma$, also due to the loop contributions of additional Higgs bosons *H*, *A* and $H^\pm$. The latter effect can be significant even in the SM-like limit where $\sin (\beta -\alpha )=1$ as long as *M* is not too large. At the LHC (300 fb$^{-1}$), the HL-LHC (3000 fb$^{-1}$), and the ILC (1 TeV-up) [268, 339], $\kappa _\gamma$ is expected to be measured with 5–7, 2–5 and 2.4 %, respectively. If deviations in $\kappa _\gamma$ and $\kappa _g$ are detected in future precision measurements at the LHC and the ILC, we can directly extract information of new particles in the loop such as their mass scales.

The triple Higgs-boson coupling *hhh* is essentially important to be measured to obtain the information of the Higgs potential. The tree-level behaviour of the *hhh* coupling constant has been discussed in the 2HDM in Refs. [220, 365–367]. The deviation from the SM predictions are sensitive to the mixing parameters $\tan \alpha$ and $\sin (\beta -\alpha )$. In the SM-like limit $\sin (\beta -\alpha )=1$, the value of the *hhh* coupling coincide with that in the SM. At the one-loop level, even when the SM-like limit, the *hhh* coupling can deviate from the SM prediction due to the quantum-loop effects of *H*, *A* and $H^\pm$ [284, 368]. For the SM-like limit $\sin (\beta -\alpha )=1$, the one-loop corrected effective *hhh* coupling in the 2HDM can be expressed as

47 $$\begin{aligned} \lambda _{hhh}^{eff}= & {} \frac{3 m_h^2}{v} \left\{ 1 + \frac{m_{H}^4}{12 \pi ^2 m_h^2 v^2} \left( 1 - \frac{M^2}{m_H^2}\right) ^3 \right. \nonumber \\&\left. + \frac{m_{A}^4}{12 \pi ^2 m_h^2 v^2} \left( 1 - \frac{M^2}{m_A^2}\right) ^3 + \frac{m_{H^\pm }^4}{6 \pi ^2 m_h^2 v^2} \left( 1 - \frac{M^2}{m_{H^\pm }^2}\right) ^3\right. \nonumber \\&\left. - \frac{N_{c_t} m_t^4}{3 \pi ^2 m_h^2 v^2} + {\mathscr {O}} \left( \frac{p^2_i m_\Phi ^2}{m_h^2 v^2}, \frac{m_\Phi ^2}{v^2}, \frac{p^2_i m_t^2}{m_h^2 v^2}, \frac{m_t^2}{v^2} \right) \right\} , \end{aligned}$$

where $m_\Phi ^{}$ and $p_i$ represent the mass of *H*, *A* or $H^\pm$ and the momenta of external Higgs lines, respectively. The deviation from the SM prediction can be ${\mathscr {O}}(100)$ % under the constraint from perturbative unitarity and vacuum stability as well as the current LHC results, in the non-decoupling case $v^2 \sim M^2$. For $M^2 \gg v^2$, such a large quantum effect decouples in the *hhh* coupling because of the decoupling theorem.

**Fig. 71:**
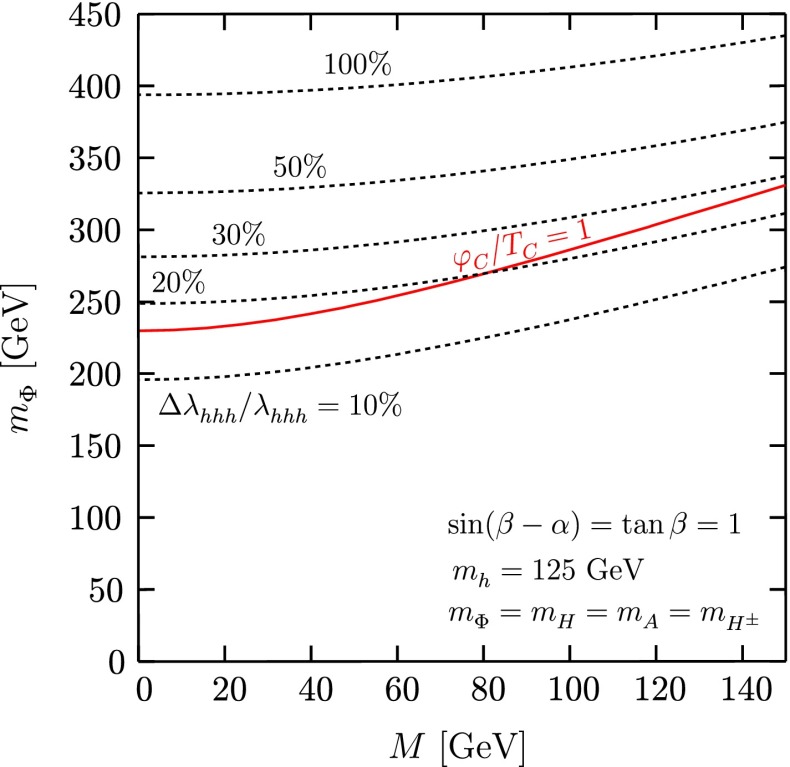
Contour plots of the deviation in the *hhh* coupling in the $(m_\Phi ^{}, M)$ plane for $m_h=125$ GeV and $\sin (\beta -\alpha )=1$. The *red line* indicates $\varphi _c/T_c=1$, above which the strong first order phase transition occurs ($\varphi _c/T_c >1$) [363, 364]

It is well known that such a large non-decoupling loop effect on the triple Higgs-boson coupling is related to the strong first-order phase transition of the electroweak gauge symmetry [369], which is required for successful electroweak baryogenesis [370–373].^18^ In the scenario of electroweak baryogenesis, one of the Sakharov conditions of the departure from thermal equilibrium is satisfied when $\varphi _c/T_c >1$, where $T_c$ is the critical temperature and $\varphi _c$ is the order parameter at $T_c$. With the mass of the discovered Higgs boson to be 125 GeV, the SM cannot satisfy this condition. On the other hand, in the extended Higgs sector, the condition $\varphi _c/T_c >1$ can be satisfied without contradicting the current data. In Fig. 71, the correlation between the large deviation in the *hhh* coupling and the first order phase transition is shown [363, 364, 369]. These results show that we may be able to test the scenario of electroweak baryogenesis by measuring the *hhh* coupling by the 13 % accuracy [339]. Such a precision measurement can be achieved at the ILC.

#### Higgs triplet models

We here discuss the Higgs boson properties in the minimal Higgs triplet model (HTM). A motivation to study this model is that tiny neutrino masses can be explained via the so-called type-II seesaw mechanism [376–380]. The Higgs sector of the HTM is composed of one isospin doublet field $\Phi$ with hypercharge $Y=1$ and the triplet field ${\varDelta }$ with $Y=2$. The Higgs fields can be parameterised by

48 $$\begin{aligned} \Phi= & {} \left[ \begin{array}{c} \phi ^+\\ \textstyle \frac{1}{\sqrt{2}}(\phi +v_\phi +i\chi ) \end{array}\right] ,\quad {\varDelta } = \left[ \begin{array}{c@{\quad }c} \textstyle \frac{{\varDelta }^+}{\sqrt{2}} &{} {\varDelta }^{+\!+}\\ {\varDelta }^0 &{}\textstyle -\frac{{\varDelta }^+}{\sqrt{2}} \end{array}\right] \nonumber \\&\text {with } {\varDelta }^0=\frac{1}{\sqrt{2}}(\delta +v_{\varDelta }+i\eta ), \end{aligned}$$

where $v_\phi$ and $v_{\varDelta }$ are the VEVs of the neutral components of doublet Higgs field $\phi ^0$ and the triplet Higgs field $\delta ^0$, respectively, which satisfy $v^2\equiv v_\phi ^2+2v_{\varDelta }^2\simeq$ (246 GeV)$^2$. The masses of the *W* boson and the *Z* boson are obtained at the tree level as

49 $$\begin{aligned} m_W^2 = \frac{g^2}{4}(v_\phi ^2+2v_{\varDelta }^2),\quad m_Z^2 =\frac{g^2}{4\cos ^2\theta _W}(v_\phi ^2+4v_{\varDelta }^2). \end{aligned}$$

One of the striking features of the HTM is the prediction that the electroweak $\rho$- parameter $\rho$ deviates from unity at the tree level due to the non-zero VEV of the triplet field $v_{\varDelta }$. From Eq. (32), we obtain

50 $$\begin{aligned} \rho \equiv \frac{m_W^2}{m_Z^2\cos ^2\theta _W}=\frac{1+\frac{2v_{\varDelta }^2}{v_\phi ^2}}{1+\frac{4v_{\varDelta }^2}{v_\phi ^2}}. \end{aligned}$$

The experimental value of the $\rho$-parameter is quite close to unity, so that $v_{\varDelta }$ has to be less than about 8 GeV from the tree-level formula given in Eq. (50).

The Yukawa interaction for neutrinos [376–380] is given by

51 $$\begin{aligned} \mathscr {L}_Y=h_{ij}\overline{L_L^{ic}}i\tau _2{\varDelta } L_L^j+\text {h.c.}, \end{aligned}$$

where $h_{ij}$ is the $3\times 3$ complex symmetric Yukawa matrix. Notice that the triplet field ${\varDelta }$ carries a lepton number of $-2$. The mass matrix for the left-handed neutrinos is obtained as

52 $$\begin{aligned} (\mathscr {M}_\nu )_{ij}=\sqrt{2}h_{ij}v_{\varDelta }. \end{aligned}$$

Current neutrino oscillation data can be explained in the HTM [381–394]. It is seen from Eq. (52) that the neutrino mixing pattern is simply determined by the $h_{ij}$ matrix. Since the decay rate of $H^{\pm \pm }$ into the same-sign dilepton is proportional to $|h_{ij}|^2$, the type-II seesaw scenario can be tested by looking at the same-sign dilepton decay mode of $H^{\pm \pm }$ [381–394].

The Higgs potential of the HTM is given by

53 $$\begin{aligned} V(\Phi ,{\varDelta })= & {} m^2\Phi ^\dagger \Phi +M^2\text {Tr}({\varDelta }^\dagger {\varDelta }) +\left[ \mu \Phi ^Ti\tau _2{\varDelta }^\dagger \Phi +\text {h.c.}\right] \nonumber \\&+\,\lambda _1(\Phi ^\dagger \Phi )^2+\lambda _2\left[ \text {Tr}({\varDelta }^\dagger {\varDelta })\right] ^2 +\lambda _3\text {Tr}[({\varDelta }^\dagger {\varDelta })^2] \nonumber \\&+\,\lambda _4(\Phi ^\dagger \Phi )\text {Tr}({\varDelta }^\dagger {\varDelta })+\lambda _5\Phi ^\dagger {\varDelta }{\varDelta }^\dagger \Phi , \end{aligned}$$

where *m* and *M* are the dimension full real parameters, $\mu$ is the dimension full complex parameter which violates the lepton number, and $\lambda _1$–$\lambda _5$ are the coupling constants which are real. We here take $\mu$ to be real.

The potential respects additional global symmetries in some limits. First, there is the global *U*(1) symmetry in the potential in the limit of $\mu = 0$, which conserves the lepton number. As long as we assume that the lepton number is not spontaneously broken, the triplet field does not carry the VEV; i.e., $v_{\varDelta }=0$. Next, an additional global *SU*(2) symmetry appears in the limit where $\mu = \lambda _5 =0$. Under this *SU*(2) symmetry, $\Phi$ and ${\varDelta }$ can be transformed with the different *SU*(2) phases. All the physical triplet-like Higgs bosons are then degenerate in mass.

The mass matrices for the scalar bosons can be diagonalised by rotating the scalar fields as

54 $$\begin{aligned}&\begin{aligned}&\left( \begin{array}{c} \phi ^\pm \\ {\varDelta }^\pm \end{array}\right) = \left( \begin{array}{c@{\quad }c} \cos \beta &{} -\sin \beta \\ \sin \beta &{} \cos \beta \end{array} \right) \left( \begin{array}{c} G^\pm \\ H^\pm \end{array}\right) ,\\&\left( \begin{array}{c} \chi \\ \eta \end{array}\right) = \left( \begin{array}{c@{\quad }c} \cos \beta ' &{} -\sin \beta ' \\ \sin \beta ' &{} \cos \beta ' \end{array} \right) \left( \begin{array}{c} G^0\\ A \end{array}\right) ,\\&\left( \begin{array}{c} \phi \\ \delta \end{array}\right) = \left( \begin{array}{c@{\quad }c} \cos \alpha &{} -\sin \alpha \\ \sin \alpha &{} \cos \alpha \end{array} \right) \left( \begin{array}{c} h\\ H \end{array}\right) , \end{aligned} \end{aligned}$$

with the mixing angles

55 $$\begin{aligned}&\tan \beta =\frac{\sqrt{2}v_{\varDelta }}{v_\phi },\quad \tan \beta ' = \frac{2v_{\varDelta }}{v_\phi }, \nonumber \\&\tan 2\alpha =\frac{v_{\varDelta }}{v_\phi }\frac{2v_\phi ^2(\lambda _4+\lambda _5)-4M_{\varDelta }^2}{2v_\phi ^2\lambda _1-M_{\varDelta }^2-2v_{\varDelta }^2(\lambda _2+\lambda _3)}. \end{aligned}$$

In addition to the three Nambu–Goldstone bosons $G^\pm$ and $G^0$ which are absorbed by the longitudinal components of the *W* boson and the *Z* boson, there are seven physical mass eigenstates; i.e., a pair of doubly charged (singly charged) Higgs bosons $H^{\pm \pm }$ ($H^\pm$), a ${\textit{CP}}$-odd Higgs boson *A* and ${\textit{CP}}$-even Higgs boson *H* and *h*, where *h* is taken as the SM-like Higgs boson. The six parameters $\mu$ and $\lambda _1$–$\lambda _5$ in the Higgs potential in Eq. (53) can be written in terms of the physical scalar masses, the mixing angle $\alpha$ and VEVs $v_\phi$ and $v_{\varDelta }$.

As required by the $\rho$- parameter data, when the triplet VEV $v_{\varDelta }$ is much less than the doublet VEV $v_\phi$, there is relationships among the masses of the triplet-like Higgs bosons by neglecting $\mathscr {O}(v_{\varDelta }^2/v_\phi ^2)$ terms as

56 $$\begin{aligned}&m_{H^{+\!+}}^2-m_{H^{+}}^2=m_{H^{+}}^2-m_{A}^2~~\left( =-\frac{\lambda _5}{4}v^2\right) , \end{aligned}$$

57 $$\begin{aligned}&m_A^2=m_{H}^2~~(=M_{\varDelta }^2). \end{aligned}$$

In the limit of $v_{\varDelta }/v_\phi \rightarrow 0$, the four mass parameters of the triplet-like Higgs bosons are determined by two parameters. Eqs. (56) and (57) can be regarded as the consequence of the global symmetries mentioned above.

**Fig. 72:**
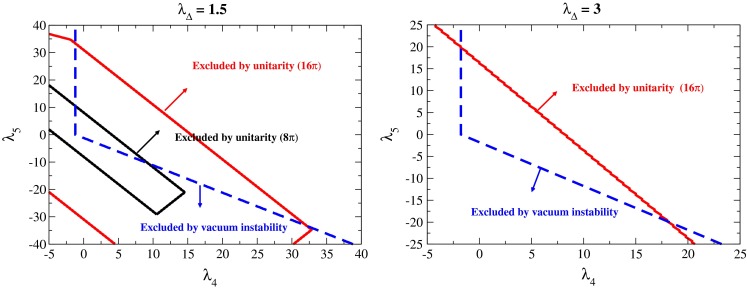
Constraints from the unitarity and vacuum stability bounds for $\lambda _1=m_h^2/(2v^2)\simeq 0.13$ in the $\lambda _4$–$\lambda _5$ plane. We take $\lambda _{\varDelta }=1.5$ for the *left panel* and $\lambda _{\varDelta }=3$ for the *right panel* with $\lambda _{\varDelta }=\lambda _2=\lambda _3$ [375]

**Fig. 73:**
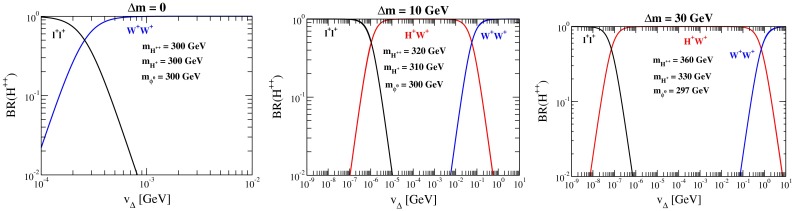
Decay branching ratio of $H^{+\!+}$ as a function of $v_{\varDelta }$. In the *left figure*, $m_{H^{+\!+}}$ is fixed to be 300 GeV, and ${\varDelta } m$ is taken to be zero. In the *middle figure*, $m_{H^{+\!+}}$ is fixed to be 320 GeV, and ${\varDelta } m$ is taken to be 10 GeV. In the *right figure*, $m_{H^{+\!+}}$ is fixed to be 360 GeV, and ${\varDelta } m$ is taken to be 30 GeV

The condition for the vacuum stability bound has been derived in Ref. [395], where we require that the Higgs potential is bounded from below in any direction of the large scalar fields region. The unitarity bound in the HTM has been discussed in Ref. [395]. In Fig. 72, the excluded regions by the unitarity bound and the vacuum stability condition are shown for $\lambda _1 = m_h^2/(2v^2)\simeq 0.13$ in the $\lambda _4$–$\lambda _5$ plane [375]. We take $\lambda _{\varDelta }=1.5$ (3) in the left (right) panel. Excluded regions by the unitarity and vacuum stability bounds are shown.

**Fig. 74:**
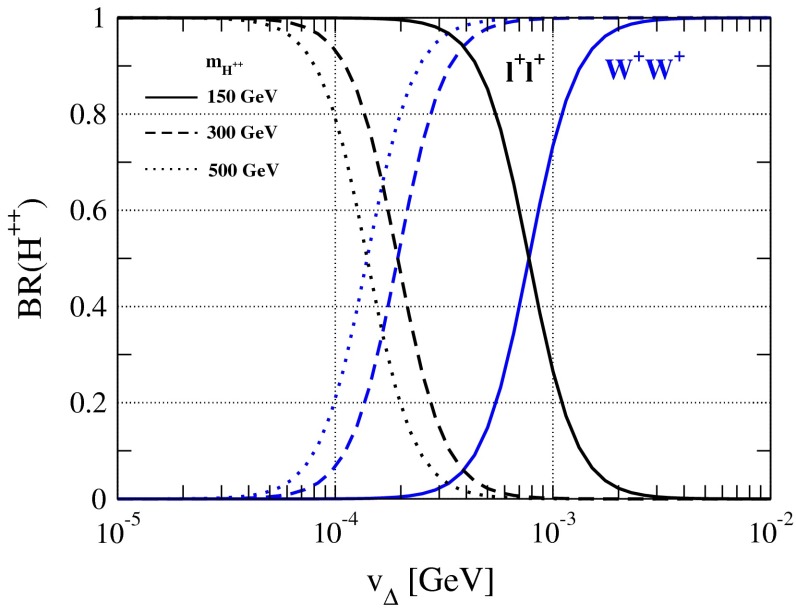
Decay branching ratio of $H^{+\!+}$ as a function of $v_{\varDelta }$ with $m_{H^+}=m_{H^{+\!+}}$. The *solid*, *dashed* and *dotted curves*, respectively, show the results in the case of $m_{H^{+\!+}}=150$, 300 and 500 GeV [375]

The most interesting feature of the HTM is the existence of doubly charged Higgs bosons $H^{\pm \pm }$. Their discovery at colliders can be a direct probe of the exotic Higgs sectors. The doubly charged Higgs bosons $H^{\pm \pm }$ can decay into $\ell ^\pm \ell ^\pm$, $H^\pm W^\pm$ and $W^\pm W^\pm$ depending on the magnitude of $v_{\varDelta }$ [396]. In Fig. 73, the branching ratios are shown as a function of the vacuum expectation value of the triplet field, $v_{\varDelta }$, for the cases with the mass difference ${\varDelta } m=m_{H^{+\!+}}-m_{H^+}=0$, 10 and 30 GeV [397]. The decay branching ratio of $H^{\pm \pm }$ is shown in Fig. 74 assuming all the elements in $(M_\nu )_{ij}$ to be 0.1 eV. The dominant decay mode changes from the same-sign dilepton mode to the same-sign diboson mode at $v_{\varDelta }=0.1$–1 MeV.

When the triplet-like Higgs bosons are degenerate in mass or $H^{\pm \pm }$ is the lightest of all of them, the main decay mode of $H^{\pm \pm }$ is the same-sign dilepton (diboson) in the case where $v_{\varDelta }$ is less (larger) than about 1 MeV. The signal directly shows the existence of the doubly charged scalar boson with lepton number 2, which can be a strong evidence for the neutrino mass generation via Eq. (51). At the LHC, $H^{\pm \pm }$ are produced by the Drell–Yan process $pp\rightarrow Z^*/\gamma ^* \rightarrow H^{+\!+}H^{--}$ and the associated process $pp\rightarrow W^* \rightarrow H^{\pm \pm }H^{\mp }$. The search for $H^{\pm \pm }$ in the dilepton decay scenario has been performed at the LHC. The scenario based on the same-sign dilepton decay of $H^{\pm \pm }$ has been studied in Refs. [381–394]. The strongest lower limit on $m_{H^{+\!+}}$ has been given by 459 GeV [398] at the 95 % CL assuming the 100 % decay of $H^{\pm \pm }\rightarrow \mu ^\pm \mu ^\pm$ from the 7 TeV and 4.9 fb$^{-1}$ data. This bound becomes weaker as 395 GeV [398] when we only use the pair production process. However, when $H^{\pm \pm }$ mainly decay into the same-sign diboson, this bound can no longer be applied.

**Fig. 75:**
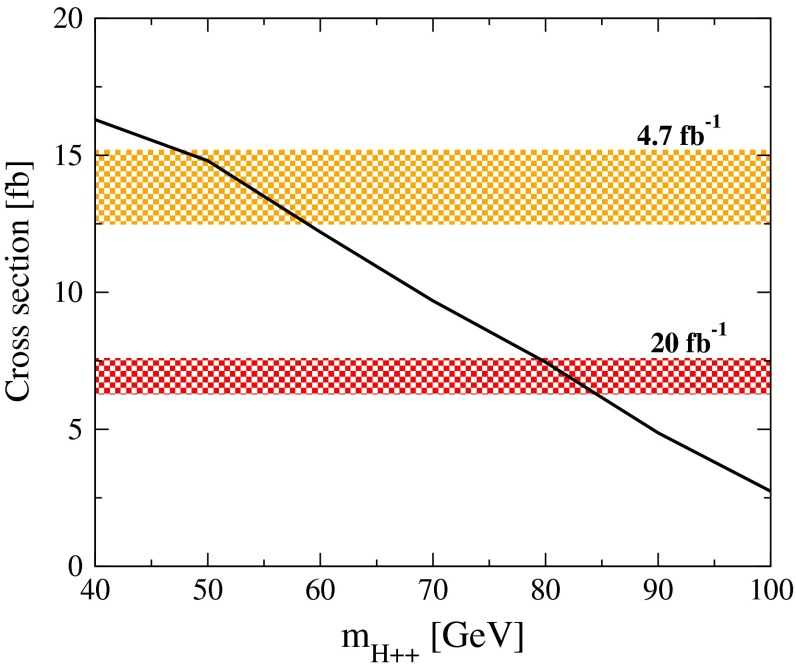
The signal cross section as a function of $m_{H^{+\!+}}$ with the collision energy to be 7 TeV from Ref. [399]. The *light* (*dark*) *shaded band* shows the 95 % CL (expected) upper bound for the cross section from the data with the integrate luminosity to be 4.7 fb$^{-1}$ (20 fb$^{-1}$)

When $v_{\varDelta }$ is sufficiently larger than $10^{-3}$ GeV, the diboson decay $H^{\pm \pm } \rightarrow W^\pm W^\pm$ becomes dominant. In this case, the signal can also be of four same-sign leptons, but its rate is reduced by the branching ratios of leptonic decays of *W*s. The scenario for the same-sign diboson decay of $H^{\pm \pm }$ has been discussed in Refs. [390, 391, 400]. The discovery potential of $H^{\pm \pm }$ at the LHC has also been investigated in Ref. [400] in the HTM and also the Georgi–Machacek model [278]. In Ref. [399], the lower bound on $m_{H^{+\!+}}$ has been obtained by using the same-sign dilepton event measured at the LHC with 7 TeV and 4.7 fb$^{-1}$ data [401]. In Fig. 75, the sum of the cross sections of the processes

58 $$\begin{aligned}&pp\rightarrow H^{+\!+}H^{--} \rightarrow W^{+(*)}W^{+(*)}H^{--}\rightarrow \mu ^+\mu ^+ E_{\text {miss}}H^{--},\nonumber \\&pp\rightarrow H^{+\!+}H^{-} \rightarrow W^{+(*)}W^{+(*)}H^{-}\rightarrow \mu ^+\mu ^+ E_{\text {miss}}H^{-}, \end{aligned}$$

are shown as a function of $m_{H^{+\!+}}$ assuming $m_{H^+}=m_{H^{+\!+}}$. We can see that $m_{H^{+\!+}}$ smaller than about 60 GeV is excluded at the 95 % CL. The bound is much relaxed as compared to that in the dilepton decay scenario. By the extrapolation of the data to 20 fb$^{-1}$ with the same collision energy, the lower limit is obtained as 85 GeV. Therefore, a light $H^{\pm \pm }$ such as around 100 GeV is still allowed by the current data at the LHC, and in this case the ILC may be able to discover the doubly charged Higgs boson. See also recent progress in Ref. [402].

**Fig. 76:**
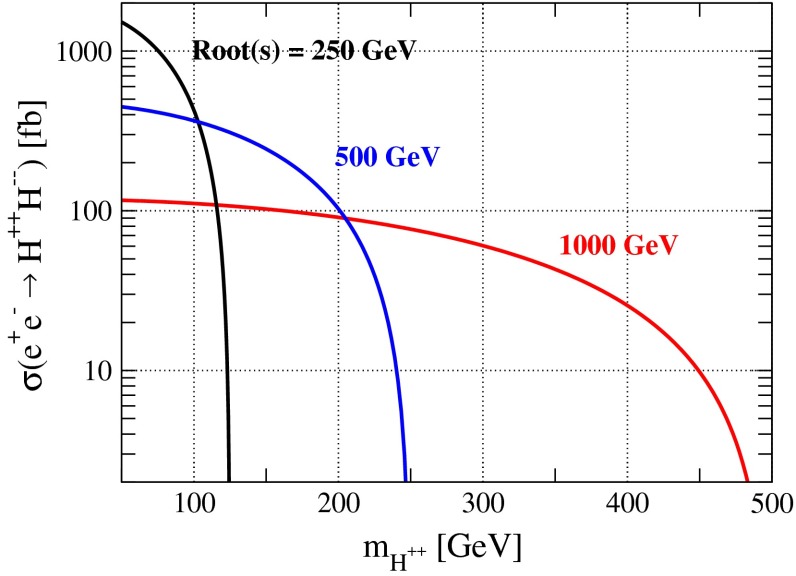
Production cross section of the $e^+e^-\rightarrow H^{+\!+}H^{--}$ process as a function of $m_{H^{+\!+}}$. The *black*, *blue* and *red curves* are, respectively, the results with the collision energy $\sqrt{s}=$250, 500 and 1000 GeV

**Fig. 77:**
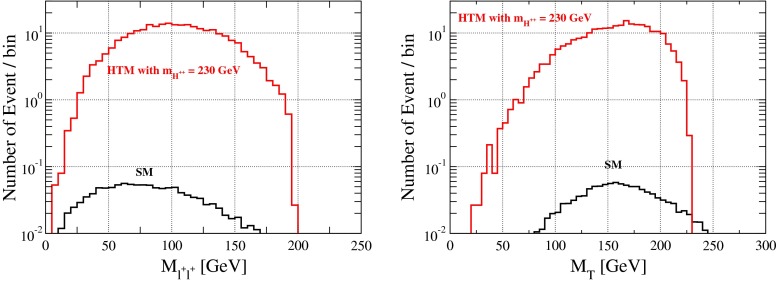
The invariant mass distribution (*left panel*) and the transverse mass distribution (*right panel*) for the $\ell ^+\ell ^+$ and $\ell ^+\ell ^+E_{\text {miss}}$ systems, respectively, in the case of $m_{H^{+\!+}}=230$ GeV and $\sqrt{s}=500$ GeV [375]. The integrated luminosity is assumed to be 500 fb$^{-1}$

At the ILC, doubly charged Higgs bosons are produced via the pair production $e^+e^-\rightarrow H^{+\!+}H^{--}$. In the diboson decay scenario, the final state is the same-sign dilepton, missing energy and multi-jets; i.e., $e^+e^-\rightarrow H^{+\!+}H^{--}\rightarrow \ell ^+\ell ^+ E_\text {miss}jjjj$, where $\ell =e,\mu$ [375]. The background comes from the four *W* bosons production; i.e., $e^+e^-\rightarrow W^+W^+W^-W^-\rightarrow \ell ^\pm \ell ^\pm E_\text {miss}jjjj$. For example, when $\sqrt{s}=500$ GeV and the $m_{H^{+\!+}}=230$ GeV is taken, the signal (background) cross section of the final-state $\ell ^\pm \ell ^\pm E_\text {miss}4j$ is obtained to be 1.07 fb (2.37$\times 10^{-3}$ fb) (Fig. 76) [375]. The above numbers are obtained after taking the following basic kinematic cuts:

59 $$\begin{aligned} p_T^\ell \ge 15 ~\text {GeV},\quad |\eta ^\ell | \le 2.5, \end{aligned}$$

where $p_T^\ell$ and $\eta ^\ell$ are the transverse momentum and pseudo rapidity for $\ell$, respectively. Therefore, this process is almost background free. In Fig. 77, the invariant mass $M_{\ell ^+\ell ^+}$ for the $\ell ^+\ell ^+$ system (left panel) and the transverse mass $M_T$ (right panel) distributions for $\ell ^+\ell ^+E_{\text {miss}}$ system are shown. The red and black curves denote the distribution from the signal and background, respectively. Around 230 GeV, there is an endpoint in the $M_T$ distribution that corresponds to $m_{H^{+\!+}}$. The $M_T$ distribution is useful to measure $m_{H^{+\!+}}$.

If the triplet-like Higgs bosons are light enough, the direct detection of them at the LHC and the ILC is the most important probe of the HTM as already discussed. On the other hand, they can also be indirectly tested by measuring the deviations from the SM in the Higgs-boson couplings for the SM-like Higgs boson *h*, such as the coupling constants with the weak gauge bosons *hVV*, the Yukawa couplings $hf\bar{f}$ and the triple Higgs-boson coupling *hhh*, where *V* represents gauge bosons, and *f* does quarks and leptons. The indirect searches can be useful even when no new particles is directly found. At the ILC, the Higgs-boson couplings are expected to be precisely measured. For example, the Higgs-boson couplings with the weak gauge bosons (*hZZ* and *hWW*) and the Yukawa couplings ($hb\bar{b}$, $h\tau \bar{\tau }$ and $ht\bar{t}$) are expected to be measured with $\mathscr {O}(1)~\%$ accuracy [268, 339, 360, 403–407]. In the HTM, the loop induced $h\gamma \gamma$ coupling has been calculated in Refs. [408–412]. The one-loop corrections to the *hWW*, *hZZ* and *hhh* vertices have also been calculated in Refs. [413, 414]. In Ref. [414], it has been found that there is a correlation among the deviation in the Higgs-boson couplings. For example, when the decay rate of $h\rightarrow \gamma \gamma$ deviates by 30 % (40 %) from the SM prediction, deviations in the one-loop corrected *hVV* and *hhh* vertices are predicted to be about $-0.1~\%$ ($-2~\%$) and $-10~\%$ ($150~\%$), respectively.^19^ By comparing these deviations with the precisely measured value at the ILC, we can discriminate the HTM from the other models.

**Table 22:** The fraction of the VEVs $\tan \beta$ and the scaling factors $\kappa _f$ and $\kappa _V$ in the extended Higgs sectors with universal Yukawa couplings [340]

	$\tan \beta$	$\kappa _f$	$\kappa _V^{}$
Doublet-singlet model	–	$\cos \alpha$	$\cos \alpha$
Type-I THDM	$v_0/v_\text {ext}^{}$	$\cos \alpha /\sin \beta =\sin (\beta -\alpha )+\cot \beta \cos (\beta -\alpha )$	$\sin (\beta -\alpha )$
GM model	$v_0/(2\sqrt{2}v_\text {ext}^{})$	$\cos \alpha /\sin \beta$	$\sin \beta \cos \alpha -\tfrac{2\sqrt{6}}{3} \cos \beta \sin \alpha$
Doublet-septet model	$v_0/(4v_\text {ext}^{})$	$\cos \alpha /\sin \beta$	$\sin \beta \cos \alpha -4 \cos \beta \sin \alpha$

#### Other exotic models

Precision measurements for the couplings of the SM-like Higgs boson *h* at the ILC can also discriminate exotic Higgs sectors. According to Refs. [339, 340], we here consider various extended Higgs sectors which satisfy $\rho =1$ at the tree level; i.e., the model with an additional singlet scalar field with $Y=0$, the 2HDM (Type I), the model with a septet scalar field with $Y=4$ [415, 416], and the Georgi–Machacek model where a complex ($Y=2$) and a real ($Y=0$) triplet scalar fields are added to the SM-like Higgs doublet [278]. In these models, all quark and leptons receive their masses from only one scalar doublet. Consequently, the Yukawa coupling constants with respect to the SM-like Higgs boson $h \bar{f} f$ from the SM values are commonly suppressed due to the mixing between the two (or more) neutral states. In a, we have a universal suppression on the coupling constants, $\kappa _F^{} = \kappa _V^{} = \cos \theta$ with $\theta$ being the mixing angle between the doublet field and the singlet field. However, $\kappa _F^{} \ne \kappa _V^{}$ is usually predicted in more complicated Higgs sectors such as the 2HDM (Type I), the Georgi–Machacek model [278] and the doublet–septet model [415, 416]. Notice that in exotic models with higher representation scalar fields such as the Georgi–Machacek model and doublet–septet model, $\kappa _V$ can be greater than 1. This can be a signature of exotic Higgs sectors. From Eq. (32), a VEV from these additional scalar multiplets do not change $\rho =1$ at the tree level. All the VEVs $v_\text {ext}$ of these additional Higgs multiplets except for that of the singlet partially contribute to the spontaneous breaking of the electroweak gauge symmetry. The VEVs satisfy $v^2= v_0^2 + (\eta _\text {ext}\, v_\text {ext})^2$, where $v_\phi$ is the VEV of the SM-like Higgs doublet $\Phi$ and $\eta _{\text {ext}}$ = 1 and 4 in the Type-I THDM and the model with the septet, respectively. It is convenient to define the ratio of the VEVs as $\tan \beta = v_0/(\eta _\text {ext}\, v_\text {ext})$ [340]. In Table 22, the scaling factors $\kappa _f$ and $\kappa _V$ are listed in terms of $\alpha$ and $\beta$ in the four models.

**Fig. 78:**
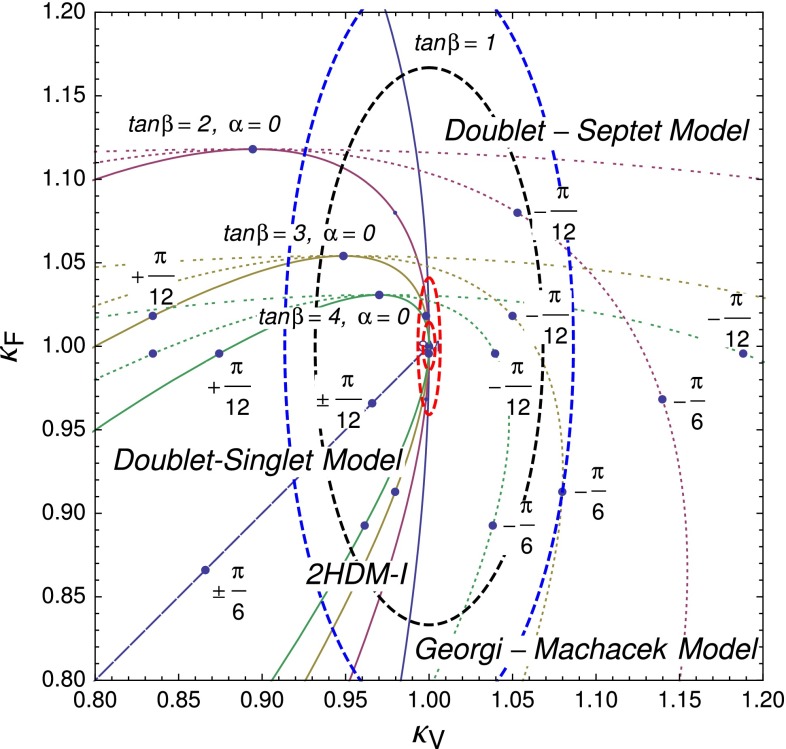
The scaling factors in models with universal Yukawa couplings. The current LHC bounds and the expected LHC and ILC sensitivities are also shown at the 68.27 % CL. For details, see Ref. [340]

In Fig. 78, the predictions for the scale factors of the universal Yukawa coupling $\kappa _F$ and the gauge coupling $\kappa _V$ are plotted in exotic Higgs sectors for each set of mixing angles. The current LHC bounds, expected LHC and ILC sensitivities for $\kappa _F$ and $\kappa _V$ are also shown at the 68.27 % CL. Therefore, exotic Higgs sectors can be discriminated by measuring $\kappa _V$ and $\kappa _F$ precisely. For details, see Refs. [339, 340].

#### Summary

Although the Higgs boson with the mass 125 GeV was found at the LHC, knowledge about the structure of the Higgs sector is very limited. Since there are no theoretical principles for the minimal Higgs sector with one Higgs doublet, there are many possibilities of non-minimal Higgs sectors. Such extended Higgs sectors appear in many new physics models beyond the SM. Therefore, the Higgs sector is a window to new physics, and we can explore new physics from clarifying the structure of the Higgs sector by coming collider experiments. At the LHC, direct discovery of additional Higgs bosons can be expected as long as they are not too heavy. On the other hand, the Higgs sector can also be explored by precisely measuring the properties of the discovered Higgs boson *h* accurately. The precision measurements will be performed partially at the high luminosiity LHC with 3000 fb$^{-1}$. Using the high ability of the ILC for measuring the Higgs-boson couplings, we can further test extended Higgs sectors, and consequently narrow down the new physics models.

### Higgs physics in strong-interaction scenarios^20^

The Higgs mechanism [1–4], which has been introduced to provide masses for the fermions and gauge bosons without violating gauge principles, can describe EWSB but fails to explain it. Within the SM there is no dynamics leading to the typical mexican hat shape of the Higgs potential. Moreover, in order to keep the Higgs-boson mass at the experimentally measured value of 125 GeV [62, 94] in the presence of high scales at which the SM will eventually has to be amended, a substantial amount of fine tuning is necessary unless the mass is protected from higher order corrections due to some symmetry. Such a symmetry must act non-linearly on the Higgs field. Besides supersymmetry a prominent example is given by a global symmetry when the Higgs boson appears as a pseudo Nambu–Goldstone boson. A Higgs boson is needed to ensure the proper decoupling of the longitudinal polarisations of the massive EW gauge bosons at high energy. Indeed, these longitudinal modes of $W^\pm$ and *Z* can be described by Nambu–Goldstone bosons associated to the coset $SU(2)_L\times SU(2)_R / SU(2)_{\mathrm{isospin}}$. Their kinetic term corresponds to the gauge boson mass terms,

60 $$\begin{aligned} \frac{1}{2}m_Z^2 Z_\mu Z^\mu + m_W^2 W_{\mu }^+ W^{-\mu }= \frac{v^2}{4} \text{ Tr } ( D_\mu \Sigma ^\dagger D^\mu \Sigma ) \end{aligned}$$

with $\Sigma = e^{i\sigma ^a \pi ^a/v}$, where $\sigma ^a$ ($a=1,2,3$) are the usual Pauli matrices. Due to the Goldstone boson equivalence theorem the non-trivial scattering of the longitudinal gauge bosons *V* ($V=W^\pm ,Z$) is controlled by the contact interactions among four pions from the expansion of the Lagrangian Eq. (60), leading to amplitudes growing with the energy,

61 $$\begin{aligned} {\mathscr {A}} (V^a_L V^b_L \rightarrow V^c_L V^d_L)= & {} {\mathscr {A}}(s) \delta ^{ab} \delta ^{cd} + {\mathscr {A}} (t) \delta ^{ac} \delta ^{bd} \nonumber \\&+\, {\mathscr {A}} (u) \delta ^{ad} \delta ^{bc} \quad \text{ with } \quad {\mathscr {A}} (s) \approx \frac{s}{v^2}. \end{aligned}$$

Here *s*, *t*, *u* denote the Mandelstam variables, and *v* represents the vacuum expectation value (VEV) with $v \approx 246$ GeV. The amplitude grows with the centre-of-mass (c.m.) energy squared *s*, and therefore perturbative unitarity will be lost around $4 \pi v \sim 3$ TeV, unless there is a new weakly coupled elementary degree of freedom. The simplest realisation of new dynamics restoring perturbative unitarity is given by a single scalar field *h*, which is singlet under $SU(2)_L\times SU(2)_R / SU(2)_{ \text{ isospin }}$ and couples to the longitudinal gauge bosons and fermions as [417–419],

62 $$\begin{aligned}&{\mathscr {L}}_{EWSB}= \frac{1}{2} (\partial _\mu h)^2 - V(h) + \frac{v^2}{4} \,\text{ Tr } (D_\mu \Sigma ^\dagger D^\mu \Sigma )\nonumber \\&\qquad \times \left( 1+2a \frac{h}{v} + b \frac{h^2}{v^2} + \sum _{n\ge 3} b_n \frac{h^n}{v^n}+\ldots \right) \nonumber \\&\qquad - \frac{v}{\sqrt{2}} (\bar{u}^i_L \bar{d}^i_L) \Sigma \left( 1 + c \frac{h}{v} + \sum _{n\ge 2}c_n \frac{h^n}{v^n} + \cdots \right) \left( \begin{array}{c} y^u_{ij} u^j_R \\ y^d_{ij} d^j_R \end{array}\right) \\&\qquad +\,\mathrm{h.c.} \end{aligned}$$

with

63 $$\begin{aligned} V(h)= & {} \frac{1}{2} m_h^2 h^2 + \frac{d_3}{6} \left( \frac{3m_h^2}{v}\right) h^3 + \frac{d_4}{24} \left( \frac{3m_h^2}{v^2}\right) h^4 + \cdots \end{aligned}$$

For $a=1$ the scalar exchange cancels the piece growing with the energy in the $V_L V_L$ amplitude. If in addition $b=a^2$ then also in the inelastic amplitude $V_L V_L \rightarrow hh$ unitarity is maintained, while for $ac=1$ the $V_L V_L \rightarrow f \bar{f}'$ amplitude remains finite. The SM Higgs boson is defined by the point $a=b=c=1$ and $d_3=d_4=1$, $c_{n\ge 2}=b_{n\ge 3}=0$. The scalar resonance and the pions then combine to form a doublet which transforms linearly under $SU(2)_L \times SU(2)_R$. The Lagrangian Eq. (62) describes either an elementary or a composite Higgs boson. For $a\ne 1$ the Higgs boson alone cannot fully unitarise the $V_L V_L$ scattering, with the breakdown of perturbative unitarity pushed to a higher scale now, which is of the order $4\pi v/ \sqrt{1-a^2}$. The residual growth of the scattering amplitude ${\mathscr {A}}(s) \approx (1-a^2) s/v^2$ will finally be cancelled by the exchange of other degrees of freedom. The Lagrangian Eqs. (62), (63) introduces deviations in the Higgs boson phenomenology [417, 420] away from the SM point by rescaling all Higgs couplings through the modifiers *a*, *b* and *c*,

64 $$\begin{aligned} g_{hVV} = a g_{hVV}^{\mathrm{SM}} ,\quad g_{hhVV}= b g_{hhVV}^{\mathrm{SM}} , \quad g_{hf\bar{f}'} = c g_{hf \bar{f}'}^{\mathrm{SM}} , \end{aligned}$$

while keeping the same Lorentz structure. With *c* being flavour-universal, minimal flavour violation is built in and the usual SM Yukawa couplings are the only source of flavour violation. There are additional new couplings as, e.g., the $c_2$ coupling between two Higgs bosons and two fermions, which contributes to multi-Higgs production [417–419].

In composite Higgs models, the deviations from the SM point $a=b=1$ are controlled by the ratio of the weak scale over the compositeness scale *f*. In these models the Higgs boson is a composite bound state which emerges from a strongly interacting sector [421–426]. The good agreement with the electroweak precision data is achieved by a mass gap that separates the Higgs scalar from the other resonances of the strong sector. This mass gap arises dynamically in a natural way if the strongly interacting sector has a global symmetry *G*, which is spontaneously broken at a scale *f* to a subgroup *H* so that the coset *G* / *H* contains a fourth Nambu–Goldstone boson which is identified with the Higgs boson. Composite Higgs models can be viewed as a continuous interpolation between the SM and technicolour type models. With the compositeness scale of the Higgs boson given by the dynamical scale *f*, the limit $\xi \equiv v^2/f^2 \rightarrow 0$ corresponds to the SM where the Higgs boson appears as an elementary light particle and the other resonances of the strong sector decouple. In the limit $\xi \rightarrow 1$ the Higgs boson does not couple to the $V_L$ any longer and other (heavy) resonances are necessary to ensure unitarity in the gauge boson scattering. The $\xi \rightarrow 1$ limit corresponds to the technicolour paradigm [90, 91] where the strong dynamics directly breaks the electroweak symmetry down to the electromagnetism subgroup.

#### Effective Lagrangian and Higgs couplings

Independently of its dynamical origin, the physics of a strongly interacting light Higgs (SILH) boson can be captured in a model-independent way by an effective Lagrangian which involves two classes of higher-dimensional operators: (1) those being genuinely sensitive to the new strong force and which will qualitatively affect the Higgs boson phenomenology and (2) those being sensitive only to the spectrum of the resonances and which will simply act as form factors. The size of the various operators is controlled by simple rules and the effective Lagrangian can be cast into the generic form [417]

65 $$\begin{aligned} \mathscr {L}_\mathrm{SILH}= & {} \frac{c_H}{2f^2} \left( \partial _\mu |H|^2 \right) ^2 + \frac{c_T}{2f^2} \left( H^\dagger {\overleftrightarrow { D^\mu }} H\right) ^2\nonumber \\&- \frac{c_6\lambda }{f^2} |H|^6 + \left( \frac{c_yy_f}{f^2} |H|^2 {\bar{f}}_L Hf_R +\mathrm{h.c.}\right) \nonumber \\&+\frac{ic_Wg}{2m_\rho ^2}\left( H^\dagger \sigma ^i {\overleftrightarrow { D^\mu }} H \right) ( D^\nu W_{\mu \nu })^i\nonumber \\&+\frac{ic_Bg'}{2m_\rho ^2}\left( H^\dagger {\overleftrightarrow { D^\mu }} H \right) ( \partial ^\nu B_{\mu \nu }) +\cdots \end{aligned}$$

with the SM electroweak (EW) couplings $g,g'$, the SM Higgs quartic coupling $\lambda$ and the SM Yukawa coupling $y_f$ to the fermions $f_{L,R}$. The coefficients in Eq. (65) are expected to be of order 1 unless protected by some symmetry. The SILH Lagrangian gives rise to oblique corrections at tree level. The coefficient $c_T$ vanishes in case the strong sector is assumed to respect custodial symmetry. The form-factor operators induce a contribution to the $\hat{S}$ parameter, $\hat{S} = (c_W + c_B)m_W^2/m_\rho ^2$, where $m_\rho$ denotes the mass scale of the heavy strong sector resonances, which imposes a lower bound $m_\rho \ge 2.5$ TeV. Since the Higgs couplings to the SM vector bosons receive corrections of the order $v^2/f^2$ the cancellation between the Higgs and the gauge boson contributions taking place in the SM, is spoiled and the $\hat{S}$ and $\hat{T}$ parameters become logarithmically divergent [427] when all the low energy degrees of freedom are considered. This infrared (IR) contribution imposes an upper bound of $\xi \lesssim ~0.1$ [428–431] which can be relaxed by a factor of 2 if a partial cancellation of ${\mathscr {O}}(50~\%)$ with contributions from other states is allowed. Light top partners, as required to generate the Higgs mass, also contribute to the EW oblique parameters and can change the range of value of $\xi$ preferred by EW precision data [432]. The Higgs kinetic term, which receives a correction from the operator $c_H$, can be brought back to its canonical form by rescaling the Higgs field. This induces in the Higgs couplings a universal shift by a factor $1-c_H \xi /2$. For the fermions, it adds up to the modified Yukawa interactions.

The effective Lagrangian Eq. (65) represents the first term in an expansion in $\xi = v^2/f^2$. For large values of $\xi \sim {\mathscr {O}}(1)$ the series has to be resummed, examples of which have been given in explicit models such as those constructed in 5D warped space based on the coset *SO*(5) / *SO*(4) [433–435]. In the MCHM4 [434], where the SM fermions transform as spinorial representations of *SO*(5), all SM Higgs couplings are suppressed by the same modification factor as a function of $\xi$, so that the branching ratios are unchanged and only the total width is affected. In the MCHM5 [435] with the fermions in the fundamental representation of *SO*(5) on the other hand the Higgs couplings to gauge bosons and to fermions are modified differently inducing non-trivial changes both in the branching ratios and the total width. The relations between the couplings in the effective Lagrangian Eq. (62), the SILH Lagrangian Eq. (65) and the MCHM4 and MCHM5 models is summarised in Table 23, see also Refs. [436–439].

**Table 23:** Higgs coupling values of the effective Lagrangian Eq. (62), in the SILH set-up Eq. (65) and in explicit SO(5)/SO(4) composite Higgs models built in warped 5D space-time, MHCM4 and MHCM5. From Ref. [93]

Parameters	SILH	MCHM4	MCHM5
*a*	$1-c_H\xi /2$	$\sqrt{1-\xi }$	$\sqrt{1-\xi }$
*b*	$1-2 c_H \xi$	$1-2\xi$	$1-2\xi$
$b_3$	$-\frac{4}{3}\xi$	$-\frac{4}{3} \xi \sqrt{1-\xi }$	$-\frac{4}{3} \xi \sqrt{1-\xi }$
*c*	$1-(c_H/2+c_y) \xi$	$\sqrt{1-\xi }$	$\frac{1-2\xi }{\sqrt{1-\xi }}$
$c_2$	$-(c_H+3c_y)\xi /2$	$-\xi /2$	$-2\xi$
$d_3$	$1+(c_6 - 3 c_H/2) \xi$	$\sqrt{1-\xi }$	$\frac{1-2\xi }{\sqrt{1-\xi }}$
$d_4$	$1+(6 c_6 - 25 c_H/3) \xi$	$1-7 \xi /3$	$\frac{1-28\xi (1-\xi )/3}{1-\xi }$

**Fig. 79:**
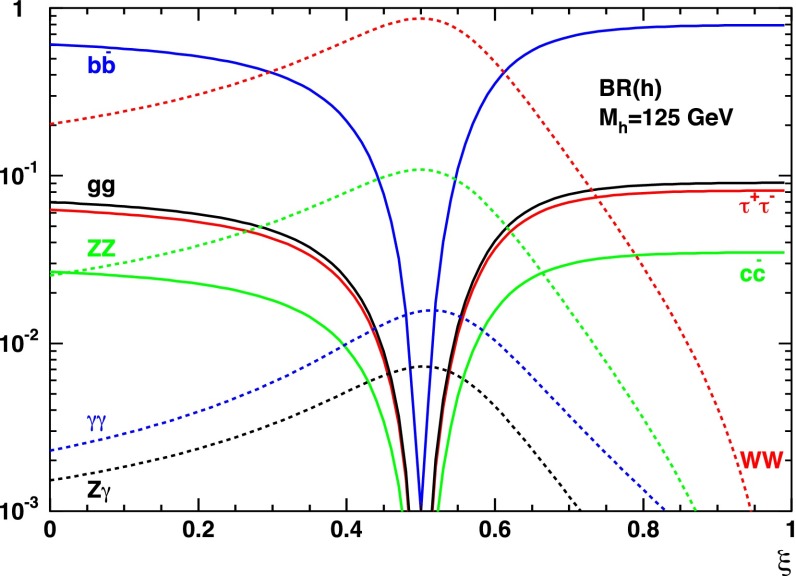
Higgs boson branching ratios in MCHM5 as a function of $\xi$ for $M_h=125$ GeV

The Higgs anomalous couplings affect both the Higgs production and decay processes. The Higgs boson branching ratios of a 125 GeV Higgs boson are shown in Fig. 79 for MCHM5. For $\xi =0.5$ the Higgs boson becomes fermiophobic and the branching ratios into fermions and gluons vanish, while the ones into gauge bosons become enhanced. As explained above, in MCHM4 the branching ratios are unchanged. The modified production cross sections can easily be obtained from the corresponding SM results by rescaling with the appropriate coupling modification factors squared. As the QCD couplings are not affected the higher order QCD corrections can be taken over from the SM, while the EW corrections would change and have to be omitted as they are not available so far.

The anomalous couplings can be tested by a measurement of the Higgs interaction strengths. In case of a universal coupling modification as, e.g., in MCHM4 the production rates and the total width have to be tested. At an $e^+e^-$ linear collider an accuracy of a few per-cent can be achieved in the measurement of the SM Higgs couplings to gauge bosons and fermions [56]. For an investigation of the prospects for the determination of $\xi$ at the LHC, see Ref. [440]. In Ref. [367] a study of Higgs couplings performed in the context of genuine dimension-six operators showed that a sensitivity of up to $4\pi f \sim 40$ TeV can be reached for a 120 GeV Higgs boson already at 500 GeV with $1ab^{-1}$ integrated luminosity. At the high-energy phase of the CLIC project, i.e., at 3 TeV with $2ab^{-1}$ integrated luminosity, the compositeness scale of the Higgs boson will be probed up to 60–90 TeV [441]. Also the total width of a 125 GeV Higgs boson can be measured at a few per-cent precisely already at the low-energy phase of the ILC programme.

#### Strong processes

If no new particles are discovered at the LHC, deviations from the SM predictions for production and decay rates can point towards models with strong dynamics. It is, however, only the characteristic signals of a composite Higgs boson in the high-energy region which unambiguously imply the existence of new strong interactions. Since in the composite Higgs scenario the $V_L V_L$ scattering amplitude is not fully unitarised the related interaction necessarily becomes strong and eventually fails tree-level unitarity at the cutoff scale. The *VV* scattering therefore becomes strong at high energies. As the transversely polarised vector boson scattering is numerically large in the SM, the test of the energy growth in longitudinal gauge boson scattering is difficult at the LHC [418]. Another probe of the strong dynamics at the origin of EWSB is provided by longitudinal vector boson fusion in Higgs pairs which also grows with the energy. For the test of strong double Higgs production the high-luminosity upgrade of the LHC would be needed, however [418]. Besides testing the high-energy behaviour in strong double Higgs production, new resocances unitarising the scattering amplitudes can be searched for. The ILC has been shown to be able to test anomalous strong gauge couplings up to a scale $\sim$3 TeV and exclude $\rho$-like resonances below 2.5 TeV [56].

#### Non-linear Higgs couplings

**Fig. 80:**
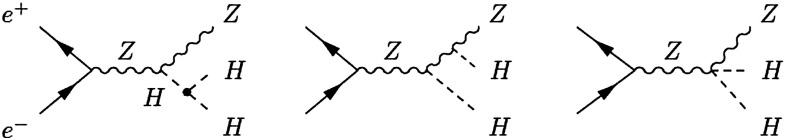
Generic Feynman diagrams contributing to Higgs pair production via Higgs-strahlung off *Z* bosons

**Fig. 81:**
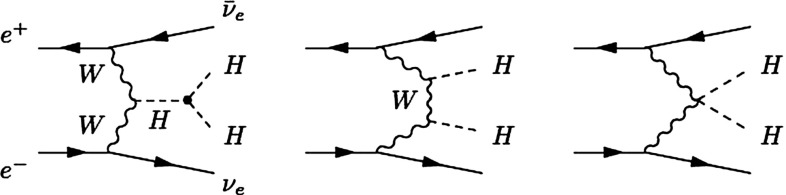
Generic Feynman diagrams contributing to Higgs pair production via *W* boson fusion

Vertices involving more than one Higgs boson could also provide a way to test the composite nature of the Higgs. Double Higgs production is a process that depends on the Higgs self-coupling and on the coupling between two Higgs bosons and two massive gauge bosons. At a low-energy $e^+e^-$ collider, double Higgs production proceeds mainly via double Higgs-strahlung off *Z* bosons, $e^+e^- \rightarrow ZHH$, and *WW* boson fusion to Higgs pairs, $e^+e^- \rightarrow HH \nu \bar{\nu }$ [79]. Generic diagrams are shown in Fig. 80 for double Higgs-strahlung and Fig. 81 for *WW* boson fusion.

**Fig. 82:**
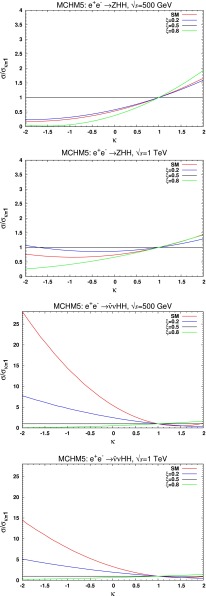
The *ZHH* (*upper two*) and *WW* fusion (*lower two*) cross sections in the SM (*red*) and the MCHM5 for $\xi =0.2$ (*blue*), $\xi =0.5$ (*black*) and $\xi =0.8$ (*green*) divided by the cross section of the corresponding model at $\kappa$=1, as a function of $\kappa$, for $\sqrt{s}=500$ GeV and $\sqrt{s}=1$ TeV

The double Higgs-strahlung process dominates at low energies, and in the MCHM4 and MCHM5 it is always smaller than in the SM, which is due to the suppressed Higgs-gauge couplings. On the other hand, the *WW* fusion process, which becomes important for higher c.m. energies, is enhanced compared to the SM for non-vanishing values of $\xi$ [442, 443]. This are due to interference effects related to the anomalous Higgs couplings. Furthermore, the amplitude grows like the c.m. energy squared contrary to the SM where it remains constant. The sensitivity of double Higgs-strahlung and gauge boson fusion processes to the tri-linear Higgs self-coupling of the corresponding model can be studied by varying the Higgs tri-linear coupling in terms of the respective self-interaction of the model in consideration, hence $\lambda _{HHH} (\kappa )= \kappa \, \lambda _{HHH}^{{\mathrm{MCHM4,5}}}$. This gives an estimate of how accurately the Higgs pair production process has to be measured in order to extract $\lambda _{HHH}$ within in the investigated model with a certain precision. Note, however, that this does not represent a test of models beyond the actually investigated theory. Figure 82 shows for the SM and for the MCHM5 with three representative $\xi$ values ($\xi =0.2,0.5,0.8$) the normalised double Higgs production cross sections for Higgs-strahlung and gauge boson fusion, respectively, at two c.m. energies, $\sqrt{s}=500$ GeV and 1 TeV, as a function of the modification factor $\kappa$. The cross sections are normalised with respect to the double Higgs production cross sections at $\kappa =1$ of the respective model. As can be inferred from the figure, both Higgs-strahlung and double Higgs production are more sensitive to $\lambda _{HHH}$ at lower c.m. energies. This is due to the suppression of the propagator in the diagrams which contain the tri-linear Higgs self-coupling with higher energies. In addition in *WW* fusion the *t*- and *u*-channel diagrams, insensitive to this coupling, become more important with rising energy. A high-energy $e^+e^-$ collider can exploit the *WW* fusion process to study the deviations in the coupling between two Higgs bosons and two gauge bosons by looking at the large $m_{HH}$ invariant mass distribution [441]. The sensivity obtained on $\xi$ via this process is almost an order of magnitude better than the one obtained from the study of double Higgs-strahlung [441].

The parton level analysis in Refs. [442, 443] showed that both double Higgs-strahlung and *WW* fusion have, in the 4*b* final state from the decay of the two 125 GeV Higgs bosons, sensitivity to a non-vanishing $\lambda _{HHH}$ at the 5$\sigma$ level in almost the whole $\xi$ range, with the exception of $\xi =0.5$ in MCHM5, where the tri-linear Higgs coupling vanishes, cf. Table 23.

#### Top sector

The fermionic sector of composite Higgs models, in particular the top sector, also shows an interesting phenomenology. With the fermion coupling strengths being proportional to their masses the top quark has the strongest coupling to the new sector and is most sensitive to new physics. It is hence natural to consider one of the two top helicities to be partially composite. The top-quark mass then arises through linear couplings to the strong sector. ATLAS and CMS already constrained the top partners to be heavier than 600–700 GeV at 95 % confidence level [444]. The associated new heavy top quark resonances have been shown to influence double Higgs production through gluon fusion [445, 446]. At $e^+e^-$ colliders these new resonances can be searched for either in single or in pair production [447].

#### Summary

**Fig. 83:**
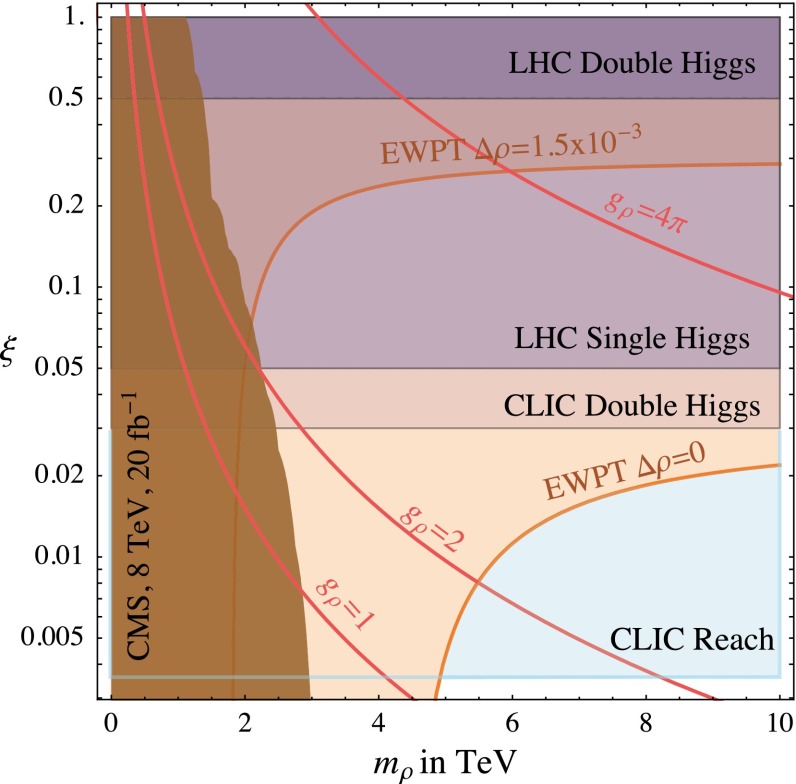
Summary plot of the current constraints and prospects for direct and indirect probes of Higgs compositeness. The *dark brown region* shows the current LHC limit from direct search for vector resonance. The *dark* (*medium light*) *horizontal purple bands* indicate the sensitivity on $\xi$ expected at the LHC from double (single) Higgs production with 300 fb$^{-1}$ of integrated luminosity. The *pink horizontal band* reports the sensitivity reach on $\xi$ from the study of double Higgs processes alone at CLIC with $1 ab^{-1}$ of integrated luminosity at 3 TeV, while the *light-blue horizontal band* shows the sensitivity reach on $\xi$ when considering single Higgs processes. Finally, experimental electroweak precision tests (EWPT) favour the region below the *orange thick line* with and without additional contributions to ${\varDelta } \rho$. From Ref. [441]

Composite Higgs models offer a nice possibility to solve the hierarchy problem by introducing a Higgs boson which emerges as pseudo Nambu–Goldstone boson from a strongly interacting sector. The phenomenology of these models is characterised by a light Higgs resonance which is separated through a mass gap from the other resonances of the strong sector, and which has modified couplings to the SM fermions and gauge bosons. At an $e^+e^-$ collider these couplings can be tested at high accuracy, and interactions with more than one Higgs boson, among which the Higgs self-interactions, will also be accessible. Genuine probes of the strong sector are provided by strong double Higgs production through gauge boson fusion and longitudinal gauge boson scattering, which both rise with the energy. A high-energy $e^+e^-$ collider like CLIC can also become sensitive to the tails of the spin-1 resonance contributions to the $WW\rightarrow WW$ and $WW\rightarrow HH$ amplitudes. Assuming partial compositeness in the top sector, new top resonances arise which can also be searched for at a future linear collider above the current LHC bound around 700 GeV. Figure 83 summarises the sensitivities at the LHC and CLIC for observing non-SM signatures from the composite nature of the Higgs boson in the plane of $\xi$ and $m_\rho$, the typical mass scale of the strong sector resonances.

### The Higgs portal^21^

A large fraction of matter in the universe is dark and not incorporated in the SM. Nevertheless, this new kind of invisible matter is expected to interact with the SM fields, naturally by gravitational interaction. However, another path could be opened by a Higgs portal which connects the SM Higgs field with potential Higgs fields in the dark sector, respecting all symmetry principles and well-founded theoretical SM concepts like renormalisability.

Even though the particles of the novel sector are invisible, the portal nevertheless induces observable signals in the SM, in the Higgs sector in particular. Mixings among Higgs bosons of the SM and of the dark sector modify Higgs couplings to the SM particles and give rise to invisible Higgs decays (beyond the cascades to neutrinos).

Crucial to an extraction of the $m_H\simeq 125~{\text {GeV}}$ Higgs boson candidate’s couplings to known matter is a good understanding of Higgs production *p* and decay mechanisms *d*, which can be constrained by measuring

66 $$\begin{aligned} \sigma _p \times \text {BR}_d \sim {{\varGamma }_p\,{\varGamma }_q\over {\varGamma }_{\text {tot}}}\sim {g_p^2\,g_d^2\bigg / \bigg ( \sum _{\text {modes}} g_i^2 \bigg )} \,, \end{aligned}$$

where $\sigma _p$, BR$_d$, and $g_i$ denote the involved production cross sections, branching ratios and couplings, as usual. Precisely reconstructing these underlying parameters is systematically hindered by the unavailable measurement of the total decay width ${\varGamma }_{\text {tot}}$. As a matter of fact, un-adapted search strategies at LHC miss certain non-SM decay modes, which naturally arise in models beyond the SM [448–452] and which would then manifest as an invisible branching ratio [453] in global fits. The expected constraint on such an invisible Higgs-boson decay at the LHC is ${\text {BR}}(H\rightarrow \text {invisible})\simeq 10~\%$ [454], a bound too loose to efficiently constrain physics beyond the SM, especially models where the Higgs field provides a portal to a hidden sector [80, 81, 455], which can provide a viable DM candidate [456, 457].

At a LC it is straightforward to derive the total width of the Higgs boson by combining the model-independent measurement of the partial width ${\varGamma }(ZZ^*)$ in semiinclusive Higgs-strahlung with the measurement of the branching ratio ${\text {BR}}(ZZ^*)$:

67 $$\begin{aligned} {\varGamma }_{\text {tot}}(H) = {\varGamma }(ZZ^*)/{\text {BR}}(ZZ^*) \,. \end{aligned}$$

Subsequently ${\text {BR}}(H\rightarrow \text {invisible})$ can be determined in a model-independent way [458].

From Eq. (66), we need to interpret the strong Higgs exclusion for heavy Higgs masses as a sign of a highly suppressed production cross section for heavier Higgs-like resonances. That heavy Higgs copies need to be weakly coupled in simple model-building realisations is already known from the investigation of electroweak precision measurements performed during the LEP era. This complements the requirement to include unitarising degrees of freedom for longitudinal gauge boson scattering $$V_LV_L\rightarrow V_L V_L$$$$(V=W^\pm , Z)$$, and, constraining to less extent, massive quark annihilation to longitudinal gauge bosons $$q\bar{q}\rightarrow V_LV_L$$. Saturating all three of these requirements fixes key characteristics of the phenomenological realisation of the Higgs mechanism, and does not allow dramatic modifications of the couplings $$\{g_i\}$$ in Eq. (66) away from the SM expectation of a light Higgs – the common predicament of electroweak-scale model building. In this sense gaining additional sensitivity to invisible Higgs decays (or the Higgs total width in general) beyond the limitations of the LHC hadronic environment is crucial to the understanding of electroweak physics at the desired level, before the picture will be clarified to the maximum extent possible at a LC.

The aforementioned Higgs-portal model [80, 81, 455] provides a theoretically well-defined, renormalisable, and yet minimal framework to explore both effects in a consistent way [460]: the influence of ${\varGamma }_{\text {inv}}$ on the Higgs phenomenology is captured, while heavier Higgs boson-like particles with suppressed couplings are naturally incorporated. Therefore, the Higgs-portal model not only provides a well-motivated SM Higgs sector extension in the context of DM searches^22^ and current data, but it represents an ideal model to generalise the SM in its phenomenologically unknown parameters to facilitate the SM’s validation by constraining the additional portal parameters beyond introducing biases (e.g. ${\varGamma }_H^{\text {tot}}\equiv {\varGamma }_H^{\text {SM}}$).

In its simplest form, leading to both a modified electroweak phenomenology and an invisible Higgs decay channel, the Higgs portal is given by the potential

68 $$\begin{aligned} \mathscr {V}= & {} \mu ^2_s |\phi _s|^2 + \lambda _s |\phi _s|^4 + \mu ^2_h |\phi _h|^2 + \lambda _h |\phi _h|^4\nonumber \\&+\, \eta _\chi |\phi _s|^2 |\phi _h|^2 \,, \end{aligned}$$

where $\phi _{s,h}$ are the SM and the hidden Higgs-doublet fields, respectively, *i.e.* the Higgs sector is mirrored [465]. The visible sector communicates to the hidden world via the additional operator $\eta _\chi |\phi _s|^2 |\phi _h|^2$, which exploits the fact that both $|\phi _s|^2$ and $|\phi _h|^2$ are singlet operators under both the SM and the invisible gauge groups.

After symmetry breaking which is triggered by the Higgs fields acquiring vacuum expectation values $|\phi _{s,h}|=v_{s,h}/\sqrt{2}$, the would-be-Nambu Goldstone bosons are eaten by the $W^\pm$, *Z* fields, and correspondingly in the hidden sector. The only effect (formulated here in unitary gauge) is a two-dimensional isometry which mixes the visible and the hidden Higgs bosons $H_{s,h}$:

69 $$\begin{aligned} H_1= & {} \cos \chi \, H_s + \sin \chi \, H_h\,, \nonumber \\ H_2= & {} -\sin \chi \, H_s + \cos \chi \, H_h \,, \end{aligned}$$

with the mixing angle

70 $$\begin{aligned} \tan \, 2\chi = \frac{\eta _\chi v_s v_h}{\lambda _s v^2_s - \lambda _h v^2_h} . \end{aligned}$$

The masses of the two Higgs fields are given by

71 $$\begin{aligned} M^2_{1,2}= & {} [\lambda _s v^2_s + \lambda _h v^2_h] \nonumber \\&\mp \, | \lambda _s v^2_s - \lambda _h v^2_h | \, \sqrt{1+\tan ^2 2\chi } . \end{aligned}$$

We assume $M_1\simeq 125~{\text {GeV}}$ in the following. The inverse phenomenological situation $M_1<M_2\simeq 125~{\text {GeV}}$, i.e. a Higgs field hiding below the upper LEP2 bound, is obviously reconciled by $\chi \rightarrow \pi -\chi$ since the potential has a ${\mathbb {Z}}_2$ symmetry. Consistency with electroweak precision data and an efficient unitarisation of the $V_LV_L$ scattering amplitudes relies in this case on $\cos ^2\chi$ being close to unity.

**Fig. 84:**
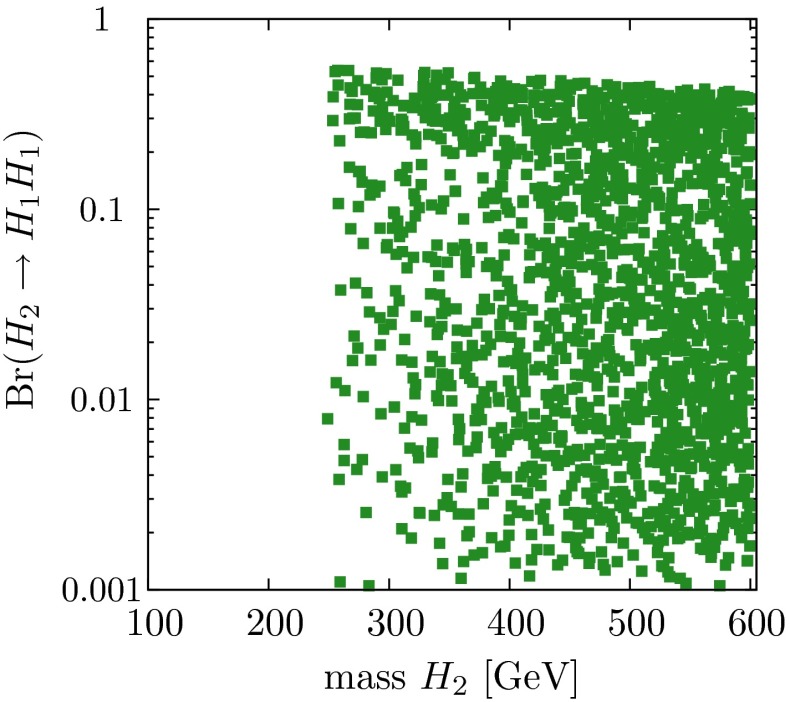
Scan over the Higgs-portal potential Eq. 68. We include the constraints from electroweak precision measurements

As a consequence of the mixing we have universally suppressed cross sections of the SM-Higgs

72a $$\begin{aligned}&\sigma _{1} = \cos ^2\chi \, \sigma ^\text {SM}_1 \nonumber \\&\sigma _{2} = \sin ^2\chi \, \sigma ^\text {SM}_2 , \end{aligned}$$

and

72b $$\begin{aligned}&{\varGamma }^\text {vis}_{1,2} = \cos ^2\chi \, \{\sin ^2\chi \} \, {\varGamma }^\text {SM}_{1,2} + {\varDelta }^\mathrm{vis}_2 \, {\varGamma }^{HH}_2 \nonumber \\&{\varGamma }^\text {inv}_{1,2} = \sin ^2\chi \, \{\cos ^2\chi \} \, {\varGamma }^\text {hid}_{1,2} + {\varDelta }^\mathrm{inv}_2 \, {\varGamma }^{HH}_2, \end{aligned}$$

where ${\varDelta }^{vis\{inv\}}_2 = \zeta ^2 \, \{[1-\zeta ]^2\} \ne 0$ and $\zeta = 1/[1+\tan ^2\chi$  ${\varGamma }_1^\text {hid}/ {\varGamma }^\text {SM}_{\text {tot},1}]$. We understand the index in ${\varDelta }_2$ such that this contribution only arises for the heavier state labelled with index=2.

We have also included cascade decays ${\varGamma }^{HH}_2$ (if they are kinematically allowed for $M_2\ge 2M_1$) and the possibility for a hidden partial decay width in Eq. (72b). The latter naturally arise if the hidden sector has matter content with $2m\le m_{H_1}$, *i.e.* in models with light DM candidates. Weak coupling of the heavier Higgs-like state is made explicit when correlating the Higgs-portal model with electroweak precision constraints [460].

Generically, the branching ratio of the heavier Higgs boson to two light Higgs states is small (Fig. 84) and kinematically suppressed, so that a direct measurement of the cascade decay at the LHC is challenging. Measurement strategies targeting invisible Higgs-boson decays at the LHC [466] are based on measurements in weak boson fusion [467] and associated production [468, 469]. Recent re-analysis of the monojet+Higgs production [452, 470], however, suggest that additional sensitivity can be gained in these channels, at least for the 7 and 8 TeV data samples.

The production of multiple final-state Higgs particles is another strong test of this model, since it predicts resonant contributions which can be large, see Fig. 84. A measurement of the involved tri-linear coupling $H_2H_1H_1$ is challenging at the LHC [471, 472] and can be achieved more straightforwardly at a high-luminosity LC [79]. Especially because we can separate the different final states of the light Higgs decay at the latter experiment, we can use the prediction of the various tri-linear couplings that arise from Eq. (68) to reconstruct the potential.

**Fig. 85:**
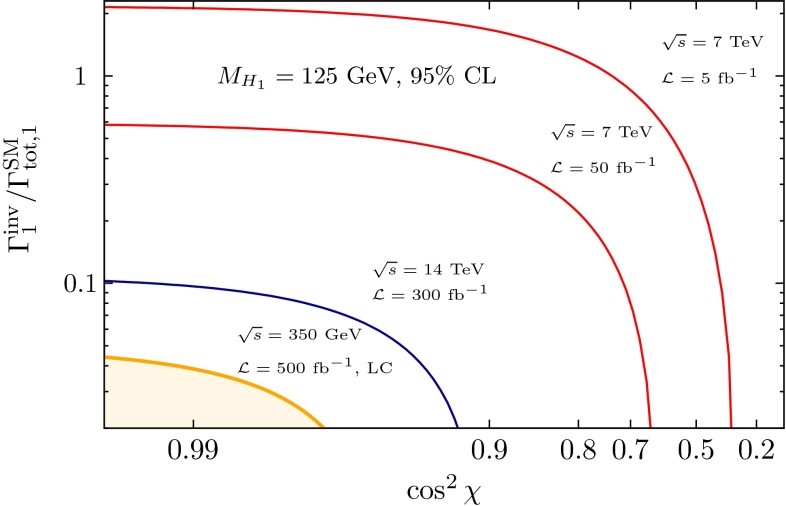
95% confidence level contours for a measurement of ${\varGamma }_1^{\text {hid}}/{\varGamma }_1^{\text {SM}}$ at the LHC and a $350~{\text {GeV}}$ LC. We use Sfitter [459] for the LHC results and we adopt the linear collider uncertainties of reference [458]

The precision to which invisible decays can be studied at the LHC is ultimately limited by the machine’s systematics which will saturate at luminosities ${\mathscr {L}}\simeq 300~{\text {fb}}^{-1}$, see Fig. 85. Bounds on visible decays are typically expressed as ratios to the SM expectation, which, for the lighter $M_1\simeq 125~{\text {GeV}}$ state, can be rephrased in the portal model for either $i = pp$ or $e^+e^-$ initial stares

73 $$\begin{aligned} \dfrac{\sigma [i \rightarrow H_1 \rightarrow F]}{\sigma [i \rightarrow H_1 \rightarrow F]^\text {SM}} = \dfrac{\cos ^2\chi }{1 + \tan ^2\chi \, [{{\varGamma }^\text {hid}_1}/{{\varGamma }^\text {SM}_{\text {tot},1}}]} \le \mathscr {R}_1 , \end{aligned}$$

where ${\mathscr {R}}_1$ denotes the observed exclusion limit (signal strength). An identical quantity can be derived from future constraints on invisible decays

74 $$\begin{aligned} \dfrac{\sigma [i \rightarrow H_1 \rightarrow inv]}{\sigma [i \rightarrow H_1]^\text {SM}} = \dfrac{\sin ^2\chi \, [{\varGamma }^\text {hid}_1 / {\varGamma }^\text {SM}_{\text {tot},1}]}{1 + \tan ^2\chi \, [{{\varGamma }^\text {hid}_1}/{{\varGamma }^\text {SM}_{\text {tot},1}}]} \le \mathscr {J}_1 . \end{aligned}$$

Similar relations hold for $H_2$, and there are portal-specific sum rules which facilitate the reconstruction of the mixing angle from measurements of ${\mathscr {J}}_{1,2}$ and ${\mathscr {R}}_{1,2}$,

75 $$\begin{aligned} {\mathscr {R}}_1+{\mathscr {J}}_1=&\cos ^2\chi \,,\nonumber \\ {\mathscr {R}}_2+{\mathscr {J}}_2=&\sin ^2\chi \,. \end{aligned}$$

While the LHC running at 14 TeV will eventually probe small visible production cross sections ${\mathscr {R}}_{2}$ (Eq. (74) becomes an equality), the invisible decay searches at the LHC will most likely yield a 95 % confidence level bound [473] on ${\mathscr {J}}_{1,2}$ [466] rather than a statistically significant observation. The bounds can be vastly improved by performing by performing precision spectroscopy of the 125-GeV Higgs candidate in the associated production channel $e^+e^-\rightarrow HZ$ at, e.g., a 350 GeV LC (see also Ref. [474]). Still, invisible Higgs searches that solely provide upper limits on both ${\mathscr {J}}_{1,2}$ are not enough to fully reconstruct the portal model if a second Higgs-like state is discovered as a result of Eq. (75). Only the precise *measurement*, which is impossible at the LHC, solves this predicament, but an LC is the perfect instrument to pursue such an analysis in the associated production channel.

**Fig. 86:**
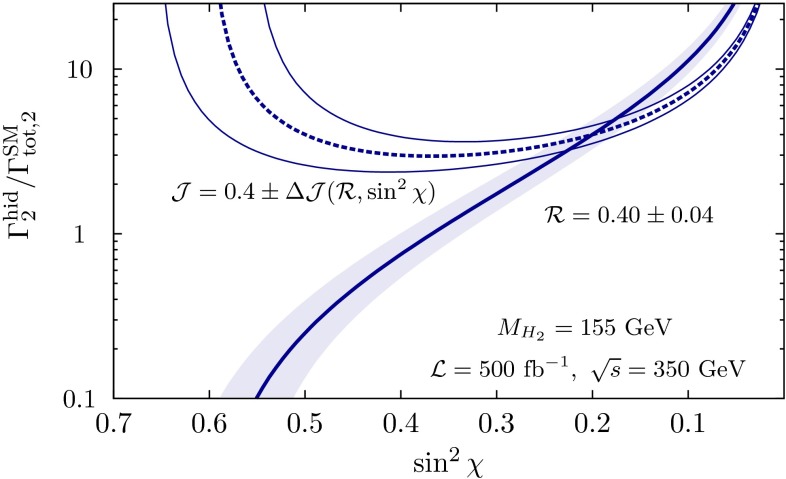
Measurement of a hypothetical portal model at a 350 GeV linear collider, uncertainties are adopted from Ref. [458]. A measurement of ${\mathscr {R}}_2$ at the LHC, with only an *upper* 95 % confidence level bound on ${\mathscr {J}}_2$ does not constrain the region ${\varGamma }_2^{\text {hid}}/{\varGamma }_{{\text {tot}},2}^{\text {SM}}$ below the ${\mathscr {J}}_2$ curve. This degeneracy is lifted with a measurement at a linear collider

In Fig. 86 we show a hypothetical situation, where $H_2$ is discovered at the LHC with ${\mathscr {R}}_2=0.4$; the error is given by a more precise measurement at a 350 GeV LC, see Fig. 84. The measurement of ${\mathscr {J}}_2=0.4$ allows one to reconstruct $\sin ^2\chi$, which can be seeded to a reconstruction algorithm [460] that yields the full Higgs-portal potential Eq. (68).

From Eq. (75) we also obtain the sum rule

76 $$\begin{aligned} {\mathscr {R}}_1+{\mathscr {J}}_1+ {\mathscr {R}}_2+{\mathscr {J}}_2=1. \end{aligned}$$

which provides a strong additional test of the portal model Eq. (75) when a measurement of the invisible branching ratios via ${\mathscr {J}}_{1,2}$ becomes available at a future linear collider.

To summarise, the Higgs portal can open the path to the dark sector of matter and can allow crucial observations on this novel kind of matter in a global way. While first hints may be expected from LHC experiments, high-precision analyses of Higgs properties and the observation of invisible decays at LC can give rise to a first transparent picture of a new world of matter.

### The NMSSM^23^

In the Next-to-Minimal Supersymmetric Standard Model (NMSSM) the Higgs sector of the MSSM is extended by an additional gauge singlet superfield $\widehat{S}$ [89]. It is the simplest supersymmetric extension of the standard model with a scale invariant superpotential; the $\mu$-term $\mu \widehat{H_u} \widehat{H_d}$ in the superpotential $W_{\mathrm {MSSM}}$ of the MSSM is replaced by

77 $$\begin{aligned} W_{\mathrm {NMSSM}} = \lambda \widehat{S} \widehat{H_u} \widehat{H_d} + \frac{\kappa }{3} \widehat{S}^3\; . \end{aligned}$$

Once the scalar component *S* of the superfield $\widehat{S}$ assumes a vacuum expectation value *s*, the first term in the superpotential (77) generates an effective $\mu$-term with

78 $$\begin{aligned} \mu _{\mathrm {eff}} = \lambda s. \end{aligned}$$

In addition to the NMSSM-specific Yukawa couplings $\lambda$ and $\kappa$, the parameter space of the NMSSM contains soft supersymmetry breaking tri-linear couplings $A_\lambda$, $A_\kappa$ and soft supersymmetry breaking mass terms $m_{H_u}^2$, $m_{H_d}^2$ and $m_S^2$. The order of *s* and hence of $\mu _{\mathrm {eff}}$ is essentially determined by $A_\kappa$ and $m_S^2$, hence $\mu _{\mathrm {eff}}$ is automatically of the order of the soft supersymmetry breaking terms.

The physical states in the Higgs sector of the NMSSM (assuming ${\textit{CP}}$-conservation) consist in three neutral ${\textit{CP}}$-even states $H_i$ (ordered in mass), two neutral ${\textit{CP}}$-odd states $A_i$ and charged Higgs bosons $H^\pm$. The ${\textit{CP}}$-even states $H_i$ are mixtures of the real components of the weak eigenstates $H_u$, $H_d$ and *S*:

79 $$\begin{aligned} H_i = S_{1,d}\ H_d + S_{1,u}\ H_u +S_{1,s}\ S , \end{aligned}$$

where the mixing angles $S_{i,j}$ depend on the a priori unknown parameters in the Higgs potential. Similarly, the two ${\textit{CP}}$-odd states $A_i$ are mixtures of the imaginary components of the weak eigenstates $H_u$, $H_d$ and *S* without the Goldstone boson. In addition, the fermionic component of the superfield $\widehat{S}$ leads to a fifth neutralino, which mixes with the four neutralinos of the MSSM.

In view of the mass of 125–126 GeV of the at least approximately Standard Model-like Higgs boson $H_{\mathrm {SM}}$ measured at the LHC, the NMSSM has received considerable attention: In contrast to the MSSM, no large radiative corrections to the Higgs mass (implying fine tuning in parameter space) are required in order to obtain $M_{H_{\mathrm {SM}}}$ well above $M_Z$, the upper bound on $M_{H_{\mathrm {SM}}}$ at tree level in that model. In the NMSSM, additional tree-level contributions to $M_{H_{\mathrm {SM}}}$ originate from the superpotential Eq. (77) [89]. Also a mixing with a lighter mostly singlet-like Higgs boson can increase the mass of the mostly Standard-Model-like Higgs boson [475], in which case one has to identify $H_{\mathrm {SM}}$ with $H_2$. Both effects allow one to obtain $M_{H_{\mathrm {SM}}}\sim$ 125–126 GeV without fine tuning and, moreover, such a mixing could easily explain an enhanced branching fraction of this Higgs boson (from now on denoted as $H_{125}$) into $\gamma \gamma$ [260, 476–485].

Depending on the mixing angles, on the masses of the additional Higgs bosons and on their branching fractions, the LHC can be blind to the extended Higgs sector of the NMSSM beyond the mostly standard model-like state. Then the detection of the additional states will be possible only at a LC. Also if hints for such an extended Higgs sector are observed at the LHC, only a LC will be able to study its properties in more detail. Earlier studies of the detection of NMSSM Higgs bosons at $e^+ e^-$ colliders can be found in [486–491].

The dominant production modes of ${\textit{CP}}$-even Higgs bosons at a LC (associate *ZH* production and VBF) depend on the Higgs couplings to the electroweak gauge bosons. Denoting the coupling of $H_{\mathrm {SM}}$ to electroweak gauge bosons by $g_\mathrm {SM}$, the couplings $g_i$ of the ${\textit{CP}}$-even states $H_i$ satisfy the sum rule

80 $$\begin{aligned} \sum _i g_i^2 = g_\mathrm {SM}^2\; . \end{aligned}$$

If a measurement of the coupling $g_i$ of the 125 GeV Higgs boson at the LC gives a value significantly below $g_\mathrm {SM}$, one can deduce the presence of additional Higgs states. The scenario where $H_{125}=H_2$ is particularly natural in the parameter space of the NMSSM. Then the coupling $g_1$ of the lightest Higgs boson $H_1$ must satisfy constraints from LEP II, if its mass is below $\sim$114 GeV.

**Fig. 87:**
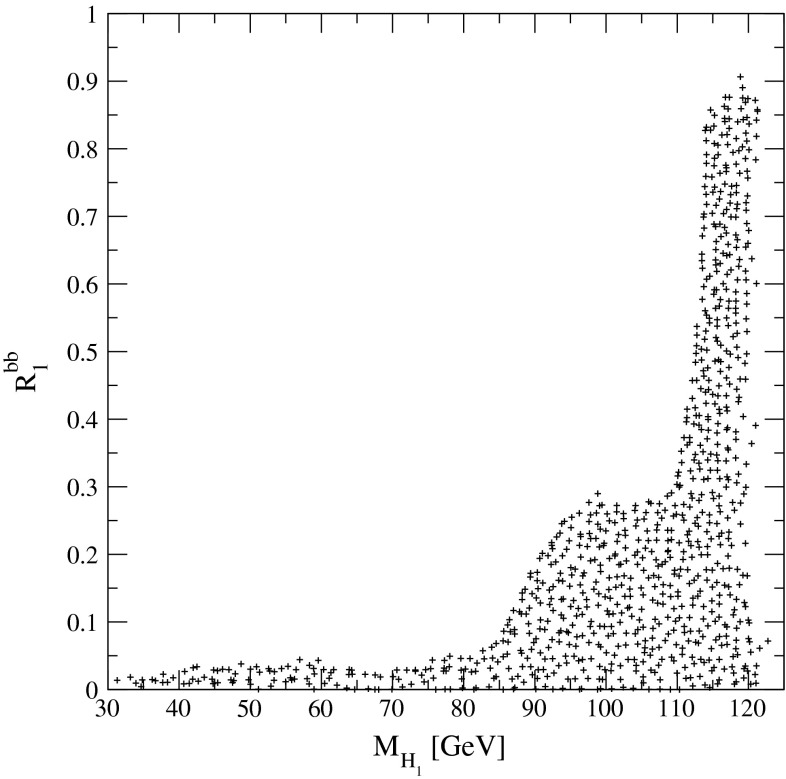
The reduced signal cross section $R_1^{bb}$ at a $e^+ e^-$ collider as defined in the text, as a function of $M_{H_1}$ in the semiconstrained NMSSM (from [482])

**Fig. 88:**
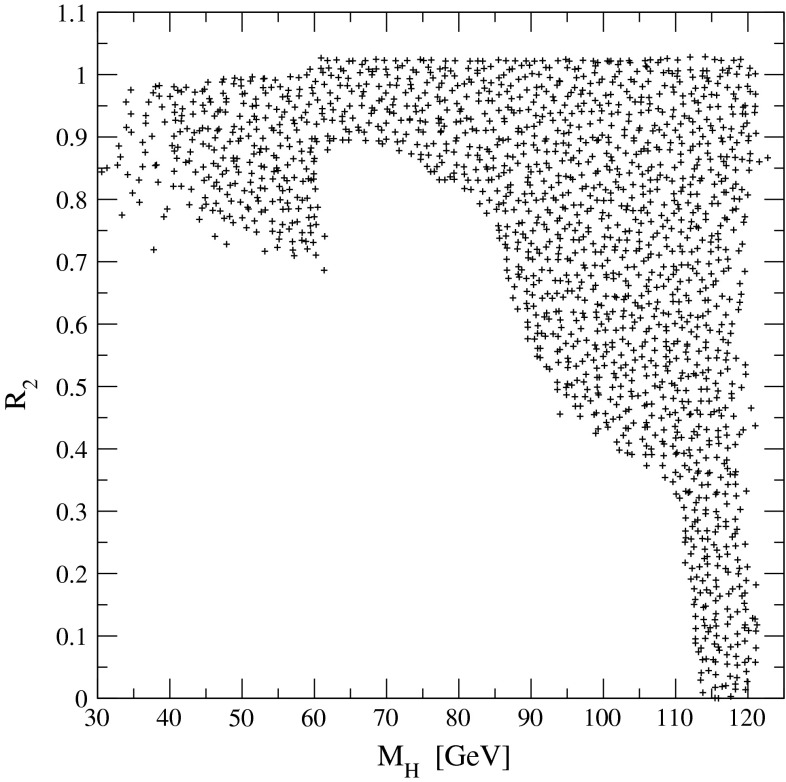
The reduced signal cross section $R_2^{bb}$ as function of $M_{H_1}$ in the semiconstrained NMSSM (from [482]).

The allowed gauge couplings$$^2$$$$\times $$ branching fractions into *bb* of $$H_1$$ and $$H_2$$ have been studied as a function of $$M_{H_1}$$, once $$M_{H_2}\sim 125$$ GeV is imposed, in the parameter space of the semiconstrained NMSSM in [482]. (In the semiconstrained NMSSM, squark and slepton masses at the GUT scale are given by a common value $$m_0$$, gaugino masses by a common value $$M_{1/2}$$, but the NMSSM-specific soft Higgs masses and tri-linear couplings are left free.) The results for the allowed values of $$R_i^{bb} = \frac{g_i^2 \times \mathrm{BR}(H_i \rightarrow bb)}{g_\mathrm {SM}^2 \times \mathrm{BR}(H_\mathrm {SM} \rightarrow bb)}$$ are shown in Figs. 87 and 88. Since here $$\mathrm{BR}(H_i \rightarrow bb) \approx \mathrm{BR}(H_\mathrm {SM} \rightarrow bb)$$, one has $$R_i^{bb} \approx \frac{g_i^2}{g_\mathrm {SM}^2 }$$.

For $M_{H_1} < 114$ GeV, the upper bounds on $R_1^{bb}$ in Fig. 87 follow from the LEP II constraints in [321]. Still, even for $M_{H_1} < 110$ GeV, a detection of $H_1$ at a LC is possible (but difficult at the LHC within the semiconstrained NMSSM). From Fig. 88 one finds that, if $M_{H_1} > 114$ GeV, $R_2^{bb}$ can assume all possible values from 0 to 1. Note that $R_1^{bb}$ and $R_2^{bb}$ satisfy approximately $R_1^{bb}+R_2^{bb}\sim 1$.

For $M_{H_1} \sim 100$ GeV and $R_1^{bb} \sim 0.1$–0.25, $H_1$ can explain the $\sim 2 \sigma$ excess in the *bb* final state for this range of Higgs masses at LEP II [321]. Properties of such points in the parameter space of the semiconstrained NMSSM have been studied in [492], amongst others the production cross sections of the various Higgs bosons in various channels at a LC.

**Fig. 89:**
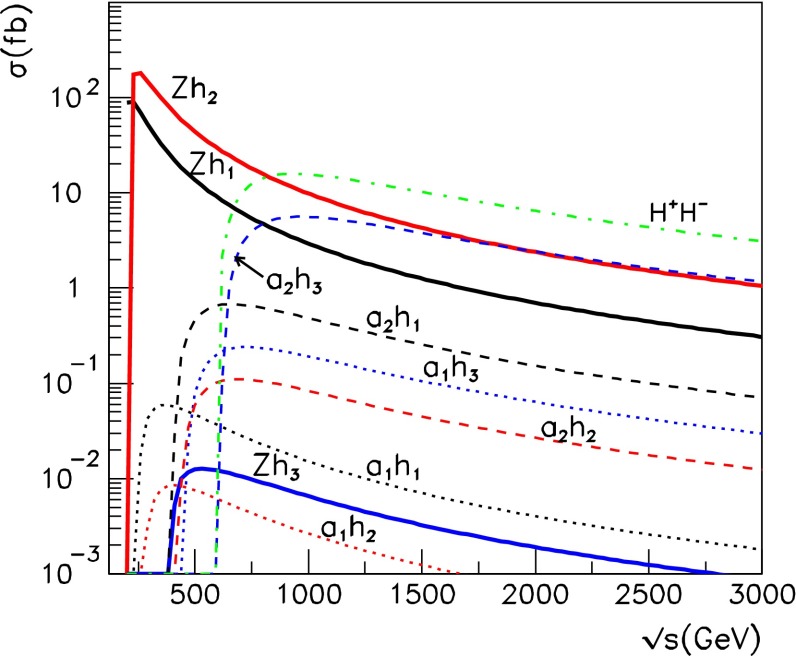
Higgs production cross sections at a $e^+ e^-$ collider in the channels $Z H_1$, $Z H_2$, $H^+ H^-$ and $H_i A_j$ for a point in the parameter space of the semiconstrained NMSSM with Higgs masses as indicated in the text, from [492]

For a typical point with $M_{H_1} \sim 99$ GeV, $M_{H_2} \sim 124$ GeV (and an enhanced signal rate in the $\gamma \gamma$ final state at the LHC), $M_{H_3} \sim 311$ GeV, $M_{A_1} \sim 140$ GeV, $M_{A_2} \sim 302$ GeV and $M_{H^\pm } \sim 295$ GeV, the production cross sections in the channels $Z H_1$, $Z H_2$, $H^+ H^-$ and $H_i A_j$ are shown in Fig. 89 as function of $\sqrt{s}$ of a LC (from [492]). Note that, for suitable mixing angles of $H_i$ and $A_j$, also $H_i A_j$ production via $e^+ + e^- \rightarrow H_i A_j$ is possible as in the MSSM.

However, an additional ${\textit{CP}}$-even Higgs boson with sizeable coupling $g_i$ can also be heavier than 125 GeV; such a scenario is motivated by best fits to present LHC and Tevatron data [493].

Other NMSSM-specific scenarios are possible Higgs-to-Higgs decays (see, e.g., [494]). For the 125 GeV Higgs boson, the measured standard model-like decay modes at the LHC indicate that Higgs-to-Higgs decays are not dominant for this state, but branching fractions of ${\mathscr {O}}(10~\%)$ are allowed. In the NMSSM, $H_{125}$ could decay into pairs of lighter ${\textit{CP}}$-even or ${\textit{CP}}$-odd states (if kinematically possible). If these states are heavier than $\sim 10$ GeV and decay dominantly into *bb*, such decay modes of $H_{125}$ into 4*b* (or $2b2\tau$) would be practically invisible at the LHC. At a LC, using the leptonic decays of *Z* in the *ZH* Higgs production mode and/or VBF, such unconventional decays can be discovered [490].

In addition, more Higgs-to-Higgs decays involving all three ${\textit{CP}}$-even states *H* and both ${\textit{CP}}$-odd states *A* (omitting indices for simplicity) like $H\rightarrow HH$, $H\rightarrow AA$, $H\rightarrow ZA$, $A\rightarrow AH$, $A\rightarrow ZH$, $H^\pm \rightarrow W^\pm H$ and $H^\pm \rightarrow W^\pm A$ are possible whenever kinematically allowed, and visible whenever the “starting point” of the cascade has a sufficiently large production cross section (see, e.g., Fig. 89) and the involved couplings are not too small. Even if a mostly standard model-like Higgs boson at 125 GeV is imposed, the remaining unknown parameters in the Higgs sector of the NMSSM allow for all of these scenarios.

The relevance of a $\gamma \gamma$ collider for the study of Higgs-to-Higgs decays in the NMSSM has been underlined in [495]. Astonishingly, also pure singlet-like states *H* and *A* can be produced in the $\gamma \gamma$ mode of a LC. In the standard model, a $H\gamma \gamma$-vertex is loop-induced with mainly $W^\pm$ bosons and top-quarks circulating in the loops. In the case of the NMSSM and dominantly singlet-like states $H_S$ and $A_S$ (without couplings to $W^\pm$ bosons or top quarks), higgsino-like charginos can circulate in the loops. The corresponding couplings of $H_S$ and $A_S$ to higgsino-like charginos originate from the term $\lambda \widehat{S} \widehat{H_u} \widehat{H_d}$ in the superpotential (77) and are absent for the MSSM-like ${\textit{CP}}$-even and ${\textit{CP}}$-odd Higgs states.

**Fig. 90:**
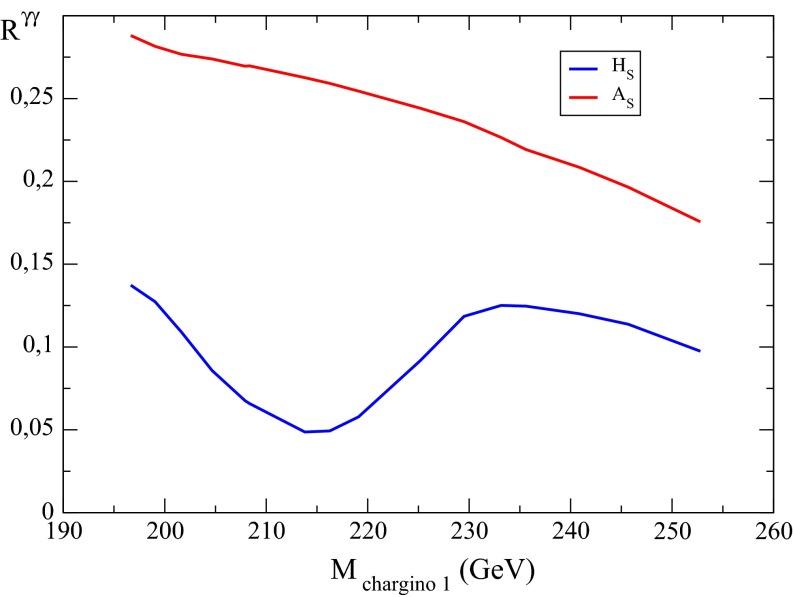
The reduced coupling $R^{\gamma \gamma }$, as defined in Eq. (81), as function of $M_{\mathrm {chargino}_1}$ for $M_{A_S} \sim M_{H_S} \sim 260$ GeV, for a scenario explaining a 130 GeV photon line from dark matter annihilation in the galactic centre

Possible values of the reduced couplings $R^{\gamma \gamma }$ of such nearly pure singlet-like states $H_S$ and $A_S$ are shown in Fig. 90, where we define

81 $$\begin{aligned} R^{\gamma \gamma } = \frac{{\varGamma }(H/A \rightarrow \gamma \gamma )}{{\varGamma }(H_\mathrm {SM} \rightarrow \gamma \gamma )} \end{aligned}$$

for a standard model-like $H_\mathrm {SM}$ of the same mass as $H_S$ or $A_S$. The production cross sections of these states in the $\gamma \gamma$ mode of a LC are given by the production cross section of $H_\mathrm {SM}$ multiplied by same ratio $R^{\gamma \gamma }$.

The values of $R^{\gamma \gamma }$ shown in Fig. 90 correspond to a region in the parameter space of the NMSSM where the Standard Model-like $H_\mathrm {SM}$ has a mass of $\sim$125 GeV and, simultaneously, DM annihilation in the galactic centre can give rise to a 130 GeV photon line [496]. Hence the LSP mass is 130 GeV, $M_{A_S} (\equiv M_{A_1}) \sim 260$ GeV in order to produce two photons from LSP annihilation with $A_S$ exchange in the *s*-channel, and $M_{H_S} (\equiv M_{H_2}) \approx 260$ GeV such that $H_S$ exchange in the *s*-channel gives a relic density compatible with WMAP. $\lambda$ varies between 0.6 andd 0.65, the wino mass parameter is fixed to $M_2=300$ GeV, but $\mu _\mathrm {eff}$ varies from 250–350 GeV. The nature of the chargino$_1$ varies slightly with $\mu _\mathrm {eff}$, but is always $\approx 50\%$ wino and higgsino-like. The values shown in Fig. 90 have been obtained using the code NMSSMTools [497, 498]. We see in Fig. 90 that notably $R^{\gamma \gamma }(A_S)$ can assume values close to 0.3, leading to a significant production cross section in the $\gamma \gamma$ mode of a LC.

**Fig. 91:**
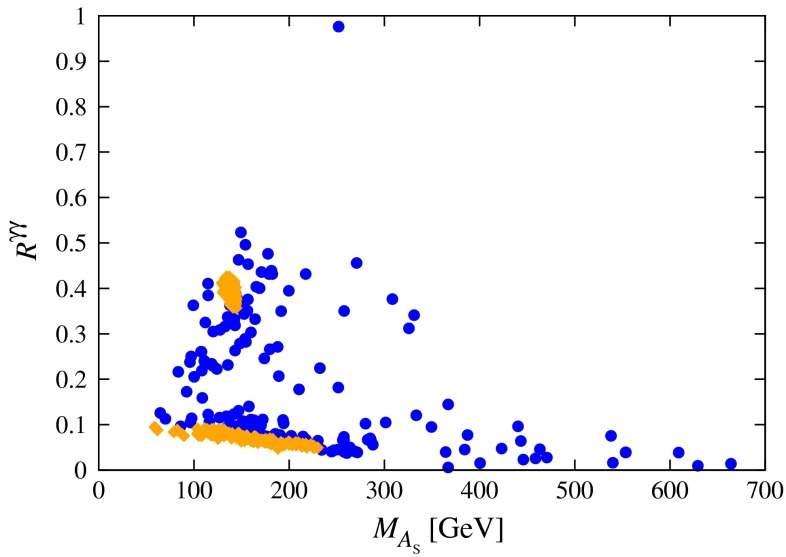
The reduced coupling $R^{\gamma \gamma }$ as a function of $M_{A_S}$, for points in the semiconstrained NMSSM where $H_S$ with $M_{H_S}\sim 100$ GeV explains the excess in *bb* at LEP II (from [492]; orange diamonds satisfy the WMAP constraint on the dark matter relic density)

**Fig. 92:**
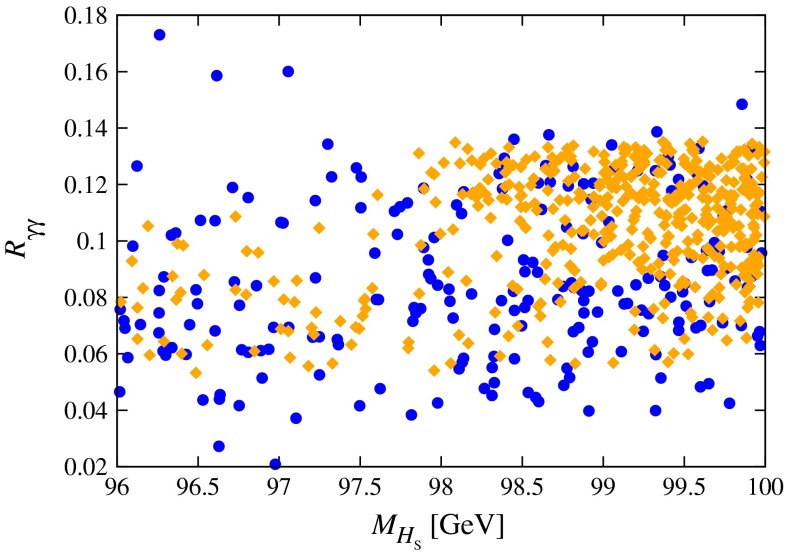
The reduced coupling $R^{\gamma \gamma }$ as a function of $M_{H_S}$, for points in the semiconstrained NMSSM where $H_S$ with $M_{H_S}\sim 100$ GeV explains the excess in *bb* at LEP II (from [492]; orange diamonds satisfy the WMAP constraint on the dark matter relic density)

Returning to the semiconstrained NMSSM with$M_{H_1} \equiv M_{H_S} \sim 100$ GeV and $M_{H_2} \sim 125$ GeV, scatter plots for $R^{\gamma \gamma }(A_S)$ and $R^{\gamma \gamma }(H_S)$ as a function of $M_{A_S}$ and $M_{H_S}$ are shown in Figs. 91 and 92 (from [492]). Again we see that the prospects for $A_S$/$H_S$ discovery are quite promising for sufficiently large luminosity, since the production cross sections are typically about 10 % (possibly larger) than those of a SM-like Higgs boson of a corresponding mass.

Finally the NMSSM differs from the MSSM also due to the presence of a fifth neutralino, the fermionic component of the superfield $\widehat{S}$. Phenomenological analyses of pair production of neutralinos in the NMSSM at $e^+\,e^-$ colliders at higher energies have been performed in [43, 44, 499–503]. Since the information on the neutralino sector from the LHC will be quite limited, a $e^+\,e^-$ collider can be crucial to distinguish the NMSSM neutralino sector from the one of the MSSM [502], although it cannot be guaranteed that the difference is visible if one is close to the decoupling limit $\lambda ,\ \kappa \rightarrow 0$. This question has also been addressed in the radiative production of the lightest neutralino pair, $e^+\,e^- \rightarrow \tilde{\chi }^0_1\,\tilde{\chi }^0_1\,\gamma$, at a LC with $\sqrt{s} = 500$ GeV in [503].

To summarise, the NMSSM is a well-motivated supersymmetric extension of the standard model, notably in view of the discovery of a Higgs boson at 125 GeV and a potentially enhanced branching fraction into $\gamma \gamma$. Due to their reduced couplings to electroweak gauge bosons it is not clear, however, whether the LHC will be able to verify the extended Higgs and neutralino sectors of the NMSSM. Only a LC will be able to perform measurements of such reduced couplings, correspondingly reduced production cross sections, and possible unconventional decay modes. These incompass both possible Higgs-to-Higgs cascade decays, as well as cascades in the neutralino sector.

### Little Higgs^24^

The Little Higgs (LH) model [504–506] is well known to be one of the attractive scenarios for physics beyond the standard model (SM). In this subsection, we review the physics of the model at future linear collider experiments by referring to several studies reported so far.

#### About the LH model

The cutoff scale of the standard model (SM) is constrained by electroweak precision measurements: If we assume the existence of a $\sim$125 GeV SM Higgs-like resonance, the cutoff scale should be higher than roughly 5 TeV [507, 508]. However, such a relatively high cutoff scale requires a fine tuning in the Higgs potential because the Higgs potential receives the quadratic divergent radiative correction.

In LH models, the Higgs boson is regarded as a pseudo Nambu–Goldsone (NG) boson which arises from a global symmetry breaking at high energy, $\sim$10 TeV. Although Yukawa and gauge couplings break the global symmetry explicitly, some global symmetry is not broken by one of these couplings: in LH models, the breaking of such a symmetry is achieved only by two or more couplings, which is called “collective” symmetry breaking. Because of the collective symmetry breaking, the quadratic divergence from SM loop diagrams is cancelled by new-particle diagrams at the one-loop level.

As a bottom-up approach, specifying a coset group, we investigate the phenomenology of such a scenario by a non-linear sigma model. In particular, the littlest Higgs (LLH) model [506] described by an *SU*(5) / *SO*(5) symmetry breaking and the simplest little Higgs (SLH) model [509] described by an $[SU(3)\times U(1)]^2/ [SU(2)\times U(1)]^2$ symmetry breaking have been studied about its expected phenomenology well so far. Here we review the ILC physics mainly focusing on the LLH model.

The LLH model is based on a non-linear sigma model describing an *SU*(5) / *SO*(5) symmetry breaking with the vacuum expectation value $f \sim \mathscr {O}$(1) TeV. An [$SU(2) \times U(1)]^2$ subgroup of the *SU*(5) is gauged and broken down to the SM $SU(2)_L\times U(1)_Y$. Fourteen NG bosons arise and it can be decomposed into $\mathbf{1}_0 \oplus \mathbf{3}_0 \oplus \mathbf{2}_{\pm 1/2} \oplus \mathbf{3}_{\pm 1}$ under the electroweak gauge group. The $\mathbf{1}_0 \oplus \mathbf{3}_0$ are eaten by heavy gauge bosons $A_H, Z_H, W_H^\pm$, and $\mathbf{2}_{\pm 1/2} \oplus \mathbf{3}_{\pm 1}$ are the SM Higgs field *h* and new triplet Higgs field $\Phi$, respectively. To realise the collective symmetry breaking, *SU*(2) singlet vector-like top quark partners, $T_L$ and $T_R$, are also introduced. These heavy particles have masses which are proportional to *f* and depend also on the gauge coupling, charges and Yukawa couplings. The Higgs potential is generated radiatively and it depends also on parameters of UV theory at the cutoff scale ${\Lambda } \sim 4 \pi f$.

Even in the model, the new-particle contributions are strongly constrained at precision measurements.

Pushing new-particle masses up to avoid the constraint, the fine tuning in the Higgs potential is reintroduced. To avoid the reintroducing the fine tuning, implementing of the $Z_2$ symmetry called *T*-parity has been proposed [510–512].^25^

In the LLH model, the *T*-parity is defined as the invariance under the exchanging gauged $[SU(2) \times U(1)]_1$ and $[SU(2) \times U(1)]_2$. Then, for all generations of the lepton and squark sector, new heavy fermions are introduced to implement this symmetry. Under the parity, the new particles are assigned to be a minus charge (T odd), while the SM particles have a plus charge (T even). Thus, heavy particles are not mixing with SM particles. Then the tree-level new particle contribution to electroweak precision measurements are forbidden and the new-particle masses can be light.

It has been suggested that the *T*-parity is broken by anomalies in the typical strongly coupled UV theory [515, 516] and the possibilities of the conserved *T*-parity scenario and another parity are also studied [517–521]. If the *T*-parity is an exact symmetry, the lightest T-odd particle, heavy photon in the LLH model, is stable and provides a DM candidate. Even if the *T*-parity is broken by anomalies, contribution to electroweak precision measurements are still suppressed, while the lightest T-odd particle would decay at colliders [522, 523].

As described above, top quark partner, new gauge bosons and additional scalar bosons are expected in LH models, while its details strongly depend on models. In the model with *T*-parity, T-odd quark partners and lepton partners are introduced additionally. The Higgs boson phenomenology would be different from the SM prediction due to the new-particle contributions as well as deviations from the SM coupling which would appear from higher-dimensional operators.

#### Higgs phenomenology in LH

In LH models, parameters of the Higgs potential cannot be estimated without calculating the contribution of a specifying UV theory. As a phenomenological approach, we consider these parameters as free parameters and these are determined by observables, e.g., Higgs mass. As described here, there are possibilities to change the Higgs boson phenomenology from the SM prediction and it may be checked at the ILC.

*Higgs decay from loop diagram* One of the possibility to change the Higgs phenomenology is contributions from top partner as well as the deviation from the SM couplings. It leads to deviations in the decay branching ratios of the $h \rightarrow gg$ (also indicating deviations in the main Higgs production channel at the LHC) and $h \rightarrow \gamma \gamma$ modes, via the top partner-loop diagrams. The extra gauge bosons and charged scalar bosons also contribute to the $h \rightarrow \gamma \gamma$ decay.

**Fig. 93:**
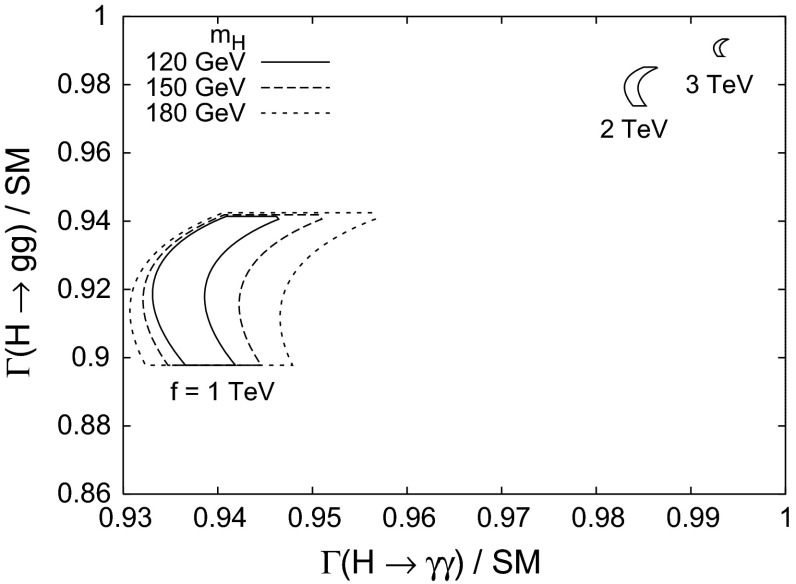
Accessible range of ${\varGamma }(h \rightarrow gg)$ and ${\varGamma }(h \rightarrow \gamma \gamma )$ normalised to the SM value in the LLH model (from [524])

Figure 93 shows the range of partial decay widths, ${\varGamma }(h \rightarrow gg)$ and ${\varGamma }(h \rightarrow \gamma \gamma )$, in the LLH model varying model parameters [524]. In the model, the deviation of the top Yukawa coupling suppresses the ${\varGamma }(h \rightarrow gg)$, while contributions from top partner and mixing in the top sector enhance the partial decay width. Totally, these additional top sector contributions suppresses the ${\varGamma }(h \rightarrow gg)$ in Fig. 93. On the other hand, it enhances the ${\varGamma }(h \rightarrow \gamma \gamma )$ because the *W* boson loop contribution is dominant in the SM and the fermion-loop contributions have a minus sign. The contribution from the heavy gauge bosons suppresses the ${\varGamma }(h \rightarrow \gamma \gamma )$ as well as the deviation of the gauge boson coupling and mixing in the gauge boson sector due to the sign of the $W_H W_H h$ coupling. The charged Higgs contribution leads to an enhancement. The doubly charged Higgs contribution is small because the coupling to the Higgs boson is suppressed; thus, it is neglected here [524]. In a similar way the $\gamma Z$ decay would be affected [525].

**Fig. 94:**
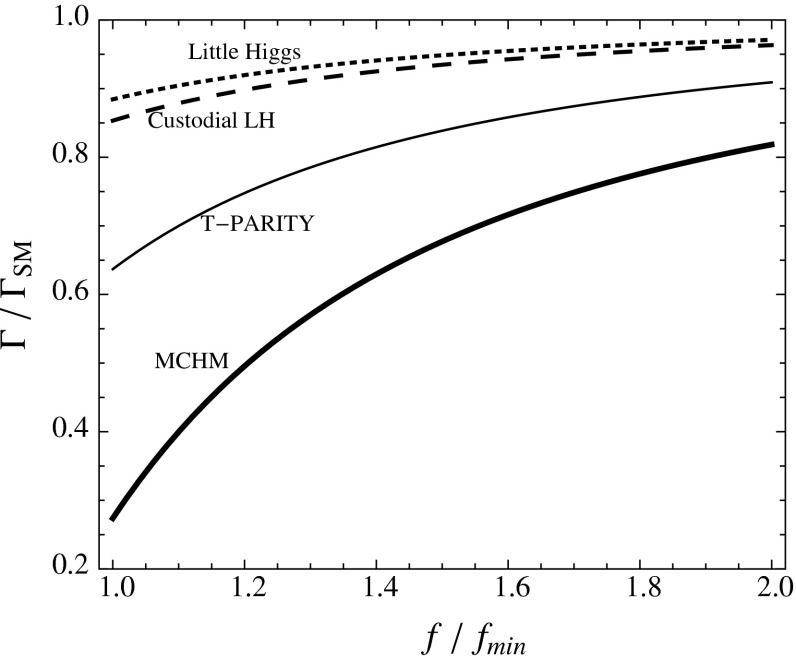
The ${\varGamma }(h \rightarrow gg)$ normalised to the SM value (from [528]). The $f_\mathrm{min}$ is defined as the smallest value allowed by electroweak precision measurements and the values are 1.2 TeV for the LLH model, 500 GeV for *T*-parity case, 700 GeV for custodial littlest Higgs model and 500 GeV for minimal composite Higgs model, respectively (for details, see [528])

In the model with *T*-parity, there is also the contribution from T-odd heavy fermions and the contribution is negative to ${\varGamma }(h \rightarrow gg)$ and positive to ${\varGamma }(h \rightarrow \gamma \gamma )$ [526]. Furthermore, in the model with *T*-parity case (and also in a decoupling gauge partner case, e.g., [527]), the new particle can be light consisting with electroweak precision measurements, thus, the deviation could be greater than the case without *T*-parity. For example, in the littlest Higgs model with *T*-parity (LHT), the ${\varGamma }(h \rightarrow gg)$ normalised to the SM value can be around $60 \%$ at $f = 500$ GeV case [528] (see Fig. 94).

The expected precision for measurements of the Higgs coupling including $h \rightarrow \gamma \gamma$ and $h \rightarrow gg$ branch at ILC are summarised in Sect. 2.3. One of the possibilities to measure the deviation of the ${\varGamma }(h \rightarrow \gamma \gamma )$ is the $\gamma \gamma \rightarrow h \rightarrow b \bar{b}$ mode in photon collider option [529, 530].

*Higgs decay at tree level* The deviation of the SM coupling and new particles would also change the Higgs phenomenology at tree level. The deviation of $ht\bar{t}$ and top partner change the cross section of $ht\bar{t}$ production [531–533]. In LHT, production cross section of the $e^+e^- \rightarrow ht\bar{t}$ normalised to the SM value is about 90 % at $f=1$ TeV [532].

The deviation of *hWW* and *hZZ* couplings (e.g. [534] in LLH model) also change the cross sections of the Higgs-boson production as well as the decay branching ratio.^26^ In some case, the deviation rates of partial decay widths are the same, then the branching ratio of the Higgs decay can be close to the SM prediction [526].

**Fig. 95:**
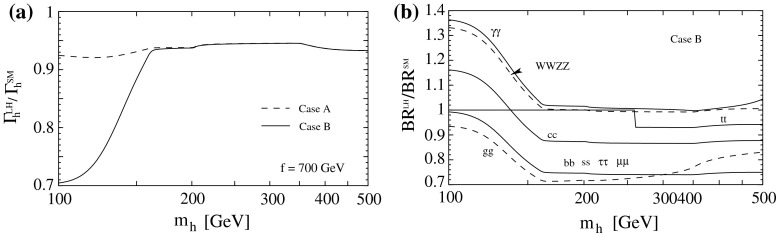
The (**a**) shows the total decay width normalised to the SM value in the LHT (from [526]). The difference between case A and case B comes from the definition of the down-type Yukawa term (for details, see [526]). The (**b**) shows the partial Higgs branching ratios normalised to the SM value (from [526])

However, the down-type Yukawa coupling has model dependence and the couplings could be significantly suppressed in some case of the LHT [526]. Thus, the decay branching ratio of a light Higgs boson ($m_h < 2 m_W$) could significantly change because the dominant decay width, $h \rightarrow b\bar{b}$ is suppressed. Figure 95 shows the correction of the branching ratio from the SM prediction [526].

*Higgs decay to new particles* Another possibility is additional decay branches of Higgs boson into new particles. For example, the lightest new particle in the LHT is the heavy photon which mass is $$\sim $$60 GeV with $$f = 400$$ GeV. If it kinematically possible, the Higgs boson also decays into two heavy photons and the value of the branching ratio could be large ($$> $$80 %) in the 125 GeV Higgs boson case because it decays via the gauge coupling [538, 539]. If the *T*-parity is an exact symmetry, it is the invisible decay. On the other hand, the produced heavy photon decays mainly into SM fermions in such a light Higgs boson case if the *T*-parity is broken by anomaly. The decay width is about $$10^{-1}$$–$$10^{-2}$$ eV [522, 523].

*Additional scalar bosons* In some models, e.g., simple group models, there could be a pseudo-scalar, $\eta$, although the mass depends on the models. The Higgs boson could also decay into $\eta \eta$ and $Z \eta$ [540] if it is kinematically possible. Furthermore, because the *Z*–*h*–$\eta$ coupling cannot appear in product group models, the measurement at ILC helps to distinguish the kind of LH models [541]. Other phenomenology studies for $\eta$ can be found in Refs. [542, 543]. As another example of additional scalars, there is the triplet Higgs boson in the LLH model, although these mass is proportional *f* [544–547].

*Higgs self-coupling* The measurement of Higgs self-coupling is one of the important test for the Higgs boson. In the LH models, the triplet and quartet coupling could slightly change from the SM expectation. Study for *Zhh* process in LLH [548] and the one-loop correction to the *hhh* coupling from vector-like top quarks [549] have been studied.

#### Other direct LH signals

Since the LH model is discussed only in this subsection, we also mention here other signals of the model at future liner collider experiments. The signals can be divided into two categories; direct and indirect signals. The direct signals means the direct productions of new particles predicted by the LH model. The indirect signals are, on the other hand, the LH contributions to the processes whose final states are composed only of SM particles. We consider only the direct signals, while we omit to discuss the indirect ones for want of space. Please see references [534, 550–571] for the indirect signals.

The direct signals can future be divided into two subcategories; the direct productions of coloured particles and non-coloured ones. This is because the LH model requires the cancellation of quadratically divergent corrections to the Higgs mass term from top loop and those of electroweak gauge bosons at one-loop level, and thus the model inevitably predicts both coloured and non-coloured new particles. When the *T*-parity (or some other Z$_2$-symmetry distinguishing SM and new particles) is not imposed on the model like the littlest or the simplest Higgs model, non-coloured new particles will be produced by following two processes: single productions (i.e., $e^+ e^- \rightarrow V_H$) [572–580] and associate productions (i.e., $e^+ e^- \rightarrow V_H + \gamma (Z)$) [581–585], where $V_H$ is the LH partner of the weak gauge boson (heavy gauge boson). On the other hand, when the *T*-parity is imposed like the case of the LHT, non-coloured new particles must be produced in pair (i.e., $e^+ e^- \rightarrow V_H V_H$) [586–591]. For the productions of coloured new particles, associate productions (i.e., $e^+ e^- \rightarrow T + t$) and pair productions (i.e. $e^+ e^- \rightarrow f_H f_H$) are frequently considered to find LH signals [592–594], where *T* is the LH partner of the top quark (top partner) and $f_H$ is the new coloured fermion like the top partner or heavy fermions which are introduced by imposing the *T*-parity on the model.

We first consider the productions of non-coloured new particles. Among several relevant studies reported so far, the most comprehensive one involving realistic numerical simulations has been performed in reference [591]. They have considered following five pair production processes in the framework of the LHT; $e^+ e^- \rightarrow Z_H Z_H$, $Z_H A_H$, $W_H^+ W_H^-$, $e_H^+ e_H^-$, and $\nu _{eH} \bar{\nu }_{eH}$, which are followed by the decays $Z_H \rightarrow A_H h$, $W_H^\pm \rightarrow A_H W^\pm$, $e_H^\pm \rightarrow Z_H e^\pm$, $\nu _{eH} \rightarrow W_H^+ e^-$ ($\bar{\nu }_{eH} \rightarrow W_H^- e^+$), where $e_H^-$ ($e_H^+$) and $\nu _{eH}$ ($\bar{\nu }_{eH}$) are the *T*-parity partners of electron (positron) and electron neutrino (anti-neutrino), respectively. The mass spectrum of the non-coloured new particles used in this study is the following (to be taken as a representative example):

**Table:** 

	$M_{A_H}$	$M_{W_H}$	$M_{Z_H}$	$M_{e_H}$	$M_{\nu _{eH}}$
Mass (GeV)	81.9	368	369	410	400

The above mass spectrum has been obtained by choosing the vacuum expectation value of the global symmetry *f* and the Yukawa coupling of the heavy electron $\kappa _e$ to be 580 GeV and 0.5, respectively.^27^ Flavour-changing effects caused by the heavy lepton Yukawa couplings are implicitly assumed to be negligibly small.

By measuring the energy distribution of visible (SM) particles emitted in each production process, the masses of the non-coloured new particles can be precisely extracted. This is because the initial energy of electron (positron) is completely fixed at the $e^+ e^-$ colliders and thus measuring the energy distribution allow us to reconstruct the process accurately without any assumption of the LHT model. With assuming the integrated luminosity of 500 fb$^{-1}$ at $\sqrt{s} =$ 1 TeV running and use of the four processes, $e^+ e^- \rightarrow Z_H Z_H$, $W_H^+ W_H^-$, $e_H^+ e_H^-$, and $\nu _{eH} \bar{\nu }_{eH}$, the resultant accuracies of the mass extractions turns out to be as follows [591].

**Table:** 

	$M_{A_H}$	$M_{W_H}$	$M_{Z_H}$	$M_{e_H}$	$M_{\nu _{eH}}$
Accuracy (%)	1.3	0.20	0.56	0.46	0.1

Since the relevant physics of the LHT model is described with only two model parameters *f* and $\kappa _e$, the masses of non-coloured new particles are also given by the parameters. Performing these model-independent mass measurements therefore provides strong evidence that the discovered new particles are indeed LHT particles. The parameters *f* and $\kappa _e$ are eventually extracted from the measurements very accurately; *f* and $\kappa _e$ are extracted at accuracies of 0.16 and 0.01 %.

More interestingly, by assuming the vertex structures of the LHT model (i.e. the Lorentz structure, the ratio of right- and left-handed couplings, etc.), it is possible to extract the couplings concerning heavy gauge bosons/heavy leptons through cross section measurements. There are a total of eight vertices concerning the five pair production processes. Extracting all the couplings is therefore possible by measuring the total cross sections of the five processes and the angular distribution (the difference cross section) of the produced heavy gauge boson for appropriate three processes. See Ref. [591] for more detailed strategy to extract the couplings. Though numerical simulations for the three differential cross sections are not performed yet, the measurement accuracies for the five total cross sections have already been obtained as follows.

**Table:** 

$e^+ e^- \rightarrow$	$A_H Z_H$	$Z_H Z_H$	$e_H^+ e_H^-$	$\nu _{eH} \bar{\nu }_{eH}$	$W_H^+ W_H^-$
Accuracy (%)	7.70	0.859	2.72	0.949	0.401

Only $Z_H A_H$ process has been analysed with 500 fb$^{-1}$ data at $\sqrt{s} =$ 500 GeV running, while others have been done with the same luminosity at 1 TeV running.

We next consider the direct productions of coloured new particles. Among several coloured new particles, the most important one is the top partner *T* (and its *T*-parity partner $T_-$), because it is responsible for the cancellation of the quadratically divergent correction to the Higgs mass term from top loop. Since the top partner has a colour-charge, it is expected to be constrained by the LHC experiment when its mass is not heavy. Thus we summarise the current status of the constraint before going to discuss the physics of the top partner at future linear collider experiments.

The most severe limit on the mass of the top partner comes from its pair production process followed by the decay $T \rightarrow b W$ [595]. The limit is $m_T >$ 650 GeV at 95 % CL with assuming BR($T \rightarrow bW$) = 1. Since the top partner has other decay channels like $T \rightarrow tZ/T \rightarrow th$ and the branching fraction to *bW* is typically about 40 %, the actual limit on the mass is $m_T >$ 500 GeV. On the other hand, the *T*-parity partner of the top partner $T_-$ decays into $tA_H$ with BR($T_- \rightarrow t A_H$) $\simeq$ 1. The most severe limit on its mass again comes from its pair production process, which gives $m_{T_-} >$ 420 GeV at 95 % CL when $A_H$ is light enough [596].

The physics of the top partner at future linear collider experiments has been discussed in some details in reference [594]. When $m_T \simeq$ 500 GeV, the cross section of its pair production process ($e^+ e^- \rightarrow T \bar{T}$) is ${\mathscr {O}}(100)$ fb, while that of the associate production process ($e^+ e^- \rightarrow t \bar{T} + \bar{t} T$) is ${\mathscr {O}}(1$–10) fb with appropriate centre-of-mass energy. It has been shown that the Yukawa coupling of the top partner and the coupling of the interaction between *h*, *t*, and *T* can be precisely measured with use of the threshold productions of these processes. Since these couplings are responsible for the cancellation of the quadratically divergent correction to the Higgs mass term from top loop, these measurements will give a strong test of the LH model.

The physics of the *T*-parity partner $T_-$ at future LC experiments has been discussed in some details in reference [593]. When $m_{T_-} \simeq$ 500 GeV, the cross section of its pair production process ($e^+ e^- \rightarrow T_- \bar{T}_-$) is ${\mathscr {O}}(100)$ fb with appropriate centre-of-mass energy. Since $T_-$ decays into $t A_H$, the masses of both $T_-$ and $A_H$ can be precisely measured using the energy distribution of reconstructed top quarks, which will provide an excellent test of the LHT model by comparing this signal with those of non-coloured new particles. Furthermore, it has also been pointed out that the process can be used to discriminate new physics models at the TeV scale. This is because many new physics models predict similar processes, a new coloured particle decaying into *t* and an invisible particle like a squark decaying into *t* and a neutralino in the MSSM.

As a recent review and recent studies for current status of new particles and DM in LHT, please see also [269, 597–599].

### Testing Higgs physics at the photon linear collider^28^

A photon collider (hereafter we use abbreviation PLC – Photon linear collider) is based on photons obtained from laser light back-scattered from high-energy electrons of LC. Various high-energy gamma–gamma and electron–gamma processes can be studied here. With a proper choice of electron beam and laser polarisation, the high-energy photons with high degree polarisation (dependent on energy) can be obtained. The direction of this polarisation can be easily changed by changing the direction of electron and laser polarisation. By converting both electron beams to the photon beams one can study $\gamma \gamma$ interactions in the energy range up to $\sqrt{s_{\gamma \gamma }}\sim 0.8 \cdot \sqrt{s_{ee}}$, whereas by converting one beam only the $e\gamma$ processes can be studied up to $\sqrt{s_{e\gamma }}\sim 0.9 \cdot \sqrt{s_{ee}}$ [600–602].

**Fig. 96:**
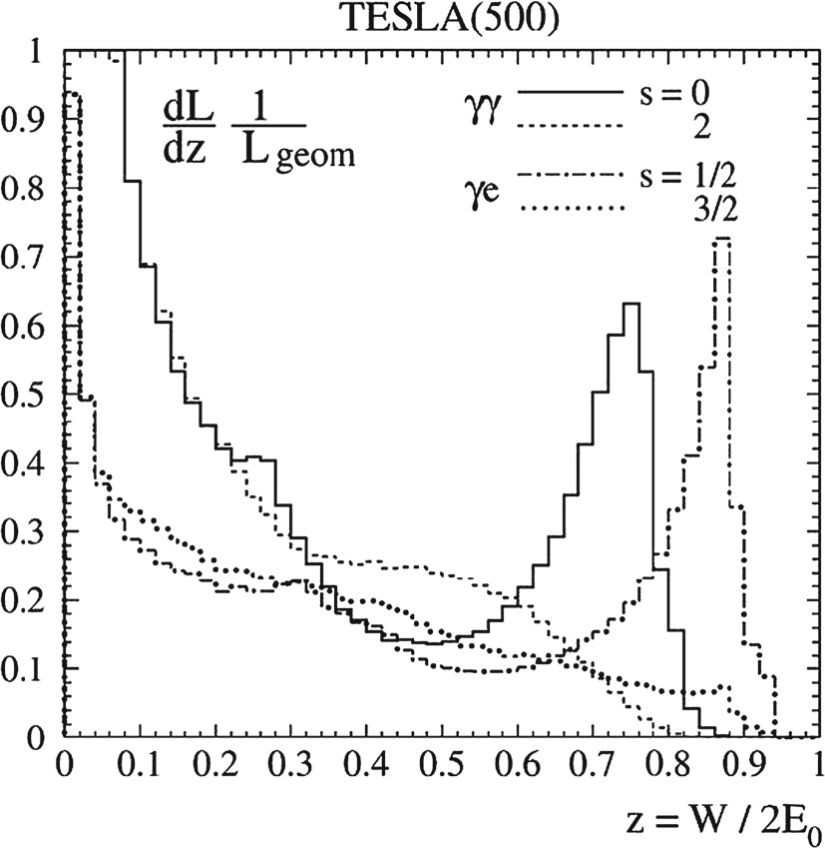
The distribution of $\gamma \gamma$ and $e \gamma$ centre-of-mass energy *W* with respect to the $e^+e^-$ energy (2$E_0$) from simulation of the PLC luminosity spectra [603]. Contributions of various spin states of produced system are shown

In a nominal LC option, i.e. with the electron-beam energy of 250 GeV, the geometric luminosity $L_\mathrm{geom}=12\cdot 10^{34} \mathrm{cm}^{-2}\,\mathrm{s}^{-1}$ can be obtained, which is about four times higher than the expected $e^+ e^-$ luminosity. Still, the luminosity in the high-energy $\gamma \gamma$ peak (see Fig. 96) corresponds to about $\frac{1}{3}$ of the nominal $e^+ e^-$ luminosity – so we expect $L_{\gamma \gamma }(\sqrt{s_{\gamma \gamma }}> 0.65 \cdot \sqrt{s_{ee}})$ equal to about 100 $\mathrm{fb}^{-1}$ per year (400 $\mathrm{fb}^{-1}$ for a whole energy range) [603, 604]. Adjusting the initial electron-beam energy and direction of polarisations of electrons and laser photons at fixed laser photon energy one can vary a shape of the $\gamma \gamma$ effective-mass spectrum.

At a $\gamma \gamma$ collider the neutral C-even resonance with spin 0 can be produced, in contrast to C-odd spin 1 resonances in the $e^+e^-$ collision. Simple change of signs of polarisations of incident electron and laser photon for one beam transforms PLC to a mode with total helicity 2 at its high-energy part. It allows one to determine degree of possible admixture of state with spin 2 in the observed Higgs state. The *s*-channel resonance production of $J^{PC}=0^{+\!+}$ particle allows to perform precise measurement of its properties at PLC.

In summer 2012 a Higgs boson with mass about 125 GeV has been discovered at LHC [94]. We will denote this particle as $\mathscr {H}$. The collected data [605, 606] allow one to conclude that *the SM-like scenario*, suggested e.g. in [607, 608], is realised [609]: all measured $\mathscr {H}$ couplings are close to their SM values in their *absolute value*. Still the following interpretations of these data are discussed: A) ${\mathscr {H}}$ is Higgs boson of the SM. B) We deals with phenomenon beyond SM, with ${\mathscr {H}}$ being some other scalar particle (e.g. one of neutral Higgs bosons of Two Higgs Doublet Model (2HDM) – in particular MSSM, in the ${\textit{CP}}$-conserving 2HDM that are *h* or *H*). In this approach the following opportunities are possible: (1) measured couplings are close to SM values; however, some of them (especially the *ttH* coupling) with a “wrong” sign. (2) In addition some new heavy charged particles, like $H^\pm$ from 2HDM, can contribute to the loop couplings. (3) The observed signal is not due to *one* particle but it is an effect of two or more particles, which were not resolved experimentally – *the degenerated Higgses.* Each of these opportunities can lead to the enhanced or suppressed, as compared to the SM predictions, ${\mathscr {H}}\gamma \gamma$, ${\mathscr {H}}gg$ and ${\mathscr {H}}Z\gamma$ loop-coupling.

The case with the observed Higgs-like signal being due to degenerated Higgses $h_i$ demands a special effort to diagnose it. In this case the numbers of events with production of some particle *x* are proportional to sums like $\sum _i ({\varGamma }^x_i/{\varGamma }^{\mathrm{tot}}_i){\varGamma }^{gg}_i$. Data say nothing about couplings of the individual Higgs particles and there are no experimental reasons in favour of the SM-like scenario for *one* of these scalars. In such case each of degenerated particles have low total width, and there is a hope that the forthcoming measurements at PLC can help to distinguish different states due to much better effective-mass resolution. The comparison of different production mechanisms at LHC, $e^+e^-$ LC and PLC will give essential impact in the problem of resolution of these degenerated states. Below we do not discuss the case with degenerated Higgses with masses $\sim$125 GeV in more detail, concentrating on the case when observed is one Higgs boson $\mathscr {H}$, for which the SM-like scenario is realised.

In the discussion we introduce useful *relative couplings*, defined as ratios of the couplings of each neutral Higgs boson $h^{(i)}$ from the considered model, to the gauge bosons *W* or *Z* and to the quarks or leptons ($j=V (W,Z),u,d,\ell \ldots$), to the corresponding SM couplings: $\chi _j^{(i)}=g_j^{(i)}/g_j^\mathrm{SM}$. Note that all couplings to EW gauge bosons $\chi _V^{(i)}$ are real, while the couplings to fermions are generally complex. For ${\textit{CP}}$-conserving case of 2HDM we have in particular $\chi _j^h$, $\chi _j^H$, $\chi _j^A$ (with $\chi _V^A=0$), where couplings of fermions to *h* and *H* are real, while couplings to *A* are purely imaginary.

*The SM-like scenario* for the observed Higgs ${\mathscr {H}}$, to be identified with some neutral $h^{(i)}$, corresponds to $|\chi _j^{\mathscr {H}}|\approx 1$. Below we assume this scenario is realised at present.

It is well known already since a long time ago that the PLC is a very good observatory of the scalar sector of the SM and beyond SM, leading to important and in many cases complementary to the $e^+e^-$ LC case tests of the EW symmetry breaking mechanism [610–612]. The $e^+e^-$ LC, together with its PLC options ($\gamma \gamma$ and $e \gamma$), is very well suited for the precise study of properties of this newly discovered ${\mathscr {H}}$ particle, and other scalars. In particular, the PLC offers a unique opportunity to study resonant production of Higgs bosons in the process $\gamma \gamma \rightarrow \mathrm{Higgs}$, which is sensitive to charged fundamental particles of the theory. In principle, PLC allows one to study also resonant production of heavier neutral Higgs particles from the extension of the SM. Other physics topic which could be studied well at PLC is the ${\textit{CP}}$ property of Higgs bosons. Below we discuss the most important aspects of the Higgs physics which can be investigated at PLC. Our discussion is based on analyses done during last two decades and takes into account also some recent “realistic” simulations supporting those results.

#### Studies of 125-GeV Higgs ${\mathscr {H}}$

The discussion in this section is related to the case when $\mathscr {H}$ is one of the Higgs bosons $h^{(i)}$ of 2HDM. In the ${\textit{CP}}$-conserving case of 2HDM it can be either *h* or *H*.

**Fig. 97:**
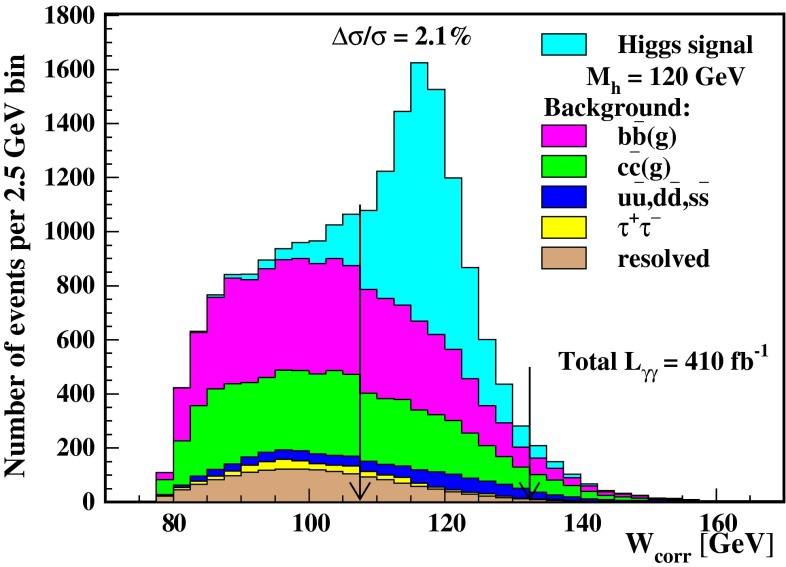
Distributions of the corrected invariant mass, $W_\mathrm{corr}$, for selected $b \bar{b}$ events; contributions of the signal, for $M_{H_\mathrm{SM}} =$ 120 GeV, and of the different background processes, are shown separately [613]

Several NLO analyses of the production at the PLC of a light SM-Higgs boson $H_\mathrm{{SM}}$ decaying into the $b \bar{b}$ final state were performed, including the detector simulation, e.g. [614–617]. These analyses demonstrate a high potential of this collider to measure accurately the Higgs two-photon width. By combining the production rate for $\gamma \gamma \rightarrow H_\mathrm{{SM}}\rightarrow b \bar{b}$ (Fig. 97), to be measured with 2.1 % accuracy, with the measurement of the $\mathrm{BR}(H_{\mathrm{SM}}\rightarrow bb)$ at $e^+e^-$ LC, with accuracy $\sim$ 1 %, the width ${\varGamma }(H_{\mathrm{SM}} \rightarrow \gamma \gamma )$ for $H_{\mathrm{SM}}$ mass of 120 GeV can be determined with precision $\sim$2 %. This can be compared to the present value of the measured at LHC signal strength for 125 GeV $\mathscr {H}$ particle, which ratio to the expected signal for SM Higgs with the same mass (approximately equal to the ratio of $|g_{\gamma \gamma {\mathscr {H} }}|^2/|g_{\gamma \gamma {H_{\mathrm{SM}} }}|^2$), are 1.17$\pm$0.27 and 1.14$^{+0.26}_{-0.23}$ from ATLAS [101] and CMS [618], respectively.

The process $\gamma \gamma \rightarrow {\mathscr {H}}\rightarrow \gamma \gamma$ is also observable at the PLC with reasonable rate [617]. This measurement allows one to measure directly two-photon width of Higgs without assumptions as regards unobserved channels, couplings, etc.

Neutral Higgs resonance couples to photons via loops with charged particles. In the Higgs $\gamma \gamma$ coupling the heavy charged particles, with masses generated by the Higgs mechanism, do not decouple. Therefore the ${\mathscr {H}}\rightarrow \gamma \gamma$ partial width is sensitive to the contributions of charged particles with masses even far beyond the energy of the $\gamma \gamma$ collision. This allows one to recognise which type of extension of the minimal SM is realised. The $H^+$ contribution to the ${\mathscr {H}} \gamma \gamma$ loop coupling is proportional to ${\mathscr {H}} H^+H^-$ coupling, which value and sign can be treated as free parameters of model.^29^ The simplest example gives a 2HDM with type II Yukawa interaction (2HDM II). For a small $m_{12}^2$ parameter, see Sect. 2.6, the contribution of the charged Higgs boson $H^+$ with mass larger than 400 GeV leads to 10% suppression in the ${\mathscr {H}}\rightarrow \gamma \gamma$ decay width as compare to the SM one, for $M_{\mathscr {H}}$ around 120 GeV [607, 608], Table 24 (solution A). The enhancement or decreasing of the ${\mathscr {H}} \gamma \gamma$ coupling is possible, as discussed for 2HDM with various Yukawa interaction models in [276, 619] as well in the inert doublet model^30^ [620, 621].

**Fig. 98:**
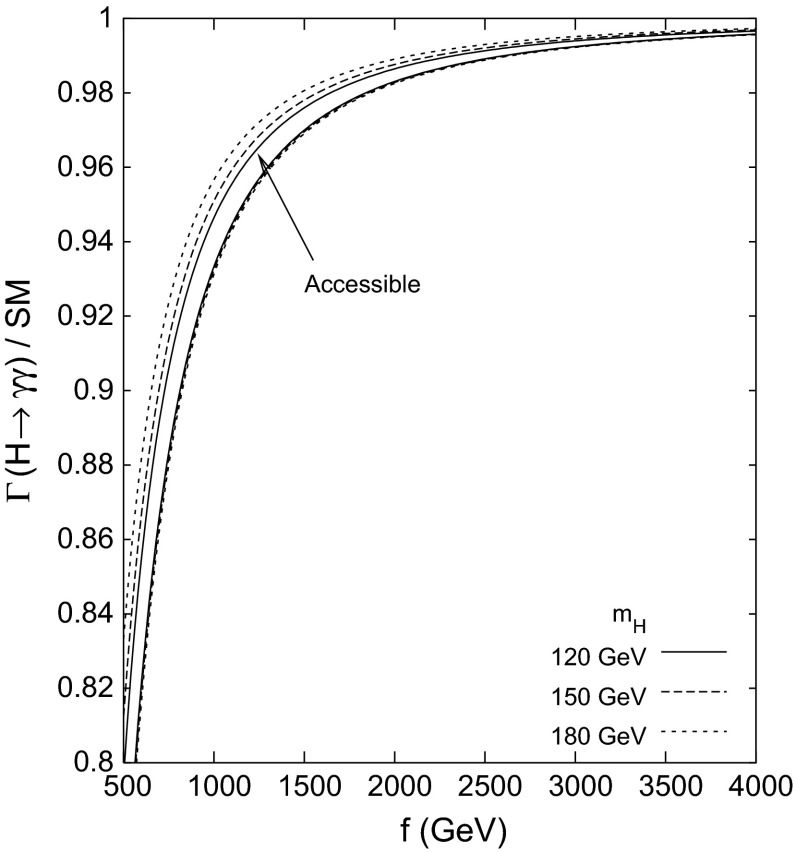
Ratio $\dfrac{{\varGamma }(h \rightarrow \gamma \gamma )}{{\varGamma }(h\rightarrow \gamma \gamma )^{\mathrm{SM}}}$ as a function of the mass scale of the new physics *f* in the Littlest Higgs model [524], for different Higgs-boson masses. “Accessible” indicates the possible variation of the rate for fixed *f* labelfig

In the Littlest Higgs model a 10 % suppression of the $\gamma \gamma$ decay width for $M_{\mathscr {H}}\approx 120$ GeV is expected due to the new heavy particles with mass around 1 TeV at the suitable scale of couplings for these new particles [524, 559], see Fig. 98.

The Higgs $\gamma \gamma$ loop coupling is sensitive to the relative signs of various contributions. For example, in 2HDM II sign of some Yukawa couplings may differ from the SM case, still strength (ie. absolute value) of all squared direct Higgs couplings to *WW* / *ZZ* and fermions being as in the SM. This may lead to the enhancement of the ${\mathscr {H}} \rightarrow \gamma \gamma$ decay-width with respect to the SM predictions, up to 2.28 for a “wrong” sign of the ${\mathscr {H}} tt$ for $M_{\mathscr {H}}=120$ GeV (1.28 for ${\mathscr {H}} \rightarrow gg$ and 1.21 for ${\mathscr {H}} \rightarrow Z\gamma$, respectively) coupling, Table 24 (solution $B_{{\mathscr {H}} t}$), [607].^31^ The “wrong” sign of ${\mathscr {H}}bb$ coupling (solution $B_{{\mathscr {H}} b}$ in Table 24) could lead to a enhancement in the ${\mathscr {H}} \rightarrow gg$, and in the corresponding rate for gluon fusion of Higgs at LHC, similarly as the “wrong” sign of ${\mathscr {H}}tt$ coupling. Such solution is still considered as a possible for 125 GeV ${\mathscr {H}}$ particle [605].

**Table 24:** SM-like realisations in the 2HDM II [607, 608] together with ratios of loop-induced partial widths to their SM values at $M_{\mathscr {H}}=120$ GeV, $M_{H^{\pm }}= 800$ GeV, $|m_{12}^2|\le 40$ GeV$^2$

Solution	Basic couplings	$|\chi _{gg}|^2$	$|\chi _{\gamma \gamma }|^2$	$|\chi _{Z\gamma }|^2$
$A_{{\mathscr {H}}}$	$\chi _V\approx \chi _b\approx \chi _t\approx \pm 1$	1.00	0.90	0.96
$B_{{\mathscr {H}} b}$	$\chi _V\approx -\chi _b\approx \chi _t\approx \pm 1$	1.28	0.87	0.96
$B_{{\mathscr {H}} t}$	$\chi _V\approx \chi _b\approx -\chi _t\approx \pm 1$	1.28	2.28	1.21

The observed Higgs particle can have definite ${\textit{CP}}$ parity or can be admixture of states with different ${\textit{CP}}$ parity (*CP mixing*). In the latter case the PLC provides the best among all colliders place for the study of such mixing. Here, the opportunity to simply vary polarisation of photon beam allows one to study this mixing via dependence of the production cross section on the incident photon polarisation [623–630]. In particular, the change of sign of circular polarisation ($+\!+ \leftrightarrow --$) results in variation of production cross section of the 125-GeV Higgs in 2HDM by up to about 10 %, depending on a degree of ${\textit{CP}}$-admixture. Using mixed circular and linear polarisations of photons gives opportunity for more detailed investigations [631].

The important issue is to measure a Higgs selfcoupling, ${\mathscr {H}}{\mathscr {H}}{\mathscr {H}}$. In the SM this selfcoupling is precisely fixed via Higgs mass (and v.e.v. $v=246$ GeV), while deviations from its SM value would be a clear signal of more complex Higgs sector. Both at the $e^+e^-$ collider and at the $\gamma \gamma$ collider the two neutral Higgs bosons are produced in processes both with and without selfinteraction, namely

$$\begin{aligned}&e^+e^-\rightarrow Z\rightarrow {{\mathscr {H}}(Z\rightarrow Z{\mathscr {H}})}\nonumber \\&\quad \oplus \, e^+e^-\rightarrow Z\rightarrow {Z({\mathscr {H}}\rightarrow {\mathscr {H}}{\mathscr {H}})};\\&\gamma \gamma \rightarrow \text{ loop } \rightarrow { {\mathscr {H}}{\mathscr {H}}}\oplus \gamma \gamma \rightarrow \text{ loop } \rightarrow {{\mathscr {H}}\rightarrow {\mathscr {H}}{\mathscr {H}}}. \end{aligned}$$

In the SM case the cross sections for above processes are rather low but measurable, so that coupling under interest can be extracted, both in the $e^+e^-$ and $\gamma \gamma$, modes of $e^+e^-$ LC, see [632, 633]. The feasibility of this measurement at a PLC has been performed recently in [634]. For Higgs mass of 120 GeV and the integrating luminosity 1000 fb$^{-1}$ the statistical sensitivity as a function of the $\gamma \gamma$ energy for measuring the deviation from the SM Higgs selfcoupling $\lambda =\lambda _\mathrm{SM} (1+\delta \kappa )$ has been estimated. The optimum $\gamma \gamma$ collision energy was found to be around 270 GeV for a such Higgs mass, assuming that large backgrounds due to *WW* / *ZZ* and *bbbb* production can be suppressed for correct assignment of tracks. As a result, the Higgs pair production can be observed with a statistical significance of 5 $\sigma$ by operating the PLC for 5 years.

The smaller but interesting effects are expected in $e\gamma \rightarrow e{\mathscr {H}}$ process with $p_{\bot e}> 30$ GeV, where ${\mathscr {H}}Z\gamma$ vertex can be extracted with reasonable accuracy [635].

#### Studies of heavier Higgses, for 125 GeV ${{\mathscr {H}}=h^{(1)}}$

A direct discovery of other Higgs bosons and measurement of their couplings to gauge bosons and fermions is necessary for clarification the way the SSB is realised. In this section we consider the case when observed 125-GeV Higgs is the lightest neutral Higgs, ${\mathscr {H}}=h^{(1)}$ (in particular in the ${\textit{CP}}$-conserving case this means ${\mathscr {H}}=h$). A single Higgs production at $\gamma \gamma$ collider allows one to explore roughly the same mass region for neutral Higgs bosons at the parent $e^+e^-$ LC but with higher cross section and lower background. The $e\gamma$ collider allows one in principle to test wider mass region in the process $e\gamma \rightarrow eH, eA$, however, with a lower cross section. Before general discussion, we present some properties of one of the simplest Higgs model beyond the minimal SM, namely 2HDM (in particular, also the Higgs sector of MSSM), having in mind that the modern data are in favour of a SM-like scenario. Let us enumerate here some important properties of 2HDM for each neutral Higgs scalar $h^{(i)}$ in the ${\textit{CP}}$-conserving case $h^{(1)}=h$, $h^{(2)}=H$, $h^{(3)}=A$:

(i) *For an arbitrary Yukawa interaction* there are sum rules for coupling of different neutral Higgses to gauge bosons $V=W,\,Z$ and to each separate fermion *f* (quark or lepton)
  82 $$\begin{aligned}&\sum \limits _{i=1}^{3} (\chi _V^{(i)})^2=1. \quad \sum \limits _{i=1}^{3}(\chi _f ^{(i)})^2=1. \end{aligned}$$
  The first sum rule (to the gauge bosons) was discussed e.g. in [353, 636]. The second one was obtained only for Models I and II of Yukawa interaction [637], however, in fact it holds for any Yukawa sector [638].
  In the first sum rule all quantities $\chi _V^{(i)}$ are real. Therefore, in SM-like case (i.e. at $|\chi _V^{(1)}|\approx 1$) both couplings $|\chi _V^{2,3}|$ are small. The couplings entering the second sum rule (for fermions) are generally complex. Therefore this sum rule shows that for $|\chi _f^{(1)}|$ close to 1, either $\left| \chi _f^{(2)}\right| ^2$ and $\left| \chi _f^{(3)}\right| ^2$ are simultaneously small, or $\left| \chi _f^{(2)}\right| ^2 \approx \left| \chi _f^{(3)}\right| ^2$.
(ii) For the 2HDM I there are simple relations, which in the ${\textit{CP}}$ conserved case are as follows:
  83 $$\begin{aligned}&\chi _u^{(h)}=\chi _d^{(h)}\,,\qquad \chi _u^{(H)}=\chi _d^{(H)}\,. \end{aligned}$$
(iii) In the 2HDM II following relations hold:
  - *The pattern relation* among the relative couplings for *each neutral Higgs particle*$h^{(i)}$ [639, 640]:
    84a $$\begin{aligned}&(\chi _u^{(i)} +\chi _d^{(i)})\chi _V^{(i)}=1+\chi _u^{(i)} \chi _d^{(i)} . \end{aligned}$$
  - For each neutral Higgs boson $h^{(i)}$ one can write a horizontal sum rule [641]:
    84b $$\begin{aligned}&|\chi _u^{(i)}|^2\sin ^2\beta +|\chi _d^{(i)}|^2\cos ^2\beta =1\,. \end{aligned}$$

Above, in Table 25, we present benchmark points for the SM-like *h* scenario in the ${\textit{CP}}$-conserving 2HDM II. The total widths for *H* and *A* for various $\chi _t^A=1/\tan \beta$ are shown assuming with $\chi _V^h\approx 0.87$, $|\chi _V^H|=0.5$ and $|\chi _t^h|=1$ for *H* and *A*.^32^

**Table 25:** Total width (in MeV) of *H*, *A* in some benchmark points for the SM-like *h* scenario ($M_h=125$ GeV) in the 2HDM ($\chi _V^h\approx 0.87$, $|\chi _V^H|=0.5$ and $|\chi _t^h|=1$). Results for $\tan \beta =1/7, \, 1 \ \ \mathrm{and} \ \ 7$ are shown

$M_{H,A}$	$\tan \beta =1/7$		$\tan \beta =1$		$\tan \beta =7$	
	${\varGamma }_H$	${\varGamma }_A$	${\varGamma }_H$	${\varGamma }_A$	${\varGamma }_H$	${\varGamma }_A$
200	0.35	$8 \times 10^{-5}$	0.35	$4\times 10^{-3}$	0.4	0.2
300	2.1	$1.2\times 10^{-4}$	2.1	$6\times 10^{-3}$	0.75	0.3
400	138	132	8.8	2.7	2.5	0.45
500	537	524	22.8	10.7	6.1	0.7

In the SM-like *h* scenario it follows from the sum rule (82) that the *W*-contribution to the $H\gamma \gamma$ width is much smaller than that of would-be heavy SM Higgs, with the same mass, $M_{H_\mathrm{SM}}\approx M_H$. At the large $\tan \beta$ also $H\rightarrow tt$, $A\rightarrow tt$ decay widths are extremely small, so that the total widths of *H*, *A* become very small.^33^

Let us compare properties of heavy *H*, *A* in 2HDM with a would-be heavy SM Higgs-boson with the same mass. The cross section for production of such particles in the main gluon–gluon fusion channel, being $\propto {\varGamma }_{H,A}^{gg}{\varGamma }_{H,A} /M_H^3$, is lower than that in SM. At large $\tan \beta$ resonances *H*, *A* become very narrow, as discussed above, besides, the two-gluon decay width become about $1/\tan ^2\beta$ smaller. Consequently, these main at LHC production channels cross section are suppressed by roughly $1/\tan ^4\beta$ w.r.t. the would-be SM Higgs boson with the same mass and *H* and *A* can escape observation in these channels at the LHC. (The same is valid for $e^+e^-$ LC due to small value of $\chi _V^H$ for *H* and $\chi _V^A=0$.)

**Fig. 99:**
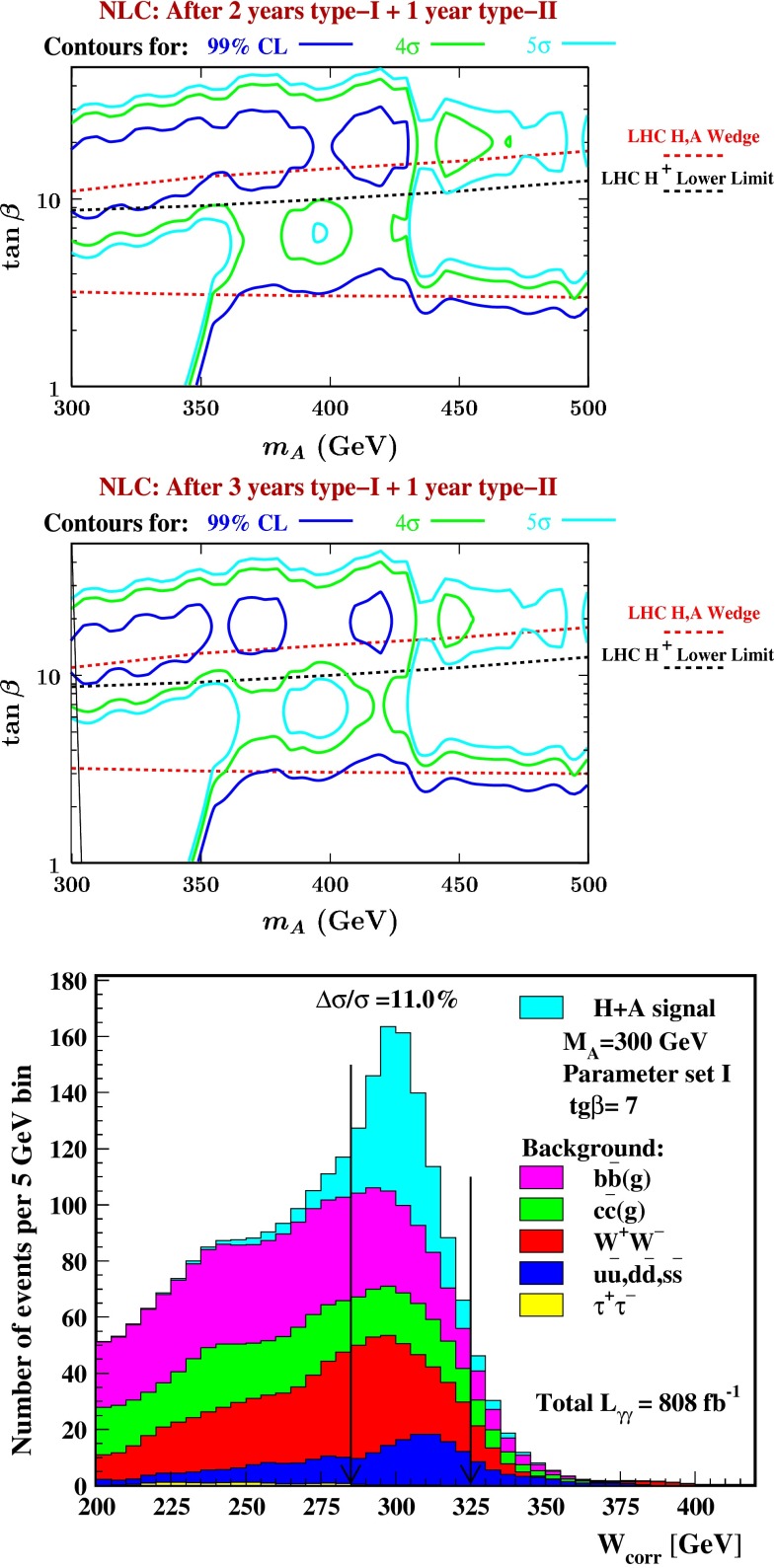
*Top* production of A and H, with parameters corresponding to the LHC wedge, at the $\gamma \gamma$ collider. Exclusion and discovery limits obtained for NLC collider for $\sqrt{ee} =$630 GeV, after 2 or 3 years of operation [642], *Bottom* the case $M_H=M_A=300$ GeV at $\chi _V^H\approx 0$ in the MSSM. Distributions of the corrected invariant mass $W_{corr}$ for selected $b \bar{b}$ events at $\tan \beta =7$ [643]

Moreover, in MSSM with $M_h=125$ GeV we can have heavy and degenerate *H* and *A*, $M_H\approx M_A$. At large $\tan \beta$ the discovery channel of *H* / *A* at LHC is $gg\rightarrow b\bar{b}\rightarrow b\bar{b}H/A$. Nevertheless, in some region of parameters, at intermediate $\tan \beta$, these $H{\, \mathrm{and}\,}\,A$ are elusive at LHC. That is the so-called *LHC wedge region* [644]; see the latest analysis [645]. The PLC allows one to diminish this region of elusiveness, since here the *H* and *A* production is generally not strongly suppressed and the $b\bar{b}$ background is under control [274, 642, 643, 646]. Figure 99 show that PLC allows one to observe joined effect of $H,\,A$ within this wedge region. Precision between 11 and 21 % for $M_A$ equal to 200–300 GeV, $\tan \beta$ = 7 of the Higgs-boson production measurement ($\mu$ =200 GeV (the Higgs mixing parameter) and $A_f=1500$ GeV (the tri-linear Higgs-sfermion couplings)) can be reached after one year [643]. To separate these resonances even in the limiting case $\chi _V^H=0$ is a difficult task, since the total number of expected events is small.

At $\chi _V^H\ne 0$, taking $\chi _V^H \sim$0.3–0.4 as an example (what is allowed by current LHC measurement of couplings of ${\mathscr {H}} = h$ to *ZZ*), an observation of $H\rightarrow ZZ$ decay channel can be good method for the *H* discovery in 2HDM. The signal $\gamma \gamma \rightarrow H\rightarrow WW, ZZ$ interferes with background of $\gamma \gamma \rightarrow WW, ZZ$, what results in irregular structure in the effective-mass distribution of products of reaction $\gamma \gamma \rightarrow WW, ZZ$ (this interference is constructive and destructive below and above resonance, respectively). The study of this irregularity seems to be the best method for discovery of heavy Higgs, decaying to $WW,\, ZZ$ [647], and to measure the corresponding $\phi _{\gamma \gamma }$ phase, provided it couples to *ZZ* / *WW* reasonably strong.^34^

Just as it was described above for the observed 125-GeV Higgs, PLC provides the best among colliders place for the study of spin and the ${\textit{CP}}$ properties of heavy $h^{(2)}$, $h^{(3)}$. That are ${\textit{CP}}$ parity in the ${\textit{CP}}$ conserved case [with ($h^{(2)}$, $h^{(3)}$ = ($H,\,A$)], and (complex) degree of the admixtures of states with different ${\textit{CP}}$ parity, if ${\textit{CP}}$ is violated. This admixture determines dependence on the Higgs production cross section on direction of incident photon polarisation [624, 626–630, 650]. These polarisation measurements are useful in the study of the case when the heavy states $h^{(2)}$, $h^{(3)}$ ($H,\,A$) are degenerated in their masses. A study [631] shows that the 3-years operation of PLC with linear polarisation of photons, the production cross section of the *H* and *A* corresponding to the LHC wedge for MSSM (with mass $\sim 300$ GeV) can be separately measured with precison 20 %. Pure scalar versus pure pseudoscalar states can be distinguished at the $\sim$4.5 $\sigma$ level.

We point out on important difference between the ${\textit{CP}}$ mixed and the mass-degenerate states. In the degeneracy of some resonances *A* and *B* one should distinguish two opportunities:

- Instrumental degeneracy when $|M_B-M_A|>{\varGamma }_B+{\varGamma }_A$, with mass difference within a mass resolution of detector. This effect can be resolved with improving of a resolution of the detector.
- Physical degeneracy when $|M_B-M_A|<{\varGamma }_B+{\varGamma }_A$.

In the ${\textit{CP}}$-conserving case for both types of degeneracy the overlapping of $H,\,A$ resonances does not result in their mixing, and the production of a resonante state cannot vary with change of sign of photon beam polarisation. In the ${\textit{CP}}$-violating case, the overlapping of resonances results in additional mixing of incident $h^{(2)}$, $h^{(3)}$ states, and the production cross section varies with the change of polarisation direction of incident photons.

**Fig. 100:**
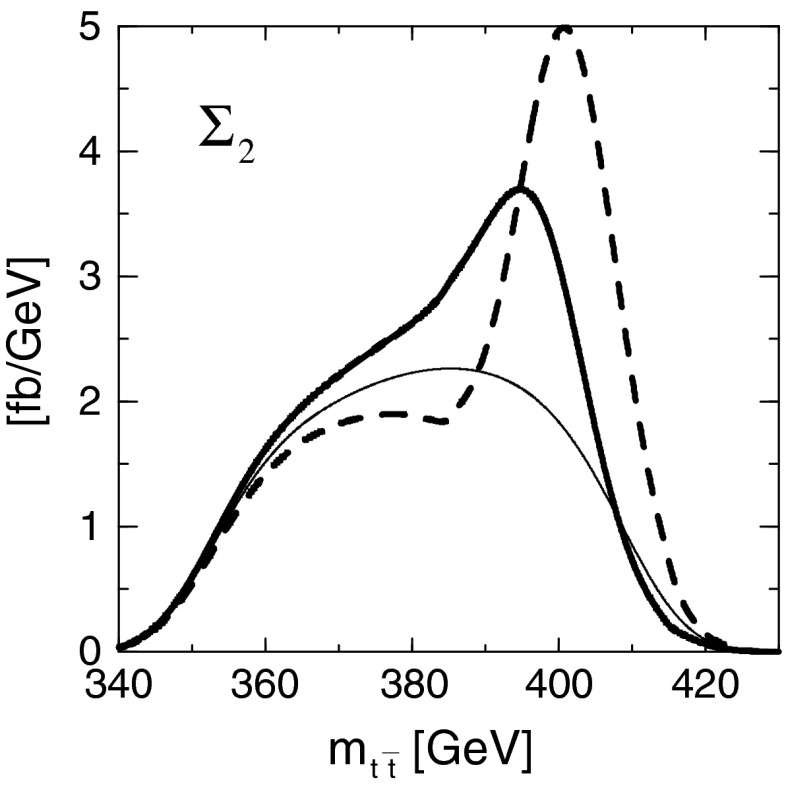
The specific decay angular distributions $\Sigma _i$ in the $\gamma \gamma \rightarrow h^{(i)}\rightarrow t\bar{t}$ process in dependence on the $t\bar{t}$ invariant mass for the scalar (*dashed*) and pseudoscalar (*thick solid*) $h^{(i)}$ with $M_H= 400$ GeV [649]

Another method for study of ${\textit{CP}}$ content of a produced particle provides the measurement of angular distribution of decay products [623, 651, 652]. In the $t \bar{t}$ decay mode one can perform a study of the ${\textit{CP}}$-violation, exploiting fermion polarisation. The interference between the Higgs exchange and the continuum amplitudes can be sizeable for the polarised photon beams, if helicities of the top and antitop quarks are measured. This enables to determine the ${\textit{CP}}$ property of the Higgs boson completely [649, 653], Fig. 100.

The discovery of charged Higgses $H^\pm$ will be a crucial signal of the BSM form of the Higgs sector. These particles can be produced both at the LC ($e^+e^-\rightarrow H^+H^-$) and at the PLC ($\gamma \gamma \rightarrow H^+H^-$). These processes are described well by QED. The $H^+H^-$ production process at PLC has a worse energy-threshold behaviour than the corresponding process at the LC, but a higher cross section. On the other hand, the process $e^+e^-\rightarrow H^+H^-$ can be analysed at LC better by measurements of decay products due to known kinematics. At the PLC the variation of a initial-beam polarisation could be used for checking up the spin of $H^\pm$ [654]. See also the analysis for flavour violation models in [655, 656].

After a $H^\pm$ discovery, the observation of the processes $e^+e^-\rightarrow H^+H^-h$ and $\gamma \gamma \rightarrow H^+H^-h$, $H^+H^-H$, $H^+H^-A$ may provide direct information on a triple Higgs ($H^+H^-h$) coupling $\lambda$, with cross sections in both cases $\propto \alpha ^2\lambda ^2$. The $\gamma \gamma$ collisions are preferable here due to a substantially higher cross section and the opportunity to study polarisation effects in the production process via a variation of the initial photon polarisations.

Synergy of LHC, LC and PLC colliders may be useful in the determination of the Higgs couplings, as different production processes dominating at these colliders lead to different sensitivities to the gauge and Yukawa couplings. For example LC Higgs-strahlung leads to a large sensitivity to the Higgs coupling to the EW gauge bosons, while at PLC $\gamma \gamma$ and $Z\gamma$ loop couplings depend both on the Higgs gauge and Yukawa couplings, as well as on coupling with $H^+$; see the results both for the ${\textit{CP}}$-conserving/${\textit{CP}}$-violating cases in e.g. [652, 657, 658].

## Top and QCD^35^

### Introduction

The experimental studies of electron–positron annihilation into hadrons were historically essential to establish Quantum Chromodynamics (QCD) as *the* theory of the strong interaction: from the measurement of the *R*-ratio ${\sigma _{\text{ had }}/\sigma _t}$ the number of colours could be determined, the discovery of three-jet events at PETRA provided the first direct indication of the gluon, and the measurement of the Bengtson–Zerwas and Nachtmann–Reiter angles illustrated the non-abelian gauge structure of QCD – to name only a few milestones on the road to develop the theory of the strong interactions.

At the Large Electron Positron Collider (LEP) the experimental tests of QCD were further refined. Three-, four-, and even five-jet rates were measured with unprecedented accuracy. These measurements provided important input to constrain the structure constants of the underlying non-abelian gauge group and to determine the QCD coupling constant $\alpha _s$ with high precision. The *R*-ratio and the forward–backward asymmetry were studied in detail including precise investigations of the flavour (in-)dependence. At SLD the measurements were extended to polarised electrons in the initial state. The tremendous experimental effort has been complemented over the time by a similar effort on the theory side: Next-to-leading order (NLO) calculations have been performed for event-shape observables and jet-rates involving jets originating from massless as well as massive quarks. New jet-algorithms with an improved theoretical behaviour were developed. Very recently theoretical predictions for three-jet rates have been extended to next-to-next-to-leading order (NNLO) accuracy. For inclusive hadron production the theoretical predictions have been extended to N$^3$LO accuracy in QCD. Beyond fixed order perturbation theory also power corrections and soft gluon resummation have been considered. All this effort has paved the way to establish QCD as the accepted theory of the strong interaction.

Today QCD is a mature theory and no longer the primary target of experimental studies. Assuming QCD as the underlying theory of strong interaction the precision measurements possible in $e^+e^-$ annihilation can be used to determine fundamental parameters like coupling constants and particle masses. For example three-jet rates at LEP have been used to measure the QCD coupling constant and the *b*-quark mass. Since the small *b*-quark mass leads only to effects of the order of 5 % at the *Z*-resonance (compared to massless *b*-quarks), this example nicely illustrates the impressive theoretical and experimental precision reached. The steadily increasing experimental accuracy together with LHC as a “QCD machine” and the perspective of a future linear collider have kept QCD a very active field, where significant progress has been achieved in the last two decades. Conceptually effective field theories have been further developed with specific realisations for dedicated applications. For example, soft collinear effective theory (SCET) is nowadays used to systematically improve the quality of the perturbative expansion through the resummation of logarithmically enhanced contributions. SCET may also help to deepen our current understanding of factorisation of QCD amplitudes. Applications to the production of top-quark pair production have also demonstrated the power of this approach to assess the impact of non-perturbative corrections. Non-relativistic QCD (NRQCD) provides the well-established theoretical framework to analyse the threshold production of top-quark pair production where binding effects between top quarks are important. The theoretical description of unstable particles in the context of effective field theories have demonstrated another successful application of effective field theories. Theoretical predictions for a future Linear Collider will profit from the improved theoretical understanding in terms of an increased precision. Recently we have witnessed a major breakthrough in the development of technologies for one-loop calculations. One-loop calculations involving multiplicities of five or even more particles in the final state – which were a major bottleneck over several years in the past – are today regularly performed for a variety of different processes. The new techniques have also led to an increased automation of the required calculations. Various programmes are now publicly available to generate NLO matrix elements. Furthermore a standardised interface allows the phase-space integration within MC event generators like for example Sherpa. Also the two-loop technology has seen important progress and is now a continuously growing field. The description of threshold effects in the production of heavy particles notably heavy quarks has been further improved to include higher order corrections in the perturbative expansion.

The detailed understanding of QCD achieved today has been proven essential for the current interpretation of LHC results and the very precise measurements performed so far. Evidently LHC data can also be used for QCD studies in the TeV regime. However, owing to the complicated hadronic environment it will be difficult to reach accuracies at the per-cent level or even below. In contrast $e^+e^-$ Linear Colliders allows one to test QCD at the sub per-cent level at energies above the *Z* resonance. The reachable precision of any measurement involving strongly interacting particles will depend on the ability of making accurate predictions within QCD. QCD studies will thus continue to play an important role at a future Linear Collider. Since non-perturbative effects are intrinsically difficult to assess, the highest accuracy – and thus the most precise tests of the underlying theory – can be reached for systems, where these effects are believed to be small or even negligible. A particular interesting example is provided by top-quark physics. With a mass almost as heavy as a Gold atom the top quark is the heaviest elementary fermion discovered so far.

Top quarks have unique properties, making them a highly interesting research topic on their own right. The large mass leads to an extremely short life time such that top quarks decay before they can form hadronic bound states. This simple observation has several important consequences. First of all the finite width essentially cuts off non-perturbative physics such that top-quark properties can be calculated with high accuracy in perturbative QCD. Top-quark physics thus allows one to study the properties of a ‘bare quark’. In the standard model top quarks decay almost exclusively through electroweak interactions into a *W*-boson and a *b*-quark. The parity-violating decay offers the possibility to study the polarisation of top quarks through the angular distribution of the decay products. Polarisation studies, which are difficult in the case of the lighter quarks since hadronisation usually dilutes the spin information, offer an additional opportunity for very precise tests of the underlying interaction. This is of particular interest since top-quark physics is controlled in the standard model by only ‘two parameters’: The top-quark mass and the relevant Cabbibo–Kobayashi–Maskawa matrix elements. Once these parameters are known top-quark interactions are predicted through the structure of the standard model. In particular all the couplings are fixed through local gauge invariance. Top-quark physics thus allows one to test the consistency of the standard model with high precision. A prominent example is the relation between the top-quark mass and the mass of the *W*-boson. Obviously the accuracy of such tests is connected to the precision with which the top-quark mass – as a most important input parameter – can be determined. While the LHC achieved already an uncertainty in the mass measurements of one GeV, it is expected that a Linear Collider will improve this accuracy by an order of magnitude down to 100 MeV or even below. Using top quarks to test the standard model with high precision and search for new physics is very well motivated. In addition to the high experimental and theoretical accuracy achievable in top-quark measurements, top-quarks provide a particular sensitive probe to search for standard model extensions. Due to their large mass, top quarks are very sensitive to the mechanism of EWSB. In many extensions of the standard model which aim to present an alternative mechanism of EWSB top quarks play a special role. It is thus natural to ask whether the top-quark mass, being so much larger than the masses of the lighter quarks, is indeed produced by the Englert–Brout–Higgs–Guralnik–Hagen–Kibble mechanism. A detailed measurement of the top-quark Yukawa coupling to the Higgs boson, which is very difficult to assess at a hadron collider, will provide a crucial information to answer this question. In the past top quarks have been extensively studied at the Tevatron and the LHC. With exception of the forward–backward charge asymmetry studied at the Tevatron the measurements are in very good agreement with the standard model predictions. However, it should be noted that due to the complex environment at a hadron collider the accuracy is often limited. The top-quark mass which is now measured with sub per cent accuracy represents an important exception. While the measurements at the Tevatron and the LHC are perfectly consistent the precise interpretation of the measured mass value in terms of a renormalised parameter in a specific scheme is still unclear. The mass which is determined from a kinematical reconstruction of the top-quark decay products is assumed to be close to the pole mass. Since precise theoretical predictions for the measured observable are lacking the exact relation between the measured mass and the pole mass has not been quantified so far. An alternative method in which the mass is determined from cross section measurements where the renormalisation is uniquely fixed through a higher order calculation gives consistent results. However, the experimental uncertainties of this method are quite large owing to the weak sensitivity of the total cross section with respect to the top-quark mass. A new method using top-quark pair production in association with an additional jet represents an interesting alternative but will most likely also be limited in precision to one GeV. Although it is not better in precision, the advantage of this method lies in the fact that the method gives a clear interpretation of the measured value in a specific renormalisation scheme. Given the importance of a precise determination of the top-quark mass, going significantly below one GeV may remain the task of a future Linear Collider.

In the following we shall briefly describe in Sect. 3.2 recent progress in QCD with a special emphasis on $e^+e^-$ annihilation. In Sect. 3.3 we summarise new developments in top-quark physics in particular concerning the theoretical understanding of top-quark production at threshold. In the last Section we briefly comment on the physics potential of a future linear collider with respect to QCD and top-quark physics. In particular the prospects of a precise measurement of the top-quark mass are discussed.

### Recent progress in QCD

#### Inclusive hadron production

The inclusive cross section for the production of hadrons in $e^+e^-$ annihilation or alternatively the *R*-ratio is a fundamental observable to be studied at any $e^+e^-$ collider. For hadrons originating from the fragmentation of massless quarks substantial progress has been obtained over the last 10 years. Starting from the $n_f^2\alpha _s^4$ contribution presented in Ref. [659] more than ten years ago the full N$^3$LO result including all colour structures have been derived over the last decade in a ground breaking calculation [660–662]. Using $\sin ^2\theta _W=0.231$ for the sine squared of the weak mixing angle the result for the hadronic decay width of the Z-boson reads [662]:

85 $$\begin{aligned} {\varGamma }_Z= & {} \frac{G_F M_Z^3}{24\pi \sqrt{2}} R^{nc} \end{aligned}$$

86 $$\begin{aligned} R^{nc}= & {} 20.1945 + 20.1945\, a_s \nonumber \\&+\, (28.4587 - 13.0575 + 0)\, a_s^2 \nonumber \\&+\, (-257.825 - 52.8736 - 2.12068)\, a^3_s \nonumber \\&+\, (-1615.17 + 262.656 - 25.5814)\, a^4_s, \end{aligned}$$

with $a_s=\alpha _s(M_Z)/\pi$. The three terms inside the brackets display the non-singlet, axial singlet and vector singlet contributions. An important application of the improved theoretical description is the determination of the QCD coupling constant. It is thus interesting to investigate the impact of the newly calculated correction on the determined $\alpha _s$ value. For $\alpha _s(M_Z)=0.1190$ the impact of the four-loop correction on the extracted $\alpha _s$ value is found to be very small. A shift $\delta \alpha _s=-0.00008$ in the $\alpha _s$ value when extracted from the hadronic cross section is expected. For the quality of the perturbative expansion not only the size of the corrections is important but also the residual renormalisation scale dependence. In Ref. [662] it has been shown that the scale dependence is also improved by including the four-loop contributions. As far as the order in the QCD coupling constant is concerned the *R* ratio is certainly one of the best known QCD observables.

#### Three-jet production at NNLO

Jet production in $e^+e^-$ annihilation is another classical QCD observable. The underlying physical picture explaining the outgoing bundles of hadrons called *jets* is the production of coloured high-energetic partons in a short-distance process. The partons are then assumed to fragment into uncoloured hadrons. As a consequence, the naive expectation is that the fragmentation products somehow share the momentum of the mother parton. This simple picture is reflected in iterative jet algorithms which try to bridge the gap between the experimentally observed hadrons and the partonic final states used in the theoretical predictions. To make contact between theory and experiment, in both analyses the same jet algorithms are applied and the results are compared. In the Born approximation the number of partons is equal to the number of jets. In this case each jet is thus ‘modelled’ by a single parton. Including additional real radiation in higher order predictions allows for the recombination of two or even more partons into one jet and gives thus an improved theoretical description of the jets. Three jet production in $e^+e^-$ annihilation is of particular interest since the three-jet rate is directly proportional to the coupling constant of the strong interaction. Until recently the precision of $\alpha _s$ extracted from three-jet rates was limited due to the unknown NNLO corrections. The main problems which had to be overcome were the evaluation of the two-loop amplitudes for the process $e^+e^-\rightarrow (Z^*,\gamma ^*)\rightarrow q\bar{q} g$ and the systematic cancellation of mass and infrared singularities present in individual contributions. The former problem was solved in Refs. [663–665]. The highly non-trivial combination of virtual corrections, real emission at one-loop order, and double real emission took another five years until completion. Predictions for different observables at NNLO accuracy in QCD have been presented in Refs. [666–673] by two competing groups. The fixed order NNLO calculation lead to a 10 % smaller central value for $\alpha _s$ [674]. In addition the inclusion of the NNLO corrections reduce the variation in $\alpha _s$ extracted from different event-shape observables. The NNLO corrections thus lead to a more coherent description of the data. Furthermore the scale uncertainty is reduced by a factor of 2 compared to the NLO calculation. However, the scale uncertainty still dominates the extraction of $\alpha _s$ when compared to uncertainties due to finite statistics and hadronisation. The scale uncertainty is roughly three times larger than the uncertainty due to hadronisation. In Ref. [675] the fixed order NNLO predictions have been extended by resumming large logarithmic corrections due to multiple soft gluon emission at next-to-leading logarithmic accuracy (NLLA). It turns out that the resummation has very little impact on the central value of $\alpha _s$ determined from different event shapes. However the theoretical uncertainties due to scale variations are slightly increased. As a final result

87 $$\begin{aligned} \alpha _s(M_Z)= & {} 0.1224 \pm 0.0009 \text{(stat.) } \pm 0.0009 \text{(exp.) } \nonumber \\&\pm \, 0.0012 \text{(had.) } \pm 0.0035 \text{(theo.) } \end{aligned}$$

is quoted [675]. Evidently the NNLO predictions will also find application at a future linear collider. Even with a limited statistics a future measurement above the Z resonance will be interesting due to the possibility to further constrain $\alpha _s$ at a high scale. It is also conceivable that the theoretical uncertainties are slightly reduced at higher energies due to the smaller value of $\alpha _s$.

#### NLO QCD corrections to 5-jet production and beyond

At the LEP experiments exclusive production of jet multiplicities up to five jets were studied experimentally. However, until very recently only NLO results for four-jet production were available due to the tremendous growth in complexity of the theoretical calculations. In Ref. [676] the NLO QCD corrections to five-jet production are presented.

The virtual corrections were calculated using generalised unitarity (for more details as regards this method we refer to Sect. 3.2.4), relying to a large extent on amplitudes calculated in Ref. [677] where one-loop corrections to $W^+ + \text{3-jet }$ production in hadronic collisions were studied. The real corrections are calculated using MadFKS [678] – an implementation of the Frixione–Kunszt–Signer (FKS) subtraction scheme [679] into Madgraph. The Durham jet algorithm is used to define the jets. Results for the five-jet rate, differential with respect to the parameter $y_{45}$, which determines the $y_{\text{ cut }}$-value at which a five-jet event becomes a four-jet event, are shown. Furthermore the five-jet rate as function of the jet resolution parameter $y_{\text{ cut }}$ is presented. In addition hadronisation corrections are analysed using the Sherpa event generator. At fixed order in perturbation theory it is found that the scale uncertainty is reduced from about $[-30~\%,+45~\%]$ in LO to about $[-20~\%,+25~\%]$ in NLO. In this analysis the renormalisation scale has been chosen to be $\mu =0.3\sqrt{s}$ and variations up and down by a factor of 2 were investigated. The central scale is chosen smaller than what is usually used for lower jet multiplicities. The reasoning behind this is that for increasing multiplicities the average transverse momentum per jet becomes smaller. This is taken into account by using $\mu =0.3\sqrt{s}$ instead of the more common setting $\mu =\sqrt{s}$. It would be interesting to compare with a dynamical scale like $H_T$, the sum of the ‘transverse energies’, which has been proven in four- and five-jet production at hadron colliders to be a rather useful choice [680–682]. Using in LO $\alpha _s$= 0.130 and in NLO $\alpha _s$= 0.118 NLO corrections of the order of 10–20 % are found. It is noted that using the same value of $\alpha _s$ in LO and NLO would amount to corrections at the level of 45–60 %. Including hadronisation corrections through Sherpa the theoretical results are used to extract $\alpha _s$ from the experimental data. As final result $\alpha _s(M_Z) = 0.1156^{+0.0041}_{-0.0034}$ is quoted which is well consistent with the world average and also shows the large potential of $\alpha _s$ measurements using jet rates for high multiplicities: The uncertainty is similar to the $\alpha _s$ determinations from three-jet rates using NNLO + NLLA predictions [675]. As an interesting observation it is also pointed out in Ref. [676] that hadronisation corrections calculated with standard tools like HERWIG, PYTHIA and ARIADNE are typically large and uncertain unless the tools are matched/tuned to the specific multi-jet environment. It is suggested to use in such cases event generators like SHERPA which incorporates high-multiplicity matrix elements through CKKW matching.

Recently an alternative method to calculate one-loop corrections has been used to calculate the NLO corrections for six- and seven-jet production. The method developed in [683–690] combines the loop integration together with the phase-space integration. Both integrations are done together using Monte Carlo integration. Since the analytic structure of the one-loop integrand is highly non-trivial special techniques have to be developed to enable a numerical integration. In Ref. [691] this technique has been applied to the NLO calculation of the six- and five-jet rate in leading colour approximation. No phenomenological studies are presented. It is, however, shown that the method offers a powerful alternative to existing approaches.

#### Progress at NLO

An essential input for NLO calculations are the one-loop corrections. Four momentum conservation at each vertex attached to the loop does not fix the momentum inside the loop. As a consequence an additional integration over the unconstrained loop momentum is introduced. Since the loop momenta appears not only in the denominator through the propagators but also in the numerator in general tensor integrals have to be evaluated. The traditional method to deal with these tensor integrals is the so-called Passarino–Veltman reduction which allows one to express the tensor integrals in terms of a few basic scalar one-loop integrals [692]. All relevant scalar integrals have been calculated and can be found for example in Refs. [693–695]. In practical applications the Passarino–Veltman reduction procedure may lead to large intermediate expressions when applied analytically to processes with large multiplicities or many different mass scales. An alternative to overcome this problem is to apply the reduction procedure numerically. In this case, however, numerical instabilities may appear in specific phase-space regions where the scalar one-loop integrals degenerate for exceptional momentum configurations. Approaching these exceptional momentum configurations the results behave as “0/0”. Evaluating the limit analytically one finds a well-defined result. The numerical evaluation, however, will typically lead to instabilities unless special precautions are taken to deal with these configurations. In the past various approaches have been developed to stabilise the numerical evaluation of exceptional momentum configurations. Details can be found for example in Refs. [696–705] and references therein. With the steadily increasing computing power of modern CPUs today an alternative approach is frequently used: instead of stabilising the numerical evaluation it is checked during the numerical evaluation whether instabilities were encountered. If this is the case the numerical evaluation of the respective phase-space point is repeated using extended floating point precision. The price to pay in this approach is a slight increase of computing time which is, however, affordable as long as the fraction of points needed to be recomputed remains small.

Beside the numerical evaluation of tensor integrals the significant increase in complexity when studying virtual corrections for processes with large multiplicities is another major bottleneck of one-loop calculations. Here the recently developed method of generalised unitarity may provide a solution. The starting point of this method is the observation that any one-loop amplitude can be written in terms of scalar one-point, two-point, three-point and four-point one-loop integrals. No higher point scalar integrals are required. This observation is a direct consequence of the Passarion–Veltman reduction procedure. Starting from this observation one can reformulate the problem of one-loop calculations: How do we calculate most efficiently the coefficients in this decomposition? One answer to this question is the method proposed by Ossola, Papadopoulos, Pittau (OPP) [706]. The idea of this method is to perform a decomposition at the integrand level: the integrand is decomposed into contributions which integrate to zero or lead to scalar integrals. To derive the decomposition at integrand level internal propagators are set on-shell. As a consequence the integrand factorises into a product of on-shell tree amplitudes. For more details as regards the method of generalised unitarity we refer to the recent review of Ellis, Kunszt, Melnikov and Zanderighi [707]. From the practical point of view the important result is that the algorithm can be implemented numerically and requires as input only on-shell tree amplitudes. For on-shell tree amplitudes very efficient methods to calculate them, like for example the Berends-Giele recursion, exist [708]. In principle it is also possible to use analytic results for the tree-level amplitudes or apply on-shell recursions à la Britto, Cachazo, Feng, and Witten ((BCFW) see for example Ref. [709]). Using tree amplitudes instead of individual Feynman diagrams helps to deal with the increasing complexity of processes for large multiplicities. It may also lead to numerically more stable results since the tree amplitudes are gauge invariant and gauge cancellation – usually occurring in Feynman diagramatic calculations – are avoided. The enormous progress made recently is well documented in the increasing number of publicly available tools to calculate one-loop amplitudes, see for example Refs. [710–715]. As can be seen from recent work e.g. Refs. [691, 716, 717] further progress can be expected in the near future (for the method discussed in Ref. [691] see also the discussion at the end of the previous section). As mentioned already the calculation of real emission processes can be considered as a solved problem since very efficient algorithms to calculate the required Born matrix elements are available. In principle also the cancellation of the infrared and collinear singularities appearing in one-loop amplitudes as well as in the real emission processes can be considered as solved. General algorithms like Catani–Seymour subtraction method [718] or FKS subtraction [679] exist to perform the required calculation. Also here significant progress has been obtained in the recent past towards automation. The required subtractions can now be calculated with a variety of publicly available tools [678, 719–722]. While most of the aforementioned tools have been applied recently to LHC physics it is evident that an application to $e+e-$ annihilation is also possible. It can thus be assumed that for a future Linear Collider all relevant NLO QCD corrections will be available.

### Recent progress in top-quark physics

In the standard model the top quark appears in the third family as up-type partner of the bottom quark. As missing building block of the third family the existence of the top quark was predicted long before its discovery in 1994. Top-quark interactions are fixed through the gauge structure of the standard model. The coupling strengths follow from the local $SU(3)\times SU(2)_L\times U(1)_Y$ gauge invariance. In particular the QCD coupling to the gluons is the same as for the lighter quarks. The coupling to the *Z*-boson involves vector and axial-vector couplings, while the coupling to the *W*-boson is of $V-A$ type. The couplings can be expressed in terms of the third component of the weak isospin $T_3$, the hypercharge *Y* (or alternatively the electric charge *Q*) and the weak mixing angle ${\theta _W}$. For example the coupling to the *Z*-boson reads

88 $$\begin{aligned} -i {e\over \sin {\theta _W}\cos {\theta _W}}\left( T_3\gamma ^\mu {1\over 2}(1-\gamma _5) -\sin ^2{\theta _W}Q\gamma _\mu \right) . \end{aligned}$$

As a matter of fact top-quark specific aspects or more general flavour dependencies enter only through the top-quark mass and the Cabbibo–Kobayashi–Maskawa (CKM) matrix which relates the mass eigenstates and the eigenstates of the weak interaction. Assuming three families and unitarity the CKM matrix elements are highly constrained from indirect measurements. A global fit of available flavour data gives [723]:

89 $$\begin{aligned} V =\left( \begin{array}{c@{\quad }c@{\quad }c} 0.97427 \pm 0.00015 &{} 0.22534 \pm 0.00065 &{} 0.00351^{+0.00015}_{-0.00014}\\ 0.22520 \pm 0.00065 &{} 0.97344 \pm 0.00016 &{} 0.0412^{+0.0011}_{-0.0005}\\ 0.00867^{+0.00029}_{-0.00031}&{} 0.0404^{+0.0011}_{-0.0005} &{} 0.999146^{+0.000021}_{-0.000046} \end{array} \right) \end{aligned}$$

Very recently $V_{tb}$ has been determined also in direct measurements using single-top-quark production at Tevatron and LHC. Combining the various measurements the Particle Data Group quotes [723]:

90 $$\begin{aligned} |V_{tb}|= 0.89 \pm 0.07. \end{aligned}$$

The result is consistent with the indirect measurements. However, the complicated experimental environment leads to large uncertainties. Further improvements can be expected from future measurements at the LHC.

The top-quark mass has been measured at the Tevatron and the LHC with various techniques. At the Tevatron a combination [724] of various D0 and CDF measurements gives

91 $$\begin{aligned} M_t = 173.18\pm 0.56 \text{(stat.) } \pm 0.75 \text{(syst.) } \text{ GeV } . \end{aligned}$$

The measurements performed at the LHC are in perfect agreement with the Tevatron results. For example CMS [725] finds, using lepton + jets final states,

92 $$\begin{aligned} 173.49 \pm 0.43 \text{(stat.+JES) } \pm 0.98 \text{(syst.) } \text{ GeV }. \end{aligned}$$

Strictly speaking the renormalisation scheme of the experimentally determined mass parameter is not properly fixed using a kinematic reconstruction of the top-quark mass. Nevertheless it is usually assumed that the aforementioned mass values correspond to the so-called on-shell/pole mass.

From the precise knowledge of the CKM matrix elements and the top-quark mass all other properties can be predicted within the standard model. Given the large value of $V_{tb}$ the dominant decay of the top quark assuming the SM is the decay into a *W*-boson and a *b*-quark. In LO the top-quark decay width is given by

93 $$\begin{aligned} {\varGamma }(t\rightarrow bW)= & {} \frac{G_F|V_{tb}|^2 M_t^3 }{8\sqrt{2}\pi } \left( 1-\frac{M_W^2}{M_t^2} \right) ^2 \left( 1+\frac{2M_W^2}{M_t^2}\right) . \end{aligned}$$

Higher order electroweak and QCD corrections to the width have been calculated as detailed in the following. In Refs. [726, 727] the electroweak one-loop corrections have been calculated. The NNLO QCD corrections are known for $M_W = 0$ [728] and $M_W\not =0$ [729]. Including the radiative corrections the top quark decay width is approximately ${\varGamma }_t\approx 1.4 \, \mathrm{GeV}$. As mentioned earlier the life time is thus almost an order of magnitude smaller than the typical time scale for hadronisation. The top quark thus decays without forming hadrons.

#### Top-quark decays at next-to-next-to-leading order QCD

In Refs. [728, 729] only the NNLO QCD corrections to the inclusive decay width were calculated. The calculation for massless *W*-bosons of Ref. [728] has been extended in Ref. [729] to include also the effects of the finite *W*-boson mass through an expansion in $M_W^2/M_t^2$. These results have been extended recently in various directions. In Ref. [730] the partial decay widths for top quarks decaying into polarised *W*-bosons is investigated. The partial decay widths are particular interesting since the polarisation of the *W*-boson allows one to test the *tWb* vertex independently from the top-quark production mechanism. Assuming massless *b*-quarks the $V-A$ nature of the charged currents forbids the decay into right-handed *W*-bosons in LO. The measurement of the *W*-polarisation in top-quark decays thus provides a sensitive tool to test the $V-A$ structure and to search for possible extensions of the standard model. Obviously a finite *b*-quark mass leads to calculable corrections. Evidently also higher order corrections which include in general also real emission processes can alter the LO predictions. It is thus very important to calculate the branching fractions

94 $$\begin{aligned} f_{\pm } = {{\varGamma }_\pm \over {\varGamma }(t\rightarrow Wb)}, \quad f_L = {{\varGamma }_L\over {\varGamma }(t\rightarrow Wb)} \end{aligned}$$

where ${\varGamma }(t\rightarrow Wb)$ denotes the inclusive top-quark decay width and ${\varGamma }_{-/+}$ (${\varGamma }_L$) denote the decay width into left/right-handed (longitudinally) polarised *W*-bosons. Similar to what has been done in previous work an expansion in $x=M_W/M_t$ is used in Ref. [730] to calculate the partial decay width in NNLO QCD. For $\alpha _s(M_Z) = 0.1176$ and $M_Z=91.1876$ GeV the results read

95 $$\begin{aligned} F_L= & {} 0.6978 - 0.0075 - 0.0023, \end{aligned}$$

96 $$\begin{aligned} F_+= & {} 0 + 0.00103 + 0.00023, \end{aligned}$$

97 $$\begin{aligned} F_-= & {} 0.3022 + 0.0065 + 0.0021, \end{aligned}$$

where the individual terms correspond to the LO, NLO and NNLO prediction. Note that the ratios in Eq. (94) for the fractions are not expanded in $\alpha _s$. The sum of $F_L$, $F_+$ and $F_-$ is thus equal to one which does not hold anymore if the ratios are expanded in $\alpha _s$. As one can see the NNLO corrections are about one third of the NLO corrections. Since $F_+$ is non-zero only in NLO the evaluation of the NNLO corrections are very important to test the reliability of the theoretical predictions. We observe that $F_+$ remains very small even after the inclusion of the NNLO corrections. Any observation of $F_+$ significantly larger than 0.001 would thus signal new physics. In Ref. [558] the impact of various standard model extensions on the *tWb* vertex have been investigated. In particular the MSSM, a generic two-Higgs-doublet model (2HDM) and a top-colour assisted technicolour model are investigated. In top-colour assisted technicolour models a modification of the left chiral couplings by several per-cent is possible. In Ref. [731] a more detailed analysis of the *W*-boson polarisation, which goes beyond the study of helicity fractions, has been proposed.

The fact that the top quark decays before hadronisation plays a major role. Since the dominant decay is parity violating, the top-quark polarisation of an ensemble of top quarks is accessible through the angular distribution of the decay products. In the Born approximation a straightforward calculation leads to

98 $$\begin{aligned} {1\over {\varGamma }} {\mathrm{d}{\varGamma }\over \mathrm{d}\cos {\vartheta }} = {1\over 2} (1+\alpha _f\cos \vartheta ) \end{aligned}$$

where $\vartheta$ denotes the angle between the direction of flight of the respective top-quark decay product *f* and the top-quark spin in the top-quark rest frame. The parameter $\alpha _f$ measures how efficient a specific decay product analyses the top-quark polarisation. For the *b*-quark one finds $\alpha _b=-0.423$, while for the charged lepton from *W*-boson decay a value of $\alpha _\ell =1$ is found. The NLO corrections are also known and turn out to be small. In Refs. [732, 733] the NNLO corrections for the fully differential decay width have been calculated. The NNLO corrections to differential distributions are found to be small. In Ref. [733] also the *W*-boson helicity fractions have been calculated. The results agree with the aforementioned results of Ref. [730].

#### Two-loop QCD corrections to heavy quark form factors and the forward–backward asymmetry for heavy quarks

The measurements of the forward–backward asymmetry $A_{\mathrm{FB}}^b$ for *b*-quarks differ significantly from the standard model predictions [734]. The theoretical predictions take into account NNLO QCD corrections, however, the *b*-quark mass has been neglected at NNLO. The forward–backward asymmetry for massive quarks may be calculated from the fully differential cross section. As far as the two-loop QCD corrections are concerned this requires the calculation of the two-loop form factor for heavy quarks. These corrections have been calculated recently. In Ref. [735] the NNLO QCD corrections for the vector form factor are calculated. In Ref. [736] the results are extended to the axial-vector form factor. The anomaly contribution has been studied in Ref. [737]. The two-loop corrections need to be combined with the one-loop corrections for real emission and the Born approximation for double real emission. All individual contributions are of order $\alpha _s^2$ and thus contribute. The cancellation of the collinear and soft singularities encountered in the different contributions is highly non-trivial. In Refs. [738, 739] ‘antenna functions’ are derived, which match the singular contributions in the double real emission processes. As an important result also the integrated antenna functions are computed in Refs. [738, 739]. In principle all building blocks are now available to calculate the differential cross section for heavy quark production in NNLO accuracy in QCD. Evidently these results, once available, can also be applied to top-quark pair production.

#### Threshold cross section

Threshold production of top-quark pairs in electron–positron annihilation is an unique process where one can extract the top-quark mass through a threshold scan by measuring the total cross section $\sigma (e^+e^-\rightarrow t\bar{t})$. It is a counting experiment of the production rate of the colour singlet $t\bar{t}$ bound state. Therefore the measurement of the threshold cross section for $e^+ e^-\rightarrow t\bar{t}$ is very clean experimentally as well as theoretically concerning QCD non-perturbative effects.

**Fig. 101:**
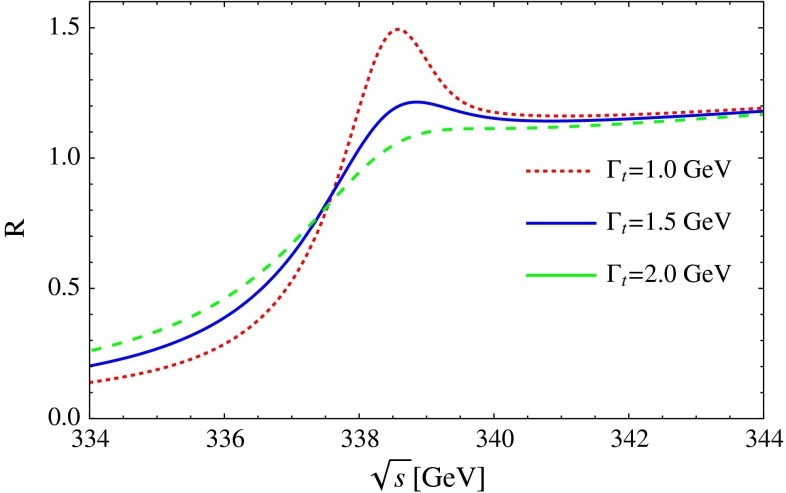
The top quark production cross section *R* for $m_t=170\,\mathrm{GeV}$ and three values for top quark width. The LO formula for the cross section and $\alpha _s(30\mathrm{GeV})=0.142$ is used

The $t\bar{t}$ cross section normalised to the point particle cross section near threshold [740, 741] can be written at LO as

99 $$\begin{aligned} R_{t\bar{t}} = \left( \frac{6\pi N_c e_t^2}{m_t^2}\right) \mathrm{Im} \, G_c(\mathbf {0}, \mathbf {0};\,E+i{\varGamma }_t), \end{aligned}$$

where $E=\sqrt{s} -2m_t$ and $G_c(\mathbf {r}^{\, '}, \mathbf {r}; E+i{\varGamma }_t)$ is the non-relativistic Coulomb Green function. The Green function contains resonances at energies

$$\begin{aligned} E_n=-m_t (C_F \alpha _s)^2/(4n^2) \end{aligned}$$

corresponding to Coulomb boundstates, and its residue is given by the Coulomb wave function $|\psi _n(0)|^2{=}(m_t \alpha _s C_F)^3/(8\pi n^3)$:100Thus the peak position and the magnitude of the cross section is determined by the Coulomb energy levels $E_n$ and the wave-functions $|\psi _n(\mathbf {0})|^2$, respectively. In practice the resonance structure of $G_c$ is smeared due to the large top quark width ${\varGamma }_t \sim 1.4\, \mathrm{GeV}$. In Fig. 101 the threshold cross section is shown for $m_t=170 \mathrm{GeV}$ varying the top-quark width. Only the $n=1$ ground-state peak can be seen for ${\varGamma }_t =$1.0–1.5 GeV as rather wide prominence of the cross section, and the resonance states are completely smeared out creating a flat plateau for ${\varGamma }_t=2\, \mathrm{GeV}$. Although the resonant structures are washed out for a large top-quark width, it is still possible to extract top-quark parameters, $m_t, {\varGamma }_t$ and also $\alpha _s$ by performing a threshold scan, provided a precise theory prediction for the total cross section is at hand.

*QCD corrections* Studies of top quark production near threshold [742–745] at linear colliders were started several decades ago, and NNLO QCD corrections were completed by several groups [746–752] and summarised in Ref. [753]. One main achievement there was the stabilisation of the peak position against QCD corrections taking into account of renormalon cancellation using short-distance masses like 1S-, kinetic-, PS- masses. However, despite the completion of the second order QCD corrections the normalisation of the total cross section still suffers from an uncertainty of about 20 %.

**Fig. 102:**
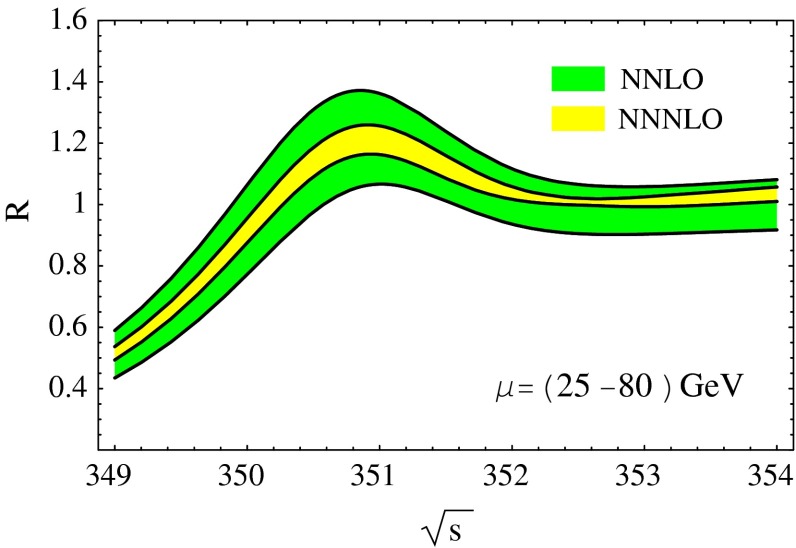
Total cross section for top quark production near threshold at NNNLO (with an estimated third order matching coefficients) and NNLO from [761], where a scale variation of $(20- 80)\, \mathrm{GeV}$ is shown by the *coloured bands*. A top quark PS mass $m_\mathrm{PS}(20\mathrm{GeV})=175\, \mathrm{GeV}$ is used

**Fig. 103:**
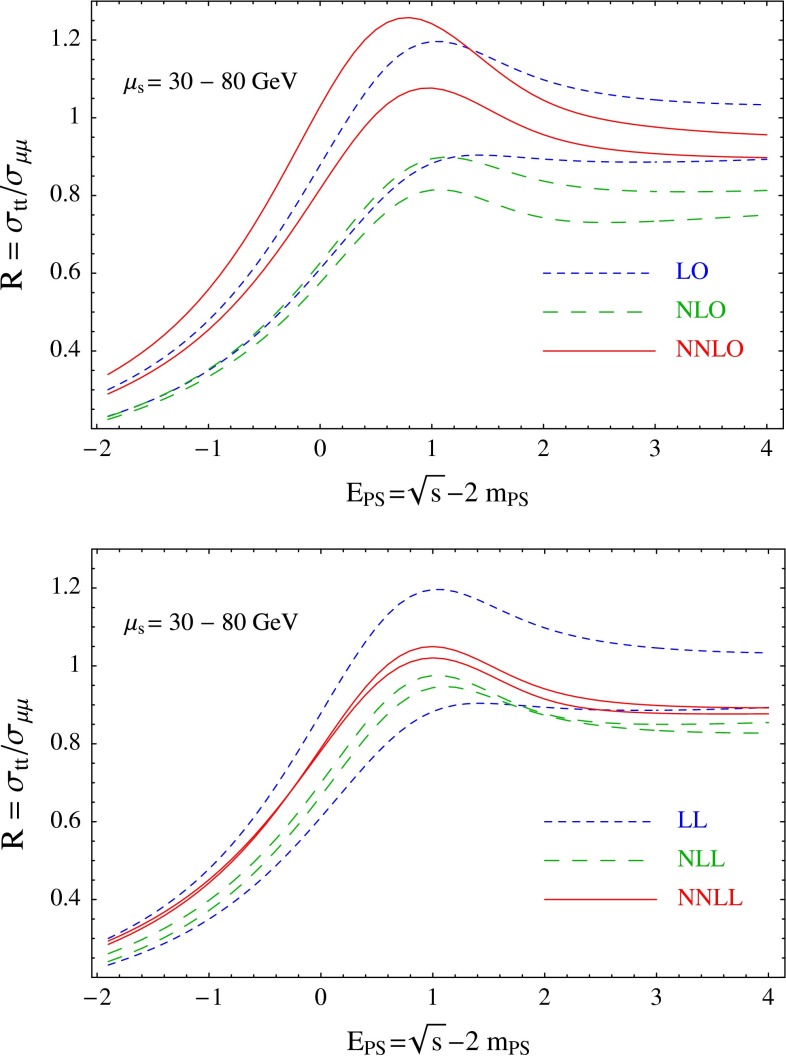
The threshold cross section at fixed order (*upper pannel*) and renormalisation group improvement (*lower pannel*) is shown from Ref. [756]. The bands between *two coloured lines* at each orders show the scale dependence of the results. The RG improved cross sections are stable against scale variation, while fixed order result suffers from large dependence on values of $\mu _s$

There are efforts to improve the accuracy of the NNLO total cross section. These include the resummation of potentially large logarithms by renormalisation group (RG) methods [754–758] and by brute-force computations of NNNLO corrections [759–763] to increase the precision of the cross section. Figure 102 shows the NNNLO result (using an ad hoc estimate of some third order matching coefficients) [761] compared to the NNLO cross section. The coloured bands correspond to the uncertainty originating from a QCD renormalisation scale variation between 25 and $80\,\mathrm{GeV}$. A significant reduction of the scale dependence is observed when going to NNNLO comparing with the NNLO result. In Fig. 103 the RG improved total cross section [754–758] is shown, where the uppper/lower pannels show the result with fixed order/RG improvement, respectively. Two curves at each order are obtained by varying the soft scale $\mu _s$ between (30–80) GeV. The large scale dependence of the fixed order curves is improved by RG resummation in the lower pannel. The plot shows that the cross section at the peak position has scale dependence of order 2 %. The most complete analysis in RG approach has been performed in [758], where new ultra-soft NNLL contributions [757] are included. These two approaches, NNNLO computation and RG improvement to NNLL, are complementary to each other. The fixed order computation provides the non-logarithmic contributions, while the RG improvement reveals the structure of the potentially large logarithmic terms to all orders. Therefore it is expected that the theory prediction of $t\bar{t}$ cross section with $\delta \sigma _{t\bar{t}}/\sigma _{t\bar{t}} =$ 2–3 % will be possible by a combination of the two approaches as far as QCD corrections are concerned. For such a high precision more dedicated theoretical studies will be needed, for instance, the calculation of electroweak effects and final-state interactions in top-quark decays.

*Electroweak corrections and effect of unstable top* In early studies of the $e^+ e^- \rightarrow t\bar{t}$ threshold it was recognised [740, 741] that the effect of the top quark width can be consistently incorporated into the computation of the total cross section by the replacement $E\rightarrow E+i{\varGamma }_t$. This prescription works well up to NLO, but it turns out that in NNLO an uncancelled ultraviolet divergence appears, which is proportional to the top-quark width (in dimensional regularisation an example of such a term is $R_{t\bar{t}}\sim \alpha _s {\varGamma }_t/\epsilon$). This is a signal of an improper treatment of electroweak effects, and the solution of this problem is to abandon the amplitude $e^+ e^- \rightarrow t\bar{t}$ where the unstable $t\bar{t}$ is treated as a final state of the S-matrix. Physical amplitudes should treat stable particles as final states of S-matrix, i.e. $e^+ e^- \rightarrow t\bar{t} \rightarrow (bW^-) (\bar{b}W^+)$^36^ and the unstable particles can appear only as intermediate states.

Electroweak corrections to the production vertex $t\bar{t}-\gamma /Z$ were first described in [764] and re-derived in [765, 766]. In the later refence it is readily realised that amplitudes for single top production, e.g. $e^+ e^- \rightarrow t b W$, and even no-top quark production $e^+ e^- \rightarrow b W^+ \bar{b} W^-$ can contribute to (or mix with) the top-pair production because the physical final state is the same.

The top-quark width is generated by the EW interaction, $t\rightarrow b W$, therefore the effects of the top-quark finite width are intimately related to the EW corrections of the process. To take into account certain electroweak non-resonant effects a method referred to as *phase-space matching* was introduced in [767, 768].

This idea has been further developped and rephrased in the framework of an effective theory for unstable particle [769, 770]. (See Refs. [771, 772] for an application of the method to *W*-pair production in $e^+ e^-$ annihilation.) A systematic analysis of the electroweak effects in top-quark pair production has started rather recently, and NLO electroweak non-resonant contributions were computed [773], e.g. $R(e^+e^-\rightarrow t\bar{b}W^-)\sim \alpha _\mathrm{EW}$, where resonant (on-shell) top quarks decay and the final state $(b W^+) (\bar{b} W^+)$ is measured assuming stable *W*-bosons and *b*-quarks. In this work invariant mass cuts on the top-quark and antitop-quark decay products are implemented. It is found that the non-resonant correction results in a negative 5 % shift of the total cross section which is almost energy independent, in agreement with Ref. [768]. The dominant NNLO non-resonant corrections were computed in Refs. [774, 775] and it was shown that the single resonant amplitudes (e.g. $e^+ e^- \rightarrow t (\bar{b} W^-) g$) provide the counter terms for the uncancelled ultraviolet divergence $\alpha _s {\varGamma }/\epsilon$ discussed previously for the double resonant $e^+ e^- \rightarrow t\bar{t}$ amplitude at NNLO QCD. Therefore, the non-resonant corrections provide together with NNLO QCD a consistent treatment of top quark width effects.

It is also known that the final-state corrections [776, 777] between top quarks and decay products have to be considered for observables other than the total cross section. A systematic analysis of these effects is still missing beyond NLO. Dedicated studies of the electroweak corrections to the threshold cross section have started rather recently.

**Fig. 104:**
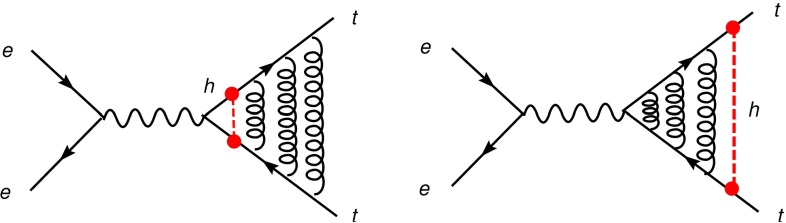
Corrections due to Higgs exchange in $e^+ e^-\rightarrow t\bar{t}$. In the *left diagram* the Higgs exchange contributes to the production vertex for $\gamma t\bar{t}, Zt\bar{t}$, which occurs at short distance when the $t\bar{t}$-pair is separated by $r\sim 1/m_t$. In the *right diagram* Higgs exchanges occurs after bound-state formation between top and anti-top quarks separated by the scale of the bound state $r\sim 1/(m_t \alpha _s)$

*Influence of the Higgs boson on the total cross section* In the SM the large top-quark mass leads to a large top-quark Yukawa coupling to the Higgs boson, therefore it is expected that Higgs boson exchange in top-quark production may lead to observable corrections. Such a Higgs exchange effect appears in two different ways in top and anti-top production near threshold (see Fig. 104). One is a short-distance contribution which enhances the top quark production vertex as $\bar{t} \gamma ^\mu t \rightarrow (1+c_h) \bar{t} \gamma ^\mu t$. The one-loop Higgs correction $c_h^{(1)}$ was determined in Refs. [764], and Higgs and EW mixed two-loop correction $c_{h}^{(2)}$ in Ref. [778]. The enhancement factor for the cross section is given by

101 $$\begin{aligned} \delta R/R_\mathrm{LO}\approx 2 c_h^{(1)} = 6.7/3.4/0.9\times 10^{-2} \end{aligned}$$

using $$m_h=120/200/500\, \mathrm{GeV}$$.

In addition, there is a long-distance effect described by the Yukawa potential $V_h(r)$ for the top quark pair:

102 $$\begin{aligned} V_h(r)= & {} -\frac{y_t^2}{8\pi } \frac{e^{-m_h r}}{r} \simeq -\frac{y_t^2}{2m_h^2}\delta (\mathbf {r}), \end{aligned}$$

where the second expression is a good approximation for $m_h r \gg 1$ assuming $m_h\sim 125\,\mathrm{GeV}$ and $r\sim (m_t \alpha _s)^{-1}$. In the SM the Yukawa coupling is related to the top-quark mass by $y_t=\sqrt{2} e m_t/(s_W M_W)$.

**Fig. 105:**
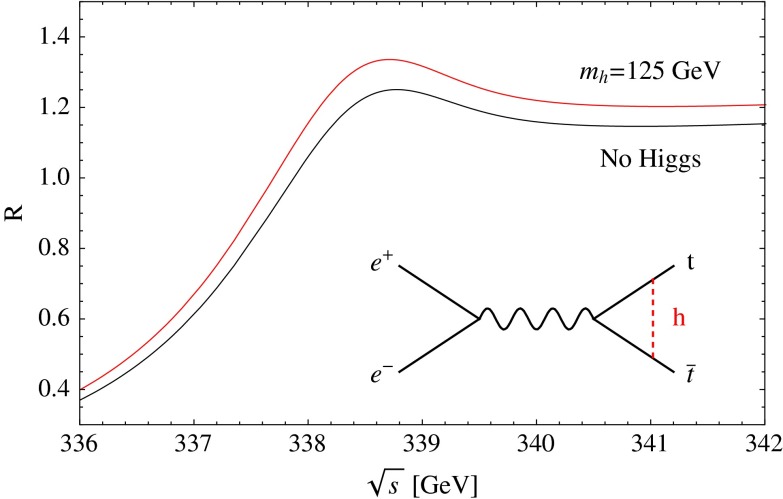
Cross section for $e^+ e^- \rightarrow t\bar{t}$ for $m_t=170\, \mathrm{GeV}$ with/without one-loop Higgs boson corrections. A Higgs-boson mass of $m_h=125\, \mathrm{GeV}$ is used

In Fig. 105 the threshold cross section is shown taking into account of Higgs loop effects through $c_h$ and $V_h$. One can see that the threshold cross section gets an almost energy independent enhancement. The Higgs potential $V_h$ produces corrections to the energy and to the wave function as

103 $$\begin{aligned}&\delta E_1/E_\mathrm{LO} = 3.2/1.2/0.2\times 10^{-2}, \nonumber \\&\delta |\Psi _{1}(0)|^2/ |\Psi _\mathrm{LO}(0)|^2 = 4.6/1.6/0.3\times 10^{-2}, \end{aligned}$$

using $$m_t=175\, \mathrm{GeV},$$$$\mu =30\,$$$$\mathrm{GeV}$$ and $$m_h=120/200/500\,\mathrm{GeV}$$, respectively. The above value for $$\delta E_1$$ can be translated into a shift $$\delta m_t=25/9/1 \, \mathrm{MeV}$$ of the top-quark mass determined in a threshold scan.

*Distribution and Asymmetry* In the threshold production, the top-quark momentum $\mathbf {p}_t$ can be reconstructed from its decay products. Therefore the top-quark momentum distribution [742–744] provides complementary information. Theoretically it is given by

104 $$\begin{aligned}&\frac{1}{\sigma _0} \frac{\text {d}\sigma _{LO}}{\text {d} p_t} (e^+ e^- \rightarrow t\bar{t})\nonumber \\&\quad = \frac{p_t^2}{2\pi ^2} \left( \frac{6\pi N_c e_t^2}{m_t^2} \right) {\varGamma }_t \, | \tilde{G}_c( \mathbf {p}, \mathbf {r}=\mathbf {0}; E+i {\varGamma }_t)|^2, \end{aligned}$$

where $\tilde{G}_c(\mathbf {p},\mathbf {r};E+i{\varGamma }_t)$ is the Fourier transformation of the Coulomb Green function. For the momentum distribution NNLO QCD results [747, 752] are available in the literature.

**Fig. 106:**
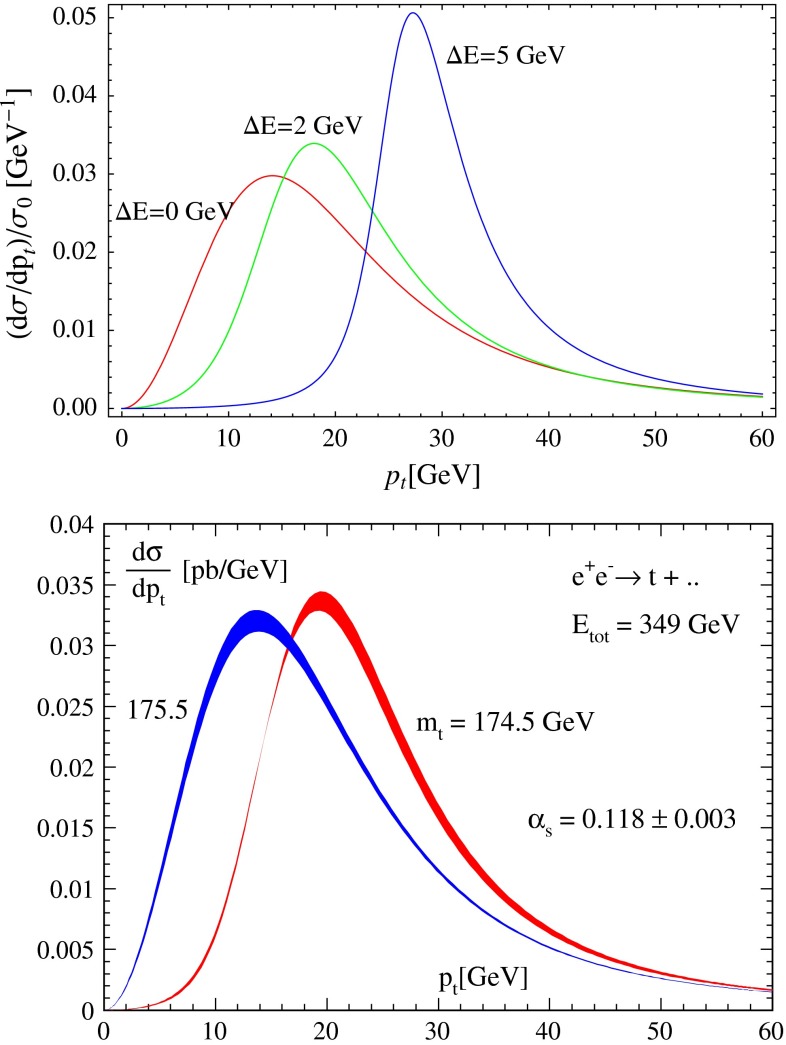
Top quark momentum distribution at ${\varDelta } E= E- E_{1}= 0, 2, 5$ GeV (*top*) for $m_t=170$ GeV and top-quark mass dependence (*bottom*) on the momentum distribution

Figure 106 shows the momentum distribution at specific energy points ${\varDelta } E=0, 2, 5$ GeV (left panel) and for different top-quark masses. In the lower panel the bands correspond to the uncertainty of the QCD coupling constant assuming $\alpha _s=0.118\pm 0.003$. As the Green function $\tilde{G}_c(\mathbf {p}, \mathbf {r}; E+i{\varGamma })$ is essentially the momentum space wave function averaged over the resonances, a measurement of the top-quark momentum distribution gives information on the bound-state wave function $\tilde{\phi }(\mathbf {p})$. Therefore the momentum space distribution gives independent information on the bound state and can be used to test the understanding of the QCD dynamics.

Another useful observable which can be measured in top-quark production near threshold is the forward–backward asymmetry defined as

105 $$\begin{aligned} A_{\mathrm{FB}}= & {} \frac{1}{\sigma _{t\bar{t}}} \left\{ \int _{0}^1 d\cos \theta -\int _{-1}^0 d\cos \theta \right\} \frac{d\sigma (e^+e^-\rightarrow t\bar{t})}{d\cos \theta }. \end{aligned}$$

At lepton colliders top-quark pair production occurs through $e^+e^-\rightarrow \gamma ^*/Z^*\rightarrow t\bar{t}$ and the forward–backward asymmetry receives a non-zero contribution from the interference of vector and axial-vector couplings. Vector and axial-vector interactions produces s-wave and p-wave bound states, respectively, due to angular momentum conservation. Therefore the forward–backward asymmetry is sensitive to the interference between s-wave and p-wave top-quark production. The asymmetry is sensitive to the top-quark width ${\varGamma }_t$ because the s-wave and p-wave overlap is non-zero due to ${\varGamma }_t$.

**Fig. 107:**
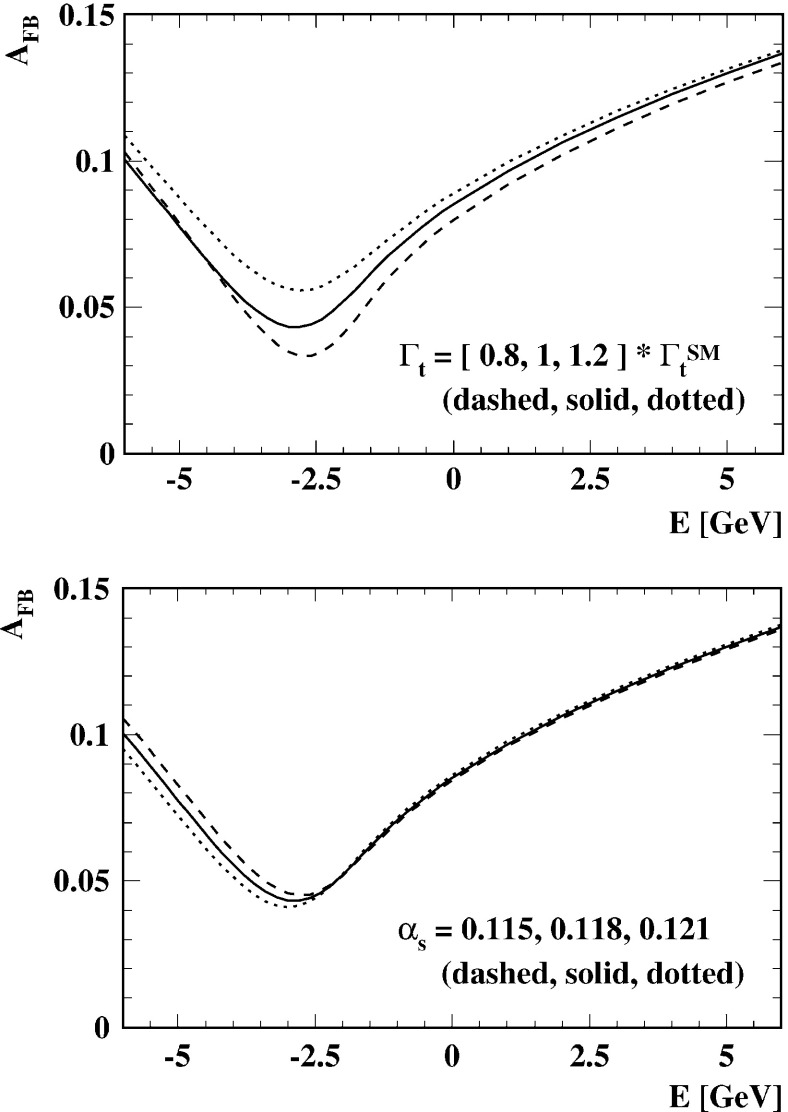
Dependence of the forward–backward asymmetry $A_\mathrm{FB}$ on the top quark width (*upper plot*) and the strong coupling $\alpha _s$ (*lower plot*). Figures are taken from Ref. [779]

In Fig. 107 the forward–backward asymmetry is plotted as a function of energy *E*. Top and bottom panels show the dependence on ${\varGamma }_t$ and $\alpha$, respectively. As discussed above the asymmetry $A_{\mathrm{FB}}$ is an effect of $\gamma$ and *Z*-boson interference. Therefore, the asymmetry provides useful information on the mechanism of top-quark production near threshold.

#### Top-quark production in the continuum

The total cross section for the production of heavy quarks in electron–positron annihilation has been calculated in Refs. [780–783] at order $\alpha _s^2$ in QCD. The results are not applicable very close to the threshold since in that region Coulomb effects lead to $1/\beta$ corrections where $\beta$ denotes the velocity of the top quark. For reliable predictions in the threshold region these contributions need to be resummed (see also the discussion in the previous section). In Ref. [783] it has been estimated that the fixed order results should be applicable in the case of top-quark pair production, provided that the centre-of-mass energy is about 12 GeV above threshold. In Ref. [784] the results have been extended to order $\alpha _s^3$. In particular the quartic mass corrections with respect to the massless calculation were calculated. Using the minimal subtraction scheme (MS) to renormalise the mass parameters, sizeable corrections were found in order $\alpha _s^3$. However, it is also shown in Ref. [784] that using the invariant mass $\hat{m}$ defined through

106 $$\begin{aligned} m(\mu ) = \hat{m} \exp \left\{ \int da {\gamma _m(a)\over a\beta (a)}\right\} , \end{aligned}$$

where $m(\mu )$ denotes the running mass, $\gamma _m$ the anomalous dimension of $m(\mu )$ and $\beta (a)$ the QCD beta function in terms of $a=\alpha _s/\pi$, the convergence of the perturbative expansion can be improved. As discussed in Sect. 3.3.2 the work on the differential cross section at order $\alpha _s^2$ is still on-going. In Refs. [785, 786] jet observables in top-quark pair production at high energy have been investigated. The process is characterised by different mass scales: the centre-of-mass energy $\sqrt{s}$, the top-quark mass $M_t$, the top-quark width ${\varGamma }_t$ and ${\Lambda }_{\mathrm{QCD}}$. Large logarithmic corrections connected with the different mass scales are resummed in Ref. [785] at next-to-leading logarithmic accuracy. This requires the introduction of soft functions capturing non-perturbative soft QCD effects. The soft functions can be obtained from massless dijet events. In Ref. [786] the application to top-quark mass measurements is discussed. In particular it is demonstrated that a top-quark mass measurement with a precision of ${\Lambda }_{\mathrm{QCD}}$ is possible, significantly above the production threshold.

### Physics potential

The excellent possibilities for precision top-quark measurements at $e^+e^-$ colliders have been confirmed by experimental studies of the physics potential of linear colliders, which, in particular in the framework of recent reports of the CLIC and ILC physics and detector projects, often are based on full detector simulations. Particular emphasis has been placed on the measurement of the top-quark mass, which has been studied both at and above threshold, and on the study of the $t\bar{t}Z/\gamma ^*$ vertex through the measurement of asymmetries. For all of these measurements, precise flavour tagging and excellent jet reconstruction are crucial to identify and precisely reconstruct top-quark pair events. The detectors being developed for linear colliders provide these capabilities, and, together with the rather modest background levels in $e^+e^-$ collisions, allow one to acquire high-statistics high-purity top-quark samples. In the following, the most recent published results from simulation studies of top-quark mass measurements are discussed. The studies of top-quark couplings, which make use of the possibilities for polarised beams at linear colliders, are still on-going. Preliminary results indicate a substantially higher precision than achievable at hadron colliders.

#### Top-quark mass measurement at threshold

**Fig. 108:**
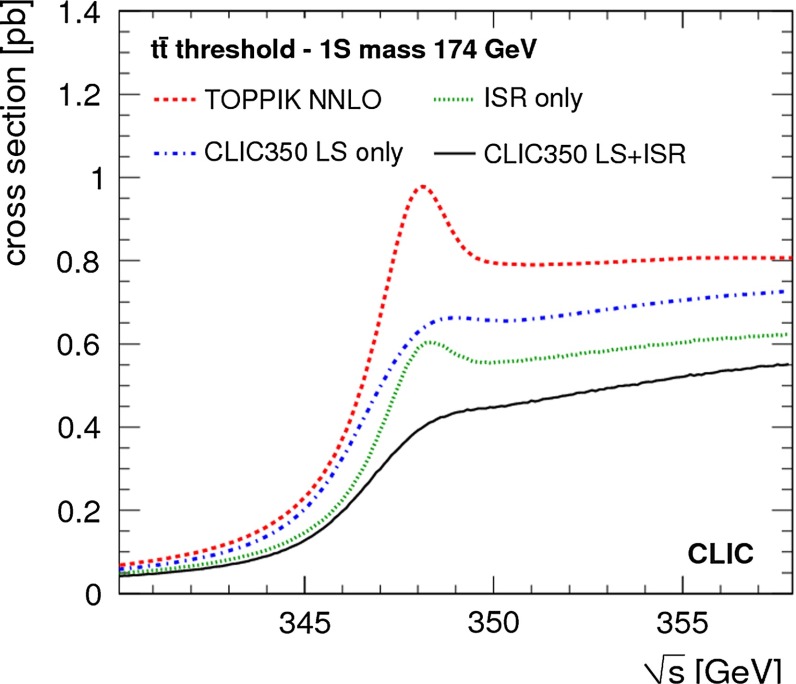
The top-quark production cross section calculated with TOPPIK for a top mass of 174 GeV in the 1S mass scheme, showing the effects of initial-state radiation and of the luminosity spectrum of CLIC. Figure taken from Ref. [40]

**Fig. 109:**
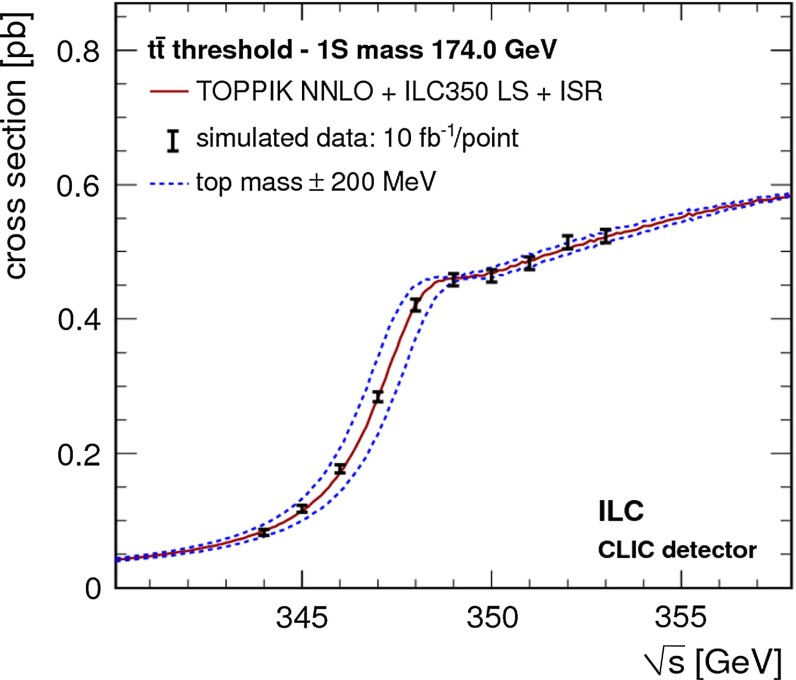
Simulated measurement of the background-subtracted $t\bar{t}$ cross section with 10 fb$^{-1}$ per data point, assuming a top-quark mass of 174 GeV in the 1S scheme with the ILC luminosity spectrum for the CLIC_ILD detector. Figure taken from Ref. [40]

The measurement of $t\bar{t}$ production cross section in a scan around the threshold provides direct access to the top quark, as discussed above. In the experiment, the calculated cross section is modified by initial-state radiation and by the luminosity spectrum of the collider. These two effects are illustrated in Fig. 108 [40], where the pure $t\bar{t}$ cross section is calculated with TOPPIK at NNLO [746, 747] for a top-quark mass of 174 GeV in the 1S mass scheme, and the luminosity spectrum of CLIC at 350 GeV is assumed. Both lead to a smearing of the cross section, resulting in a substantial reduction of the prominence of the cross section peak, and to an overall reduction of the cross section due to the lowering of the luminosity available above the production threshold. Since the beam-energy spread at ILC is smaller than at CLIC, the threshold turn-on is slightly steeper, as visible in Fig. 109.

Recently, an experimental study has been performed in which the NNLO cross section shown in Fig. 108 was used, together with signal efficiencies and background contamination determined with full Geant4 simulations of a CLIC variant of the ILD detector, including the use of the full reconstruction chain. In the context of a threshold scan, where the focus is on the efficient identification of $t\bar{t}$ events, the difference in performance between the ILC and CLIC detector concepts is expected to be negligible, allowing us to apply this study to both accelerator concepts by using the appropriate luminosity spectra. The experimental precision of a threshold scan with a total integrated luminosity of 100 fb$^{-1}$ spread over ten points spaced by 1 GeV for the ILC case is illustrated in Fig. 109.

Since the cross section depends not only on the top-quark mass, but also on $\alpha _s$, those two values are determined simultaneously with a two-dimensional fit, resulting in a statistical uncertainty of 27 MeV on the mass and 0.0008 on $\alpha _s$. Assuming the CLIC luminosity spectrum, which is characterised by a somewhat more pronounced beamstrahlung tail and a larger energy spread, the uncertainties increase to 34 MeV and 0.0009, respectively. Systematic uncertainties from the theoretical cross-section uncertainties, from the precision of the background description and the understanding of the detector efficiency as well as from the absolute knowledge of the beam energy are expected to be of similar order as the statistical uncertainties. Thus, the differences between different linear collider concepts for a top threshold scan are negligible, and total uncertainties of below 100 MeV on the mass are expected [40]. For a phenomenological interpretation, the measured 1S mass typically has to be converted into the standard $\overline{\text{ MS }}$ mass. This incurs additional uncertainties of the order of 100 MeV, depending on the available precision of $\alpha _s$ [747].

As discussed in detail in Ref. [787], in addition to the mass and the strong coupling constant, also the top-quark width can be determined in a threshold scan. The use of additional observables such as the top-quark momentum distribution and the forward–backward asymmetry has the potential to further reduce the statistical uncertainties. The cross section around threshold is also sensitive to the top-quark Yukawa coupling, as discussed above. However, its effect on the threshold behaviour is very similar to that of the strong coupling constant, so an extraction will only be possible with a substantially improved knowledge of $\alpha _s$ compared to the current world average uncertainty of 0.0007, and with reduced theoretical uncertainties on the overall cross section.

#### Top-quark mass measurement in the continuum

In the continuum above the $t\bar{t}$ threshold, the top-quark mass is measured experimentally by directly reconstructing the invariant mass from the measured decay products, a *W* boson and a *b* quark. This is possible with high precision both in fully hadronic (e.g. both *W* bosons produced in the $t\bar{t}$ decay decaying into hadrons) and semileptonic (e.g. one *W* boson decaying into hadrons, one into an electron or muon and a neutrino) top-quark pair decays. Due to the well-defined initial state in $e^+e^-$ collisions, full three-dimensional kinematic constraints can be used for kinematic fitting, substantially improving the invariant mass resolution compared to a free measurement.

**Fig. 110:**
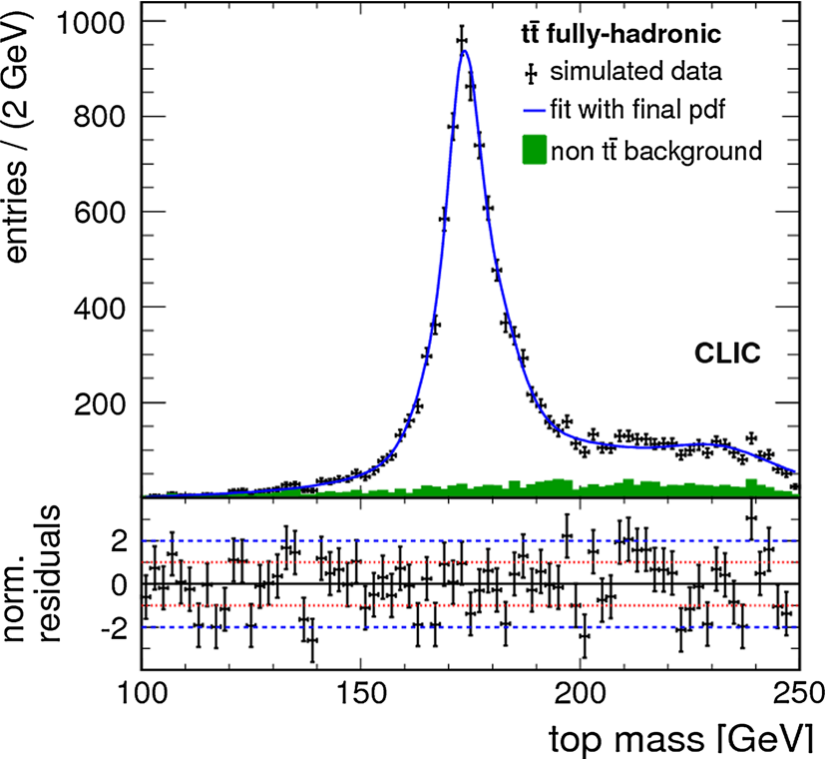
Simulated measurement of the top-quark invariant mass in the all-hadronic decay channel of top-quark pairs for an integrated luminosity of 100 fb$^{-1}$ at CLIC in the CLIC_ILD detector at a centre-of-mass energy of 500 GeV. The *solid green histogram* shows the remaining non $t\bar{t}$ background in the data sample. The mass is determined with an unbinned maximum likelihood fit to the distribution. Figure taken from Ref. [40]

For both CLIC and ILC this measurement has been studied using full detector simulations with all relevant physics backgrounds at an energy of 500 GeV. In the case of the CLIC study, also the influence of background from hadron production in two-photon processes was included, which is more severe at CLIC than at ILC due to the very high bunch-crossing frequency. The reconstructed invariant mass after background rejection and kinematic fitting for the fully hadronic final state at CLIC is shown in Fig. 110. The figure also illustrates the high purity achievable for top quarks at linear colliders. For an integrated luminosity of 100 fb$^{-1}$, combined statistical precisions of 70 and 80 MeV are obtained for ILC [207] and CLIC [40], respectively. The CLIC study showed that it is expected that systematic uncertainties due to the jet energy scale can be limited to below the statistical uncertainty by constraining the light jet-energy scale through the direct reconstruction of the *W* bosons in the top-quark decay. The *b* jet energy scale in turn can be determined in a similar way from $Z\rightarrow b\bar{b}$ decays. Also other experimental systematics, such as the knowledge of the beam energy, which enters in the kinematic fit, and uncertainties from colour reconnection effects are expected to be small.

However, in contrast to the measurement via a threshold scan, the mass determined by direct reconstruction is theoretically not well defined. Rather, it is obtained in the context of the event generator used to determine the detector and reconstruction effects on the measured invariant mass. At present, no conversion of this invariant mass value to the $\overline{\text{ MS }}$ mass exists. This leads to additional uncertainties in the interpretation of the result, which potentially far exceed the experimental accuracy of the invariant mass measurement.

#### Measurement of coupling constants

For precise test of the standard model and New Physics searches a precise determination of the standard model couplings together with the search for anomalous couplings is important. In the following we try to review the prospects of a future Linear Collider and compare where possible with the LHC. From top-quark pair production at hadron collider the top-quark coupling to gluons is already constrained. As mentioned in Sect. 3.4.1 the threshold studies can be used to measure the top-quark mass together with $\alpha _s$. Top-quark pairs produced in association with an additional jet can be used to search directly for anomalous top-gluon couplings. This can be done independent of the production mechanism in hadronic collisions as well as in electron–positron annihilation. For hadronic $t\bar{t} + \text {1-Jet}$ production dedicated NLO calculations are available [788–791]. For electron–positron annihilation the corresponding calculations for massive *b*-quarks [792–796] can be applied by adjusting the coupling constants. A dedicated analysis of top-quark pair $+$ 1-jet production at a future Linear Collider can be found in Ref. [797]. Since anomalous couplings will show up more likely in the couplings to the weak gauge bosons no detailed study of the sensitivity to anomalous top-gluon couplings has been performed so far for a future Linear Collider.

The *Wtb*-coupling can be probed through top-quark decay and single-top-quark production. A detailed measurement of this coupling is interesting because the $V-A$ structure of the vertex can be tested. Furthermore the existence of a fourth family – if not yet ruled out by other measurements – could significantly change the SM predictions for the respective coupling. Tevatron and LHC measurements constrain the coupling already through the measurement of the top-quark width [798] and the measurements of the *W*-boson helicity fractions [799–801]. A measurement of the top-quark width from threshold studies can be used to indirectly constraint the coupling in electron–positron annihilation. A direct measurement of the *Wtb* coupling at a Linear Collider is difficult [779]. In top-quark pair production close to the threshold the coupling enters only through the branching ratio for $t\rightarrow Wb$, which is expected to be very close to one and thus does not lead to a strong dependence on the *Wtb* coupling. Measurements using single-top-quark production are difficult owing to sizeable backgrounds. In Ref. [554] it has been argued that using $e^+e^-\rightarrow W^+bW^-\bar{b}$ events below the $t\bar{t}$ threshold the coupling can be measured at ILC with an accuracy of about 3 % using an integrated luminosity of about 100/fb.

The top-quark coupling to the photon or more precisely the top-quark charge is constrained through indirect measurements at hadron colliders. Using the charge of the top-quark decay products reconstructed from top-candidate events the top-quark charge has been measured in Ref. [802] to be

107 $$\begin{aligned} Q = 0.64 \pm 0.02 (\mathrm{stat.}) \pm 0.08 (\mathrm{syst.}) \end{aligned}$$

in units of the electron charge. A direct measurement of $t\bar{t} +\gamma$ production is difficult at the LHC due to the small cross sections although a measurement with an uncertainty of 10 % might nevertheless be feasible [779]. (First results have been presented already by CDF [803] and ATLAS [804].) At the Linear Collider the analysis of the SM couplings is usually combined with the search for anomalous couplings. As a starting point one may use a form-factor decomposition of the form [779]:

108 $$\begin{aligned} {\varGamma }_\mu ^{ttX}(k^2,q,\bar{q})= & {} i e \bigg \{ \gamma _\mu \left( \tilde{F}^X_{1V}(k^2)+\gamma _5 \tilde{F}^{X}_{1A}\right) \nonumber \\&+\, {(q-\bar{Q})_\mu \over 2M_t}\left( \tilde{F}^X_{2V}(k^2)+\gamma _5 \tilde{F}^{X}_{2A}\right) \bigg \} \end{aligned}$$

where *X* can be a photon as well as a *Z* boson. In Refs. [7, 269, 805] it has been shown that the precision with which the various couplings can be determined can be improved at a Linear Collider by about a factor of 10 compared to what is possible at the LHC. At the LHC the precision for $\tilde{F}_{1V}^\gamma$ and $\tilde{F}_{1A}^\gamma$ is at the level of 10 % [805] and much larger for the remaining couplings.

Given that the top quark is so much heavier than the next heavy quark it seems reasonable to question whether the mechanism to generate the top-quark mass is the same as for the lighter quarks. In this context the measurement of the $t\bar{t}H$ Yukawa coupling is of great importance. At the LHC this coupling can be accessed through the measurement of top-quark pair production in association with a Higgs boson. A recent study of the sensitivity where the subsequent decay $H\rightarrow b\bar{b}$ has been used can be found for example in Ref. [806]. In Ref. [268] it has been estimated that the *ttH* coupling can be measured at the LHC with an accuracy of about 15 % assuming an integrated luminosity of 300/fb at 14 TeV centre-of-mass energy. With an increased luminosity of 3000/fb a measurement at the level of 7–14 % may become feasible. Due to the large mass of the final state it is difficult to improve this measurement significantly at a linear collider operating at 500 GeV. For an integrated luminosity of 1000/fb at 500 GeV centre-of-mass energy an uncertainty of 10 % has been estimated [268]. Increasing the energy to 1 TeV (ILC) or even 1.4 TeV (CLIC) will help to improve the situation: In both cases a precision of 4 % seems to be feasible. Using the ILC design at 1 TeV would require 1000/fb of integrated luminosity, while at 1.4 TeV 1500/fb would be required.

Very recently it has been argued in Ref. [807] that the *ttH* coupling could also be inferred at the LHC from single-top-quark production in association with an additional Higgs. Since the cross section of this process is below 100 fb such a measurement will be challenging. In the standard model the cross section is reduced through an accidental cancellation. As a consequence BSM models may show sizeable deviations compared to the Standard Model prediction.

#### The top-quark polarisation

Top quarks produced in electron–positron annihilation are polarised. Furthermore the spin of the top quark is also correlated with the spin of the antitop quark. As mentioned in Sect. 3.3.1 the top-quark polarisation can be inferred from the angular distributions of the decay products. The top-quark polarisation thus provides an additional observable which allows a more detailed test of the top-quark interactions. The top-quark polarisation and spin correlations in electron–positron annihilation have been studied in detail for example in Refs. [808–816]. In Ref. [817] the impact of the beam polarisation on the polarisation of the produced top quarks has been investigated. In difference from the production rate the observables sensitive to the top-quark polarisation depend only on the effective beam polarisation

109 $$\begin{aligned} P_{eff} = {P_{e^-} -P_{e^+}\over 1-P_{e^-}P_{e^+}} \end{aligned}$$

where $\lambda _- (\lambda _-)$ denotes the longitudinal polarisation of the incoming electrons (positrons). While the top-quark polarisation depends strongly on the $P_{\mathrm{eff}}$ the longitudinal spin correlation depends only weakly on $P_{\mathrm{eff}}$. At a centre-of-mass energy of 500 GeV the polarisation is close to maximal for $|P_{\mathrm{eff}}|=1$. For higher energies the polarisation is reduced. However, for $|P_{\mathrm{eff}}|=1$ a polarisation above 85 % is still possible.

## Exploring the quantum level: precision physics in the SM and BSM^37^

We review the LC capabilities to explore the electroweak (EW) sector of the SM at high precision and the prospects of unveiling signals of BSM physics, either through the presence of new particles in higher-order corrections or via direct production of extra EW gauge bosons. We discuss the experimental and theory uncertainties in the measurement and calculation of EWPO, such as the *W* boson mass, *Z* pole observables, in particular the effective weak mixing angle, $\sin ^2{\theta ^\ell }_{\mathrm {eff}}$, and the anomalous magnetic moment of the muon, $a_\mu$. We concentrate on the MSSM to illustrate the power of these observables for obtaining indirect information on BSM physics. In particular, we discuss the potential of two key EWPOs at a LC, $M_W$ and $\sin ^2{\theta ^\ell }_{\mathrm {eff}}$, to provide a stringent test of the SM and constraints on the MSSM parameter space. Naturally, the recent discovery of a Higgs-like particle at the LHC has a profound impact on EW precision tests of the SM. We present a study of the impact of this discovery on global EW fits, and also include a discussion of the important role of the top-quark mass in performing these high precision tests of the SM. Finally, we review the anticipated accuracies for precision measurements of triple and quartic EW gauge boson couplings, and how deviations from SM gauge boson self interactions relate to different BSM scenarios. These observables are of special interest at a LC, since they have the potential of accessing energy scales far beyond the direct kinematical reach of the LHC or a LC. We conclude with a discussion of the LC reach for a discovery of extra EW gauge bosons, $Z'$ and $W'$, and the LC’s role for pinning down their properties and origin, once discovered.

### The role of precision observables

The SM cannot be the ultimate fundamental theory of particle physics. So far, it succeeded in describing direct experimental data at collider experiments exceptionally well with only a few notable exceptions, e.g., the left–right ($A_\mathrm{LR}^e$(SLD)) and forward–backward ($A_\mathrm{FB}^b$(LEP)) asymmetry (see Sect. 4.3.3), and the muon magnetic moment $g_\mu -2$ (see Sect. 4.6). However, the SM fails to include gravity, it does not provide cold DM, and it has no solution to the hierarchy problem, i.e. it does not have an explanation for a Higgs-boson mass at the electroweak scale. On wider grounds, the SM does not have an explanation for the three generations of fermions or their huge mass hierarchies. In order to overcome (at least some of) the above problems, many new physics models (NPM) have been proposed and studied, such as supersymmetric theories, in particular the MSSM, two-Higgs-doublet models (THDM), technicolour, little Higgs models, or models with (large, warped, or universal) extra spatial dimensions. So far, the SM has withstood all experimental tests at past and present collider experiments, such as the LEP and SLC $e^+ e^-$ colliders, the HERA *ep*, Tevatron $p \bar{p}$, and LHC *pp* collider. Even the recently discovered Higgs-like particle at the LHC, after analysing the 2012 data agrees with the SM Higgs boson expectation, albeit more precise measurements of its properties will be needed to pin down its identity. Measurements of precision observables and direct searches for NPM particles succeeded to exclude or set stringent bounds on a number of these models. The direct search reach is going to be significantly extended in the upcoming years, when the LHC is scheduled to run at or close to its design energy of 14 TeV. Future $e^+e^-$ colliders, such as the ILC or CLIC, have good prospects for surpassing the LHC direct discovery reach, especially in case of weakly interacting, colourless NPM particles (see, e.g., Sect. 4.8).

Even if a direct discovery of new particles is out of reach, precision measurements of SM observables have proven to be a powerful probe of NPM via virtual effects of the additional NPM particles. In general, precision observables (such as particle masses, mixing angles, asymmetries etc.) that can be predicted within a certain model, including higher order corrections in perturbation theory, and thus depending sensitively on the other model parameters, and that can be measured with equally high precision, constitute a test of the model at the quantum-loop level. Various models predict different values of the same observable due to their different particle content and interactions. This permits to distinguish between, e. g., the SM and a NPM, via precision observables. Naturally, this requires a very high precision of both the experimental results and the theoretical predictions. The wealth of high-precision measurements carried out at the *Z* pole at LEP and SLC, the measurement of the *W* boson at LEP and the Tevatron [21, 822, 824], as well as measurements at low-energy experiments, such as $a_\mu =(g_\mu -2)/2$ at the “Muon $g-2$ Experiment” (E821) [818], are examples of EWPOs that probe indirect effects of NPM particles. These are also examples where both experiment and theory have shown that they can deliver the very high precision needed to fully exploit the potential of these EWPOs for detecting minute deviations from the SM. The most relevant EWPOs in which the LC plays a key role are the *W* boson mass, $M_W$, and the effective leptonic weak mixing angle, $\sin ^2{\theta ^\ell }_{\mathrm {eff}}$. In the MSSM, the mass of the lightest ${\mathscr {CP}}$-even MSSM Higgs boson, $M_h$, constitutes another important EWPO [819]. Note that in these examples, the top quark mass plays a crucial role as input parameter.

Also EWPOs that cannot be measured at a LC can be very relevant in the assessment of its physics potential. A prominent role in this respect plays the muon magnetic moment, $(g_\mu -2)$. It already provides some experimental indication for NPM particles in reach of a LC, and its role in constraining NPM and its complementarity to the LC is summarised in Sect. 4.6.

Another type of PO is connected to the self interactions of EW gauge bosons in multiple EW gauge boson production, i.e. they directly probe the triple and quartic EW gauge boson couplings. Deviations from SM predictions would indicate new physics, entering either through loop contributions or are due to new heavy resonances, which at low energy manifest themselves as effective quartic gauge boson couplings. Precision measurements of these POs could provide information as regards NPM sectors far beyond the kinematic reach of the LHC and LC.

As discussed above, in this report we focus our discussion on the EWPO, i.e. (pseudo-) observables like the *W*-boson mass, $M_W$, the effective leptonic weak mixing angle, $\sin ^2{\theta ^\ell }_{\mathrm {eff}}$, and the anomalous magnetic moment of the muon. Since in the literature virtual effects of NPM particles are often discussed in terms of *effective* parameters instead of the EWPO we briefly discuss this approach in the following.

A widely used set of effective parameters are the *S*, *T*, *U* parameters [820]. They are defined such that they describe the effects of new physics contributions that enter only via vacuum-polarisation effects (i.e. self-energy corrections) to the vector boson propagators of the SM (i.e. the new physics contributions are assumed to have negligible couplings to SM fermions). The *S*, *T*, *U* parameters can be computed in different NPMs as certain combinations of one-loop self-energies, and then can be compared to the values determined from a fit to EW precision data, i.e. mainly from $M_W, M_Z$ and ${\varGamma }_Z$ (see, e.g., the review in [821]). A non-zero result for *S*, *T*, *U* indicates non-vanishing contributions of new physics (with respect to the SM reference value). According to their definition, the *S*, *T*, *U* parameters are restricted to leading order contributions of new physics. They should therefore be applied only for the description of *small* deviations from the SM predictions, for which a restriction to the leading order is permissible. Examples of new physics contributions that can be described in the framework of the *S*, *T*, *U* parameters are contributions from a fourth generation of heavy fermions or effects from scalar quark loops to the *W*- and *Z*-boson observables. A counter example, i.e. where the *S*, *T*, *U* framework is not sufficicent, are SUSY corrections to the anomalous magnetic moment of the muon. Due to these restrictions of this *effective* description of BSM effects in *W* and *Z* boson observables, in this report we decided to only present investigations of these effects in the EWPO themselves.

This review of precision physics in the SM and BSM at the LC is organised as follows: in Sect. 4.2 we concentrate on $M_W$ from both the experimental and the theoretical view points, and then turn to a discussion of *Z* pole observables, in particular $\sin ^2{\theta ^\ell }_{\mathrm {eff}}$, in Sect. 4.3. The relevance of the top-quark mass in EW precision physics is briefly summarised in Sect. 4.4, before we present the prospects of extracting information as regards the SM Higgs-boson mass from a global EW fit in Sect. 4.5. We close our discussion of EWPOs with an overview of predictions for the muon magnetic moment in NPM in Sect. 4.6. An overview of possible parametrisations of non-standard EW gauge boson couplings, available calculations and the experimental prospects for precision measurements of these couplings is presented in Sect. 4.7. Finally, in Sect. 4.8 we present an overview of studies of new gauge bosons at the LC.

### The $\varvec{W}$ boson mass

The mass of the *W* boson is a fundamental parameter of the electroweak theory and a crucial input to electroweak precision tests. The present world average for the *W*-boson mass [822],

110 $$\begin{aligned} M_W= 80.385 \pm 0.015\;\mathrm {GeV}\,, \end{aligned}$$

is dominated by the results from the Tevatron, where the *W* boson mass has been measured in Drell–Yan-like single-*W*-boson production. At LEP2, the *W*-boson mass had been measured in *W*-pair production with an error of $33\mathrm {MeV}\,$ from direct reconstruction and $\sim$200 $\mathrm {MeV}\,$ from the cross section at threshold [823, 824]. In this section we will review the prospects for the $M_W$ measurements at the LC from the experimental and theoretical side, as well as the possibility to constrain indirectly parameters of NPM using a precise $M_W$ measurement and prediction.

#### Experimental prospects for a precision measurement of ${M_W}$ a the ILC^38^

The ILC facility^39^  can contribute decisively by making several complementary measurements of the *W* mass using $e^{+}e^{-}$ collisions at centre-of-mass energies spanning from near *WW* threshold to as high as 1 TeV. Data samples consisting of between 10 and 100 million *W* decays can be produced, corresponding to an integrated luminosity of about 250fb$^{-1}$ at $\sqrt{s} = 250$ GeV (and correspondingly lower integrated luminosity at higher energies).

The main production channels of *W* bosons at ILC are pair production, $e^{+} e^{-} \rightarrow W^+ W^-$ and single-*W* production, $e^{+} e^{-} \rightarrow W e \overline{\nu }_e$, which proceeds mainly through $\gamma -W$ fusion. Pair production dominates at lower centre-of-mass energies, while single-*W* production dominates over other $e^+e^-$ sources of hadronic events at the higher energies.

The three most promising approaches to measuring the *W* mass are:

- Polarised threshold scan of the $W^+W^-$ cross section as discussed in [825].
- Kinematically constrained reconstruction of $W^+W^-$ using constraints from four-momentum conservation and optionally mass-equality as was done at LEP2.^40^
- Direct measurement of the hadronic mass. This can be applied particularly to single-*W* events decaying hadronically or to the hadronic system in semileptonic $W^+W^-$ events.

Much of the existing literature on $M_W$ measurement from LEP2 is still very relevant, but one should be aware of a number of LC features which make the LC experimental programme for $M_W$ measurements qualitatively different. Notable advantages are: availability of longitudinally polarised beams, energy and luminosity reach, and much better detectors. Notable concerns are related to potential degradation of the precision knowledge of the initial state.

We first give an outline of statistical considerations for $M_W$ measurements and then outline the strategies considered for being able to make use of this considerable statistical power in experimentally robust ways.

The *statistical* errors on a *W* mass determination at ILC are driven by the cross sections, the intrinsic width of the *W* (${\varGamma }_W \approx 2.08 \mathrm {GeV}\,$), the potential integrated luminosity, the availability of polarised beams, and where appropriate the experimental di-jet mass resolution, event selection efficiencies and backgrounds. The width is the underlying fundamental issue. This broadens the turn-on of the *W*-pair cross section near threshold, decreasing its dependence on $M_W$. It also broadens the *W* line-shape, diluting the statistical power of mass measurements for both kinematically constrained reconstruction and direct mass reconstruction. For the detectors envisaged at ILC, hadronically decaying *W*s should be measured with mass resolutions in the 1–2 $\mathrm {GeV}\,$ range.

**Fig. 111:**
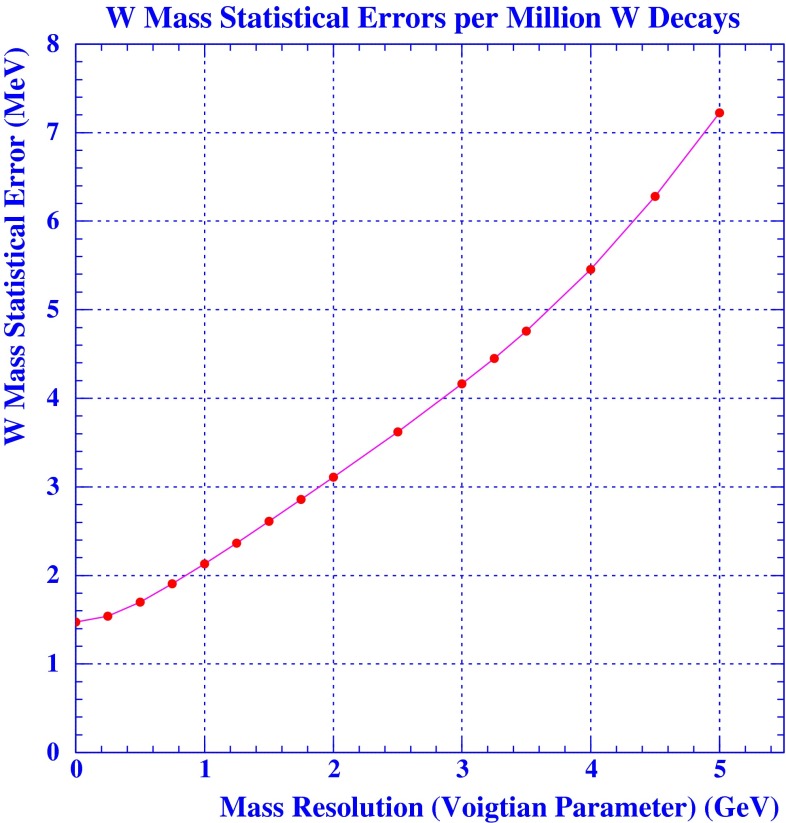
Statistical precision on $M_W$ from the Voigtian fit (see text)

We have estimated the statistical sensitivity dependence on experimental mass resolution quantitatively using a fit to the simulated measured line-shape for one million *W* decays, while varying the assumed experimental mass resolution (per decay). Results of a fit with a (non-relativistic) Breit–Wigner convolved with a Gaussian of known width (Voigtian fit) are shown in Fig. 111. One sees from this that statistical sensitivities of around 2.5 $\mathrm {MeV}\,$ per million *W* decays are to be expected for mass resolutions in the 1–2 $\mathrm {GeV}\,$ range. In practice experiments will use a variety of analysis techniques such as convolution fits where one takes into account the mass resolution on an event-by-event basis maximising the statistical power of well-measured events and de-weighting events with worse resolution. With a data-sample with several tens of millions of *W* decays, the end result will be statistical sensitivity on $M_W$ below 1 $\mathrm {MeV}\,$ and potentially in the 0.5 $\mathrm {MeV}\,$ range.

Statistical errors from a single cross-section measurement near threshold ($\sqrt{s} \approx 2 M_W+ 0.5\;\mathrm {GeV}\,$) are discussed in [826]. The statistical sensitivity factor on $M_W$ for an optimised single cross-section measurement assuming unpolarised beams, 100 % efficiency and no backgrounds is $0.91\,\mathrm {MeV}\,/\sqrt{{\mathscr {L}}_\mathrm{int} [\mathrm {ab}^{-1}]}$. For an integrated luminosity of ${\mathscr {L}}_\mathrm{int} = 100~\mathrm {fb}^{-1}$ this translates to 2.9 $\mathrm {MeV}\,$. However experimental systematic errors on such a single cross-section measurement of $\sim$0.25 % enter directly and would give a corresponding 4.2 $\mathrm {MeV}\,$ experimental systematic uncertainty. At the ILC, the statistical sensitivity factor can be further improved using polarised beams colliding with the appropriate helicities corresponding effectively for practical polarisation values (80–90, 40–60%) to a factor of up to 3 *WW*-production luminosity upgrade.

The method of a polarised threshold scan is discussed in some detail in [825] based on conservative extrapolations from the measurements using the LEP detectors. The idea is to use the measurement of the threshold dependence of the cross section to determine $M_W$. The study is based on 100 fb$^{-1}$ allocated to 5 scan points near threshold and 1 scan point at 170 $\mathrm {GeV}\,$. Data are collected mostly with $e^{-}_{L} e^{+}_{R}$ but other combinations of two-beam, single-beam and no beam polarisation are used to control the backgrounds and polarisation systematics. The $170\,\mathrm {GeV}\,$ point has little sensitivity to $M_W$ but helps to constrain the efficiency systematics. The overall experimental error on the *W* mass (excluding beam-energy systematic and eventual theoretical errors) is estimated to be $5.2~\mathrm {MeV}\,$.

A critical external input needed to interpret the threshold dependence of the cross section in terms of $M_W$ is knowledge of the centre-of-mass energies. Various measurements sensitive to the centre-of-mass energy can be made using $e^+e^- \rightarrow \ell \ell \gamma$ ($\ell = e, \mu$) events. From knowledge of the polar angles of the leptons, under the assumption of a 3-body final state, one can measure statistically the luminosity-weighted centre-of-mass energy with an error of 31 ppm for the proposed scan. This translates into a $M_W$ error of $2.5\,\mathrm {MeV}\,$ per 100 fb$^{-1}$ polarised scan. A related method using the momenta of the two leptons (particularly the muons) can determine the centre-of-mass energy with much better statistical precision. The tracker momentum scale needs to be controlled – this is feasible using *Z*’s – and potentially with other particles with well-measured masses.

In summary, it is estimated that $M_W$ can be measured to $6\,\mathrm {MeV}\,$ experimental accuracy using this method which uses dedicated running near threshold. This number includes also the anticipated uncertainties from the beam energy ($\sim$1.9 $\mathrm {MeV}\,$) and from theory ($\sim$2.5 $\mathrm {MeV}\,$), where the corresponding theoretical issues will be discussed in the next subsection.

Much of the ILC programme is likely to take place at energies significantly above the *WW* threshold in a regime where both *WW* production and single-*W* production are prevalent. Consequently, a direct reconstruction of the hadronic mass can be very important. One can use *WW* events with one *W* decaying leptonically ($e, \mu , \tau$) and the other decaying hadronically, and also single-*W* events with the *W* decaying hadronically to measure $M_W$ from the measured hadronic mass. Beam polarisation can be used to enhance the cross sections. The critical issue is being able to control the jet energy scale. A number of approaches are plausible and should be pursued. One approach consists of using *Z*($\gamma$) radiative return events where the *Z* decays hadronically and the photon is unmeasured within or close to the beam-pipe. Another approach attempts to do a jet-energy calibration from first principles using the individual components that make up the measured jet energy, namely using the calibration of the tracker momentum scale and the calorimeter energy scales at the individual particle level determined from for example calibration samples of well-known particles ($J/\psi$, $K^0_S$, ${\Lambda }$, $\pi ^0$ etc.). The latter has the advantage that it does not rely directly on the *Z* mass. Other calibration possibilities are using *ZZ*, *Zee* and Z$\nu \nu$ events. Assuming a sample of $5\,10^6$ hadronic *Z*s for calibration one should be able to approach a jet-energy scale related statistical error of around $2.0\,\mathrm {MeV}\,$ for $M_W$. Systematic limitations in the *Z*-based methods is the knowledge of the *Z* mass (currently $2.1\,\mathrm {MeV}\,$) – and any residual quark-flavour related systematics that make the detector response of hadronic *W*s different from hadronic *Z*s. It seems plausible to strive for an overall error of $5\,\mathrm {MeV}\,$ from these methods.

A kinematically constrained reconstruction of *WW* pairs was the work-horse of LEP2 – but has received little attention to date for ILC studies related to *W* mass measurement. By imposing kinematic constraints, the LEP2 experiments were able to compensate for modest jet-energy resolution. At ILC, the constraints are no longer as valid (beamstrahlung) the detector resolution is much better (of the same order as ${\varGamma }_W$), and until recently, it seemed that the beam energy could not be determined with adequate precision at high energy. Lastly, at the order of precision that is being targeted, it seems unwise to bank on the fully hadronic channel where it is quite possible that final-state interactions such as colour reconnection may cause the mass information to be corrupted. So it seems that the kinematically constrained reconstruction method is most pertinent to the $q \bar{q} e \nu _e$ and $q \bar{q} \mu \nu _\mu$ channels.

Recent work exploring the reconstruction of the centre-of-mass energy using the measured muon momenta in $e^+e^- \rightarrow \mu ^+ \mu ^- (\gamma )$ events indicates that it is very feasible to measure the luminosity-weighted centre-of-mass energy with high precision, and that this approach is promising also at relatively high centre-of-mass energies.

In addition, given the impetus for potentially running the ILC at a centre-of-mass energy of around $250\,\mathrm {GeV}\,$, not far above LEP2, there seems a clear potential to improve the $M_W$ measurement by including information from the leptons in the mass estimate. This lower energy regime should be the most favourable for beamstrahlung and beam-energy determination outlook. Probably by performing kinematically constrained fits that build on the existing methods one would be able to get complementary information, which would be significantly uncorrelated in several of the main systematics with the direct reconstruction method. This deserves more study – but errors at the $5\,\mathrm {MeV}\,$ level or less may be achievable.

To summarise, the ILC facility has three principal ways of measuring $M_W$. Each method can plausibly measure $M_W$ to a precision in the $5\,\mathrm {MeV}\,$ range. The three methods are largely uncorrelated. If all three methods do live up to their promise, one can target an overall uncertainty on $M_W$ in the 3–4 $\mathrm {MeV}\,$ range.

#### Theory aspects concerning the *WW* threshold scan^41^

While in the previous subsection the experimental precision for the *W* boson mass measurement at the LC was discussed, this subsection deals with the correspondingly required theory calculations and precisions, in particular for the *WW* threshold scan.

The theoretical uncertainty (TU) for the direct mass reconstruction at LEP2 has been estimated to be of the order of $\sim$5–10 $\mathrm {MeV}\,$ [827, 828], based on results of YFSWW [829] and RacoonWW [830], which used the double-pole approximation (DPA) for the calculation of the NLO corrections. This is barely sufficient for the accuracies aimed at a LC. These shortcomings of the theoretical predictions have been cured by dedicated calculations.

In [831, 832] the total cross section for the charged-current four-fermion production processes $$e^+e^-\rightarrow \nu _\tau \tau ^+\mu ^-\bar{\nu }_\mu $$, $$u\bar{d}\mu ^-\bar{\nu }_\mu $$, $${u}\bar{d}s\bar{c}$$ was presented including the complete electroweak NLO corrections and all finite-width effects. This calculation was made possible by using the complex-mass scheme for the description of the *W*-boson resonances and by novel techniques for the evaluation of the tensor integrals appearing in the calculation of the one-loop diagrams. The full $${\mathscr {O}}(\alpha )$$ calculation, improved by higher-order effects from ISR, reduced the remaining TU due to unknown electroweak higher-order effects to a few 0.1 % for scattering energies from the threshold region up to $$\sim $$500 $$\mathrm {GeV}\,$$; above this energy leading high-energy logarithms, such as Sudakov logarithms, beyond one loop have to be taken into account to match this accuracy [833]. At this level of accuracy, also improvements in the treatment of QCD corrections to semileptonic and hadronic $$e^+e^-\rightarrow 4f$$ processes are necessary. The corrections beyond DPA, were assessed by comparing predictions in DPA from the generator RacoonWW to results from the full four-fermion calculation [831, 832], as coded in the follow-up program Racoon4f (which is not yet public). This comparison revealed effects on the total cross section without cuts of $$\sim $$$$0.3\,\% (0.6\,\%)$$ for CM energies ranging from $$\sqrt{s}\sim 200\,\mathrm {GeV}\,$$ ($$170\,\mathrm {GeV}\,$$) to $$500\,\mathrm {GeV}\,$$. The difference to the DPA increases to 0.7–1.6 % for $$\sqrt{s}\sim 1{-}2\,{\text {TeV}}$$. At threshold, the full $${\mathscr {O}}(\alpha )$$ calculation corrects the IBA by about 2 %. While the NLO corrections beyond DPA have been calculated only for the processes $$e^+e^-\rightarrow \nu _\tau \tau ^+\mu ^-\bar{\nu }_\mu $$, $$u\bar{d}\mu ^-\bar{\nu }_\mu $$, $${u}\bar{d}s\bar{c}$$ so far, the effect for the other four-fermion processes, which interfere with *ZZ* production, should be similar. Once the corrections to those channels are needed, they can be calculated with the available methods.

Using methods from effective field theory, the total cross section for 4-fermion production was calculated near the *W* pair production threshold [771, 772]. These calculations used unstable-particle effective field theory to perform an expansion in the coupling constants, ${\varGamma }_W/M_W$, and in the non-relativistic velocity *v* of the *W* boson up to NLO in ${\varGamma }_W/M_W\sim \alpha _\mathrm{ew}\sim v^2$. In [771] the theoretical error of an $M_W$ determination from the threshold scan has been analysed. As a result, the resummation of next-to-leading collinear logarithms from initial-state radiation is mandatory to reduce the error on the *W* mass from the threshold scan below $30\,\mathrm {MeV}\,$. It was found that the remaining uncertainty of the pure NLO EFT calculation is $\delta M_W\approx 10{-}15\,\mathrm {MeV}\,$ and is reduced to about $5\,\mathrm {MeV}\,$ with additional input from the NLO four-fermion calculation in the full theory. In order to reduce this error further, in [772] the (parametrically) dominant next-to-next-to-leading order (NNLO) corrections (all associated with the electromagentic Coulomb attraction of the intermediate *W* bosons) in the EFT have been calculated leading to a shift of $\delta M_W\sim 3\,\mathrm {GeV}\,$ and to corrections to the cross section at the level of 0.3 %. The effect of typical angular cuts on these corrections was shown to be completely negligible. Thus, one may conclude that the inclusive partonic four-fermion cross section near the *W*-pair production threshold is known with sufficient precision.

In summary, all building blocks for a sufficiently precise prediction of the *W*-pair production cross section in the threshold region are available. They require the combination of the NLO calculation of the full four-fermion cross section with the (parametrically) dominant NNLO corrections, which are calculated within the EFT. For the precise determination of the cross section at energies above $500\,\mathrm {GeV}\,$ the leading two-loop (Sudakov) corrections should be included in addition to the full NLO corrections. Combining the theoretical uncertainties with the anticipated precision from a threshold scan (see the previous subsection) a total uncertainty of $7\,\mathrm {MeV}\,$ can be estimated [834].

#### Theory predictions for $M_W$ in the SM and MSSM^42^

The precise measurement of the *W* boson mass can be used to test NPM via their contribution to quantum corrections to $M_W$. However, this requires a precise prediction of $M_W$ in the respective models. Here we will concentrate on the prediction of $M_W$ in the MSSM.

The prediction of $M_W$ in the MSSM depends on the masses, mixing angles and couplings of all MSSM particles. Sfermions, charginos, neutralinos and the MSSM Higgs bosons enter already at one-loop level and can give substantial contributions to $M_W$. Consequently, it is expected to obtain restrictions on the MSSM parameter space in the comparison of the $M_W$ prediction and the experimental value of Eq. (110).

The results for the general MSSM can be obtained in an extensive parameter scan [835]. The ranges of the various SUSY parameters are given in Table 26. $\mu$ is the Higgsino mixing parameter, $M_{\tilde{F}_i}$ denotes the soft SUSY-breaking parameter for sfermions of the *i*th family for left-handed squarks ($F = Q$), right-handed up- and down-type squarks ($F = U, D$), left-handed sleptons ($F = L$) and right-handed sleptons ($F = E$). $A_f$ denotes the tri-linear sfermion–Higgs couplings, $M_3$ the gluino mass parameter and $M_2$ the *SU*(2) gaugino mass parameter, where the *U*(1) parameter is fixed as $M_1 = 5/3 s_W^2/c_W^2 M_2$. $M_A$ is the ${\mathscr {CP}}$-odd Higgs boson mass and $\tan \beta$ the ratio of the two Higgs vacuum expectation values.

**Table 26:** Parameter ranges. All parameters with mass dimension are given in GeV

Parameter	Minimum	Maximum
$\mu$	$-$2000	2000
$M_{\tilde{E}_{1,2,3}}=M_{\tilde{L}_{1,2,3}}$	100	2000
$M_{\tilde{Q}_{1,2}}=M_{\tilde{U}_{1,2}}=M_{\tilde{D}_{1,2}}$	500	2000
$M_{\tilde{Q}_{3}}$	100	2000
$M_{\tilde{U}_{3}}$	100	2000
$M_{\tilde{D}_{3}}$	100	2000
$A_e=A_{\mu }=A_{\tau }$	$-$3$\,M_{\tilde{E}}$	3$\,M_{\tilde{E}}$
$A_{u}=A_{d}=A_{c}=A_{s}$	$-$3$\,M_{\tilde{Q}_{12}}$	3$\,M_{\tilde{Q}_{12}}$
$A_b$	$-$3 max($M_{\tilde{Q}_{3}},M_{\tilde{D}_{3}}$)	3 max($M_{\tilde{Q}_{3}},M_{\tilde{D}_{3}}$)
$A_t$	$-$3 max($M_{\tilde{Q}_{3}},M_{\tilde{U}_{3}}$)	3 max($M_{\tilde{Q}_{3}},M_{\tilde{U}_{3}}$)
$\tan \beta$	1	60
$M_3$	500	2000
$M_A$	90	1000
$M_2$	100	1000

All MSSM points included in the results have the neutralino as LSP and the sparticle masses pass the lower mass limits from direct searches at LEP. The Higgs and SUSY masses are calculated using FeynHiggs (version 2.9.4) [226, 836–839]. For every point it was tested whether it is allowed by direct Higgs searches using the code HiggsBounds (version 4.0.0) [250, 251]. This code tests the MSSM points against the limits from LEP, Tevatron and the LHC.^43^

**Fig. 112:**
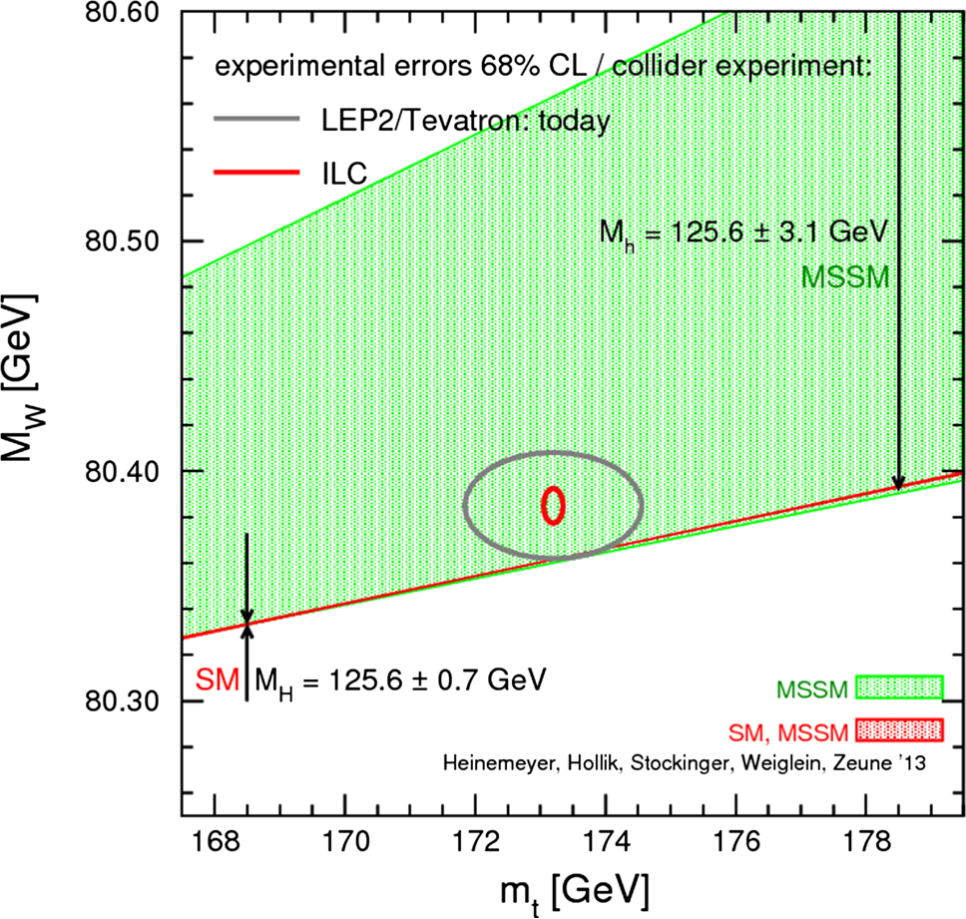
Prediction for $M_W$ as a function of $m_{t}\,$. The plot shows the $M_W$ prediction assuming the light ${\mathscr {CP}}$-even Higgs *h* in the region $125.6 \pm 3.1\,\mathrm {GeV}\,$. The *red band* indicates the overlap region of the SM and the MSSM with $M_H^\mathrm{SM}=125.6\pm 3.1$ GeV. All points are allowed by HiggsBounds. The *grey ellipse* indicates the current experimental uncertainty, whereas the *red ellipse* shows the anticipated future ILC/GigaZ precision

The evaluation of $M_W$ includes the full one-loop result and all known higher order corrections of SM- and SUSY-type, for details see [835, 840] and references therein. The results for $M_W$ are shown in Fig. 112 as a function of $m_{t}\,$. In the plot the green region indicated the MSSM $M_W$ prediction assuming the light ${\mathscr {CP}}$-even Higgs *h* in the region $125.6 \pm 3.1\,\mathrm {GeV}\,$. The red band indicates the overlap region of the SM and the MSSM. The leading one-loop SUSY contributions arise from the stop sbottom doublet. However, requiring $M_h$ in the region $125.6 \pm 3.1\,\mathrm {GeV}\,$ restricts the parameters in the stop sector [248] and with it the possible $M_W$ contribution. Large $M_W$ contributions from the other MSSM sectors are possible, if either charginos, neutralinos or sleptons are light.

The grey ellipse indicates the current experimental uncertainty, see Eqs. (110), (120), whereas the red ellipse shows the anticipated future ILC/GigaZ precision. While at the current level of precision SUSY might be considered as slightly favoured over the SM by the $M_W$–$m_{t}\,$ measurement, no clear conclusion can be drawn. The small red ellipse, on the other hand, indicates the discrimination power of the future ILC/GigaZ measurements. With the improved precision a small part of the MSSM parameter space could be singled out. The comparison of the SM and MSSM predictions with the ILC/GigaZ precision could rule out either of the models.

### $\varvec{Z}$ pole observables

Other important EWPOs are the various observables related to the *Z* boson, measured in four-fermion processes, $e^+ e^- \rightarrow \gamma ,Z \rightarrow f \bar{f}$, at the *Z* boson pole. We review the theoretical precision of SM predictions for various *Z* boson pole observables and the anticipated experimental precision at GigaZ. As for $M_W$, we also review the potential of a precise measurement and prediction of $\sin ^2{\theta ^\ell }_{\mathrm {eff}}$ to obtain information as regards the MSSM parameter space.

#### Theoretical prospects^44^

Near the *Z*-peak the differential cross section for $e^+e^- \rightarrow f\bar{f}$ can be written as^45^

111 $$\begin{aligned}&\frac{d\sigma }{d\cos \theta } = {\mathscr {R}}_\mathrm{ini} \biggl [ \frac{9}{2}\pi \nonumber \\&\qquad \times \frac{{\varGamma }_{ee}{\varGamma }_{ff}(1-{\mathscr {P}}_e{\mathscr {A}}_e) (1+\cos ^2\theta ) + 2({\mathscr {A}}_e-{\mathscr {P}}_e){\mathscr {A}}_f\cos \theta }{(s-M_Z^2)^2-M_Z^2{\varGamma }_Z^2}\\&\qquad +\, \sigma _{\text {non-res}} \biggr ], \end{aligned}$$

where

112 $$\begin{aligned}&{\varGamma }_{ff} = {\mathscr {R}}^f_V g_{Vf}^2 + {\mathscr {R}}^f_A g_{Af}^2, \quad {\varGamma }_Z = \sum \nolimits _f {\varGamma }_{ff},\end{aligned}$$

113 $$\begin{aligned}&{\mathscr {A}}_f = 2\frac{g_{Vf}/g_{Af}}{1+(g_{Vf}/g_{Af})^2} = \frac{1-4|Q_f|\sin ^2\theta ^f_\mathrm{eff}}{1-4\sin ^2\theta ^f_\mathrm{eff} + 8(\sin ^2\theta ^f_\mathrm{eff})^2}. \end{aligned}$$

Here ${\varGamma }_Z$ is the total *Z* decay width, ${\varGamma }_{ff}$ is the partial width for the decay $Z\rightarrow f\bar{f}$, and $g_{Vf}/g_{Af}$ are the effective vector/axial-vector couplings that mediate this decay. These effective couplings include higher-order loop corrections to the vertex, except for QED and QCD corrections to the external $f\bar{f}$ system, which are captured by the radiator functions ${\mathscr {R}}^f_V$ and ${\mathscr {R}}^f_A$. The factor $\mathscr {R}_\mathrm{ini}$, on the other hand, accounts for QED radiation in the initial state. (Specifically, as written in Eq. (111), it describes these effects *relative* to the final-state radiation contribution for $e^+e^-$.)

Equation (111) explicitly spells out the leading *Z*-pole contribution, while additional effects from photon exchange and box corrections are included in the remainder $\sigma _{\text {non-res}}$.

The ratio of $g_{Vf}$ and $g_{Af}$ is commonly parametrised through the effective weak mixing angle $\sin ^2\theta ^f_\mathrm{eff}$. It can be determined from the angular distribution with respect to $\cos \theta$ or from the dependence on the initial electron polarisation ${\mathscr {P}}_e$. On the other hand, the partial and total widths are determined from the total cross section $\sigma (s)$ for different values of *s* and from branching ratios (see the previous subsection).

For leptonic final states, the effective weak mixing angle $\sin ^2\theta ^\ell _\mathrm{eff}$ has been calculated in the SM to the complete two-loop order [842–849], and three- and four-loop corrections of order ${\mathscr {O}}(\alpha \alpha _s^2)$ [850–853] and ${\mathscr {O}}(\alpha \alpha _{s}^3)$ [854–856] are also known. Furthermore, the leading ${\mathscr {O}}(\alpha ^3)$ and ${\mathscr {O}}(\alpha ^2\alpha _s)$ contributions for large values of $m_t$ [857, 858] or $m_H$ [859, 860] have been computed.

The current uncertainty from unknown higher orders is estimated to amount to about $4.5\times 10^{-5}$ [849], which mainly stems from missing ${\mathscr {O}}(\alpha ^2\alpha _s)$ and ${\mathscr {O}}(N_f^2\alpha ^3,\,N_f^3\alpha ^3)$ contributions beyond the leading $m_t^4$ and $m_t^6$ terms, respectively. (Here $N_f^n$ denotes diagrams with *n* closed fermion loops. Based on experience from lower orders, the ${\mathscr {O}}(\alpha ^3)$ diagrams with several closed fermion loops are expected to be dominant.) The calculation of these corrections requires three-loop vertex integrals with self-energy sub-loops and general three-loop self-energy integrals, which realisitically can be expected to be worked out in the forseeable future. The remaining ${\mathscr {O}}(\alpha ^3)$ and four-loop terms should amount to $\sim 10^{-5}$.^46^

For quark final states, most two-loop corrections to $\sin ^2\theta ^q_\mathrm{eff}$ have been computed [849, 861–863], but only the ${\mathscr {O}}(N_f\alpha ^2)$ and ${\mathscr {O}}(N_f^2\alpha ^2)$ contributions are known for the electroweak two-loop corrections, while the diagrams without closed fermion loops are still missing. However, based on experience from the leptonic weak mixing angle, they are expected to amount to $\lesssim$10$^{-5}$. However, the ${\mathscr {O}}(\alpha \alpha _s^2)$ also not known in this case, leading to an additional theory error of $\sim 2\times 10^{-5}$. The calculation of the missing ${\mathscr {O}}(\alpha \alpha _s^2)$ corrections, as well as the ${\mathscr {O}}(\alpha ^2\alpha _s)$ corrections, involves general three-loop vertex corrections to $Z \rightarrow q\bar{q}$, which will only be possible with serious progress in calculational techniques.

When extracting $\sin ^2\theta ^\ell _\mathrm{eff}$ from realistic observables [left–right (LR) and forward–backward (FB) asymmetries, see the next subsection], the initial- and final-state QED radiator functions ${\mathscr {R}}_i$ must be taken into account. In general, the QED corrections are known to ${\mathscr {O}}(\alpha )$ for the differential cross section and to ${\mathscr {O}}(\alpha ^2)$ for the integrated cross section (see Ref. [864] for a summary). However, for the LR asymmetry they complete cancel up to NNLO [865, 866], while for the FB asymmetry they cancel if hard-photon contributions are excluded, i.e. they cancel up to terms of order $E_\gamma /\sqrt{s}$ [865–869]. Therefore, a sufficiently precise result for the soft-photon contribution with $E_\gamma < E_\gamma ^\mathrm{cut}$ can be obtained using existing calcations for small enough $E_\gamma ^\mathrm{cut}$, while the hard-photon contribution ($E_\gamma > E_\gamma ^\mathrm{cut}$) can be evaluated with numerical Monte-Carlo methods. A similar procedure can be carried out for final-state QCD effects for $\sin ^2\theta ^q_\mathrm{eff}$ although the corrections beyond NLO are not fully implemented in existing programs (see below).

For the branching fraction $R_b = {\varGamma }_b/{\varGamma }_\mathrm{had}$ and the total width ${\varGamma }_Z$, two-loop corrections of ${\mathscr {O}}(\alpha \alpha _s)$, ${\mathscr {O}}(N_f\alpha ^2)$, and ${\mathscr {O}}(N_f^2\alpha ^2)$ are known [862, 863, 870–872]. Assuming geometric progression of the perturbative series, the remaining higher-order contributions are estimated to contribute at the level of $\sim 1.5\times 10^{-4}$ and 0.5 MeV, respectively. As before, the contribution from electroweak two-loop diagrams without closed fermion loops is expected to be small. The dominant missing contributions are the same as for $\sin ^2\theta ^q_\mathrm{eff}$.

The current status of the theoretical calculations and prospects for the near future are summarised in Table 27. Note that $\sigma _{\text {non-res}}$ is suppressed by ${\varGamma }_Z/M_Z$ compared to the leading pole term, so that the known one-loop corrections are sufficient to reach NNLO precision at the *Z* pole.

**Table 27:** Some of the most important precision observables for *Z*-boson production and decay (first column), their present-day estimated theory error (second column), the dominant missing higher-order corrections (third column), and the estimated improvement when these corrections are available (fourth column). In many cases, the leading parts in a large-mass expansion are already known, in which case the third column refers to the remaining pieces at the given order. The numbers in the last column are rough order-of-magnitude guesses. Entries in *[italics]* indicate contributions that probably will require very significant improvements in calculational techniques to be completed

Quantity	Cur. theo. error	Lead. missing terms	Est. improvem.
$\sin ^2\theta ^\ell _\mathrm{eff}$	$4.5\times 10^{-5}$	${\mathscr {O}}(\alpha ^2\alpha _s)$, ${\mathscr {O}}(N_f^{\ge 2}\alpha ^3)$	Factor 3–5
$\sin ^2\theta ^q_\mathrm{eff}$	$5\times 10^{-5}$	${\mathscr {O}}(\alpha ^2)$, ${\mathscr {O}}(N_f^{\ge 2}\alpha ^3)$	Factor 1–1.5
		[${\mathscr {O}}(\alpha \alpha _s^2)$, ${\mathscr {O}}(\alpha ^2\alpha _s)$]	[*Factor 3–5*]
$R_b$	$\sim$ $1.5\times 10^{-4}$	${\mathscr {O}}(\alpha ^2)$, ${\mathscr {O}}(N_f^{\ge 2}\alpha ^3)$	Factor 1–2
		[${\mathscr {O}}(\alpha \alpha _s^2)$, ${\mathscr {O}}(\alpha ^2\alpha _s)$]	[*Factor 3–5*]
${\varGamma }_Z$	$0.5\,\text {MeV}$	${\mathscr {O}}(\alpha ^2)$, ${\mathscr {O}}(N_f^{\ge 2}\alpha ^3)$	Factor 1–2
		[${\mathscr {O}}(\alpha \alpha _s^2)$, ${\mathscr {O}}(\alpha ^2\alpha _s)$]	[*Factor 3–5*]

The known corrections to the effective weak mixing angles and the leading corrections to the partial widths are implemented in programs such as Zfitter [864, 873] and Gfitter [874] (see also Sect. 4.5), while the incorporation of the recent full fermionic two-loop corretions is in progress. However, these programs are based on a framework designed for NLO but not NNLO corrections. In particular, there are mismatches between the electroweak NNLO corrections to the $Zf\bar{f}$ vertices and QED/QCD corrections to the external legs due to approximations and factorisation assumptions. Another problem is the separation of leading and sub-leading pole terms in Eq. (111) [849]. While these discrepancies may be numerically small, it would be desirable to construct a new framework that treats the radiative corrections to *Z*-pole physics systematically and consistently at the NNLO level and beyond. Such a framework can be established based on the pole scheme [875, 876], where the amplitude is expanded about the complex pole $s=M_Z^2-i M_Z{\varGamma }_Z$, with the power counting ${\varGamma }_Z/M_Z\sim \alpha$.

#### Experimental prospects^47^

The effective weak mixing angle $\sin ^2\theta _{\mathrm {eff}}^{\ell }$ can be measured at a linear collider running at the *Z*-mass using the left–right asymmetry [877]. With at least the electron beam polarised with a polarisation of ${\mathscr {P}}$, $\sin ^2\theta _{\mathrm {eff}}^{\ell }$ can be obtained via

114 $$\begin{aligned}&A_{\mathrm {LR}}= \frac{1}{{\mathscr {P}}}\frac{\sigma _L-\sigma _R}{\sigma _L+\sigma _R} = {\mathscr {A}}_{e}= \frac{2 g_{V_e}g_{A_e}}{g_{V_e}^2 +g_{A_e}^2}\nonumber \\&{g_{V_e}}/{g_{A_e}} = 1 - 4 \sin ^2\theta _{\mathrm {eff}}^{\ell }\end{aligned}$$

independent of the final state. With $10^9$*Z*s, an electron polarisation of 80 % and no positron polarisation the statistical error is ${\varDelta } A_{\mathrm {LR}}= 4 \cdot 10^{-5}$. The error from the polarisation measurement is ${\varDelta } A_{\mathrm {LR}}/A_{\mathrm {LR}}= {\varDelta } {\mathscr {P}}/{\mathscr {P}}$. With electron polarisation only and ${\varDelta } {\mathscr {P}}/{\mathscr {P}} = 0.5\,\%$ one has ${\varDelta } A_{\mathrm {LR}}= 8 \cdot 10^{-4}$, much larger than the statistical precision. If also positron polarisation is available ${\mathscr {P}}$ in Eq. (114) has to be replaced by ${\mathscr {P}}_\mathrm{{eff}}= \frac{{\mathscr {P}}_{e^+}+{\mathscr {P}}_{e^-}}{1+{\mathscr {P}}_{e^+}{\mathscr {P}}_{e^-}}$. For ${\mathscr {P}}_{e^-}({\mathscr {P}}_{e^+}) = 80\,\%(60\,\%)$, due to error propagation, the error in ${\mathscr {P}}_\mathrm{{eff}}$ is a factor of 3 to four smaller than the error on ${\mathscr {P}}_{e^+},\, {\mathscr {P}}_{e^-}$ depending on the correlation between the two measurements. If one takes, however, data on all four polarisation combinations the left–right asymmetry can be extracted without absolute polarimetry [878] and basically without increasing the error if the positron polarisation is larger than 50 %. Polarimetry, however, is still needed for relative measurements like the difference of absolute values of the positive and the negative helicity states. Assuming conservatively ${\varDelta } A_{\mathrm {LR}}= 10^{-4}$ leads to ${\varDelta } \sin ^2\theta _{\mathrm {eff}}^{\ell }= 0.000013$, more than a factor 10 better than the LEP/SLD result.

The largest possible uncertainty comes from the knowledge of the beam energy. $\sqrt{s}$ must be known with $1\,\mathrm{MeV}$ relative to the *Z*-mass. The absolute precision can be calibrated in a *Z*-scan, however, a spectrometer with a relative precision of $10^{-5}$ is needed not to be dominated by this uncertainty. Similarly the beamstrahlung must be known to a few per-cent relative between the calibration scans and the pole running. However, both requirements seem to be possible.

Apart from $\sin ^2\theta _{\mathrm {eff}}^{\ell }$ also some other *Z*-pole observables can be measured at a LC. Running at the *Z* peak gives access to the polarised forward–backward asymmetry for *b*-quarks which measures $\sin ^2\theta _{\mathrm {eff}}^{b}$ and the ratio of the *b* to the hadronic partial width of the *Z*-boson $R_{b}^0={\varGamma }_{b \overline{b}}/{\varGamma }_{\mathrm{had}}$. Both quantities profit from the large statistics and the much improved *b*-tagging capabilities of an ILC detector compared to LEP.

$R_{b}^0$ can be measured using the same methods as at LEP. The statistical error will be almost negligible and the systematic errors shrink due to the better *b*-tagging. In total ${\varDelta } R_{b}^0= 0.00014$ can be reached which is an improvement of a factor 5 compared to the present value [877].

$\sin ^2\theta _{\mathrm {eff}}^{b}$ can be measured from the left–right–forward–backward asymmetry for *b*-quarks, $A_\mathrm{FB,LR}^b = 3/4 {\mathscr {P}} {\mathscr {A}}_{b}$. ${\mathscr {A}}_{b}$ depends on $\sin ^2\theta _{\mathrm {eff}}^{b}$ as shown in Eq. (114), however, in general one has ${g_{V_f}}/{g_{A_f}} = 1 - 4 q_f \sin ^2\theta _{\mathrm {eff}}^{f}$ and due to the small *b*-charge the dependence is very weak. At present $\sin ^2\theta _{\mathrm {eff}}^{b}$ is known with a precision of 0.016 from $A_\mathrm{FB,LR}^b$ measured at the SLC and the forward–backward asymmetries for *b*-quarks at LEP combined with $\sin ^2\theta _{\mathrm {eff}}^{\ell }$ measurements at LEP and SLC [879]. Using the left–right–forward–backward asymmetry only at the ILC an improvement by more than a factor 10 seems realistic [877].

The total *Z*-width ${\varGamma }_Z$ can be obtained from a scan of the resonance curve. The statistical error at GigaZ will be negligible and the systematic uncertainty will be dominated by the precision of the beam energy and the knowledge of beamstrahlung. If a spectrometer with a precision of $10^{-5}$ can be built, ${\varGamma }_Z$ can be measured with $1\,\mathrm{MeV}$ accuracy [877]. However, no detailed study on the uncertainty due to beamstrahlung exists.

#### Constraints to the MSSM from $\sin ^2{\theta ^\ell }_{\mathrm {eff}}$^48^

As for $M_W$ we review examples showing how the MSSM parameter space could be constrained by a precise measurement of $\sin ^2{\theta ^\ell }_{\mathrm {eff}}$. We also discuss the relevance of this measurement in a combined $M_W$–$\sin ^2{\theta ^\ell }_{\mathrm {eff}}$ analysis.

In the first example it is investigated whether the high accuracy achievable at the GigaZ option of the LC would provide sensitivity to indirect effects of SUSY particles even in a scenario where the (strongly interacting) superpartners are so heavy that they escape detection at the LHC [880].

**Fig. 113:**
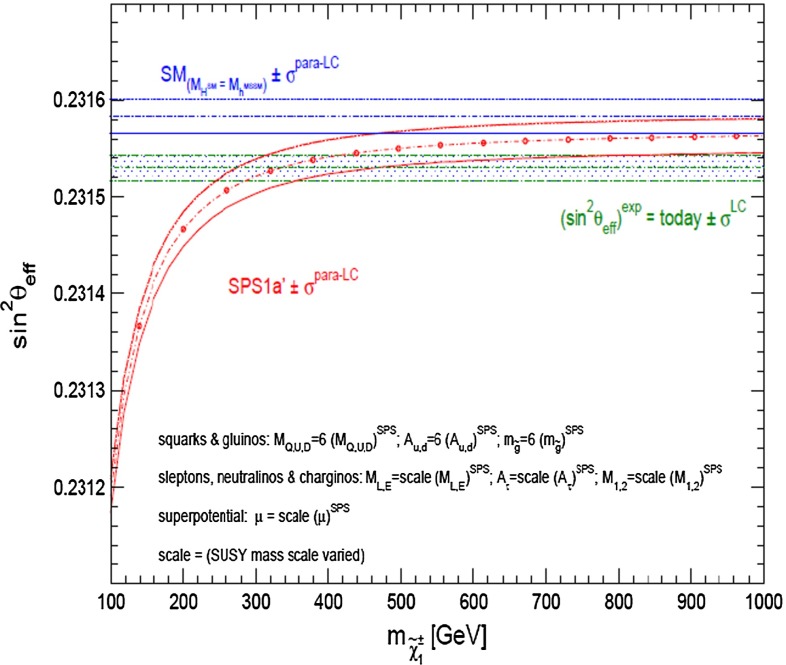
Theoretical prediction for $\sin ^2{\theta ^\ell }_{\mathrm {eff}}$ in the SM and the MSSM (including prospective parametric theoretical uncertainties) compared to the experimental precision at the LC with GigaZ option. An SPS 1a$'$ inspired scenario is used, where the *squark* and gluino mass parameters are fixed to 6 times their SPS 1a$'$ values. The other mass parameters are varied with a common scale factor

We consider in this context a scenario with very heavy squarks and a very heavy gluino. It is based on the values of the SPS 1a’ benchmark scenario [881], but the squark and gluino mass parameters are fixed to 6 times their SPS 1a’ values. The other masses are scaled with a common scale factor except $M_A$, which we keep fixed at its SPS 1a’ value. In this scenario the strongly interacting particles are too heavy to be detected at the LHC, while, depending on the scale factor, some colour-neutral particles may be in the LC reach. In Fig. 113 we show the prediction for $\sin ^2{\theta ^\ell }_{\mathrm {eff}}$ in this SPS 1a’ inspired scenario as a function of the lighter chargino mass, $m_{\tilde{\chi }^\pm _{1}}$. The prediction includes the parametric uncertainty, $\sigma ^{\text {para-LC}}$, induced by the LC measurement of $m_{t}\,$, $\delta m_{t}\,= 100\,\mathrm {MeV}\,$ (see Sect. 3), and the numerically more relevant prospective future uncertainty on ${\varDelta }\alpha ^{(5)}_{\text {had}}$, $\delta ({\varDelta }\alpha ^{(5)}_{\text {had}})=5\times 10^{-5}$. The MSSM prediction for $\sin ^2{\theta ^\ell }_{\mathrm {eff}}$ is compared with the experimental resolution with GigaZ precision, $\sigma ^\mathrm{LC} = 0.000013$, using for simplicity the current experimental central value. The SM prediction (with $M_H^\mathrm{SM}=M_h^\mathrm{MSSM}$) is also shown, applying again the parametric uncertainty $\sigma ^{\text {para-LC}}$.

Despite the fact that no coloured SUSY particles would be observed at the LHC in this scenario, the LC with its high-precision measurement of $\sin ^2{\theta ^\ell }_{\mathrm {eff}}$ in the GigaZ mode could resolve indirect effects of SUSY up to $m_{\tilde{\chi }^\pm _1} \lesssim 500\,\mathrm {GeV}\,$. This means that the high-precision measurements at the LC with GigaZ option could be sensitive to indirect effects of SUSY even in a scenario where SUSY particles have *neither* been directly detected at the LHC nor the first phase of the LC with a centre of mass energy of up to $500\,\mathrm {GeV}\,$.

We now analyse the sensitivity of $\sin ^2{\theta ^\ell }_{\mathrm {eff}}$ together with $M_W$ to higher-order effects in the MSSM by scanning over a broad range of the SUSY parameter space. The following SUSY parameters are varied independently of each other in a random parameter scan within the given range:

115 $$\begin{aligned}&\mathrm{sleptons} :M_{{\tilde{L}_{1,2,3}},{\tilde{E}_{1,2,3}}} = 100\ldots 2000\,\mathrm {GeV}\,, \nonumber \\&\mathrm{light~squarks} :M_{{\tilde{Q}_{1,2}},{\tilde{U}_{1,2}},{\tilde{D}_{1,2}}} = 100\ldots 2000\,\mathrm {GeV}\,, \nonumber \\&\quad \tilde{t}/\tilde{b}\mathrm{~doublet} :M_{{\tilde{Q}_3},{\tilde{U}_3}, {\tilde{D}_3}} = 100\ldots 2000\,\mathrm {GeV}\,, \nonumber \\&\quad A_{\tau ,t,b} = -2000\ldots 2000\,\mathrm {GeV}\,, \nonumber \\&\mathrm{gauginos} :M_{1,2}=100\ldots 2000\,\mathrm {GeV}\,, \end{aligned}$$

116 $$\begin{aligned}&\quad m_{\tilde{g}}=195\ldots 1500\,\mathrm {GeV}\,, \nonumber \\&\mu = -2000\ldots 2000\,\mathrm {GeV}\,, \nonumber \\&\mathrm{Higgs} :M_A=90\ldots 1000\,\mathrm {GeV}\,, \nonumber \\&\quad \tan \beta = 1.1\ldots 60. \end{aligned}$$

Only the constraints on the MSSM parameter space from the LEP Higgs searches [321, 882] and the lower bounds on the SUSY particle masses previous to the LHC SUSY searches were taken into account. However, the SUSY particles strongly affected by the LHC searches are the squarks of the first and second generation and the gluino. Exactly these particles, however, have a very small effect on the prediction of $M_W$ and $\sin ^2{\theta ^\ell }_{\mathrm {eff}}$ and thus a negligible effect on this analysis.

**Fig. 114:**
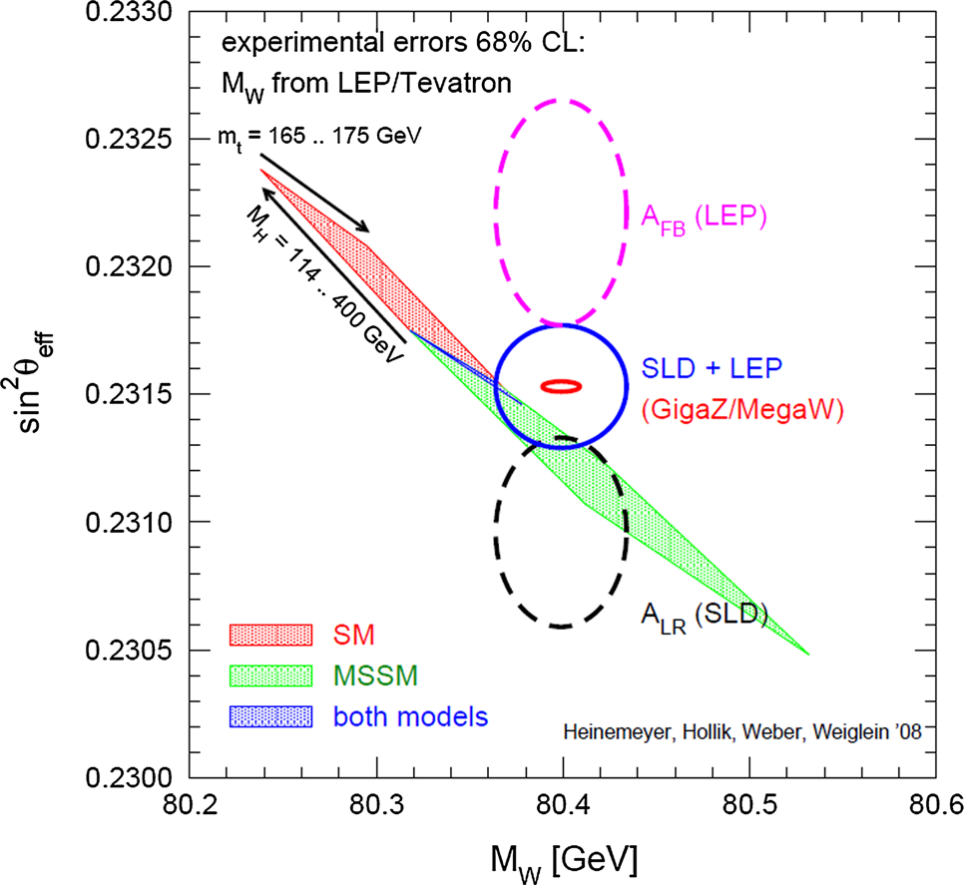
MSSM parameter scan for $M_W$ and $\sin ^2{\theta ^\ell }_{\mathrm {eff}}$ over the ranges given in Eq. (116) with $m_{t}\,= 165 \ldots 175 \mathrm {GeV}\,$. Todays 68 % CL ellipses (from $A_\mathrm{FB}^b(\mathrm{LEP})$, $A_\mathrm{LR}^e(\mathrm{SLD})$ and the world average) are shown as well as the anticipated GigaZ/MegaW precisions, drawn around todays central value

In Fig. 114 we compare the SM and the MSSM predictions for $M_W$ and $\sin ^2{\theta ^\ell }_{\mathrm {eff}}$ as obtained from the scatter data. The predictions within the two models give rise to two bands in the $M_W$–$\sin ^2{\theta ^\ell }_{\mathrm {eff}}$ plane with only a relatively small overlap region [indicated by a dark-shaded (blue) area]. The parameter region shown in the SM [the medium-shaded (red) and dark-shaded (blue) bands] arises from varying the mass of the SM Higgs boson, from $M_H^\mathrm{SM} = 114\,\mathrm {GeV}\,$, the old LEP exclusion bound [882] [lower edge of the dark-shaded (blue) area], to $400\,\mathrm {GeV}\,$ [upper edge of the medium-shaded (red) area], and from varying $m_{t}\,$ in the range of $m_{t}\,= 165 \ldots 175\,\mathrm {GeV}\,$. The value of $M_H^\mathrm{SM} \sim 125.5\,\mathrm {GeV}\,$ corresponds roughly to the dark-shaded (blue) strip. The light shaded (green) and the dark-shaded (blue) areas indicate allowed regions for the unconstrained MSSM, where no restriction on the light ${\mathscr {CP}}$-even Higgs mass has been applied. The decoupling limit with SUSY masses, in particular of scalar tops and bottoms, of ${\mathscr {O}}(2\,{\text {TeV}})$ yields the upper edge of the dark-shaded (blue) area. Including a Higgs mass measurement into the MSSM scan would cut out a small part at the lower edge of the light shaded (green) area.

The 68 % CL experimental results for $M_W$ and $\sin ^2{\theta ^\ell }_{\mathrm {eff}}$ are indicated in the plot. The centre ellipse corresponds to the current world average given in Eq. (119). Also shown are the error ellipses corresponding to the two individual most precise measurements of $\sin ^2{\theta ^\ell }_{\mathrm {eff}}$, based on $A_\mathrm{LR}^e$ by SLD and $A_\mathrm{FB}^b$ by LEP, corresponding to

117 $$\begin{aligned}&A_\mathrm{FB}^b(\mathrm{LEP}):\sin ^2{\theta ^\ell }_{\mathrm {eff}}^\mathrm{exp,LEP} = 0.23221 \pm 0.00029, \end{aligned}$$

118 $$\begin{aligned}&A_\mathrm{LR}^e(\mathrm{SLD}):\sin ^2{\theta ^\ell }_{\mathrm {eff}}^\mathrm{exp,SLD} = 0.23098 \pm 0.00026, \end{aligned}$$

119 $$\begin{aligned}&\sin ^2{\theta ^\ell }_{\mathrm {eff}}^\mathrm{exp,aver.} = 0.23153 \pm 0.00016~, \end{aligned}$$

where the latter one represents the average [21]. The first (second) value prefers a value of $M_H^\mathrm{SM} \sim 32 (437)\,\mathrm {GeV}\,$ [883]. The two measurements differ by more than $3\sigma$. The averaged value of $\sin ^2{\theta ^\ell }_{\mathrm {eff}}$, as given in Eq. (119), prefers $M_H^\mathrm{SM} \sim 110\,\mathrm {GeV}\,$ [883]. The anticipated improvement with the GigaZ/MegaW options (the latter one denoting the *WW* threshold scan, see Sect. 4.2), indicated as small ellipse, is shown around the current experimental central data. One can see that the current averaged value is compatible with the SM with $M_H^\mathrm{SM} \sim 125.5\,\mathrm {GeV}\,$ and with the MSSM. The value of $\sin ^2{\theta ^\ell }_{\mathrm {eff}}$ obtained from $A_\mathrm{LR}^e$(SLD) clearly favours the MSSM over the SM. On the other hand, the value of $\sin ^2{\theta ^\ell }_{\mathrm {eff}}$ obtained from $A^b_\mathrm{FB}$(LEP) together with the $M_W$ data from LEP and the Tevatron would correspond to an experimentally preferred region that deviates from the predictions of both models. This unsatisfactory solution can only be resolved by new measurements, where the a *Z* factory, i.e. the GigaZ option would be an ideal solution. Thus, the unclear experimental situation regarding the two single most precise measurements entering the combined value for $\sin ^2{\theta ^\ell }_{\mathrm {eff}}$ has a significant impact on the constraints that can be obtained from this precision observable on possible New Physics scenarios. Measurements at a new $e^+e^-$*Z* factory, which could be realised in particular with the GigaZ option of the ILC, would be needed to resolve this issue. As indicated by the solid light shaded (red) ellipse, the anticipated GigaZ/MegaW precision of the combined $M_W$–$\sin ^2{\theta ^\ell }_{\mathrm {eff}}$ measurement could put severe constraints on each of the models and resolve the discrepancy between the $A_\mathrm{FB}^b$(LEP) and $A_\mathrm{LR}^e$(SLD) measurements. If the central value of an improved measurement with higher precision should turn out to be close to the central value favoured by the current measurement of $A_\mathrm{FB}^b(\mathrm{LEP})$, this would mean that the EWPO $M_W$ and $\sin ^2{\theta ^\ell }_{\mathrm {eff}}$ could rule out both the SM and the most general version of the MSSM.

### The relevance of the top-quark mass^49^

The mass of the top quark, $m_{t}\,$, is a fundamental parameter of the electroweak theory. It is by far the heaviest of all quark masses and it is also larger than the masses of all other known fundamental particles. For details of the experimental determination of $m_{t}\,$, see Sect. 3.4.1. The top quark is deeply connected to many other issues of high-energy physics:

- The top quark could play a special role in/for EWSB.
- The experimental uncertainty of $m_{t}\,$ induces the largest parametric uncertainty in the prediction for EWPO [819, 884] and can thus obscure new physics effects.
- In SUSY models the top-quark mass is an important input parameter and is crucial for radiative EWSB and unification.
- Little Higgs models contain “heavier tops”.

The large value of $m_{t}\,$ gives rise to a large coupling between the top quark and the Higgs boson and is furthermore important for flavour physics. It could therefore provide a window to new physics. (The correct prediction of $m_{t}\,$ will be a crucial test for any fundamental theory.) The top-quark mass also plays an important role in electroweak precision physics, as a consequence in particular of non-decoupling effects being proportional to powers of $m_{t}\,$. A precise knowledge of $m_{t}\,$ is therefore indispensable in order to have sensitivity to possible effects of new physics in electroweak precision tests.

The current world average for the top-quark mass from the measurement at the Tevatron and the LHC is [885],

120 $$\begin{aligned} m_{t}\,= 173.34 \pm 0.76\,\mathrm {GeV}\,. \end{aligned}$$

The prospective accuracy at the LHC is $\delta m_{t}\,^\mathrm{exp} \approx 500\,\mathrm {MeV}\,$ [447], while at the ILC a very precise determination of $m_{t}\,$ with an accuracy of $\delta m_{t}\,^\mathrm{exp} \lesssim 100\,\mathrm {MeV}\,$ will be possible, see Sect. 3.4.1. This uncertainty contains both the experimental error of the mass parameter extracted from the $t \bar{t}$ threshold measurements at the ILC and the envisaged theoretical uncertainty from its transition into a suitable short-distance mass (like the $\overline{\text {MS}}$ mass).

The relevance of the $m_{t}\,$ precision as parametric uncertainty has been discussed for the *W* boson mass, $M_W$, in Sect. 4.2, and for the effective leptonic weak mixing angle, $\sin ^2{\theta ^\ell }_{\mathrm {eff}}$, in Sect. 4.3.

**Fig. 115:**
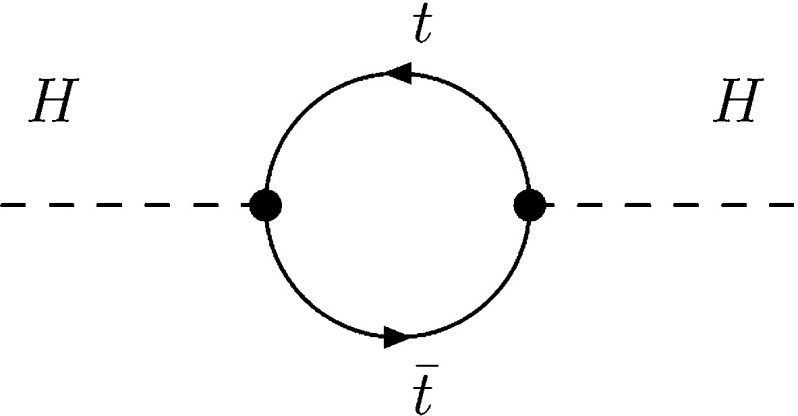
Loop contribution of the top quark to the Higgs-boson mass

Because of its large mass, the top quark is expected to have a large Yukawa coupling to Higgs bosons, being proportional to $m_{t}\,$. In each model where the Higgs-boson mass is not a free parameter but predicted in terms of the other model parameters (as e.g. in the MSSM), the diagram in Fig. 115 contributes to the Higgs mass. This diagram gives rise to a leading $m_{t}\,$ contribution of the form

121 $$\begin{aligned} {\varDelta }M_H^2 \sim G_F\; N_C \; C \; m_{t}\,^4, \end{aligned}$$

where $G_F$ is the Fermi constant, $N_C$ is the colour factor, and the coefficient *C* depends on the specific model. Thus the experimental error of $m_{t}\,$ necessarily leads to a parametric error in the Higgs-boson mass evaluation.

Taking the MSSM as a specific example (including also the scalar top contributions and the appropriate renormalisation) $N_C \, C$ is given for the light ${\mathscr {CP}}$-even Higgs-boson mass in leading logarithmic approximation by

122 $$\begin{aligned} N_C \, C = \frac{3}{\sqrt{2}\,\pi ^2\,\sin ^2\!\beta } \; \log \left( \frac{m_{\tilde{t}_1}m_{\tilde{t}_2}}{m_{t}\,^2} \right) ~. \end{aligned}$$

Here $m_{\tilde{t}_{1,2}}$ denote the two masses of the scalar tops. The current precision of $\delta m_{t}\,\sim 1 \mathrm {GeV}\,$ leads to an uncertainty of $\sim$2.5 % in the prediction of $M_H$, while the ILC will yield a precision of $\sim$ 0.2%. These uncertainties have to be compared with the anticipated precision of the future Higgs boson mass measurements. With a precision of $\delta M_H^\mathrm{exp,LHC} \approx 0.2\,\mathrm {GeV}\,$ the relative precision is at the level of $\sim$0.2 %. It is apparent that only the LC precision of $m_{t}\,$ will yield a parametric error small enough to allow a precise comparison of the Higgs-boson mass prediction and its experimental value.

Another issue that has to be kept in mind here (in SUSY as in any other model predicting $M_H$) is the intrinsic theoretical uncertainty due to missing higher-order corrections. Within the MSSM currently the uncertainty for the lightest ${\mathscr {CP}}$-even Higgs is estimated to $\delta M_h^\mathrm{intr,today} \approx 2$–$3\,\mathrm {GeV}\,$ [226, 819].^50^ In the future one can hope for an improvement down to $\lesssim 0.5\,\mathrm {GeV}\,$ or better [819], i.e. with sufficient effort on higher-order corrections it should be possible to reduce the intrinsic theoretical uncertainty to the level of $\delta M_H^\mathrm{exp, LHC}$.

Confronting the theoretical prediction of $M_H$ with a precise measurement of the Higgs-boson mass constitutes a very sensitive test of the MSSM (or any other model that predicts $M_H$), which allows one to obtain constraints on the model parameters. However, the sensitivity of the $M_H$ measurement cannot directly be translated into a prospective indirect determination of a single model parameter. In a realistic situation the anticipated experimental errors of *all* relevant SUSY parameters have to be taken into account. For examples including these parametric errors see Refs. [491, 884].

### Prospects for the electroweak fit to the SM Higgs mass^51^

The global fit to electroweak precision data allows among other constraints to extract information on the Higgs mass from Higgs loops modifying the values of *Z* boson asymmetry observables and the *W* mass [21, 823, 886–888]. Assuming the new boson discovered by the ATLAS [61] and CMS [62] experiments at the LHC to be the SM Higgs boson, the electroweak fit is overconstrained and can be used to quantify the compatibility of the mass (and couplings) of the discovered boson with the electroweak precision data in an overall goodness-of-fit measure. Similarly, it allows one to confront indirect determinations of the *W* boson mass, the effective weak mixing angle predicting the *Z* asymmetries, and the top-quark mass with the measurements. The LHC and a next generation electron–positron collider have the potential to significantly increase the precision of most of the observables that are relevant to the fit. This section reports on a prospective study of the electroweak fit following the approach published in earlier works by the Gfitter group [888–890] (and compares briefly to a corresponding fit from the LEPEWWG).

**Table 28:** Input values and fit results for the observables and parameters of the global electroweak fit in a hypothetical future scenario. The first and second columns list respectively the observables/parameters used in the fit, and their experimental values or phenomenological estimates (see text for references). The subscript “theo” labels theoretical error ranges. The third column indicates whether a parameter is floating in the fit and in the fourth column the fit results are given without using the corresponding experimental or phenomenological estimate in the given row

Parameter	Input value	Free in fit	Predicted fit result
$M_{H}$ [GeV]	$125.8 \pm 0.1$	Yes	$125.0^{\,+12}_{\,-10}$
$M_{W}$ [GeV]	$80.378\pm 0.006$	–	$80.361\pm 0.005$
${\varGamma }_{W}$ [GeV]	–	–	$2.0910\pm 0.0004$
$M_{Z}$ [GeV]	$91.1875\pm 0.0021$	Yes	$91.1878 \pm 0.0046$
${\varGamma }_{Z}$ [GeV]	–	–	$2.4953\pm 0.0003$
$\sigma _\mathrm{had}^{0}$ [nb]	–	–	$41.479\pm 0.003$
$R^{0}_{\ell }$	$20.742\pm 0.003$	–	–
$A_\mathrm{FB}^{0,\ell }$	–	–	$0.01622 \pm 0.00002$
$A_\ell$	–	–	$0.14706 \pm 0.00010$
$\sin \!^2\theta ^{\ell }_{\mathrm{eff}}$	$0.231385\pm 0.000013$	–	$0.23152\pm 0.00004$
$A_{c}$	–	–	$0.66791\pm 0.00005$
$A_{b}$	–	–	$0.93462\pm 0.00002$
$A_\mathrm{FB}^{0,c}$	–	–	$0.07367\pm 0.00006$
$A_\mathrm{FB}^{0,b}$	–	–	$0.10308\pm 0.00007$
$R^{0}_{c}$	–	–	$0.17223\pm 0.00001$
$R^{0}_{b}$	–	–	$0.214746\pm 0.000004$
$\overline{m}_c\,$ [GeV]	$1.27^{+0.07}_{-0.11}$	Yes	–
$\overline{m}_b\,$ [GeV]	$4.20^{+0.17}_{-0.07}$	Yes	–
$m_{t}$ [GeV]	$173.18\pm 0.10$	Yes	$173.3\pm 1.2$
${\varDelta }\alpha _\mathrm{had}^{(5)}(M_Z^2)\,$ $^{(\bigtriangleup )}$	$2757.0\pm 4.7$	Yes	$2757 \pm 10$
$\alpha _s(M_{Z}^{2})$	–	Yes	$0.1190\pm 0.0005$
$\delta _\mathrm{th}\,M_W$ [MeV]	$[-2.0,2.0]_\mathrm{theo}$	Yes	–
$\delta _\mathrm{th}\,\sin \!^2\theta ^{\ell }_{\mathrm{eff}}$ $^{(\bigtriangledown )}$	$[-1.5,1.5]_\mathrm{theo}$	Yes	–

$^{(\bigtriangleup )}$ In units of $10^{-5}$. $^{(\bigtriangledown )}$ Rescaled due to $\alpha _s$ dependency

For the study aiming at a comparison of the accuracies of the measured and predicted electroweak observables, the central values of the input observables are chosen to agree with the SM prediction for a Higgs mass of 125.8 GeV. Total experimental uncertainties of 6 MeV for $M_W$, $1.3 \cdot 10^{-5}$ for $\sin \!^2\theta ^{\ell }_{\mathrm{eff}}$, $4\cdot 10^{-3}$ for $R^{0}_{\ell }$, and 100 MeV for $m_t$ (interpreted as pole mass) are used. The exact achieved precision on the Higgs mass is irrelevant for this study. For the hadronic contribution to the running of the QED fine structure constant at the *Z* pole, ${\varDelta }\alpha _\mathrm{had}^{(5)}(M_Z^2)\,$, an uncertainty of $4.7\cdot 10^{-5}$ is assumed (compared to the currently used uncertainty of $10\cdot 10^{-5}$ [890, 891]), which benefits below the charm threshold from the completion of BABAR analyses and the on-going programme at VEPP-2000, and at higher energies from improved charmonium resonance data from BES-3, and a better knowledge of $\alpha _s$ from the $R^{0}_{\ell }$ measurement and reliable lattice QCD predictions. The other input observables to the electroweak fit are taken to be unchanged from the current settings [890].

For the theoretical predictions, the calculations detailed in [888] and references therein are used. They feature among others the complete $\mathscr {O}(\alpha _s^4)$ calculation of the QCD Adler function [661, 662] and the full two-loop and leading beyond-two-loop prediction of the *W* mass and the effective weak mixing angle [848, 849, 892]. An improved prediction of $R^0_b$ is invoked that includes the calculation of the complete fermionic electroweak two-loop (NNLO) corrections based on numerical Mellin–Barnes integrals [870]. The calculation of the vector and axial-vector couplings in Gfitter relies on accurate parametrisations [893–896].

The most important theoretical uncertainties in the fit are those affecting the $M_W$ and $\sin \!^2\theta ^{\ell }_{\mathrm{eff}}$ predictions. They arise from three dominant sources of unknown higher-order corrections: ${\mathscr {O}}(\alpha ^2\alpha _s)$ terms beyond the known contribution of ${\mathscr {O}}(G_{F}^2 \alpha _s m_{t}\,^4)$, ${\mathscr {O}}(\alpha ^3)$ electroweak three-loop corrections, and ${\mathscr {O}}(\alpha _s^3)$ QCD terms, see Sect. 4.3.1. The quadratic sums of the above corrections amount to $\delta _\mathrm{th}\,M_W=4\,\mathrm {MeV}\,$ and $\delta _\mathrm{th}\,\sin \!^2\theta ^{\ell }_{\mathrm{eff}}=4.7 \cdot 10^{-5}$, which are the theoretical ranges used in present electroweak fits. We assume in the following that theoretical developments have let to improved uncertainties of $\delta _\mathrm{th}\,M_W=2\,\mathrm {MeV}\,$ and $\delta _\mathrm{th}\,\sin \!^2\theta ^{\ell }_{\mathrm{eff}}=1.5 \cdot 10^{-5}$, see Table 28. Within the *R*fit scheme employed here [897, 898], theoretical uncertainties are treated as uniform likelihoods in the fit, corresponding to an allowed offset from the predicted value within the defined range (we discuss the difference with respect to standard Gaussian theoretical uncertainties below).

**Fig. 116:**
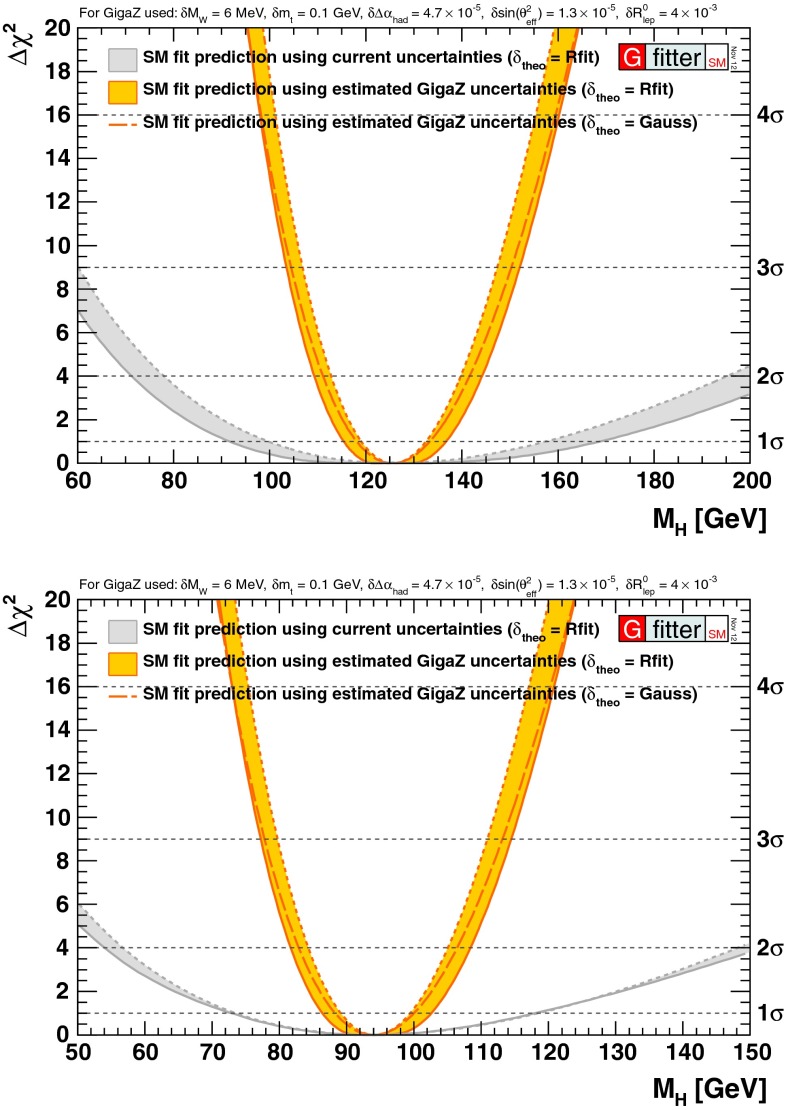
${\varDelta }\chi ^2$ profiles as a function of the Higgs mass for electroweak fits compatible with an SM Higgs boson of mass 125.8 $\mathrm {GeV}$ (*left*) and 94 $\mathrm {GeV}$ (*right*), respectively. The measured Higgs-boson mass is not used as input in the fit. The *grey* bands show the results obtained using present uncertainties [890], and the *yellow* bands indicate the results for the hypothetical future scenario given in Table 28 (*left plot*) and corresponding input data shifted to accommodate a 94 $\mathrm {GeV}$ Higgs boson but unchanged uncertainties (*right plot*). The right axes depict the corresponding Gaussian ‘sigma’ lines. The *thickness* of the bands indicates the effect from the theoretical uncertainties treated according to the *R*fit prescription. The *long-dashed line* in *each plot* shows the curves one would obtain when treating the theoretical uncertainties in a Gaussians manner just like any other uncertainty in the fit

**Fig. 117:**
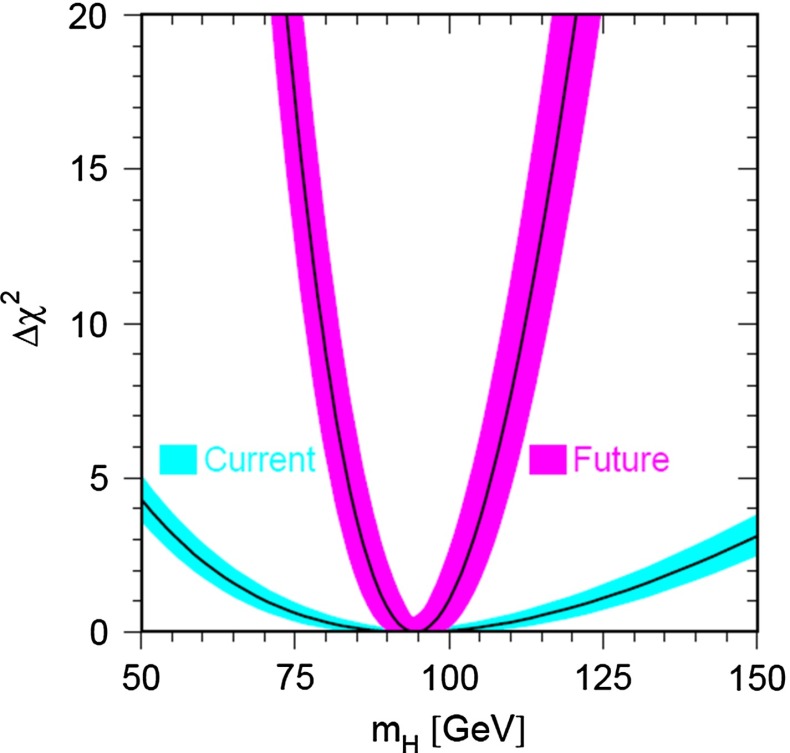
${\varDelta }\chi ^2$ profiles as a function of the Higgs mass for electroweak fits compatible with an SM Higgs boson with mass 94 $\mathrm {GeV}$ using the LEPEWWG approach [21]. The *blue* (*pink*) parabola shows the current (future) fit (see text)

Table 28 gives the input observables and values used (first and second columns) and the predictions obtained from the fit to all input data except for the one that is predicted in a given row (last column). It allows one to compare the accuracy of direct and indirect determinations. To simplify the numerical exercise the *Z*-pole asymmetry observables are combined into a single input $\sin \!^2\theta ^{\ell }_{\mathrm{eff}}$, while for the reader’s convenience the fit predictions are provided for all observables.

The indirect prediction of the Higgs mass at 125 $\mathrm {GeV}$  achieves an uncertainty of $^{+12}_{-10}\,\mathrm {GeV}\,$. For $M_W$ the prediction with an estimated uncertainty of 5 $\mathrm {MeV}$ is similarly accurate as the (assumed) measurement, while the prediction of $\sin \!^2\theta ^{\ell }_{\mathrm{eff}}$ with an uncertainty of $4\cdot 10^{-5}$ is three times less accurate than the experimental precision. The fit would therefore particularly benefit from additional experimental improvement in $M_W$. It is interesting to notice that the accuracy of the indirect determination of the top mass (1.2 $\mathrm {GeV}$ ) becomes similar to that of the present experimental determination. An improvement beyond, say, 200 $\mathrm {MeV}$ uncertainty cannot be exploited by the fit. The input values of $M_Z$ and ${\varDelta }\alpha _\mathrm{had}^{(5)}(M_Z^2)\,$ are twice more accurate than the fit predictions, which is sufficient to not limit the fit but further improvement would certainly be useful.

Keeping the present theoretical uncertainties in the prediction of $M_W$ and $\sin \!^2\theta ^{\ell }_{\mathrm{eff}}$ would worsen the accuracy of the $M_H$ prediction to $^{+20}_{-17}\,\mathrm {GeV}\,$, whereas neglecting theoretical uncertainties altogether would improve it to $\pm$7 $\mathrm {GeV}$ . This emphasises the importance of the required theoretical work.

Profiles of ${\varDelta }\chi ^2$ as a function of the Higgs mass for present and future electroweak fits compatible with an SM Higgs boson of mass 125.8 and 94 $\mathrm {GeV}$ , respectively, are shown in Fig. 116 (see caption for a detailed description). The measured Higgs-boson mass is not used as input in these fits. If the experimental input data, currently predicting $M_H=94^{+25}_{-22}\,\mathrm {GeV}\,$ [890], were left unchanged with respect to the present values, but had uncertainties as in Table 28, a deviation of the measured $M_H$ exceeding $4\sigma$ could be established with the fit (see right-hand plot in Fig. 116). Such a conclusion does not strongly depend on the treatment of the theoretical uncertainties (*R*fit versus Gaussian) as can be seen by comparison of the solid yellow and the long-dashed yellow ${\varDelta }\chi ^2$ profiles.

A similar result has also been obtained by the LEPEWWG, as can be seen in Fig. 117 [21]. The ${\varDelta }\chi ^2$ profile of their fit is shown as a function of the Higgs mass. The blue band shows the current result with a best-fit point at $\sim$94 $\mathrm {GeV}$ with an uncertainty of $\sim \pm 30 \mathrm {GeV}\,$. The pink parabola shows the expected improvement under similar assumptions to Fig. 116. This confirms that a strong improvement of the fit can be expected taking into account the anticipated future LC accuracy for the electroweak precision data.

### The muon magnetic moment and new physics^52^

One of the prime examples of precision observables sensitive to quantum effects are the magnetic moments $(g-2)$ of the electron and muon. In particular after the measurements at Brookhaven [22], the muon magnetic moment $a_\mu =(g_\mu -2)/2$ has reached a sensitivity to all sectors of the SM and to many NPM. The currently observed deviation between the experimental value and the SM prediction is particularly well compatible with NPM which can also be tested at a LC. Before the startup of a future LC, new $a_\mu$ measurements are planned at Fermilab [23] and J-PARC [24]. For these reasons it is of interest to briefly discuss the conclusions that can be drawn from current and future $a_\mu$ results on LC physics.

Like many LC precision observables, $a_\mu$ is a flavour- and ${\textit{CP}}$-conserving quantity; unlike the former it is chirality-flipping and therefore particularly sensitive to modifications of the muon Yukawa coupling or more generally the muon mass-generation mechanism. A simple consideration, however, demonstrates that like a LC, $a_\mu$ is generically sensitive to NPM with new weakly interacting particles at the weak scale [899].

Because of the similar quantum field theory operators relevant for $m_\mu$ and $a_\mu$, contributions of a NPM at some scale ${\Lambda }$ to both quantities, $a_\mu (\text{ N.P. })$ and $\delta m_\mu (\text{ N.P. })$, are linked as

123 $$\begin{aligned} a_\mu (\text{ N.P. })={\mathscr {O}}(1)\times \left( \frac{m_\mu }{{\Lambda }}\right) ^2 \times \left( \frac{\delta m_\mu (\text{ N.P. })}{m_\mu }\right) . \end{aligned}$$

All coupling constants and loop factors are contained in the constant $C := \delta m_\mu (\text{ N.P. })/m_\mu$, which is highly model-dependent. A first consequence of this relation is that new physics can explain the currently observed deviation of [900] (based on [891]),

124 $$\begin{aligned} a_\mu ^\mathrm{exp} - a_\mu ^\mathrm{SM} = (28.7 \pm 8.0)\times 10^{-10}, \end{aligned}$$

only if ${\Lambda }$ is at the TeV scale or smaller (assuming no fine tuning in the muon mass, $|C|<1$).

Equation (123) also illustrates how widely different contributions to $a_\mu$ are possible.

- For models with new weakly interacting particles (e.g. $Z'$, $W'$, see Sect. 4.7, little Higgs or universal extra dimension models) one typically obtains perturbative contributions to the muon mass $C={\mathscr {O}}(\alpha /4\pi )$. Hence, for weak-scale masses these models predict very small contributions to $a_\mu$ and might be challenged by the future more precise $a_\mu$ measurement, see e.g. [901, 902]. Models of this kind can only explain a significant contribution to $a_\mu$ if the new particles interact with muons but are otherwise hidden from the searches. An example is the model with a new gauge boson associated to a gauged lepton number $L_\mu -L_\tau$ [903, 904], where a gauge boson mass of ${\mathscr {O}}(100 \text{ GeV })$ is viable, If this model is the origin of the observed $a_\mu$ deviation it would be highly desirable to search for the new $Z'$, corresponding to the $L_\mu -L_\tau$-symmetry. This would be possible at the LHC in part of the parameter space but also at the LC in the process $e^+e^-\rightarrow \mu ^+\mu ^-Z'$ [903, 904].
- For SUSY models one obtains an additional factor $\tan \beta$, the ratio of the two Higgs vacuum expectation values, see e.g. [905] and references therein. A numerical approximation for the SUSY contributions is given by
  125 $$\begin{aligned} a_\mu ^\mathrm{SUSY} \approx 13\times 10^{-10}\left( \frac{100\,\mathrm GeV}{M_\mathrm{SUSY}}\right) ^2\, \tan \beta \ \text{ sign }(\mu ), \end{aligned}$$
  where $M_\mathrm{SUSY}$ denotes the common superpartner mass scale and $\mu$ the Higgsino mass parameter. It agrees with the generic result Eq. (123) for $C={\mathscr {O}}(\tan \beta \times \alpha /4\pi )$ and is exactly valid if all SUSY masses are equal to $M_\mathrm{SUSY}$. The formula shows that the observed deviation could be explained e.g. for relevant SUSY masses (smuon, chargino and neutralino masses) of roughly 200 GeV and $\tan \beta \sim 10$ or SUSY masses of 500 GeV and $\tan \beta \sim 50$. This is well in agreement with current bounds on weakly interacting SUSY particles and in a very interesting range for a LC. This promising situation has motivated high-precision two-loop calculations of $a_\mu ^\mathrm{SUSY}$ [906, 907], which depend on all sfermion, chargino and neutralino masses and will benefit particularly from precise SUSY mass measurements at a LC.
- Models with large $C\simeq 1$ are of interest since there the muon mass is essentially given by new physics loop effects. Some examples of such radiative muon mass-generation models are given in [899]. For examples within SUSY see e.g. [908, 909]. In such models $a_\mu$ can be large even for particle masses at the TeV scale, potentially beyond the direct reach of a LC. The possibility to test such models using precision observables at the LC has not yet been explored in the literature.

**Fig. 118:**
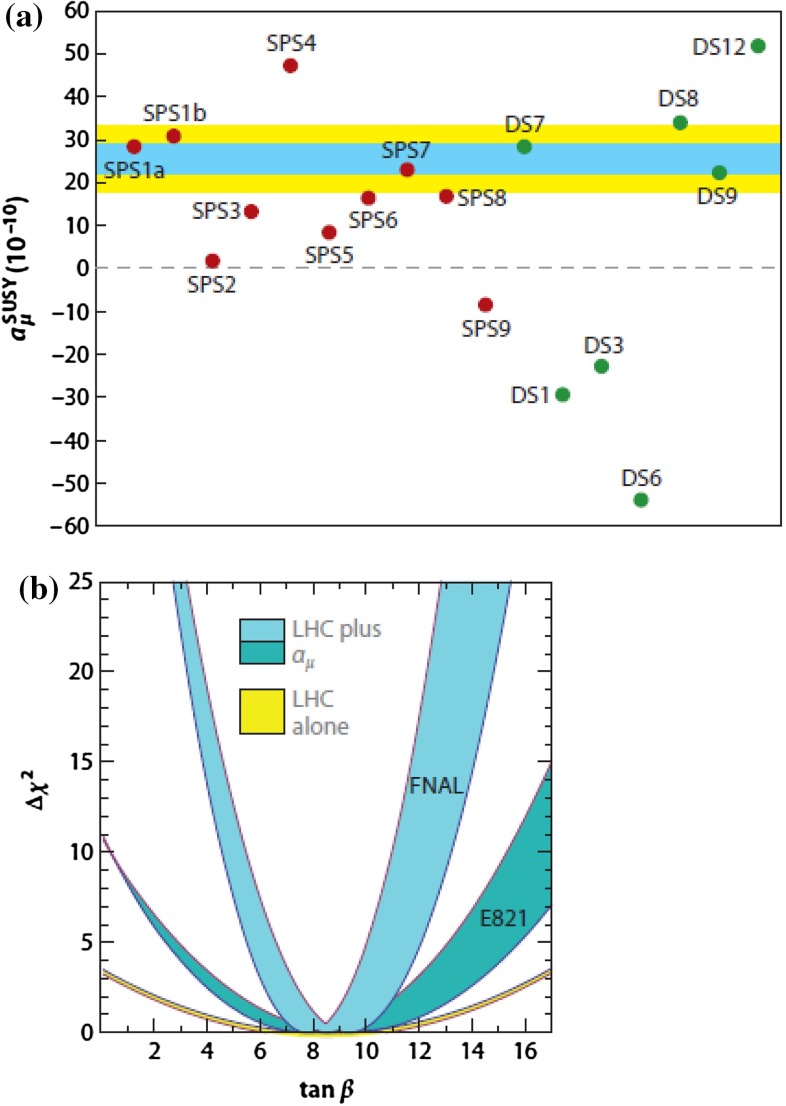
**a** SUSY contributions to $a_\mu$ for the SPS benchmark points (*red*), and for the “degenerate solutions” from Ref. [910]. The *yellow* and *blue* band indicate the current and an improved experimental result, respectively. **b** Possible future $\tan \beta$ determination assuming that a slightly modified MSSM point SPS1a (see text) is realised. The bands show the ${\varDelta }\chi ^2$ parabolas from LHC data alone (*yellow*) [911], including the $a_\mu$ with current precision (*dark blue*) and with prospective precision (*light-blue*). The width of the *blue* *curves* results from the expected LHC uncertainty of the parameters (mainly smuon and chargino masses) [911]. Taken from [912]

Figure 118 illustrates the complementarity of $a_\mu$ and LC measurements in investigating SUSY.

The upper plot shows the $a_\mu (\mathrm SUSY)$-values for the SPS benchmark points [881], of which only the weakly interacting sector is relevant. The contributions span a wide range and can be positive or negative.^53^ The discriminating power of the current (yellow band) and an improved (blue band) measurement is evident from Fig. 118a. The green points illustrate that the LHC alone is not sufficient to discover SUSY and measure all its parameters. They correspond to “degenerate solutions” as defined in Ref. [910] – different SUSY parameter points which cannot be distinguished at the LHC alone. They have very different $a_\mu$ predictions, in particular different signs for $\mu$, and hence $a_\mu$ can resolve such LHC degeneracies. However, the LC can go much further and rule out the wrong parameter choices with far higher significance [910].

The lower plot of Fig. 118 illustrates that the SUSY parameter $\tan \beta$ can be measured more precisely by combining LHC data with $a_\mu$. It is based on the assumption that SUSY is realised, found at the LHC and the origin of the observed $a_\mu$ deviation in Eq. (124). To fix an example, we use a slightly modified SPS1a benchmark point with $\tan \beta$ scaled down to $\tan \beta =8.5$ such that $a_\mu ^\mathrm{SUSY}$ is equal to an assumed deviation ${\varDelta } a_\mu =255\times 10^{-11}$.^54^ Reference [911] has shown that then mass measurements at the LHC alone are sufficient to determine $\tan \beta$ to a precision of $\pm$4.5 only. The corresponding ${\varDelta }\chi ^2$ parabola is shown in yellow in the plot. In such a situation one can study the SUSY prediction for $a_\mu$ as a function of $\tan \beta$ (all other parameters are known from the global fit to LHC data) and compare it to the measured value, in particular after an improved measurement. The plot compares the LHC ${\varDelta }\chi ^2$ parabola with the ones obtained from including $a_\mu$, ${\varDelta }\chi ^2=[(a_\mu ^\mathrm{SUSY}(\tan \beta )-{\varDelta } a_\mu )/\delta a_\mu ]^2$ with the errors $\delta a_\mu =80\times 10^{-11}$ (dark blue) and $34\times 10^{-11}$ (light-blue). Here the widths of the parabolas mainly originate in the experimental uncertainties of the relevant electroweak particles, such as smuons and charginos. It can be seen that on the one hand future measurements of $a_\mu$ would drastically improve the $\tan \beta$ determination. On the other hand, an LC measurement of the electroweak masses would also be important to obtain a very good fit to $\tan \beta$.

Reference [910] has also studied the impact of a LC on the $\tan \beta$-determination in a similar context, and a similar improvement was found as in the case of $a_\mu$. Here it is noteworthy that in the MSSM, $\tan \beta$ is a universal quantity entering all sectors, like $\sin \theta _W$ in the SM, but that $a_\mu$ and LC measurements are sensitive to $\tan \beta$ in different sectors, the muon Yukawa coupling and sparticle masses, respectively. These examples show how the LC will complement information from $a_\mu$ and test NPM compatible with $a_\mu$.

The situation would be quite different if the $a_\mu$ deviation is real but not due to weak-scale new particles but to very light, sub-GeV new particles, as suggested e.g. in [913]. In such a case, such new light dark-force particles could be probed by dedicated low-energy precision experiments such as the next generation $a_\mu$ measurements, but the full understanding of whatever physics at the electroweak scale there is to be found at the LHC would be left as a task of a future LC.

### Anomalous gauge boson couplings

#### Electroweak gauge boson interactions: effective field theory and anomalous couplings^55^

One possibility to search for new physics in the electroweak sector is the precision investigation of the couplings of the electroweak gauge bosons. At the LC at tree level, the incoming leptons interact via an exchange of an electroweak gauge boson. This allows for precise studies of tri-linear gauge couplings in $e^{+} e^{-} \rightarrow W^{+} W^{-}$ as well as quartic gauge couplings occurring in a variety of final states like $e^{+} e^{-} \rightarrow V V V$ with *VVV* being *WWZ* or $W W \gamma$. In contrast to a hadron collider the advantages are the absence of parton distribution functions so that the centre-of-mass energy at which the hard scattering takes place is exactly known. This also allows one to tune the beam energy according to the occurring resonances similar to what has already be done at LEP. The second advantage is the clean environment. At a hadron collider the most likely processes involve QCD radiation and therefore jets in the final state. Triple or quartic gauge boson scatterings are typically detected via VBF processes which however have to be discriminated from irreducible background processes.

One approach to parametrise new physics in a model-independent way is to write down an effective Lagrangian with all possible vertices and general coupling constants. For the tri-linear electroweak gauge couplings (TGC) this has been suggested in [914] for instance, resulting in the following effective Lagrangian including anomalous TGCs:

126 $$\begin{aligned} {\mathscr {L}}_\mathrm{TGC}= & {} ig_{WWV}\biggl (g_1^V(W_{\mu \nu }^+W^{-\mu }-W^{+\mu }W_{\mu \nu }^-)V^\nu \nonumber \\&+\,\kappa ^V W_\mu ^+W_\nu ^-V^{\mu \nu } +\frac{\lambda ^V}{M_W^2}W_\mu ^{\nu +}W_\nu ^{-\rho }V_\rho ^{\mu }\nonumber \\&+\,ig_4^VW_\mu ^+W^-_\nu (\partial ^\mu V^\nu +\partial ^\nu V^\mu ) \nonumber \\&-\,ig_5^V\epsilon ^{\mu \nu \rho \sigma }(W_\mu ^+\partial _\rho W^-_\nu -\partial _\rho W_\mu ^+W^-_\nu )V_\sigma \nonumber \\&\quad +\,\tilde{\kappa }^V W_\mu ^+W_\nu ^-\tilde{V}^{\mu \nu } +\frac{\tilde{\lambda }^V}{m_W^2}W_\mu ^{\nu +}W_\nu ^{-\rho }\tilde{V}_\rho ^{\mu } \biggr ),\nonumber \\ \end{aligned}$$

with $V=\gamma ,Z$; $W_{\mu \nu }^\pm = \partial _\mu W_\nu ^\pm - \partial _\nu W_\mu ^\pm$, $V_{\mu \nu } = \partial _\mu V_\nu - \partial _\nu V_\mu$ and $\tilde{V}_{\mu ,\nu }=\epsilon _{\mu \nu \rho \sigma } V_{\rho \sigma }/2$. The overall coupling constants are given by $g_{WW\gamma }=-e$ and $g_{WWZ}=-e\cot \theta _W$ (with $\cos \theta _W = M_W/M_Z$). In the same spirit, one can write down an effective Lagrangian describing quartic gauge boson couplings (QGC) as follows [915]:

127 $$\begin{aligned} {\mathscr {L}}_\mathrm{QGC}= & {} e^2\left( g_1^{\gamma \gamma }A^{\mu }A^{\nu }W^{-}_{\mu }W^{+}_{\nu } -g_2^{\gamma \gamma }A^{\mu }A_{\mu }W^{-\nu }W^{+}_{\nu }\right) \nonumber \\&+\,e^2\frac{c_w}{s_w}\left( g_1^{\gamma Z} A^{\mu } Z^{\nu }(W^{-}_{\mu }W^{+}_{\nu } +W^{+}_{\mu }W^{-}_{\nu }) \right. \nonumber \\&-\left. 2g_2^{\gamma Z}A^{\mu } Z_{\mu }W^{-\nu }W^{+}_{\nu }\right) \nonumber \\&+\,e^2\frac{c^2_w}{s^2_w}\left( g_1^{Z Z} Z^{\mu }Z^{\nu }W^{-}_{\mu }W^{+}_{\nu }\right. \nonumber \\&\left. -\,g_2^{Z Z} Z^{\mu }Z^{\mu }W^{-\nu }W^{+}_{\nu }\right) \nonumber \\&+\frac{e^2}{2s^2_w}\left( g_1^{WW}W^{-\mu }W^{+\nu }W^{-}_{\mu }W^{+}_{\nu }\right. \nonumber \\&\left. -\,g_2^{WW}(W^{-\mu }W^{+}_{\mu })^2\right) +\frac{e^2}{4s^2_wc^4_w}h^{ZZ}(Z^{\mu }Z_{\mu })^2. \end{aligned}$$

In the SM the couplings in Eq. (126) are given by

128 $$\begin{aligned} g_1^{\gamma ,Z} =\kappa ^{\gamma , Z} =1, \quad g_{4,5}^{\gamma ,Z} =\tilde{\kappa }^{\gamma , Z} =1, \quad \lambda ^{\gamma ,Z}=\tilde{\lambda }^{\gamma ,Z}=0, \end{aligned}$$

whereas the SM values of the QGCs are

129 $$\begin{aligned} g_1^{VV'}=g_2^{VV'}=1 (VV' = \gamma \gamma , \gamma Z, ZZ, WW),\quad h^{ZZ}=0. \end{aligned}$$

In the context of the recent discovery of a particle compatible with a SM Higgs boson [241, 242] it will be interesting to study the couplings of the Higgs boson to the electroweak gauge bosons. A parametrisation of tri-linear couplings can be found in [916, 917], for instance, and reads

130 $$\begin{aligned} {\mathscr {L}}_\mathrm{TGC}^{H}= & {} g_{H \gamma \gamma } H A_{\mu \nu } A^{\mu \nu } + g^{(1)}_{H Z \gamma } A_{\mu \nu } Z^{\mu } \partial ^{\nu } H \nonumber \\&+\, g^{(2)}_{H Z \gamma } H A_{\mu \nu } Z^{\mu \nu } + g^{(1)}_{H Z Z} Z_{\mu \nu } Z^{\mu } \partial ^{\nu } H \nonumber \\&+\, g^{(2)}_{H Z Z} H Z_{\mu \nu } Z^{\mu \nu } + g^{(2)}_{H W W} H W^+_{\mu \nu } W_{-}^{\mu \nu } \; \nonumber \\&+\, g^{(1)}_{H W W} \left( W^+_{\mu \nu } W_{-}^{\mu } \partial ^{\nu } H +\mathrm{h.c.}\right) . \end{aligned}$$

Note that none of the terms in Eq. (130) has a SM contribution as the *HVV* vertex in the SM is given by

131 $$\begin{aligned} {\mathscr {L}}_\mathrm{SM}^{H}= \frac{1}{2} \frac{g}{\cos \theta _W} M_ZH Z_{\mu } Z^{\mu } + g M_WW^{+}_{\mu } W^{-\mu }. \end{aligned}$$

In Eqs. (126), (127), (130) the number of possible additional interaction terms in the Lagrangian is restricted by the requirement of electroweak gauge and Lorentz invariance. If one loosens this requirement, there would be many more possibilities as discussed for instance in [918].

A slightly different approach to a model-independent parametrisation of new physics is based on the idea of an effective field theory (EFT) [919–925], where additional, higher-dimensional operators are added to the SM Lagrangian,

132 $$\begin{aligned} {\mathscr {L}}_\mathrm{eff}= {\mathscr {L}}_\mathrm{SM} +\sum _{n=1}^{\infty }\sum _{i}\frac{f_{i}^{(n)}}{{\Lambda }^n}{\mathscr {O}}_i^{(n+4)}. \end{aligned}$$

As the Lagrangian is required to have dimension four, this means that higher-dimensional operators are accompanied by dimensionful coupling constants. It is not possible to construct operators of dimension five that are Lorentz and gauge invariant, so the first additional operators are of dimension six. A general analysis of dimension six operators has been presented in [926]. The choice of the basis of these operators is, however, not unique, and especially for operators involving electroweak gauge bosons a number of different choices have been discussed in the literature; a common representation can be found in [928]. In the EFT approach one first specifies the particle content of the theory and derives the corresponding vertices and coupling constants from there. At a first glance the two approaches, i.e. the EFT and the effective Lagrangian approach, may lead to the same results, as one can express the coupling constants of Eqs. (126), (127), (130) as functions of the coefficients $f_{i}^{(n)}/{\Lambda }^n$ of Eq. (132) [928], as follows:

133 $$\begin{aligned}&g_1^Z = 1 + f_W\frac{m_Z^2}{2{\Lambda }^2}, \nonumber \\&\kappa _Z = 1 + \left[ f_W -\sin ^2 \theta _W(f_B +f_W)\right] \frac{m_Z^2}{2{\Lambda }^2},\nonumber \\&\kappa _{\gamma } = 1 +(f_B + f_W)\frac{m_W^2}{2{\Lambda }^2},\nonumber \\&\lambda _{\gamma } = \lambda _{Z} = \frac{3 m_W^2 g^2}{2{\Lambda } ^2} f_{WWW}. \end{aligned}$$

The corresponding Lagrangian using the EFT approach of Eq. (132) leading to Eq. (133) is given by [928]

134 $$\begin{aligned} {\mathscr {L}}_{eff}= & {} {\mathscr {L}}_{\mathrm{SM}} +\frac{f_B}{{\Lambda }^2}(D_{\mu }\phi )^{\dagger }\hat{B}^{\mu \nu }(D_{\nu } \phi ) \nonumber \\&+ \frac{f_W}{{\Lambda }^2}(D_{\mu }\phi )^{\dagger }\hat{W}^{\mu \nu }(D_{\nu } \phi ) \nonumber \\&+\frac{f_{WWW}}{{\Lambda }^2}\text {Tr}\left[ \hat{W}_{\mu \nu } \hat{W} ^{\nu \rho } \hat{W}_{\rho }^{\mu }\right] , \end{aligned}$$

with $\hat{B}^{\mu \nu }=i\frac{g'}{2}B^{\mu \nu }$ and $\hat{W}^{\mu \nu }=ig\frac{\sigma ^a}{2}W^{a,\mu \nu }$. However, the EFT approach offers a better interpretation of the origin of these additional couplings as we will describe in more detail next.

The scale ${\Lambda }$ denotes the energy scale at which the structure of the full theory is resolved. At lower energies, the heavy degrees of freedom of this full theory are considered to be integrated out, appearing as higher-dimensional operators in the EFT that describes the low-energy physics. One example for such an EFT is Fermi’s theory of weak interactions. At an energy scale well below the *W* boson mass the weak interaction of leptons and neutrinos can be described by a four-fermion operator of dimension six. The corresponding scale ${\Lambda }$ in an EFT description of weak interaction would then be the *W* boson mass. For energies well below the (usually unknown) scale ${\Lambda }$, the higher-dimensional operators are suppressed by powers of ${\Lambda }$. This ensures that the higher-dimensional operators are more suppressed than lower-dimensional operators, i.e. dimension eight operators can usually be neglected compared to dimension six operators. In the limit ${\Lambda } \rightarrow \infty$ one recovers the SM. The EFT is only valid at energies well below ${\Lambda }$. As soon as one approaches this scale the operators of dimension greater than six are no longer suppressed. They contribute equally and can no longer be neglected. At this point the EFT breaks down and has to be replaced by the UV completion of the underlying full theory. Therefore the EFT provides a handle on the energy range in which it is valid, which cannot be deduced from the effective Lagrangians of Eqs. (126), (127), (130).

One very important feature of higher-dimensional operators is their high-energy behaviour. Due to their higher dimension, the effects of these operators increase with energy and would eventually violate unitarity. The energy at which (tree-level) unitarity is violated depends on the operator and in general also depends on the helicity [929]. Typically this problem is solved by introducing form factors which suppress the effects of the operators hence rendering the cross section unitary. These form factors are, however, completely arbitrary as long as they preserve unitarity and from the viewpoint of an EFT they are not needed because at this energy the effective theory is no longer valid [930].

The effects of anomalous couplings in electroweak gauge boson interactions in the production of multiple gauge bosons have been calculated both for $e^{+} e^{-}$ colliders [931–934] as well as for hadron colliders [916, 935–941] and many available results also include next-to-leading order QCD and/or electroweak corrections. For the extraction of limits on anomalous TGCs and QGCs it is essential that precise predictions of the relevant processes are provided in the form of Monte Carlo programs including the effects of anomalous couplings. The implementation of anomalous couplings in publicly available Monte Carlo programs ranges from specific processes to a general implementation at the level of the Lagrangian. For $e^+e^-$ colliders anomalous couplings for the production of four fermions (and a photon) are contained in RacoonWW [942–944], including NLO EW corrections to four-fermion production in double-pole approximation. A broader implementation of anomalous couplings for $e^+e^-$ colliders is provided in WHIZARD [945, 946], which can also be used for hadron colliders. VBFNLO [947–949] provides NLO QCD predictions for processes at hadron colliders including tri-linear and quartic couplings as well as anomalous couplings of electroweak gauge bosons to the Higgs boson. CalcHEP and CompHEP [950–952] can import anomalous couplings from LanHEP [953–955] which generates them at the level of the Lagrangian. FeynRules also can generate anomalous couplings at the Lagrangian level and the corresponding Feynman rules can be implemented via the UFO format [956] to any Monte Carlo program that supports this format, as for instance MadGraph [957].

#### Anomalous gauge couplings: experimental prospects^56^

We briefly review the capabilities of an LC to measure triple and quartic gauge couplings (based on Ref. [269] and references therein). As mentioned earlier, the effects of higher-dimensional operators are suppressed at low energies and their impact increases with increasing centre-of-mass energy. Therefore a general pattern is the deviation from the SM best visible in the high-energy tails of distributions like $p_T$, $H_T$ or invariant masses.

The couplings among the electroweak gauge bosons are directly given by the structure of the gauge group, see the previous section. This structure can thus directly be determined by a measurement of the gauge boson interactions. Particularly sensitive is the process $e^+e^- \rightarrow W^+W^-$, since any “naive” change in the gauge couplings would lead to a violation of unitarity, and small changes lead to relatively large variations.

To date, EWPO together with the LEP data yielded the strongest constraints on anomalous couplings [958–960]. For the triple gauge couplings the bounds are [959, 960]

135 $$\begin{aligned}&{\varDelta } g_1^Z = -0.033 \pm 0.031,\nonumber \\&{\varDelta } \kappa _{\gamma } = 0.056 \pm 0.056,\nonumber \\&{\varDelta } \kappa _Z = -0.0019 \pm 0.044,\\&\lambda _{\gamma } = -0.036 \pm 0.034,\nonumber \\&\lambda _Z = 0.049 \pm 0.045 .\nonumber \end{aligned}$$

The bounds currently available from LHC data are weaker but approach the precision of the LEP results [961].

Turning to the ILC, the different types of couplings can be disentangled experimentally by analysing the production angle distribution of the *W* boson and the *W* polarisation structure, which can be obtained from the decay angle distributions. Anomalous couplings for $WW\gamma$ and *WWZ* result in similar final-state distributions. However, using beam polarisation, they can be disentangled, where a large beam polarisation, in particular for the left-handed $e^-$ is required. Also positron polarisation is required for an optimal resolution [45].

A fast detector simulation analysis was performed for $\sqrt{s} = 500\,\mathrm {GeV}\,$ and $800\,\mathrm {GeV}\,$ [962]. The results for single parameter fits are shown in Table 29. Correlations in the multi-parameter fits were taken into account where possible. For $\sqrt{s} = 800\,\mathrm {GeV}\,$ they are relatively small, not increasing the uncertainties by more than $\sim$20 %. At $\sqrt{s} = 500\,\mathrm {GeV}\,$ the effect is larger, and uncertainties can increase by up to a factor of 2, see also Ref. [7].

**Table 29:** Results of the single parameter fits ($1 \sigma$) to the different triple gauge couplings at the ILC for $\sqrt{s}=500$ GeV with ${\mathscr {L}}= 500$ fb$^{-1}$ and $\sqrt{s}=800$ GeV with ${\mathscr {L}}=1000$ fb$^{-1}$; ${\mathscr {P}}_{e^-} = 80\,\%$ and ${\mathscr {P}}_{e^+} = 60\,\%$ has been used. Taken from [962]

Coupling	Error $\times 10^{-4}$	
	$\sqrt{s}=500\,\mathrm {GeV}\,$	$\sqrt{s}=800\,\mathrm {GeV}\,$
${\varDelta } g^{Z}_1$	15.5	12.6
${\varDelta } \kappa _{\gamma }$	3.3	1.9
$\lambda _{\gamma }$	5.9	3.3
${\varDelta } \kappa _{Z}$	3.2	1.9
$\lambda _{{Z}}$	6.7	3.0
$g^Z_{5}$	16.5	14.4
$g^Z_{4}$	45.9	18.3
$\tilde{\kappa }_{Z}$	39.0	14.3
$\tilde{\lambda }_{Z}$	7.5	3.0

**Fig. 119:**
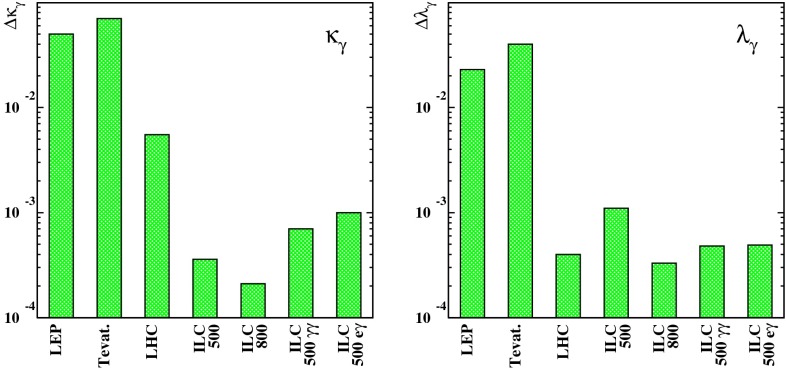
Comparison of ${\varDelta } \kappa _\gamma$ and ${\varDelta } \lambda _\gamma$ at different machines. For LHC and ILC 3 years of running are assumed (LHC: 300 fb$^{-1}$, ILC $\sqrt{s}=500$ GeV: 500 fb$^{-1}$, ILC $\sqrt{s}=800$ GeV: 1000 fb$^{-1}$). If available the results from multi-parameter fits have been used. Taken from [269]

Additional information on the triple gauge couplings can be obtained when going to the $e\gamma$ and $\gamma \gamma$ options at the ILC. In this environment the $WW\gamma$ couplings can be measured without the *WWZ* couplings entering the analysis. It was shown [963, 964] that $\kappa _{\gamma }$ can be measured better in $e^+e^-$ collisions, while for $\lambda _\gamma$ the $e\gamma$ and $\gamma \gamma$ modes can add relevant information. Figure 119 shows the results for $\kappa _\gamma$ and $\lambda _\gamma$ obtained at different machines. The measurement of $\kappa _\gamma$ can be improved substantially at the ILC. The other coupling, $\lambda _\gamma$, on the other hand can be measured with similar accuracy at the LHC and the various ILC options.

Apart from the triple electroweak gauge boson couplings, the ILC is also sensitive to the quartic couplings. Two processes are important in this context: $e^+e^- \rightarrow VVV$ (triple gauge boson production, $V = W^\pm , Z$) and $e^+e^- \rightarrow VV' l_1 l_2$ ($l_{1,2} = e, \nu$, $V = W^\pm , Z$), see Ref. [915] and references therein. This study uses complete six-fermion matrix elements in unweighted event samples, fast simulation of the ILC detector and a multidimensional parameter fit of the set of anomalous couplings. It also includes a study of triple weak boson production which is sensitive to the same set of anomalous couplings. It was shown that, under the assumption of custodial symmetry, sensitivities for $h^{ZZ}$ and $g_2^{WW}$ at and below the level of $\sim$5 % can be found [915] for $\sqrt{s} = 1$ TeV and 1 ab$^{-1}$ (see also [269]).

As mentioned earlier, apart from the investigation of diboson and triple gauge boson production processes, constraints on the coefficients of higher-dimensional operators that lead to new tri-linear gauge couplings can also be obtained from their contributions to EWPOs. For instance, modifications of gauge boson self energies induced by these higher-dimensional operators can be described with the help of *S*, *T* and *U* parameters [820, 965] and their extensions [966], and by precisely measuring these oblique parameters the effects of these operators can be severely constrained [928, 967, 968]. Typically, bounds from EWPOs mainly affect those operators that contribute already at tree level to the observables. The effects of operators contributing only at the one loop level are suppressed and therefore their bounds are weaker compared to the bounds that can be derived from direct measurements [967, 968].

Recently, constraints on anomalous quartic gauge couplings have been obtained from studies of $WW\gamma$ and $WZ\gamma$ production [969] and like-sign *WWjj* production [970] at the 8 TeV LHC.

### New gauge bosons^57^

Extra gauge bosons, $Z'$s and $W'$s, are a feature of many models of physics beyond the SM [572, 971–974]. Examples of such models are Grand Unified theories based on groups such as *SO*(10) or $E_6$ [974], Left–Right symmetric models [975], Little Higgs models [506, 509, 534, 976], and Technicolour models [977–980] to name a few. In addition, resonances that arise as Kaluza–Klein excitations in theories of finite size extra dimensions [981] would also appear as new gauge bosons in high energy experiments. It is therefore quite possible that the discovery of a new gauge boson could be one of the first pieces of evidence for physics beyond the SM. Depending on the model, the dominant $Z'$ decay may be either into leptons or jets, leading to a resonance in the reconstructed dilepton or dijet invariant mass distribution, respectively.

Currently, the highest mass bounds on most extra neutral gauge bosons are obtained by searches at the large hadron collider by the ATLAS and CMS experiments. The most recent results based on dilepton resonance searches in $\mu ^+\mu ^-$ and $e^+e^-$ final states use data from the 7 TeV proton collisions collected in 2011 and more recent 8 TeV data collected in 2012. ATLAS [982] obtains the exclusion limits at 95 % CL $M(Z^\prime _\mathrm{SSM})>2.49$ TeV, $M(Z^\prime _\eta )>2.15$ TeV, $M(Z^\prime _\chi )>2.24$ TeV and $M(Z^\prime _\psi )> 2.09$ TeV using only the 8 TeV (6 fb$^{-1}$) dataset and CMS [983] obtains 95 % CL exclusion limits of $M(Z^\prime _\mathrm{SSM})>2.59$ TeV and $M(Z^\prime _\psi )> 2.26$ TeV using the 7 TeV (5 fb$^{-1}$) and 8 TeV (4 fb$^{-1}$) datasets. It is expected that the LHC should be able to see evidence for $Z'$s up to $\sim$5 TeV once the LHC reaches its design energy and luminosity [984–988] and to distinguish between models up to $M_{Z'}\simeq 2.1$ TeV (95 % CL) [989].

It is expected that the LHC will be able to discover $W'$s up to masses of $\sim$5.9 TeV in leptonic final states assuming SM couplings [985]. Based on searches for a new *W* boson decaying to a charged lepton and a neutrino using the transverse mass variable CMS [990] excludes the existence of a SSM $W'$ boson with a mass below 2.85 TeV at 95 % CL using the $\sqrt{s}=8$ TeV, ${\mathscr {L}}_\mathrm{int}=3.7$ fb$^{-1}$ dataset while ATLAS excludes the existence of a $W^*$ with a mass below 2.55 TeV at 95 % CL using the 7 TeV dataset with ${\mathscr {L}}_\mathrm{int}=4.7$ fb$^{-1}$ [991].

For models that predict $Z'$ or $W'$ bosons that decay to two quarks, searches have been performed that require two well-separated jets with high transverse momentum. The CMS Collaboration excludes the existence of a SSM $Z'$ boson with mass below 1.6 TeV at 95 % CL and a SSM $W'$ with mass below 2.12 TeV using the $\sqrt{s}=8$ TeV, ${\mathscr {L}}_\mathrm{int}=4.0$ fb$^{-1}$ dataset [992]. The CMS Collaboration also developed a dedicated search for $b\bar{b}$ resonances and excluded existence of a SSM $Z'$ boson with mass below 1.5 TeV at 95 % CL in the $b\bar{b}$ channel [993]. For models with larger branching fractions to *b*-quarks the limit improves considerably, excluding a larger mass range.

If a narrow resonance were discovered, the crucial next step would be to measure its properties and determine the underlying theory. While LHC measurements [971, 994] and low-energy precision measurements [995] can to some extent constrain new gauge boson couplings, precise measurements will need a LC.

#### New gauge boson studies at high-energy $\varvec{e^+ e^-}$ colliders

Although the LHC will have explored the energy regime accessible to on-shell $Z'$ production by the time a LC is built, a high-energy $e^+e^-$ collider will be sensitive to new gauge bosons with $M_{Z' , W'} \gg \sqrt{s}$. In $e^+e^-$ collisions below the on-shell production threshold, extra gauge bosons manifest themselves as deviations from SM predictions due to interference between the new physics and the SM $\gamma /Z^0$ contributions. $e^+e^-\rightarrow f\bar{f}$ reactions are characterised by relatively clean, simple final states where *f* could be leptons (*e*, $\mu$, $\tau$) or quarks (*u*, *d*, *s*, *c*, *b*, *t*), for both polarised and unpolarised $e^\pm$. The baseline ILC configuration envisages electron beam polarisation greater than 80 % and positron beam polarisation of $\sim$30 % might be initially achieved, eventually increasing to $\sim$60 %. The basic $e^+e^-\rightarrow f\bar{f}$ processes can be parametrised in terms of four helicity amplitudes which can be determined by measuring various observables: the leptonic cross section, $\sigma (e^+e^-\rightarrow \mu ^+\mu ^-)$, the ratio of the hadronic to the QED point cross section $R^\mathrm{had}=\sigma ^\mathrm{had}/\sigma _0$, the leptonic forward–backward asymmetry, $A^\ell _\mathrm{FB}$, the leptonic longitudinal asymmetry, $A^\ell _\mathrm{LR}$, the hadronic longitudinal asymmetry, $A^\mathrm{had}_\mathrm{LR}$, the forward–backward asymmetry for specific quark or lepton flavours, $A^f_\mathrm{FB}$, the $\tau$ polarisation asymmetry, $A_{\mathrm{pol}}^\tau$, and the polarised forward–backward asymmetry for specific fermion flavours, $A^f_\mathrm{FB}(\mathrm{pol})$ [996] (see also Sect. 4.3). The indices $f=\ell , \; q$, $\ell =(e,\mu ,\tau )$, $q=(c, \; b)$, and $\mathrm{had}=$ ‘sum over all hadrons’ indicate the final-state fermions. Precision measurements of these observables for various final states ($\mu ^+\mu ^-$, $b\bar{b}$, $t\bar{t}$) can be sensitive to extra gauge boson masses that by far exceed the direct search limits that are expected at the LHC [984, 986, 996, 997]. Further, precision measurements of cross sections to different final state fermions using polarised beams can be used to constrain the gauge boson couplings and help distinguish the underlying theory [9, 10, 997–1002]. A deviation for one observable is always possible as a statistical fluctuation. In addition, different observables have different sensitivities to different models (or more accurately to different couplings). As a consequence, a more robust strategy is to combine many observables to obtain a $\chi ^2$ figure of merit.

**Fig. 120:**
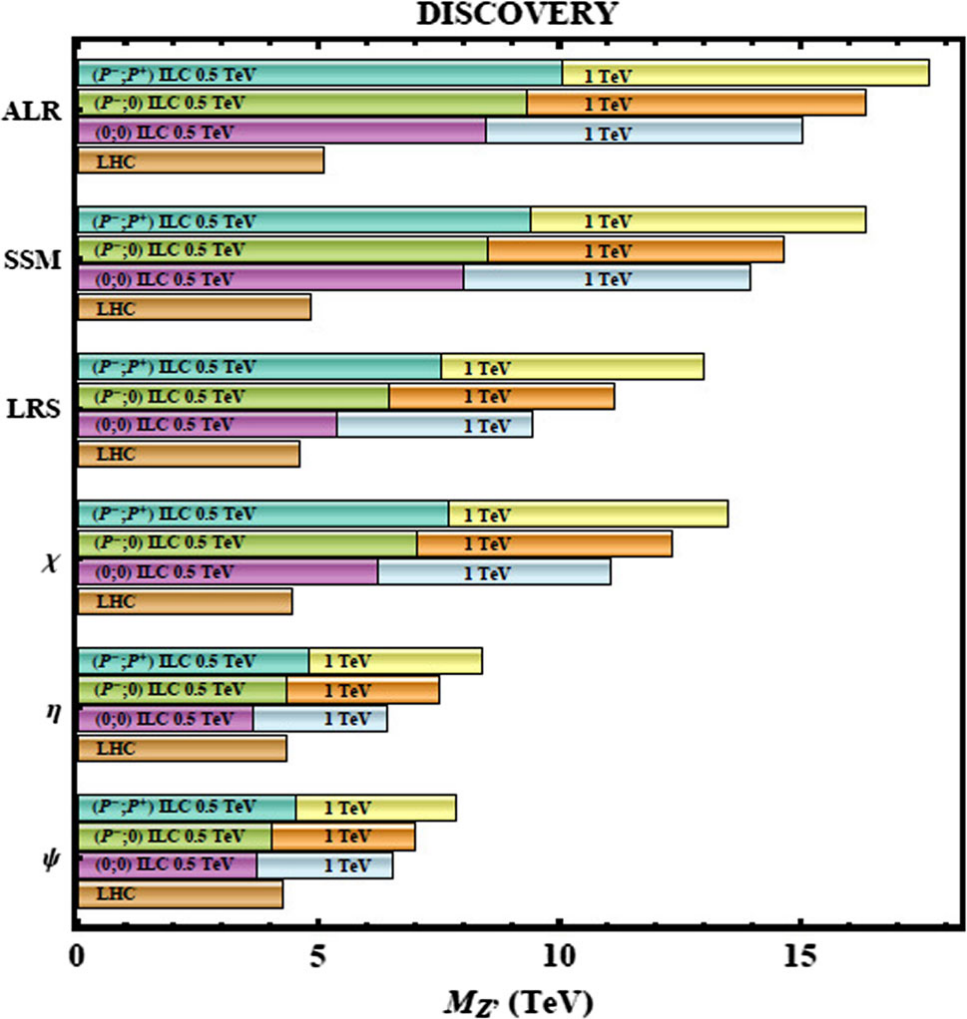
Discovery reach of the ILC with $\sqrt{s}=0.5$ (1.0) TeV and ${\mathscr {L}}_{\mathrm{int}}=500$ (1000) fb$^{-1}$. The discovery reach of the LHC for $\sqrt{s}=14$ TeV and 100 fb$^{-1}$ via the Drell–Yan process $pp\rightarrow \ell ^+\ell ^- +X$ are shown for comparison. From Ref. [997] with kind permission of The European Physical Journal (EPJ)

The ILC sensitivity to $Z'$s is based on high statistics precision cross section measurements so that the reach will depend on the integrated luminosity. For many models a 500 GeV $e^+e^-$ collider with as little as 50 fb$^{-1}$ integrated luminosity would see the effects of a $Z'$ with masses as high as $\sim 5$ TeV [984]. The results of a recent study [997] is shown in Fig. 120. That study finds that a 500 GeV ILC with 500 fb$^{-1}$ and a 1 TeV ILC with 1 ab$^{-1}$ can see evidence or rule out a $Z'$ with masses that can exceed $\sim$7 and $\sim$12 TeV for many models, for the two respective energies [997]. These recent results also consider various polarisations for the $e^-$ and $e^+$ beams and show that beam polarisation will increase the potential reach of the ILC, see also Ref. [45].

**Fig. 121:**
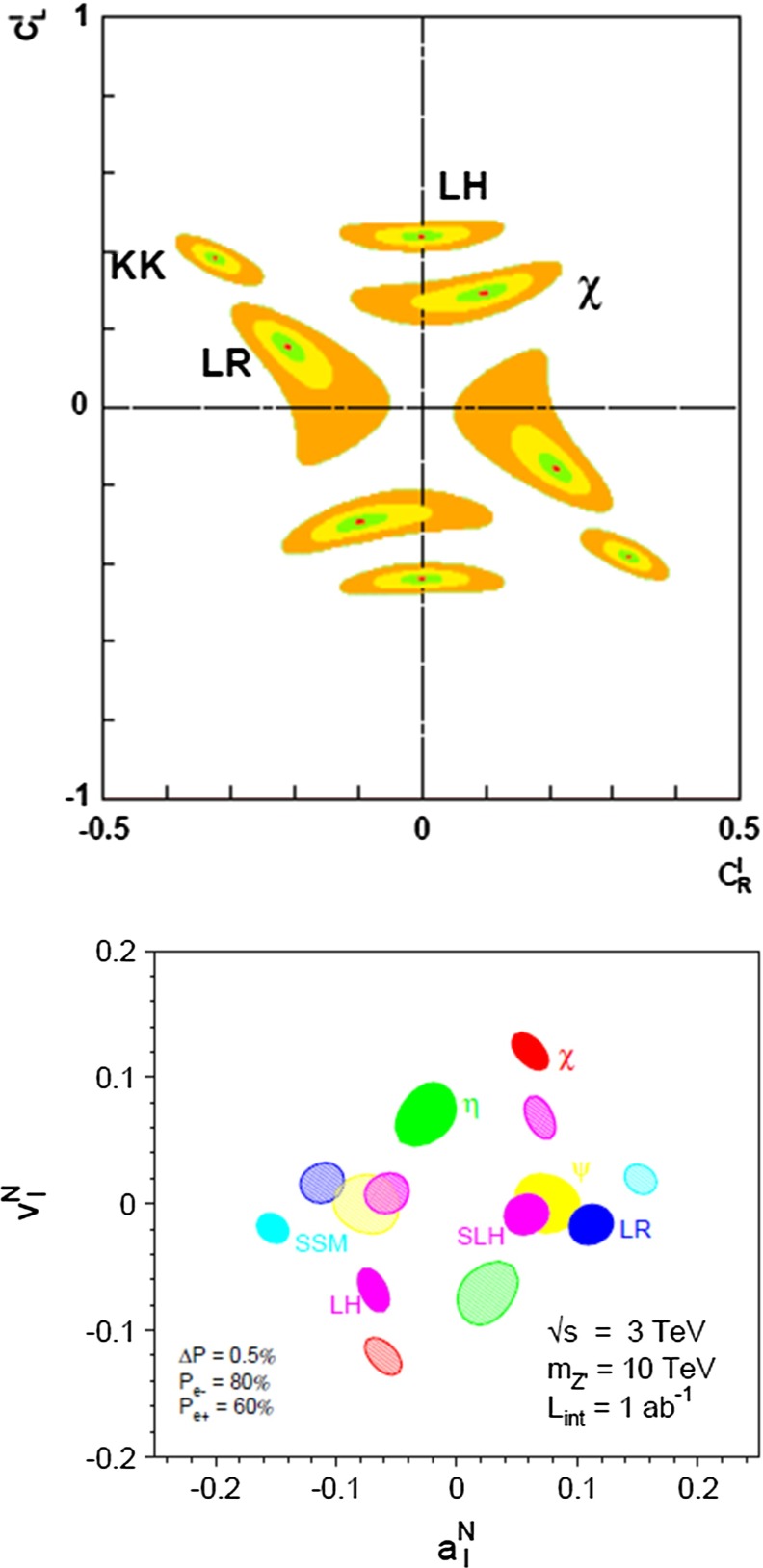
*Top* Resolving power (95 % CL) for $M_{Z'}=1,1.5,$ and 2 TeV and $\sqrt{s}=500$ GeV, ${\mathscr {L}}_\mathrm{int}=1$ ab$^{-1}$, $|P_{e^-}|=80$ %, $|P_{e^+}|=60$ %, for leptonic couplings based on the leptonic observables $\sigma$, $A_\mathrm{LR}$, $A_\mathrm{FB}$. The couplings correspond to the $E_6$ $\chi$, LR, LH, and KK models. From Ref. [1000]. *Bottom* Expected resolution at CLIC with $\sqrt{s}=3$ TeV and ${\mathscr {L}}= 1$ ab$^{-1}$ on the “normalised” leptonic couplings of a 10 TeV $Z'$ in various models, assuming lepton universality. The mass of the $Z'$ is assumed to be unknown. The couplings correspond to the $E_6$ $\chi$, $\eta$, and $\psi$, the SSM, LR, LH and SLH models. The couplings can only be determined up to a two-fold ambiguity. The degeneracy between the $\psi$ and SLH models might be lifted by including other channels in the analysis ($t\bar{t}$, $b\bar{b}$,...). From Refs. [9, 10, 1001]

#### Measurement of $\varvec{Z'}$ couplings at high-energy $\varvec{e^+ e^-}$ colliders

If a $Z'$ were discovered at the LHC, measurements of 2-fermion processes at the ILC could provide valuable constraints on its couplings and discriminate between models. Figure 121 (top panel) shows the expected resulting precision on $Z'$ couplings to leptons for $\sqrt{s}=500$ GeV and ${\mathscr {L}}_\mathrm{int}=1$ ab$^{-1}$ for 3 values of $M_{Z'}$ for several representative models [1000]. In this figure, the KK case should not be taken too literally as the couplings do not in fact correspond to the KK $Z'$ couplings but are an effective coupling, reflecting that in this model there are both photon and *Z* KK excitations roughly degenerate in mass. The point is simply that the KK model can be distinguished from other models. One notes that there is a two-fold ambiguity in the signs of the lepton couplings since all lepton observables are bi-linear products of the couplings. Hadronic observables can be used to resolve this ambiguity since for this case the quark and lepton couplings enter the interference terms linearly. Studies [997, 1000] have demonstrated that beam polarisation plays an important role in the measurement of the $Z'$-fermion couplings and therefore in the discrimination between models.

Rather than measure the $Z'$-fermion couplings one could pose the question; if measurements resulted from a true BSM model, could one rule out other possibilities? A recent analysis given in Ref. [997] showed that the ILC could discriminate models for $Z'$ masses up to 4–8 TeV for a 500 GeV ILC and up to 6–11 TeV for a 1 TeV ILC, depending on the true model. This exceeds the corresponding discovery reach at the LHC and is only slightly lower than the discovery reach at the ILC due to the relatively large differences between angular distributions for $e^+e^- \rightarrow f\bar{f}$ for the different models. More crucially, the ILC is significantly more powerful for measuring $Z'$ couplings than is possible at the LHC. These results are based on purely leptonic processes. Measurements of *c*- and *b*-quark pair production cross sections would contribute important complementary information for identifying the underlying theory.

If deviations from the SM were observed but there was no direct evidence for a $Z'$ from the LHC one could still exclude a “tested” model for any value of $M_{Z'}$ below some value for a given set of ILC measurements. To see how one can extract such limits consider normalised couplings defined by $C_{v,a}^{fN}={C_{v,a}^{f'}}\sqrt{s/(M_{Z'}^2-s)}$. Figure 121 (bottom panel) shows contraints on “normalised” couplings for a 10 TeV $Z'$ and $\sqrt{s}=3$ TeV and ${\mathscr {L}}_\mathrm{int}=1$ ab$^{-1}$ [9, 10, 1001]. One can see how, if a model with a 10 TeV $Z'$ were the true model, other models could be excluded. Reference [997] finds that for the models they considered one might be able to distinguish between $Z'$ models, at 95 % CL, up to $M_{Z'}\simeq 3.1$ TeV (4.0 TeV) for unpolarised (polarised) beams at the 0.5 TeV ILC and 5.3 TeV (7.0 TeV) at the 1 TeV ILC. Presented another way, they find that if one of the six models they studied is true, the other five candidates can be ruled out by a 500 GeV ILC for $Z'$ masses up to 4–8 TeV, depending on the true model. This discrimination reach is only slightly below the discovery reach due to order-one differences among the angular distributions in $e^+e^- \rightarrow f\bar{f}$ predicted by the different models and in all cases is significantly higher than that of the LHC.

#### Discovery and identification of $\varvec{W'}$ bosons in $\varvec{e^+e^-}$

While there is a broad literature on $Z'$ properties, $W'$ studies for high-energy $e^+e^-$ colliders are rather limited. One study showed that the process $e^+ e^-\rightarrow \nu \bar{\nu } \gamma$ would be sensitive to $W'$ masses up to several TeV depending on the model, the centre-of-mass energy, and the assumed luminosity [1003]. For example, evidence for a SSM $W'$ could be seen up to $M_{W'}=4.3, 5.3,$ and 6.0 TeV for $\sqrt{s}=0.5,1.0$, and 1.5 TeV, respectively, with ${\mathscr {L}}_\mathrm{int}=500$ fb$^{-1}$, while a LR $W'$ could only be detected up to $M_{W'}=1.2, 1.6$, and 1.9 TeV for the same collider parameters. Another process that has been considered is $e\gamma \rightarrow \nu q +X$ where the photon is produced by a back-scattered laser or is a Weizsäcker–Williams photon [1004]. These processes yield discovery limits for $W'_\mathrm{SSM}$ of 4.1 (2.5), 5.8 (3.6) and 7.2 (4.5) TeV for the back-scattered laser (Weizsäcker–Williams) cases and for the three values for $\sqrt{s}$ and ${\mathscr {L}}_\mathrm{int}$ given above. Limits for the LR model are substantially lower.

In general we do not expect an $e^+e^-$ collider to be sensitive to $W'$s with masses larger than could be discovered at the LHC. If new gauge bosons were discovered first in other processes, the ILC could measure $W'$ (and $Z' \nu \bar{\nu }$) couplings which would complement measurements made at the LHC.

## Supersymmetry^58^

### Introduction and overview

The recent discovery of a Higgs-like resonance at $M_h=(125.15\pm 0.24)$ GeV by the Atlas and CMS experiments at the CERN LHC seemingly completes the identification of all matter states predicted to exist by the standard model of particle physics. In spite of this extraordinary achievement, the SM remains beset by an array of shortcomings which strongly suggest that new physics exists at, or around, the TeV energy scale. Chief among these is the gauge hierarchy problem, which arises if fundamental scalar fields (such as the Higgs field) do exist. In this case, the scalar field mass term diverges quadratically, and we would expect the Higgs field to have mass far beyond the 125 GeV level unless an exquisite degree of fine tuning between bare and loop corrections is invoked at each order in perturbation theory.

Along with the gauge hierarchy problem, the SM is lacking in that it provides no particle to explain cold dark matter (CDM) in the universe, it does not allow for baryogenesis in the early universe, it does not allow for the suggested unification of SM forces, it contains no solution to the strong ${\textit{CP}}$ problem and it provides no avenue for a sensible inclusion of quantum gravity into its structure.

While a variety of solutions to the gauge hierarchy problem have been proposed, weak-scale supersymmetry [1005–1009], or SUSY, is the most theoretically engaging and one which also appears to be, at least indirectly, supported by experimental data. Supersymmetry is a quantum space-time symmetry that predicts a correspondence between bosonic and fermionic degrees of freedom. In SUSY theories, scalar fields inherit the protective chiral symmetry enjoyed by fermions, reducing their quadratic divergence to merely logarithmic. Since the log of a large number can be small, the required tuning between bare mass and loop mass is greatly reduced, allowing disparate mass scales to coexist within the same theoretical structure.

To be phenomenologically viable, supersymmetrised versions of the SM must include *soft* SUSY breaking [1010], i.e. only those SUSY-breaking terms which maintain the cancellation of quadratic divergences. In the MSSM, a variety of new matter states – spin 0 squarks and sleptons along with additional Higgs bosons and spin $1\over 2$ charginos, neutralinos and gluinos – are expected to exist at or around the weak scale.

The MSSM has received some indirect experimental support from the measured values of the strong and electroweak forces: these unify to a single value at energy scales $M_{\mathrm{GUT}}\sim 2\times 10^{16}$ GeV under renormalisation group (RG) evolution. Also, the measured value of the top quark ($m_t\simeq 173.2$ GeV) turns out to be sufficiently large as to induce a radiatively driven breaking of electroweak symmetry. In addition, while the SM allows for a Higgs mass within a wide range, $100<M_H<1000$ GeV, the MSSM restricts the lightest SUSY Higgs boson $100<M_h<135$ GeV. The fact that the newly discovered Higgs-like state falls within the narrow mass range predicted by SUSY may also be regarded as an indirect support of this picture. Simple arguments based on electroweak naturalness would suggest that superpartners should exist at or below the $\sim 1$ TeV scale, motivating a significant effort for their search at the LHC and inspiring the physics programme of a future $e^+e^-$ linear collider. Finally, SUSY provides us with at least three viable candidates for DM: the lightest neutralino $\tilde{\chi }^0_1$ (a WIMP candidate) the gravitino $\tilde{G}$ and the axino $\tilde{a}$ (the spin-1/2 superpartner of the axion)[1011].

SUSY theories also offer at least three mechanisms for baryogenesis, including weak-scale baryogenesis (now nearly excluded in the MSSM), thermal and non-thermal leptogenesis and Affleck–Dine baryo- and leptogenesis [1012]. Local SUSY (supergravity) theories necessarily include spin-2 gravitons and spin-3/2 gravitinos, and reduce to Einstein’s general relativity in the classical limit.

This chapter provides an overview of the capabilities of a linear $e^+e^-$ collider in the search for supersymmetry, in view of the constraints and indications derived from present experimental data, in particular the LHC results from the 7 and 8 TeV data for the SUSY direct searches and the Higgs properties. The limits derived in these searches seem to require SUSY particles beyond the TeV scale, seemingly in contradiction to the aforementioned arguments based on electroweak naturalness. However, it is important to observe that the strongly interacting SUSY particles – which LHC is most sensitive to – are also those with less direct connection to the electroweak naturalness. Taken in this context, there remains a huge role for LHC operation at 13–14 TeV and for subsequent operation of a linear $e^+e^-$ collider of sufficient centre of mass energy, $\sqrt{s}$, to play a decisive role in the search for, and proof of, SUSY. Indeed, even if no SUSY particles are seen at the LHC at 13–14 TeV, then a 0.5–1 TeV linear $e^+e^-$-collider may still retain its role as *discovery* machine for SUSY [1013, 1014] in that *the most natural SUSY models require light higgsinos* with mass $\sim$100–200 GeV which can easily elude LHC searches (due to the small energy release from their compressed spectra), but which can easily be detected in $e^+e^-$ collisions of sufficient energy $\sqrt{s}>2m(\mathrm{higgsino})$.

If supersymmetric matter is indeed found at LHC or the $e^+e^-$-LC, then a programme of precision measurements, which can be made in high energy $e^+e^-$ collisions, will be crucial for pinning down SUSY particle masses, mixings and other properties. From such measurements, it may be possible to clarify the role of SUSY in cosmic DM production and possibly also in baryogenesis, thus establishing even more closely the link between particle physics and cosmology. If indeed a desert exists between the weak scale and some high scale such as $M_{\mathrm{GUT}}$ or $M_{\mathrm{string}}$, then it may be possible to extrapolate SUSY parameters to these ultra-high scales, thus testing ideas about unification, SUSY breaking, and string theory. We will conclude that a linear $e^+e^-$ collider of sufficient energy and luminosity is absolutely needed for providing a detailed experimental exploration of the intriguing concept of weak-scale supersymmetry, if it is realised in nature.

### Models of supersymmetry

The superfield formalism provides an algorithm for the direct supersymmetrisation of the SM [1015, 1016]. In this case, each SM matter fermion of a given chirality is elevated to a chiral superfield which also contains a spin-0 superpartner. The SM gauge fields are elevated to gauge superfields which also contain spin-$1\over 2$ gauginos. The SM Higgs doublet is embedded in a chiral superfield necessitating introduction of spin-$1\over 2$ higgsinos. The addition of extra higgsinos carrying gauge quantum numbers destroys the elegant anomaly cancellation mechanism in the SM, unless one introduces as well a second Higgs/higgsino doublet superfield carrying opposite weak hypercharge.

The resulting supersymmetrised SM enjoys exact, rigid supersymmetry – but this is known not to be true since it would imply e.g. the existence of spin-0 partners of the electron (selectrons) with the same mass as the electron: such matter states would easily have been detected long ago. Hence, SUSY must be a broken symmetry. SUSY can be broken explicitly by adding by hand *soft SUSY-breaking* (SSB) terms to the Lagrangian. These terms include mass terms for spin-0 superpartners, mass terms for each gaugino, and bi-linear and tri-linear scalar interactions (so-called *B* and *A* terms).

In addition, a plethora of terms are allowed in the superpotential which violate baryon- and lepton-number conservation, and lead to rapid proton decay. Such terms are suppressed by invoking an *R*-parity (which naturally arises in SUSY GUT theories based on *SO*(10)). If *R*-parity is conserved, then SUSY particles can only be produced in pairs at colliders, SUSY particles must decay to other SUSY particles, and the lightest SUSY particle must be absolutely stable, perhaps offering a good DM candidate.

The resulting theory, called the minimal supersymmetric standard model, or MSSM, is the direct supersymmetrisation of the SM that is consistent with all known constraints. It includes more than 100 adjustable parameters [1015], most of these consisting of flavour or ${\textit{CP}}$-violating terms. Under the assumption of minimal flavour violation (MFV) and minimal ${\textit{CP}}$-violation (MCPV), these are set to zero, so that FV and CPV arise solely from the Yukawa sector. The pMSSM model with 19 adjustable weak-scale parameters is a popular model for this approach.

#### Gravity mediation

An appealing approach to SUSY breaking comes from invoking *local* SUSY, or supergravity (SUGRA). If SUSY is local, then one must necessarily include a graviton–gravitino supermultiplet. One may include a so-called *hidden sector* of fields whose sole purpose is to allow for spontaneous breaking of SUSY via the superHiggs mechanism [1017]. Under the superHiggs mechanism, hidden sector fields acquire a SUSY-breaking VEV $\langle F\rangle \sim m^2$ so that the gravitino gains a mass $m_{3/2}\sim m^2/M_P$, while the graviton remains massless: if $m_{3/2}\sim M_{\mathrm{weak}}$, then $m\sim 10^{11}$ GeV.

The above-mentioned soft SUSY-breaking terms arise via tree-level gravitational interactions with magnitude $\sim m_{3/2}$. More generally, “gravity-mediated supersymmetry breaking” denotes any theory in which supersymmetry breaking is communicated to the visible sector by $M_P$-suppressed interactions at the tree level, not necessarily just involving the gravitational multiplet, and therefore gives soft parameters of the order $m_{3/2}$. If $m_{3/2}\sim M_{\mathrm{weak}}$, then in the limit $M_P\rightarrow \infty$, while keeping $m_{3/2}$ constant we obtain a theory with weak-scale rigid supersymmetry plus soft SUSY-breaking terms.

The minimal supergravity model (mSUGRA [1018] or CMSSM [1019]) assumes all matter scalars and both Higgs fields receive a common soft mass $m_0$ at some high scale, usually taken to be $M_{\mathrm{GUT}}\simeq 2\times 10^{16}$ GeV, the scale where gauge couplings unify in the MSSM. Likewise, all gauginos receive a common mass $m_{1/2}$, and all *A* terms are set to a common value $A_0$. While this ansatz is simple, and receives some experimental motivation in that such choices suppress flavour and ${\textit{CP}}$-violating terms, one must remember that it is at best merely a simplifying assumption that is not likely to remain true for realistic models [1020].

One of the virtues of SUSY models defined at a high scale such as $Q=M_{\mathrm{GUT}}$ is that the large top quark Yukawa coupling drives exactly the right scalar Higgs field $m_{H_u}^2$ to negative squared values, resulting in a radiatively driven breakdown of electroweak symmetry (REWSB) [1021]. Upon EWSB, the $B\mu$ parameter may be traded for a parameter $\tan \beta =v_u/v_d$, the ratio of Higgs field VEVs, and the magnitude of the Higgsino mass parameter $\mu$ is fixed to yield the measured *Z*-boson mass. Then all sparticle masses and mixings, and hence production and decay rates, are determined by the well-known parameter set: $m_0,\ m_{1/2},\ A_0,\ \tan \beta ,\ \ \mathrm{and}\ sign(\mu ).$ However, many more parameters are allowed if one deviates from the simplistic assumption listed above, resulting in models with *non-universal* soft SUSY-breaking terms.

#### GMSB and AMSB

In addition to models of gravity-mediated SUSY breaking, other possibilities exist. One of these is *gauge-mediated SUSY breaking*, or GMSB [1022, 1023]. In this class of theories, the hidden sector couples to a messenger sector (which carries SM gauge quantum numbers) which acts as an intermediary between the visible and hidden sectors. In GMSB, loop diagrams containing messenger states induce visible sector soft SUSY-breaking terms.

The gravitino again gets a mass $m_{3/2}\sim \langle F\rangle /M_P$, while the sparticles gain soft masses of the order $\frac{g^2}{16\pi ^2}\frac{F}{M}$, where *M* is the messenger mass and *g* is any MSSM gauge coupling. For $M\ll M_P$, the SUSY particles may still be at the TeV scale, while gravitinos can be much lighter, so that the gravitino may play the role of the LSP. In the simplest GMSB models, the tri-linear SSB terms are suppressed, so there is little mixing in the top squark sector. Thus, these models have trouble generating a light Higgs scalar of mass $\sim$125 GeV as is now required by data [1024, 1025]. More general gauge mediation models [1026] are now required for phenomenological viability.

A third possibility is *anomaly-mediated SUSY breaking* [1027, 1028]. In any model of SUSY-breaking mediation, there are contributions to SSB terms arising from the super-Weyl anomaly. These are, however, suppressed by a loop factor with respect to $m_{3/2}$ and therefore subdominant in gravity mediation or GMSB. They become relevant in *sequestered models* where the gravity- and gauge-mediated soft masses are negligible, e.g. because the hidden sector is spatially separated from the visible sector in extra dimensions.

In AMSB, the SSB terms are governed by the RG beta functions and anomalous dimensions divided by loop factors. In this case, the wino-like neutralino turns out to be LSP, while $m_{3/2}\sim 25\text {--}50$ TeV, thus solving the cosmological gravitino problem. Since minimal versions of these models fail to generate a large *A*-term, they also seem disfavoured by the recently measured Higgs boson mass. Moreover, the minimal anomaly-mediated model predicts tachyonic sleptons, which is an even more serious shortcoming. However, various string-inspired modifications of the minimal framework do lead to viable phenomenology [1029–1032].

#### Hybrid mediation schemes

Embedding the MSSM into a more fundamental model at high scales, for instance into the EFT of some superstring compactification, can naturally lead to hybrid mediation scenarios. These are attractive also from the phenomenological point of view.

An example, motivated from both heterotic and type IIB string models, is *mirage mediation* [1033–1035]: if gravity-mediated contributions to the gaugino masses are only mildly suppressed, they may be of similar magnitude as the anomaly-mediated contributions. A combination of gravity and anomaly mediation allows one to interpolate between unified gaugino masses at the GUT scale (as predicted by the simplest gravity-mediated GUT models) and unified gaugino masses at some arbitrary lower *mirage scale* (after adding the anomaly-mediated contributions, since these are given by the very same beta function coefficients that govern the gaugino mass RGEs). An immediate consequence is a compressed low-scale gaugino mass spectrum if the mirage scale is low [1036–1039]. This allows for a lower gluino mass without conflicting with the LHC search bounds, thus possibly reducing the fine tuning. Depending on the underlying model, a “natural SUSY” pattern for the squark masses, with sub-TeV stops but multi-TeV first- and second-generation squarks, may also be realised [1037, 1040]. Sub-TeV charginos and neutralinos are common in these models. Such models, realised within the MSSM, do have problems generating a light Higgs scalar with $M_h\simeq 125$ GeV [1041], while maintaining naturalness [1042].

A more extreme example is the case where the gravity-mediated contributions to the gaugino masses vanish altogether, e.g. because they are forbidden by some symmetry under which the goldstino superfield is charged [1028]. In this case (which suffers from extreme fine tuning with regards to EWSB) the squarks and sleptons have gravity-mediated masses up to around 100 TeV, while the gaugino masses follow the anomaly mediation pattern and are lighter by a loop factor [1043–1047]. The LSP is a wino-like neutralino which is nearly degenerate with a wino-like chargino.

Alternatively, for a high messenger scale just below the scale of grand unification (which is well motivated within certain F-theory and heterotic models [1048, 1049]), gauge mediation can coexist with gravity mediation. This is because the GUT scale is about a loop factor below the Planck scale. Generic models of high-scale gauge mediation tend to have problems with flavour constraints [1050, 1051], which should be solved similarly as in ordinary gravity mediation. Such hybrid gauge-gravity mediation models naturally allow one to obtain near-degenerate higgsino-like charginos and neutralinos with masses around the electroweak scale, while the rest of the spectrum can be in the multi-TeV range [1049, 1052]. Models with mixed gauge, gravity and anomaly mediation are also a possibility [1053].

All the above hybrid mediation scenarios have in common that the coloured superpartners may be difficult to see at the LHC, either because they are heavy or because the spectrum is compressed. In particular, large parameter space regions survive the constraints from LHC8. At the same time, at least some of the charginos and neutralinos are often light enough to be produced, detected, and studied at a linear $e^+e^-$ collider.

### Naturalness and fine tuning

The main reason we expect supersymmetric matter states to arise with masses around the electroweak scale derives from the notion of electroweak naturalness. A model is considered to be natural in the electroweak sector if there are no large, unnatural cancellations (fine tunings) required in deriving the measured values of both $M_Z$ and $M_h$.

A quantitative measure of fine tuning of a supersymmetric model was introduced over 25 years ago, while SUSY was being searched for at LEP [1054–1056]). The so-called *Barbieri–Giudice* measure, ${\varDelta }_{\mathrm{BG}}$, is defined as

136 $$\begin{aligned} {\varDelta }_{\mathrm{BG}}\equiv max_i\left[ c_i\right] \quad \mathrm{where}\quad c_i=\left| \frac{\partial \ln M_Z^2}{\partial \ln a_i}\right| =\left| \frac{a_i}{M_Z^2}\frac{\partial M_Z^2}{\partial a_i}\right| \end{aligned}$$

where the set $a_i$ constitute the fundamental parameters of the model. Thus, ${\varDelta }_{\mathrm{BG}}$ measures the fractional change in $M_Z^2$ due to fractional variation in model parameters $a_i$. The $c_i$ are known as *sensitivity coefficients* [1057].

For models with parameters defined at very high scales (e.g. at ${\Lambda }=M_{\mathrm{GUT}}$), as those discussed above, the evaluation of ${\varDelta }_{\mathrm{BG}}$ requires one to express $M_Z^2$ in terms of high-scale parameters using semianalytic solutions of the renormalisation group equations for the corresponding soft term and $\mu$ [1057–1059].

The ${\varDelta }_{\mathrm{BG}}$ measure picks off the coefficients of the various terms and recales by the soft term squared over the *Z*-mass squared: e.g. $c_{M_{Q_3}^2}=0.73\cdot (M_{Q_3}^2/M_Z^2)$. For example, if one allows $M_{Q_3}\sim 3$ TeV (in accord with requirements from the measured value of $M_h$) the result is $c_{M_{Q_3}^2}\sim 800$ and so ${\varDelta }_{\mathrm{BG}}\ge 800$. In this case, one expects SUSY would be electroweak fine tuned to about 0.1 %. However, in constrained SUSY models where the high scale parameters are related, then cancellations between positive and negative contributions can occur. For instance, in models with universal scalar masses, then third-generation fine tuning is greatly reduced in the focus point region. More generally, in models of gravity-mediated SUSY breaking, then for any hypothesised hidden sector, the SUSY soft-breaking terms are all calculated as numerical coefficients times the gravitino mass $m_{3/2}$ [1060].

These shortcomings can be cured by modifying the definition of the fine-tuning measure. In the calculation of the SUSY mass spectrum, the actual fine tuning occurs when enforcing the *electroweak minimisation condition* which is written as

137 $$\begin{aligned} \frac{M_Z^2}{2} = \frac{M_{H_d}^2+\Sigma _d^d - (M_{H_u}^2+\Sigma _u^u) \tan ^2\beta }{\tan ^2\beta -1} -\mu ^2. \end{aligned}$$

In the above expression, $M_{H_u}^2$ and $M_{H_d}^2$ are weak-scale soft SUSY-breaking masses, while the terms $\Sigma _d^d$ and $\Sigma _u^u$ incorporate a variety of radiative corrections (a complete list of one-loop corrections is provided in Ref. [1061].)

For typical SUSY models with parameters defined at some high scale ${\Lambda }$ (where ${\Lambda }$ is frequently taken as high as $M_{\mathrm{GUT}}\simeq 2\times 10^{16}$ GeV), the positive value of $M_{H_u}^2({\Lambda })$ is driven radiatively to negative values at the weak scale (owing to the large top quark Yukawa coupling) so that electroweak symmety is radiatively broken. In models where large TeV-scale values of $-M_{H_u}^2$ are generated at the weak scale, then a compensating value of $\mu ^2$ must be dialed/tuned to enforce the measured value of $M_Z\simeq 91.2$ GeV.

The amount of fine tuning required in Eq. 137 can be quantified by defining the *electroweak fine tuning measure*[1061–1063]

138 $$\begin{aligned} {\varDelta }_{\mathrm{EW}} \equiv \max _i \left| C_i\right| /(M_Z^2/2), \end{aligned}$$

where $C_{H_d}=M_{H_d}^2/(\tan ^2\beta -1)$, $C_{H_u}=-M_{H_u}^2\tan ^2\beta /(\tan ^2\beta -1)$ and $C_\mu =-\mu ^2$. Also, $C_{\Sigma _u^u(k)} =-\Sigma _u^u(k)\tan ^2\beta /(\tan ^2\beta -1)$ and $C_{\Sigma _d^d(k)}=\Sigma _d^d(k)/(\tan ^2\beta -1)$, where *k* labels the various loop contributions included in Eq. 137.

Since ${\varDelta }_{\mathrm{EW}}$ depends only upon the weak-scale SUSY spectrum, it is *model-independent* (within the MSSM) in that different models giving rise to exactly the same spectrum will have the same values of ${\varDelta }_{\mathrm{EW}}$. For models with parameters defined at the weak scale, such as the pMSSM, then ${\varDelta }_{\mathrm{BG}}\approx {\varDelta }_{\mathrm{EW}}$ since the sensitivity coefficients $c_{\mu }=C_{\mu }$ and $c_{H_u}=C_{H_u}$.

For $\tan \beta \mathop {\sim }\limits ^{>}5$ and neglecting radiative corrections, the condition Eq. 137 reduces to $M_Z^2/2\simeq -M_{H_u}^2-\mu ^2$, so that models with weak-scale naturalness require that $-M_{H_u}^2\sim M_Z^2$ and also $\mu ^2\sim M_Z^2$. The first of these conditions obtains crisis when $M_{H_u}^2$ is driven to small rather than large negative values during the process of radiative EWSB. The second condition implies a spectrum of light higgsino-like “electroweakinos” (i.e. charginos and neutralinos) with mass the closer to $M_Z$ the better:

- $$m_{\tilde{\chi }^{\pm }_1}, m_{\tilde{\chi }^0_{1,2}} \sim |\mu |\sim $$ 100–250 GeV.

Such light higgsinos would be accessible at an $e^+e^-$ linear collider of centre-of-mass energy, $\sqrt{s}=250$-500 GeV, i.e. exceeding twice their mass. In such a case, then a high-energy $e^+e^-$ collider would function as a *higgsino factory* [1064] in addition to a Higgs factory! While such light higgsinos might be produced at some sizeable rates at the LHC, the kinematics of their visible decay products may make it difficult if not impossible to observe them in hadronic collisions. The compressed spectra reduce the transverse momentum of the produced jets and leptons bringing them below the cuts applied by the triggers and the subsequent offline event selection criteria.

### Indirect constraints

In spite of the many attractive features of SUSY models, no sign of supersymmetric matter has yet emerged and DM is still to be observed at ground-based direct detection experiments. Here, we review the constraints on SUSY particle masses and parameters derived from precision measurements of low-energy processes and the DM relic density. Constraints from the direct search for SUSY particles at the LHC will be addressed in the following section.

#### Flavour physics

Flavour physics provides indirect information as regards supersymmetry which can play an important and complementary role compared to direct searches at colliders. Several decays of *b* hadrons which are suppressed in the SM may offer sensitivity to SUSY through additional contributions mediated by supersymmetric particles, which do not suffer the same suppression and may substantially modify the decay rate. The main processes of interest are the $\bar{B} \rightarrow X_s \gamma$, $B_s \rightarrow \mu ^+\mu ^-$ and $B_u\rightarrow \tau \nu _\tau$ decays.

The decay $\bar{B} \rightarrow X_s \gamma$ is a loop-induced flavour changing neutral current (FCNC) process that offers high sensitivity to supersymmetry due to the fact that additional contributions to the decay rate – in which SM particles are replaced by SUSY particles such as charged Higgs, charginos or top squarks – are not suppressed by a loop factor relative to the SM contribution. Within a global effort, a perturbative QCD calculation to the NNLL level has been performed [1065], leading to [1066]:

139 $$\begin{aligned} {\mathrm {BR}}(\bar{B} \rightarrow X_s \gamma )_\mathrm{NNLL} = (3.08 \pm 0.23) \times 10^{-4}, \end{aligned}$$

for a photon energy cut at $E_\gamma = 1.6$ GeV, and using the updated input parameters of PDG [821]. The non-perturbative corrections to this decay mode are sub-leading [1067] and their error is included in the above prediction. The averaged experimental value by the HFAG group [1068] gives

140 $$\begin{aligned} {\mathrm {BR}}(\bar{B} \rightarrow X_s \gamma )_\mathrm{exp} = (3.43 \pm 0.21 \pm 0.07) \times 10^{-4}, \end{aligned}$$

where the first error is the combined statistical and systematic uncertainties and the second represents the photon energy extrapolation. The SM prediction and the experimental average are hence consistent at the 1.2$\sigma$ level, and therefore this decay has a restrictive power on the SUSY parameter space. Recently, the first practically complete NLL calculation of the decay rate in the MSSM has been finalised [1069]. The dominant SUSY contributions are provided by diagrams with top squarks and charginos, which grow linearly with $\tan \beta$ [1070]. This decay is therefore particularly constraining in the regions with large $\tan \beta$ or spectra with both light top squarks and charginos. The charged Higgs contributions on the other hand are not $\tan \beta$ enhanced.

Recently, the purely leptonic decay of $B_s \rightarrow \mu ^+\mu ^-$ has received special attention due to the progress on both experimental results and theory calculations. This rare decay is very sensitive to supersymmetric contributions which are free from the helicity suppression of the SM diagrams. The recent observation of this decay by the LHCb [1071] and CMS [1072] experiments allows for a combined determination of its branching fraction to be

141 $$\begin{aligned} \mathrm {BR}(B_s\rightarrow \mu ^+\mu ^- )=(2.9\pm 0.7)\times \ 10^{-9} . \end{aligned}$$

While this is in accord with the SM prediction of $(3.53 \pm 0.38) \times 10^{-9}$ [1073], it also provides a stringent limit on the viable parameter space of many supersymmetric models. The SUSY contributions to the decay *amplitudes* are dominated by Higgs-mediated penguin diagrams [1074–1076] and are proportional to

142 $$\begin{aligned} -\mu A_t \, \frac{\tan ^3\beta }{(1+\epsilon _b \, \tan \beta )^2} \; \frac{M_t^2}{M_{\tilde{t}}^2} \, \frac{M_b M_\mu }{4\sin ^2\theta _W M_W^2 M_A^2}. \end{aligned}$$

The sensitivity of $B_s \rightarrow \mu ^+ \mu ^-$ to SUSY contributions is significant in regions at large $\tan \beta$ and small to moderate $M_A$ values, regions which are also probed by direct SUSY particle searches at ATLAS and CMS, in particular $H/A \rightarrow \tau ^+ \tau ^-$. As a result, while the constraints derived from the current LHCb result remove a large fraction of points at large $\tan \beta$ and low $M_A$, nonetheless for intermediate $\tan \beta$ values and/or large masses of the pseudoscalar Higgs boson *A*, the branching fraction in the MSSM does not deviate much from its SM prediction, leaving a sizeable fraction of SUSY parameter regions totally unconstrained [1077].

The decay $B\rightarrow K^* \mu ^+ \mu ^-$ gives also access to angular distributions, in addition to the differential branching fraction, and offers a variety of complementary observables. However, these observables suffer from large uncertainties, in particular due to form factors. A set of optimised observables has been defined from the ratios of angular coefficients to minimise hadronic uncertainties, while preserving the sensitivity to new physics effects [1078, 1079]. They have been recently measured by the LHCb Collaboration [1080] highlighting a tension in several binned observables. While these tensions remain even when including the SUSY contributions, the overall agreement with the MSSM predictions is within 1$\sigma$-level for an appropriate choice of the model parameters [1081].

Finally, the purely leptonic decay of $B_u\rightarrow \tau \nu _\tau$ is sensitive to supersymmetry through the exchange of a charged Higgs boson already at tree level, which does not suffer from the helicity suppression of the SM contribution with the exchange of a *W* boson. The branching ratio of $B_u \rightarrow \tau \nu _\tau$ in supersymmetry relative to the SM is given by

143 $$\begin{aligned} \frac{\mathrm {BR}(B_u\rightarrow \tau \nu _\tau )_{\mathrm {MSSM}} }{\mathrm {BR}(B_u\rightarrow \tau \nu _\tau )_{\mathrm {SM}}}=\left[ 1-\frac{m_B^2}{M_{H^+}^2}\, \frac{\tan ^2\beta }{1+\epsilon _0\tan \beta }\right] ^2, \end{aligned}$$

where $\epsilon _0$ is an effective coupling parametrising the non-holomorphic correction to the down-type Yukawa coupling induced by gluino exchange. This decay is therefore also very sensitive to the MSSM parameter region at large $\tan \beta$ and small $M_{H^+}$ values, and much less sensitive to other SUSY parameters. The branching fraction for the decay is calculated in the SM to be $(1.10\pm 0.29)\times 10^{-4}$ [1082], which exhibits a slight tension with the experimental averaged value of $(1.14\pm 0.22)\times 10^{-4}$ [1068].

#### Muon magnetic moment

The SUSY contribution to the muon magnetic moment is given by [1083]

144 $$\begin{aligned} {\varDelta } a_\mu ^{\mathrm{SUSY}}\propto \frac{M_\mu ^2\mu M_i\tan \beta }{M_{\mathrm{SUSY}}^4} \end{aligned}$$

where $i=1,2$ stands for electroweak gaugino masses and $M_{\mathrm{SUSY}}$ is the characteristic sparticle mass circulating in the muon–muon–photon vertex correction: $M_{\tilde{\mu }_{L,R}}$, $M_{\tilde{\nu }_\mu }$ and $M_{\tilde{\chi }_i}$.

The anomalous magnetic moment of the muon $a_\mu \equiv \frac{(g-2)_\mu }{2}$ was measured by the Muon *g*-2 Collaboration [818] which gives a $3.6\sigma$ discrepancy when compared to the SM calculations based on $e^+e^-$ data [891], ${\varDelta } a_\mu =a_\mu ^{\mathrm{meas}}-a_\mu ^{\mathrm{SM}}[e^+e^-]=(28.7\pm 8.0)\times 10^{-10}$. As discussed in more detail in Sect. 4, the SM prediction depends on the estimate of the hadronic vacuum polarisation contribution. Using $\tau$-decay data rather than low energy $e^+e^-$ annihilation data reduces the discrepancy to $2.4\sigma$ giving ${\varDelta } a_\mu =a_\mu ^{\mathrm{meas}}-a_\mu ^{\mathrm{SM}}[\tau ]=(19.5\pm 8.3)\times 10^{-10}$.

Attempts to explain the muon *g*-2 anomaly using supersymmetry usually invoke sparticle mass spectra with relatively light smuons and/or large $\tan \beta$ (see e.g. Ref. [1084]). Some SUSY models where $M_{\tilde{\mu }_{L,R}}$ is correlated with squark masses (such as mSUGRA) are now highly stressed to explain the $(g-2)_\mu$ anomaly, given the bounds from the LHC direct searches. In addition, since naturalness favours a low value of $|\mu |$, tension again arises between a large contribution to ${\varDelta } a_\mu ^{\mathrm{SUSY}}$ and naturalness conditions. The current $3\sigma$-deviation is clearly not sufficient to prove the existence of new physics, but in the future, progress can be expected both on the experimental side (due to a new measurement at Fermilab with four-fold improved precision [23]) as well as on the theoretical side [1085, 1086].

#### Dark matter and cosmological constraints

During the past several decades, a very compelling and simple scenario has emerged to explain the presence of dark matter in the universe with an abundance roughly five times that of ordinary baryonic matter. The WIMP miracle scenario posits that WIMPs would be in thermal equilibrium with the cosmic plasma at very high temperatures $T \ge M_{{\mathrm {WIMP}}}$. As the universe expands and cools, the WIMP particles would freeze out of thermal equilibrium, locking in a relic abundance that depends inversely on the thermally averaged WIMP (co)-annihilation cross section [1087, 1088]:

145 $$\begin{aligned} {\varOmega }_{\chi } h^2\simeq \frac{s_0}{\rho _c/h^2}\left( \frac{45}{8 \pi ^2 g_*}\right) ^{1/2} \frac{x_f}{M_P}\frac{1}{\langle \sigma v\rangle } \end{aligned}$$

where $s_0$ is the present entropy density, $\rho _c$ is the critical closure density, $g_*$ measures the degrees of freedom, $x_f=m/T_f$ is the inverse freeze-out temperature rescaled by the WIMP mass, $M_P$ is the reduced Planck mass and $\langle \sigma v\rangle$ is the thermally averaged WIMP annihilation cross section with *v* being the WIMP relative velocity. The WIMP “miracle” occurs in that a weak strength annihilation cross section gives roughly the measured relic abundance provided the WIMP mass is also of order the weak scale [1089].

**Fig. 122:**
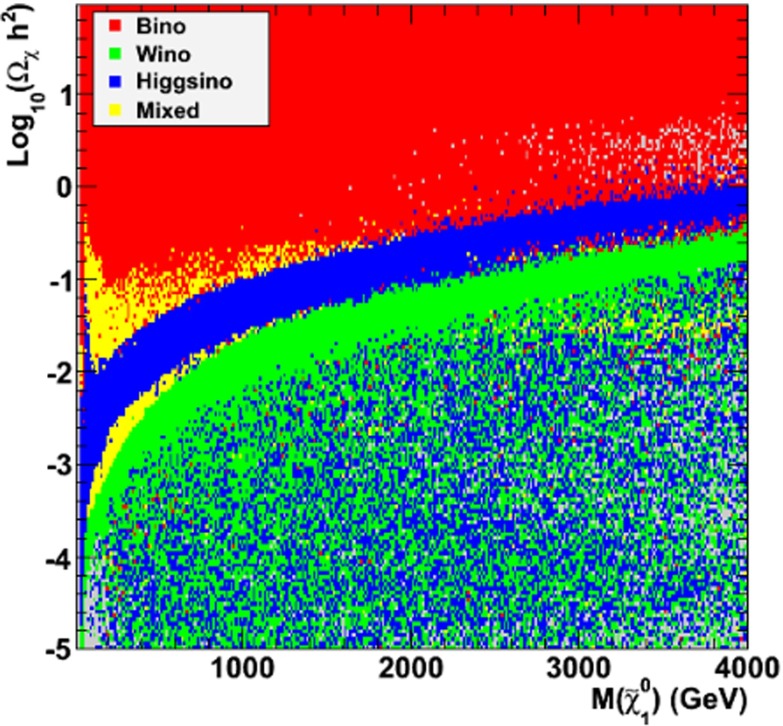
Neutralino relic density as a function of the neutralino LSP mass from a scan of the pMSSM parameter space. The colours indicate the nature of the neutralino LSP with the largest occurrence in each bin

The lightest neutralino of SUSY models has been touted as a prototypical WIMP candidate [1090–1092]. The precise determination of the DM relic density, ${\varOmega }_{{\mathrm {CDM}}} h^2$, obtained from the cosmic microwave background (CMB) by the WMAP satellite experiment first [1093] and the Planck mission [1094], now stands as a reference constraint for SUSY models. While the comparison of the measured abundance of CDM with the neutralino DM relic density, ${\varOmega }_{\chi } h^2$, computed in an assumed SUSY scenario, is affected by cosmological uncertainties which may be large [1095], it is certainly appropriate to require at least that SUSY models do not violate the upper bound on the CDM abundance, after accounting for these uncertainties. A predicted overabundance of thermally produced WIMPs may in fact be allowed in some specific models with either *R*-parity-violating WIMP decays, late WIMP decays to an even lighter LSP (e.g. axino or gravitino) or by late time entropy injection from moduli or saxion decays.

Despite the WIMP “miracle”, SUSY theories where the lightest neutralino plays the role of a thermally produced WIMP have a relic abundance ${\varOmega }_{\chi }h^2$ spanning over a broad range of values from several orders of magnitude larger than the value derived from the CMB spectrum in the case of a bino-like neutralino, and up to two-to-three orders of magnitude lower in the case of wino- or higgsino-like neutralinos [1096] with a mass of order 100 GeV; see Fig. 122. A wino- or higgsino-like neutralino LSP in the generic MSSM gives a relic density compatible with the CMB data for masses in the range 0.9–3 TeV, while bino-like or mixed neutralinos may match the CMB data for lighter masses. A deficit is, in principle, acceptable, since the neutralino may not be the only source of DM and its relic density should not necessarily saturate the measured value. As an example, in the case of the axion solution to the strong ${\textit{CP}}$ problem within the SUSY context, DM is due to a mixture of axions and neutralinos [1097]. For the case of bino-like LSPs where the abundance might be expected to exceed the WMAP/Planck value, an efficient annihilation mechanism – such as coannihilation, resonance annihilation or mixed bino–higgsino or mixed wino–bino annihilation – is needed. Such enhanced annihilation mechanisms define specific patterns of the masses of one or more SUSY particles compared to the lightest neutralino, which are important for searches at colliders.

**Fig. 123:**
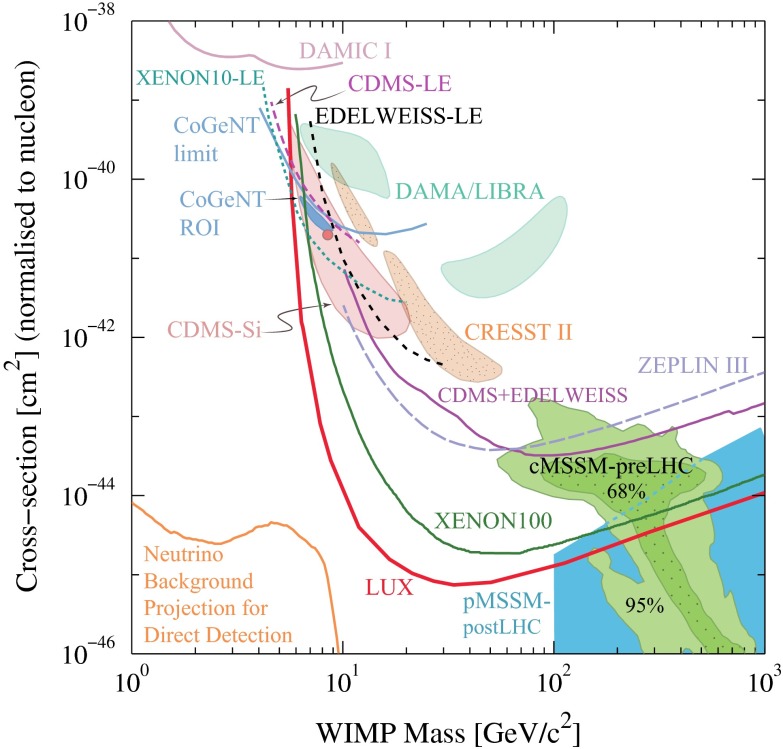
Limits on the $\chi$–*p* spin-independent scattering cross section vs. the $\chi ^0_1$ mass. The *shaded regions* include MSSM points compatible with recent LHC SUSY searches and Higgs mass results [1098]. Also indicated is the most stringent recent limit from the LUX experiment [1099]

The relic abundance constraint is now complemented by upper limits on WIMP-nucleon scattering cross sections from underground DM direct detection experiments. The $\tilde{\chi }p$ spin-independent scattering process receives SUSY contributions from scalar quark exchange and *t*-channel Higgs exchange [1092]. The latter dominates over a vast region of the parameter space. The scattering cross section retains a strong sensitivity on the scalar Higgs-boson mass and $\tan \beta$ [1100]. Limits on spin-independent $\chi$–nucleon scattering from the initial run of the LUX experiment [1099] are shown in Fig. 123 along with some expected SUSY parameter space.

**Fig. 124:**
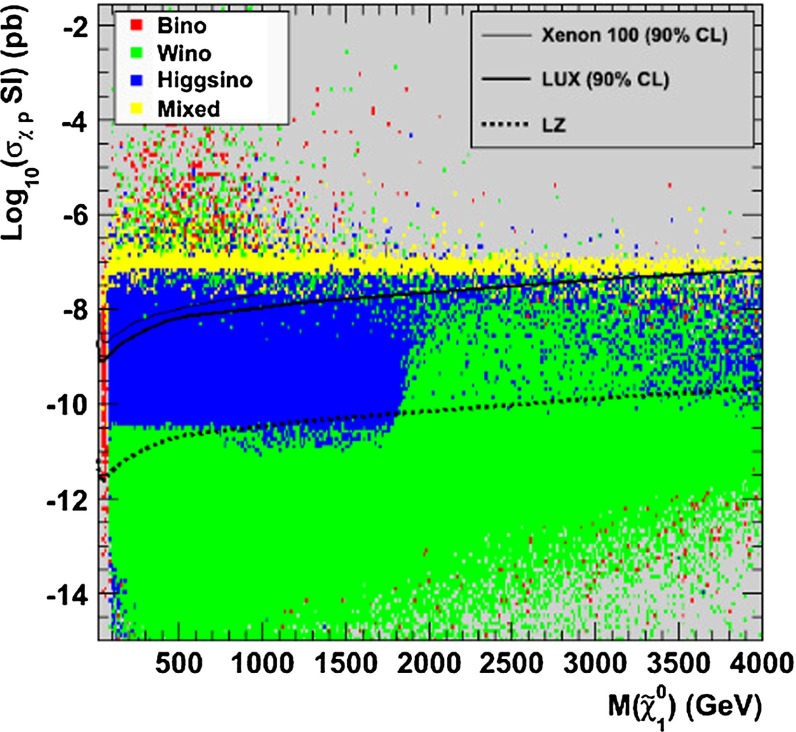
Neutralino–nucleon spin-independent scattering cross section vs. the $\chi ^0_1$ mass. The *colours* indicate the nature of the neutralino LSP with the largest occurrence in each bin

There is a large number of recent results reported by experiments using crystals [1101, 1102], semiconductors [1103, 1104] and noble gases [1099, 1105] as sensitive material. The excess of events reported by some of these experiments [1101, 1102, 1104, 1106], which would appear to point to a very light WIMP, are confronted by the stringent limits set by negative results in the searches by the xenon-based detectors, Xenon-100 [1107] and LUX [1099]. These limits are cutting into the region of scattering cross sections typical of the MSSM (see Fig. 124) and therefore provide some meaningful bounds, even if the systematics and model dependencies due to the assumed DM profile in the galaxy are known to be sizeable [1108]. In particular, the Xenon-100 and LUX bounds – if taken at face value – exclude a sizeable fraction of the viable SUSY points with neutralino DM at small values of the $\mu$ and $M_2$ parameters, which would give chargino- and neutralino-pair production observables at a linear collider with $\sqrt{s}$ below 1 TeV and small fine tuning, as discussed above. In the case where WIMPs make up only a portion of the total DM abundance (perhaps the bulk is composed of axions), these direct detection predictions would have to be rescaled by a factor $\xi ={\varOmega }_\chi ^{TP} h^2/0.12$, in which case the search limits are much less constraining.

In gravity mediation, the gravitino mass sets the scale for the soft breaking terms so that one expects gravitinos to have a mass comparable to the SUSY partners. While gravitinos may decouple from collider physics, they can be produced at large rates proportional to $T_R$ in the early universe. The gravitino decay rate to SUSY particles is suppressed by $1/M_P^2$ so that they may decay well after BBN has started, thus upsetting the successful prediction of light element production from Big Bang nucleosynthesis [1109]. To avoid this so-called “gravitino problem” [1110], one typically requires $T_R\mathop {\sim }\limits ^{<} 10^5$ GeV for $m_{3/2}<5$ TeV. Alternatively, if the gravitino is very heavy – $m_{3/2}\mathop {\sim }\limits ^{>}5$ TeV – then gravitinos typically decay before the onset of BBN. In addition, overproduction of gravitinos may lead to overproduction of LSPs from gravitino decay. To avoid overproduction of WIMPs arising from thermally produced gravitinos, one must typically obey the less restrictive bound $T_R\mathop {\sim }\limits ^{<} 10^5$ GeV.

Besides the case of neutralino DM, it is possible that gravitinos are the lightest SUSY particles and could be responsible for DM. The case of gravitino LSPs with a weak-scale value of $m_{3/2}$ is called the super-WIMP scenario and is again highly restricted by BBN bounds on late decaying WIMP to gravitino decays. Also, superWIMP gravitino LSPs can be thermally overproduced as DM unless constraints are again imposed on the reheating temperature [1111–1113]. For weak-scale gravitino DM, a reheating temperature above $10^9$ GeV can only be achieved in small corners of the model parameter space which impose strict bounds on the superparticle mass spectrum [1114].

Alternatively, the gravitino mass might be far below the weak scale; this scenario is a viable option and occurs naturally in GMSB scenarios. For such a small gravitino mass, the goldstino couplings are enhanced, which helps to evade the BBN constraints on NLSP decay to gravitinos. In addition, expectations for thermal overproduction of gravitino DM in GMSB are modified and can depend as well on the messenger mass scale [1115, 1116].

### Constraints from LHC

The searches performed by ATLAS and CMS on the 7 and 8 TeV LHC data in channels with jets, leptons and missing transverse energy (MET) have already significantly re-shaped our views of the high-energy frontier in relation to SUSY. Searches for the signatures of production and decay of supersymmetric particles with large MET have failed to reveal any significant excess of events compared to SM expectations.

A variety of final states have been probed in LHC searches which are sensitive to the production and decay modes of both strongly and weakly interacting SUSY particles. The results of searches for gluinos and squarks of the first two generations are easy to interpret in generic models. The analyses of the almost 25 fb$^{-1}$ results of combined 7 TeV and 8 TeV data have led to mass limits in the range of $m_{\tilde{g}}\mathop {\sim }\limits ^{>}1$–1.3 TeV and $m_{\tilde{q}}\mathop {\sim }\limits ^{>}0.4$–1.8 TeV for scalar quarks of the first two generations. There is an important exception to these limits which originates from scenarios with compressed spectra giving rise to highly degenerate masses and correspondingly low transverse energies from the produced jets and leptons: the visible energy from such compressed spectra often falls below analysis cuts or even the trigger thresholds, which causes generic LHC limits to collapse.

**Fig. 125:**
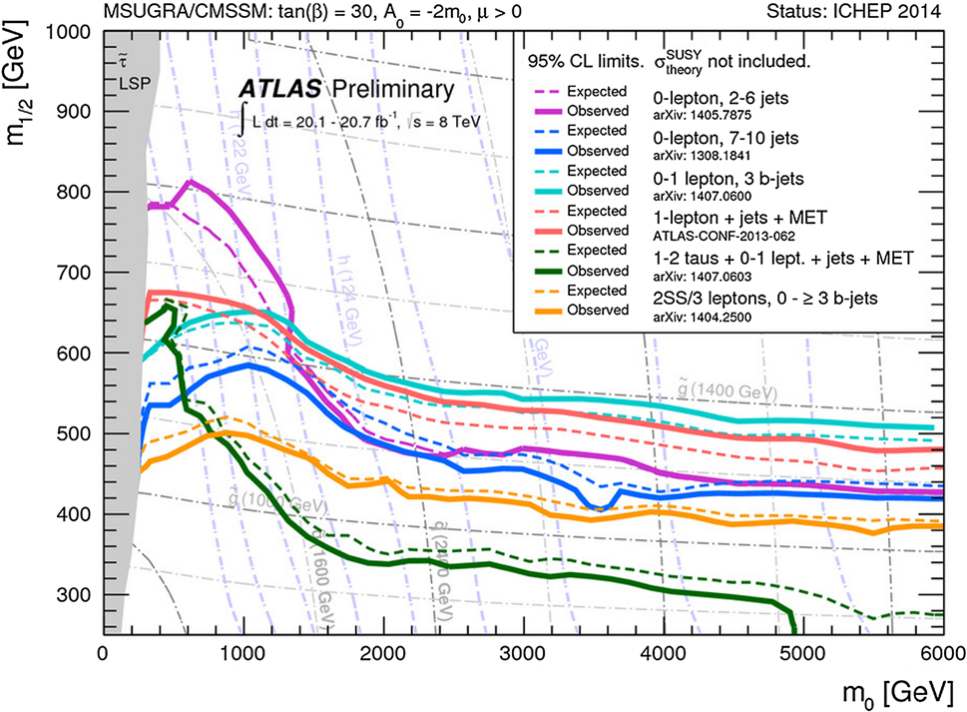
95 % CL exclusion limits for MSUGRA/CMSSM models with $\tan \beta =30$, $A_{0}=-2m_0$ and $\mu >0$ presented in the $[M_{0}, M_{1/2}]$ plane obtained by the ATLAS experiment with 20 fb$^{-1}$ of data at 8 TeV (from [1117])

These results have rapidly excluded most of the benchmark points adopted in the last two decades of SUSY studies and have put significant pressure on highly constrained SUSY models such as the CMSSM/mSUGRA model (discussed above) where SUSY soft terms are unified at a high scale. In fact, the LHC searches have excluded regions of parameter space which had been clearly preferred by fits performed on the pre-LHC data, pushing the masses of squarks and gluinos beyond 1–2 TeV (see Fig. 125). To further aggravate the crisis of such highly constrained models, it has also become difficult to accommodate a lightest Higgs boson with mass $\sim$125 GeV in the CMSSM, except for very specific parameter values [227, 1024]. In view of this, adopting more generic MSSM models without implicit correlations between the masses of the various SUSY particles, such as the so-called phenomenological MSSM (pMSSM), has become presently more common for studying SUSY theories at the LHC and at linear $e^+e^-$ colliders.

Still, the benchmark studies carried out for linear colliders keep much of their validity with respect to the sensitivity and accuracy of the measurements, even if the underlying models used in those studied have already been excluded by the LHC data.

**Fig. 126:**
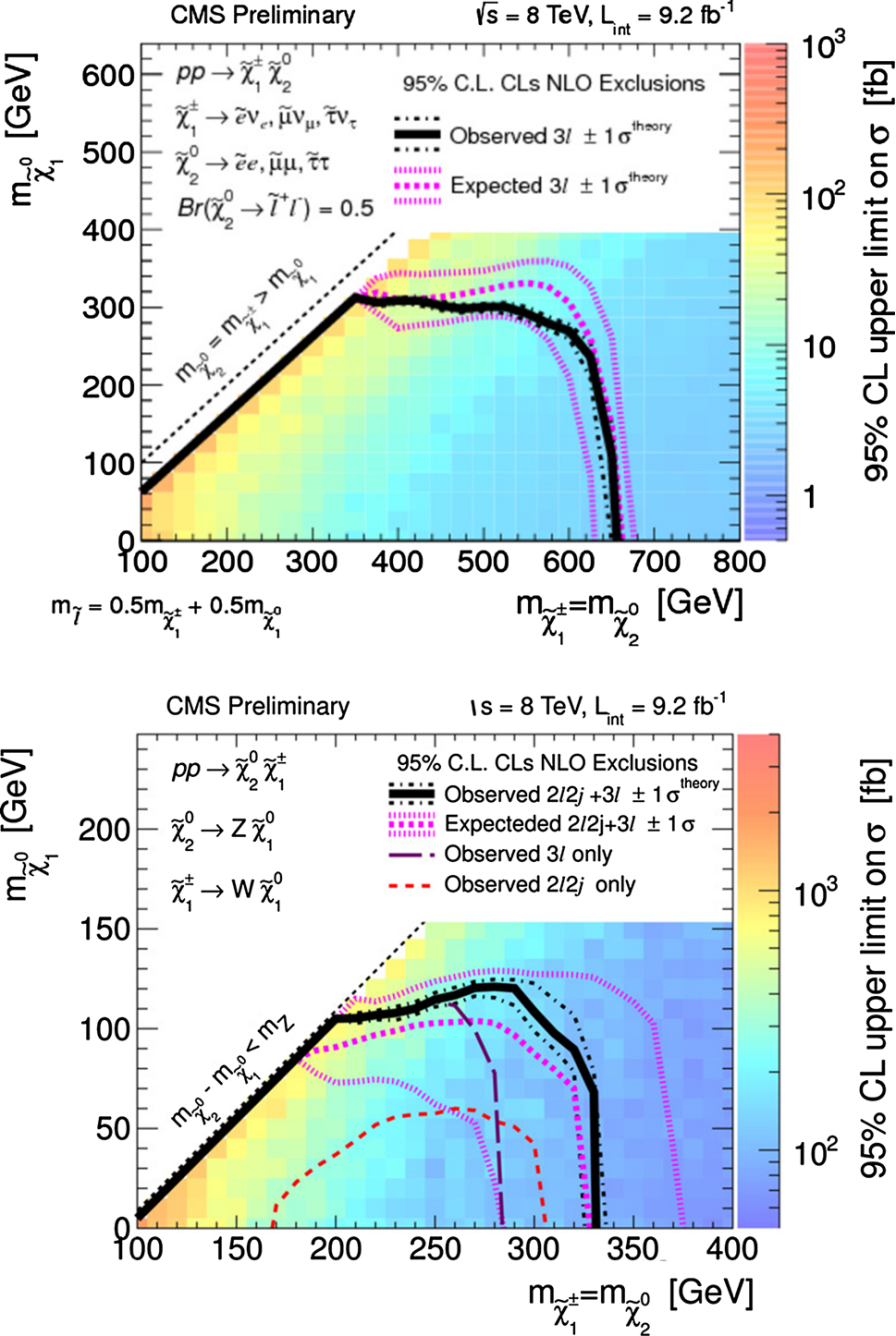
95 % CL exclusion limits on the chargino–neutralino production NLO cross section times branching fraction in the flavour-democratic scenario, for the three-lepton (*upper panel*), dilepton *WZ* $+$ MET and trilepton (*lower panel*) CMS searches with 9.2 fb$^{-1}$ of data at 8 TeV (from [1122])

Contrary to the case of constrained models, the mass limits for strongly interacting sparticles (in particular the gluino $\tilde{g}$ and the scalar quarks of the first two generations $\tilde{q}$) have little impact on the mass scale of their weakly interacting counterparts (charginos, neutralinos and scalar leptons) in generic models of supersymmetry, such as the pMSSM [1118–1121]. Searches for weakly interacting SUSY particle partners at LHC, of which the first results have recently been reported, are more model-dependent than the case of gluino and squark searches, since they depend not only on the mass splitting with respect to the lightest neutralino, but also on the mass hierarchy of the neutralinos and sleptons, as well as on the neutralino mixing matrix: e.g. the neutralino decay channels which yield multiple lepton final states used as experimental signatures include $\tilde{\chi }_2^0 \rightarrow \tilde{\ell } \ell$, $Z\tilde{\chi }_1^0$ or $\ell ^+ \ell ^-\tilde{\chi }_1^0$. These searches are probing charginos and neutralinos of mass up to $\sim$300–650 GeV, under these specific conditions (see Fig. 126). Extensive scans of the pMSSM have shown that significant regions of parameters giving rise to relatively light weakly interacting SUSY particles still remain unexplored and will not be probed even after the first operation of the LHC at its design energy of 14 TeV [1118, 1119, 1121].

There are regions in SUSY parameter space that are not well covered by the searches for missing energy and require more exotic search strategies. One example are scenarios where an electrically or colour-charged NLSP becomes long-lived on collider time-scales. This situation occurs either through strongly suppressed couplings of the LSP or through kinematic suppression. The former case naturally occurs in GMSB models where the lighter stau often is the NLSP. The clean signature of the resulting highly ionising charged tracks at the LHC typically lead to stronger limits on sparticle masses in such a model [1123, 1124]. The latter case occurs, e.g., in scenarios with a wino- or higgsino-like neutralino LSP being almost mass degenerate to the lightest chargino. Another example of exotic SUSY signatures are models with *R*-parity-violating couplings.

The recent observation of a Higgs-like particle with mass $\simeq$ 125 GeV at the LHC is opening new perspectives for SUSY searches at colliders. The mass of the newly discovered particle sets some non-trivial constraints on the SUSY parameters. In particular, the relatively large mass value observed implies strong restrictions on the scalar top mass and the mixing in the top sector [1024, 1125]. Heavy scalar top quarks and/or large mixing are required to bring the *h* boson mass around 125 GeV. The first measurement of the yields (or signal strengths $\mu$) in the decay channels studied so far – including $\gamma \gamma$, $ZZ^*$ and $WW^*$ – (although limited in accuracy and only at the level of upper limits in the important *bb* and $\tau \tau$ channels) will add further constraints. In particular, if interpreted within the SUSY framework, the data point towards a decoupling scenario, with a relatively heavy *A* boson. A possible enhancement in the $\gamma \gamma$ channel, observed by ATLAS and recently confirmed by the updated ATLAS study with 13 fb$^{-1}$ of 8 TeV data, may be a first hint of deviation from the SM expectations and could be explained through a reduction of the $b \bar{b}$ width as an effect of SUSY particle loops with intermediate, positive values of $\mu \tan \beta$ [256, 1126], or the contribution of light staus [1127–1129] or charginos [256]. Several of the preferred scenarios complying with $M_h \simeq$ 125 GeV and low values of the fine tuning parameter have sbottom particles lighter than the stops with multiple decay modes with comparable rates [1130]. This allows them to evade in part the constraints from direct LHC searches which assume a single dominant decay channel.

One of the indirect probes on the scale of SUSY particles is fine tuning. The gradual exclusion of SUSY particles at lower masses as a consequence of LHC searches naively affects the value of the fine tuning parameter, ${\varDelta }$, for the surviving SUSY models. It has been noted that in generic MSSM models, fine tuning is mostly determined by the $\mu$ parameter and an acceptably low fine tuning corresponds to small to moderate value of $|\mu |$. If fine tuning is taken as a criterion to select MSSM scenarios compatible with the 125 GeV Higgs mass, (setting ${\varDelta } < 100$ as has been proposed [1131]^59^) a constraint on the mass scale of weakly interacting sparticles is implicitly derived with values of $m_{\chi ^{\pm }_1}\le$ 270 GeV. This would match particularly well with the reach of a linear $e^+e^-$ collider with $\sqrt{s}$ energy in the range 0.5–1.0 TeV.

**Fig. 127:**
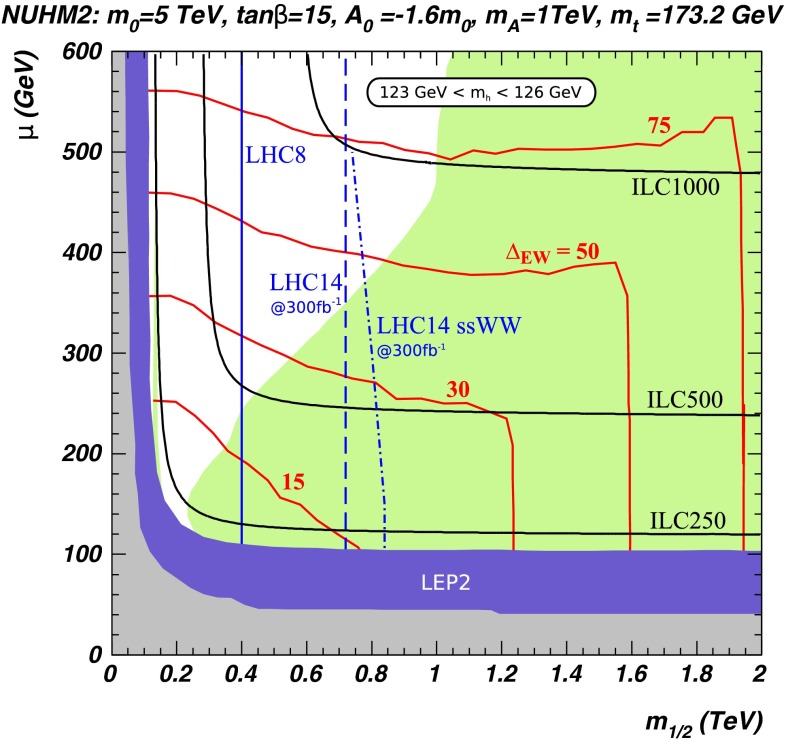
Plot of ${\varDelta }_{\mathrm{EW}}$ contours in the $m_{1/2}$ vs. $\mu$ plane of NUHM2 model for $A_0=-1.6 m_0$ and $m_0=5$ TeV and $\tan \beta =15$. We also show the region accesses by LHC8 gluino pair searches, and the region accessible to LHC14 searches with 300 fb$^{-1}$ of integrated luminosity. We also show the reach of various ILC machines for higgsino pair production. The *green-shaded* region has ${\varOmega }_{\tilde{\chi }^{0}_1}^{std}h^2<0.12$. Figure from [1132]

In summary, despite the far reaching constraints derived by the direct searches for SUSY production at the LHC, specific classes of models exist in the general MSSM and in constrained models such as NUHM2, which are consistent with the current bounds and have SUSY particles within reach of an $e^+ e^-$ collider operating at $\sqrt{s}\sim 0.25$–0.5 TeV and above. A recent study showed that over 20 % of the viable pMSSM models, not yet excluded by the combined LHC searches at 7 and 8 TeV, have the lightest chargino, $\chi ^{\pm }_1$, accessible at $\sqrt{s} = 0.5$ TeV increasing to 58 % for $\sqrt{s} = 1$ TeV and 94 % for 2 TeV [1130]. In addition, a study of natural SUSY NUHM2 parameter space in the $\mu$ vs. $m_{1/2}$ parameter plane shows the LHC8 and LHC14 reach (assuming 300 fb$^{-1}$) which will cover only a portion of the ${\varDelta }_{\mathrm{EW}}<30$ favoured parameter space. However, a $\sqrt{s}=0.5$–0.6 TeV $e^+e^-$ collider would access the entire low ${\varDelta }_{\mathrm{EW}}$ parameter space, thus either discovering light higgsinos or ruling out natural SUSY; see Fig. 127.

These considerations highlight the role of a high-energy $e^+ e^-$ collider as a complementary discovery machine compared to the LHC.

### Linear collider capabilities

As mentioned earlier, a linear $e^+e^-$ collider operating with $\sqrt{s}\mathop {\sim }\limits ^{>}2m(\mathrm{sparticle})$ can serve as a discovery machine, not only in models like natural SUSY, but also in DM motivated cases such as the stau coannihilation region or in *R*-parity-violating models where the LSP decays hadronically so that the SUSY signal is buried beneath QCD multi-jet backgrounds at the LHC.

Since SUSY is expected to (more than) double the number of physical particles over a possibly wide mass spectrum, an $e^+e^-$ collider with (1) a broad energy range, (2) the capability to precisely tune its $\sqrt{s}$ energy at well-defined values corresponding to new particle production thresholds, (3) the added analysing power afforded by beam polarisation and (4) possibly different beam species ($\gamma \gamma$, $e^- e^-$) appears ideally suited for a programme of detailed, high precision studies. The cross sections for pair production of SUSY particles are in the range 0.1–30 fb for masses of 200, 400 and 1200 GeV at $\sqrt{s} = 0.5, 1$ and 3 TeV, respectively. For comparison, those for the two SM processes $e^+e^- \rightarrow W^+ W^- \nu \bar{\nu }$ and $e^+e^- \rightarrow \mu ^+ \mu ^- \nu \bar{\nu }$ – which are the irreducible backgrounds to chargino and smuon pairs production – are 2, 10 and 25 fb and 25, 35 and 45 fb, respectively, at the same collision energies. These cross sections ensure a favourable signal-to-background ratio after appropriate selection cuts and make the study of SUSY particle pair production at a linear collider extremely promising!

**Fig. 128:**
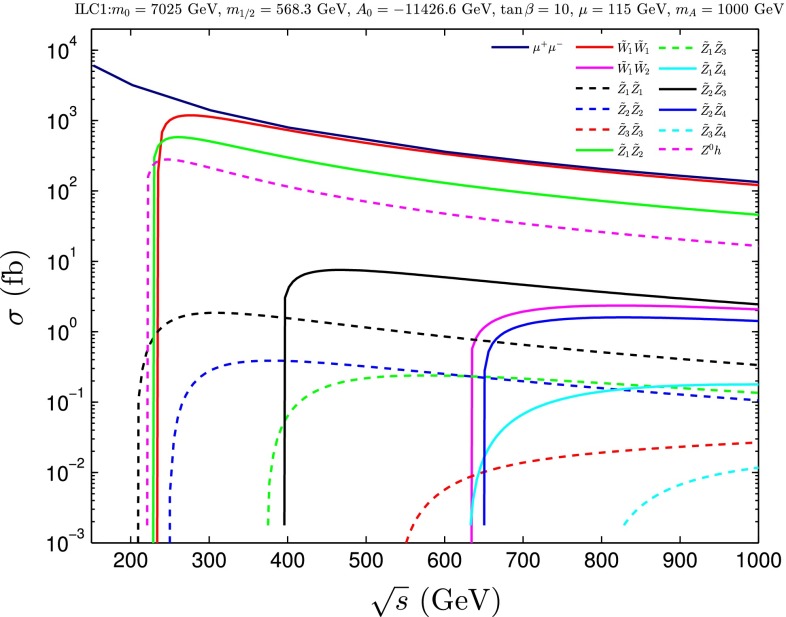
Sparticle production cross sections vs. $\sqrt{s}$ at a Higgsino factory for a radiatively driven natural SUSY benchmark point [1064]

Typical values of sparticle production cross sections are shown as a function of the collider energy, $\sqrt{s}$ in Fig. 128. If the fine tuning and naturalness arguments summarised in the previous section are taken as guidance, it is possible to identify scenarios where LHC searches may cover only a part of the parameter space, while a $\sqrt{s}=0.5$–0.8 TeV $e^+e^-$ collider would access the entire parameter space corresponding to low ${\varDelta }_{\mathrm{EW}}$ values. These considerations highlight the possible role of a linear $e^+e^-$ collider as a SUSY discovery machine, complementary to the LHC.

If SUSY exists, one of the major undertakings of collider physics is the precise determination of the quantum numbers and decay properties of the SUSY particle partners. At a linear collider, the masses of SUSY particles can be determined either by the end points of the energy distribution of the visible SM particle emitted in two-body decays (or even 3-body decays) or – more precisely but more demanding for the accelerator design and tuning – by dedicated energy scans at the onset of the pair production process. For typical SUSY spectra – having particles spaced from tens to hundreds of GeV – threshold scans set specific requirements on the accelerator design implying the flexibility to deliver collisions at several $\sqrt{s}$ energies with comparable luminosity and within the operating plan.

The capability of a linear collider in the study of SUSY has been studied for the last 20 years with increasing realism from the adoption of detailed simulation and reconstruction. New techniques for the optimal reconstruction of physics observables, such as the parton energy or the jet flavour, have been developed and new detector concepts and sensor technologies, tailored to the requirements of the linear collider physics programme have been introduced and demonstrated under realistic operating conditions. Supersymmetry has played an important role in setting these requirements and shaping the detector concepts. The recent studies for the ILC letters of intent (LoI) [207, 1133] and also the CLIC conceptual design report (CDR) [1134] have adopted full Geant-4 [1135] based simulation and detailed reconstruction, accounting for machine induced backgrounds. In most cases, the SUSY signatures can be clearly discriminated from the SM processes. Inclusive SUSY production often appears to be the major source of background for specific processes. In fact, different SUSY cascade decay chains [1136] may lead to the same final states. The ability to fully reconstruct the events with excellent energy resolution and to suppress some processes by changing the beam energy and, possibly, the beam polarisation offer excellent tools for ensuring an efficient study of each individual channel of interest. For example, the interference of the contribution of $\tilde{\chi }^+_1 \tilde{\chi }^-_1$ decays with $\tilde{e}^+_L \tilde{e}^-_L$ in *WW*$+$ missing energy and $\tilde{\chi }^0_2 \tilde{\chi }^0_2$ decays with $\tilde{\nu } \tilde{\nu }$ in *hh*$+$ missing energy is studied in detail with full simulation in [1137] and the separation of neutral and charged sleptons of the first/second generation in [1138]. Another important source of background is due to two-photon events, which may obscure the production of sfermion pairs, in particular in scenarios with small mass splitting. This background source can be controlled by ensuring electron tagging capability in the detector down to very small angles [1139].

#### Particle property measurements

*Mass measurements*

(a) In the continuum

The precise and unambiguous determination of SUSY particle masses is essential for the reconstruction of the theory fundamental parameters and for determining that the nature of the new physics is indeed supersymmetric. Mass reconstruction can be performed at an $e^+e^-$ linear collider by the reconstruction of the kinematics in SUSY particle pair production and by threshold energy scans. Threshold scans also provide us with access to the particle width, which is important since the narrow width approximation largely used in the context of the SM fails in general theories of new physics [1140].

In the two-body decay process $\tilde{A} \rightarrow B \tilde{C}$ of a SUSY particle $\tilde{A}$ into a lighter sparticle $\tilde{C}$ and a SM particle *B*, the masses of the parent and daughter sparticle can be extracted from the position of the kinematic edges of the energy spectrum of *B* since $\tilde{A}$ is produced with fixed, known energy in the pair production $e^+e^- \rightarrow \tilde{A} \tilde{A}$. The technique was first proposed in [1141] for two-body decays of sleptons and charginos, for squarks in [1142] and three-body and cascade decays in [1143] and later extended to other two-body decays [1144]. In the case of neutralino and chargino decays into bosons, where the daughter mass $M_B$ cannot be neglected (as in the case of squark and slepton decays), the relation between the energy endpoints and the masses of the particle involved in the decay process are given by

146 $$\begin{aligned} {E_{BH,BL}}= \gamma \left( E_B^{*} \pm \beta E_B^{*} \right) \end{aligned}$$

where

147 $$\begin{aligned}&E_B^{*} = \frac{M_A^2 + M_B^2 - M_C^2}{2 M_A},\end{aligned}$$

148 $$\begin{aligned}&\mathrm{with}\quad \gamma = \frac{\sqrt{s}}{2 M_A},\quad \mathrm{and}\quad \beta = \sqrt{\frac{1 - 4 M_A^2}{s}}. \end{aligned}$$

These formulae can be extended in a straightforward way to the case in which the particle $\tilde{A}$ is not directly produced in the $e^+e^-$ collisions but originates from the decay of a heavier particle, $\tilde{A}^{\prime }$, by replacing *s* with $E_A^2$, where $E_A$ is its energy. In the case of cascading decays $\tilde{A}^{\prime } \rightarrow \tilde{A} B^{\prime } \rightarrow B \tilde{C}$, $E_A$ is obtained as $\sqrt{s}-E_{B^{\prime }H} < E_A < \sqrt{s}-E_{B^{\prime }L}$.

The determination of the lower and upper endpoints of the energy spectrum constrains the ratio of the mass of $\tilde{A}$ to that of $\tilde{C}$. If the mass of $\tilde{C}$ – in most cases the lightest neutralino – is independently known, then $M_{\tilde{A}}$ can be extracted. The accuracy in the extraction of the masses by the endpoint technique depends on the resolution in determining $E_B$, which may be the resolution in measuring the momentum of a lepton in the case of sleptons or the energy of a jet (di-jet) in the case of a scalar quark (chargino or neutralino decaying into a boson). Excellent energy and momentum resolution are therefore essential. The energy of the beams at collision must also be known accurately because this enters in the determination of $\beta$. Beam–beam effects which induce radiation off the beam particles before collision are responsible for distortions of the luminosity spectrum, which must be precisely measured from collision data.

**Fig. 129:**
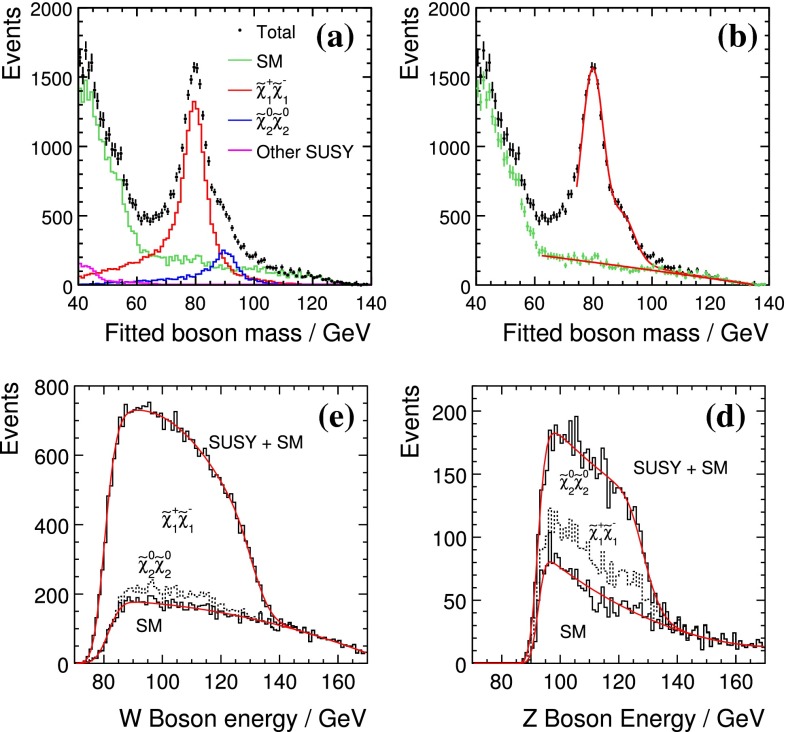
Di-jet mass (*upper plots*) and energy spectra (*lower plots*) for chargino and neutralino production at 0.5 TeV (from [207])

Detailed analyses, based on full Geant-4 detector simulation, digitisation and reconstruction and including the inclusive SM backgrounds, have validated earlier results on the expected accuracy on the mass determination for sleptons, gaugino and squarks at $\sqrt{s} = 0.5$ and 3 TeV. Studies for the ILD and SiD LoIs performed for the ILC parameters at 0.5 TeV [207, 1133], have shown that the kinematic endpoints of the energy spectrum of *W* and *Z* bosons produced in decays of chargino and neutralinos (see Fig. 129), respectively, can be determined with an accuracy of better than 1 GeV, thanks to the excellent performance of energy flow with highly segmented calorimeters in the reconstruction of parton energy [1145]. Kinematic fitting imposing equal masses of pair produced particle can be applied to improve the energy resolution. This translates into relative statistical accuracies in the determination of the $\tilde{\chi }^{\pm }_1$, $\tilde{\chi }^0_2$ and $\tilde{\chi }^0_1$ masses of 1, 0.5 and 0.7 %, respectively. These results confirm, with the realism of full simulation and reconstruction and full SM backgrounds, the findings of earlier studies indicating that the masses of gaugino could be measure to a relative statistical accuracy of $\sim$1 %.

The excellent momentum resolution, required by the study of the Higgs-strahlung process, implies that the accuracy on the mass determination is dominated by the beamstrahlung effects. Not only the dominant modes, such as $\tilde{\mu }^+_R \tilde{\mu }^-_R \rightarrow \mu ^+ \mu ^- \tilde{\chi }^0_1 \tilde{\chi }^0_1$, but also the subdominant process $\tilde{\mu }^+_L \tilde{\mu }^-_L \rightarrow \mu ^+ \mu ^- \tilde{\chi }^0_1 \tilde{\chi }^0_1$ can be studied in the 2-lepton $+$ missing energy final state. Scalar $\tilde{t}$ and $\tilde{b}$ quarks can be observed almost up to the kinematical threshold for the pair production process even in the case of small mass splitting with the $\tilde{\chi }^0_1$ with the signal cross section measured with a statistical accuracy of $\sim$15 % for the case of the $\tilde{b}$ [1133]. These scenarios at small mass splitting are of special relevance in relation to the DM relic density since stop or sbottom coannihilation may be responsible for reducing ${\varOmega }_{\chi } h^2$ to values compatible with the WMAP results and are very difficult for LHC searches. In addition, an $e^+e^-$ collider of sufficient energy to produce scalar top pairs can determine the stop mixing angles through a study of the $e_L^+e_R^- \rightarrow \tilde{t}_1 \tilde{t}_1$ and $e_R^+e_L^- \rightarrow \tilde{t}_1 \tilde{t}_1$ production with polarised beams along with study of the decays into multiple channels with comparable rate: such cases are difficult, if not impossible, at the LHC.

Much of the accuracy demonstrated by the detailed ILC studies at 0.5 TeV is preserved at multi-TeV energies, as confirmed by some of the studies carried out for the CLIC CDR [1134], which focussed on 3 TeV $e^+e^-$ collisions. Chargino and neutralino masses in the range 600–1000 GeV can be determined with a relative statistical accuracy of 1–2 % with unpolarised beams and 2 ab$^{-1}$ of data [1134, 1137]. The mass of $\tilde{\mu }_R$ of 1.1 TeV is again determined to $\sim$2 % with unpolarised beams and 1 % with polarised electrons and positrons, accounting for backgrounds [1134, 1146]. In addition to the weakly interacting SUSY particles, multi-TeV collisions may access scalar quark pair production, providing unique accuracy on their masses. In the case of a 1.1 TeV right-handed squark of the first generation a detailed study performed for 2 ab$^{-1}$ of integrated luminosity at $\sqrt{s} = 3$ TeV demonstrated a relative statistical accuracy on the mass of 0.5 % [1147]. The linear collider opportunities for precision study of SUSY particles extend to three-body decays [1148] of gauginos [1149, 1150], sleptons [1151] and scalar quarks [1152], which are more difficult for the LHC. In the study of these processes, SUSY becomes a possible background to the searches where different production and decay channels lead to the same final state or topology. In these cases, special attention must be paid to the use of tight cuts on discriminants based on neural networks or multivariate techniques which may induce strong biases on the kinematics and configuration of the selected events.

(b) At the threshold

An $e^+e^-$ linear collider with tunable beam energy can determine the sparticle masses by performing energy scans of their pair production cross section near threshold. In principle, this method often provides a better mass accuracy compared to the kinematic endpoint method discussed above, and also, in most cases, a constraint on the particle width. Threshold energy scans put significant requirements on the machine performance and versatility. Not only the beam energy needs to be varied over a broad range, but since the cross section at threshold is small a large amount of integrated luminosity must be dedicated to each scan. Effects from beamstrahlung, finite sparticle widths, and Sommerfeld rescattering [1153–1155] are important at threshold, while SUSY backgrounds are reduced, at least for the lighter states. It turns out to be preferable to concentrate the luminosity in a small number of scan points [1156]. Measurements at energies very close to the kinematic threshold are most sensitive to the width while those on the cross section rise above threshold are most sensitive to the mass. In general, on can achieve few per-mille precision for the mass determination from a threshold scan. In absolute numbers, the uncertainty for the width measurement is comparable, but since electroweak sparticle widths are typically a factor 1000 smaller than their mass, only an upper bound on the width can be established in most cases. With an $e^-e^-$ running option for the ILC, on the other hand, the selectron masses and widths can be measured with up to ten-fold better precision than in $e^+e^-$ collisions [1154, 1155], which is due to the fact that $\tilde{e}_R^-\tilde{e}_R^-$ and $\tilde{e}_L^-\tilde{e}_L^-$ pairs are produced in a s-wave rather than a p-wave, leading to a steep $\propto \beta$ rise near threshold.

A comparison of ILC mass measurements for various sparticles via continuum and via threshold measurements is shown in Table 30 (from Refs. [491, 1154, 1155, 1157]). Note that the threshold scans require some rough a priori knowledge of the sparticle masses and take significant amount of the running time at various energy points, which will reduce the statistics available at the highest energy. There have been a few detailed studies of run plan scenarios including threshold scans for SUSY particles which show the feasibility to acquire data at the thresholds of a few important processes, while accumulating a sizeable dataset at the highest operational energy [1158]. The scenarios adopted in those studies are now made obsolete by the recent LHC bounds, but the findings are still applicable in a general sense.

**Table 30:** Expected precision on sparticle masses (in GeV) for the SPS1a scenario [881] using polarised $e^\pm$ beams ($P_L(e^-) = 0.8, P_L(e^+) = 0.6$). ${\varDelta } m_{c}$ is from decay kinematics measured in the continuum (${\mathscr {L}} = 200/500/1000\,\text {fb}^{-1}$ at $\sqrt{s} = 400/500/750$ GeV), and $\delta m_\mathrm{th}$ and $\delta {\varGamma }_\mathrm{th}$ are from threshold scans (${\mathscr {L}}=100$ fb$^{-1}$ for $e^+e^-$ and ${\mathscr {L}}=5$ fb$^{-1}$ for $e^-e^-$). From Refs. [491, 1154, 1155, 1157]

$e^+e^-$	*m*	$\delta m_{c}$	$\delta m_\mathrm{th}$	${\varGamma }_\mathrm{th}$
$\tilde{\mu }_R$	143.0	0.2	0.2	$<$0.5
$\tilde{\mu }_L$	202.1		0.5	–
$\tilde{e}_R$	143.0	0.1	0.15	$<$0.4
$\tilde{e}_L$	202.1	0.8	0.3	$<$0.4
$\tilde{\nu }_{e}$	186.0	1.2	0.8	$<$0.7
$\tilde{\tau }_1$	133.2	0.3		
$\tilde{\chi }^\pm _1$	176.4	1.5	0.55	
$\tilde{\chi }^\pm _2$	378.2	3		
$\tilde{\chi }^0_1$	96.1	0.1		
$\tilde{\chi }^0_2$	176.8	2	1.2	
$\tilde{\chi }^0_3$	358.8	3–5		
$\tilde{\chi }^0_4$	377.8	3–5		

$e^-e^-$	*m*	$\delta m_\mathrm{th}$	${\varGamma }_\mathrm{th}$	
$\tilde{e}_R$	143.0	0.05	$0.21\pm 0.05$	
$\tilde{e}_L$	202.1	0.25	$0.25\pm 0.04$	

*Cross Sections, Width and Branching fractions*

**Fig. 130:**
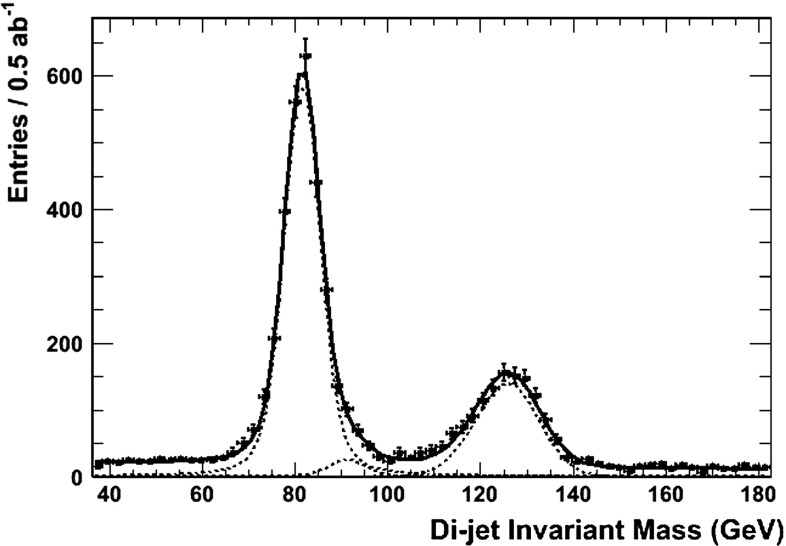
Di-jet invariant mass distribution in inclusive 4-jet $+$ missing energy SUSY events produced in $\sqrt{s}=3$ TeV $e^+e^-$ collisions for 0.5 ab$^{-1}$ of fully simulated events. The result of the fit to extract the boson content is shown by the *continuous line* with the individual *W*, *Z* and *h* components represented by the *dotted lines* (from [1162])

Decays of charginos and neutralinos into bosons, such as $\tilde{\chi }^{\pm }_1 \rightarrow W^{\pm } \tilde{\chi }^0_1$ and $\tilde{\chi }^{0}_2 \rightarrow Z \tilde{\chi }^0_1$ or $\tilde{\chi }_1^0 h$, are well suited to $e^+e^-$ collider capabilities. The four-jet + missing energy final states can be studied with good accuracy thanks to the small background and the excellent di-jet mass resolution ensuring separation of *W* from *Z* or *h* masses. Production cross sections of pairs of chargino and neutralino with mass of 216 GeV have been studied at 0.5 TeV and the statistical uncertainty on the cross section has been estimated at 0.6 and 2 %, respectively. It is interesting to observe that decays of SUSY particles, in particular neutralinos into the lightest Higgs boson, *h*, are common and even enhanced in specific models and combinations of MSSM parameters [1159–1162]. This opens up an interesting perspective of studying SUSY processes through the reconstruction of *h* pairs $+$ missing energy in four jet events, where Higgs-boson production is selected from that of other bosons by di-jet mass (see Fig. 130) and also *b*-tagging. A further possibility is the study of single Higgs boson plus missing *E* production via $e^+e^-\rightarrow \tilde{\chi }_1^0\tilde{\chi }_2^0$ with the decay $\tilde{\chi }_2^0\rightarrow \tilde{\chi }_1^0h$.

In addition, the determination of the dependence of the cross section for production of gaugino pairs, including $\tilde{\chi }^0_2 \tilde{\chi }^0_2$ and $\tilde{\chi }^+_1 \tilde{\chi }^-_1$, with the beam polarisation and energy is important to establish the nature of the $\tilde{\chi }^0_2$ and measure the chargino mixing angles and the $\mu$ parameter [1163].

$$\tau $$-*polarisation*

**Fig. 131:**
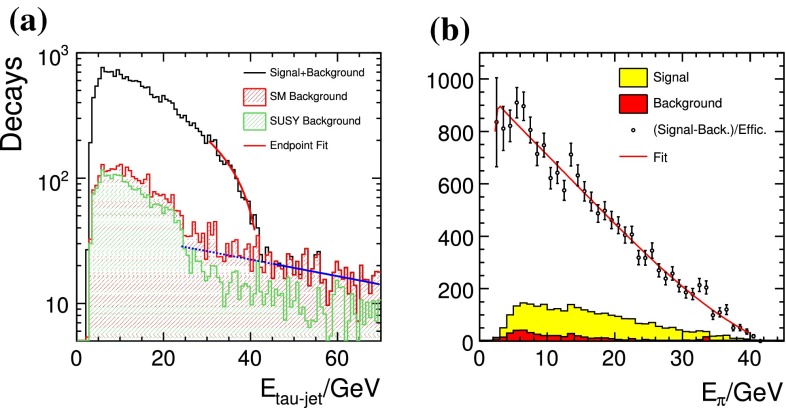
Energy spectrum of reconstructed $\tau$ leptons from $\tilde{\tau }_1$ decays (*left*) and energy distribution of the pions from 1-prong decays with the fit for the determination of the polarisation for fully simulated $e^+e^-$ events at 0.5 TeV (from [207])

The measurement of $\tau$ polarisation, $P_{\tau }$, in $\tilde{\tau }_1$ decays offers sensitivity to the mixing of interaction and mass eigenstates in the stau sector [1164]. $P_{\tau }$ is extracted from the energy spectrum of the pion emitted in the 1-prong decay $\tau \rightarrow \pi \nu$. Again, the $\pi$ energy spectrum depends on the collision energy and thus on beamstrahlung. Nonetheless, using realistic parameters for the ILC, the $\tau$ polarisation can be determined to a 15 % accuracy (see Fig. 131).

*CP-violating asymmetries*

The sub-leading, two-body decay $\tilde{\chi }^0_i \rightarrow \tilde{\ell }_{R} \ell \rightarrow \ell \ell \tilde{\chi }^0_1$ is sensitive to ${\textit{CP}}$ asymmetries in the triple product of the final particle momenta. This measurement, which would open the way to the detection of SUSY ${\textit{CP}}$ phases, is discussed below in more detail. While the measurement may be possible also at the LHC, the sensitivity of a linear collider is expected to be far superior. A detailed analysis, based on full simulation and reconstruction and which makes use of event kinematics, obtained values of $|M_1|$, $\mu$ and $M_2$ to a relative accuracy of 1% or better and the ${\textit{CP}}$ phases to $\sim$10 % resolving the sign ambiguity, for states accessible at $\sqrt{s} = 0.5$ TeV using polarised electron and positron beams [1165].

#### Testing the SUSY character

One of the most important aspects of new physics searches is to really identify the new physics model. Concerning SUSY theories, such an identification requires measurements beyond just determining the mass and spin of the new particle. In order to prove that the new physics candidate is indeed the SUSY partner of the corresponding SM particle, one also has to measure precisely their couplings [1166] and their quantum numbers. In this context also the special feature of carrying a Majorana character has to be proven for the neutral gauginos.

*Spin determination*

The spin is one of the fundamental characteristics of all particles and it must be determined experimentally for any new particles so as to clarify the nature of the particles and the underlying theory. In particular, this determination is crucial to distinguish the supersymmetric interpretation of new particles from other models.

In supersymmetric theories, spin-1 gluons and electroweak gauge bosons, and spin-0 Higgs bosons are paired with spin-1/2 gluinos, electroweak gauginos and higgsinos, which mix to form charginos and neutralinos in the non-coloured sector. This calls for a wide spectrum of necessary attempts to determine the nature of the new particles experimentally.

The measurement of the spins in particle cascades at LHC is quite involved [1167–1170]. While the invariant mass distributions of the particles in decay cascades are characteristic for the spins of the intermediate particles involved, detector effects strongly reduce the signal in practice.

In contrast, the spin measurement at $e^+e^-$ colliders is straightforward [1171, 1172]. A sequence of techniques – increasing in complexity – can be exploited to determine the spin of supersymmetric particles in pair production of sleptons, charginos and neutralinos in $e^+e^-$ collisions:

- rise of the excitation curve near the threshold,
- angular distribution in the production process,
- angular distribution in decays of the polarised particles and,
- angular correlations between decay products of two particles.

Within the general theoretical framework it can be proven that the second step (b) is already sufficient in the slepton sector, although in general the final-state analysis is required to determine the spin unambiguously in the chargino and neutralino sectors.

**Fig. 132:**
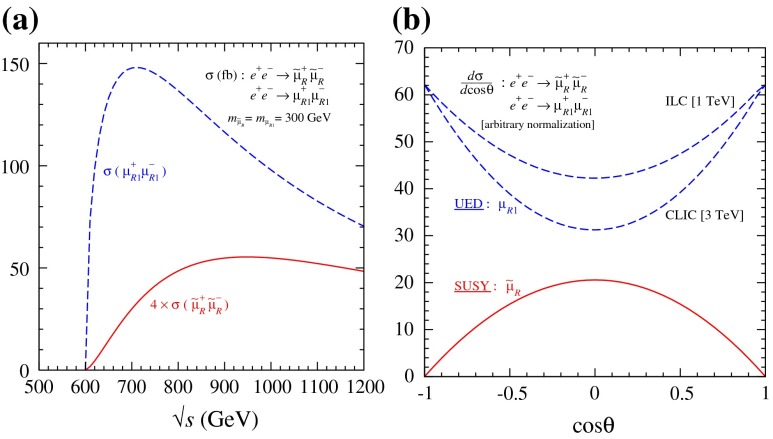
The threshold excitation (**a**) and the angular distribution (**b**) in pair production of smuons in the MSSM, compared with the first spin-1/2 Kaluza–Klein muons in a model of universal extra dimensions; for details, see Ref. [1172]

As shown clearly in Fig. 132, the threshold excitation curve and the production angle distribution for smuons in the MSSM are characteristically different from those for the first Kaluza–Klein muons in a model of universal extra dimension. Even though the p-wave onset of the excitation curve is generally a necessary but not sufficient condition, the $\sin ^2\theta$ law for the angular distribution in the production of sleptons (for selectrons close to threshold) is a unique signature of the fundamental spin-0 character.

**Fig. 133:**
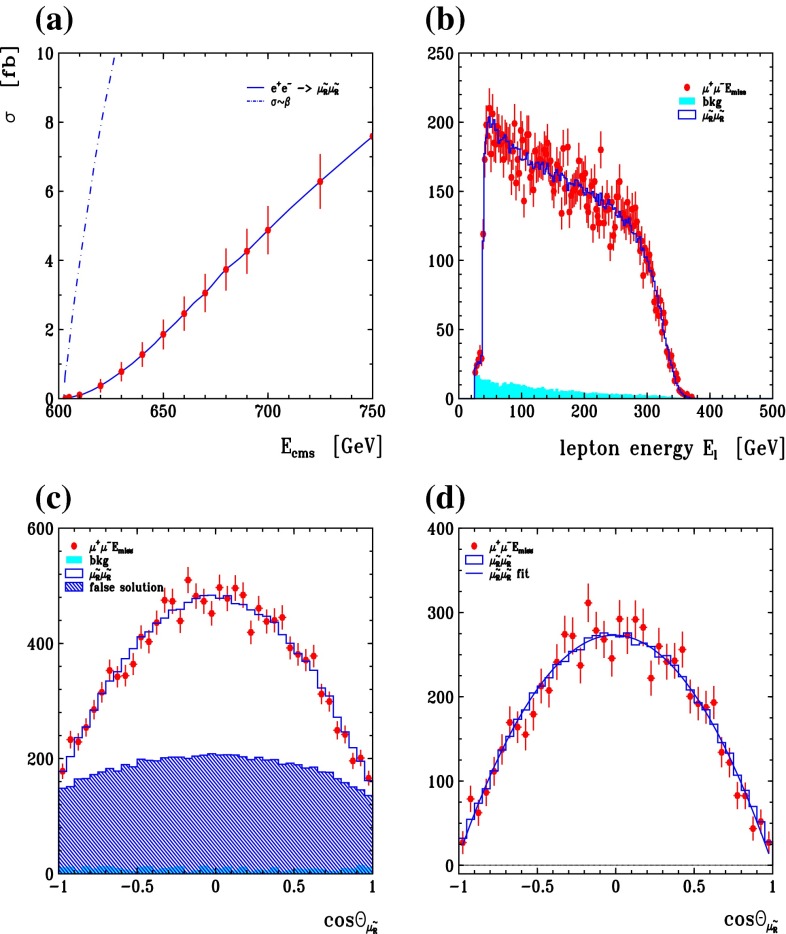
**a** The unpolarised cross section of $e^+e^-\rightarrow \tilde{\mu }_R^+ \tilde{\mu }^-_R$ production close to threshold, including QED radiation, beamstrahlung and width effects; the statistical errors correspond to ${\mathscr {L}}=10\,\mathrm{fb}^{-1}$ per point, **b** energy spectrum $E_\mu$ from $\tilde{\mu }_R^-\rightarrow \mu ^-\tilde{\chi }^0_1$ decays; polar-angle distribution $\cos \theta _{\tilde{\mu }_R}$ **c** with and **d** without contribution of false solution. The simulation for the energy and polar-angle distribution. The simulation for the energy and polar-angle distribution is based on polarised beams with $(P_{e^-},P_{e^+}) = (+0.8, -0.6)$ at $\sqrt{s}=1\,\mathrm{TeV}$ and ${\mathscr {L}}=500\, \mathrm{fb}^{-1}$. For details, see Ref. [1172]

The measurement of the cross section for smuon pair production $\tilde{\mu }^+_R\tilde{\mu }^-_R$ can be carried out by identifying acoplanar $\mu ^+\mu ^-$ pairs (with respect to the $e^\pm$ beam axis) accompanied by large missing energy carried by the invisible lightest neutralino $\tilde{\chi }^0_1$ in the decays $\tilde{\mu }^\pm _R\rightarrow \mu ^\pm \tilde{\chi }^0_1$. In addition, initial and final-state QED radiations, beamstrahlung and detector effects, etc. needs to be taken into account for reconstructing the theoretically predicted distributions. As shown in Fig. 133 through a detailed simulation, the characteristic p-wave threshold excitation and the production, as well as the flat decay distribution for the process $e^+e^-\rightarrow \tilde{\mu }^+_R\tilde{\mu }^-_R$ followed by the decays $\tilde{\mu }^\pm _R\rightarrow \mu ^\pm \tilde{\chi }^0_1$, can be reconstructed experimentally.

Unlike the slepton sector, the chargino and neutralino sectors in general have much more involved patterns. Neither the onset of the excitation curves near threshold nor the angular distribution in the production processes provides unique signals of the spin of charginos and neutralinos. However, decay angular distributions of polarised charginos and neutralinos, as generated naturally in $e^+e^-$ collisions, can provide an unambiguous determination of the spin-1/2 character of the particles albeit at the expense of more involved experimental analyses [1172]. Using polarised electron and/or positron beams will in general assure that the decaying spin-1/2 particle is polarised; reasonable polarisation analysis power is guaranteed in many decay processes.

Generally, quantum interference among helicity amplitudes – reflected typically in azimuthal angle distributions and correlations – may provide another method for determining spins [1173], although this method depends strongly on the masses of the decay products and the $\sqrt{s}$ energy, as the quantum interference disappears with increasing energy.

To summarise, the spin of sleptons, charginos and neutralinos can be determined in a model-independent way at $e^+e^-$ colliders. Methods similar to those applied to slepton pair production can be applied in the squark sector. For gluinos, a quite different methodology is required since these are not produced at tree level in $e^+e^-$ collisions.

*Yukawa couplings*

The SM/SUSY coupling relations are not affected by SUSY breaking and therefore the couplings of the SM particle are the same as those of their SUSY partners. That means, for instance, that the *SU*(3), *SU*(2) and *U*(1) gauge couplings $g_S$, *g* and $g'$ have to be identical to the corresponding SUSY Yukawa couplings $g_{\tilde{g}}$, $g_{\tilde{W}}$ and $g_{\tilde{B}}$. These tests are of fundamental importance. Concerning the test of the SUSY-QCD Yukawa couplings, first examinations could be performed at the LHC via determining the couplings in $\tilde{q}\tilde{g}$, $\tilde{g}\tilde{g}$ and $\tilde{q}\tilde{q}$ productions [1174]. These SUSY-QCD Yukawa studies have been accomplished by the analysis at a LC in[1175], so that one expects in total an uncertainty of about 5–10 % in the determination of the SUSY-QCD Yukawa couplings.

The SUSY-EW Yukawa coupling, however, is one of the final targets of LC experiments which should provide a complete picture of the electroweak gaugino sector with a resolution at the level of at least 1 % [43, 44]. Under the assumption that the *SU*(2) and *U*(1) parameters have been determined in the chargino/higgsino sector (see Sect. 5.7), we test precisely the equality of the Yukawa and gauge couplings via measuring polarised cross sections: varying the left-handed and right-handed Yukawa couplings has consequences on the measured cross sections. Depending on the electron (and positron) beam polarisation and on the luminosity, a per-cent level precision can be achieved.

*Quantum numbers*

One of the important tasks at future experiments is to determine model-independently the underlying quantum numbers of any new particles and check whether they correspond to their standard model counterparts. For instance, a particularly challenging measurement is the determination of the chiral quantum numbers of the sfermions. Although these are scalar particles, they have to carry the chiral quantum numbers of their standard model partners. Since chirality can be identified in the high-energy limit via helicity and its conservation, it will be part of the charge of a linear collider to prove such an association. Since the limits from LHC for the electroweak SUSY spectrum are not very strong, it is still the case that a rather light spectrum selectrons, smuons, staus continues to be viable.

**Fig. 134:**
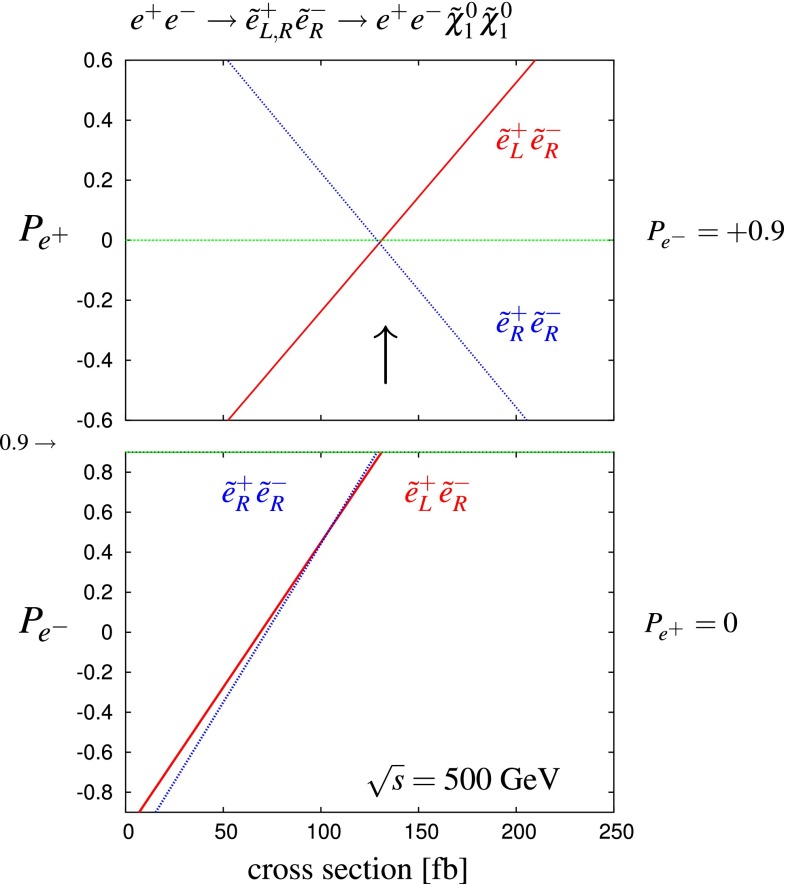
Polarised cross section versus $P(e^-)$ (*left panel*) or $P(e^+)$ (*right panel*) for $e^+e^-\tilde{e}\tilde{e}$-production with direct decay in $\tilde{\chi }^0_1e$ in a scenario where the non-coloured spectrum is similar to a SPS1a-modified scenario but with $m_{\tilde{e}_L}=200$ GeV, $m_{\tilde{e}_R}=195$ GeV [45]

In $e^+e^-$ collisions, the associated production reactions $e^+e^-\rightarrow \tilde{e}_L^+\tilde{e}_R^-,\ \tilde{e}_R^+\tilde{e}_L^-$ occur only via *t*-channel exchange, whereas the pair production reactions $\tilde{e}_L\tilde{e}_L$, $\tilde{e}_R\tilde{e}_R$ occur also via *s*-channel $\gamma$ and *Z* exchange. Since $m_{\tilde{e}_L}$ is in general not equal to $m_{\tilde{e}_R}$, then the electron energy distribution endpoints will be different for each of the four possible reactions as will the positron energy distributions. Furthermore, the total cross sections for each reaction depend strongly on beam polarisation so that by dialing the polarisation, one can move between distinct spectral possibilities, which allows one to disentangle the individual $\tilde{e}_L$ and $\tilde{e}_R$ masses, and to distinguish which one is which: *e.g.* measure their chiral quantum numbers; see Fig. 134. The masses of $m_{\tilde{e}_L}=200$ GeV, $m_{\tilde{e}_R}=195$ GeV are close, both particles decay directly to $\tilde{\chi }^0_1 e$.

**Fig. 135:**
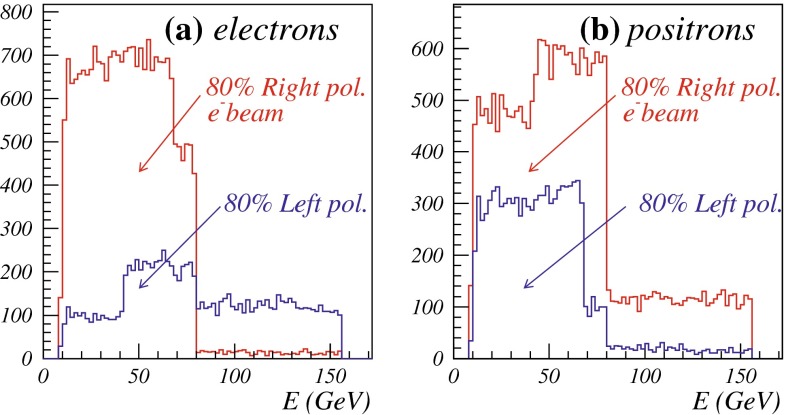
Electron and positron energy distributions for selectron pair production with the indicated beam polarisations and an integrated luminosity of 50 fb$^{-1}$ at $\sqrt{s}=500$ GeV (E. Goodman, U. Nauenberg et al. in Ref. [12])

The polarisation of $P(e^+)$ is mandatory in such cases. An example from Ref. [12] using an Isajet simulation is shown in Fig. 135.

*Majorana character*

Experimental tests of the Majorana character of gluinos and neutralinos will provide non-trivial insight into the realisation of SUSY in nature. There are several powerful methods for probing the nature of neutralinos in $e^\pm e^-$ collisions with polarised beams.

The parallelism between self-conjugate neutral vector gauge bosons and their fermionic supersymmetric partners induces the Majorana nature of these particles in the minimal $N=1$ supersymmetric extension of the standard model (MSSM). Therefore, experimental tests of the Majorana character of coloured gluinos and non-coloured electroweak neutralinos would provide non-trivial insight into the realisation of SUSY in nature, since extended supersymmetric models can include Dirac gauginos and/or higgsinos [1176–1178].

A theoretical basis for formulating a solid testing ground for Dirac gauginos is provided by a model with a continuous global *U*(1) *R* symmetry [1177, 1178] under which the Grassmann coordinates transform as $\theta \rightarrow e^{i\xi }\theta$, i.e. $R(\theta )=1$. It implies that the component fields of a supersymmetric superfield differ by the *R*-charge. Since the gauge superfields $\hat{G}$ are real, they must have a zero *R*-charge, $R(\hat{G})=0$, implying that $R=0$ for the gauge vector fields $G^\mu$ and $R=1$ for the spin-1/2 gauginos $\tilde{G}^\alpha$. Every term in the superpotential must have $R=2$ to provide a *R*-symmetric potential, while any soft-SUSY-breaking terms must have $R=0$.

When the *R*-charges of the MSSM matter, *H*-Higgs and gauge vector superfields are assigned as in Table 31, not only the supersymmetric $\mu$ term and the baryon- and lepton-number breaking terms but also the soft-SUSY-breaking Majorana mass terms and tri-linear *A* terms are forbidden. As a result, the sfermion left–right mixing and the proton decay through dimension-five operators are absent (while Majorana neutrino masses can be generated).

**Table 31:** The *R*-charges of the matter, Higgs and gauge superfields in the minimal *R*-symmetric supersymmetric standard model [1177]

Field	Superfield	*R* Charge
Matter	$\hat{L}, \hat{E}^c$	1
	$\hat{Q}, \hat{D}^c, \hat{U}^c$	1
*H*-Higgs	${\hat{H}}_{d,u}$	0
*R*-Higgs	${\hat{R}}_{d,u}$	2
Gauge vector	$\hat{G} = \{G^\mu , \tilde{G}^\alpha \}$	0
Gauge chiral	$\hat{\Sigma } = \{ \sigma , \tilde{G}'^\alpha \}$	0

Since the gaugino Majorana-type mass terms and the conventional higgsino $\mu$ term are forbidden in the *R*-symmetric theory, the superfield content of the minimal theory needs to be extended so as to give non-zero masses to gluinos, electroweak gauginos and higgsinos. The simplest extension, called the minimal *R*-symmetric supersymmetric standard model (MRSSM) [1177], is to introduce new chiral superfields $\hat{\Sigma }=\{\sigma , \tilde{G}'^\alpha \}$ in the adjoint representation of the SM gauge group in addition to the standard vector superfields as well as two iso-doublet chiral superfields $\hat{R}_d$ and $\hat{R}_u$ (*R*-Higgs) to complement the standard *H*-Higgs superfields $\tilde{H}_d$ and $\hat{H}_u$. (For a simpler formulation, see Ref. [1179].)

In the colour sector the original MSSM $$R=1$$ gluino $$\tilde{g}^a$$ and the new $$R=-1$$ gluino $$\tilde{g}'^a$$ ($$a=1$$–8) are coupled by the SUSY-breaking but *R*-symmetric Dirac mass term so that they can be combined into a single Dirac fermion field $$\tilde{g}^a_D = \tilde{g}^a_L + \tilde{g}'^a_R$$ with $$R=1$$. Note that $$\tilde{g}_D$$ is not self-conjugate any more, i.e. $$\tilde{g}^C_D\ne \tilde{g}_D$$ as the anti-gluino carries $$R=-1$$. In a similar manner the original electroweak gauginos, $$R=1$$$$\tilde{B}$$ and $$\tilde{W}^i$$ ($$i=1$$–3) and $$R=-1$$*H*-higgsinos, $$\tilde{H}_u$$ and $$\tilde{H}_d$$ are coupled with the new electroweak gauginos, $$R=-1$$$$\tilde{B}'$$ and $$\tilde{W}'^i$$ ($$i=1$$–3) and $$R=1$$*R*-higgsinos, $$\tilde{R}_u$$ and $$\tilde{R}_d$$, giving rise to four Dirac neutralinos $$\tilde{\chi }^0_{D1,\ldots ,D4}$$ with $$R=1$$ and four Dirac anti-neutralinos with $$R=-1$$.

The extension from the minimal model MSSM with Majorana gluinos and neutralinos to the *R*-symmetric MRSSM with Dirac gluinos and neutralinos as well as new *R*-Higgs bosons and adjoint scalar fields $\sigma$ leads to a lot of distinct phenomenological consequences on sparticle productions at the LHC and $e^\pm e^-$ colliders [1178, 1180–1182], flavour and ${\textit{CP}}$ problems [1177, 1183] and cold DM issues [1184–1186].

**Fig. 136:**
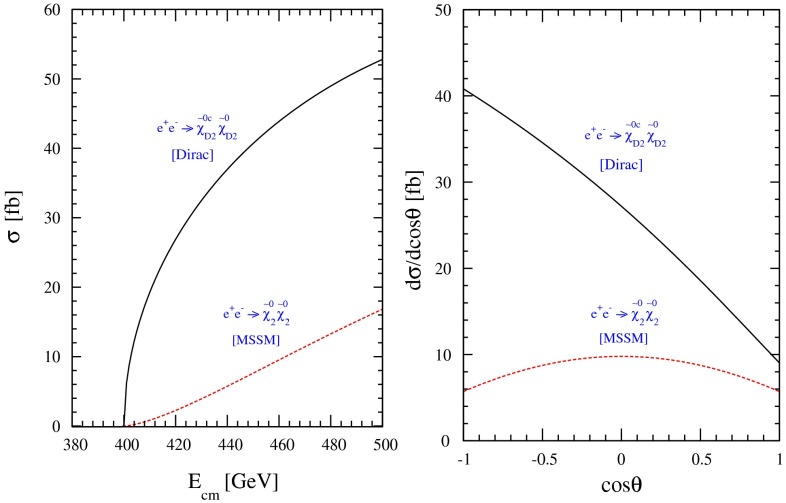
*Left* the total cross sections for pair production of wino-like neutralinos near threshold in the MSSM and the Dirac theory. *Right* dependence of the cross sections on the production angle $\theta$ for $\sqrt{s}=E_\mathrm{cm}=500$ GeV. The sparticle masses in both plots are $m_{\tilde{\chi }_2^0} = m_{\tilde{\chi }_{D2}^0} = 200$ GeV and $m_{\tilde{e}_L} = 400$ GeV (For the details, see Ref. [1192])

There are several methods to investigate the nature of gluinos at the LHC. In the original form, decays to heavy stop/top quarks are exploited [1187–1189] to test whether the final state in the fermion decay $\tilde{g} \rightarrow \tilde{t} \bar{t} + {\tilde{t}}^*t$ is self-conjugate. The standard production processes for investigating the nature of gluinos [1190] are the production of a pair of equal-chirality squarks, $q_Lq'_L\rightarrow \tilde{q}_L\tilde{q}'_L$ and $q_Rq'_R\rightarrow \tilde{q}_R\tilde{q}'_R$. While the cross section for the scattering processes with equal-chirality quarks is non-zero in the Majorana theory, it vanishes in the Dirac theory. Owing to the dominance of *u*-quarks over *d*-quarks in the proton, the Majorana theory predicts large rates of like-sign dilepton final states from squark pair production with an excess of positively charged leptons [1191], while they are absent, apart from a small number of remnant channels, in the Dirac theory. (In a realistic analysis one has to include gluino production processes which can also feed the like-sign dilepton signal but can be discriminated by extra jet emission from the gluino decays.) In addition, the nature of neutralinos could be checked at the LHC if cascade squark-decay chains involving intermediate sleptons and neutralinos are identified, as the final-state $q\ell ^\pm$ invariant mass distributions are distinct [1192].

An $e^\pm e^-$ collider with polarised beams is an ideally clean and powerful instrument for testing the nature of neutralinos. In parallel to the squark pair production through quark–quark collisions, the processes $e^-e^-\rightarrow \tilde{e}^-_R\tilde{e}^-_R$ or $\tilde{e}^-_L\tilde{e}^-_L$ with equal chirality indices and $e^+e^-\rightarrow \tilde{e}^+_L\tilde{e}^-_R$ or $\tilde{e}^+_R\tilde{e}^-_L$ are forbidden due to the conserved *R* charge in the Dirac case, while the processes occur in general in the Majorana case as in the MSSM [1190, 1192–1194].

Another powerful experimental test for characterising the nature of neutralinos is based on the threshold behaviour of the neutralino diagonal-pair production and its polar-angle distribution (Fig. 136). In the case with Dirac neutralinos $\tilde{\chi }^0_D$, the cross section for the process $e^+e^-\rightarrow \tilde{\chi }^0_{Di}\tilde{\chi }^0_{Di}$ ($i=1$–4) exhibits a typical sharp s-wave excitation and a forward–backward asymmetric angular distribution, while in the case with Majorana neutralinos the cross section for neutralino diagonal pair production in $e^+e^-$ collisions is excited in the characteristic slow p-wave, and the angular distribution is forward–backward symmetric [1192].

To summarise, the gluinos, the electroweak gauginos and the electroweak higgsinos are either Majorana or Dirac fermions in extended supersymmetric models. The $e^\pm e^-$ colliders and the LHC provide us with various complementary and powerful tests for probing the nature of new fermionic states from which we can get non-trivial insight into the realisation of SUSY in nature and find new directions for collider phenomenology as well as many related fields.

### From SUSY measurements to parameter determination

The measurements which can be performed from operating a linear collider with a large enough energy $\sqrt{s}\ge$0.5 TeV and luminosity, to collect of order of 0.5–2 ab$^{-1}$ of data, can be turned into precise predictions on the fundamental MSSM parameters of the Lagrangian of the theory, on their evolution to the unification scale, and on the relic density of light neutralinos in the universe inferred from collider data. These quantities are crucial to understand the underlying structure and to identify the SUSY model and its connections to cosmology. In this section, we discuss the extraction of these parameters based on the anticipated accuracy of measurements of SUSY particle properties at a linear collider.

#### General strategy

Since the general MSSM depends already on over 100 new parameters, it is a true challenge to measure all parameters in as model-independent fashion as is possible. Therefore often model assumptions – in particular on the SUSY-breaking mechanism and mass unifications – are made (see Sect. 5.2) resulting in a reduction to just 4–6 SUSY parameters. Then for unravelling the underlying SUSY model one needs a model-independent strategy for measuring the parameters. Since the current results from LHC point towards the TeV scale for the coloured SUSY partners, it is clear that one would need a combined approach from LHC and the LC to resolve the SUSY puzzle. The determination of the fundamental SUSY parameters at low energy would allow a critical test of the theory: extrapolating the mass parameters to the GUT scale points to which SUSY-breaking scheme might be realised in nature. Such extrapolations would be an important achievement, which illustrates well the complementarity of data from the LHC and a linear collider [1163, 1195, 1196] (see also Sect. 5.7.7).

The fundamental parameters of the gaugino/higgsino sector are the *U*(1) and *SU*(2) gaugino masses $M_1$ and $M_2$, and the higgsino mass parameter $\mu$, where also $M_i$ and $\mu$ can contain ${\textit{CP}}$-violating phases. In addition, also $\tan \beta$ enters the mixing of this electroweak particle SUSY sector. These parameters can – very accurately and independently of the underlying SUSY breaking scheme – be determined at a LC. This has been shown in many detailed studies[1141, 1149, 1197].

In the case the full spectrum, $\tilde{\chi }^0_i$, $\tilde{\chi }^{\pm }_j$, $i=1,\ldots ,4$, $j=1,2$, is accessible, the determination of the fundamental parameters via measurements of masses and cross sections seems to be trivial and is therefore not discussed here in detail. In this case, however, stringent tests of the closure of the system can be designed. Models with additional chiral and vector superfields extend the gaugino/higgsino sector. Since unitary matrices diagonalise the system, powerful sum rules can be set up for the couplings and a unique test whether the observed 4-system is closed or not might be possible. These sum rules for couplings can be directly converted into high-energy sum rules for production cross sections of neutralinos [1197]:

149 $$\begin{aligned} \mathrm{lim}_{s\rightarrow \infty }s \Sigma ^{4}_{i\le j}\sigma _{ij}= & {} \frac{\pi \alpha ^2}{48\cos ^4\theta _W\sin ^4\theta _W}\nonumber \\&\times \, [64 \sin ^4\theta _W -8 \sin ^2\theta _W +5 ] \end{aligned}$$

In this case, one also has to provide a measurement for the production $\tilde{\chi }^0_1\tilde{\chi }^0_1$. This final state is invisible in *R*-parity invariant theories where $\tilde{\chi }_1^0$ is the LSP. Nevertheless, it can be studied indirectly by photon tagging in the final state $\gamma \tilde{\chi }^0_1\tilde{\chi }^0_1$, which can be observed with a rather high accuracy at a LC. More details of photon tagging are included in the ’light higgsino’ section.

The powerful test via sum rules stresses the importance of upgrading the collider to achieve high $\sqrt{s}$ energies, if physics dictates it, in addition to combining LC and LHC results. In order to reconstruct the complete MSSM Lagrangian and evolve the parameters to the GUT scale [1198], it is generally needed to combine the linear collider measurements with those of squarks and gluinos (and possibly heavier gauginos) observed probably first at the LHC. Results at 0.5 TeV and 3 TeV are discussed in [1134, 1196].

#### Parameter determination with $\tilde{\chi }^{\pm }_1$, $\tilde{\chi }^{0}_{1,2}$ only

Even if only $\tilde{\chi }^0_{1,2}$ and $\tilde{\chi }^{\pm }_1$ were accessible, the precise measurements of the masses as well as polarised cross section for $\tilde{\chi }^+_1\tilde{\chi }^-_1$, $\tilde{\chi }^0_1\tilde{\chi }^0_2$ in different beam polarisation configurations is sufficient to determine the fundamental SUSY parameters and allow mass predictions of the heavier particles, yet unseen SUSY states.

The diagonalisation of the two chargino system can be parametrised by two mixing angles $\phi _L$, $\phi _R$. Defining the mixing angles in the unitary matrices diagonalising the chargino mass matrix ${\mathscr {M}}_C$ by $\phi _L$ and $\phi _R$ for the left- and right-chiral fields, the fundamental SUSY parameters $M_2$, $|\mu |$, $\cos \Phi _\mu$ and $\tan \beta$ can be derived from the chargino masses and the cosines $c_{2L,R}=\cos 2\phi _{L,R}$ of the mixing angles [1199, 1200].

If only the light charginos $\tilde{\chi }^\pm _1$ can be produced, the mass $m_{\tilde{\chi }^\pm _1}$ as well as both mixing parameters $\cos 2\phi _{L,R}$ can be measured. The quantities $\cos 2\phi _{L,R}$ can be determined uniquely if the polarised cross sections are measured at one energy including transverse beam polarisation, or else if the longitudinally polarised cross sections are measured at two different energies.

The heavy chargino mass is bounded from above after $m_{\tilde{\chi }^\pm _1}$ and $\cos 2\phi _{L,R}$ are measured experimentally. At the same time, it is bounded from below by not observing the heavy chargino in mixed light–heavy pair production:

150 $$\begin{aligned} {{\frac{1}{2}}} \sqrt{s} - m_{\tilde{\chi }^\pm _1}\le & {} m_{\tilde{\chi }^\pm _2}\nonumber \\\le & {} \sqrt{m^2_{\tilde{\chi }^\pm _1} + 4 m^2_W/| \cos 2\phi _L-\cos 2\phi _R|}. \end{aligned}$$

If both the light chargino mass $m_{\tilde{\chi }^\pm _1}$ and the heavy chargino mass $m_{\tilde{\chi }^\pm _2}$ can be measured, the fundamental parameters $M_2$, $\mu$, $\tan \beta$ can be extracted unambiguously. However, if $\tilde{\chi }^\pm _2$ is not accessible, their determination depends on the ${\textit{CP}}$ properties of the higgsino sector.

**Fig. 137:**
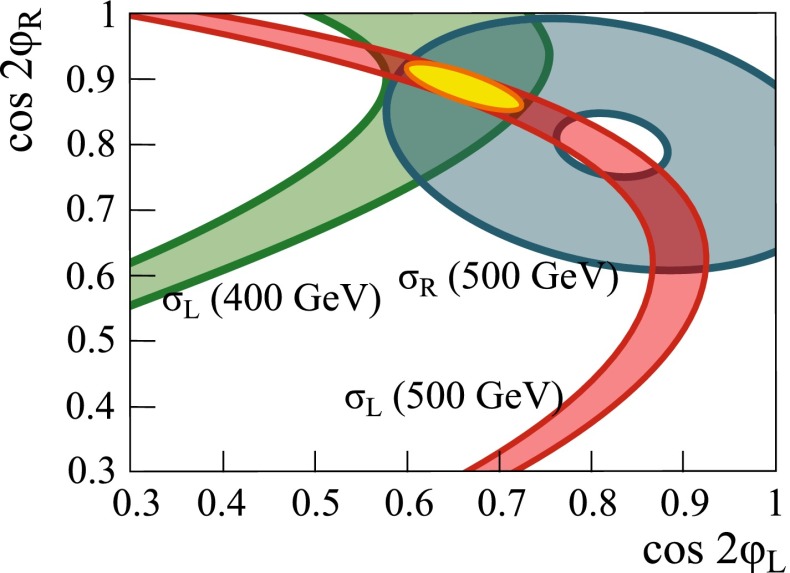
Determination of the chargino mixing angles $\cos 2 \Phi _{L,R}$ from LC measurements in $e^+e^-\rightarrow \tilde{\chi }^+_1\tilde{\chi }^-_1$ with polarised beams at different cms energies. The electroweak part of the spectrum in this scenario is a modified benchmark scenario SPS1a

**(A)** If the higgsino sector is *CP invariant*^60^, one can determine $m^2_{\tilde{\chi }^\pm _2}$ from the condition $\cos \Phi _\mu =\pm 1$, up to at most a two-fold ambiguity; see Refs. [1199, 1200]. This ambiguity can be resolved as well as the gaugino parameter $M_1$ be determined if observables from the neutralino sector, in particular, the mixed-pair $\tilde{\chi }^0_1 \tilde{\chi }^0_2$ production cross sections and $m_{\tilde{\chi }^0_{1,2}}$ are included; see Fig. 137.

**(B)** If $\tilde{\chi }^\pm _2$ is not accessible, the parameters $M_2$, $\mu$, $\tan \beta$, $\cos \Phi _{\mu }$ cannot be determined in a *CP non-invariant* theory in the chargino sector alone. They remain dependent on the unknown heavy chargino mass $m_{\tilde{\chi }^\pm _2}$. However, two trajectories can be generated in $\{M_2, \mu ; \tan \beta \}$ space, parametrised by $m_{\tilde{\chi }^\pm _2}$ and classified by the two possible values $\Phi _\mu$ and $(2\pi -\Phi _\mu )$ for the phase of the higgsino mass parameter. Including information from the neutralino sector, namely the measured masses and the polarised cross sections of the two light neutralino states $\tilde{\chi }^0_1$ and $\tilde{\chi }^0_2$, the heavy chargino mass $m_{\tilde{\chi }^\pm _2}$ can be predicted in the MSSM and subsequently the entire set of fundamental gaugino and higgsino parameters can be determined uniquely [1197, 1202]: the symmetric neutralino mass matrix ${\mathscr {M}}_N$ is diagonalised by a unitary matrix, defined such that the mass eigenvalues $m_{\tilde{\chi }^0_i}$ of the four Majorana fields $\tilde{\chi }^0_i$ are positive.

The squared mass eigenvalues of ${\mathscr {M}}_N {\mathscr {M}}^\dagger _N$ are solutions of the characteristic equations [1197]

151 $$\begin{aligned} m_{\tilde{\chi }^0_i}^8-a\,m_{\tilde{\chi }^0_i}^6 +b\,m_{\tilde{\chi }^0_i}^4-c\,m_{\tilde{\chi }^0_i}^2+d=0 \end{aligned}$$

for $i=1,2,3,4$ with the invariants *a*, *b*, *c* and *d* given by the fundamental *SU*(2) and *U*(1) gaugino mass parameters $M_2$ and $M_1$, and the higgsino mass parameter $\mu$, i.e. the moduli $M_2$, $|M_1|$, $|\mu |$ and the phases $\Phi _1$, $\Phi _\mu$. Each of the four invariants *a*, *b*, *c* and *d* is a binomial of $\mathrm{Re}(M_1)=|M_1|\,\cos \Phi _1$ and $\mathrm{Im}(M_1)=|M_1|\,\sin \Phi _1$. Therefore, each of the characteristic equations in the set (151) for the neutralino mass squared $m^2_{\tilde{\chi }^0_i}$ can be rewritten in the form

152 $$\begin{aligned} \mathrm{Re}(M_1)^2+\mathrm{Im}(M_1)^2+ u_i\, \mathrm{Re}(M_1)+ v_i\, \mathrm{Im}(M_1)=w_i \end{aligned}$$

for $i=1$–4. The coefficients $u_i$, $v_i$ and $w_i$ are functions of the parameters $M_2$, $|\mu |$, $\Phi _\mu$, $\tan \beta$ and the mass eigenvalue $m^2_{\tilde{\chi }^0_i}$ for fixed *i*. The coefficient $v_i$ is necessarily proportional to $\sin \Phi _\mu$ because physical neutralino masses are ${\textit{CP}}$-even; the sign ambiguity for $\sin \Phi _\mu$, a result of the two-fold cos solution $\Phi _\mu \leftrightarrow (2\pi -\Phi _\mu )$, transfers to the associated sign ambiguity in the ${\textit{CP}}$-odd quantity $\mathrm{Im}(M_1)$, i.e. in $\sin \Phi _1$.

The characteristic Eq. (152) defines a circle in the $\mathrm{Re} M_1, \mathrm{Im} M_1$ plane for each neutralino mass $m_{\tilde{\chi }^0_i}$. With only two light neutralino masses $m_{\tilde{\chi }^0_1}$ and $m_{\tilde{\chi }^0_2}$ measured, we are left with a two-fold ambiguity. The intersection points of the two crossing points depend on the unknown heavy chargino mass $m_{\tilde{\chi }^\pm _2}$. By measuring the pair-production cross sections $\sigma _L\{\tilde{\chi }^0_1\tilde{\chi }^0_2\}$ and $\sigma _R\{\tilde{\chi }^0_1\tilde{\chi }^0_2\}$, a unique solution, for both the parameters $m_{\tilde{\chi }^\pm _2}$ and $\mathrm{Re}(M_1), \mathrm{Im}(M_1)$ can be found at the same time [1197]. As a result, the additional measurement of the cross sections leads to a unique solution for $m_{\tilde{\chi }^\pm _2}$ and subsequently to a unique solution for $\{M_1, M_2; \mu ; \tan \beta \}$ (assuming that the discrete ${\textit{CP}}$ ambiguity in the associated signs of $\sin \Phi _\mu$ and $\sin \Phi _1$ has been resolved by measuring the normal $\tilde{\chi }^0_2$ polarisation).

#### Sensitivity to heavy virtual particles via spin correlations

Detection of charginos and neutralinos provides not only a way to measure electroweakino sector parameters (discussed in the previous sections) but is also sensitive to heavy virtual particles exchanged in chargino or neutralino production. Chargino production in the MSSM proceeds by exchange of photon and *Z* boson in *s*-channel or sneutrino exchange in *t*-channel.

**Fig. 138:**
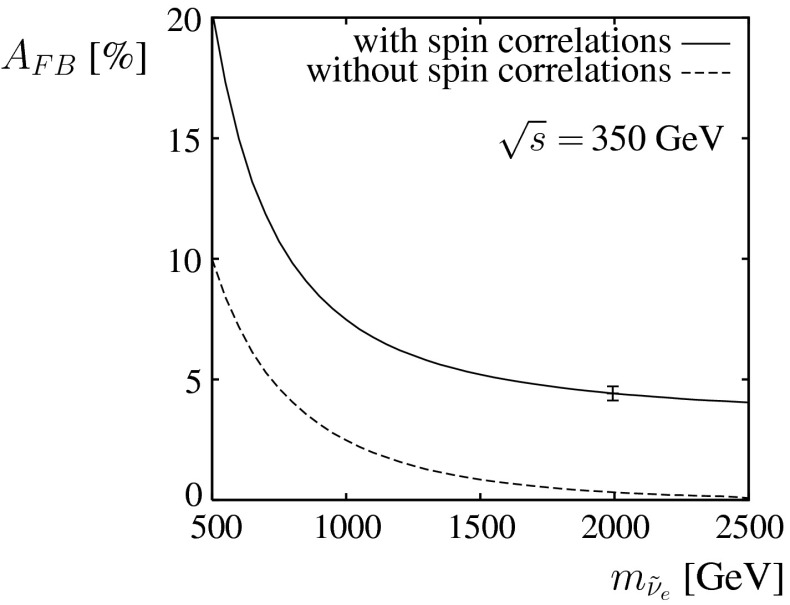
Forward–backward asymmetry of $e^-$ in $e^+e^-\rightarrow \tilde{\chi }^+_1\tilde{\chi }^-_1$, $\tilde{\chi }^-_1\rightarrow \tilde{\chi }^0_1\ell ^-\bar{\nu }$ as a function of $m_{\tilde{\nu }}$ at $\sqrt{s}=350$ GeV and with $P(e^-)=-90\,\%$, $P(e^+)=+60\,\%$. For a nominal value of $m_{\tilde{\nu }}=1994$ GeV the statistical error in the asymmetry is shown [1203]

In a study Ref. [1203], it was shown that the mass of a multi-TeV sneutrino can be measured up to precision of 10 % at the ILC. Forward–backward asymmetries of the final-state leptons and quarks from chargino decays. These asymmetries are spin-dependent observables: therefore, a correct evaluation of such asymmetries requires inclusion of spin correlations between production and decay of charginos. The asymmetry is in turn a highly sensitive probe of a particle exchanged in the *t*-channel, in this case mediated by a heavy sneutrino. This dependence, showing also the importance of including spin correlations, can be seen in Fig. 138.

In a scenario studied in Ref. [1203], the following set of parameters has been assumed:

153 $$\begin{aligned}&M_1 = 60\ {\mathrm {GeV}},\; M_2 = 121\ {\mathrm {GeV}},\; \mu =540\ \mathrm {GeV} \nonumber \\&\tan \beta = 20,\; m_{\tilde{\nu }} = 2\ {\mathrm {TeV}}. \end{aligned}$$

Using the light chargino production cross sections and mass, together with forward–backward asymmetries of decay products, a $\chi ^2$ fit has been performed to obtain the relevant MSSM parameters. The mass of the otherwise kinematically inaccessible sneutrino could be determined with a precision of

154 $$\begin{aligned} m_{\tilde{\nu }} = 2000 \pm 100 \ {\mathrm {GeV}} \end{aligned}$$

when forward–backward asymmetries for both leptonic and hadronic decays of chargino are used.

**Table 32:** Table of parameters (with the exception of $\tan \beta$ in GeV), for scenarios 1 (S1) and 2 (S2). Here $M_{(l/q)_{i}}$ and $M_{(e/u)_{i}}$ represent the left and right handed mass parameters for of a slepton/squark of generation *i* respectively, and $A_f$ is the tri-linear coupling for a sfermion $\tilde{f}$

S1				S2			
Parameter	Value	Parameter	Value	Parameter	Value	Parameter	Value
$M_1$	125	$M_2$	250	$M_1$	106	$M_2$	212
$\mu$	180	$M_{A^0}$	1000	$\mu$	180	$M_{A^0}$	500
$M_3$	700	$\tan \beta$	10	$M_3$	1500	$\tan \beta$	12
$M_{e_{1,2}}$	1500	$M_{e_{3}}$	1500	$M_{e_{1,2}}$	125	$M_{e_{3}}$	106
$M_{l_{i}}$	1500	$M_{q_{1,2}}$	1500	$M_{l_{i}}$	180	$M_{q_{i}}$	1500
$M_{{q/u}_{3}}$	400	$A_f$	650	$M_{{u}_{3}}$	450	$A_f$	$-1850$

#### Sensitivity to heavy virtual particles via loop effects

With the accuracy achievable at a linear collider, one requires loop corrections in order to draw meaningful conclusions about the underlying new physics parameters. For the electroweakino sector, a study was carried out in Ref. [1204] where one-loop predictions of the cross section and forward–backward asymmetry for chargino pair production and of the accessible chargino and neutralino masses were fitted to expected measurements. A number of one-loop calculations in the gaugino–higgsino sector can be found in the literature [1205–1218]. Although complex parameters were not considered in Ref. [1204], the renormalisation was performed following Refs. [1215–1218], where a dedicated renormalisation scheme in the complex MSSM was defined, in order that the analysis could easily be extended to the complex case. At tree level, there are four real parameters to be used in the fit: $M_1$, $M_2$, $\mu$ and $\tan \beta$, as well as the sneutrino mass, provided it is beyond the direct reach of the LC. The study aimed to provide information as regards the sensitivity to the remaining MSSM parameters which contribute to the masses and production amplitude via virtual effects. In the fit, the polarised cross sections and forward–backward asymmetry for chargino production as well as the $\tilde{\chi }_1^{\pm },\tilde{\chi }_2^{\pm }$ and $\tilde{\chi }^0_{1}, \tilde{\chi }^0_{2}, \tilde{\chi }^0_{3}$ masses – calculated at NLO in an on-shell scheme as described in Ref. [1204] – were used. Note that the masses are assumed to have been measured at the LC using the threshold scan method: however, the change in fit precision if the masses were obtained from the continuum was also investigated [7]. Further details of the fit method and errors are given in Ref. [1204]. The fit was performed for two scenarios, S1 and S2, shown in Table 32.^61^ The scenarios were chosen such as to be compatible with the current status of direct LHC searches [1219, 1220], indirect limits, checked using micrOmegas 2.4.1 [1221, 1222], and flavour physics constraints i.e. the branching ratio $\mathscr {B}(b\rightarrow s\gamma )$ and ${\varDelta }(g_\mu -2)/2$. Note that although in S1, $M_h$ is not compatible with the recent Higgs results from the LHC [96, 209], this could easily be rectified by changing $A_t$, which would have minimal effects on the results. The one-loop corrections to the polarised cross section and forward–backward asymmetry for $e^+e^-\rightarrow \tilde{\chi }^+_1\tilde{\chi }^-_1$ are calculated in full within the MSSM, following [1217, 1218], including soft and hard radiation.

For S1, the inputs for the fit included: the masses of the charginos ($\tilde{\chi }_1^{\pm },\,\tilde{\chi }_2^{\pm }$) and three lightest neutralinos ($\tilde{\chi }^0_{1},\,\tilde{\chi }^0_{2},\,\tilde{\chi }^0_{3}$), the production cross section $\sigma (\tilde{\chi }^+_1\tilde{\chi }^-_1)$ with polarised beams at $\sqrt{s} = 350$ and 500 GeV, the forward–backward asymmetry $A_{\mathrm{FB}}$ at $\sqrt{s} = 350$ and 500  $\mathrm {GeV}$ and the branching ratio $\mathscr {B}(b\rightarrow s\gamma )$ calculated using micrOmegas [1221, 1222].

For S2, the inputs for the fit were the same as in S1, with $\sqrt{s}=400$ GeV instead of 350 GeV and supplemented by the Higgs boson mass $M_h$. The sneutrino mass would have been measured. The results for S1, given in Table 33, show the fit to the 8 MSSM parameters: $M_1$, $M_2$, $\mu$, $\tan \beta$, $m_{\tilde{\nu }}$, $\cos \theta _{\tilde{t}}$, $m_{\tilde{t}_1}$, and $m_{\tilde{t}_2}$. We find that the gaugino and higgsino mass parameters are determined with an accuracy better than 1 %, while $\tan \beta$ is determined with an accuracy of 5 %, and 2–3 % for the sneutrino mass. The limited access to the stop sector (Table 33) could nevertheless lead to hints allowing a well-targeted search at the LHC. In Table 33, we also compare the fit results obtained using masses of the charginos and neutralinos from threshold scans to those obtained using masses from the continuum. For the latter, the fit quality deteriorates, clearly indicating the need to measure these masses via threshold scans. The results for S2 in Table 33 show that the fit is further sensitive to $m_{\tilde{t}_2}$, with an accuracy better than 20 %. In addition, an upper limit on the mass of the heavy Higgs boson can be placed at 1000 GeV, at the 2$\sigma$ level.

**Table 33:** Fit results (masses in GeV) for S1 (left) and S2 (right), for masses obtained from threshold scans (threshold fit) and from the continuum (continuum fit). Numbers in brackets denote $2\sigma$ errors

Parameter	S1		S2
	Threshold fit	Continuum fit	Threshold fit
$M_1$	$125 \pm 0.3 \;(\pm 0.7)$	$125 \pm 0.6\;(\pm 1.2)$	$106 \pm 0.3\;(\pm 0.5)$
$M_2$	$250 \pm 0.6\;(\pm 1.3)$	$250 \pm 1.6\;(\pm 3)$	$212 \pm 0.5\;(\pm 1.0)$
$\mu$	$180 \pm 0.4\;(\pm 0.8)$	$180 \pm 0.7\;(\pm 1.3)$	$180 \pm 0.4\;(\pm 0.9)$
$\tan \beta$	$10 \pm 0.5\;(\pm 1)$	$10 \pm 1.3\;(\pm 2.6)$	$12 \pm 0.3\;(\pm 0.7)$
$m_{\tilde{\nu }}$	$1500 \pm 24\;(^{+60}_{-40})$	$1500 \pm 20\;(\pm 40)$	–
$\cos \theta _{\tilde{t}}$	$0.15 ^{+0.08}_{-0.06}\;(^{+0.16}_{-0.09})$	$0 \pm 0.15\;(^{+0.4}_{-0.3})$	–
$m_{\tilde{t}_1}$	$400 ^{+180}_{-120}\;(^{\text {at limit}}_{\text {at limit}})$	–	$430 ^{+200}_{-130}\;(^{+300}_{-400})$
$m_{\tilde{t}_2}$	$800 ^{+300}_{-170}\;(^{+1000}_{-290})$	$800 ^{+350}_{-220}\;(^{\text {at limit}}_{\text {at limit}}$)	$1520 ^{+200}_{-300}\;(^{+300}_{-400})$
$m_{A^0}$	–	–	$<$650 ($<$1000)

Therefore, incorporating NLO corrections was shown to be required for the precise determination of the fundamental electroweakino parameters at the LC, and to provide sensitivity to the parameters describing particles contributing via loops. This work will soon be extended to a consideration of both the sensitivity to complex parameters and the neutralino production cross section.

#### Challenging scenarios: light higgsinos with sub-GeV mass gaps

In the MSSM, higgsino-like charginos and neutralinos are preferred to have masses of the order of the electroweak scale by naturalness arguments, as discussed in Sect. 5.3 of this review. If gauginos are heavy, such light $\tilde{\chi }^0_1$, $\tilde{\chi }^0_2$ and $\tilde{\chi }^\pm _1$ states will be almost mass degenerate and it will be very challenging to study them at the LHC. On the other hand, the clean experimental environment afforded by the ILC may allow one to perform a measurement of their properties. An analysis to assess the prospects of light higgsino measurements at the ILC, based on detailed simulations, is presented in [1223]. Two scenarios with light charginos and neutralinos and mass splitting between them in the range of 0.8–2.7 GeV, but all the other SUSY particle masses in the multi-TeV range were chosen (i.e. $\mu \sim 170$ GeV, $M_1\sim 5$ TeV, $M_2\sim 10$ TeV, $\tan \beta \sim 48$).

For such small mass differences, the decay products of chargino are soft pions and leptons, while the largest decay mode of $\tilde{\chi }^0_2$ is to photon and LSP. Despite the fact that these final states will suffer from large SM backgrounds, a suitable set of cuts provides separation of the signal [1223]. The effective tool for background rejection here is the tag of ISR photons recoiling against the chargino or neutralino system.

The masses of chargino and neutralino $\tilde{\chi }^0_2$ are then reconstructed from the distribution of the reduced centre-of-mass energy of the system recoiling against the hard ISR photon. The expected mass resolution ranges from 1.5 to 3.3 GeV depending on the scenario. The mass difference between $\tilde{\chi }^\pm _1$ and the LSP is measured by fitting energy distribution of soft pions in the respective decays. The accuracy up to 40 MeV can be obtained for $m_{\tilde{\chi }^\pm _1} - m_{\tilde{\chi }^0_1} = 770$ MeV. Finally, the polarised cross sections for chargino pair production and $\tilde{\chi }^0_1 \tilde{\chi }^0_2$ can be measured with order of per-cent statistical accuracy. These results are greatly encouraging for the potential of a linear collider to tackle even such difficult scenarios. Still, detailed studies with full detector simulation and reconstruction and the incorporation of machine-induced backgrounds will be necessary to fully quantify this potential.

**Fig. 139:**
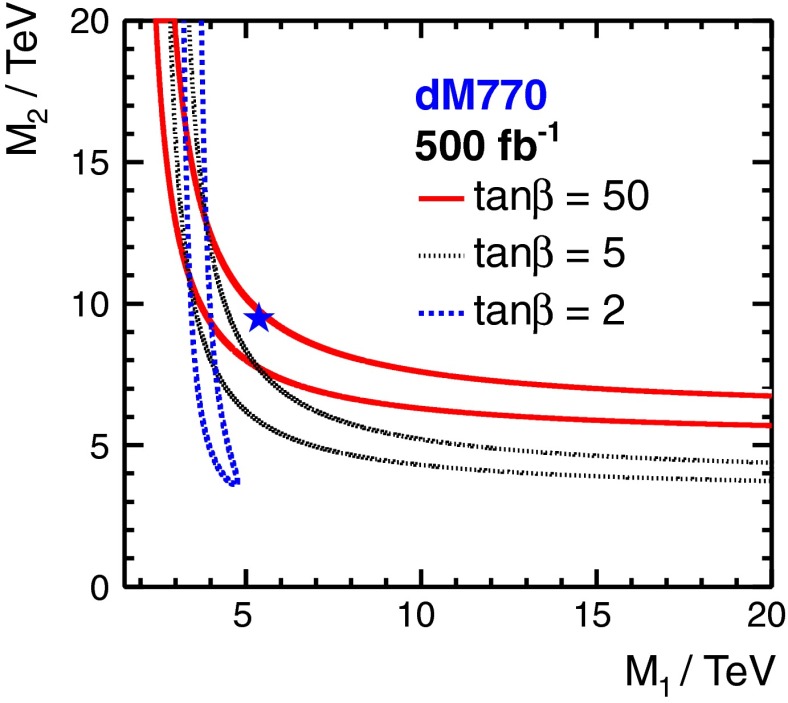
The contours for determination of $M_1$ and $M_2$ in scenario with $m_{\tilde{\chi }^\pm _1} - m_{\tilde{\chi }^0_1} = 770$ MeV. The *star* denotes input values. See Ref. [1223] for more details

The fundamental MSSM parameters $M_1$, $M_2$, $\mu$ and $\tan \beta$ can be extracted from these types of observables. For the specific benchmarks chosen, the $\mu$ parameter can be determined to $\pm$4 %. For the gaugino mass parameters, $M_1$ and $M_2$, the lower bounds can be set in the multi-TeV range, depending on the value of $\tan \beta$, which cannot be fixed from the above measurements alone, see Fig. 139. If the uncertainties could be reduced by a factor of 2 by including additional observables or increasing the integrated luminosity, the constraints on gaugino mass parameters would be significantly more restrictie and less dependent on $\tan \beta$.

#### Parameter fits

The determination of SUSY parameters in global fits using hypothetical measurements at the ILC has been studied in detail [1224] using the Fittino [1225] package for model points such as SPS1a’ [881]. However, this point is now excluded from generic searches for SUSY at the LHC (see e.g. [1220, 1226] for early exclusions). Since then, no new complete analysis have been preformed for parameter determinations in global fits using data from low-energy precision experiments, cosmological measurements, Higgs mass and rate measurements, up-to-date LHC constraints on SUSY production, and hypothetical ILC measurements. Therefore, in this section we revert to the existing SPS1a’ results, keeping in mind that measurements of SUSY production properties at a currently realistic SUSY point would be less favourable both for the LHC and for the ILC. The reason is that the higher mass scale of first- and second-generation squarks and gluinos very strongly reduces the statistics in potential SUSY cascade decay signatures at the LHC. At the same time, given the current LHC bounds, the resolution of the small mass splittings between particles in the cascade decays typically required to allow light gauginos and sleptons at $m_{\tilde{\ell },\tilde{\chi }}\le 250$ or 500 GeV is more challenging, however, yet possible at the ILC.

**Table 34:** Result of the fit of the CMSSM model to the precision measurements and to the hypothetical results from LHC with ${\mathscr {L}}^{\mathrm {int}}=300\,\mathrm {fb}^{-1}$ and ILC

Parameter	Nominal value	Fit	LHC uncertainty	ILC uncertainty
$\tan \beta$	10	9.999	$\pm$0.36	$\pm$0.050
$M_{1/2}$ (GeV)	250	249.999	$\pm$0.33	$\pm$0.076
$M_0$ (GeV)	100	100.003	$\pm$0.39	$\pm$0.064
$A_0$ (GeV)	$-$100	$-$100.0	$\pm$12.0	$\pm$2.4

As a relative comparison between the possible LHC and LHC$+$ILC performance, either SUSY models constrained at the GUT scale (such as the CMSSM) or models defined at the TeV scale can be used. The CMSSM results from [1224] are shown in Table 34. The LHC result is based on actual precision measurements from *B*-factories and on $(g-2)_{\mu }$, on the neutralino relic abundance ${\varOmega }_{\mathrm{CDM}}h^2$, on LEP1 SM precision measurements, and on hypothetical LHC measurements of the Higgs mass and of kinematical quantities measured in SUSY cascade decays. For a detailed list see [1224]. For the ILC, realistically modelled studies of Higgs mass, cross section and branching fraction measurements, hypothetical measurements of kinematical edges in SUSY decays, and a large amount of measurements of cross section times branching fractions for every kinematically accessible SUSY decay chain at sufficient rate is assumed. A time-consuming running scenario with measurements at $\sqrt{s}=400,500$ and 1000 GeV at different combinations of beam polarisations is employed to disentangle the mixing of the gauginos and heavy sleptons.

The results in Table 34 clearly show a significant improvement by a factor of about 5 between the LHC results and the same fit but now including additional ILC information. However, an even stronger improvement is observed when moving towards a SUSY model with significantly more freedom in the parameter choice. One possibility is the pMSSM. Here, a minimal flavour-violating MSSM with unification in the first two generations is constructed at the TeV scale, here called the MSSM18. The value $m_{t}$ is kept fixed due to the high expected accuracy at the ILC. This is a very favourable assumption for the LHC, because for a fit without information on $m_{t}$ from the ILC, the parametric uncertainties – especially on the Higgs mass – would be expected to degrade the precision of the fit result from the LHC. For details on the model, see [1224] again.

**Fig. 140:**
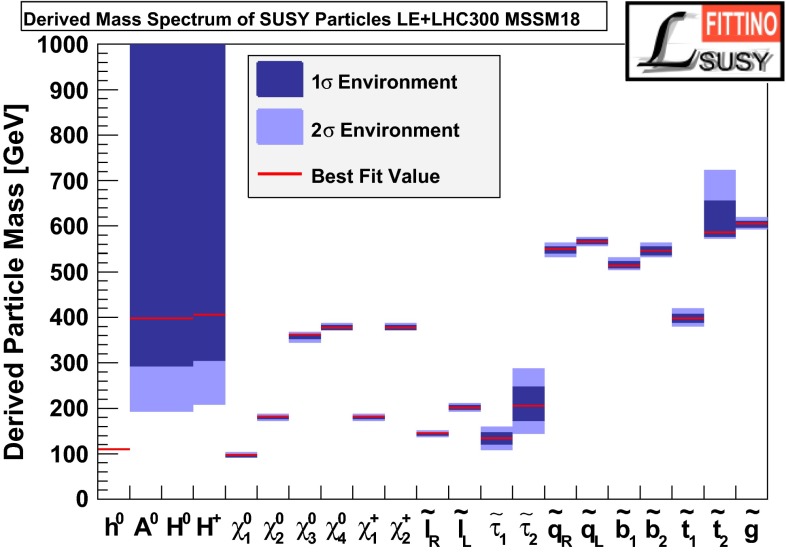
SUSY mass spectrum consistent with the existing low-energy measurements and the hypothetical LHC measurements at ${\mathscr {L}}^{\mathrm {int}}=300\,\mathrm {fb}^{-1}$ for the MSSM18 model. The uncertainty ranges represent model dependent uncertainties of the sparticle masses and not direct mass measurements

**Fig. 141:**
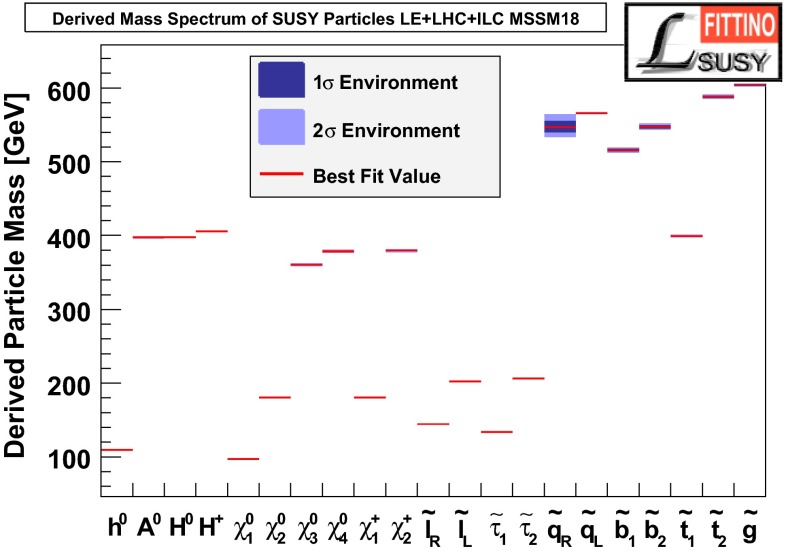
Derived mass distributions of the SUSY particles using low-energy measurements, hypothetical results from LHC with ${\mathscr {L}}^{\mathrm {int}}=300\,\mathrm {fb}^{-1}$ and hypothetical results from ILC. When comparing to Fig. 140, please note the difference in the scale

For a graphical comparison of the power of the ILC at a very favourable, albeit now excluded model point, see the difference between the LHC precision of a model-dependent determination of a SUSY mass spectrum in Fig. 140 and the corresponding spectrum for the added ILC information in Fig. 141. An enormous improvement is observed in the heavy Higgs sector, stemming from the hypothetical direct measurements of the heavy Higgs bosons at the ILC, while they would have remained inaccessible at the LHC. Also for the other masses, improvements of a factor of 10 to 100 are possible [1224]. For the SPS1a’ like MSSM18, the added benefit of the ILC over the LHC is much more apparent due to the larger freedom in the model. For a model with only four free parameters, such as the CMSSM, a few measurements with relatively good precision are enough to constrain the parameters in a reasonable range, such as for the LHC in the hypothetical SPS1a’ CMSSM. However, once the less accessible states decouple from the more accessible ones, such as in the MSSM18, the direct information on states like the light ${\textit{CP}}$-even Higgs boson *h* and the squark mass scales does not suffice to constrain less accessible states anymore (like the heavy Higgses) since they are controlled by additional parameters like $m_A$ and $X_{f}$ in the MSSM18: these cannot easily be accessed otherwise. At the $e^+e^-$ LC, however, the high-precision measurements of the full Higgs sector (as for SPS1a’) and the very high-precision measurements of sparticle masses and couplings, would have allowed one to disentangle the mixings and mass parameters in the gaugino, the heavy slepton and the stop sector individually. Such determinations reduce the model dependence dramatically and improve the fit precision accordingly, by providing independent precise probes of all degrees of freedom of the model.

#### Extrapolation to GUT scale

As discussed in Sect. 5.2, many of the commonly used SUSY models impose strong assumptions at the high scale inspired by suppositions on the SUSY-breaking mechanism. In the CMSSM – with the input parameters $m_0$, $m_{1/2}$, $\tan \beta$, $A_0$, sign$\mu$ at the GUT scale $M_\mathrm{GUT}\approx 2\cdot 10^{16}$ GeV – all gauge couplings $\alpha _{1,2,3}$ and also all gaugino masses $M_{1,2,3}$ and scalar masses unify at $M_\mathrm{GUT}$.

**Fig. 142:**
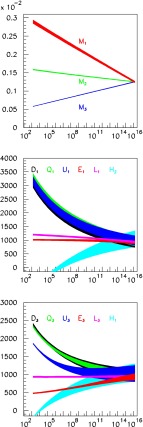
Evolution of gaugino and sfermion (first and third generation) parameters in the CMSSM for $m_0=966$ GeV, $m_{1/2}=800$ GeV, $A_0=0$, $\tan \beta =51$, sign$\mu =+1$[9, 10] to the GUT scale

Generally, in order to test such model-dependent assumptions, one can start from a precisely measured particle spectrum at lower energies and extrapolate the underlying parameter to higher energies, up to $M_\mathrm{GUT}$, as described in [1198]. The evolution of the parameters happens via applying the renormalisation group equations (RGEs). In practically all studies, it is assumed to combine measurements of the non-coloured spectrum at the LC with measurements of the coloured spectrum at the LHC.

As one example, we choose benchmark ‘Model I’ from Refs. [9, 10] with the GUT scale parameters $m_0=966$ GeV, $m_{1/2}=800$ GeV, $A_0=0$, $\tan \beta =51$, sign$(\mu )=+1$, which determine the particle spectrum at low energy. In [9, 10], it has been shown that the masses of neutralinos and the sleptons of the first two generation can be measured with a precision of 1–2% at a 3-TeV collider. In addition, one assumes to measure the gluino mass $m_g=1812$ GeV with 5 % precision at the LHC and at a 3 % level for all other sfermion masses at the LC. Based on the mass and cross section measurements of the neutralino/chargino sector, one can reconstruct the quantities at tree level: $M_1$, $M_2$, $\mu$ and $\tan \beta$.

Since we measure on-shell masses, but use $\overline{DR}$ parameters for the evolution of parameters, the corresponding shifts must be calculated. This intertwines the different sectors: naively one would expect that the relative precision of the masses transfers one to one to the precision on the gaugino mass parameters. However, in case of the gluino mass parameters, the uncertainty due to the squark mass measurements can increases the uncertainty on $M_3$ by up to a factor 2, e.g. instead of a 5 per-cent uncertainty one obtains roughly a ten per-cent uncertainty. At the level of one-loop RGEs, the relative uncertainties are approximately scale invariant as at this level $M_i/\alpha _i$ is an RGE invariant. However, at the two-loop level, also the tri-linear *A*-parameters of the third generation enter and, thus, one should know them to a precision of at least 40 % as otherwise the uncertainties at the high scale can be significantly worse compared to the one at the electroweak scale. The tri-linear couplings can be determined via cross-section measurements and sfermion decays involving Higgs bosons (or decays of heavy Higgs bosons into sfermions) [910, 1198]. Under the above assumption, we find a unification of the gaugino mass parameters to about 10 %; see Fig. 142 (top panel).

In the evaluation of the sfermion mass parameters, also the gaugino mass parameters enter where in particular $M_3$ is important for the evolution of the squark mass parameters. In the case of third-generation sfermions and the Higgs mass parameters, also large Yukawa couplings as well as the *A*-parameters enter the RGEs and intertwine them in a non-trivial way. Taking the same assumptions as above, we find a clear overlap between all scalar mass parameters when running up to the GUT scale, see Fig. 142 (middle and bottom panel), pointing clearly to the 1000 GeV region for $m_0$.

### Lepton flavour and ${\textit{CP}}$ violation

The general structure of supersymmetry admits several possible extensions to the MSSM, either by switching on new couplings or introducing new parameters, such as ${\textit{CP}}$-violating phases or adding new fields, each resulting in new, specific phenomenology. Because of its versatility and the limited SM backgrounds, a linear collider is best suited to investigate these scenarios. In this section, we review the sources of lepton flavour and ${\textit{CP}}$ violation in extended SUSY models and their phenomenology in $e^+e^-$ collisions.

#### Lepton flavour violation

A significant body of data from atmospheric, solar, reactor and accelerator neutrino experiments [1227–1234] have revealed the non-zero value of neutrino masses and oscillations with near-maximal $\nu _\mu$–$\nu _\tau$ and large $\nu _e$–$\nu _\mu$ mixing. A very attractive explanation for the smallness of neutrino masses and their mixings is a seesaw mechanism embedded within the framework of SUSY models. In this case [1235–1237], masses and mixings in the neutrino system are caused by very heavy right-handed Majorana neutrinos with masses close to the GUT scale. Even if the sfermion mass matrices are diagonal at the GUT scale, flavour-violating mixings are induced radiatively [1238, 1239]. A substantial $\nu _\mu$–$\nu _\tau$ mixing leads to large $\tilde{\mu }_L$–$\tilde{\tau }_L$ and $\tilde{\nu }_\mu$–$\tilde{\nu }_\tau$ mixings. It is natural to expect that charged-lepton flavour violation (cLFV) should occur at some level thus raising the interesting possibility of observing these processes in low-energy rare decays $\mu \rightarrow e \gamma$, or $\tau \rightarrow \mu \gamma$, or $\mu$–*e* conversion [1240–1243] and at a high-energy $e^+e^-$ collider.

In the standard model, cLFV processes are strongly suppressed due to the GIM mechanism. However, in SUSY, virtual superpartner loops may provide an enhancement [1242, 1243] making them observable. Moreover, if sleptons are directly produced, cLFV can also be directly tested in their production and decay processes. For nearly degenerate sleptons, supersymmetric LFV contributions to low-energy rare decay processes are suppressed as ${\varDelta } m_{\tilde{\ell }}/m_{\tilde{\ell }}$ through the superGIM mechanism and constraints from the yet unobserved radiative decays $\ell _i\rightarrow \ell _j\gamma$ are not very stringent. On the other hand, in direct decays of sleptons, this kind of supersymmetric lepton flavour violation is suppressed only as ${\varDelta } m_{\tilde{\ell }}/{\varGamma }_{\tilde{\ell }}$ [1244, 1245]. Since $m_{\tilde{\ell }}/{\varGamma }_{\tilde{\ell }}$ can be large, spectacular signals may be expected leading to possible discoveries at the LHC and in particular at future lepton collider experiments. Among the possibilities considered so far, there is slepton pair production at a linear collider as well as signals from electroweak gaugino production and their subsequent cascade decays $\tilde{\chi }^0_2\rightarrow \tilde{\chi }^0_1 + e^\pm \mu ^\mp$, $\tilde{\chi }^0_2\rightarrow \tilde{\chi }^0_1 + \mu ^\pm \tau ^\mp$, at both a linear collider and the LHC [1244–1259].

At a LC, the cLFV signals can be looked for directly in slepton pair production, for example

155 $$\begin{aligned}&e^+e^- \rightarrow \tilde{\ell }^-_i\tilde{\ell }^+_j \rightarrow \tau ^+\mu ^- \tilde{\chi }^0_1 \tilde{\chi }^0_1, \nonumber \\&e^+e^- \rightarrow \tilde{\nu }_i\tilde{\nu }^c_j \rightarrow \tau ^+\mu ^- \tilde{\chi }^+_1 \tilde{\chi }^-_1 \end{aligned}$$

or indirectly via sleptons produced singly in chain decays of heavier charginos and/or neutralinos $\tilde{\chi }_2\rightarrow \ell _i\tilde{\ell }_j$, $\tilde{\ell }_j\rightarrow \ell _k\tilde{\chi }_1$:

156 $$\begin{aligned}&e^+e^- \rightarrow \tilde{\chi }^+_2\tilde{\chi }^-_1 \rightarrow \tau ^+\mu ^- \tilde{\chi }^+_1\tilde{\chi }^-_1 \nonumber \\&e^+e^- \rightarrow \tilde{\chi }^0_2 \tilde{\chi }^0_1 \rightarrow \tau ^+\mu ^- \tilde{\chi }^0_1\tilde{\chi }^0_1. \end{aligned}$$

With $\tilde{\chi }^\pm _1 \rightarrow \tilde{\chi }^0_1 f\bar{f}'$, and $\tilde{\chi }^0_1$ escaping detection, the signature therefore would be $\tau ^{\pm }\mu ^{\mp }+\mathrm{jets}+ {E\!\!\!/}_T$, $\tau ^{\pm }\mu ^{\mp }+ \ell + {E\!\!\!/}_T$, or $\tau ^{\pm }\mu ^{\mp }+ {E\!\!\!/}_T$, depending on hadronic or leptonic $\tilde{\chi }^\pm _1$ decay mode.

In the case of narrow widths and small mass differences between the sleptons of different generations, ${\varDelta } \tilde{m}_{ij} \ll \tilde{m} = \frac{1}{2}(m_2+m_3)$ and $\tilde{m}\overline{{\varGamma }}_{ij} \simeq (\tilde{m}_i{\varGamma }_i+\tilde{m}_j{\varGamma }_j)/2\ll \tilde{m}^2$, and assuming a pure 2–3 inter-generation mixing between $\tilde{\nu }_\mu$ and $\tilde{\nu }_\tau$, generated by a near-maximal mixing angle $\tilde{\theta }_{23}$, and ignoring any mixings with $\tilde{\nu }_e$,^62^ the cross sections for $\tau ^+\mu ^-$ in the final state simplify considerably  [1244, 1245, 1260]. For $\tau ^+\mu ^-$ produced in the decays of a pair of sleptons, Eq. (156), the cross section can be approximated as

157 $$\begin{aligned} \sigma ^\mathrm{pair}_{23} = \chi _{23}(3-4 \chi _{23}) \sin ^2 2\tilde{\theta }_{23} \times \sigma _0\times Br, \end{aligned}$$

whereas for $\tau ^+\mu ^-$ produced from the gaugino decay, Eq. (156), it takes the form

158 $$\begin{aligned} \sigma ^\mathrm{casc}_{23}=\chi _{23} \sin ^2 2\tilde{\theta }_{23} \times \sigma _0\times Br. \end{aligned}$$

Here the cLFV effect is taken into account by the factors $\sin ^2 2\tilde{\theta }_{23}$ and $\chi _{23} \equiv x_{23}^2/2(1+x_{23}^2)$ where $x_{23} \equiv {\varDelta } \tilde{m}_{23}/\overline{{\varGamma }}_{23}$. The difference between Eqs. (157) and (158) is due to the correlated slepton pair production in the processes Eq. (156). In the above expressions, $\sigma _0$ is the corresponding sparticle pair-production cross section in $e^+e^-$ collision and *Br* is the product of relevant branching ratios for the corresponding decay chains without cLFV contributions.

**Fig. 143:**
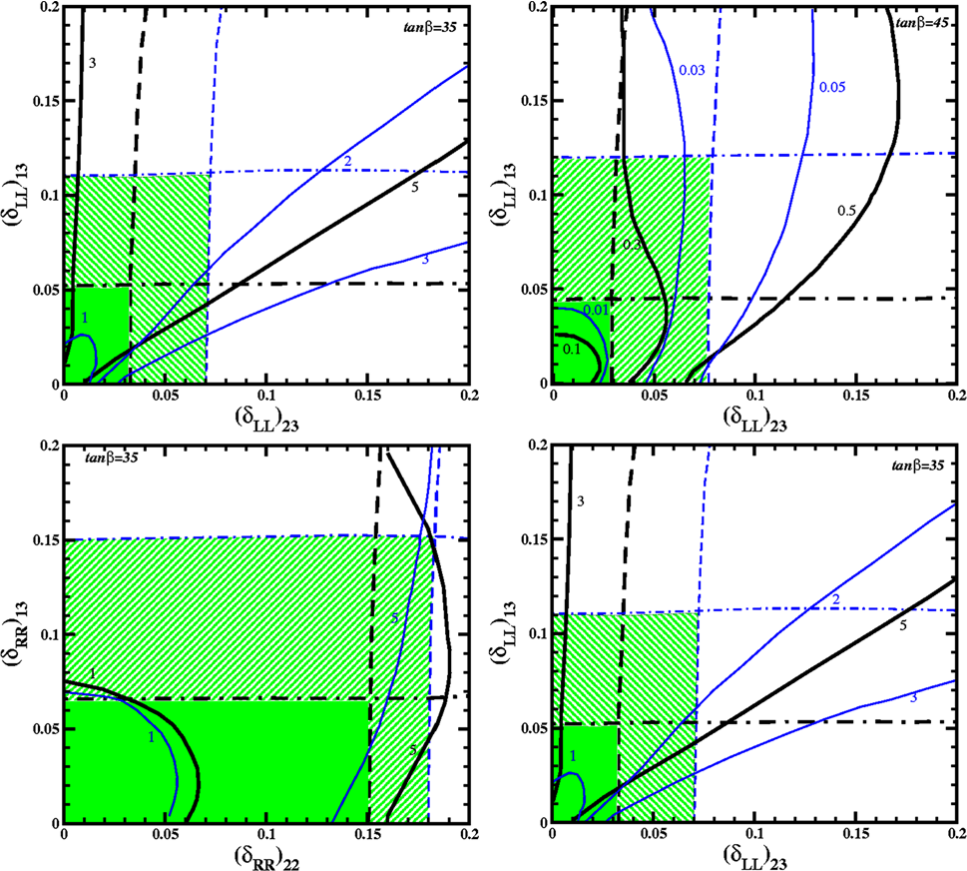
Constraints on the magnitudes of the mixing parameters and possible LFV effects for reference points from [1261]. The *shaded areas* are those allowed by current limits on BR($\tau \rightarrow e \gamma$) (*dot-dash line*) and BR($\tau \rightarrow \mu \gamma$) (*dash line*) using four different reference points (shown by the *thick lines* bounding the *solid shaded* areas and the *thin blue* lines bounding the *ruled shaded* areas). The *solid lines* are contours of $\sigma (e^+e^-\rightarrow \tau ^\pm \mu ^\mp +2 \chi ^0)$ in fb for $\sqrt{s}=2000 \mathrm{~GeV}$

The potential of exploring the cLFV at a LC has recently been revisited in final states with $\tau \mu$ [1261] and $e\mu$ [1262]. Both analyses adopted the cMSSM framework with benchmark points chosen to be consistent with the limits from the LHC searches and cosmological relic LSP density. The benchmarks feature relatively low values of $m_0$ (compared to $m_{1/2}$) to provide a relatively light slepton spectrum accessible at a LC, while avoiding the LHC bounds on the strongly interacting sector. To assess the sensitivity of the cross section measurements to the LFV terms $(\delta _{LL,RR})_{ij}$, where the flavour mixing entries encode the inter-generation elements of the slepton mass matrix $(\delta _{XX})_{ij}=(M^2_{XX})^{ij}/(M^2_{XX})^{ii}$, $(X=L,R)$, Fig. 143 shows current constraints and possible LFV effects for reference points. Despite the SM and SUSY charged-current backgrounds, the expected number of signal events should allow us to probe cLFV in extensive regions of the SUSY seesaw parameter space. Both direct slepton pair production and sleptons produced in cascade decays may provide interesting signals in the cosmologically favoured region of the supersymmetric parametric space. In comparison to the LHC, the LC could provide additional insights by virtue of its greater kinematic range for slepton production and its sensitivity to both RR and LL mixing.

**Fig. 144:**
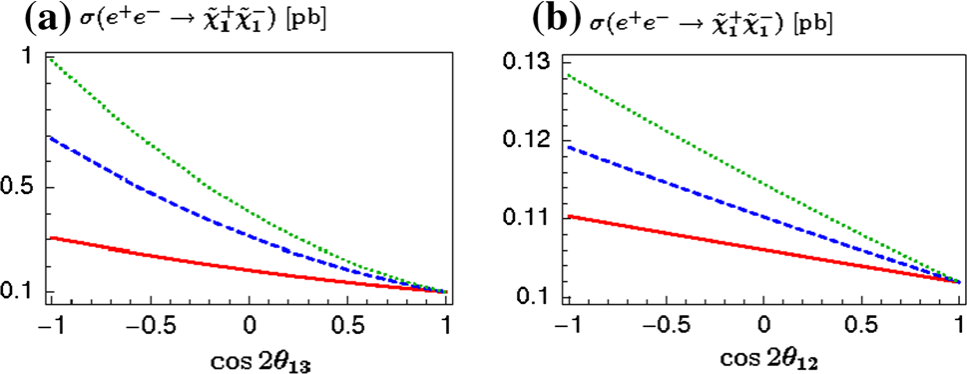
Cross section $\sigma (e^+e^-\rightarrow \tilde{\chi }^+_1\tilde{\chi }^-_1)$ as a function of the mixing parameter $\cos 2\theta _{13}$ (a) and $\cos 2\theta _{12}$ (b) at a LC with cm energy of 500 GeV and polarised beams: $P_L=-0.9$ for electrons and $P_L=0.6$ for positrons. Details of assumed scenarios (a) and (b) are in [1254]

Lepton flavour violation can also reveal itself in other processes such as $e^+e^-\rightarrow \tilde{\chi }^+_i\tilde{\chi }^-_j$. This process proceeds through *s*-channel $\gamma /Z$ and also *t*-channel $\tilde{\nu }_e$ exchange. In the LFV scenario, the $\tilde{\nu }_e$ is a mixture of three mass eigenstates. The production cross section for chargino pair production may change by a factor of 2 or more in the presence of $\tilde{\nu }_e$–$\tilde{\nu }_\tau$ mixing even if current bounds on LFV rare lepton decays are significantly improved (see Fig. 144) [1254]. The effect of $\tilde{\nu }_e$–$\tilde{\nu }_\mu$ mixing, due to stronger experimental bounds, is less dramatic, as seen in the right panel of Fig. 144.

#### ${\textit{CP}}$ violation

Since the first observation of ${\textit{CP}}$ violation almost 50 years ago, the “cryptic message from nature” it conveys still needs to be deciphered in full. An attractive feature of SUSY is that it allows for new sources of ${\textit{CP}}$ violation which are needed if the baryon-antibaryon asymmetry observed in the universe is to be explained by particle physics. Compared to the case of ${\textit{CP}}$-conserving SUSY, new ${\textit{CP}}$ phases appearing in supersymmetry may change masses, cross sections, decay branching ratios, etc. providing many possible ways to detect and measure them at colliders. Since such observables are ${\textit{CP}}$-even, ${\textit{CP}}$-violating effects may be distinguished from fortuitous combinations of parameters not invoking ${\textit{CP}}$-violating phases only by the joint analyses of several ${\textit{CP}}$-even observables. For example, an observation of s-wave excitation above respective thresholds of three non-diagonal pairs of neutralinos [43, 44], or the observation of simultaneous sharp s-wave excitations of the production cross section $\sigma (e^+e^- \rightarrow \tilde{\chi }^0_i\tilde{\chi }^0_j)$ ($i \ne j$) near threshold and the $f\bar{f}$ invariant mass distribution near the end point of the decay $\tilde{\chi }^0_i\rightarrow \tilde{\chi }^0_j f\bar{f}$ [1263] is qualitative, unambiguous evidence for ${\textit{CP}}$ violation in the neutralino system. A linear collider of sufficient energy can perform all these measurements.

**Fig. 145:**
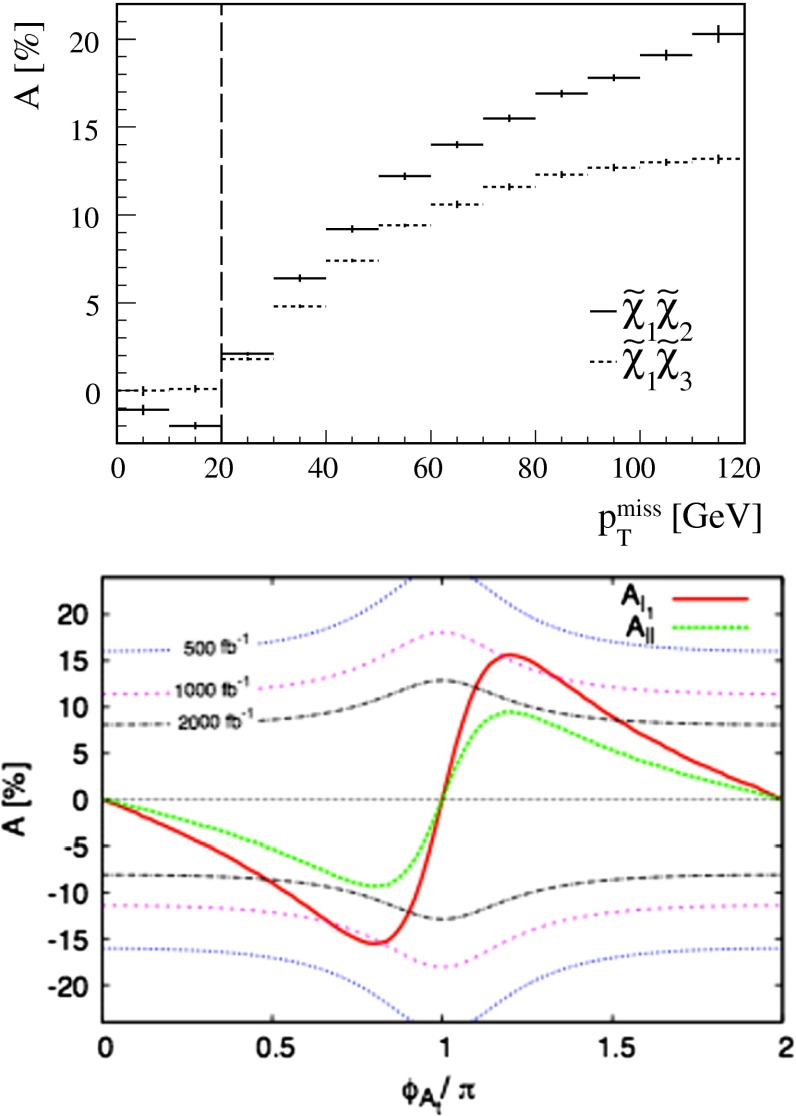
*Top panel* $p^\mathrm{miss}_T$ dependence of ${\textit{CP}}$ asymmetries in neutralino-pair production and decay processes (from [1165]). *Bottom panel* asymmetries, $A_{\ell _1}$ and $A_{\ell \ell }$ as functions of $\Phi _{A_t}$ (from [1270])

The most direct way to detect ${\textit{CP}}$-violation is to construct ${\textit{CP}}$-odd observables which cannot be mimicked by other parameters of the theory. Such quantities typically involve asymmetries constructed as triple products of momenta and/or spin vectors. Due to spin correlations, such asymmetries show unique hints for ${\textit{CP}}$ phases already at tree level. Triple product asymmetries have been proposed in many theoretical papers in which neutralino production with two- and three-body decays, charginos with two- and three-body decays, also with transversely polarised beams, have been studied in the past [1264–1269]. At tree level, the neutralino and chargino sector has two independent ${\textit{CP}}$ phases: for instance of $M_1$ and $\mu$ when rotating away the phase of $M_2$. Assuming the phase of $\mu$ – strongly constrained by EDM bounds – to be small, the phase of $M_1$ could lead to ${\textit{CP}}$ sensitive triple product asymmetries of up to 20 %; see Fig. 145. As mentioned above, a recent analysis performed with full event simulation and reconstruction [1165] shows that these asymmetries constructed from $(\mathbf {p}_{e^-}\times \mathbf {p}_{\ell ^+_N})\cdot \mathbf {p}_{\ell ^-_F}$ can be measured to $\pm 1$ % from neutralino two-body decays into slepton and lepton followed by slepton decay: $\tilde{\chi }^0_j\rightarrow \tilde{\ell }^-\ell ^+_N\rightarrow \tilde{\chi }^0_1\ell ^-_F\ell ^+_N$. From a fit to the measured neutralino cross sections, masses and ${\textit{CP}}$-asymmetries, $|M_1|$ and $|\mu |$ can be determined to a few per-mille, $M_2$ to a few per-cent, $\Phi _1$ to 10 % as well as $\tan \beta$ and $\Phi _\mu$ to 16 and 20 %, respectively.

The sfermion sector brings in the ${\textit{CP}}$ phase of the tri-linear scalar coupling $\Phi _{A}$. The sensitivity of the linear collider to the ${\textit{CP}}$ phase in the stop sector has been looked at recently [1270] by analysing a chain decay $\tilde{t}_1\rightarrow \tilde{\chi }^0_2 (\rightarrow \tilde{\chi }^0_1\ell ^{\mp }_N \ell _F^\pm )\, + t (\rightarrow W^+b)$. Such decays allow one to construct two triple products originating from the covariant product in the spin–spin-dependent part of the amplitude, namely $A_{\ell _1}\sim \mathbf {p}_{\ell _1^\mp } \cdot ( \mathbf {p}_W\times \mathbf {p}_t)$ calculated in the reconstructed $\tilde{\chi }^0_2$ rest frame, and $A_{\ell \ell }\sim \mathbf {p}_b \cdot (\mathbf {p}_{\ell ^+} \times \mathbf {p}_{\ell ^-})$ calculated in the reconstructed *W* rest frame. The right panel of Fig. 145 shows that ${\textit{CP}}$ sensitive asymmetries can reach 10–15 %. Under the assumption of accurate momentum reconstruction, this asymmetry could be measured for 2 $ab^{-1}$ (1 $ab^{-1}$) of data collected at $\sqrt{s}=1$ TeV in the region of a maximal ${\textit{CP}}$-violating angle, $1.10 \pi < \Phi _{A_t} < 1.5\pi$ ($1.18\pi < \Phi _{A_t} < 1.33\pi$).

Finally, it is worthwhile to recall that the ${\textit{CP}}$-odd observables can also be constructed in the non-diagonal chargino pair production process $e^+e^-\rightarrow \tilde{\chi }^\pm _1 \tilde{\chi }^\mp _2$ from unpolarised cross sections at one loop [1271, 1272]. Obviously, at tree level the ${\textit{CP}}$-asymmetry $A^{12}\sim \int [\mathrm{d}\sigma (\tilde{\chi }^-_1 \tilde{\chi }^+_2)-\mathrm{d}\sigma (\tilde{\chi }^-_2 \tilde{\chi }^+_1)]\mathrm{d}\cos \theta$ (with the polar angle $\theta$ of $\tilde{\chi }^-_j$ with respect to the $e^-$ momentum direction) vanishes even in ${\textit{CP}}$-non-invariant theories. In order to obtain a non-zero asymmetry in the chargino production it requires another source of non-trivial imaginary contributions to the amplitude. Such a term can be generated by the absorptive part of a loop diagram when some of the intermediate state particles in loop diagrams go on-shell. The ${\textit{CP}}$-odd asymmetry is generated due to interference between the imaginary part of the loop integrals and imaginary parts of the couplings. Numerical analyses show that the asymmetries can be of the order of a few per-cent and in principle might be measurable, allowing for discovery of the ${\textit{CP}}$-violating phases via simple event counting experiments.

### Beyond the MSSM

#### The NMSSM

The supersymmetric $\mu$ problem arises because the higgsino mass $\mu$ term in the MSSM superpotential is not a SUSY-breaking term, but instead preserves SUSY. Thus, naively one would expect $\mu \sim M_P$ instead of $M_{\mathrm{weak}}$; this possibility seems phenomenologically disallowed. One solution, endemic to gravity mediation, is for the $\mu$ term to be forbidden by some symmetry, such as a Peccei–Quinn (PQ) symmetry, but then to re-generate it via interactions with either the PQ sector [1273] or the hidden sector [1274]. An alternative possibility occurs by extending the MSSM with an additional gauge singlet superfield *N*, where the $\mu$ term then arises from its coupling to the Higgs fields in the superpotential, $\lambda N H_uH_d$. This extension is known as the next-to-minimal SUSY extension of the SM, or NMSSM. In the NMSSM, an effective $\mu =\lambda x$ term is expected to be generated around the electroweak scale when the scalar component of the singlet *N* acquires a vacuum expectation value $x=\langle N\rangle$. Moreover, the NMSSM is additionally motivated in that it provides additional quartic contributions to the light Higgs scalar mass $M_h$, thus perhaps more easily accommodating the rather large value $M_h\sim 125$ GeV, which otherwise requires TeV-scale top squarks, which some authors consider to have a conflict with naturalness. Further reduction in the fine tuning of the NMSSM can be achieved by introducing extra matter terms [1275]. Independently, a bottom-up approach for addressing the fine-tuning problem, via “natural SUSY”, calls for the third-generation sfermions and the higgsino to be light, while the rest of the superpartners can be heavy. However, the higgsino cannot then be the sole DM candidate since higgsinos annihilate too rapidly into *WW* and *ZZ*.

**Fig. 146:**
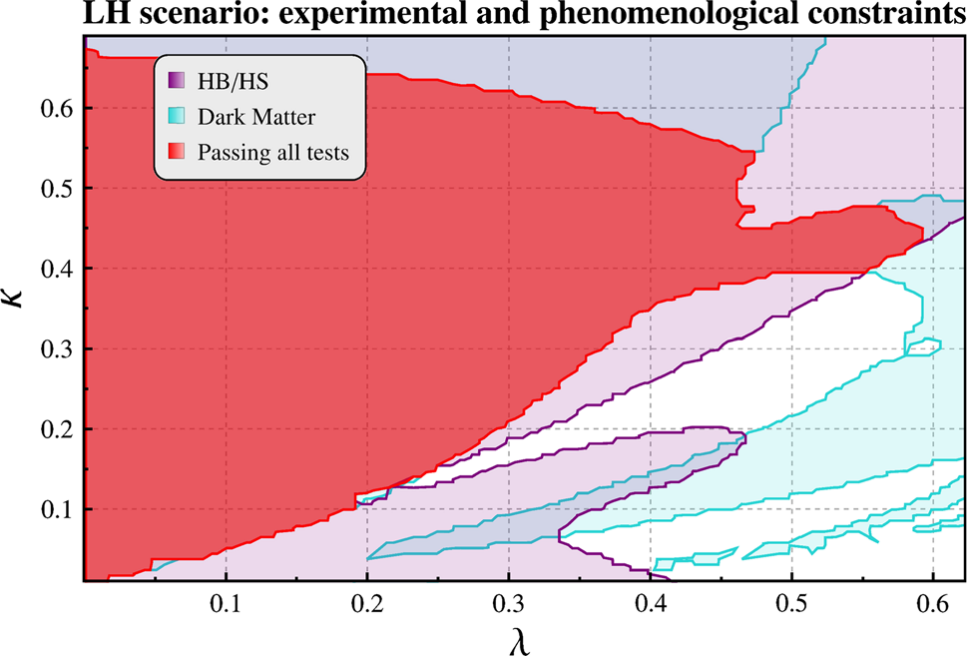
Lightest neutralino $\tilde{\chi }^0_1$ is mainly higgsino-like: regions in the ($\lambda$–$\kappa$)-plane allowed by experimental and phenomenological constraints. The *light-blue-shaded regions* delimited by the *light-blue boundary* pass DM constraints. The *coloured regions* delimited by the *purple boundary* pass checks within HiggsBounds [1276] and HiggsSignals [1277]. The *red area* is allowed by all the constraints [1278]

Within the extended Higgs sector of the NMSSM, the new singlino state, with mass below that of the higgsino, might serve as a DM particle, or the LSP might have a significant singlino component. The phenomenology of different scenarios for the mixing character of the lightest neutralino – singlino, higgsino, gaugino-like – has been systematically analysed in the plane of the NMSSM-specific Yukawa couplings $\lambda -\kappa$-plane, cf. also Fig. 146.

**Fig. 147:**
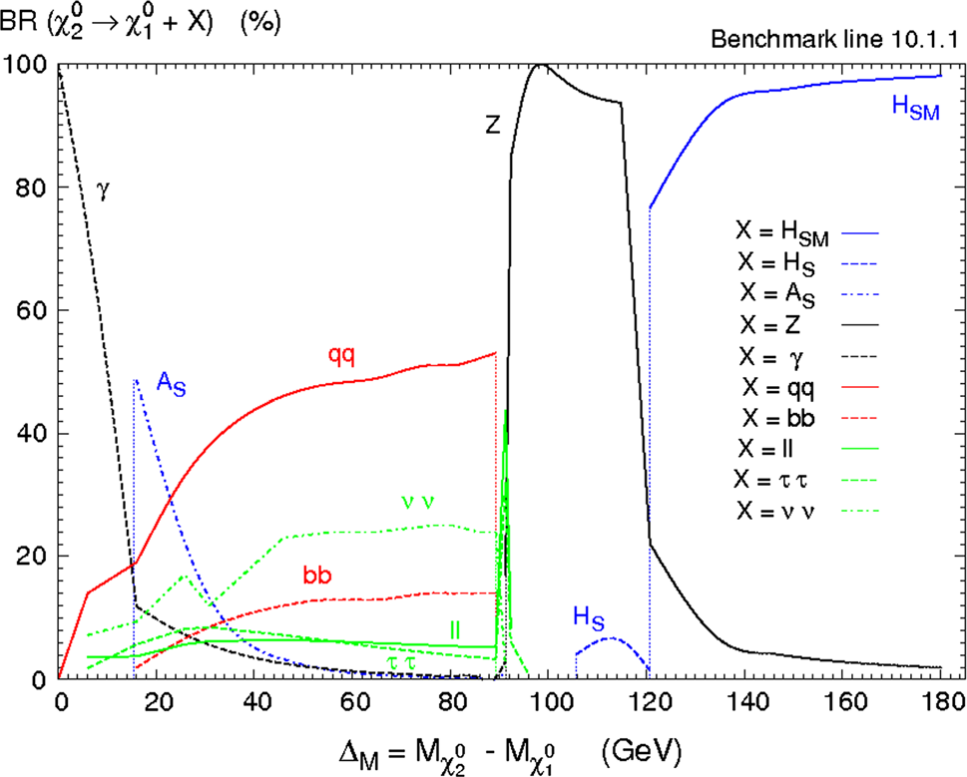
Neutralino decay $\tilde{\chi }^0_2\rightarrow \tilde{\chi }^0_1 + X$ branching fractions as function of the mass splitting ${\varDelta } M = M_{\chi ^0_2} - M_{\chi ^0_1}$ (from [1279])

In the first case, the decay width of the higgsino to the singlino is of order 100 MeV. The pattern of decays can be rich (see Fig. 147), providing us with clear signatures which can be studied at a LC of sufficient energy. The precision measurement of these decay branching ratios will illuminate the structure of the extended model [1279]. These decay products are quite soft, however, and are expected to be virtually invisible under the standard LHC trigger conditions. Whether or not these particles can be seen at the LHC, the linear collider would again be needed for a complete study, which requires the determination of their branching fractions. The singlino–higgsino mixing angle, which determines the annihilation cross section of the LSP and the thermal DM density, could be measured at the LC through a determination of the higgsino width using a threshold scan, as discussed above, or by precision measurements of the NMSSM mass eigenvalues.

The LC capabilities in distinguishing between the NMSSM and the MSSM, when the observable particle spectrum and the corresponding decay chains are very similar in pattern, has been studied in detail [43, 44, 502]. From data taken in $e^+e^-$ collisions at three different centre-of-mass energies, the distinction is possible. When exploiting the available information by applying a global fit, just two $\sqrt{s}$ choices can be sufficient, depending on the mixing character of the lightest neutralino states [502, 1278]. If the full neutralino/chargino spectrum is accessible at the maximum collider energy, sum rules for the production cross sections, yielding a different energy behaviour in the two models, may also be exploited. In scenarios with dominant couplings of a mostly singlino LSP to the NLSP particle, as predicted for large values of the *x* parameter, the existence of displaced vertices leads to a particularly interesting signature that can be precisely resolved with the excellent detector resolution envisaged at a linear collider.

#### *R*-Parity violation

The signatures for the SUSY searches discussed so far are based on the assumption that *R*-parity, the additional quantum number distinguishing SUSY particles from their SM counterparts, is conserved leading to final states with significant missing energy, due to the escaping LSPs. Introducing *R*-parity violation (RpV) changes drastically the SUSY phenomenology. *R*-parity-violating couplings allow for single production of SUSY particles and their decays to SM particles. The latter aspect makes RpV SUSY much harder to detect at the LHC due to the absence of MET, so that the currently explored region is significantly smaller than in the *R*-parity-conserving case, even when assuming mass unification at the GUT scale [1280]. Although the LSP is not stable, there are models with small *R*-parity violation which naturally yield a consistent cosmology incorporating primordial nucleosynthesis, leptogenesis and gravitino DM [1281]; axion DM is also a possibility. Since the gravitino decays into SM particles are doubly suppressed by the Planck mass and the small *R*-parity breaking parameter, its lifetime can exceed the age of the universe by many orders of magnitude, and the gravitino remains a viable DM candidate [1282].

Bi-linear *R*-parity violation (BRpV) has phenomenological motivations in neutrino mixing [53] as well as in leptogenesis [1283, 1284]. In this case, the mixing between neutrinos and neutralinos leads to one massive neutrino at tree level and the other two via loop effects [1285–1287]. Once the parameters are adjusted to satisfy the neutrino constraints, the lightest neutralino typically decays inside the detector volume [53]. Since the parameters that determine the decay properties of the LSP are the same parameters as that lead to neutrino masses and oscillations, there are strong correlations between the neutralino branching ratios and the neutrino mixing angles, e.g.,

159 $$\begin{aligned} \mathrm{BR}(\tilde{\chi }_1^0 \rightarrow W^\pm \mu ^\mp ) /\mathrm{BR}(\tilde{\chi }_1^0 \rightarrow W^\pm \tau ^\mp ) \sim \tan ^2\theta _{23}. \end{aligned}$$

**Fig. 148:**
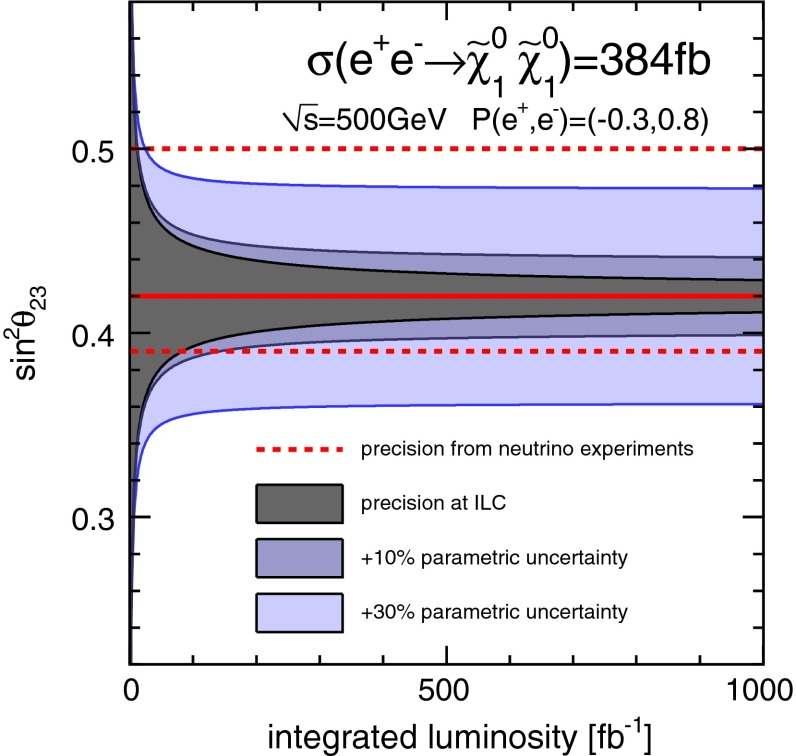
Achievable precision on $\sin ^2\theta _{23}$ from BRpV decays of the $\tilde{\chi }^0_1$ as a function of the produced number of neutralino pairs compared to the current precision from neutrino oscillation measurements. Over a large part of the $m_{1/2}$ vs. $m_0$ plane, the neutralino-pair production cross section of the order of 100 fb [52]

By measuring the ratio of the branching fractions for $\tilde{\chi }^0_1 \rightarrow W^\pm \mu ^\mp$ and $W^\pm \tau ^\mp$, the neutrino mixing angle $\sin ^2 \theta _{23}$ could be determined to per-cent-level precision, as illustrated in Fig. 148. The characteristic decay $\tilde{\chi }^0_1\rightarrow W^\pm l^\mp$ gives background-free signatures at an $e^+e^-$ linear collider, possibly with a detectable lifetime of the $\tilde{\chi }^0_1$ depending on the strength of the BRpV couplings. In the hadronic decay mode of the $W^\pm$, these events can be fully reconstructed and the $\tilde{\chi }^0_1$ mass can be measured to $\mathscr {O}$(100) MeV depending on the assumed cross section [52]. The LC results could then be checked against the measurements from neutrino oscillation experiments to prove that BRpV SUSY is indeed the origin of the structure of the neutrino sector.

**Fig. 149:**
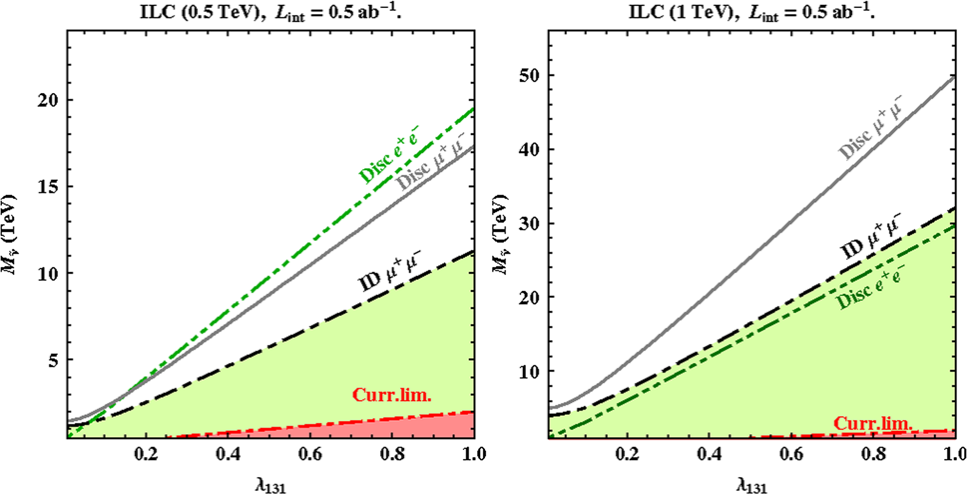
Discovery reach at 95 % CL in Bhabha scattering for the sneutrino mass as a function of $\lambda _{131}$ at $\sqrt{s}=0.5~\mathrm{TeV}$ (*left panel*) and 1 TeV (*right panel*), for $\mathcal{L}_\mathrm{int}=0.5~\mathrm{ab}^{-1}$. For comparison, the discovery reach on $M_{\tilde{\nu }}$ in muon pair production for $\lambda _{232}=0.5\times M_{\tilde{\nu }}/\mathrm{TeV}$ is also shown (from [1293])

Finally, in the case of trilinear *R*-parity violation (TRpV), the exchange of sparticles can contribute significantly to SM processes and may even produce peak or bump distortions to the distribution of cross sections [1288–1290]. Below threshold, these new spin-0 exchanges may manifest themselves via indirect effects on observables such as cross sections and asymmetries which can be precisely measured in $e^+e^-$ collisions, including spectacular decays [1291]. It has been shown recently that the observed enhancement of the semileptonic and leptonic decay rates of $B \rightarrow \tau \nu$ modes can be explained in the framework of TRpV [1292]. However, in such cases it would be important to identify the actual source among the possible non-standard interactions as many different new physics scenarios may lead to very similar experimental signatures. At the LC, a technique based on a double polarisation asymmetry formed by polarising both beams in the initial state has been proposed [1293]. This is particularly suitable to directly test for *s*-channel $\tilde{\nu }$ exchange. Again, the availability of both $e^-$ and $e^+$ polarisation plays a crucial rôle in identifying the new physics scenario (see Fig. 149). In contrast, the left–right asymmetry, $A_{\mathrm{LR}}$, obtained with only electron polarisation, does not appear to be useful for this purpose.

#### *R* symmetry

In the *R*-parity-conserving MSSM, the gravitino, gluino, and other gaugino mass terms can be introduced once supersymmetry is broken. However, it has recently been realised that requiring an additional *R*-symmetry [1294–1297] beyond *R*-parity, which can be continuous or discrete, exact or approximate, is not only phenomenologically viable, but may allow sizeable flavour-violating operators without generating large FCNC or ${\textit{CP}}$ violation. A continuous $U(1)_R$ symmetry on the MSSM, where gauginos and squarks have *R*-charges $R= +1$, and the Higgs scalars have $R = 0$, not only forbids baryon- and lepton-number changing terms in the superpotential, but also dimension-five operators mediating proton decay [926, 927].

*R* symmetry also removes some of the potentially unwanted parameters of the theory, such as tri-linear *A*-terms for the scalars, the $\mu$-term and Majorana gaugino masses, while Majorana neutrino masses are allowed. The absence of $\mu$ and *A* terms helps to solve the flavour problem without flavour-blind mediation. However, since gauginos must get masses, adjoint chiral super-fields for each gauge factor are introduced to generate *R*-symmetry preserving Dirac gaugino masses. Similarly, the Higgs sector is extended by adding multiplets $R_u$ and $R_d$ with the appropriate charges to allow *R*-symmetric $\mu$-terms with $H_u$ and $H_d$ respectively. The scalar components of the Higgs (and not the *R*-fields) acquire VEVs that break electroweak symmetry, thereby preserving the *R*-symmetry. This general class of models goes under the name of the Minimal *R*-symmetric Supersymmetric standard model (MRSSM) [1177, 1178].

The phenomenology of MRSSM is quite different from that of the MSSM. Since the mixing with additional scalars reduces the tree-level Higgs mass, loop corrections must play even more significant role than in the MSSM. Recently it has been shown [1298, 1299] that additional contributions from TeV-scale chiral adjoint superfields and *R*-Higgses allow one to accommodate a light Higgs boson of mass $\sim$125 GeV more comfortably than in models such as the cMSSM even for stop masses of order 1 TeV and absence of stop mixing. Moreover important constraints from EWPO are imposed on parameters entering the Higgs mass calculation, in particular the *W* boson mass, because *R*-symmetry necessarily introduces an *SU*(2) scalar triplet that develops a VEV. A full one-loop calculation [1299, 1300] shows that regions of parameters can be found consistent with the measured Higgs and *W* boson masses.

**Fig. 150:**
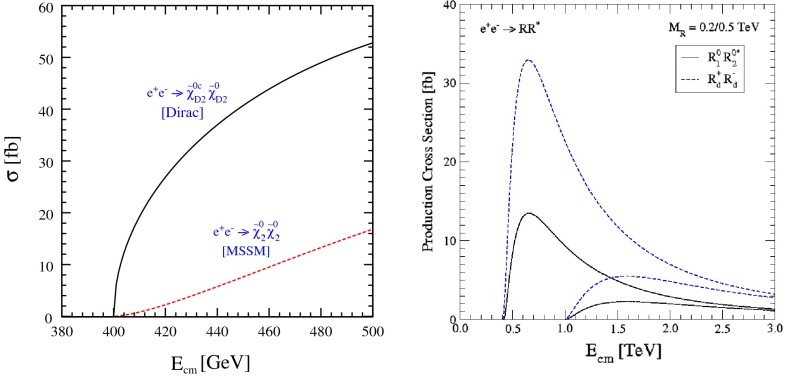
*Left panel* pair production of wino-like neutralinos near threshold in the MSSM and the Dirac theory (from [1192]. *Right panel* production of the neutral and charged *R*-Higgs boson pairs at TeV $e^+e^-$ colliders (from [1178])

Because gauginos are Dirac, scalars can naturally be lighter than gauginos. The scalar component of the adjoint *SU*(3) super-field, a sgluon, can be relatively light and accessible at the LHC [1182, 1301–1303]. The Dirac neutralinos can easily be tested at a LC by investigating the threshold production behaviour of the diagonal-pair production (Fig. 150) or by angular distributions. In contrast to standard Higgs, the *R*-Higgs bosons do not couple singly to SM fields, and all standard-type channels are shut for the single production. Nevertheless, if they are not too heavy, the *R*-Higgs bosons can be produced in pairs at the LHC, via the Drell–Yan mechanism, and at prospective $e^+e^-$ colliders (see Fig. 150).

*R*-symmetry allows *either* Yukawa or *A*-terms, but not both. With the neutrino Yukawas zero, large *A*-terms for sneutrinos are thus natural in the MRSSM. With three singlet superfields $N_i$, a $6\times 6$ sneutrino mass matrix can feature large off-diagonal *A*-terms mixing the left- and right-handed sneutrinos. In such a framework, a mixed sneutrino can serve as a successful candidate for DM, an appropriate Majorana neutrino masses can be generated and striking lepton-flavour violation signals can be expected at both LHC and linear colliders [1304].

### Relevance of $e^-e^-$, $e\gamma$ and $\gamma \gamma$ options for SUSY searches

Linear colliders offer an impressive capacity to discover and untangle new physics such as supersymmetry in $e^+e^-$ collisions. Their ability to adapt to the specific needs of various scenarios of new physics is augmented by the possibility to run such machines in $e^-e^-$, $e\gamma$ or $\gamma \gamma$ modes. In the latter two cases, the $\gamma$s are generated via laser back-scattering off of the incoming electron beams. Each of these options offers new avenues for understanding supersymmetry.

By operating in the $e^-e^-$ mode, a vast array of SM background processes that could be problematic at $e^+e^-$ colliders are automatically turned off. One might counter that most SUSY production reactions are also turned off in the $e^-e^-$ mode as well. Reactions like $e^-e^-\rightarrow \tilde{e}_R^-\tilde{e}_R^-$, $\tilde{e}_L^-\tilde{e}_L^-$, $\tilde{e}_L^-\tilde{e}_R^-$ and $\tilde{e}_R^-\tilde{e}_L^-$ provide distinctive SUSY signals [1305–1310]. These take place via *t*-channel neutralino exchange. An advantage of $e^-e^-$ collisions is obtained in threshold scans: whereas $e^+e^-\rightarrow \tilde{e}_R^+\tilde{e}_R^-,\ \tilde{e}_L^+\tilde{e}_L^-$ suffer the usual $\beta ^3$ suppression factor typical of scalar pair production, the $e^-e^-\rightarrow \tilde{e}_R^-\tilde{e}_R^-$ and $\tilde{e}_L^-\tilde{e}_L^-$ reactions are only suppressed by $\beta ^1$. This offers better accuracy in the selectron mass measurement via the unsuppressed threshold production of selectron pairs. This is especially important in that threshold scans for $\beta ^3$ suppressed processes will require very high integrated luminosity while similar or better measurements can be made on $\beta ^1$ suppressed processes at much lower integrated luminosity.

Since $\tilde{e}_R^-\tilde{e}_R^-$ takes place via pure bino exchange, the total rate for this reaction will be highly sensitive to the bino mass (assuming a nearly pure binno-like gaugino) all by itself even if $m_{\tilde{B}}$ is far beyond direct production (in such a case, perhaps the LSP would be the lightest Higgsino with $m_{\tilde{B}}$ much heavier). Furthermore, using beam polarisation, one might dial up individual reactions $\tilde{e}_L^-\tilde{e}_L^-$, $\tilde{e}_R^-\tilde{e}_R^-$ or $\tilde{e}_L^-\tilde{e}_R^-$. It has also been emphasised that $\tilde{e}_{L,R}^-\tilde{e}_{L,R}^-$ production would be an excellent environment for testing possible rare, perhaps flavour-violating, slepton decay modes to the low background environment [1305].

The possibility of $e\gamma$ colisions is important in several cases relevant for SUSY searches [1310–1312]. The first scenario is offered by the reaction $e\gamma \rightarrow \tilde{e}_{L,R}\tilde{\chi }^{0}_i$, or single production of selectrons. In this case, even if $\tilde{e}_{L,R}^+\tilde{e}_{L,R}^-$ is beyond the maximal $\sqrt{s}$ of an $e^+e^-$ collider, then if $m_{\tilde{\chi }^{0}_1}$ is light, single production of sleptons may take place for $\sqrt{S}>m_{\tilde{e}}+m_{\tilde{\chi }^{0}_1}$. The utility of an $e\gamma$ collider has also been considered for GMSB SUSY models where one may produce $\tilde{e}_{L,R}\tilde{G}$ where $m_{\tilde{G}}$ may be very light [1313], and in models with *R*-parity violation [1314].

A linear collider running in $\gamma \gamma$ mode (two back-scattered laser beams) has been considered in [1315, 1316] for chargino pair production and in [1317] for sfermion production. For $\gamma \gamma$ collisions, the couplings are pure QED so that the production cross sections depend only on the mass of the charged sparticles which are being produced. For both these cases, an advantage can be gained by scattering polarised laser light on polarised beam to gain polarised photon collisions. A variety of helicity studies can then be made on the various sparticle pair production processes.

### Summary and conclusions

It is timely to re-assess the physics opportunities related to SUSY models for an $e^+e^-$ linear collider before the start of LHC operation at 13–14 TeV. The run at 7 and 8 TeV has been marked by the discovery of a Higgs-like boson with a mass $M_h\sim 125$ GeV and has provided us with important bounds on the mass of new particles from dedicated SUSY searches. These LHC results are complemented by important data on DM, from the precision determinations of its relic density from the CMB spectrum to much improved bounds on its scattering cross section from underground search experiments.

The combination of the relatively light mass of the newly discovered Higgs-like particle, easily interpretable within SUSY, and the compelling evidence for DM, which can be explained as due to relic neutralinos or gravitinos, have reinforced the interest for supersymmetric models. The combined 7 + 8 TeV LHC data have already set significant bounds on the masses of strongly interacting SUSY particles in the jets + MET channel and have started addressing the detection of weakly interacting particles in $\ell$s + MET and *h* + MET channels and more model-independent searches for neutralino LSPs and nearly degenerate squark–neutralino scenarios with monojets.

All these searches will have a powerful impact on supersymmetric models with the Run-2 data taking at 13–14 TeV and higher luminosity. However, despite the broad range and the ingenuity of the LHC searches, scenarios with nearly degenerate sparticle–neutralino LSP masses, compressed spectra, multiple decay modes with comparable rates and some of the ’natural’ SUSY spectra may prove difficult for the LHC to probe in full. In fact, if we take guidance from the concept of naturalness and the fine tuning of supersymmetric models, we are brought to consider natural SUSY models which contain a spectrum of light higgsino particles. In these models, gluinos and scalar quarks may be as heavy as several-TeV, with TeV-values stops required to be highly mixed in order to lift $M_h$ up into the 125 GeV range. Such ‘natural’ SUSY spectra would be characterised by electroweak fine tuning at the level of $\sim$10 % and their concomitant light higgsinos could be readily detected and studied at an $e^+e^-$ linear collider of sufficient energy. When the higgsino mass $\mu$ sets the scale for fine tuning, then we expect a centre-of-mass energy $E_{CM}$ to probe electroweak fine tuning of $E_{CM}>2\mu \sim \sqrt{2{\varDelta }_{\mathrm{EW}}}M_Z$.

In these scenarios, the combination of clean environment, the well-known beam energy, the adjustable centre-of-mass energy and the availability of polarised beams at the $e^+e^-$ linear collider will provide us with the tools required for precision measurements of masses, spins and other quantum numbers of these new states. Precision mass and spin measurements can be performed either by kinematic measurements in the continuum or via threshold scans. An $e^+e^-$ collider should be able to extract precision values of scattering cross sections, branching fractions, angular distributions of final-state particles and decay widths.

These precision measurements will lead to the extraction of the fundamental SUSY Lagrangian parameters and test the unification at very high-energy scales. All together these measurements will provide us with a unique window onto the energy scales associated with grand unification.

Production of SUSY particles at an $e^+e^-$ linear collider may allow for tests of the Majorana nature of gauginos, flavour-violating decays, ${\textit{CP}}$-violating processes, *R*-parity-violating reactions (which can also elude LHC searches), *R*-symmetry effects and the presence of additional matter states such as the added singlets in extended models. In the event that just a few SUSY particles are produced at some energy scale, then the linear collider can still determine the fundamental SUSY parameters in a model-independent way and can still test higher mass scales through virtual particle exchange, such as sneutrino exchange effects in chargino pair production, and additional SUSY parameters via loop effects, for instance, to Higgs branching fractions.

The knowledge obtained from combining the data of the LHC, an $e^+e^-$ linear collider and DM experiments will be crucial for understanding the nature of DM and, possibly, test models of baryogenesis.

From all these facets, it is clear that a linear $e^+e^-$ collider operating in the $\sim$0.25–1 TeV range can play a major role in the study of supersymmetry – ranging from discovery to precision measurements – and will provide a new and more refined view as to the next level in the laws of physics as we know them.

## Connection to astroparticle physics and cosmology^63^

### Introduction

While an enormous amount of energy is spent on the search for physics beyond the standard model, perhaps the most compelling reason for expecting new physics is DM. The evidence for DM is overwhelming. On galactic scales, one observes relatively flat rotation curves [1318–1325] which cannot be accounted for by the observed luminous component of the galaxy. The simplest interpretation of these observations is that nearly all spiral galaxies are embedded in a large galactic halo of DM which lead to rather constant rotational velocities at large distances from the centre of the galaxy. X-ray emission from a hot gas surrounding large elliptical galaxies and clusters of galaxies also require a large potential well (to gravitationally bind the hot gas) which cannot be accounted for by the galaxy or gas itself [1326–1334]. Gravitational lensing also implies large gravitational potentials from unseen matter on the scale of clusters of galaxies [1335–1338]. In addition, there are observations of both X-ray emitting hot gas and gravitational lensing in the same systems [1339, 1340] which all point to the presence of dark matter.

On larger scales, baryon acoustic oscillations [1341] indicate a matter component ${\varOmega }_m = \rho _m/\rho _c \simeq 0.25$, where $\rho _c = 1.88 \times 10^{-29} h^2$ g cm$^{-3}$ is the critical energy density for spatial flatness. However, the baryon density of the universe from big bang nucleosynthesis (BBN) [1342] is restricted to ${\varOmega }_B h^2 \lesssim 0.03$ where $h = 0.71$ is the Hubble parameter in units of 100 km/s/Mpc. Furthermore, both the estimates from baryon acoustic oscillations and nucleosynthesis are in complete agreement with the determination of both the total matter density and the baryon density from the cosmic microwave background anisotropy spectrum [46, 47] which yields a DM density of

160 $$\begin{aligned} {\varOmega } h^2 = 0.1196 \pm 0.0031 . \end{aligned}$$

As we will see, there are no candidates for the DM of the universe found in the SM. Thus the body of evidence for DM clearly points to physics beyond the SM. Below, we will briefly describe some of the well-studied candidates for DM with an emphasis on their relevance for a future LC.

### Candidates

With the discovery of the Higgs boson [1343, 1344], the SM field content is complete. As DM must be stable or long lived, a priori there are only two possible candidates for DM in the SM. While baryonic DM may account for some of the DM in galactic halos, it cannot make up the bulk of the DM in the universe. As noted above, BBN limits the baryon density to less than 25 % of the total amount of non-relativistic matter in the universe, which is consistent with the determination of the baryon density from microwave background anisotropies. However, a dominant component of baryonic DM even on the galactic scale is problematic [1345]. Put simply, baryons tend to clump and form stellar-like objects. While massive objects such as white dwarfs or neutron stars or black holes may be dark, they are typically associated with heavy element production and a significant number of these objects would produce excessive metallicity. Smaller Jupiter-like objects would require a very special mass distribution to avoid constraints from luminosity density in the red and infrared. More concrete constraints are obtained from microlensing observations [1346–1351] where the contribution of such objects (collectively known as MACHOs) is limited to less than 25 % of the halo for masses $2 \times 10^{-7} M_\odot < M < 1 M_\odot$.

Another potential possibility for a DM candidate in the SM is a neutrino. Indeed, neutrino oscillation experiments indicate that at least one neutrino has a mass in excess of 0.05 eV. This would correspond to a cosmological contribution, ${\varOmega }_\nu h^2 > 5\times 10^{-4}$. However, there are upper limits to the sum of neutrino masses from large scale structure considerations. In particular, using CMB data (notably PLANCK, WMAP 9-years, ACT and SPT) and including observations from BAO and HST, one finds that the sum of neutrino masses is constrained to be $\sum m_{\nu } < 0.22$ eV corresponding to ${\varOmega }_{\nu } h^2 < 2.4 \times 10^{-3}$ [1352].

By the 7-year WMAP data and including observations from SDSS and HST, one finds that the sum of neutrino masses is constrained to be $\sum m_\nu < 0.39$ eV corresponding to ${\varOmega }_\nu h^2 < 4 \times 10^{-3}$ [1353].

At this time, if there is any firm indication of physics beyond the SM, it comes from our understanding of DM in the universe. While not all DM candidates can be probed by a future linear collider, we will restrict our attention to those that can. Thus we will not discuss possibilities such as sterile neutrinos or axions below and we concentrate on those candidates with potential signatures at the LC.

#### Supersymmetric candidates

The supersymmetric extension of the SM is one of the most studied example of physics beyond the SM and is currently being tested at the LHC. Its motivations (which we will not review here) include the stabilisation of the weak-scale hierarchy, gauge coupling unification, radiative EWSB, and the prediction of a light Higgs boson ($m_h \lesssim 130$ GeV) which has been borne out by experiment [1343, 1344]. In models with *R*-parity conservation, another prediction of supersymmetric models, is the existence of one stable particle, which if neutral, may be candidate for the DM. This is the lightest supersymmetric particle of LSP. Below, we review some of the most studied realisations of the low-energy supersymmetry.

For the most part, we will restrict our attention here to the MSSM (though see below for a discussion of the next to minimal model or NMSSM). The minimal model is defined by the superpotential

161 $$\begin{aligned} W = \bigl ( y_e H_1 L e^c + y_d H_1 Q d^c + y_u H_2 Q u^c \bigr ) + \mu H_1 H_2 , \end{aligned}$$

Beyond the parameters associated with the SM, the superpotential introduces a mixing term between the two Higgs doublets in the MSSM. The bulk of the new parameters are associated with supersymmetry breaking and are associated with soft scalar masses, gaugino masses, and so-called bi- and tri-linear terms, *B* and *A*. There are well over 100 new parameters in the minimal theory and we are thus forced to make some (well-motivated) simplifications as we discuss below.

*The CMSSM* As is clear, supersymmetry must be broken, and one way of transmitting the breaking of supersymmetry to the low energy sector of the theory is through gravity. Indeed the extension of global supersymmetry to supergravity is in some sense necessary to ensure the (near) vanishing of the cosmological constant in models with weak-scale supersymmetry breaking. Gravity-mediated supersymmetry breaking imposes a number of boundary conditions on the supersymmetry breaking masses at some high-energy renormalisation scale, which is usually taken to be the same scale at which gauge coupling unification occurs, $M_{\mathrm{GUT}}$. In gravity mediated models, one often finds that all scalar masses are equal at $M_{\mathrm{GUT}}$ defining a universal scalar mass $m_0$. Similarly, all gaugino mass and tri-linear terms are also universal at $M_{\mathrm{GUT}}$, with values $m_{1/2}$ and $A_0$, respectively.

**Fig. 151:**
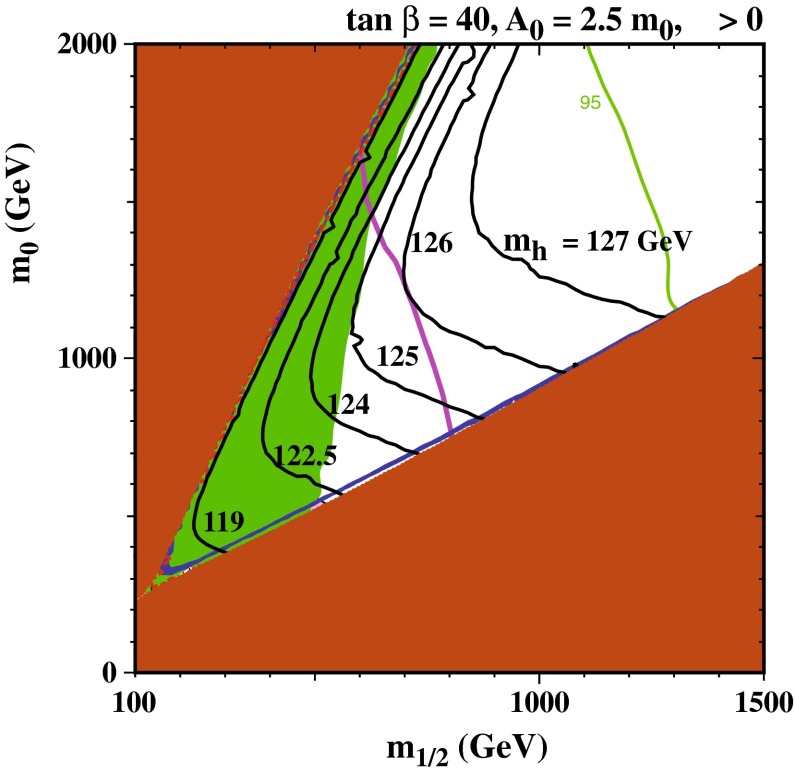
The $(m_{1/2}, m_0)$ plane for $\tan \beta = 40$ and $\mu > 0$, assuming $A_0 = 2.5 m_0, m_t = 173.2$ GeV and $m_b(m_b)^{\overline{\mathrm{MS}}}_{\mathrm{SM}} = 4.25$ GeV. Contours and shaded regions are described in the text

In these gravity-mediated supersymmetry breaking models, supersymmetry breaking masses and gauge and Yukawa couplings are run down from the universality scale and often trigger electraoweak symmetry breaking as one or both of the soft Higgs masses, $m_{1,2}^2$ run negative. In true minimal supergravity models or mSUGRA, the scalar mass is equal to the gravitino mass, $m_0 = m_{3/2}$, and the *B*-term is given by $B_0 = A_0 - m_0$. One consequence of the latter relation is the determination of the two Higgs vacuum expectation values as the soft masses are run down to the weak scale. Since one combination of the two VEVs determines the *Z* gauge boson mass, it is common to choose the two VEVs as input parameters (the other combination is the ratio of VEVs and defined as $\tan \beta = v_2/v_1$) and discard the relation between $B_0$ and $A_0$. Instead both *B* and $\mu$ can be calculated at the weak scale from $M_Z$ and $\tan \beta$. If the relation between the gravitino mass and $m_0$ is also dropped, we have the constrained version of the MSSM known as the CMSSM.

The CMSSM is therefore a four parameter theory (the sign of $\mu$ must also be specified). For given values of $\tan \beta$, $A_0$, and $sgn(\mu )$, the regions of the CMSSM parameter space that yield an acceptable relic density and satisfy other phenomenological constraints may be displayed in the $(m_{1/2}, m_0)$ plane. In Fig. 151 [1354], the dark (blue) shaded region corresponds to that portion of the CMSSM plane with $\tan \beta = 40$, $A_0 = 2.5 m_0$, and $\mu > 0$ such that the computed relic density yields the PLANCK value given in Eq. (160). For this choice of $\tan \beta$ and $A_0$, the relic density strip is *v*-shaped. Inside the ‘*v*’, the annihilation cross sections are too small to maintain an acceptable relic density and ${\varOmega }_\chi h^2$ is too large. The upper side of the ‘*v*’, at large $m_0$, is produced by coannihilation processes between the LSP and the next lightest sparticle, in this case the $\tilde{t}$ [1355–1360]. These enhance the annihilation cross section and reduce the relic density. This occurs when the LSP and NLSP are nearly degenerate in mass. The lower side of the ‘*v*’, at lower $m_0$, is produced by coannihilations between the LSP and the $\tilde{\tau }$ [1361–1367]. The dark (brown) shaded regions outside of the ‘*v*’ have either $m_{\tilde{t}}< m_\chi$ or $m_{\tilde{\tau }}< m_\chi$ and are excluded. Also shown in the figure is the constraint from $b \rightarrow s \gamma$ [1368–1371] (shaded green) which excludes the stop-coannihilation strip in the portion of the plane shown. Contours of constant Higgs mass are shown by the black curves. Higgs masses are computed using FeynHiggs [226, 261, 837, 838, 1372, 1373] and carry a roughly 1.5 GeV uncertainty. The thick purple line corresponds to the ATLAS limit on supersymmetry searches [1374]. The area to left of the line is excluded. Finally, the solid green contour corresponds to the 95 % CL upper limit to ratio of the branching fraction of $B_s \rightarrow \mu ^+ \mu ^-$ relative to the SM [1375–1377].

**Fig. 152:**
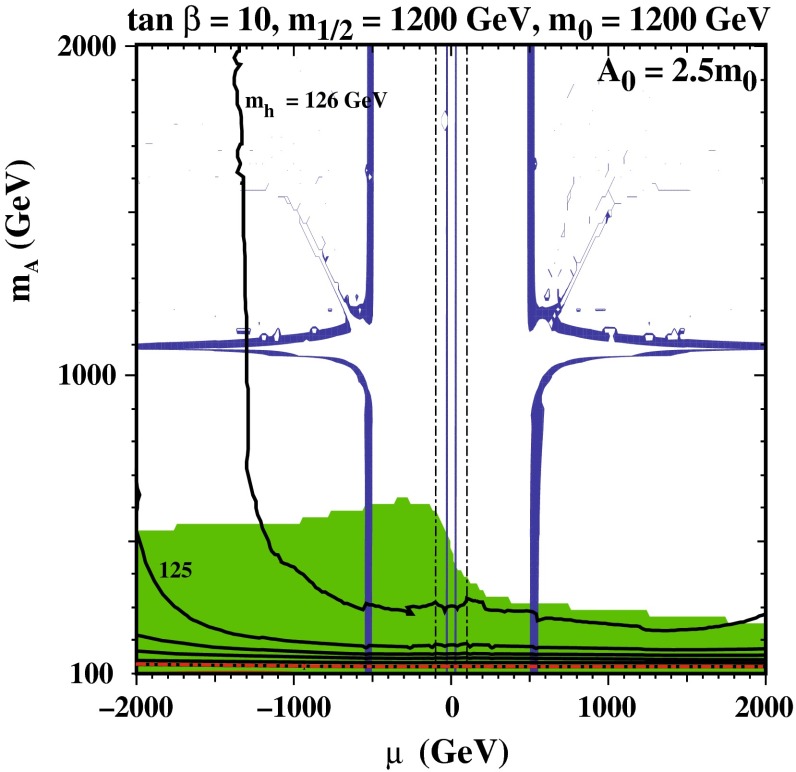
The $(\mu , m_A)$ plane for $\tan \beta = 30$, $m_{1/2} = m_0 = 1000$ GeV, assuming $A_0 = 2.5 m_0, m_t = 173.2$ GeV and $m_b(m_b)^{\overline{\mathrm{MS}}}_{\mathrm{SM}} = 4.25$ GeV. Contours and shaded regions are described in the text

Note that the choice $A \ne 0$ is made to ensure a sufficiently large Higgs mass. For $A_0 = 0$, the maximum Higgs mass along the stau-coannihilation strip is only slight greater than 120 GeV, far short of the value reported in the recent LHC results [1343, 1344]. Therefore, only the upper end of the strip is compatible with a Higgs mass around 125–126 GeV and a branching fraction for $B_s \rightarrow \mu ^+ \mu ^-$ sufficiently close to the SM value.

*NUHM* One possible generalisation of the CMSSM is the so-called NUHM in which the Higgs soft masses are not constrained to be equal to $m_0$. Indeed, as the Higgses are typically found in separate multiplets in a grand unified theory, one or both of the Higgs soft masses may be independent. In the NUHM1, we may set $m_1 = m_2 \ne m_0$, where $m_{1,2}$ are the soft masses associated with $H_{1,2}$. Instead of $m_{1,2}$, one may choose *either*$\mu$*or* the Higgs pseudoscalar mass, $m_A$ (which is a surrogate for *B*) as a free parameter in addition to $m_0$. In the NUHM2, both $m_1$ and $m_2$ are free and one can equivalently choose *both*$\mu$*and*$m_A$ as free parameters.

In Fig. 152 [1354], we show one example of a $\mu , m_A$ plane with $\tan \beta = 10$, $m_{1/2} = m_0 = 1200$ GeV, and $A_0 = 2.5 m_0$. The strips of acceptable relic density now form a cross-like shape. Outside the cross, the relic density is too large. The horizontal part of the crosses are due to an enhanced cross section through rapid *s*-channel annihilation through the heavy Higgses. For $m_{1/2} = 1200$ GeV, the neutralino mass, is roughly 520 GeV and the funnel-like region occurs when $m_A \approx 2 m_\chi$. In contrast, the vertical part of the cross occurs when $\mu$ becomes sufficiently small that the LSP picks up a significant Higgsino component (at large $|\mu |$, it is almost pure bino) which enhances certain final-state annihilation channels such as $W^+ W^-$.

The region in Fig. 152 with low $m_A$ is excluded by $b \rightarrow s \gamma$ and is slightly more pronounced when $\mu < 0$. At $\tan \beta = 10$, the branching fraction for $B_s \rightarrow \mu ^+ \mu ^-$ is sufficiently small. On the other hand, the Higgs mass is $\approx 126$ GeV across much of the plane. The vertical dashed black lines at small $|\mu |$ correspond to a chargino mass at the lower limit of 104 GeV.

*The pMSSM* As noted earlier, the most general MSSM contains more than 100 free parameters and is therefore not a convenient framework for phenomenological studies. However, with a few well-motivated assumptions (*R*-parity conservation, no new $${\textit{CP}}$$ phases, the sfermion mass matrices and tri-linear couplings are flavour diagonal, the first two generations are degenerate and their tri-linear coupling is negligible) the number of free parameters can be reduced to a more manageable number. This is the so-called phenomenological MSSM (pMSSM) with 19 free parameters in addition to the SM parameters: the gaugino mass parameters, $$M_1,M_2,M_3$$, the ratio of the Higgs VeVs, $$\tan \beta =v_1/v_2$$, the higgsino mass parameter, $$\mu $$, and the pseudoscalar mass, $$m_A$$, ten sfermion mass parameters, $$m_{\tilde{Q}_i},m_{\tilde{U}_i},m_{\tilde{D}_i},m_{\tilde{L}_i},m_{\tilde{E}_i}$$$$i=2,3$$ and three tri-linear couplings $$A_t,A_b,A_\tau $$. This model, which is not tied to a specific symmetry breaking mechanism, leads to a much broader set of predictions for experimental observables at the LHC or in the DM sector.

Relaxing the relation between the parameters of the electroweak-ino sector, which are most relevant for DM observables and those of the coloured sector, most relevant for LHC, not only relaxes some of the limits from SUSY searches at LHC but also influences the expectations for DM observables [1120, 1121, 1378, 1379]. In the pMSSM, the neutralino LSP can have any composition, making it much more likely than in the CMSSM to have a very small value for the relic density. Indeed, a significant higgsino (or wino) component both lead to an enhancement of annihilation in W pair final states as well as to enhance gaugino/higgsino coannihilations. On the other hand a higgsino LSP faces severe constraints from direct detection; see the next section. Enhanced annihilation through a Higgs funnel can occur for any value of $\tan \beta$ and for any DM mass provided $m_\mathrm{LSP}\approx m_H/2$. Finally, coannihilations can occur with any supersymmetric partners that are sufficiently degenerate in mass with the LSP.

*NMSSM* The next-to-minimal supersymmetric standard model (NMSSM) is a simple extension of the MSSM that contains an additional gauge singlet superfield. The VEV of this singlet induces an effective $\mu$ term that is naturally of the order of the electroweak scale, thus providing a solution to the naturalness problem [89]. The model contains one additional neutralino state, the singlino, as well as three scalar ($h_1,h_2,h_3$) and two pseudoscalar ($a_1,a_2$) Higgs bosons. An important feature of the model is that the singlet fields can be very light and escape the LEP bounds. This is because these fields mostly decouple from the SM fields. Furthermore large mixing with the singlet can modify the properties of the SM-like Higgs, allowing quite naturally for $m_h=126$ GeV as well as possibly an enhanced rate for its decay into two photons. With regard to DM, the NMSSM shares many of the characteristics of the MSSM. The main differences occur when the LSP has some singlino component and/or when the Higgs sector contains new light states that play a role in DM interactions. For example new Higgs states can greatly enhance DM annihilation when their mass is twice that of the LSP or can provide new annihilation channels when they can be produced in the final state. As a consequence, the NMSSM allows for the possibility of light neutralinos (much below $M_Z/2$), which annihilate efficiently through the exchange of light Higgs singlets or into light Higgs singlets [1380]. The model also accommodates the possibility of a gamma-ray line at 130 GeV, without violating any other constraints from cosmic rays. This requires fine tuning of the parameters such that (1) the mass of a pseudoscalar is precisely twice the neutralino mass and (2) the annihilation of the pseudoscalar is dominantly into two photons rather than into quarks [496].

#### Universal extra dimensions

**Fig. 153:**
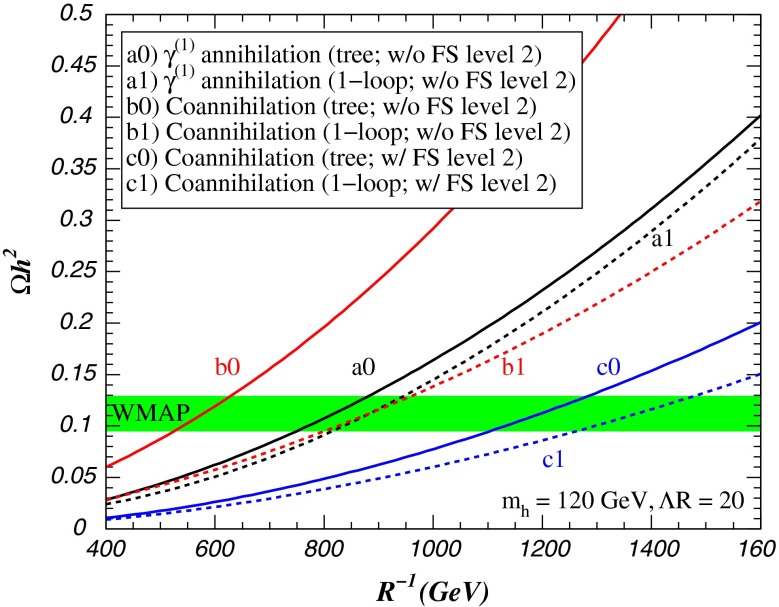
${\varOmega } h^2$ as a function of $R^{-1}$ for $m_h=120$ GeV and ${\Lambda } R=20$ including different processes as specified on the figure. Here ‘1-loop’ stands for one-loop couplings between level 2 and SM particles [1382]

Extra dimension models also propose a WIMP DM candidate. The UED scenario [1381] where all SM particles are allowed to propagate freely in the bulk is of particular interest. In this model momentum conservation in the extra dimensions entails conservation of a KK number. Orbifolding is required to obtain chiral zero modes from bulk fermions, and this breaks extra dimensional momentum conservation. However, there remains a discrete subgroup, KK parity, thus the lightest KK-odd particle is stable. In the minimal universal extra dimension model (MUED) the DM candidate is in general a vector particle, $B_1$, the Kaluza–Klein (KK) level 1 partner of the *U*(1) gauge boson. In the MUED model all KK states of a given level have nearly the same mass at tree level, *n* / *R*, where *R* is the size of the compact dimension. The mass degeneracy is lifted only by SM masses and by radiative corrections. These mass splittings are, however, small for all weakly interacting particles. This means that coannihilation channels naturally play an important role in the computation of the relic abundance of DM. Furthermore since the level 2 particles are close to twice the mass of those of level 1, annihilation or coannihilation processes can easily be enhanced by resonance effects. When including level 2 particles in the computation, the preferred scale for DM was found to be around 1.35 TeV, see line c1 in Fig. 153 [1382]. Going beyond the MUED framework one can treat mass splittings as free parameters, shifting significantly the preferred DM mass, for example in the limit where the coannihilation processes are negligible the DM mass is around 800 GeV, see line a1 in Fig. 153. The measurement of the Higgs mass and of its couplings at the LHC can be used to put a lower limit on the scale *R*. Indeed light KK particles, in particular the KK top, lead to an increase in the *hgg* coupling and to a decrease in the $h\gamma \gamma$ coupling, and to a lower bound on $R>500$ GeV [1383]. One characteristic of MUED DM is that annihilation in the galaxy has a large fraction into fermions leading to strong signal into positrons, however, the large mass scale makes the signature unlikely to be observable [1384].

#### Higgs-portal models

The Higgs portal refers to a class of models where the Higgs connects the DM (hidden) sector to the SM. Several possibilities have been considered with either a scalar, a vector or a fermion as DM. The simplest extension of the SM is the addition of a real singlet scalar field, *S*, which can be made stable by imposing a $Z_2$ symmetry. If the true vacuum of the theory satisfies $\langle S \rangle =0$, thereby precluding mixing of *S* and the SM Higgs boson and the existence of cosmologically problematic domain walls. The terms to be added to the SM Lagrangian are

162 $$\begin{aligned} {\varDelta } {\mathscr {L}}_S = -{1\over 2} m_S^2 S^2 - {1\over 4} \lambda _S S^4 - {1\over 4} \lambda _{hSS} H^\dagger H S^2 . \end{aligned}$$

**Fig. 154:**
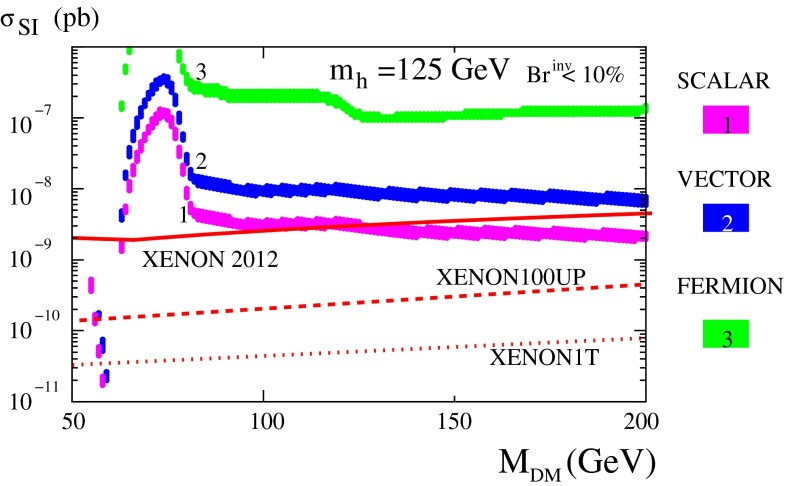
Spin-independent DM–nucleon cross section versus DM mass. The *upper band (3)* corresponds to fermion DM, the *middle one (2)* to vector DM and the *lower one (1)* to scalar DM. The *solid*, *dashed* and *dotted lines* represent XENON100 (2012 data [1105]), XENON100 upgrade and XENON1T sensitivities, respectively

A second possibility is to couple the Higgs doublet to a massive vector field $X_\mu$ from the hidden sector. $X_\mu$ can be associated with a hidden *U*(1) and becomes massive due to the Higgs or Stückelberg mechanism in the hidden sector. A third possibility is the one where DM can consist of Majorana fermions $\chi$ which interact with the SM fields only through the Higgs portal. In both cases the stability of the DM particle is ensured by a $Z_2$ parity, whose origin is model-dependent. For example, in the vector case it stems from a natural parity symmetry of abelian gauge sectors with minimal field content [1385]. The relevant terms in the Lagrangians are

163 $$\begin{aligned}&{\varDelta } {\mathscr {L}}_V = {1\over 2} m_V^2 V_\mu V^\mu + {1\over 4} \lambda _{V} (V_\mu V^\mu )^2 + {1\over 4} \lambda _{hVV} H^\dagger H V_\mu V^\mu , \nonumber \\&{\varDelta } {\mathscr {L}}_f = - {1\over 2} m_f \bar{\chi }\chi - {1\over 4} {\lambda _{hff}\over {\Lambda }} H^\dagger H \bar{\chi }\chi . \end{aligned}$$

Related ideas and analyses can be found in [454, 1385–1409] and more recent studies of Higgs-portal scenarios have appeared in [1410–1420].

In these models, the Higgs is responsible for both DM annihilation and elastic scattering of DM with nuclei. Thus, cosmological measurements made by the WMAP and PLANCK satellites [46, 47] basically determine the couplings of the Higgs to DM and thus the spin-independent DM–nucleon cross section for a given DM mass. The same coupling will also determine the Higgs partial decay widths into invisible DM particles if $m_\mathrm{DM} \le \frac{1}{2} m_h$. The discovery of a Higgs boson with a mass $m_h =125$ GeV with a small invisible decay branching ratio is incompatible with DM with $m_\mathrm{DM} \le 55$ GeV. This applies in particular to the case of scalar DM with a mass of 5–10 GeV considered, for instance, in Ref. [1407]. Figure 154 displays the predictions for the spin-independent DM–nucleon cross section $\sigma _\mathrm{SI}$ after imposing the WMAP and $\mathrm{BR}^\mathrm{inv} <$10 % constraints (allowing the invisible width to be 20% does not change the result significantly). The upper band corresponds to the fermion Higgs-portal DM and is excluded by XENON100, while scalar and vector DM are both allowed for a wide range of masses. The typical value for the scalar $\sigma _\mathrm{SI }$ is a few times $10^{-9}$ pb, whereas $\sigma _\mathrm{SI }$ for vectors is larger by a factor of 3, which accounts for the number of degrees of freedom. We note that a large fraction of the parameter space will be probed by XENON1T except for a small region where $m_\mathrm{DM}\approx m_h/2$ and the Higgs–DM coupling is extremely small.

#### Extended scalar sector

The Higgs discovery has revived the interest in models with an extended scalar sector. In such models an unbroken discrete symmetry which could be leftover from a broken gauge group at a higher scale guarantees the stability of the lightest scalar, the DM candidate. One of the nice feature of these models is that the quartic couplings between the SM-like doublet and other scalars helps stabilise the scalar potential by giving a contribution that counteracts the effect of the top Yukawa that drives the SM potential to the metastability region [75, 1421]. The archetype of scalar DM models is the inert doublet model [1422] where the second doublet has no VEV, and no coupling to quarks and leptons. Models with only additional singlets [1386–1393], with a doublet and singlet [1423, 1424] or with higher multiplets [1425–1429] have also been proposed and different discrete symmetries to stabilise the DM were considered [1423, 1424].

In the inert doublet model, the DM can be either a scalar or pseudoscalar. After imposing constraints on the model from perturbativity, stability, direct searches for charged Higgs and electroweak precision tests, several studies have found that a value of the relic density in accordance with PLANCK can be reproduced in the low mass $m_\mathrm{DM}<60$ GeV, intermediate $60<m_\mathrm{DM}<110$ GeV and high mass range ($m_\mathrm{DM}>500$ GeV) [1422, 1430–1433]. The low and intermediate mass ranges are severely constrained by Higgs measurements and direct detection. In the low-mass region, DM annihilation proceeds through Higgs exchange and as in the portal models is constrained by the upper limit on the Higgs invisible width. In the intermediate region annihilation into *W* pairs (including virtual *W*s) start to dominate. However, the Xenon and LUX upper limits forces the DM mass to be near $m_h/2$ and $m_W$. For DM masses above $m_W$ the annihilation into *W* pairs becomes very efficient thus leading to too low a value for the relic density unless the DM mass is larger than 500 GeV, These allowed mass ranges can be extended in models with more particles in the inert sector and/or in models which also involve semiannihilation [1424]. The collider signatures in the Higgs sector involve invisible decays (already severely constrained) and a modification of the two-photon decay width due to the charged Higgs contribution [1434]. At the LC, the inert Higgses can be directly produced and their decays into real or virtual gauge bosons exploited to determine the masses of all inert scalars [1435].

### Dark matter at the LHC

Direct searches for supersymmetry at the LHC have had a significant impact on the allowable regions of the supersymmetric parameter space particularly in the context of the CMSSM. An example of this is shown by the purple curve in Fig. 151. For relatively low $m_0$, the most recent results from ATLAS place a lower bound on $m_{1/2}$ of roughly 840 GeV. Perhaps of greater significance is the discovery of the Higgs boson at 125–126 GeV. While consistent with general predictions in supersymmetric models that $m_h \lesssim 128$– 130 GeV, a 125-GeV Higgs lies at the edge of what can be obtained and pushes the model to require large contributions from stop mixing (hence a large value of $A_0$ in the CMSSM) and relatively large SUSY masses. Of course, large SUSY masses are consistent with the lack of discovery of supersymmetric particles at the LHC, and they are consistent with little or no departures from the SM in rare B decays. Of course, this cannot be viewed as a ringing endorsement for supersymmetry. Indeed the past prospect of resolving the discrepancy between theory and experiment for the anomalous magnetic moment of the muon, has now essentially evaporated.

To account for the recent LHC results along with other low-energy observables, it is better to perform a global likelihood analysis which can identify regions of the parameter space which best fit the data. It is well established that Markov-Chain Monte-Carlo (MCMC) algorithms offer an efficient technique for sampling a large parameter space such as the CMSSM or its variants. MCMC has been utilised in the Mastercode [1436] framework to perform a frequentist analysis of the CMSSM and other variants of the model. The MCMC technique is used to sample the SUSY parameter space, and thereby construct the $\chi ^2$ probability function, $P(\chi ^2,N_\mathrm{dof})$. This accounts for the number of degrees of freedom, $N_\mathrm{dof}$, and thus provides a quantitative measure for the quality-of-fit such that $P(\chi ^2,N_\mathrm{dof})$ can be used to estimate the absolute probability with which the CMSSM describes the experimental data.

The results of the mastercode analysis include the parameters of the best-fit points as well as the 68 and 95 % CL regions found with default implementations of the phenomenological, experimental and cosmological constraints. These include precision electroweak data, the anomalous magnetic moment of the muon, *B*-physics observables, the Higgs-boson mass, $m_h$, and the cold DM density. In addition it includes the constraint imposed by the experimental upper limit on the spin-independent DM scattering cross section from LUX [1099]. The results described here are taken from [1437–1440].

**Fig. 155:**
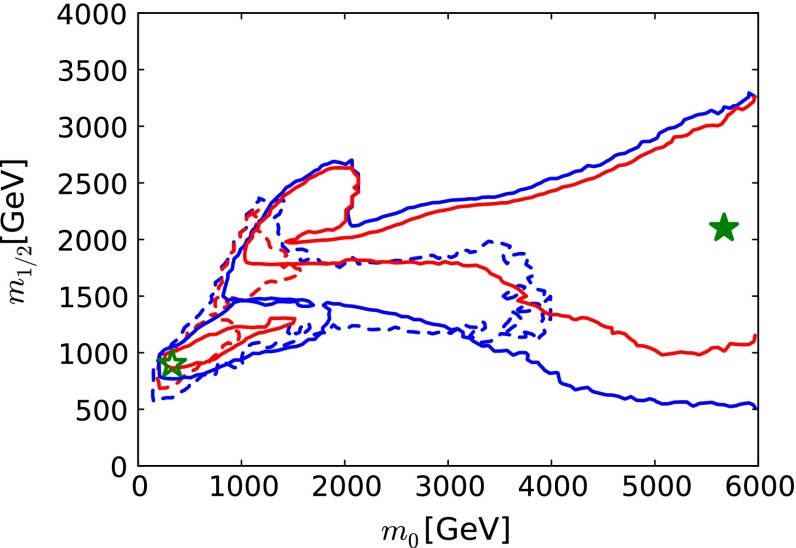
The $(m_0, m_{1/2})$ planes in the CMSSM including the ATLAS 20/fb jets + ${/ E_T}$, BR$(B_{s, d} \rightarrow \mu ^+\mu ^-)$, $m_h$, ${\varOmega }_\chi h^2$, LUX, and other constraints. The most recent results are indicated by *solid lines* and *filled stars*, and previous fit based on $\sim$5/fb of LHC data is indicated by *dashed lines* and *open stars*. The *blue lines* denote 68% CL contours, and the *red lines* denote 95 % CL contours

In Fig. 155, we show the resulting 68 % (shown in red) and 95 % (shown in blue) CL limits from the mastercode analysis [1440] in the $m_0,m_{1/2}$ plane corresponding to ${\varDelta } \chi ^2 = 2.3$ and 5.99 relative to the best-fit point (note the axes are reversed compared to Fig. 151). Results which include the ATLAS constraints at 20/fb are shown by solid curves. The best-fit point is at ($m_0, m_{1/2}$) = (5650, 2100) GeV and is shown by the filled star. At the best-fit point, we also have $A_0 \simeq -780$ GeV, and $\tan \beta = 51$ We see in Fig. 155 that the 95 % CL region in the CMSSM extends to $m_0 \mathop {\sim }\limits ^{>}6000$ GeV and $m_{1/2} \mathop {\sim }\limits ^{>}3000$ GeV. Note that the CMSSM fit features two disconnected 68 % CL ‘islands’, the one at lower $m_0$ and $m_{1/2}$ corresponding to the stau-coannihilation region, and that at larger $m_0$ and $m_{1/2}$ corresponding to the *s*-channel rapid-annihilation funnel region (the best-fit point in the lower island has $\tan \beta = 21$. The low-mass island is only disfavoured at the level of ${\varDelta } \chi ^2 \sim 0.7$, reflecting the relative flatness of the global $\chi ^2$ function.

The impact of the recent LHC results can be seen by comparing the solid curves to the dashed in Fig. 155. The pre-LHC expectations [1437, 1438] were driven to a large extent by $g_\mu -2$. The initial best-fit result was found at quite low susy masses with $(m_0, m_{1/2}) \sim (90, 360)$ GeV and had a p value of 37 %. The entire pre-LHC 68 % CL region is now excluded at 95 % CL, though much of the initial 95% CL region is still valid. The dashed curves in Fig. 155 represent the status of the CMSSM after 5/fb data were collected though assuming a 125-GeV Higgs-boson mass. The best-fit point in this case is at low $m_0, m_{1/2}$ shown by an open star. The p-value in this case is only 8.8 %. Thus already at 5/fb, the LHC results had greatly diminished the probability that the CMSSM improves the fit relative to the SM. The current results have a p value of 5.1 %, which is close to the SM value. Of course, the SM p value does not include the DM constraint as there is no candidate for DM within the SM.

### Other searches

#### Direct detection

Direct searches of DM particles through their scattering off nuclei in a large detector can establish that the DM matter is indeed made of a new stable particle. The elastic scattering of WIMPs off nuclei taking place at low momentum transfer can be conveniently described in terms of an effective Lagrangian interaction of DM with quarks and gluons giving rise to either spin-independent or spin-dependent interactions.

The spin-independent (SI) cross section for WIMPs on nuclei adds coherently and is proportional to the square of the number of nucleons, it therefore usually dominates for heavy nuclei. The spin independent cross section receives a contribution from Higgs exchange, *Z* exchange (except for Majorana fermions) and from interactions with new coloured fermions/scalars (for example new quarks in extra dimension models or squarks in supersymmetry). The latter contribution is, however, constrained by the non-observation of new coloured particles at the LHC.

The spin-dependent (SD) cross section depends solely on the nucleon that contributes to the nucleus spin, and is dominant only for light nuclei. The SD cross section receives contributions from Z exchange and/or from interactions with new coloured fermions/scalars. In order to easily compare results obtained using different nuclei, limits are normally expressed in terms of the SI or SD interaction with protons and neutrons.

At the microscopic level a positive signal in several DM direct searches could altogether lead to information on up to four independent quantities that depend on the details of the DM model, the SD/SI interactions on protons and neutrons. Note, however, that when scalar interactions are dominated by Higgs exchange the cross section on protons and neutrons are almost equal. Furthermore, if the DM has a mass comparable or below that of the nucleus, the shape of the nucleus recoil energy distribution can also be used to extract some rough information of the DM mass.

**Fig. 156:**
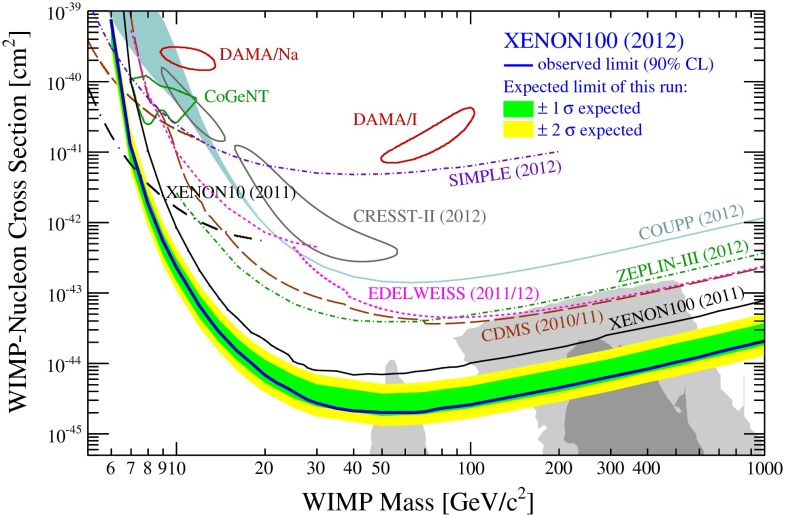
Limits on spin-independent direct detection cross section $\sigma _\mathrm{{SI}}$ on protons *vs.* dark matter mass $m_{DM}$. In grey the preferred region in the CMSSM, from a combination of [1441–1443]

**Fig. 157:**
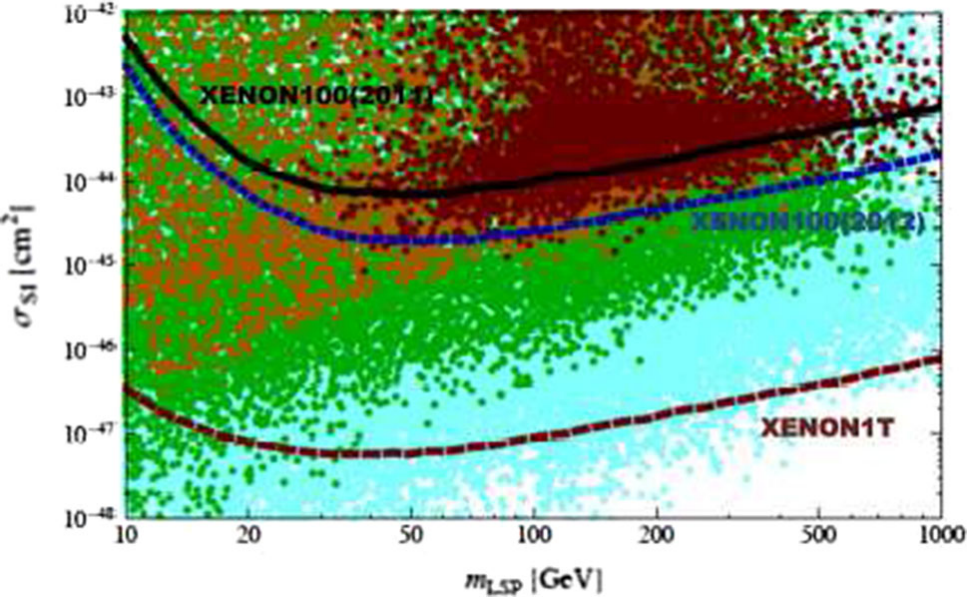
Spin-independent direct detection cross section $\sigma _\mathrm{{SI}}$ on protons *vs.* dark matter mass $m_{LSP}$, from [1450]. The *black* (*blue*) *line* are the 90 % CL limits from the XENON100(2011) [1451] and (2012) results [1105]. The *dashed brown line* is the projected sensitivity of the XENON1T experiment [1452]. The *colour code* shows the with $P>0.2$ (*red*), $0.1<P<0.2$ (*orange*) $0.01<P<0.1$ (*green*) and $0.001<P<0.01$ (*blue*). Note, however, that the relic density constraint is not imposed here

Several experiments have been taking data, some claiming potential signals compatible with the detection of a WIMP. This includes DAMA [1101] which observes an annual modulation, CoGeNT [1444], CRESST [1102] and CDMS-Si [1104] which also have found signals that would be compatible with DM in the range 5–30 GeV. These observations are, however, in conflict with the negative search results by other collaborations, notably CDMS, Edelweiss [1445], XENON [1105] or LUX [1099]. The large ton scale detectors that are planned, such as XENON, should improve by more than one order of magnitude the current sensitivity, thus resolving the apparent conflict in SI results at low masses and probing a large number of DM models. See Fig. 156 for a comparison of the current limits with the expectations in the CMSSM. In particular, the case where the neutralino is a mixed gaugino/higgsino state is challenged by current limits as illustrated in Fig. 157 where $P=\mathrm{min}(f_h,1-f_h)$ and $f_h$ is the higgsino fraction. Finally COUPP [1446], KIMS [1447], Picasso [1448] (Xenon10 [1449]) have set limits on the spin-dependent interactions on protons (neutrons).

#### Indirect detection

In general, the goal of the on-going generation of indirect detection experiments sensitive to DM is:

1. to probe the vanilla “WIMP paradigm”, at least for particles of masses at the electroweak scale and characterised by s-wave annihilation cross sections $\langle \sigma v\rangle$.
2. to clarify some of the “anomalies” presently claimed.
3. in the case of independent detection (at colliders or direct detection), provide one or several cross-checks taking advantage of the multi-messenger characteristics of this detection strategy.

Concerning the first task, it is worth pointing out that for some channels Fermi-LAT has already reached the sensitivity to test this paradigm up to a few tens of GeV (dwarf spheroidals [1453, 1454], diffuse gamma-ray halo signal [1455]) or even more for the galactic center [1456].

In general, we expect that probing the $\sim$100 GeV mass scale will be within reach with a decade worth of data, see for example the forecasts in [1457]. Preliminary results from Fermi-LAT also comfort these expectations; see [1458]. Especially for candidates annihilating into leptons, such a goal seems also within reach of Planck, which probes DM energy deposition at early times via its impact on the reionisation (see e.g. [1459]).

Needless to say, if new states are below the TeV scale, these WIMP candidates are also in the right ball-park to be probed directly or indirectly by a future ILC, hence the complementarity of the two approaches.

The current generation of ground-based imaging atmospheric Cherenkov telescopes (IACTs) is less sensitive to theoretically preferred values of $\langle \sigma v\rangle$. Nonetheless, they are already more sensitive than Fermi-LAT to TeV-scale DM, see e.g. [1460], and with the future CTA they may probe not-yet-excluded regions of parameter space for viable particle physics models; see e.g. [1461]. Typically, the galactic center is among the most promising targets, provided that the DM distribution is comparable to expectations based on pure cold DM simulation or even enhanced as a consequence of baryons [1462]. Dwarf spheroidals have also been studied by IACTs see e.g. [1463, 1464] and show some potential for interesting complementary constraints, since they are affected by very different systematics.

Performances similar to Fermi-LAT (but more dependent on astrophysical modelling of cosmic ray transport) are expected by high-precision measurements of cosmic ray antimatter, most notably antiprotons and, possibly, anti-deuterons [1465]. Positrons are significantly sensitive to astrophysical backgrounds (see e.g. [1466]) and both their primary and their secondary fluxes show a larger dependence from source distribution (in space and time) as well as from the medium properties (e.g. their *E*-losses crucially depend on *B*-field and interstellar radiation fields). While they remain challenging for a *robust detection* of DM, they may be useful for cross-checks of tentative signals. AMS-02 and, concerning anti-deuterons, GAPS, are expected to achieve the needed precision and sensitivity for such competitive results.

It is mandatory to address caveat: ultimately, if sufficient statistics is accumulated, the main limitations will come from the degree of understanding of the astrophysical foregrounds, so that most of these projections must be taken with a grain of salt.

An example of the second type of goal has been provided in the recent past by the multi-messenger constraints on the DM interpretation of the PAMELA positron fraction “rise” (where the relevance of the point just made clearly manifested) or, at present, by the tentative hint for a $\sim$130 GeV “gamma-ray line” [1467]. For this kind of task, statistics helps a lot but it is clearly not enough. Cross-checks and tests with different techniques and possibly improved resolutions are needed. Fortunately, current (HESS) or planned (CTA) IACTs may provide such a tool. This is also an arena where the proposed satellite experiment Gamma-400 [1468] might contribute, thanks to its superior resolution (see e.g. [1469]).

The third possibility has been heavily discussed in recent years in the context of direct detection “anomalies” [1101, 1102, 1104, 1444]. If interpreted in terms of “light” DM, a wealth of indirect detection cross-checks can be thought of, see e.g. [1470, 1471]. We conclude by pointing out that especially in this context (cross-checking direct detection potential signals), neutrino signals from the centre of the Sun (and possibly the Earth) are of particular relevance. In fact, they probe a similar combination (albeit not equal!) of DM–baryon cross section and local density of DM as direct detection experiments. Significant advances are expected by the IceCube in its current configuration, including the Deep-core configuration (see e.g. [1472]). Further progress may also be possible if the R&D PINGU low-energy extension will be realised [1473] (the same would apply to comparable programmes in the Mediterranean sea such as those pursued within Km3Net, of course). Finally, it is worth pointing out that this is also one of the few ways to potentially detect indirectly p-wave annihilating WIMPs, since the equilibrium flux is only dependent on the DM scattering cross section.

### Dark matter at the ILC

The goal of colliders with regard to the DM issue is first to search for a new particle, stable at the collider scale, and as a second step to determine the microscopic properties of this particle. These can then be used to reconstruct DM observables such as the relic abundance (within a cosmological model), the DM annihilation cross section in the galaxy and of the DM scattering cross sections on nucleons, thus checking the self-consistency of DM interpretation of different signals and the compatibility of specific DM models with observations.

The issues that will be most relevant at the ILC will be influenced by the forthcoming results of new physics searches at the LHC and of DM searches in direct and indirect detection. At the LHC the generic DM signature consists of jets (and leptons) plus large MET. With this signature, it is highly non-trivial to then resolve the underlying theory as well as the nature of the DM candidate. For this one needs a precise determination of their properties such as masses, spins and couplings, as was shown in many specific models [1089, 1474, 1475]. This is where the ILC has an important role to play. Failing discoveries of new particles, the role of the ILC will be to search for the DM candidate as well as for other weakly interacting particles that might have escaped the LHC searches. Indeed the direct production of electroweak particles not only suffer from small rates at the LHC, but often feature a compressed spectra that can make their identification challenging. At the ILC new electroweak particles can easily be produced provided the centre-of-mass energy is sufficient to cross the mass threshold.

It might well be that the only kinematically accessible new particle at the first stage of the ILC is the DM particle itself. In this case DM radiative production can be used. The signal is a single high-energy photon, emitted from the incoming beam or from the exchanged particle, and missing energy. Effective operators that describe the interaction of electrons with DM particles can be used to parametrise the effect of new physics. In this model-independent approach, it has been shown that for DM annihilation cross section compatible with the relic abundance of DM, the cross section for radiative DM production at the ILC can be large enough to observe this process above the irreducible background from radiative neutrino production [49]. The electron and positron beam polarisations can be used to significantly enhance the signal and suppress the background from radiative neutrino production simultaneously [1476]. Furthermore the energy spectrum of the ISR photon can be exploited to extract information on the WIMP mass and cross section, at the per-cent level [49]. Similar conclusions were reached for radiative neutralino production in the MSSM [1477], distinguishong between models through a shape discrimination analysis of the photon energy spectrum which is affected by the particle exchange in *t*-channel [1478].

A measurement of the invisible width of the Higgs also provides a unique opportunity to determine the Higgs coupling to DM particles directly when $m_\mathrm{DM}< m_h/2$. This is an essential ingredient in determining the spin-independent direct detection cross section ($\sigma ^\mathrm{SI}$) in models dominated by Higgs exchange [470]. A refined upper limit on the invisible width will constrain the maximal allowed value for $\sigma ^\mathrm{SI}$ for light DM [456, 457].

Parameter determination in order to reconstruct DM observables and in particular the relic density amounts to determining the DM mass and its couplings, the mass of the particles exchanged in either the *t*-channel or the *s*-channel and the mass splittings between the DM and the new particles that can participate in coannihilation processes. Many studies have examined within the context of specific DM scenarios whether a high enough precision can be achieved so that a meaningful comparison with observables can be made [1089, 1479, 1480]. To illustrate what could be achieved we will consider the model most studied, a supersymmetric model with a neutralino LSP, and assume that some of the supersymmetric spectrum is kinematically accessible.

The measurements of the masses of the chargino and of the heavier neutralinos (e.g. through a threshold scan), together with the determination of their mass splitting with the LSP using the endpoints of the energy spectrum of the SM particle produced, together with the LSP in the decay of the heavier SUSY particle, allow a reconstruction of the four elements of the neutralino mass matrix. Moreover, since the $e^+e^-$ production cross sections of charginos and heavier neutralinos are sensitive to the gaugino/higgsino mixing they can provide crucial information on the nature of the LSP. In a scenario where only electroweakinos are accessible at the LHC and the ILC, it was shown that with the ILC measurements at the per-cent level or better, the value of ${\varOmega } h^2$ could be inferred with an uncertainty around 10 % [1089]. Of particular importance in this scenario is the need to get a lower bound on the mass of the heavy pseudoscalar to ensure that its contribution to DM annihilation is negligible [1481]. In other scenarios, where neutralino annihilation is strongly enhanced because the pseudoscalar exchange in the *s*-channel is nearly on resonance, a determination of the pseudoscalar mass to about 3 % and its width to 20 % – is required to infer the DM relic density at the 10 % level [1479]. For these measurements it might be necessary to run the ILC at energies above 1 TeV. When coannihilation processes play an important role, the mass splitting of the coannihilating particle with the LSP – for example the stau NLSP, must be measured at the per-cent level – which requires the measurement of masses at the few per-mille level [1479]. An issue that comes up is the impact of radiative corrections, which introduce more degrees of freedom from particles appearing only in higher order loops in the reconstruction of the neutralino mass matrix. Nevertheless, it was shown in [1204] that the parameters of the electroweakino sector could still be determined at better than the per-cent level and that indirect information on the mass of e.g. the pseudoscalar could be extracted.

In conclusion, despite intensive on-going efforts to search for DM at colliders and in astrophysics, the nature of the DM, even whether it is a new weakly interacting particle, is far from being solved. While near future results from the LHC are expected to provide crucial clues – even to discover new particles – it is clear that a high precision machine such at the ILC, designed with a high enough energy to probe most of the BSM spectrum, is needed for a verification of the DM paradigm.

## Summary

Exciting times in high-energy physics are just ahead. Discovering a Higgs boson at the LHC in exactly the range predicted by electroweak precision measurements confirms the successful strategy in particle physics of confronting direct discoveries with theoretical predictions of virtual effects in indirect searches. Within the current theoretical and experimental uncertainties the properties of the Higgs boson are in agreement with the predictions of the SM. Higher precision measurements are required to reveal whether nature can be described via the SM only or whether physics beyond the SM is required at some higher scale. The direct measurement of the total width of the Higgs within a few per-cent accuracy as well as the measurement of all Higgs couplings to fermions and bosons at the per-cent level are crucial to pin down the correct model of EWSB. In this context also high precision for the Higgs mass is required. With such an accuracy one gets a high sensitivity to virtual effects and even small traces of BSM physics become measurable. In order to really establish the BEH mechanism, also the Higgs self-couplings would be required. An accuracy of 10–20 % would constitute a first test of whether the Higgs potential provides indeed the required structure for the vacuum to generate the BEH mechanism. As we have discussed in this report, the full physics programme of the linear collider could perfectly well fulfil all these requirements.

Further footprints of new physics can be detected in the measurement of the electroweak couplings of the top quark with a unique precision at the linear collider. Exploiting asymmetries with polarised beams allows one to determine the electroweak top quark form factors at the per-cent level, that is, up to one order of magnitude more precise than the expectation from corresponding analyses at the LHC with $\sqrt{s}=14$ TeV and 300 fb$^{-1}$. Polarised beams are required to fit all factors simultaneously and to measure the asymmetry.

The highest precision in measuring the top-quark mass is mandatory to match the precision of the theoretical predictions with the expected experimental precision of the EWPO, which are strongly sensitive to the effects of virtual particles far beyond the kinematic limit. In order to uniquely relate the measured quantity to a well-defined mass scheme the top-quark measurement via a threshold scan is required and one can determine the mass of the top quark with an uncertainty of ${\varDelta } m_\mathrm{top}^{\overline{\mathrm{MS}}}=100$ MeV.

The LC has also an overwhelming potential for the discovery of further electroweak interacting particles and, in particular, of a cold DM candidate. The LC has potential to resolve even challenging scenarios, for instance, via applying the ISR method and to determine precisely the interaction character of DM candidate via applying polarised beams.

As shown in many reports [7–10, 17, 30, 45] as well as discussed here in detail, a Linear Collider with precisely tunable energy in the range of $\sqrt{s}=91$ GeV up to $\ge$1 TeV, high luminosity and polarised beams provides the required flexibility and precision to tackle these physics questions left by the LHC and is well prepared for even the ‘unexpected’. With the currently promising activities towards the realisation of the ILC in Japan one could even discuss the optimisation of the physics potential in HEP via a time of concurrent running [491] of the LHC and the LC. The described physics goals as well as not-yet-thought physics questions could be addressed by this option.

The physics world has changed on July 4, 2012 with the discovery of the Higgs boson at the LHC. Crucial milestones in particle physics are expected to be achieved in the near future with data in pairs from the upgraded LHC and from a future Linear Collider. In combination with astroparticle physics, a new era for pinning down the structure of our micro- as well as macrocosm has just started.

## Footnotes

1 As one example for a recent and comprehensive review of the LHC run-1 results, see [5] and references therein.

2 The quartic coupling will not be accessible either at the LHC or at an LC. Even at the high-luminosity large hadron collider (HL-LHC), i.e. the LHC at $\sqrt{=}14$ TeV but with a ten-fold increase in luminosity, there does not exist an analysis how to get access to this coupling.

3 Editors: K. Fujii, S. Heinemeyer, P.M. Zerwas^4^ Contributing authors: M. Asano, K. Desch, U. Ellwanger, C. Englert, I. Ginzburg, C. Grojean, S. Kanemura, M. Krawczyk, J. Kroseberg, S. Matsumoto, M.M. Mühlleitner, M. Stanitzki.

5 Keisuke Fujii, Sven Heinemeyer, Peter M. Zerwas.

6 Experimental results and simulations quoted in this introduction, as well as the large *corpus* of original theoretical studies in this field, are referenced properly in the review articles included subsequently in this section.

7 Jürgen Kroseberg.

8 However, the relation between the off-shell and on-shell couplings can be severely affected by new-physics contributions, in particular via threshold effects. In fact, such effects may be needed to give rise to a Higgs-boson width that differs from the one of the SM by the currently probed amount, see also the discussion in [122]. In this sense, these analyses currently provide a consistency test of the SM rather than model-independent bounds on the total width.

9 Keisuke Fujii: The presented materials were prepared for the ILC TDR physics chapter and for the Snowmass Higgs white paper in collaboration with the members of the ILC physics working group [140, 141] and the members of the ILC physics panel. The author would like to thank them for useful discussions, especially M. Peskin, Y. Okada, S. Kanemura., H. Haber, T. Barklow, A. Miyamoto, J. Tian, H. Ono, and T. Tanabe.

10 Marcel Stanitzki: The materials presented in this subsection were prepared for the CLIC Conceptual Design Report.

11 Sven Heinemeyer.

12 Analyses with complex parameters can be found in Refs. [214, 215] and references therein.

13 In the CMSSM we have four free parameters, $m_0$, $m_{1/2}$ and $A_0$ defined at the GUT scale, as well as $\tan \beta$ defined at the EW scale. Furthermore the sign of the $\mu$ parameter remains free. In the NUHM1 in addition the Higgs sector has one free parameter at the GUT scale, $m_H$. Details of the definition as well as the differences to mSUGRA scenarios can be found in, e.g., Ref. [253] and references therein.

14 A recent analysis from CMS using the Higgs decays to *ZZ* far off-shell yielded an upper limit on the total width about four times larger than the SM width [265]. However, these constraints on the total width rely on the assumption of the equality of the on-shell and off-shell couplings of the Higgs boson. The relation between those couplings can be severely affected by new physics contributions, in particular via threshold effects, which on the other hand would be needed to give rise to a Higgs-boson width that differs from the SM one by the currently probed amount, see the discusson in Ref. [122].

15 Shinya Kanemura.

16 The decay branching ratios of *h* can be different from the SM prediction at the next-to-leading order [284, 290–294].

17 We here give a comment on the radiative correction to the *hVV* couplings in the THDMs. Although the tree-level deviations in the *hVV* couplings are described by the factor $\sin (\beta -\alpha )$, these values can be modified at the one-loop level. In Ref. [284], the one-loop corrected *hZZ* vertex has been calculated in the softly broken $Z_2$ symmetric 2HDM. It has been found that for the fixed value of $\sin (\beta -\alpha )$, the one-loop corrections to the *hZZ* vertex are less than a few $\%$.

18 See also Ref. [374].

19 In the HTM, deviations in $h\bar{f} f$ couplings are small because of $v_{\varDelta } \ll v_\phi$.

20 Christoph Grojean, M. Margarete Mühlleitner.

21 Christoph Englert.

22 In fact, there are only two other possibilities to couple the SM to a hidden sector: *U*(1) mixing [461, 462] and mixing with a right-handed sterile neutrino [463, 464]. The Higgs-portal model is least constrained amongst these possibilities.

23 Ulrich Ellwanger.

24 Masaki Asano and Shigeki Matsumoto Both M. A. and S. M. would like to thank all the members of the ILC physics subgroup [140] for useful discussions.

25 As the other possibility, for example, the model decoupling the new gauge bosons have also been proposed [513, 514].

26 For the deviation of vector boson fusion process at ILC, see [535–537].

27 The Higgs mass is assumed to be 134 GeV, because this analysis has been performed before the discovery of the Higgs-like boson. The result of the analysis is not changed significantly even if the Higgs mass is set to be around 125 GeV.

28 Maria Krawczyk and Ilya Ginzburg: We are grateful to Filip Żarnecki for clarification of old analyses as well as to Jan Kalinowski.

29 Except if some additional symmetry is present in the model.

30 That is the $Z_2$ symmetric 2HDM where one Higgs doublet plays a role of SM Higgs field $\phi _S$, interacting with fermions as in Model I, with the SM-like Higgs boson *h* and another Higgs doublet $\phi _D$, having no v.e.v. The latter one contains four scalars $D, \,D^A,\,D^\pm$, the lightest among them *D* (analogue of *H* of 2HDM) can be DM particle, scalars $D^A$ and $D^\pm$ (analogue of *A* and $H^\pm$, respectively).

31 The recent analysis of the LHC data leads to constraints of the relative ${\mathscr {H}}tt$ coupling $\chi _t^{\mathscr {H}}$ [622].

32 The total width ${\varGamma }_H$ differs from the total width ${\varGamma }_A$ by the *W* / *Z* contribution, since $\chi ^A_V=0$.

33 At $\tan \beta \ll 1$ we obtain the strong interaction in the Higgs sector mediated by *t*-quarks, what is signalizing by the fact that the calculated in standard approach total widths of heavy *H*, *A* is becoming close to or even higher than the corresponding masses. Of course, in this case such tree-level estimates become inadequate. In the same manner at $\tan \beta >70$ corresponds to the region of a strong interaction in the Higgs sector mediated by *b*-quarks. We do not consider such scenarios.

34 Similar calculations given in [648] demonstrate this opportunity for a 2HDM version $B_{hu}$.

35 Authors: Frank Simon, Peter Uwer, Kiyo Yuichiro.

36 Assuming the *W*-boson and *b*-quarks as stable or long-lived particles.

37 Editors: S. Heinemeyer, D. Wackeroth Contributors: A. Denner, S. Dittmaier, A. Freitas, S. Godfrey, N. Greiner, M. Grünewald, A. Hoecker, R. Kogler, K. Mönig, M. Schmitt, D. Stöckinger, G. Weiglein, G. Wilson, L. Zeune.

38 Graham Wilson

39 We refer in this section particularly to the ILC which has a number of advantages over other proposed facilities, notably the ability to polarise both beams, to run in an optimised fashion at a variety of centre-of-mass energies, and with a good quality luminosity spectrum.

40 The literature from the LEP2 era usually refers to these methods as “direct reconstruction”.

41 Ansgar Denner, Stefan Dittmaier.

42 Sven Heinemeyer, Georg Weiglein, Lisa Zeune.

43 An updated version of HiggsBounds became available at http://higgsbounds.hepforge.org after this study was completed.

44 Ayres Freitas.

45 For a review, see [841].

46 This estimate can be made more precise only after aforementioned calculations have been completed.

47 Klaus Moenig.

48 Sven Heinemeyer, Georg Weiglein.

49 Sven Heinemeyer and Georg Weiglein.

50 We are not aware of any such estimate in other NPM.

51 Andreas Hoecker, Roman Kogler, Martin Grünewald.

52 Dominik Stöckinger.

53 Most of the points are ruled out by LHC searches for coloured particles. However, for our purposes only the weakly interacting particles are relevant, and these are not excluded.

54 The following conclusions are neither very sensitive to the actual $\tan \beta$ value nor to the actual value of the deviation ${\varDelta } a_\mu$.

55 Nicolas Greiner.

56 Nicolas Greiner, Sven Heinemeyer, Doreen Wackeroth.

57 Stephen Godfrey.

58 Editors: H. Baer, M. Battaglia, J. Kalinowski Contributors: A. Arbey, P. Bechtle, A. Bharucha, F. Brümmer, S.Y. Choi, A. Freitas, J. Heisig, J. List, F. Mahmoudi, G. Moortgat-Pick, W. Porod, S. Porto, K. Rolbiecki H.B. would like to thank D. Mickelson and A. Mustafayev for providing several figures.

59 In this study, the authors implement the BG fine-tuning measure applied to 19 uncorrelated parameters in the pMSSM which is assumed valid up to a scale ${\Lambda } \simeq 20$ TeV. The ${\Lambda } =20$ TeV scale induces an additional factor of 3 in the fine tuning evaluation.

60 Analyses of electric dipole moments strongly suggest that ${\textit{CP}}$ violation in the higgsino sector will be very small in the MSSM if this sector is non-invariant at all [1201, 1202].

61 Note that S2 corresponds to S3 in Ref. [1204].

62 Complete expressions are usually used for phenomenological investigations.

63 Editors: G. Belanger, K. Olive Contributors: Y. Mambrini, P. Serpico.
